# Copper(I)-Catalyzed Synthesis of Unsymmetrical All-Carbon Bis-Quaternary Centers at the Opposing α-Carbons of Cyclohexanones

**DOI:** 10.1021/acs.orglett.2c01890

**Published:** 2022-06-29

**Authors:** Joshua A. Malone, Satish Chandra Philkhana, Jacob R. Stepherson, Fatimat O. Badmus, Frank R. Fronczek, Rendy Kartika

**Affiliations:** Department of Chemistry, Louisiana State University, 232 Choppin Hall, Baton Rouge, Louisiana 70803, United States

## Abstract

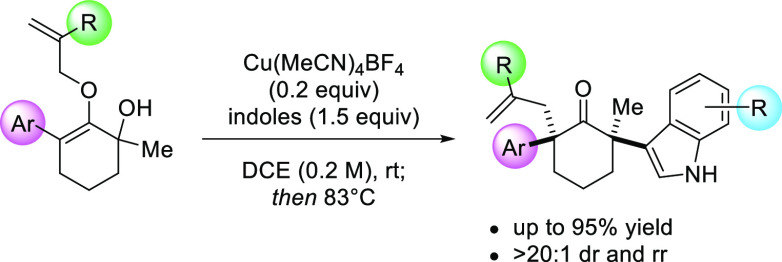

We describe a new synthetic reaction that generates all-carbon bis-quaternary centers at the opposing side of α-carbons in cyclohexanone with four different substituents in a controlled manner. Catalyzed by Cu(MeCN)_4_BF_4_ salt, this chemistry is proposed to proceed via an intermediacy of unsymmetrical *O*-allyl oxyallyl cations, which undergo a sequence of regioselective nucleophilic addition with substituted indoles and diastereoselective Claisen rearrangement in a single synthetic operation. The stereochemical outcome of the products features the *cis* diastereorelationship between the two aryl groups at the α,α′-positions.

A trend in drug discovery has progressively shifted toward the exploration of chemical structures with stereocenters.^1^ As opposed to flat aromatic compounds that tend to exhibit low solubility and bioavailability as a result of π-stacking interactions,^2^ molecules with increasing fractions of sp^3^-hybridized carbon atoms have been shown to offer more advantageous biophysical properties.^3^ Moreover, drug candidates that contain high counts of sp^3^-hybridized carbons are more likely to exhibit effective and selective binding to therapeutic proteins, including those that are difficult to target.^4^ A particular type of stereochemical systems that have gained attentions is the quaternary centers. Quaternary centers are carbon atoms that are covalently bound to four other carbon atoms at their sp^3^ tetrahedral vertices. Prominently featured in natural products, quaternary centers have become attractive structural motifs for drug discovery.^5^

One limitation that has prevented broader applications of quaternary centers in drug discovery can be attributed to the challenges associated with the synthesis of these sterically congested systems.^6^ Nonetheless, there have been efforts to develop synthetic reactions that produce quaternary centers, particularly at the α-carbon of carbonyl compounds.^7^ A carbonyl system that has been scrutinized is unsymmetrical ketones that possess two similarly acidic α-hydrogens, *viz*. **1**. The α-quaternarization of this motif could be challenging, as such a successful transformation to substituted ketones **2** would rely on judiciously designed elements to control regioselectivity. Recent methodologies that address this synthetic undertaking can be found, for instance, in the elegant work of Stoltz, who developed an extensive repertoire of transition-metal-catalyzed decarboxylative allylic alkylation reactions.^8^

Despite these profound advancements, examples of the functionalization of simple cyclic ketones at the opposing side of α-carbons with four different substituents to produce unsymmetrical bis-quaternary centers are scarce. Conceptually, this synthetic endeavor is challenging as barriers toward regioselectivity and diastereoselectivity must be regulated to produce a single isomeric product. Without these controls, bis-quaternarization of monosubstituted cyclohexanone **1** to α,α′-bis-quaternary ketones **3** would result in multiple regioisomers along with their respective diastereomers. The difficulty and complexity to create these bis-quaternary centers are evidenced.^8c,9^ For instance, Yamaguchi demonstrated bis-quaternization of diphenyl ketone **4** with a mixture of GaMe_3_, *n*-BuLi, and (chloroethynyl)triethylsilane. This reaction produced *meso* ketone **5** with modest diastereoselection.^9b^ Another precedent was conveyed by Stoltz, who applied a tandem enantioselective decarboxylative allylic alkylation that transformed substrate **6** to bis-quaternary ketone **7**. This product was isolated in 72% yield with 4:1 dr, favoring the *C*_2_-symmetric diastereomer.^8c^

In this paper, we convey a new synthetic method to install bis-quaternary centers at the α,α′-carbons of cyclic ketones with four different substituents while managing both regioselectivity and diastereoselectivity elements, mediated by our oxyallyl cation technologies (Scheme 1).^10^ Schematically, our proposed reaction began with α-hydroxy enol ether **8** that were decorated with three different groups from simple 1,2-diketone. Ionization of this substrate would generate protected oxyallyl cation **9a**.^10a^ The ensuing capture of this electrophilic species by nucleophiles could be performed in a regioselective manner, thus installing the first α-quaternary center.^10b^ To construct the second α-quaternary center, reaction intermediate **9b** underwent *in situ* diastereoselective migration of the protecting group from the oxygen atom to the opposing α-carbon.

**Scheme 1:**
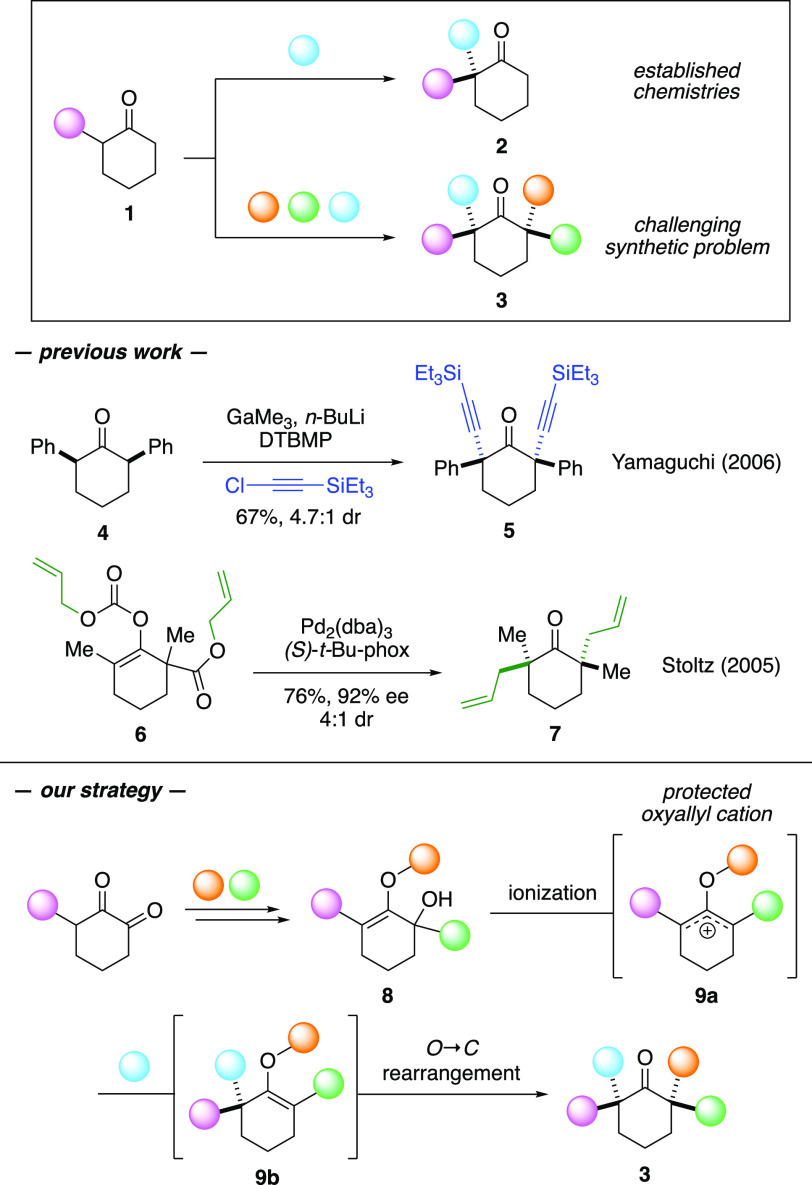
Synthesis of Bis-Quaternary Centers at the α-Positions of Ketones

Our initial studies are depicted in Scheme 2. To facilitate the intended O → C migration, we envisioned the utility of the Claisen rearrangement,^11^ which would require the protection of oxyallyl cation as an *O*-allyl ether. A suitable model substrate was realized in α-hydroxy *O*-allylenol ether **10**, which contained three out of the four intended substituents, *i.e*., methyl, phenyl, and allyl groups. This compound was easily prepared in just two simple steps from 3-phenylcyclohexane-1,2-dione upon treatment with allyl bromide and K_2_CO_3_, followed by addition of methylmagnesium bromide. The fourth substituent was incorporated by exposing substrate **10** to indole and catalytic Py·TfOH in toluene at room temperature to introduce α-quaternary center **12** in 93% yield. This reaction was assumed to occur via unsymmetrical *O*-allyl oxyallyl cation intermediate **11** that was captured by indole regioselectively at the α-methyl position.^10b^ To generate the second α-quaternary center, compound **12** was heated in toluene at reflux to promote the Claisen rearrangement, which produced α,α′-bis-quaternary ketone ***cis*****-13** and ***trans*****-13** as a 4.1:1 mixture of diastereomers with a combined yield of 96%. The relative stereochemistry of the major diastereomer ***cis*****-13** was confirmed by the X-ray structure,^12^ in which the phenyl and indole groups were both positioned in the axial direction.

**Scheme 2:**
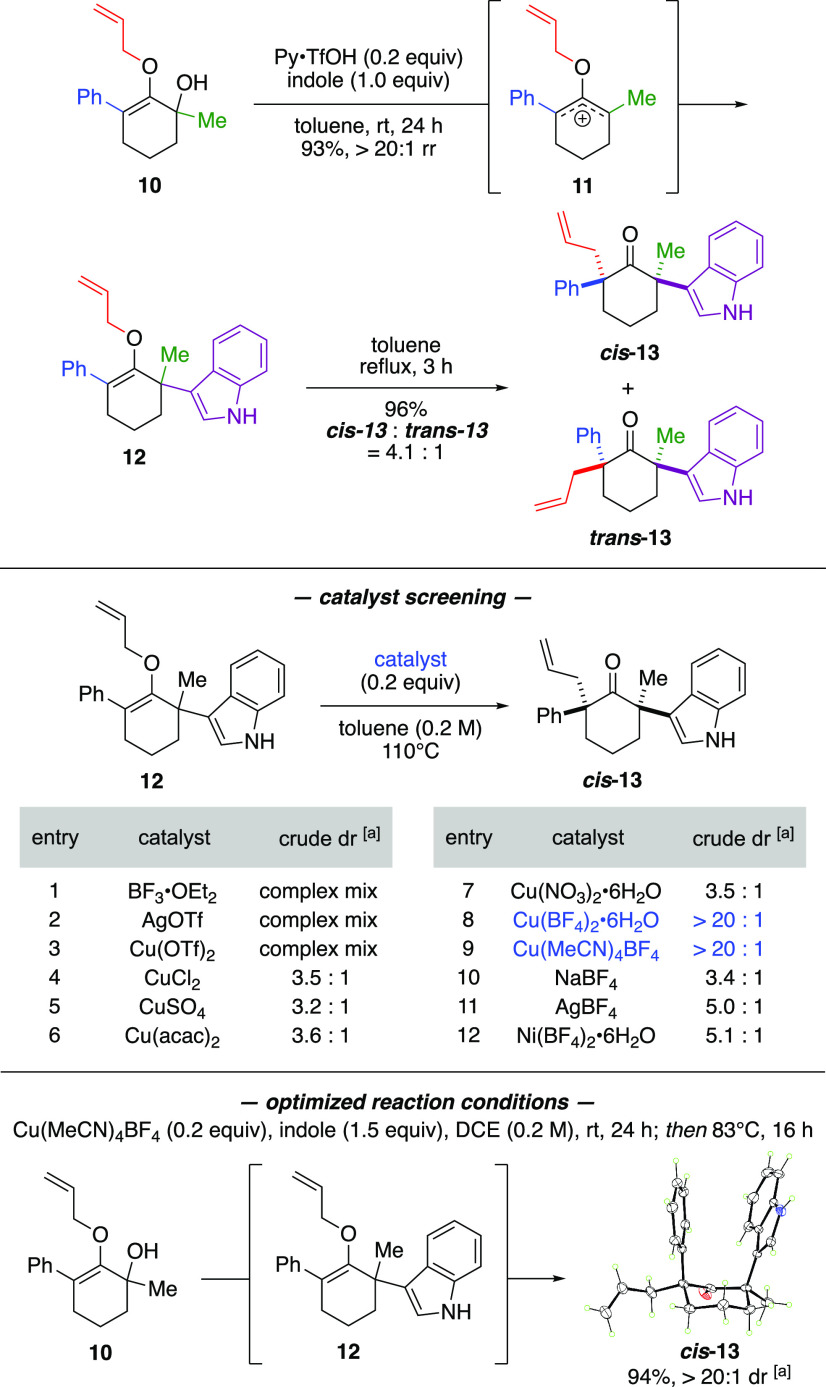
Proof-of-Concept and Reaction Optimization Diastereomeric ratio (dr) was determined by ^1^H NMR of the crude reaction mixture.

Attempts to improve diastereoselectivity in the Claisen rearrangement was then carried out by screening various Lewis acid catalysts that could hypothetically serve either as an oxyphilic activator^13^ or as a π-complex activator^14^ to compound **12**. As shown in entries 1–3, BF_3_·OEt_2_, AgOTf, and Cu(OTf)_2_ caused decomposition. The effects of counteranion were evaluated through the use of copper salts, such as CuCl_2_, CuSO_4_, Cu(acac)_2_, and Cu(NO_3_)_2_·6H_2_O (entries 4–7). Only a marginal improvement was noted. Nonetheless, we observed a remarkable enhancement in diastereoselectivity with either Cu(BF_4_)_2_·6H_2_O or Cu(MeCN)_4_BF_4_ catalysts. In fact, these reactions produced α,α′-bis-quaternary ketone ***cis*****-13** as a single diastereomer. To affirm that copper(I) and (II) species were responsible to drive the diastereocontrol, we evaluated NaBF_4_, AgBF_4_, and Ni(BF_4_)_2_·6H_2_O. Such catalysts did not yield consequential induction (entries 10–12). From the fundamental viewpoint, these screening results showcased a new mode of reactivity to dictate diastereoselectivity in the Claisen rearrangement involving simple cyclohexanone systems, in which the stereocontrol elements were provided by a catalyst and an α-quaternary center.

We envisioned that the Lewis acidity of copper(I) and -(II) tetrafluoroborate could be also exploited to ionize α-hydroxy *O*-allylenol ether **10** to unsymmetrical *O*-allyl oxyallyl cation **11**. Upon regioselective nucleophilic capture by indole, the Claisen rearrangement of the emerging α-quaternary center **12** could be induced *in situ* by the same catalyst at an elevated reaction temperature to produce α,α′-bis-quaternary ketone ***cis-*****13** in a single synthetic protocol. Indeed, we were able to achieve this cascade transformation using catalytic Cu(MeCN)_4_BF_4_ salt in dichloroethane, from which product ***cis-*****13** was isolated in 94% yield as a single diastereomer.^15a^ Detailed reaction optimization is discussed in the Supporting Information.

With the optimized conditions in hand, we evaluated the scope of reactions, starting with substituted indoles (Scheme 3).^15b^ In these examples, our reactions produced the corresponding α,α′-bis-quaternary ketone products with >20:1 dr. Commencing with electron-rich 5-methoxy-, 6-benzyloxy-, 5-*p*-methoxyphenyl (PMP)-, and 5-hydroxy-substituted indoles, ketones **14a**–**14d** were generated in 42–88% yields. Halogen-containing indoles were found to be compatible. For example, the use of 5-iodo-, 7-bromo-, and 5,6-dichloro-substituted indoles produced the respective ketones **14e**–**14g** in good yields. Electron-deficient methyl-5-carboxylate indole and protected *N*-methylindole could be also employed to furnish ketones **14h** and **14i** in 58% and 73% yields, respectively.

**Scheme 3:**
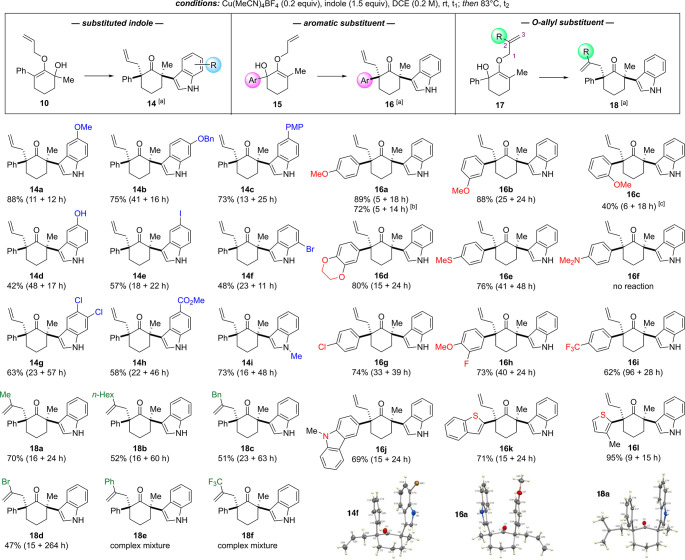
Scope of Reactions Isolated yield after column chromatography. ^1^H NMR analyses of the crude mixture indicated >20:1 dr. The reaction was performed on a 1 g scale. Crude dr of compound **16c** could not be determined due to the complex mixture.

The nature of the aromatic substituent at the α-carbon was then examined using a series of α-hydroxy *O*-allylenol ether substrates **15**. While methoxyphenyl at the *para-* and *meta-*positions produced ketones **16a** and **16b** in 89% and 88% yields, respectively, a substantial erosion in yield of product **16c** was noted with the *ortho-*counterpart. Other electron-donating substituents, such as benzodioxane and thiophenolate ether, produced the respective ketones **16d** and **16e** in excellent yields. Interestingly, substrate bearing a dimethylaminophenyl group failed to react, leading only to a recovery of the starting material. In this case, the amino group might have sequestered the copper catalyst. We surveyed halogen and electron-withdrawing groups, such as *p*-chlorophenyl, *m*-fluoro-*p*-methoxyphenyl, and *p*-trifluoromethylphenyl. These afforded products **16g**–**16i** in good yields. Heteroaromatic substituents, such as *N*-methyl carbazole, benzothiophene, and 3-methylthiophene, were well tolerated to provide ketones **16j**–**16l** in 69–95% yields.

The substituent effects in the *O*-allyl moiety were also examined using substrates **17**. In this study, methyl, *n*-hexyl, and benzyl groups were introduced at the internal C2 position, which furnished α,α′-bis-quaternary ketones **18a** to **18c** in satisfactory yields as a single diastereomer. The bromo variant was also tolerated by the reaction conditions to generate product **18d** in 47% yield, but the Claisen rearrangement required a prolonged reaction time. We then proceeded to the phenyl group. While the creation of the α-indolyl bearing quaternary center proceeded in this case, the Claisen rearrangement to install the second α-quaternary center in product **18e** unexpectedly produced complex mixtures upon heating. A similar phenomenon was also observed with an electron-withdrawing trifluoromethyl group in which the Claisen rearrangement led to decomposition instead of yielding ketone **18f**.

Scheme 4 depicts a series of reactions to gather some mechanistic insights of this reaction. The copper(I) species was the active oxidation state of the catalyst. While our reactions were performed in typical benchtop settings, treatment of either substrates **10** and **12** with Cu(MeCN)_4_BF_4_ in an oxygen-free glovebox also furnished the corresponding product ***cis*****-13** in excellent yields as a single diastereomer. The intermolecular interaction between the copper catalyst and the Claisen rearrangement substrate appeared to be rather labile as it was affected by simple steric changes in the α-aliphatic region. When effectively bound to α-methyl substrate **10**, the catalyst would provide a strong governance toward the *cis* stereoselectivity. Nonetheless, replacement of the α-methyl group with ethyl in α-hydroxy *O*-allylenol ether **19** rendered the copper-catalyzed Claisen rearrangement nondiastereoselective. The O → C allyl migration appeared to have proceeded via an intramolecular Claisen rearrangement, as opposed to the alternative transition-metal-catalyzed dissociative mechanism,^16^ based on the following key experiments. A reaction involving an equivalent amount of α-indolyl quaternary centers **21** and **22a** produced a mixture of α,α′-bis-quaternary ketones **16a** and **22b**. None of the crossover products were detected in the crude reaction mixture by ^1^H NMR analyses. The allyl migration itself was found to be stereospecific, as (*Z*)-deuterated substrate **23a** generated the corresponding ketone **23b** as a single diastereomer with the allyl group transpositioned at the γ-carbon.^17^

**Scheme 4:**
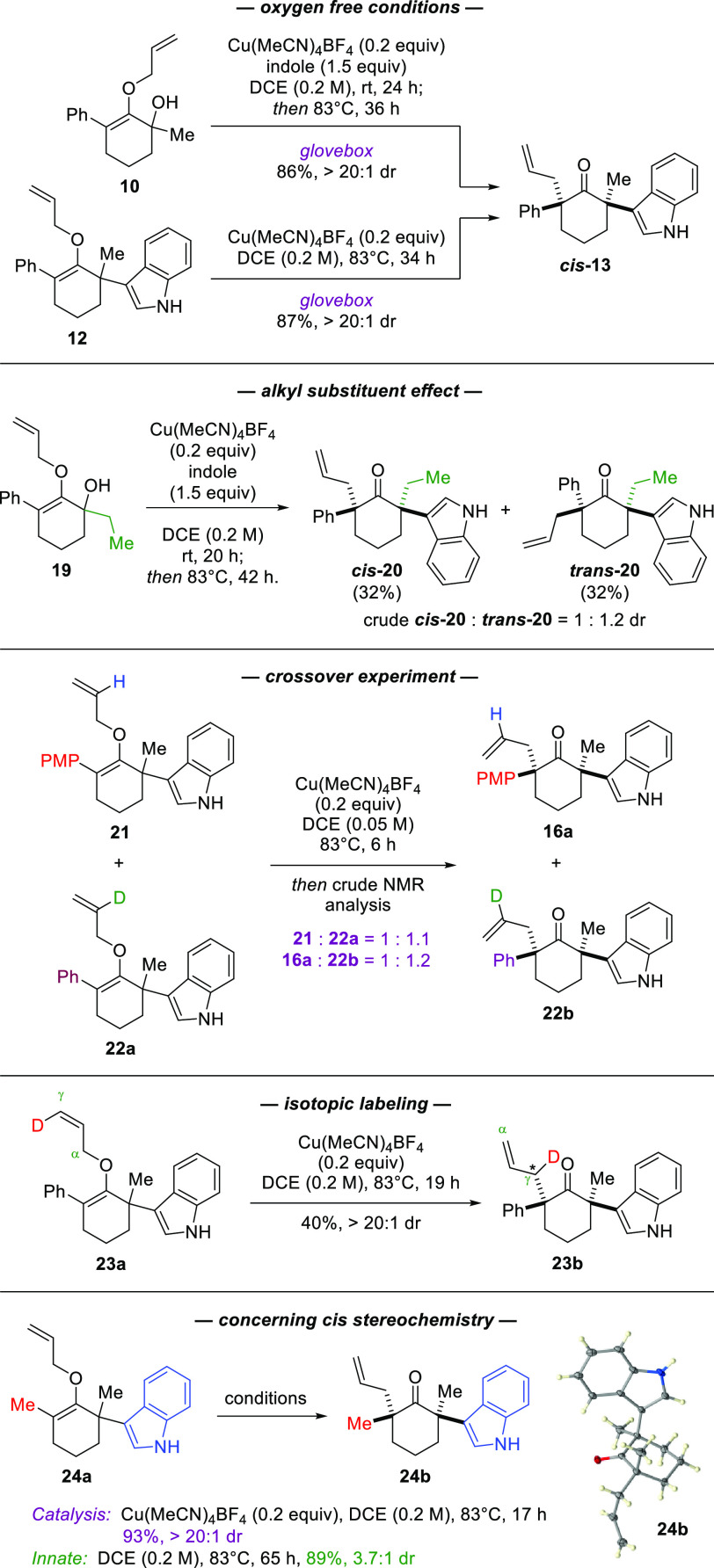
Experiments to Gather Mechanistic Insights

The relative stereochemistry of the α,α′-bis-quaternary centers in many of our products were confirmed using X-ray crystallography.^12^ This analysis also revealed a peculiar conformation in which the *cis* aromatic groups were both placed in the axial position, thus allowing orientation of these rings within a reasonable distance for possible π-stacking interactions.^18^ While the origin of this diastereochemical outcome remained unclear, we deduced that the presence of both aryl groups was not essential for the noted stereoselectivity. As corroborated in α-indolyl substrate **24a**, replacement of the phenyl substituent at the opposing side of the α-carbon with a methyl group led to the Claisen rearrangement under the catalytic conditions to produce monoaryl-substituted α,α′-bis-quaternary ketone **24b** with a *cis* stereochemical outcome as confirmed by X-ray structure analyses. In contrast, the background thermal rearrangement furnished the product with an innate 3.7:1 diastereomeric ratio.

In conclusion, we have showcased a new method to synthesize unsymmetrical ketones bearing all-carbon bis-quaternary stereocenters at the α,α′-positions. Our chemistry converted a simple and readily accessible substrate in α-hydroxy *O*-allylenol ethers to highly complex, stereochemically elaborate α,α′-bis-quaternary ketones in a single synthetic reaction. Cu(MeCN)_4_BF_4_ catalyst was found to be uniquely effective for this transformation. Further studies are ongoing in our laboratory. The results will be reported in due course.

## Supporting Information Available

Experimental procedure and characterization data (PDF)
FAIR data, including the primary NMR FID files, for compounds ***cis*****-13**, ***trans*****-13**, **14a**–**14i**, **16a**–**16e**, **16g**–**16l**, **18a**–**18d**, ***cis*****-20**, ***trans*****-20**, **22b**, **23b**, and **24b** (ZIP)

## Accession Codes

CCDC 2072233–2072242 and 2072247–2072248 contain the supplementary crystallographic data for this paper. These data can be obtained free of charge via www.ccdc.cam.ac.uk/data_request/cif, or by emailing data_request@ccdc.cam.ac.uk, or by contacting The Cambridge Crystallographic Data Centre, 12 Union Road, Cambridge CB2 1EZ, UK; fax: +44 1223 336033.

## Dedication

This work is dedicated to Prof. Emerita Carol M. Taylor for her mentorship and friendship.
