# Supplementary material for: Copper(I)-Catalyzed Synthesis of Unsymmetrical All-Carbon Bis-Quaternary Centers at the Opposing α-Carbons of Cyclohexanones
Source: Org Lett. 2022 Jun 29;24(26):4810–5. doi: 10.1021/acs.orglett.2c01890 (PMC9490817; doi:10.1021/acs.orglett.2c01890)

Copper(I)-Catalyzed Synthesis of Unsymmetrical All-Carbon *bis*-Quaternary Centers at the Opposing α-Carbons of Cyclohexanones

Joshua A. Malone,† Satish Chandra Philkhana,† Jacob R. Stepherson, Fatimat O. Badmus, Frank R. Fronczek, and Rendy Kartika*

Department of Chemistry

232 Choppin Hall

Louisiana State University

Baton Rouge, LA 70803, USA

SUPPORTING INFORMATION

TABLE OF CONTENT

1. General Information……………………………………………………………. S-3

2. Reaction Optimization ……………………………………………………………S-4

3. Characterization of Products in Scheme 2 and Table 1 ……………………….……. S-7

4. Characterization of Products in Scheme 3 ……………………………………..……. S-10

5. Characterization of Products in Scheme 4 ……………………………………..……. S-40

6. Synthesis and Characterization of Substrates …….…….……………………………... S-55

7. X-Ray Crystallography Data ……….…………………………………………………. S-87

8. ^{1}H And ^{13}C NMR Spectra …………………….……….……….…….…………. S-229

GENERAL INFORMATION

Unless otherwise noted, all materials were used as received from commercial suppliers without further purification. All anhydrous reactions were performed using oven-dried glassware, which was then cooled under vacuum and purged with nitrogen gas. Tetrahydrofuran (THF), dichloromethane (CH_2Cl_2), acetonitrile, toluene, diethyl ether (Et_2O), dimethylformamide (DMF), and *n*-hexanes were filtered through activated silica or 3Å molecular sieves under argon contained in a Solvent Purification System. All reactions were monitored by analytical thin layer chromatography (TLC Silica Gel 60 F_{254}, Glass Plates) and analyzed with 254 nm UV light and / or anisaldehyde – sulfuric acid or potassium permanganate treatment. Column chromatography was completed using silica gel (32-63μ).

Unless otherwise noted, all ^1H and ^{13}C NMR spectra were recorded in $CDCl_3$ using a Bruker Ascend 400 spectrometer operating at 400 MHz for ^1H and 100 MHz for ^{13}C or Bruker Ascend 500 spectrometer operating at 500 MHz for ^1H and 125 MHz for ^{13}C. Chemical shifts (δ) are reported in ppm relative to residual $CHCl_3$ as an internal reference (^1H: 7.26 ppm, ^{13}C: 77.00 ppm). Coupling constants (*J*) are reported in Hertz (Hz). Peak multiplicity is indicated as follows: s (singlet), d (doublet), t (triplet), q (quartet), p (pentet), (septet), h (heptet), b (broad), and m (multiplet). FT-IR spectra were recorded on Bruker Tensor 27 spectrometer and OPUS 6.5 Data Collection Program, and absorption frequencies were reported in reciprocal centimeters (cm^{-1}). High Resolution Mass Spectrometry (HRMS) analyses were performed by the Louisiana State University Mass Spectrometry Facility. X-ray structure analyses were performed by the Louisiana State University X-ray Structure Facility.

REACTION OPTIMIZATION

Table S1. Reaction Optimization

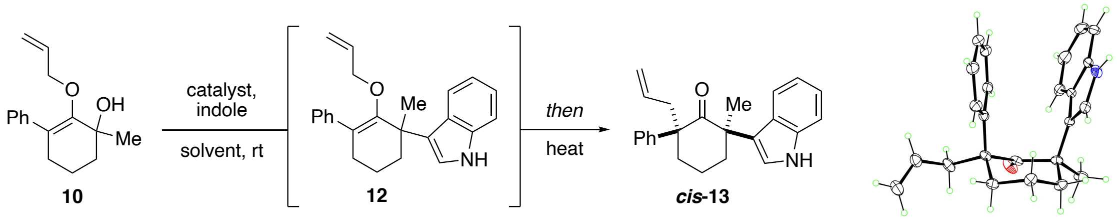

entry	catalyst	catalyst equiv	solvent	indole equiv	heat temp (°C)	reaction time (h) rt	reaction time (h) heat	yield (%) [a]	dr [b]
1	$Cu(BF_4)_2$•$6H_2O$	0.2	toluene	1.5	110	2	4	60	20 : 1
2	$Cu(MeCN)_4BF_4$	0.2	toluene	1.5	110	184	6	92	13 : 1
3	$Cu(BF_4)_2$•$6H_2O$	0.2	DCE	1.5	83	1	22	31	20 : 1
**4**	**$Cu(MeCN)_4BF_4$**	**0.2**	**DCE**	**1.5**	**83**	**24**	**16**	**94**	**20 : 1**
5	$Cu(MeCN)_4BF_4$	0.1	DCE	1.5	83	28	20	86	20 : 1
6	$Cu(MeCN)_4BF_4$	0.05	DCE	1.5	83	28	38	92	20 : 1
7	$Cu(MeCN)_4BF_4$	0.2	DCE	1.1	83	24	21	74	20 : 1
8	$Cu(MeCN)_4BF_4$	0.2	DCE	1.5	83	78	62	60 [c]	20 : 1
9	$Cu(MeCN)_4OTf$	0.2	DCE	1.5	83	0.5	2	complex mixture [d]	-
10	$Cu(MeCN)_4PF_6$	0.2	DCE	1.5	83	16	48	complex mixture [d]	-

[a] Isolated yield after column chromatography. [b] ^{1}H NMR of the crude reaction mixture was used to determine diastereomeric ratio. [c] 4 Å molecular sieves were added into the reaction mixture. [d] Complex mixture was observed as the reaction was warmed to 83 °C.

With the identification of both copper (I) and (II) tetrafluoroborate as viable catalysts for the Claisen rearrangement, we proposed that the Lewis acidity of these salts could be feasibly exploited to ionize α-hydroxy *O*-allylenol ether **10** to unsymmetrical *O*-allyl oxyallyl cation **11**. Upon regioselective nucleophilic addition by indole, the Claisen rearrangement of the emerging α-quaternary center **12** could be induced *in situ* by the same catalyst to produce α,α'-*bis*-quaternary ketone **13** in a single synthetic operation. Our optimization efforts in this endeavor are summarized in Table S1. As shown in entries 1-4, we subjected substrate **10** to both $Cu(BF_4)_2$•$6H_2O$ or $Cu(MeCN)_4BF_4$ catalysts in the presence of indole at room temperature to install the first α-quaternary center. Upon a complete consumption of the starting material as

monitored by TLC, the reaction mixtures were then warmed at reflux to promote the Claisen rearrangement. Two different solvents, *i.e.* toluene and dichloroethane, were employed in these initial experiments. In toluene (entries 1-2), we noted considerably higher diastereoselectivity of the resulting ketone ***cis*-13** using $Cu(BF_4)_2$•$6H_2O$ than that produced by $Cu(MeCN)_4BF_4$; however, the latter catalyst led to a much higher isolated product yield. Another interesting observation was that the generation of the first α-quaternary center **12** with $Cu(MeCN)_4BF_4$ required a much longer reaction time compared to that of $Cu(BF_4)_2$•$6H_2O$. This stark difference in the rate of reaction was suspected to have been attributed to the relative insolubility of $Cu(MeCN)_4BF_4$ in toluene. As the reaction solvent was replaced from toluene to dichloroethane (entries 3-4), $Cu(MeCN)_4BF_4$ was observed to outperform $Cu(BF_4)_2$•$6H_2O$ dramatically. Not only did the $Cu(MeCN)_4BF_4$ catalyst furnish the target ketone ***cis*-13** in 94% isolated yield with improved diastereoselectivity of >20:1, but the reaction time for the installation of the first α-quaternary center also drastically shortened to just 24 hours. Furthermore, ^{1}H NMR analyses of the crude reaction mixtures indicated a very clean transformation, as no other organic byproducts were formed beside the target ketone ***cis*-13** and residual unreacted indole.

Our screening studies continued with investigations to identify the optimal molar equivalences for both the copper salt and indole (entries 5-7). While our reaction was initially performed with 0.2 equiv of the catalyst, reducing the molar amount of $Cu(MeCN)_4BF_4$ to 0.05 equiv did not affect the yield or diastereoselectivity although the prolonged rate of reaction in the Claisen rearrangement was noticed. As shown in entry 7, we also examined the amount of indole by reducing from 1.5 to 1.1 equiv, which noticeably eroded the product yield to 74%. The water byproduct formed during ionization of α-hydroxy *O*-allylenol ether **10** by the copper catalyst appeared to provide beneficial effects. As we performed this experiment under completely

anhydrous conditions in the presence of 4 Å molecular sieves, the yield of this reaction dropped significantly to 60% (entry 8). Substantially lengthened reaction times in both nucleophilic addition and the Claisen rearrangement were also noted. Experiments depicted in entries 9 and 10 once again confirmed the necessity for the tetrafluoroborate counter anion through the use of $Cu(MeCN)_4PF_6$ and $Cu(MeCN)_4OTf$ salts. While these catalysts transformed substrate **10** to α-quaternary center **12**, this reaction intermediate unexpectedly decomposed to produce complex mixtures upon heating.

CHARACTERIZATION OF PRODUCTS IN SCHEME 2 AND TABLE 1

(±)-(2*R*,6*R*)-2-allyl-6-(1*H*-indol-3-yl)-6-methyl-2-phenylcyclohexan-1-one (*cis*-13) and (±)-(2*S*,6*R*)-2-allyl-6-(1*H*-indol-3-yl)-6-methyl-2-phenylcyclohexan-1-one (*trans*-13)

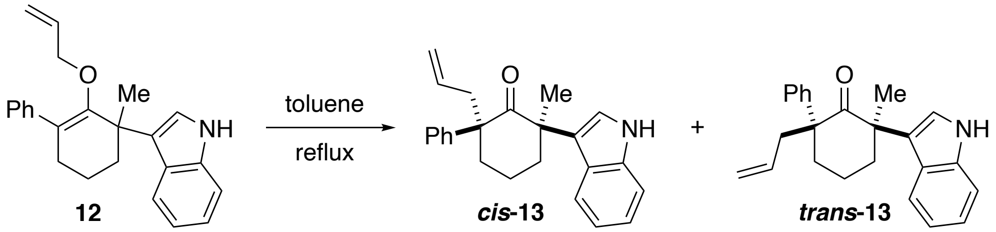

Procedure: α-Indolyl allylvinyl ether **12** (55 mg, 0.149 mmol) was dissolved in toluene (0.7 mL, 0.2 M). The mixture was then heated to 110 °C in a preheated oil bath for 3 hours. Upon complete Claisen rearrangement as monitored by TLC, the crude reaction mixture was concentrated under vacuum and purified by column chromatography using 100% hexanes to 50:50 hexanes : CH_2Cl_2 to afford compound ***cis*-13** in 77% yield (42 mg, 0.122 mmol) as a white solid and ***trans*-13** in 19% yield (10 mg, 0.029) as a colorless oil. ^{1}H NMR analysis of the crude reaction mixture indicated 4.1:1 dr (***cis*-13** : ***trans*-13**).

Product ***cis*-13**

Rf: 0.6 in 50:50 (Hexanes : CH_2Cl_2)

^{1}H NMR: (500 MHz, $CDCl_3$) δ = 7.70 (d, *J* = 7.7 Hz, 1H), 7.47 (bs, 1H), 7.10 – 7.03 (m, 3H), 6.83 (d, *J* = 8.5 Hz, 2H), 6.69 – 6.67 (m, 2H), 6.59 (t, *J* = 7.7 Hz, 2H), 5.34 – 5.26 (m, 1H), 4.89 – 4.85 (m, 2H), 2.63 – 2.59 (m, 3H), 2.58 – 2.43 (m, 2H), 1.92 – 1.89 (m, 1H), 1.88 – 1.79 (m, 2H), 1.55 (s, 3H).

^{13}C NMR: (125 MHz, $CDCl_3$) δ = 212.6, 139.6, 136.4, 134.8, 126.7, 126.2, 125.7, 125.3, 121.6, 121.3, 120.8, 119.1, 118.3, 117.4, 110.6, 55.2, 49.0, 46.7, 39.2, 33.5, 27.4, 18.7.

IR: *f* (cm^{-1}) = 3373, 2955, 2861, 1681, 1460, 1421, 1343, 1245, 1105, 917.

HRMS: (ESI-TOF) *m/z*: $(M+H)^+$ = 344.2009 calculated for $C_{24}H_{26}NO$; Found 344.2018.

X-Ray Structure: Crystal growth was performed via slow evaporation using 5% CH_2Cl_2 in hexanes.

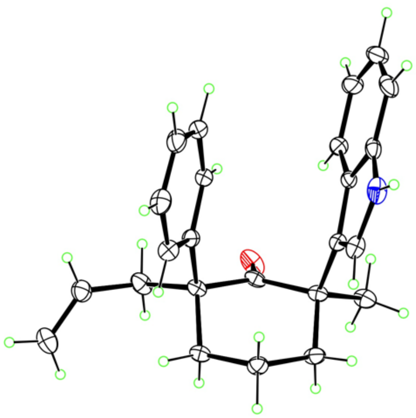

Product ***trans*-13**

Rf: 0.3 in 20:40 (Hexanes : CH_2Cl_2)

^{1}H NMR (400 MHz, $CDCl_3$) δ = 7.98 (s, 1H), 7.56 (d, *J* = 8.0 Hz, 1H), 7.42 – 7.32 (m, 5H), 7.30 – 7.26 (m, 1H), 7.19 (ddd, *J* = 8.2, 7.0, 1.2 Hz, 1H), 7.11 (ddd, *J* = 8.1, 7.1, 1.1 Hz, 1H), 6.95 (d, *J* = 2.2 Hz, 1H), 5.51– 5.40 (m, 1H), 4.96 – 4.89 (m, 2H), 2.65 – 2.46 (m, 4H), 2.10 – 2.00 (m, 2H), 1.87 – 1.77 (m, 1H), 1.76 – 1.68 (m, 1H), 1.26 (s, 3H).

^{13}C NMR: (125 MHz, $CDCl_3$) δ = 213.6, 140.8, 137.1, 134.5, 128.6, 126.7, 126.2, 125.5, 121.8, 121.1, 121.1, 120.9, 119.2, 117.8, 111.4, 55.1, 49.6, 47.0, 36.9, 30.3, 26.1, 17.7.

IR: *f* (cm^{-1}) = 3374, 2957, 2924, 2860, 1681, 1421, 1343, 1245, 1120, 1011, 917.

HRMS: (ESI-TOF) *m/z*: $(M+H)^+$ = 344.2009 calculated for $C_{24}H_{26}NO$; Found 344.2018.

(±)-(2*R*,6*R*)-2-allyl-6-(1*H*-indol-3-yl)-6-methyl-2-phenylcyclohexanone (*cis*-13)

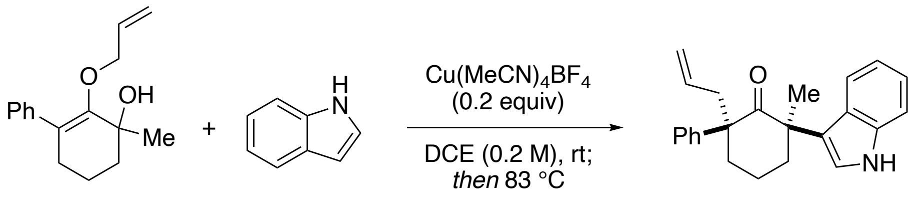

10 *cis*-**13**

Procedure: α-Hydroxyl enol ether **10** (100 mg, 0.409 mmol) was dissolved in DCE (2 mL, 0.2 M). Indole (72 mg, 0.614 mmol) was added, followed by addition of $Cu(MeCN)_4BF_4$ (26 mg, 0.081 mmol). The reaction mixture was stirred at room temperature for 24 hours, at which the starting material was fully consumed as monitored by TLC. The solution was then heated to 83 °C in a preheated oil bath for 16 hours. Upon complete Claisen rearrangement as monitored by TLC, the crude reaction mixture was concentrated under vacuum and purified by column chromatography using 100% hexanes to 90:10 hexanes : CH_2Cl_2 to afford compound ***cis*-13** in 94% yield (132 mg, 0.384 mmol) as a white solid. ^{1}H NMR analysis of the crude reaction mixture indicated >20:1 dr.

CHARACTERIZATION OF PRODUCTS IN SCHEME 3

(±)-(2*R*,6*R*)-2-allyl-6-(5-methoxy-1*H*-indol-3-yl)-6-methyl-2-phenylcyclohexanone (14a)

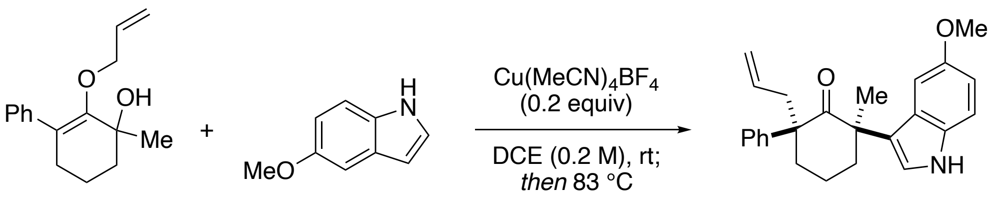

10 **14a**

Procedure: α-Hydroxyl enol ether **10** (150 mg, 0.614 mmol) was dissolved in DCE (3.1 mL, 0.2 M). 5-Methoxyindole (136 mg, 0.922 mmol) was added, followed by addition of $Cu(MeCN)_4BF_4$ (39 mg, 0.122 mmol). The reaction mixture was then stirred at room temperature for 11 hours, at which the starting material was fully consumed as monitored by TLC. The solution was subsequently heated to 83 °C in a preheated oil bath for 12 hours. Upon complete Claisen rearrangement as monitored by TLC, the crude reaction mixture was concentrated under vacuum and purified by column chromatography using 100% hexanes to 50:50 hexanes : CH_2Cl_2 to afford compound **14a** in 88% yield (201 mg, 0.538 mmol) as a white solid. ^{1}H NMR analysis of the crude reaction mixture indicated >20:1 dr.

Rf: 0.6 in 20:40 (Hexanes : CH_2Cl_2)

^{1}H NMR: (500 MHz, $CDCl_3$) δ = 7.44 (bs, 1H), 7.19 (d, *J* = 2.6 Hz, 1H), 6.96 (d, *J* = 8.8 Hz, 1H), 6.85 (d, *J* = 7.2 Hz, 2H), 6.76 (dd, *J* = 8.9, 2.5 Hz, 1H), 6.71 – 6.66 (m, 2H), 6.62 (t, *J* = 7.8 Hz, 2H), 5.36 – 5.26 (m, 1H), 4.90 – 4.86 (m , 2H), 3.91 (s, 3H), 2.67 – 2.55 (m, 3H), 2.52 – 2.43 (m, 2H), 1.97 – 1.88 (m, 1H), 1.92 – 1.82 (m, 2H), 1.53 (s, 3H).

^{13}C NMR: (125 MHz, $CDCl_3$) δ = 212.5, 153.6, 139.6, 134.7, 131.6, 126.7, 126.5, 125.6, 125.3, 121.5, 117.8, 117.4, 111.9, 111.2, 111.2, 103.1, 56.1, 55.1, 48.9, 46.7, 39.3, 33.6, 27.1, 18.8.

IR: *f* (cm^{-1}) = 3381, 2926, 2858, 1691, 1484, 1449, 1216.

HRMS: (ESI-TOF) *m/z*: $(M+H)^+$ = 374.2114 calculated for $C_{25}H_{28}NO_2$; Found 374.2105.

X-Ray Structure: Crystal growth was performed via slow evaporation using 5% CH_2Cl_2 in hexanes.

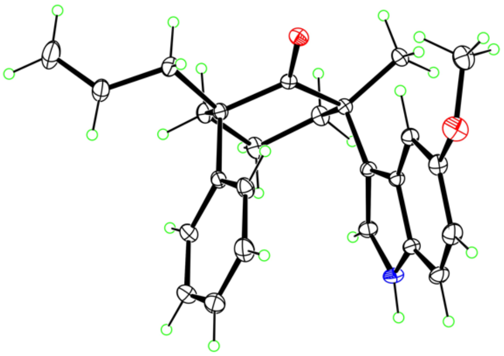

(±)-(2*R*,6*R*)-2-allyl-6-(5-(benzyloxy)-1*H*-indol-3-yl)-6-methyl-2-phenylcyclohexanone (14b)

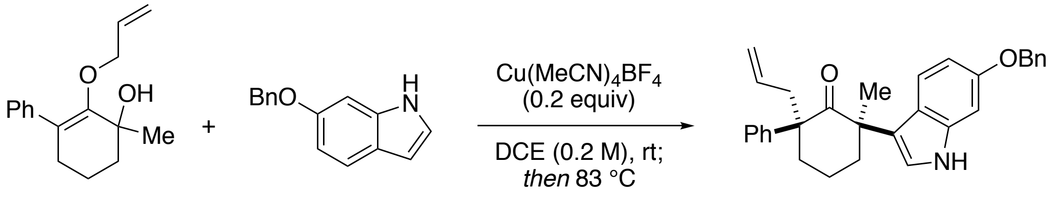

10 14b

Procedure: α-Hydroxyl enol ether **10** (150 mg, 0.614 mmol) was dissolved in DCE (3.1 mL, 0.2 M). 6-Benzyloxyindole (205 mg, 0.922 mmol) was added, followed by addition of $Cu(MeCN)_4BF_4$ (39 mg, 0.122 mmol). The reaction mixture was then stirred at room temperature for 41 hours, at which the starting material was fully consumed as monitored by TLC. The solution was then heated to 83 °C in a preheated oil bath for 16 hours. Upon complete Claisen rearrangement as monitored by TLC, the crude reaction mixture was concentrated under vacuum and purified by column chromatography using 100% hexanes to 50:50 hexanes : CH_2Cl_2 to afford compound **14b** in 75% yield (208 mg, 0.463 mmol) as a white solid. ^{1}H NMR analysis of the crude reaction mixture indicated >20:1 dr.

Rf: 0.7 in 50:50 (Hexanes : CH_2Cl_2)

^{1}H NMR: (500 MHz, $CDCl_3$) δ = 7.57 (d, J = 8.9 Hz, 1H), 7.47 (d, J = 7.2 Hz, 2H), 7.40 (t, J = 7.5 Hz, 2H), 7.38 (bs, 1H), 7.33 (t, J = 7.3 Hz, 1H), 6.84 (d, J = 8.3 Hz, 3H), 6.69 (t, J = 7.2 Hz, 1H), 6.64 – 6.59 (m, 3H), 6.55 (d, J = 2.4 Hz, 1H), 5.37 – 5.27 (m, 1H), 5.09 (s, 2H), 4.91 – 4.87 (m, 2H), 2.67 – 2.59 (m, 2H), 2.58 – 2.51 (m, 1H), 2.51 – 2.43 (m, 2H), 1.95 – 1.87 (m, 1H), 1.87 – 1.79 (m, 2H), 1.54 (s, 3H).

^{13}C NMR: (125 MHz, $CDCl_3$) δ = 212.8, 155.0, 139.6, 137.5, 137.1, 134.7, 128.5, 127.7, 127.4, 126.7, 125.7, 125.3, 121.8, 120.9, 119.8, 118.2, 117.3, 109.7, 95.8, 70.5, 55.1, 48.9, 46.7, 39.0, 33.4, 27.5, 18.7.

IR: f (cm^{-1}) = 3392, 2924, 1692, 1626, 1496, 1453.

HRMS: (ESI-TOF) *m/z*: $(M+H)^+$= 450.2427 calculated for $C_{31}H_{32}NO_2$; Found 450.2426.

(±)-(2*R*,6*R*)-2-allyl-6-(5-(4-methoxyphenyl)-1*H*-indol-3-yl)-6-methyl-2-phenylcyclohexanone (14c)

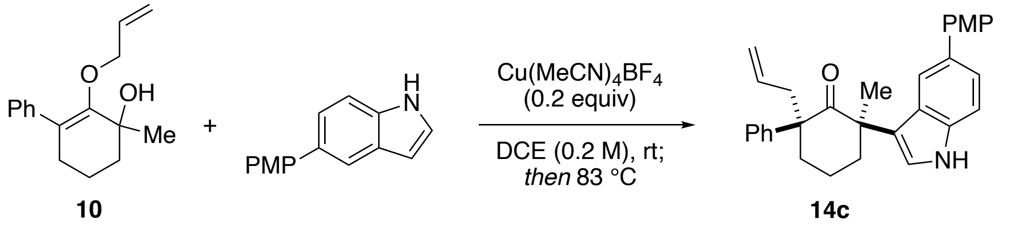

Procedure: α-Hydroxyl enol ether **10** (163 mg, 0.668 mmol) was dissolved in DCE (3.3 mL, 0.2 M). 5-(4-methoxyphenyl)-1*H*-indole (223 mg, 1.002 mmol) was added, followed by addition of $Cu(MeCN)_4BF_4$ (42 mg, 0.134 mmol). The reaction mixture was stirred at room temperature for 13 hours, at which the starting material was fully consumed as monitored by TLC. The solution was then heated to 83 °C in a preheated oil bath for 25 hours. Upon complete Claisen rearrangement as monitored by TLC, the crude reaction mixture was concentrated under vacuum and purified by column chromatography using 100% hexanes to 50:50 hexanes : CH_2Cl_2 to afford

compound **14c** in 73% yield (219 mg, 0.487 mmol) as a white solid. ^{1}H NMR analysis of the crude reaction mixture indicated >20:1 dr.

Rf: 0.7 in 50:50 (Hexanes : CH_2Cl_2)

^{1}H NMR: (500 MHz, $CDCl_3$) δ = 7.84 (s, 1H), 7.62 (d, *J* = 8.6 Hz, 2H), 7.48 (bs, 1H), 7.30 (d, *J* = 8.4 Hz, 1H), 7.10 (d, *J* = 8.4 Hz, 1H), 7.03 (d, *J* = 8.5 Hz, 2H), 6.86 (d, *J* = 7.5 Hz, 2H), 6.72 (s, 1H), 6.67 – 6.64 (m, 1H), 6.59 – 6.56 (t, *J* = 7.3 Hz, 2H), 5.32 – 5.24 (m, 1H), 4.88 – 4.85 (m, 2H), 3.89 (s, 3H), 2.68 – 2.56 (m, 3H), 2.55 – 2.43 (m, 2H), 1.98 – 1.89 (m, 1H), 1.89 – 1.80 (m, 2H), 1.56 (s, 3H).

^{13}C NMR: (125 MHz, $CDCl_3$) δ = 212.5, 158.4, 139.4, 135.6, 135.4, 134.7, 132.3, 128.4, 126.7, 126.6, 125.8, 125.4, 121.3, 121.2, 119.2, 118.5, 117.4, 114.2, 110.7, 100.0, 55.4, 55.1, 49.0, 46.8, 39.2, 33.6, 27.5, 18.8.

IR: *f* (cm^{-1}) = 3443, 2996, 2925, 2867, 1686, 1604, 1447, 1336, 1177, 1090, 1013.

HRMS: (ESI-TOF) *m/z*: $(M+H)^{+}$ = 450.2428 calculated for $C_{31}H_{32}NO_2$; Found 450.2445.

(±)-(2*R*,6*R*)-2-allyl-6-(5-hydroxy-1*H*-indol-3-yl)-6-methyl-2-phenylcyclohexanone (14d)

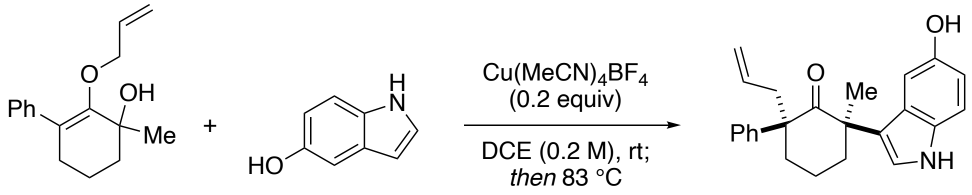

10 **14d**

Procedure: α-Hydroxyl enol ether **10** (147 mg, 0.602 mmol) was dissolved in DCE (3.0 mL, 0.2 M). 5-Hydroxyindole (120 mg, 0.902 mmol) was added, followed by addition of $Cu(MeCN)_4BF_4$ (38 mg, 0.120 mmol). The reaction mixture was stirred at room temperature for 48 hours, at which the starting material was fully consumed as monitored by TLC. The solution was then heated to 83 °C in a preheated oil bath for 17 hours. Upon complete Claisen rearrangement as monitored by

TLC, the crude reaction mixture was concentrated under vacuum and purified by column chromatography using 100% hexanes to 80:20 hexanes : EtOAc to afford compound **14d** in 42% yield (90 mg, 0.250 mmol) as a colorless solid. ^{1}H NMR analysis of the crude reaction mixture indicated >20:1 dr.

Rf: 0.5 in 80:20 (Hexanes : EtOAc)

^{1}H NMR: (500 MHz, $CDCl_3$) δ = 7.40 (bs, 1H), 7.03 (d, *J* = 2.4 Hz, 1H), 6.95 (d, *J* = 8.6 Hz, 1H), 6.90 – 6.85 (m, 2H), 6.76 – 6.65 (m, 5H), 5.35 – 5.26 (m, 1H), 4.91 – 4.84 (m, 2H), 2.66 – 2.59 (m, 2H), 2.58 – 2.52 (m, 1H), 2.49 – 2.42 (m, 2H), 1.93 – 1.79 (m, 3H), 1.51 (s, 3H).

^{13}C NMR: (125 MHz, $CDCl_3$) δ = 212.8, 149.1, 139.7, 134.7, 131.7, 126.8, 126.6, 125.8, 125.4, 121.9, 117.7, 117.4, 111.3, 111.2, 105.6, 55.1, 48.8, 46.7, 38.9, 33.3, 26.9, 18.7.

IR: *f* (cm^{-1}) = 3403, 3071, 2962, 2866, 1682, 1582, 1466, 1372, 1208, 911, 858, 797.

HRMS: (ESI-TOF) *m/z*: $(M+H)^+$ = 360.1959 calculated for $C_{24}H_{26}NO_2$; Found 360.1975.

(±)-(2*R*,6*R*)-2-allyl-6-(5-iodo-1*H*-indol-3-yl)-6-methyl-2-phenylcyclohexanone (14e)

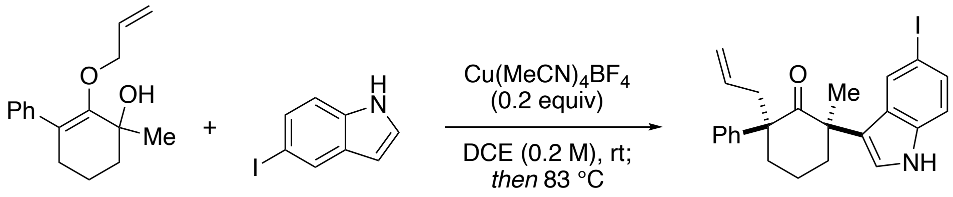

10 **14e**

Procedure: α-Hydroxyl enol ether **10** (192 mg, 0.786 mmol) was dissolved in DCE (4.0 mL, 0.2 M). 5-Iodoindole (286 mg, 1.179 mmol) was added, followed by addition of $Cu(MeCN)_4BF_4$ (54 mg, 0.157 mmol). The reaction mixture was stirred at room temperature for 18 hours, at which the starting material was fully consumed as monitored by TLC. The solution was then heated to 83 °C in a preheated oil bath for 22 hours. Upon complete Claisen rearrangement as monitored by TLC, the crude reaction mixture was concentrated under vacuum and purified by column

chromatography using 100% hexanes to 50:50 hexanes : CH_2Cl_2 to afford compound **14e** in 57% yield (211 mg, 0.450 mmol) as a colorless solid. ^{1}H NMR analysis of the crude reaction mixture indicated >20:1 dr.

Rf: 0.4 in 50:50 (Hexanes : CH_2Cl_2)

^{1}H NMR: (500 MHz, $CDCl_3$) δ = 7.96 (s, 1H), 7.55 (bs, 1H), 7.33 (dd, *J* = 8.4, 1.7 Hz, 1H), 6.85 (t, *J* = 8.0 Hz, 3H), 6.71 – 6.63 (m, 4H), 5.33 – 5.24 (m, 1H), 4.89 – 4.86 (s, 2H), 2.69 – 2.59 (m, 2H), 2.57 – 2.50 (m, 1H), 2.50 – 2.41 (m, 2H), 1.95 – 1.80 (m, 3H), 1.49 (s, 3H).

^{13}C NMR: (125 MHz, $CDCl_3$) δ = 212.3, 139.2, 135.4, 134.6, 130.1, 129.8, 128.7, 126.8, 125.8, 125.5, 121.5, 117.8, 117.5, 112.5, 82.8, 55.1, 48.8, 46.7, 38.8, 33.4, 27.4, 18.6.

IR: *ƒ* (cm^{-1}) = 3366, 2924, 2855, 1685, 1494, 1110.

HRMS: (ESI-TOF) *m/z*: $(M+H)^+$ = 470.0975 calculated for $C_{24}H_{25}INO$; Found 470.0976.

X-Ray Structure: Crystal growth was performed via slow evaporation using 5% CH_2Cl_2 in hexanes.

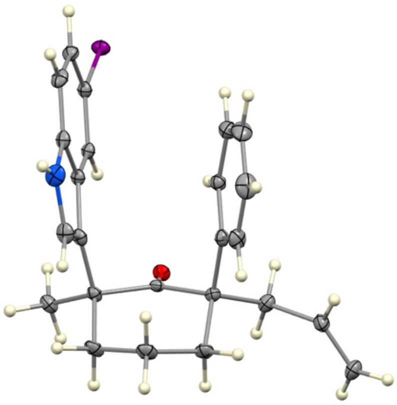

(±)-(2*R*,6*R*)-2-allyl-6-(7-bromo-1*H*-indol-3-yl)-6-methyl-2-phenylcyclohexanone (14f)

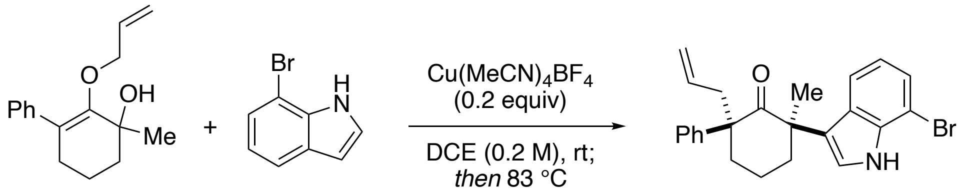

Procedure: α-Hydroxyl enol ether **10** (150 mg, 0.614 mmol) was dissolved in DCE (3.1 mL, 0.2 M). 7-Bromoindole (180 mg, 0.922 mmol) was added, followed by addition of $Cu(MeCN)_4BF_4$ (39 mg, 0.122 mmol). The reaction mixture was stirred at room temperature for 23 hours, at which the starting material was fully consumed as monitored by TLC. The solution was then heated to 83 °C in a preheated oil bath for 11 hours. Upon complete Claisen rearrangement as monitored by TLC, the crude reaction mixture was concentrated under vacuum and purified by column chromatography using 100% hexanes to 50:50 hexanes : CH_2Cl_2 to afford compound **14f** in 48% yield (124 mg, 0.293 mmol) as a white solid. ^{1}H NMR analysis of the crude reaction mixtures indicated >20:1 dr.

Rf: 0.8 in 50:50 (Hexanes : CH_2Cl_2)

^{1}H NMR: (500 MHz, $CDCl_3$) δ = δ 7.67 (bs, 1H), 7.63 (d, *J* = 8.0 Hz, 1H), 7.27 – 7.23 (m, 1H), 6.93 (t, *J* = 7.8 Hz, 1H), 6.84 – 6.77 (m, 2H), 6.77 – 6.68 (m, 2H), 6.60 (t, *J* = 7.7 Hz, 2H), 5.34 – 5.24 (m, 1H), 4.90 – 4.86 (m, 2H), 2.67 – 2.59 (m, 2H), 2.59 – 2.53 (m, 1H), 2.47 – 2.43 (m, 2H), 1.96 – 1.89 (m, 1H), 1.89 – 1.79 (m, 2H), 1.53 (s, 3H).

^{13}C NMR: (125 MHz, $CDCl_3$) δ = 212.4, 139.4, 134.9, 134.6, 127.4, 126.7, 125.7, 125.5, 123.9, 121.4, 120.6, 120.3, 119.7, 117.5, 104.2, 55.2, 49.1, 46.6, 39.1, 33.5, 27.3, 18.7.

IR: *f* (cm^{-1}) = 3412, 2925, 1689, 1433, 1374, 1208, 741, 701.

HRMS: (ESI-TOF) *m/z*: $(M+H)^+$ = 422.1114 calculated for $C_{24}H_{25}BrNO$; Found 422.1113.

X-Ray Structure: Crystal growth was performed via slow evaporation using 5% CH_2Cl_2 in hexanes.

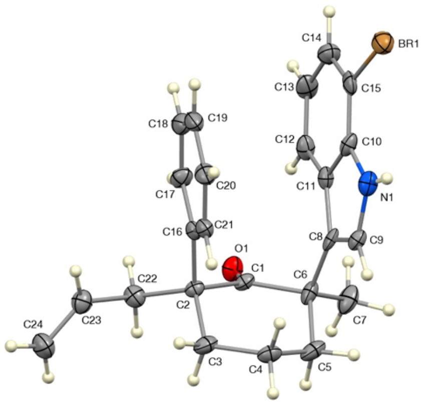

(±)-(2*R*,6*R*)-2-allyl-6-(5,6-dichloro-1*H*-indol-3-yl)-6-methyl-2-phenylcyclohexanone (14g)

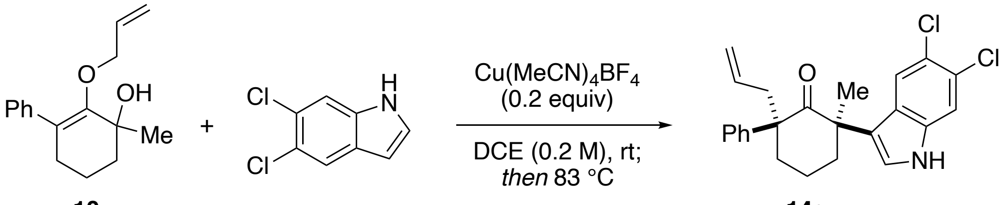

Procedure: α-Hydroxyl enol ether **10** (188 mg, 0.769 mmol) was dissolved in DCE (3.8 mL, 0.2 M). 5,6-Dichloro-1*H*-indole (214 mg, 1.154 mmol) was added, followed by addition of $Cu(MeCN)_4BF_4$ (48 mg, 0.154 mmol). The reaction mixture was stirred at room temperature for 23 hours, at which the starting material was fully consumed as monitored by TLC. The solution was then heated to 83 °C in a preheated oil bath for 57 hours. Upon complete Claisen rearrangement as monitored by TLC, the crude reaction mixture was concentrated under vacuum and purified by column chromatography using 100% hexanes to 50:50 hexanes : CH_2Cl_2 to afford compound **14g** in 63% yield (199 mg, 0.483 mmol) as a white solid. ^{1}H NMR analysis of the crude reaction mixture indicated >20:1 dr.

Rf: 0.7 in 50:50 (Hexanes : CH_2Cl_2)

¹H NMR: (500 MHz, $CDCl_3$) δ = 7.73 (s, 1H), 7.54 (s, 1H), 7.18 (s, 1H), 6.88 – 6.83 (m, 2H), 6.75 – 6.71 (m, 2H), 6.71 – 6.65 (m, 2H), 5.34 – 5.23 (m, 1H), 4.92 – 4.86 (m, 2H), 2.67 – 2.60 (m, 2H), 2.55 – 2.39 (m, 3H), 1.94 – 1.81 (m, 3H), 1.49 (s, 3H).

¹³C NMR: (125 MHz, $CDCl_3$) δ = 212.3, 139.3, 135.1, 134.5, 127.0, 125.9, 125.8, 125.7, 125.5, 123.2, 122.7, 122.2, 118.4, 117.6, 111.9, 55.1, 48.7, 46.6, 38.7, 33.2, 27.3, 18.6.

IR: *f* (cm^{-1}) = 3374, 2978, 2926, 2824, 1734, 1695, 1545, 1448, 1329, 1141, 994, 925, 859, 735.

HRMS: (ESI-TOF) *m/z*: $(M+H)^+$ = 412.1230 calculated for $C_{24}H_{24}Cl_2NO$; Found 412.1235.

(±)-Methyl 3-((1*R*,3*R*)-3-allyl-1-methyl-2-oxo-3-phenylcyclohexyl)-1*H*-indole-5-carboxylate (14h)

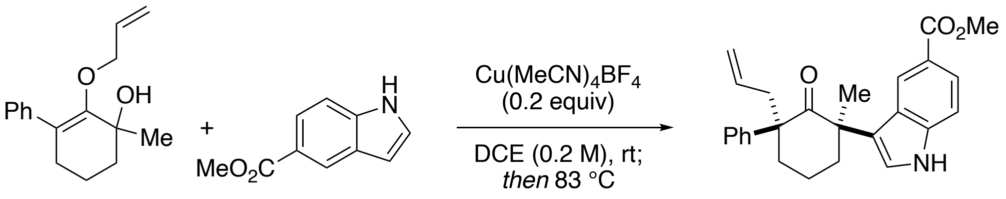

10 **14h**

Procedure: α-Hydroxyl enol ether **10** (150 mg, 0.614 mmol) was dissolved in DCE (3.0 mL, 0.2 M). Methyl 1*H*-indole-5-carboxylate (161 mg, 0.921 mmol) was added, followed by addition of $Cu(MeCN)_4BF_4$ (38 mg, 0.123 mmol). The reaction mixture was stirred at room temperature for 22 hours, at which the starting material was fully consumed as monitored by TLC. The solution was then heated to 83 °C in a preheated oil bath for 46 hours. Upon complete Claisen rearrangement as monitored by TLC, the crude reaction mixture was concentrated under vacuum and purified by column chromatography using 100% hexanes to 80:20 hexanes : EtOAc to afford compound **14h** in 58% yield (142 mg, 0.354 mmol) as a white solid. ¹H NMR analysis of the crude reaction mixture indicated >20:1 dr.

Rf: 0.6 in 70:30 (Hexanes : EtOAc)

^{1}**H NMR** (400 MHz, $CDCl_3$) δ = 8.46 (s, 1H), 7.85 – 7.77 (m, 2H), 7.09 (dd, *J* = 8.6, 0.7 Hz, 1H), 6.85 – 6.79 (m, 2H), 6.75 (d, *J* = 2.5 Hz, 1H), 6.70 – 6.64 (m, 1H), 6.58 (dd, *J* = 8.2, 6.8 Hz, 2H), 5.35 – 5.23 (m, 1H), 4.91 – 4.83 (m, 2H), 3.98 (s, 3H), 2.68 – 2.53 (m, 3H), 2.52 – 2.41 (m, 2H), 2.00 – 1.90 (m, 1H), 1.90 – 1.79 (m, 2H), 1.57 (s, 3H).

^{13}**C NMR:** (125 MHz, $CDCl_3$) δ = 212.4, 168.5, 139.4, 139.0, 134.6, 126.8, 125.8, 125.7, 125.4, 124.0, 123.1, 122.2, 121.2, 119.7, 117.5, 110.4, 55.2, 51.9, 48.9, 46.7, 39.0, 33.4, 27.7, 18.6.

IR: *f* (cm^{-1}) = 3382, 2917, 2866, 2848, 1698,1683, 1434, 1242, 1179, 1100, 953.

HRMS: (ESI-TOF) *m/z*: $(M+H)^+$ = 402.2064 calculated for $C_{26}H_{28}NO_3$; Found 402.2055.

(±)-(2*R*,6*R*)-2-allyl-6-methyl-6-(1-methyl-1*H*-indol-3-yl)-2-phenylcyclohexanone (14i)

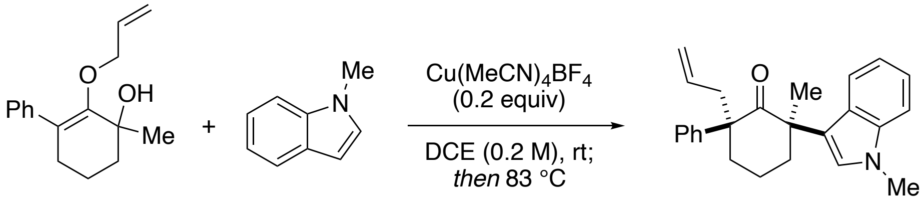

Procedure: α-Hydroxyl enol ether **10** (168 mg, 0.688 mmol) was dissolved in DCE (3.2 mL, 0.2 M). *N*-Methylindole (0.13 mL, 1.031 mmol) was added, followed by addition of $Cu(MeCN)_4BF_4$ (43 mg, 0.138 mmol). The reaction mixture was stirred at room temperature for 16 hours, at which the starting material was fully consumed as monitored by TLC. The solution was then heated to 83 °C in a preheated oil bath for 48 hours. Upon complete Claisen rearrangement as monitored by TLC, the crude reaction mixture was concentrated under vacuum and purified by column chromatography using 100% hexanes to 50:50 hexanes : CH_2Cl_2 to afford compound **14i** in 73% yield (178 mg, 0.498 mmol) as an orange solid. ^{1}H NMR analysis of the crude reaction mixture indicated >20:1 dr.

Rf: 0.5 in 50:50 (Hexanes : CH_2Cl_2)

^{1}H NMR: (400 MHz, $CDCl_3$) δ = 7.68 (d, *J* = 8.0 Hz, 1H), 7.12 (t, *J* = 7.0 Hz, 1H), 7.04 (t, *J* = 7.0 Hz, 1H), 6.99 (d, *J* = 8.1 Hz, 1H), 6.78 – 6.74 (m, 2H), 6.72 (t, *J* = 7.3 Hz, 1H), 6.56 (t, *J* = 8.0 Hz, 2H), 6.42 (s, 1H), 5.31 (dddd, *J* = 15.6, 9.2, 8.0, 6.6 Hz, 1H), 4.92 – 4.89 (m, 1H), 4.88 – 4.85 (m, 1H), 3.35 (s, 3H), 2.67 – 2.55 (m, 3H), 2.53 – 2.42 (m, 2H), 1.97 – 1.88 (m, 1H), 1.88 – 1.80 (m, 2H), 1.57 (s, 3H).

^{13}C NMR: (100 MHz, $CDCl_3$) δ = 212.2, 137.1, 134.8, 126.7, 126.5, 125.8, 125.6, 124.8, 121.2, 121.1, 118.7, 117.3, 116.3, 108.6, 55.0, 49.0, 46.6, 39.3, 33.6, 32.2, 27.4, 18.9.

IR: *ƒ* (cm^{-1}) = 3055, 2961, 2924, 1687, 1637, 1536, 1460, 1372, 1247, 1151, 1086, 1018, 1987.

HRMS: (ESI-TOF) *m/z*: $(M+H)^+$ = 358.2170 calculated for $C_{25}H_{28}NO$; Found 358.2174.

X-Ray Structure: Crystal growth was performed via slow evaporation using 5% CH_2Cl_2 in hexanes.

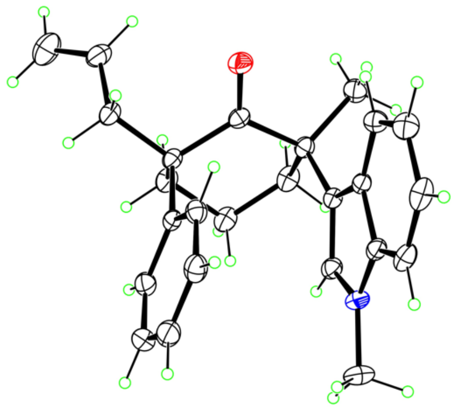

(±)-(2*R*,6*R*)-2-allyl-6-(1*H*-indol-3-yl)-2-(4-methoxyphenyl)-6-methylcyclohexanone (16a)

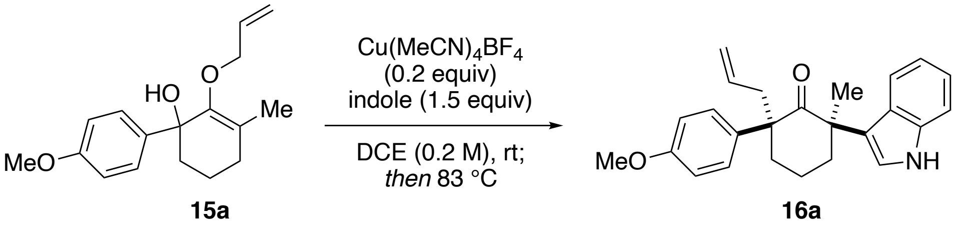

Procedure: α-Hydroxyl enol ether **15a** (157 mg, 0.572 mmol) was dissolved in DCE (2.8 mL, 0.2 M). Indole (100 mg, 0.858 mmol) was added, followed by addition of $Cu(MeCN)_4BF_4$ (36 mg, 0.114 mmol). The reaction mixture was stirred at room temperature for 5 hours, at which the starting material was fully consumed as monitored by TLC. The solution was then heated to 83 °C in a preheated oil bath for 18 hours. Upon complete Claisen rearrangement as monitored by TLC, the crude reaction mixture was concentrated under vacuum and purified by column chromatography using 100% hexanes to 30:70 hexanes : CH_2Cl_2 to afford compound **16a** in 89% yield (189 mg, 0.506 mmol) as an orange solid. ^{1}H NMR analysis of the crude reaction mixture indicated >20:1 dr.

Rf: 0.8 in 50:50 (Hexanes : CH_2Cl_2)

^{1}H NMR: (500 MHz, $CDCl_3$) δ = 7.67 (d, J = 7.9 Hz, 1H), 7.50 (bs, 1H), 7.09 – 7.02 (m, 3H), 6.74 – 6.69 (m, 3H), 6.10 (d, J = 8.9 Hz, 2H), 5.35 – 5.26 (m, 1H), 4.90 – 4.87 (m, 2H), 3.50 (s, 3H), 2.63 – 2.56 (m, 3H), 2.50 – 2.41 (m, 2H), 1.93 – 1.87 (m, 1H), 1.85 – 1.78 (m, 2H), 1.53 (s, 3H).

^{13}C NMR: (125 MHz, $CDCl_3$) δ = 213.0, 157.0, 136.5, 134.9, 131.7, 127.0, 126.3, 121.6, 121.3, 120.7, 119.1, 118.6, 117.3, 112.1, 110.6, 55.0, 54.4, 48.9, 46.6, 39.2, 33.8, 27.4, 18.8.

IR: *ƒ* (cm^{-1}) = 3402, 3064, 2929, 1964, 1690, 1608, 1511, 1417, 1248, 1183.

HRMS: (ESI-TOF) *m/z*: $(M+H)^+$ = 374.2115 calculated for $C_{25}H_{28}NO_2$; Found 374.2114.

X-Ray Structure: Crystal growth was performed via slow evaporation using 5% CH_2Cl_2 in hexanes.

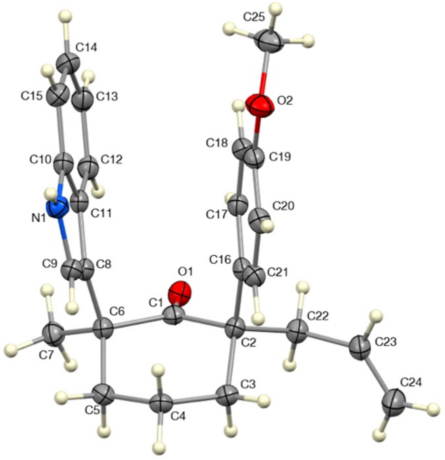

Procedure for One-Gram Synthesis: α-Hydroxyl enol ether **15a** (1.02 g, 3.72 mmol) was dissolved in DCE (18.6 mL, 0.2 M). Indole (653 mg, 5.58 mmol) was added, followed by addition of $Cu(MeCN)_4BF_4$ (234 mg, 0.744 mmol). The reaction mixture was stirred at room temperature for 5 hours, at which the starting material was fully consumed as monitored by TLC. The solution was then heated to 83 °C in a preheated oil bath for 14 hours. Upon complete Claisen rearrangement as monitored by TLC, the crude reaction mixture was concentrated under vacuum and purified by column chromatography using 100% hexanes to 30:70 hexanes : CH_2Cl_2 to afford compound **16a** in 72% yield (998 mg, 2.67 mmol) as an orange solid. ^1H NMR analysis of the crude reaction mixture indicated >20:1 dr.

(±)-(2*R*,6*R*)-2-allyl-6-(1*H*-indol-3-yl)-2-(3-methoxyphenyl)-6-methylcyclohexanone (16b)

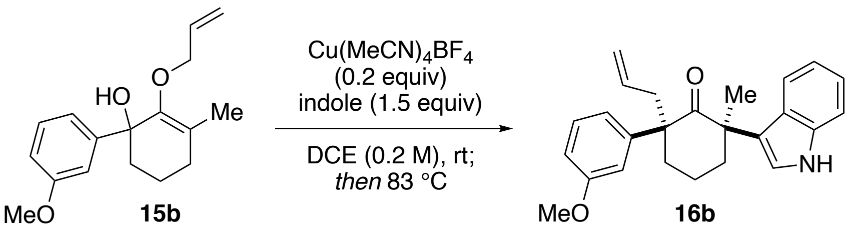

Procedure: α-Hydroxyl enol ether **15b** (168 mg, 0.612 mmol) was dissolved in DCE (3.0 mL, 0.2 M). Indole (107 mg, 0.918 mmol) was added, followed by addition of $Cu(MeCN)_4BF_4$ (38 mg, 0.122 mmol). The reaction mixture was stirred at room temperature for 25 hours, at which the starting material was fully consumed as monitored by TLC. The solution was then heated to 83 °C in a preheated oil bath for 24 hours. Upon complete Claisen rearrangement as monitored by TLC, the crude reaction mixture was concentrated under vacuum and purified by column chromatography using 100% hexanes to 30:70 hexanes : CH_2Cl_2 to afford compound **16b** in 88% yield (189 mg, 0.610 mmol) as a white solid. ^{1}H NMR analysis of the crude reaction mixture indicated >20:1 dr.

Rf: 0.8 in 50:50 (Hexanes : CH_2Cl_2)

^{1}H NMR: (500 MHz, $CDCl_3$) δ = 7.75 (dd, *J* = 7.4, 1.6 Hz, 1H), 7.57 (s, 1H), 7.13 – 7.02 (m, 3H), 6.71 (d, *J* = 2.5 Hz, 1H), 6.64 – 6.60 (m, 1H), 6.52 (dt, *J* = 7.8, 1.3 Hz, 1H), 6.32 – 6.26 (m, 2H), 5.36 – 5.28 (m, 1H), 4.93 – 4.87 (m, 2H), 3.27 (s, 3H), 2.65 – 2.56 (m, 3H), 2.54 – 2.43 (m, 2H), 1.96 – 1.89 (m, 1H), 1.88 – 1.79 (m, 2H), 1.57 (s, 3H).

^{13}C NMR: (125 MHz, $CDCl_3$) δ = 212.5, 158.5, 141.6, 136.4, 134.7, 127.7, 126.1, 121.5, 121.3, 121.0, 119.3, 118.3, 118.0, 117.4, 111.5, 111.4, 110.7, 55.2, 54.6, 49.0, 46.7, 39.2, 33.5, 27.4, 18.8.

IR: *f* (cm^{-1}) = 3405, 3070, 2961, 2927, 2865, 1691, 1598, 1489, 1338, 1244, 1105.

HRMS: (ESI-TOF) *m/z*: $(M+H)^+$ = 374.2115 calculated for $C_{25}H_{28}NO_2$; Found 374.2116.

(±)-(2*R*,6*R*)-2-allyl-6-(1*H*-indol-3-yl)-2-(2-methoxyphenyl)-6-methylcyclohexanone (16c)

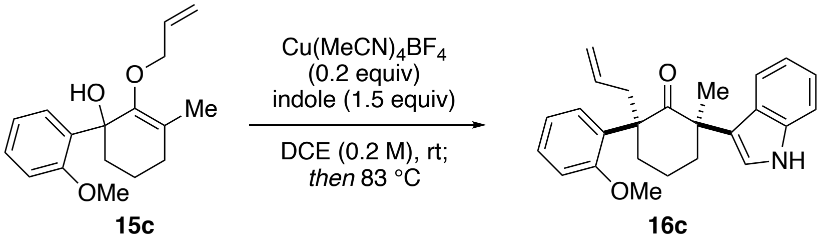

Procedure: α-Hydroxyl enol ether **15c** (148 mg, 0.539 mmol) was dissolved in DCE (2.7 mL, 0.2 M). Indole (94 mg, 0.809 mmol) was added, followed by addition of $Cu(MeCN)_4BF_4$ (34 mg, 0.108 mmol). The reaction mixture was stirred at room temperature for 6 hours, at which the starting material was fully consumed as monitored by TLC. The solution was then heated to 83 °C in a preheated oil bath for 18 hours. Upon complete Claisen rearrangement as monitored by TLC, the crude reaction mixture was concentrated under vacuum and purified by column chromatography using 100% hexanes to 50:50 hexanes : CH_2Cl_2 to afford compound **16c** in 40% yield (81 mg, 0.217 mmol) as a white solid. The crude diastereomeric ratio could not be determined with confidence due to the presence of complex mixture.

Rf: 0.8 in 50:50 (Hexanes : CH_2Cl_2)

^{1}H NMR (500 MHz, $CDCl_3$) δ = δ 7.94 – 7.90 (m, 1H), 7.57 (s, 1H), 7.21 (dd, *J* = 7.8, 1.7 Hz, 1H), 7.19 – 7.16 (m, 1H), 7.10 – 7.03 (m, 2H), 6.90 (ddd, *J* = 8.1, 7.3, 1.6 Hz, 1H), 6.73 (td, *J* = 7.5, 1.2 Hz, 1H), 6.63 (d, *J* = 2.3 Hz, 1H), 6.05 (d, *J* = 8.1 Hz, 1H), 5.37 – 5.29 (m, 1H), 4.88 – 4.81 (m, 2H), 2.98 – 2.93 (m, 1H), 2.75 (s, 3H), 2.74 – 2.68 (m, 1H), 2.55 – 2.47 (m, 1H), 2.43 (dd, *J* = 14.3, 8.4 Hz, 1H), 2.35 – 2.27 (m, 1H), 1.92 – 1.84 (m, 2H), 1.70 – 1.63 (m, 1H), 1.62 (s, 3H).

^{13}C NMR: (125 MHz, $CDCl_3$) δ = 212.8, 157.0, 136.4, 135.5, 129.6, 127.2, 126.8, 126.5, 122.7, 121.2, 120.4, 119.9, 119.1, 118.8, 116.6, 110.4, 109.7, 54.2, 53.6, 49.4, 41.1, 38.4, 34.6, 27.2, 18.0.

IR: *f* (cm^{-1}) = 3363, 3064, 2966, 2869, 2833, 1696, 1582, 1490, 1459, 1338, 1244, 1108, 1027, 910.

HRMS: (ESI-TOF) *m/z*: $(M+H)^+$ = 374.2115 calculated for $C_{25}H_{28}NO_2$; Found 374.2111.

(±)-(2*R*,6*R*)-2-allyl-2-(2,3-dihydrobenzo[*b*][1,4]dioxin-6-yl)-6-(1*H*-indol-3-yl)-6-methylcyclohexanone (16d)

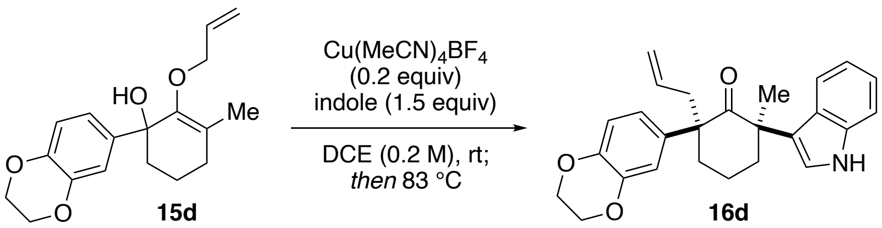

Procedure: α-Hydroxyl enol ether **15d** (151 mg, 0.499 mmol) was dissolved in DCE (2.5 mL, 0.2 M). Indole (88 mg, 0.749 mmol) was added, followed by addition of $Cu(MeCN)_4BF_4$ (31 mg, 0.100 mmol). The reaction mixture was stirred at room temperature for 15 hours, at which the starting material was fully consumed as monitored by TLC. The solution was then heated to 83 °C in a preheated oil bath for 24 hours. Upon complete Claisen rearrangement as monitored by TLC, the crude reaction mixture was concentrated under vacuum and purified by column chromatography using 100% hexanes to 60:40 hexanes : CH_2Cl_2 to afford compound **16d** in 80% yield (160 mg, 0.399 mmol) as a colorless solid. ^{1}H NMR analysis of the crude reaction mixture indicated >20:1 dr.

Rf: 0.8 in 80:20 (Hexanes : EtOAc)

^{1}H NMR: (500 MHz, $CDCl_3$) δ = 7.67 – 7.62 (m, 1H), 7.61 (s, 1H), 7.15 – 7.11 (m, 1H), 7.10 – 7.02 (m, 2H), 6.76 (d, *J* = 2.2 Hz, 1H), 6.40 – 6.33 (m, 2H), 6.15 (d, *J* = 8.4 Hz, 1H), 5.38 – 5.29 (m, 1H), 4.94 – 4.87 (m, 2H), 4.03 – 3.93 (m, 3H), 3.84 – 3.78 (m, 1H), 2.66 – 2.62 (m, 1H), 2.61

– 2.48 (m, 3H), 2.42 (dd, J = 14.0, 8.1 Hz, 1H), 1.94 – 1.85 (m, 1H), 1.85 – 1.76 (m, 2H), 1.52 (s, 3H).

^{13}C NMR: (125 MHz, $CDCl_3$) δ = 212.6, 142.0, 141.0, 136.3, 134.9, 133.0, 126.2, 121.5, 121.2, 120.6, 119.3, 118.8, 118.6, 117.2, 115.5, 115.2, 110.3, 64.0, 54.3, 48.9, 46.6, 39.0, 33.8, 27.4, 18.8.

IR: *f* (cm^{-1}) = 3401, 3067, 2967, 2927, 1688, 1638, 1587, 1458, 1372, 1285, 1207, 1051.

HRMS: (ESI-TOF) *m/z*: $(M+H)^+$ = 402.2064 calculated for $C_{26}H_{28}NO_3$; Found 402.2067.

(±)-(2*R*,6*R*)-2-allyl-6-(1*H*-indol-3-yl)-6-methyl-2-(4-(methylthio)phenyl)cyclohexanone (16e)

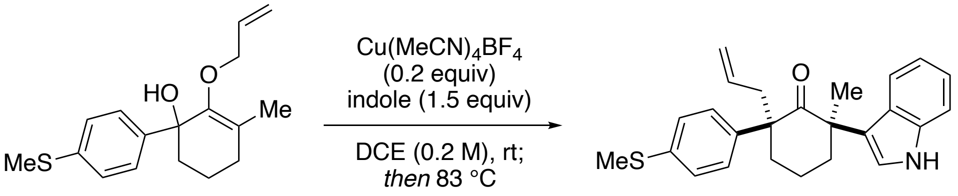

Procedure: α-Hydroxyl enol ether **15e** (191 mg, 0.658 mmol) was dissolved in DCE (3.2 mL, 0.2 M). Indole (115 mg, 0.986 mmol) was added, followed by addition of $Cu(MeCN)_4BF_4$ (41 mg, 0.132 mmol). The reaction mixture was stirred at room temperature for 41 hours, at which the starting material was fully consumed as monitored by TLC. The solution was then heated to 83 °C in a preheated oil bath for 48 hours. Upon complete Claisen rearrangement as monitored by TLC, the crude reaction mixture was concentrated under vacuum and purified by column chromatography using 100% hexanes to 40:60 hexanes: CH_2Cl_2 to afford compound **16e** in 76% yield (170 mg, 0.496 mmol) as a yellow solid. ^{1}H NMR analysis of the crude reaction mixture indicated >20:1 dr.

Rf: 0.7 in 50:50 (Hexanes : CH_2Cl_2)

^{1}H NMR: (400 MHz, $CDCl_3$) δ = 7.66 (d, J = 7.8 Hz, 1H), 7.52 (bs, 1H), 7.09 – 7.06 (m, 2H), 7.06 – 7.03 (m, 1H), 6.71 – 6.69 (m, 3H), 6.43 (d, J = 8.5 Hz, 2H), 5.35 – 5.23 (m, 1H), 4.89 –

4.83 (m, 2H), 2.61 – 2.55 (m, 3H), 2.51 – 2.38 (m, 2H), 2.22 (s, 3H), 1.90 – 1.79 (m, 3H), 1.53 (s, 3H).

^{13}C NMR: (125 MHz, $CDCl_3$) δ = 212.4, 136.5, 136.4, 134.8, 134.6, 126.3, 125.0, 121.6, 121.2, 120.7, 119.2, 118.2, 117.5, 110.6, 54.7, 49.0, 46.4, 39.4, 33.8, 27.4, 18.8, 15.7.

IR: *f* (cm^{-1}) = 3367, 3067, 2959, 2919, 1679, 1624, 1461, 1403, 1344, 1244, 1147, 1097, 1013, 993.

HRMS: (ESI-TOF) *m/z*: $(M+H)^+$ = 390.1887 calculated for $C_{25}H_{28}SNO$; Found 390.1890.

(±)-(2*R*,6*R*)-2-allyl-2-(4-chlorophenyl)-6-(1*H*-indol-3-yl)-6-methylcyclohexanone (16g)

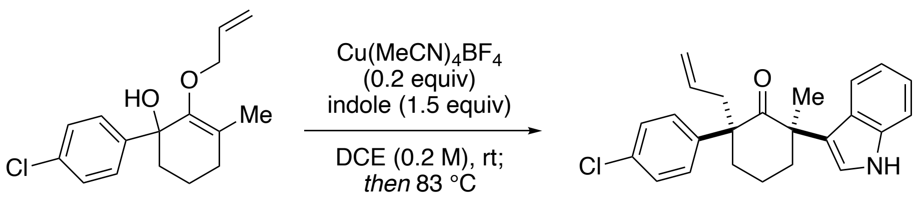

15g **16g**

Procedure: α-Hydroxyl enol ether **15g** (157 mg, 0.563 mmol) was dissolved in DCE (2.8 mL, 0.2 M). Indole (99 mg, 0.845 mmol) was added, followed by addition of $Cu(MeCN)_4BF_4$ (35 mg, 0.113 mmol). The reaction mixture was stirred at room temperature for 33 hours, at which the starting material was fully consumed as monitored by TLC. The solution was then heated to 83 °C in a preheated oil bath for 39 hours. Upon complete Claisen rearrangement as monitored by TLC, the crude reaction mixture was concentrated under vacuum and purified by column chromatography using 100% hexanes to 50:50 hexanes: CH_2Cl_2 to afford compound **16g** in 74% yield (157 mg, 0.415 mmol) as a red/pink solid. ^{1}H NMR analysis of the crude reaction mixture indicated >20:1 dr.

Rf: 0.5 in 50:50 (Hexanes : CH_2Cl_2)

^{1}H NMR: (500 MHz, $CDCl_3$) δ = 7.65 (d, *J* = 8.1 Hz, 1H), 7.56 (bs, 1H), 7.12 (d, *J* = 4.3 Hz, 2H), 7.09 – 7.02 (m, 1H), 6.72 (d, *J* = 8.7 Hz, 2H), 6.70 (d, *J* = 2.6 Hz, 1H), 6.50 (d, *J* = 8.6 Hz, 2H), 5.33 – 5.23 (m, 1H), 4.91 – 4.83 (m, 2H), 2.63 – 2.55 (m, 3H), 2.49 – 2.40 (m, 2H), 1.96 – 1.89 (m, 1H), 1.87 – 1.78 (m, 2H), 1.55 (s, 3H).

^{13}C NMR: (125 MHz, $CDCl_3$) δ = 212.0, 138.2, 136.5, 134.2, 131.1, 127.1, 126.6, 126.1, 121.9, 121.1, 120.7, 119.3, 118.0, 117.8, 110.8, 54.8, 49.1, 46.4, 39.3, 33.9, 27.3, 18.8.

IR: *f* (cm^{-1}) = 3404, 2927, 2860, 1692, 1491, 1460, 1246, 1095, 1013, 916, 824, 742.

HRMS: (ESI-TOF) *m/z*: $(M+H)^+$ = 378.1620 calculated for $C_{24}H_{25}ClNO$; Found 378.1628.

X-Ray Structure: Crystal growth was performed via slow evaporation using 5% CH_2Cl_2 in hexanes.

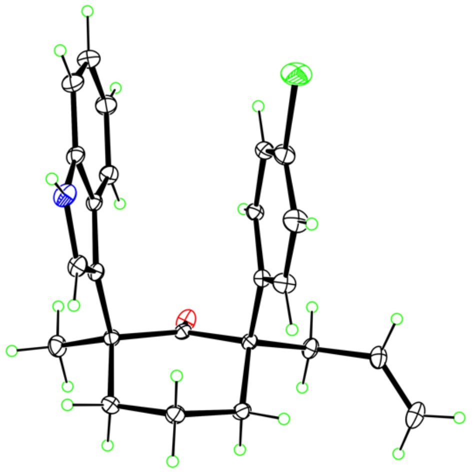

(±)-(2*R*,6*R*)-2-allyl-2-(3-fluoro-4-methoxyphenyl)-6-(1*H*-indol-3-yl)-6-methylcyclohexanone (16h)

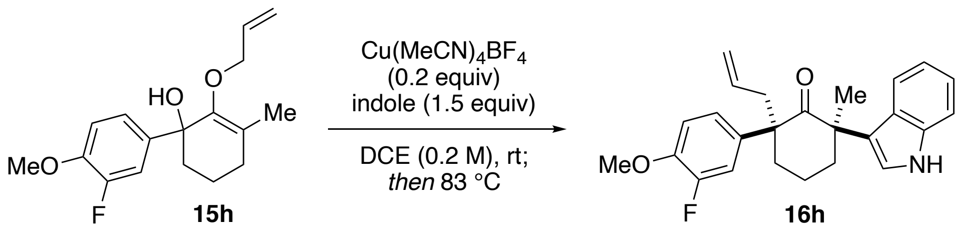

Procedure: α-Hydroxyl enol ether **15h** (151 mg, 0.517 mmol) was dissolved in DCE (2.6 mL, 0.2 M). Indole (90 mg, 0.734 mmol) was added, followed by addition of $Cu(MeCN)_4BF_4$ (32 mg, 0.103 mmol). The reaction mixture was stirred at room temperature for 40 hours, at which the starting material was fully consumed as monitored by TLC. The solution was then heated to 83 °C in a preheated oil bath for 24 hours. Upon complete Claisen rearrangement as monitored by TLC, the crude reaction mixture was concentrated under vacuum and purified by column chromatography using 100% hexanes to 30:70 hexanes : CH_2Cl_2 to afford compound **16h** in 73% yield (148 mg, 0.378 mmol) as a colorless oil. ^{1}H NMR analysis of the crude reaction mixture indicated >20:1 dr.

Rf: 0.7 in 50:50 (Hexanes : CH_2Cl_2)

^{1}H NMR: (500 MHz, $CDCl_3$) δ = 7.64 (d, *J* = 8.4 Hz, 1H), 7.58 (bs, 1H), 7.09 (d, *J* = 3.6 Hz, 2H), 7.06 – 7.03 (m, 1H), 6.75 (s, 1H), 6.67 (dd, *J* = 13.4, 2.3 Hz, 1H), 6.39 (d, *J* = 8.6 Hz, 1H), 5.94 (t, *J* = 8.6 Hz, 1H), 5.29 (dddd, *J* = 16.8, 10.7, 7.9, 6.5 Hz, 1H), 4.89 – 4.85 (m, 2H), 3.57 (s, 3H), 2.67 – 2.56 (m, 2H), 2.55 – 2.47 (m, 2H), 2.41 (dd, *J* = 14.0, 8.0 Hz, 1H), 1.94 – 1.88 (m, 1H), 1.86 – 1.78 (m, 2H), 1.52 (s, 3H).

^{13}C NMR: (125 MHz, $CDCl_3$) δ = 212.1, 136.4, 134.4, 132.8, 132.7, 126.2, 122.7, 121.7, 121.1, 120.7, 119.2, 118.1, 117.6, 113.1, 113.0, 111.6, 110.5, 56.0, 54.2, 48.9, 46.4, 39.2, 34.1, 27.4, 18.7.

IR: *f* (cm^{-1}) = 3404, 3068, 2962, 2929, 2863, 1692, 1619, 1584, 1460, 1274, 1220, 1107.

HRMS: (ESI-TOF) *m/z*: $(M+H)^+$ = 392.2021 calculated for $C_{25}H_{27}FNO_2$; Found 392.2030.

(±)-(2*R*,6*R*)-2-allyl-6-(1*H*-indol-3-yl)-6-methyl-2-(4-(trifluoromethyl)phenyl)cyclohexanone (16i)

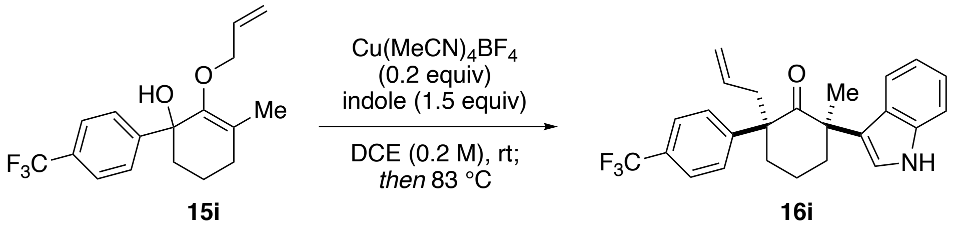

Procedure: α-Hydroxyl enol ether **15i** (202 mg, 0.647 mmol) was dissolved in DCE (3.2 mL, 0.2 M). Indole (151 mg, 1.293 mmol) was added, followed by addition of $Cu(MeCN)_4BF_4$ (101 mg, 0.3233 mmol). The reaction mixture was stirred at room temperature for 96 hours, at which the starting material was fully consumed as monitored by TLC. The solution was then heated to 83 °C in a preheated oil bath for 28 hours. Upon complete Claisen rearrangement as monitored by TLC, the crude reaction mixture was concentrated under vacuum and purified by column chromatography using 100% hexanes to 30:70 hexanes : CH_2Cl_2 to afford compound **16i** in 62% yield (165 mg, 0.401 mmol) as a white solid. ^{1}H NMR analysis of the crude reaction mixture indicated >20:1 dr.

Rf: 0.5 in 50:50 (Hexanes : CH_2Cl_2)

^{1}H NMR: (500 MHz, $CDCl_3$) δ = 7.63 (d, *J* = 7.9 Hz, 1H), 7.45 (bs, 1H), 7.10 – 7.07 (m, 1H), 7.07 – 7.04 (m, 2H), 6.87 (d, *J* = 8.2 Hz, 2H), 6.73 (d, *J* = 8.3 Hz, 2H), 6.69 (d, *J* = 2.6 Hz, 1H), 5.23 (dddd, *J* = 14.7, 10.4, 8.1, 6.7 1H), 4.88 – 4.84 (m, 2H), 2.68 – 2.55 (m, 3H), 2.54 – 2.48 (m, 1H), 2.44 (dd, *J* = 14.1, 8.0 Hz, 1H), 1.95 (td, *J* = 13.5, 4.4 Hz, 1H), 1.89 – 1.79 (m, 2H), 1.54 (s, 3H).

^{13}**C NMR:** (125 MHz, $CDCl_3$) δ = 211.5, 136.4, 133.9, 126.0, 125.9, 123.3, 123.2, 122.0, 120.9, 120.9, 119.4, 118.0, 117.4, 110.8, 55.1, 49.2, 46.4, 39.5, 34.0, 27.2, 18.8.

IR: *f* (cm^{-1}) = 3361, 3051, 2963, 2928, 2861, 1680, 1636, 1458, 1374, 1243, 1194, 1113, 998.

HRMS: (ESI-TOF) *m/z*: $(M+H)^+$ = 412.1883 calculated for $C_{25}H_{25}F_3NO$; Found 412.1886.

(±)-(2*R*,6*R*)-2-allyl-6-(1*H*-indol-3-yl)-6-methyl-2-(9-methyl-9*H*-carbazol-3-yl)cyclohexanone (16j)

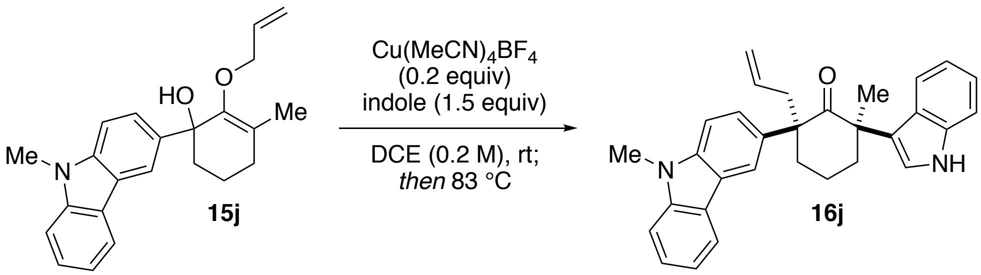

Procedure: α-Hydroxyl enol ether **15j** (172 mg, 0.495 mmol) was dissolved in DCE (2.5 mL, 0.2 M). Indole (86 mg, 0.743 mmol) was added, followed by addition of $Cu(MeCN)_4BF_4$ (31 mg, 0.990 mmol). The reaction mixture was stirred at room temperature for 15 hours, at which the starting material was fully consumed as monitored by TLC. The solution was then heated to 83 °C in a preheated oil bath for 24 hours. Upon complete Claisen rearrangement as monitored by TLC, the crude reaction mixture was concentrated under vacuum and purified by column chromatography using 100% hexanes to 40:60 hexanes : CH_2Cl_2 to afford compound **16j** in 69% yield (153 mg, 0.343 mmol) as a white solid. ^{1}H NMR analysis of the crude reaction mixture indicated >20:1 dr.

Rf: 0.6 in 30:70 (Hexanes : CH_2Cl_2)

^{1}**H NMR** (500 MHz, $CDCl_3$) δ = 7.82 (d, J = 8.0 Hz, 1H), 7.70 (d, J = 7.7 Hz, 1H), 7.59 (d, J = 1.9 Hz, 1H), 7.40 (ddd, J = 8.2, 7.1, 1.2 Hz, 1H), 7.27 – 7.24 (m, 1H), 7.13 (ddd, J = 7.9, 7.1, 1.0

Hz, 1H), 7.09 – 7.04 (m, 2H), 6.96 (ddd, *J* = 8.1, 6.9, 1.1 Hz, 1H), 6.91 (dd, *J* = 8.6, 1.9 Hz, 1H), 6.73 (d, *J* = 8.1Hz, 1H), 6.54 – 6.49 (m, 2H), 5.41 – 5.33 (m, 1H), 4.94 – 4.87 (m, 2H), 3.58 (s, 3H), 2.85 – 2.78 (m, 1H), 2.75 – 2.72 (m, 1H), 2.62 – 2.52 (m, 3H), 1.99 – 1.87 (m, 3H), 1.59 (s, 3H).

^{13}C NMR: (125 MHz, $CDCl_3$) δ = 213.3, 140.8, 139.0, 136.1, 135.1, 130.6, 126.2, 125.1, 124.5, 122.6, 121.6, 121.4, 121.4, 120.4, 120.3, 119.1, 118.6, 118.3, 117.2, 117.1, 110.3, 108.0, 106.5, 55.3, 48.9, 47.0, 39.3, 34.0, 28.8, 27.5, 18.9.

IR: *f* (cm^{-1}) = 3415, 3052, 2925, 2864, 1688, 1601, 1482, 1482, 1333, 1248, 1011.

HRMS: (ESI-TOF) *m/z*: $(M+H)^+$ = 447.2431 calculated for $C_{31}H_{31}N_2O$; Found 447.2433.

(±)-(2*S*,6*R*)-2-allyl-2-(benzo[*b*]thiophen-2-yl)-6-(1*H*-indol-3-yl)-6-methylcyclohexanone (16k)

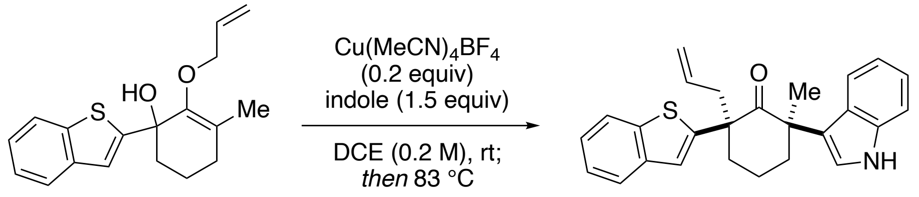

15k **16k**

Procedure: α-Hydroxyl enol ether **15k** (188 mg, 0.626 mmol) was dissolved in DCE (3.1 mL, 0.2 M). Indole (110 mg, 0.939 mmol) was added, followed by addition of $Cu(MeCN)_4BF_4$ (39 mg, 0.125 mmol). The reaction mixture was stirred at room temperature for 15 hours, at which the starting material was fully consumed as monitored by TLC. The solution was then heated to 83 °C in a preheated oil bath for 24 hours. Upon complete Claisen rearrangement as monitored by TLC, the crude reaction mixture was concentrated under vacuum and purified by column chromatography using 100% hexanes to 50:50 hexanes : CH_2Cl_2 to afford compound **16k** in 71%

yield (178 mg, 0.446 mmol) as a yellow solid. ^{1}H NMR analysis of the crude reaction mixture indicated >20:1 dr.

Rf: 0.7 in 50:50 (Hexanes : CH_2Cl_2)

^{1}H NMR: (500 MHz, $CDCl_3$) δ = 7.77 – 7.75 (m, 1H), 7.53 (d, *J* = 8.2 Hz, 1H), 7.43 (bs, 1H), 7.12 – 7.08 (m, 1H), 7.08 – 7.04 (m, 3H), 7.00 – 6.97 (m, 1H), 6.95 (d, *J* = 8.0 Hz, 1H), 6.73 (d, *J* = 2.6 Hz, 1H), 6.32 (s, 1H), 5.55 (dddd, *J* = 17.0, 10.2, 7.8, 6.7 Hz, 1H), 4.97 – 4.96 (m, 1H), 2.78 – 2.73 (m, 1H), 2.72 – 2.66 (m, 1H), 2.65 – 2.52 (m, 2H), 2.52 – 2.46 (m, 1H), 2.07 – 2.00 (m, 1H), 1.99 – 1.86 (m, 2H), 1.59 (s, 3H).

^{13}C NMR: (125 MHz, $CDCl_3$) δ = 210.9, 146.3, 139.3, 138.3, 136.5, 134.2, 125.9, 123.4, 123.3, 122.9, 121.8, 121.8, 121.3, 121.1, 120.9, 119.4, 118.5, 118.1, 110.8, 54.0, 49.0, 47.0, 38.0, 35.5, 27.3, 18.8.

IR: *f* (cm^{-1}) = 3414, 3055, 2961, 2927, 1695, 1458, 1246, 1105, 910.

HRMS: (ESI-TOF) *m/z*: $(M+H)^+$ = 400.1730 calculated for $C_{26}H_{26}NOS$; Found 400.1736.

(±)-(2*S*,6*R*)-2-allyl-6-(1*H*-indol-3-yl)-6-methyl-2-(3-methylthiophen-2-yl)cyclohexanone (16l)

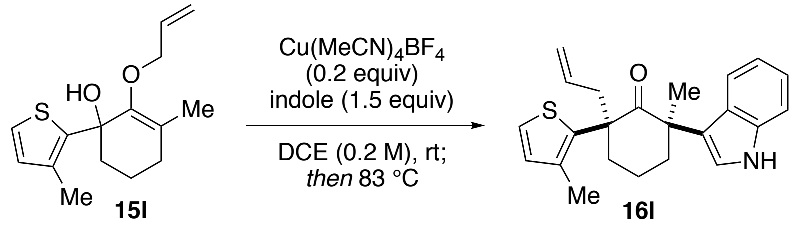

Procedure: α-Hydroxyl enol ether **15l** (166 mg, 0.628 mmol) was dissolved in DCE (3.1 mL, 0.2 M). Indole (110 mg, 0.942 mmol) was added, followed by addition of $Cu(MeCN)_4BF_4$ (40 mg, 0.126 mmol). The reaction mixture was stirred at room temperature for 9 hours, at which the starting material was fully consumed as monitored by TLC. The solution was then heated to 83

°C in a preheated oil bath for 15 hours. Upon complete Claisen rearrangement as monitored by TLC, the crude reaction mixture was concentrated under vacuum and purified by column chromatography using 100% hexanes to 50:50 hexanes : CH_2Cl_2 to afford compound **16l** in 95% yield (216 mg, 0.594 mmol) as a brown solid. ^{1}H NMR analysis of the crude reaction mixture indicated >20:1 dr.

Rf: 0.7 in 50:50 (Hexanes : CH_2Cl_2)

^{1}H NMR: (500 MHz, $CDCl_3$) δ = 7.72 (d, J = 8.1 Hz, 1H), 7.69 (s, 1H), 7.17 (d, J = 8.2, 1H), 7.10 – 7.07 (m, 1H), 6.99 – 6.94 (m, 1H), 6.85 (d, J = 2.3 Hz, 1H), 6.72 (d, J = 5.2 Hz, 1H), 6.15 (d, J = 5.1 Hz, 1H), 5.44 – 5.35 (m, 1H), 4.98 – 4.91 (m, 2H), 3.01 – 2.97 (m, 1H), 2.81 – 2.61 (m, 3H), 2.41 (dd, J = 14.2, 7.9 Hz, 1H), 1.95 – 1.85 (m, 3H), 1.61 (s, 3H), 1.59 (s, 3H).

^{13}C NMR: (125 MHz, $CDCl_3$) δ = 211.3, 136.9, 136.5, 135.1, 134.4, 131.4, 126.0, 121.7, 121.6, 120.9, 120.4, 118.8, 118.8, 117.5, 110.5, 52.9, 49.0, 42.9, 37.7, 37.2, 27.1, 18.8, 15.1.

IR: f (cm^{-1}) = 3408, 3069, 2967, 2867, 1691, 1514, 1458, 1337, 1243, 178, 1106, 1012, 911.

HRMS: (ESI-TOF) m/z: $(M+H)^+$ = 364.1730 calculated for $C_{23}H_{26}NOS$; Found 364.1743.

X-Ray Structure: Crystal growth was performed via slow evaporation using 5% CH_2Cl_2 in hexanes.

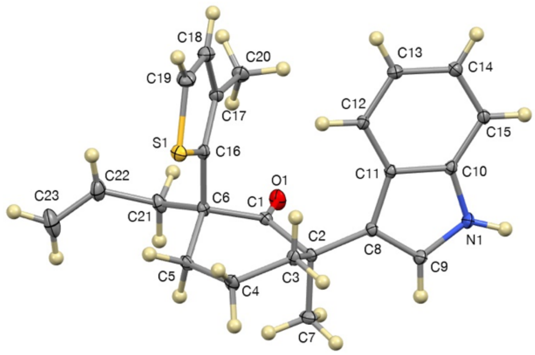

(±)-(2*R*,6*R*)-2-(1*H*-indol-3-yl)-2-methyl-6-(2-methylallyl)-6-phenylcyclohexanone (18a)

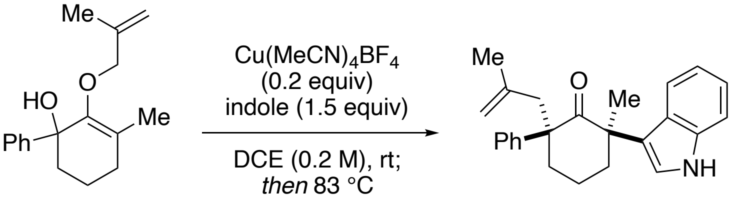

17a 18a

Procedure: α-Hydroxyl enol ether **17a** (176 mg, 0.681 mmol) was dissolved in DCE (3.4 mL, 0.2 M). Indole (119 mg, 1.022 mmol) was added, followed by addition of $Cu(MeCN)_4BF_4$ (42 mg, 0.136 mmol). The reaction mixture was stirred at room temperature for 16 hours, at which the starting material was fully consumed as monitored by TLC. The solution was then heated to 83 °C in a preheated oil bath for 24 hours. Upon complete Claisen rearrangement as monitored by TLC, the crude reaction mixture was concentrated under vacuum and purified by column chromatography using 100% hexanes to 50:50 hexanes : CH_2Cl_2 to afford compound **18a** in 70% yield (171 mg, 0.252 mmol) as an orange solid. ^{1}H NMR analysis of the crude reaction mixture indicated >20:1 dr.

Rf: 0.6 in 50:50 (Hexanes : CH_2Cl_2)

^{1}H NMR: (500 MHz, $CDCl_3$) δ = 7.70 (d, *J* = 8.0 Hz, 1H), 7.45 (s, 1H), 7.09 – 7.01 (m, 3H), 6.88 – 6.84 (m, 2H), 6.70 – 6.65 (m, 2H), 6.58 (t, *J* = 7.6 Hz, 2H), 4.69 – 4.67 (m, 1H), 4.43 – 4.41 (m, 1H), 2.77 – 2.68 (m, 1H), 2.67 – 2.48 (m, 4H), 1.97 – 1.80 (m, 3H), 1.55 (s, 3H), 1.07 (s, 3H).

^{13}C NMR: (125 MHz, $CDCl_3$) δ = 212.6, 142.9, 139.7, 136.4, 126.7, 126.2, 126.1, 125.3, 121.6, 121.3, 120.8, 119.1, 118.4, 114.7, 110.5, 54.9, 49.6, 49.1, 39.1, 33.5, 27.6, 24.2, 18.8.

IR: *f* (cm^{-1}) = 3433, 2962, 2927, 2854, 1685, 1639, 1334, 1245, 1188, 1082, 926.

HRMS: (ESI-TOF) *m/z*: $(M+H)^+$ = 358.1166 calculated for $C_{25}H_{28}NO$; Found 358.2176.

X-Ray Structure: Crystal growth was performed via slow evaporation using 5% CH_2Cl_2 in hexanes.

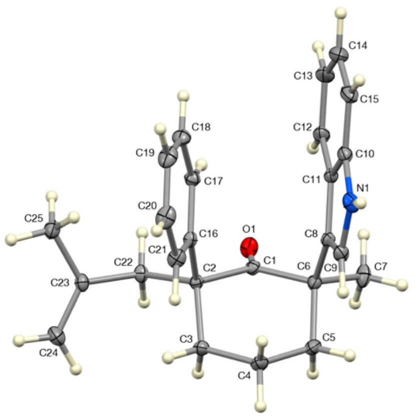

(±)-(2*R*,6*R*)-2-(1*H*-indol-3-yl)-2-methyl-6-(2-methyleneoctyl)-6-phenylcyclohexan-1-one (18b)

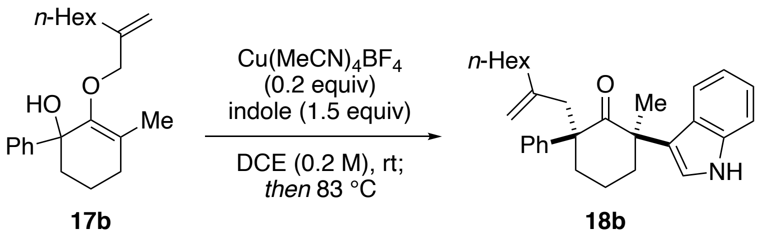

Procedure: α-Hydroxyl enol ether **17b** (89 mg, 0.270 mmol) was dissolved in DCE (1.3 mL, 0.2 M). Indole (48 mg, 0.406 mmol) was added, followed by addition of $Cu(MeCN)_4BF_4$ (17 mg, 0.054 mmol). The reaction mixture was stirred at room temperature for 16 hours, at which the starting material was fully consumed as monitored by TLC. The solution was then heated to 83 °C in a preheated oil bath for 60 hours. Upon complete Claisen rearrangement as monitored by TLC, the crude reaction mixture was concentrated under vacuum and purified by column chromatography using 100% hexanes to 50:50 hexanes : CH_2Cl_2 to afford compound **18b** in 52% yield (60 mg, 0.140 mmol) as a purple oil. ^1H NMR analysis of the crude reaction mixture indicated >20:1 dr.

Rf: 0.7 in 50:50 (Hexanes : CH_2Cl_2)

^{1}H NMR: (500 MHz, $CDCl_3$) δ = 7.72 (d, *J* = 7.5 Hz, 1H), 7.46 (bs, 1H), 7.09 – 7.03 (m, 3H), 6.84 (d, *J* = 7.2 Hz, 2H), 6.69 – 6.65 (m, 2H), 6.56 (t, *J* = 7.8 Hz, 2H), 4.67 (s, 1H), 4.42 (s, 1H), 2.75 – 2.68 (m, 2H), 2.60 – 2.55 (m, 1H), 2.52 – 2.44 (m, 2H), 1.97 – 1.77 (m, 3H), 1.56 (s, 3H), 1.32 – 1.26 (m, 1H), 1.20 – 1.13 (m, 2H), 1.11 – 1.02 (m, 5H), 1.00 – 0.87 (m, 2H), 0.80 (t, *J* = 7.3 Hz, 3H).

^{13}C NMR: (125 MHz, $CDCl_3$) δ = 212.8, 147.0, 139.6, 136.4, 126.6, 126.2, 126.1, 125.3, 121.5, 121.3, 120.8, 119.1, 118.5, 113.4, 110.5, 55.1, 49.1, 47.3, 39.1, 37.1, 33.5, 31.6, 28.8, 27.7, 27.6, 22.5, 18.8, 14.0.

IR: *f* (cm^{-1}) = 3401, 2955, 2924, 2856, 1691, 1636, 1459, 1373, 1245, 1106, 1083, 894.

HRMS: (ESI-TOF) *m/z*: $(M+H)^+$ = 428.2948 calculated for $C_{30}H_{38}NO$; Found 428.2945.

(±)-(2*R*,6*R*)-2-(2-benzylallyl)-6-(1*H*-indol-3-yl)-6-methyl-2-phenylcyclohexan-1-one (18c)

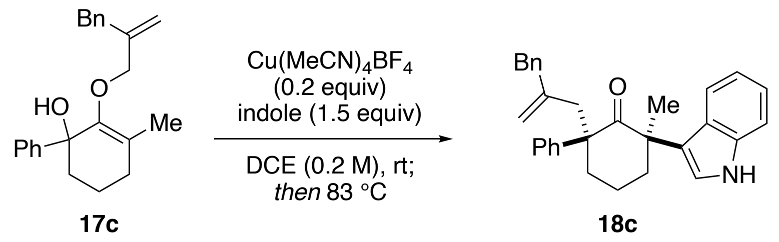

Procedure: α-Hydroxyl enol ether **17c** (118 mg, 0.353 mmol) was dissolved in DCE (1.8 mL, 0.2 M). Indole (62 mg, 0.529 mmol) was added, followed by addition of $Cu(MeCN)_4BF_4$ (22 mg, 0.070 mmol). The reaction mixture was stirred at room temperature for 23 hours, at which the starting material was fully consumed as monitored by TLC. The solution was then heated to 83 °C in a preheated oil bath for 63 hours. Upon complete Claisen rearrangement as monitored by TLC, the crude reaction mixture was concentrated under vacuum and purified by column chromatography using 100% hexanes to 65:35 hexanes : CH_2Cl_2 to afford compound **18c** in 51%

yield (78 mg, 0.180 mmol) as white solid. ^{1}H NMR analysis of the crude reaction mixture indicated >20:1 dr.

Rf: 0.5 in 50:50 (Hexanes : CH_2Cl_2)

^{1}H NMR (500 MHz, $CDCl_3$) δ = 7.70 (d, *J* = 7.9 Hz, 1H), 7.50 – 7.45 (m, 1H), 7.16 – 7.13 (m, 2H), 7.12 – 7.03 (m, 4H), 6.85 (d, *J* = 7.1 Hz, 2H), 6.76 (d, *J* = 6.9 Hz, 2H), 6.74 – 6.68 (m, 2H), 6.60 (t, *J* = 7.6 Hz, 2H), 4.62 (s, 1H), 4.61 (s, 1H), 2.86 – 2.81 (m, 1H), 2.71 (d, *J* = 13.7 Hz, 1H), 2.61 – 2.48 (m, 3H), 2.37 (d, *J* = 13.6 Hz, 1H), 2.20 (d, *J* = 15.4 Hz, 1H), 1.97 – 1.86 (m, 2H), 1.84 – 1.77 (m, 1H), 1.55 (s, 3H).

^{13}C NMR (125 MHz, $CDCl_3$) δ = 212.6, 145.9, 139.5, 139.2, 136.3, 129.0, 127.9, 126.7, 126.2, 126.2, 125.7, 125.5, 121.5, 121.3, 120.7, 119.1, 118.4, 116.2, 110.5, 54.9, 49.1, 46.7, 43.6, 39.3, 33.9, 27.6, 18.8.

IR: *f* (cm^{-1}) = 3410, 2924, 1691, 1690, 1453.

HRMS: (ESI-TOF) *m/z*: $(M+H)^{+}$ = 434.2478 calculated for $C_{31}H_{32}NO$; Found 434.2474.

(±)-(2*R*,6*R*)-2-(2-bromoallyl)-6-(1*H*-indol-3-yl)-6-methyl-2-phenylcyclohexan-1-one (18d)

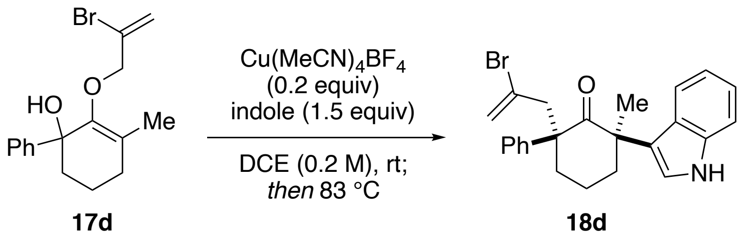

17d **18d**

Procedure: α-Hydroxyl enol ether **17d** (126 mg, 0.389 mmol) was dissolved in DCE (1.3 mL, 0.2 M). Indole (68 mg, 0.584 mmol) was added, followed by addition of $Cu(MeCN)_4BF_4$ (24 mg, 0.077 mmol). The reaction mixture was stirred at room temperature for 15 hours, at which the starting material was fully consumed as monitored by TLC. The solution was then heated to 83 °C in a preheated oil bath for 264 hours (11 days). Upon complete Claisen rearrangement as monitored

by TLC, the crude reaction mixture was concentrated under vacuum and purified by column chromatography using 100% hexanes to 50:50 hexanes : CH_2Cl_2 to afford compound **18d** in 47% yield (78 mg, 0.184 mmol) as a purple oil. ^{1}H NMR analysis of the crude reaction mixture indicated >20:1 dr.

Rf: 0.7 in 50:50 (Hexanes : CH_2Cl_2)

^{1}H NMR: (500 MHz, $CDCl_3$) δ = 7.70 (d, *J* = 7.6 Hz, 1H), 7.46 (s, 1H), 7.10 – 7.03 (m, 3H), 6.83 – 6.80 (m, 2H), 6.70 – 6.66 (m, 2H), 6.57 (t, *J* = 7.8 Hz, 2H), 5.26 (s, 1H), 4.88 (s, 1H), 3.10 – 3.05 (m, 1H), 3.02 – 2.90 (m, 2H), 2.61 – 2.49 (m, 2H), 2.07 – 1.86 (m, 3H), 1.56 (s, 3H).

^{13}C NMR: (125 MHz, $CDCl_3$) δ = 211.7, 138.3, 136.4, 128.4, 126.7, 126.2, 125.7, 121.6, 121.2, 121.1, 120.8, 119.2, 118.2, 110.6, 56.1, 52.0, 49.0, 39.1, 33.5, 27.6, 18.8.

IR: *f* (cm^{-1}) = 3408, 2923, 1691, 1620, 1459, 742.

HRMS: (ESI-TOF) *m/z*: $(M+H)^+$ = 422.1114 calculated for $C_{24}H_{25}BrNO$; Found 422.1111.

CHARACTERIZATION OF PRODUCTS IN SCHEME 4

1. Oxygen Free Conditions

(±)-(2*R*,6*R*)-2-allyl-6-(1*H*-indol-3-yl)-6-methyl-2-phenylcyclohexan-1-one (*cis*-13)

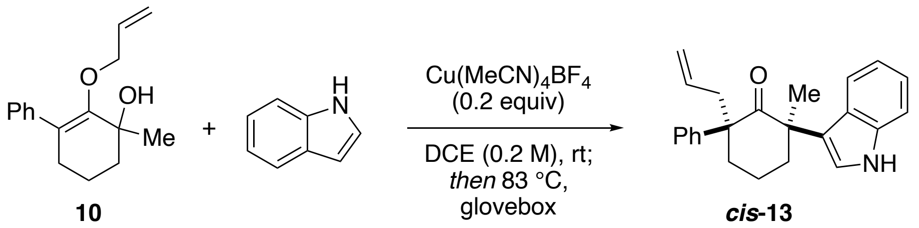

Procedure: In a glovebox, α-hydroxyl enol ether **10** (111 mg, 0.454 mmol) was dissolved in N_2 degassed DCE (2.2 mL, 0.2 M). Indole (80 mg, 0.681 mmol) was added, followed by addition of $Cu(MeCN)_4BF_4$ (29 mg, 0.091 mmol). The solution in a sealed vial was removed from the glovebox and then stirred at room temperature for 24 hours. The solution was subsequently heated to 83 °C in a preheated oil bath for 36 hours. The crude reaction mixture was concentrated under vacuum and then purified by column chromatography using 100% hexanes to 90:10 hexanes : CH_2Cl_2 to afford compound ***cis*-13** in 86% yield (135 mg, 0.393 mmol) as a white solid. ^{1}H NMR analysis of the crude reaction mixture indicated >20:1 dr.

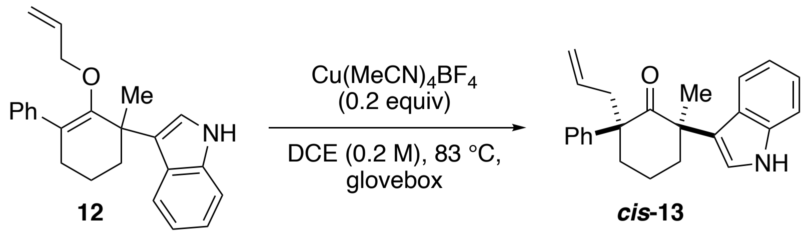

Procedure: In a glovebox, α-indolyl allylvinyl ether **12** (99 mg, 0.288 mmol) was dissolved in N_2 degassed DCE (0.7 mL, 0.2 M). The solution in a sealed vial was removed from the glovebox and

then heated to 83 °C in a preheated oil bath for 34 hours. The crude reaction mixture was concentrated under vacuum and then purified by column chromatography using 100% hexanes to 50:50 hexanes : CH_2Cl_2 to afford compound ***cis*-13** in 87% yield (87 mg, 0.253 mmol) as a white solid. ^{1}H NMR analysis of the crude reaction mixture indicated >20:1 dr.

2. Alkyl Substituent Effect

(±)-(2*R*,6*R*)-2-allyl-6-ethyl-6-(1*H*-indol-3-yl)-2-phenylcyclohexanone (*cis*-20) and (±)-(2*S*,6*R*)-2-allyl-6-ethyl-6-(1*H*-indol-3-yl)-2-phenylcyclohexan-1-one (*trans*-20)

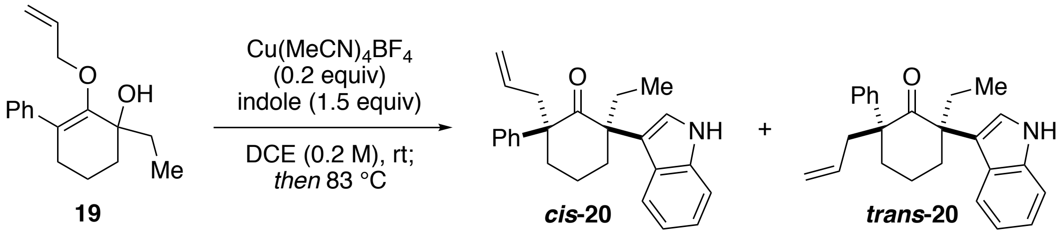

Procedure: α-Hydroxyl enol ether **19** (146 mg, 0.565 mmol) was dissolved in DCE (2.8 mL, 0.2 M). Indole (99 mg, 0.848 mmol) was added, followed by addition of $Cu(MeCN)_4BF_4$ (35 mg, 0.113 mmol). The reaction mixture was stirred at room temperature for 20 hours, at which the starting material was fully consumed as monitored by TLC. The solution was then heated to 83 °C in a preheated oil bath for 42 hours. Upon complete Claisen rearrangement as monitored by TLC, the crude reaction mixture was concentrated under vacuum and purified by column chromatography using 100% hexanes to 50:50 hexanes : CH_2Cl_2 to afford compound ***cis*-20** in 32% yield (66 mg, 0.184 mmol) as a white solid and ***trans*-20** in 32% yield (64 mg, 0.179 mmol) as a colorless oil. ^{1}H NMR analysis of the crude reaction mixture indicated 1:1.2 dr (***cis*-20**: ***trans*-20**).

Product ***cis*-20**

Rf: 0.6 in 50:50 (Hexanes : CH_2Cl_2)

^{1}H NMR: (500 MHz, $CDCl_3$) δ = 7.75 (d, *J* = 8.4 Hz, 1H), 7.49 (s, 1H), 7.09 – 7.02 (m, 3H), 6.80 (d, *J* = 8.2 Hz, 2H), 6.70 – 6.64 (m, 2H), 6.57 (t, *J* = 7.7 Hz, 2H), 5.36 – 5.26 (m, 1H), 4.89 (bs, 1H), 4.88 – 4.84 (m, 1H), 2.64 – 2.40 (m, 6H), 1.90 – 1.74 (m, 4H), 0.56 (t, *J* = 7.5 Hz, 3H).

^{13}C NMR: (125 MHz, $CDCl_3$) δ = 212.3, 139.9, 136.5, 134.8, 126.6, 126.2, 125.6, 125.2, 122.4, 121.5, 121.4, 119.1, 117.3, 115.8, 110.5, 55.3, 52.5, 46.8, 34.8, 33.1, 30.6, 18.5, 7.9.

IR: *ƒ* (cm^{-1}) = 3378, 2924, 2857, 1678, 1612, 1511, 1429, 1377, 1244, 1103, 1032, 912.

HRMS: (ESI-TOF) *m/z*: $(M+H)^+$ = 358.2166 calculated for $C_{25}H_{28}NO$; Found 358.2169.

X-Ray Structure: Crystal growth was performed via slow evaporation using 5% CH_2Cl_2 in hexanes.

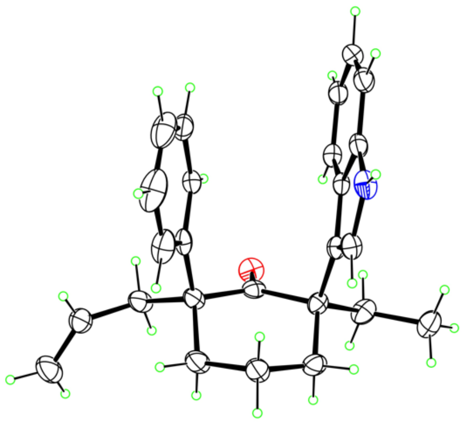

Product ***trans*-20**

Rf: 0.8 in 50:50 (Hexanes : CH_2Cl_2)

^{1}H NMR: (500 MHz, $CDCl_3$) δ = 8.10 (s, 1H), 7.80 (d, *J* = 8.1 Hz, 1H), 7.43 – 7.34 (m, 5H), 7.30 – 7.26 (m, 1H), 7.19 (ddd, *J* = 8.1, 7.0, 1.2 Hz, 1H), 7.11 (td, *J* = 7.5, 6.9, 1.1 Hz, 1H), 6.98 (d, *J* = 2.3 Hz, 1H), 5.39 – 5.30 (m, 1H), 4.88 – 4.79 (m, 2H), 2.63 (ddt, *J* = 13.9, 6.9, 1.4 Hz, 1H), 2.50

(dd, J = 13.9, 7.7 Hz, 1H), 2.41 – 2.30 (m, 2H), 2.04 – 1.92 (m, 2H), 1.90 – 1.82 (m, 2H), 1.80 – 1.69 (m, 2H), 0.57 (t, J = 7.4 Hz, 3H).

^{13}C NMR: (125 MHz, $CDCl_3$) δ = 213.2, 140.3, 136.9, 134.5, 128.5, 126.6, 126.3, 126.0, 122.1, 121.8, 121.3, 119.4, 117.6, 117.3, 111.3, 54.8, 52.9, 46.6, 31.2, 29.2, 28.0, 16.9, 8.8.

IR: *f* (cm^{-1}) = 3407, 2937, 2874, 1689, 1457, 1378, 1104, 998, 907.

HRMS: (ESI-TOF) *m/z*: $(M+H)^+$ = 358.2166 calculated for $C_{25}H_{28}NO$; Found 358.2169.

3. Crossover Experiment

(±)-(2*R*,6*R*)-2-allyl-6-(1*H*-indol-3-yl)-2-(4-methoxyphenyl)-6-methylcyclohexan-1-one (16a)

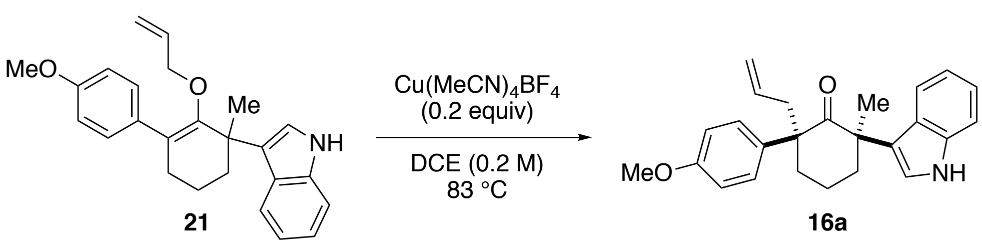

Procedure: α-Indolyl allylvinyl ether **21** (45 mg, 0.120 mmol) was dissolved in DCE (0.6 mL, 0.2 M). $Cu(MeCN)_4BF_4$ (8 mg, 0.024 mmol) was added. The reaction mixture was then heated to 83 °C in a preheated oil bath and stirred for 10 hours. Upon complete Claisen rearrangement as monitored by TLC, the crude reaction mixture was concentrated under vacuum and purified by column chromatography using 100% hexanes to 95:5 hexanes : EtOAc to afford compound **16a** in 52% yield (23 mg, 0.061 mmol) as a white solid. ^{1}H NMR analysis of the crude reaction mixture indicated >20:1 dr.

(±)-(2*R*,6*R*)-2-(allyl-2-*d*)-6-(1*H*-indol-3-yl)-6-methyl-2-phenylcyclohexan-1-one (22b)

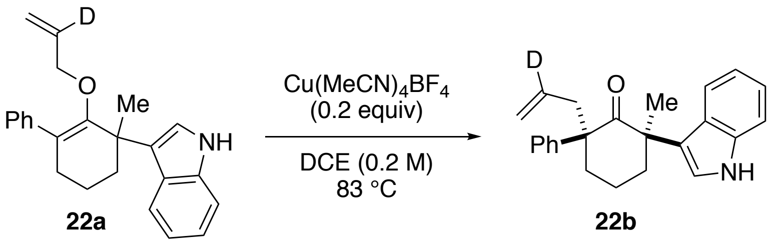

Procedure: α-Indolyl allylvinyl ether **22a** (45 mg, 0.130 mmol) was dissolved in DCE (0.65 mL, 0.2 M). $Cu(MeCN)_4BF_4$ (8 mg, 0.026 mmol) was added. The reaction mixture was then heated to 83 °C in a preheated oil bath and stirred for 10 hours. Upon complete Claisen rearrangement as monitored by TLC, the crude reaction mixture was concentrated under vacuum and purified by column chromatography using 100% hexanes to 95:5 hexanes : EtOAc to afford compound **22b** in 47% yield (21 mg, 0.061mmol) as a pink solid. ^{1}H NMR analysis of the crude reaction mixture indicated >20:1 dr.

Rf: 0.4 in 40:20 (Hexanes : CH_2Cl_2)

^{1}H NMR: (500 MHz, $CDCl_3$) δ = 7.70 (d, *J* = 7.8 Hz, 1H), 7.48 (s, 1H), 7.10 – 7.02 (m, 3H), 6.83 (d, *J* = 7.8 Hz, 2H), 6.71 – 6.66 (m, 2H), 6.59 (t, *J* = 7.7 Hz, 2H), 4.88 (s, 2H), 2.67 – 2.56 (m, 3H), 2.51 – 2.43 (m, 2H), 1.96 – 1.88 (m, 1H), 1.88 – 1.78 (m, 2H), 1.55 (s, 3H).

^{13}C NMR: (125 MHz, $CDCl_3$) δ = 212.7, 139.6, 136.4, 126.7, 126.2, 125.7, 125.3, 121.6, 121.3, 120.8, 119.1, 118.3, 117.2, 110.6, 55.2, 49.0, 46.6, 39.2, 33.5, 27.4, 18.7.

IR: *f* (cm^{-1}) = 3405, 2926, 1692, 1510, 1460, 1248.

HRMS: (ESI-TOF) *m/z*: $(M+H)^+$ = 345.2071 calculated for $C_{24}H_{25}DNO$; Found 345.2071.

X-Ray Structure: Crystal growth was performed via slow evaporation using 5% CH_2Cl_2 in hexanes.

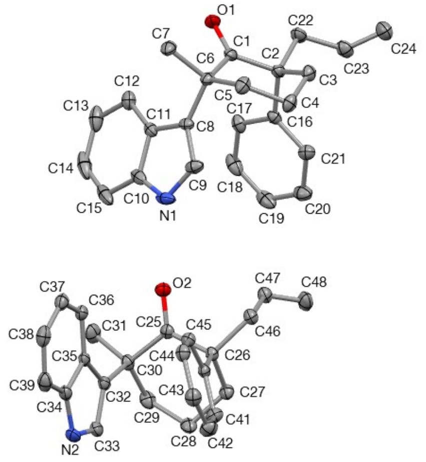

(±)-(2*R*,6*R*)-2-allyl-6-(1*H*-indol-3-yl)-2-(4-methoxyphenyl)-6-methylcyclohexan-1-one (16a) and (±)-(2*R*,6*R*)-2-(allyl-2-*d*)-6-(1*H*-indol-3-yl)-6-methyl-2-phenylcyclohexan-1-one (22b)

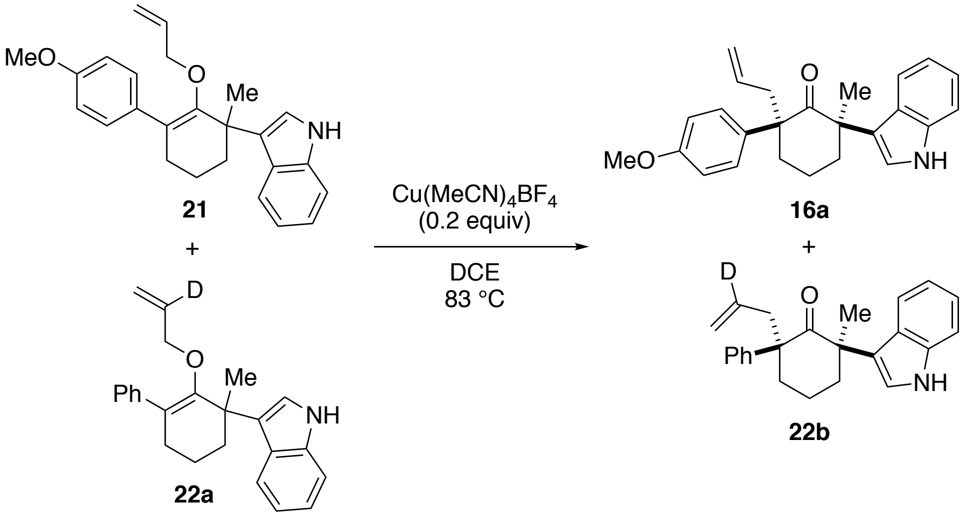

Procedure: α-Hydroxyl enol ether **21** (30 mg, 0.080 mmol) and α-hydroxyl enol ether **22a** (28 mg, 0.080 mmol) were dissolved in DCE (3.2 mL, 0.05 M). ^{1}H NMR spectrum of this mixture of starting materials was recorded, and it showed a 1:1.1 ratio of **21** : **22a**. $Cu(MeCN)_4BF_4$ (5 mg, 0.054 mmol) was then added. The reaction mixture was heated to 83 °C in a preheated oil bath

for 6 hours. Upon consumption of both starting materials as monitored by crude NMR, the reaction mixture was concentrated under vacuum. ^{1}H NMR spectrum of the crude reaction mixture was then again recorded, and it showed a 1:1.2 ratio of products **16a** : **22b**. Apart from minor decomposition materials, ^{1}H NMR signals corresponding to the crossover products were not observed. *Note:* this crossover reaction was conducted in a 0.05 M concentration to lessen the impact of decomposition, which was observed at a higher 0.2 M concentration.

The spectra below are comparative ^{1}H NMR analyses of starting materials **21** and **22a** as authentic samples and as a 1:1.1 mixture before the crossover experiment. The respective aromatic protons H_a in compound **21** and H_b in compound **22a** were readily distinguishable and quantifiable to determine the 1:1.1 ratio between the two compounds.

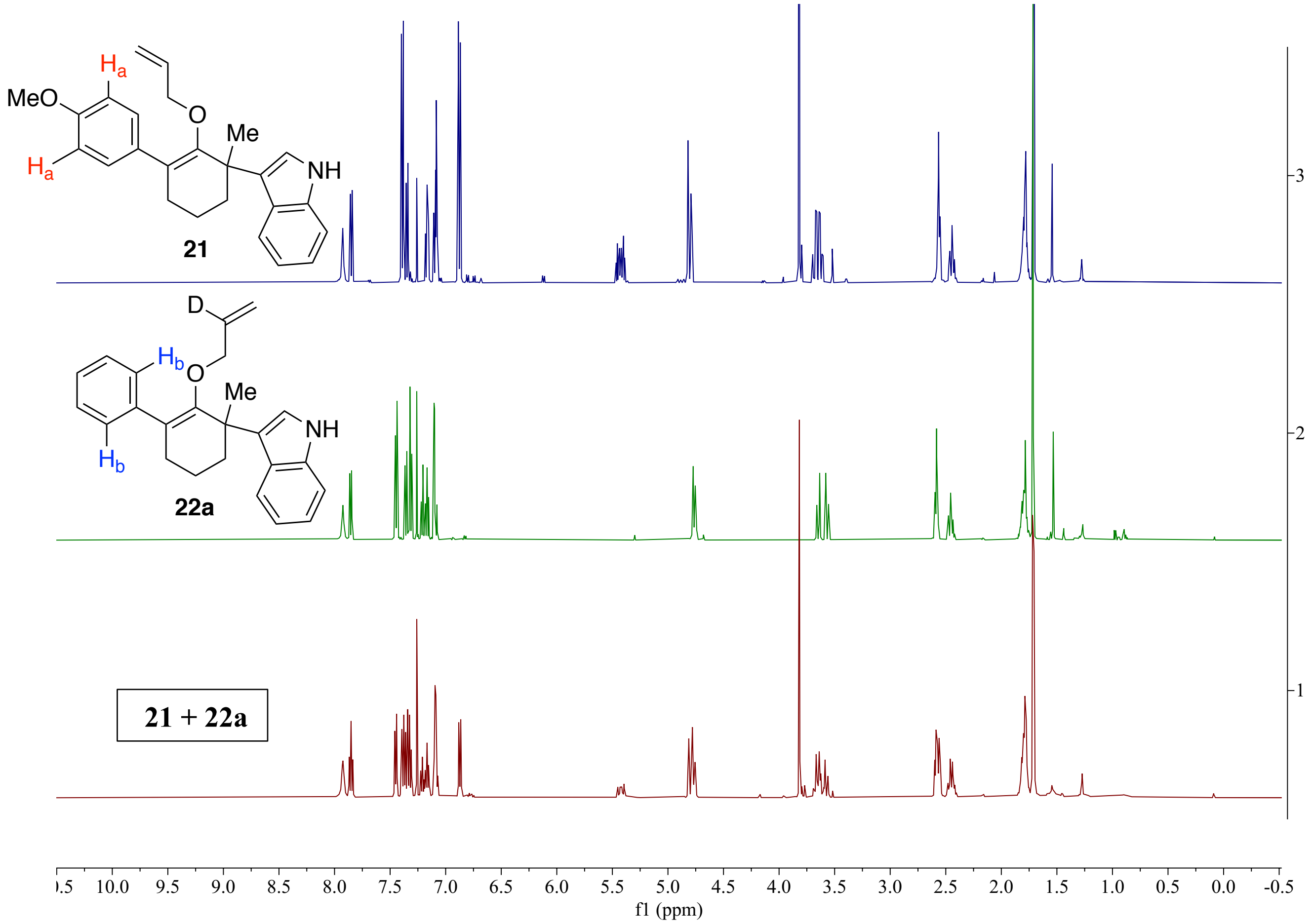

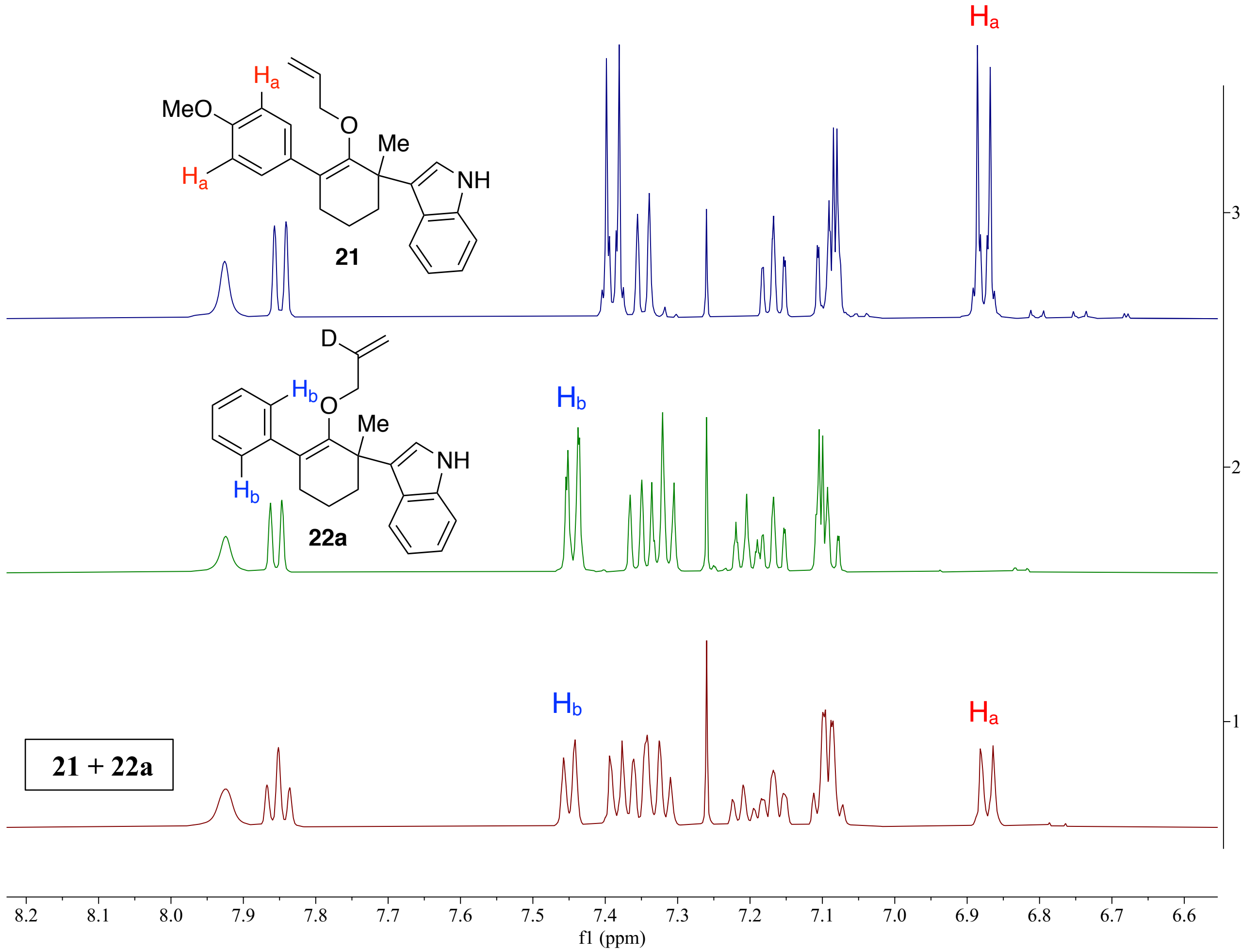

Ha
MeO
O
Me
NH
Ha
21
D
Hb
O
Me
NH
Hb
22a
Hb
Ha
21 + 22a
3
2
1
8.2 8.1 8.0 7.9 7.8 7.7 7.6 7.5 7.4 7.3 7.2 7.1 7.0 6.9 6.8 6.7 6.6
f1 (ppm)

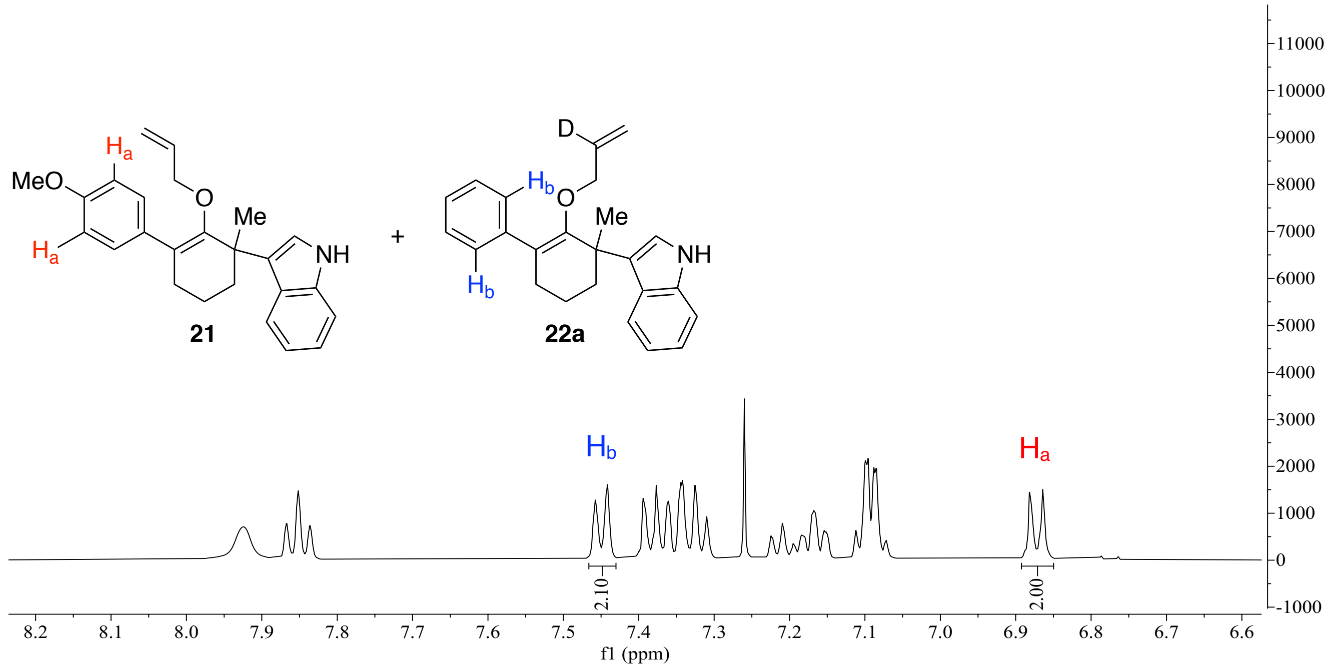

Ha
MeO
O
Me
NH
Ha
21
+
D
Hb
O
Me
NH
Hb
22a
Hb
Ha
2.10
2.00
11000
10000
9000
8000
7000
6000
5000
4000
3000
2000
1000
0
-1000
8.2 8.1 8.0 7.9 7.8 7.7 7.6 7.5 7.4 7.3 7.2 7.1 7.0 6.9 6.8 6.7 6.6
f1 (ppm)

The spectra below are comparative ^{1}H NMR analyses of Claisen rearrangement products **16a** and **22b** as authentic samples against the crude reaction mixture of the crossover experiment. The respective aromatic protons H_a in compound **16a** and H_c in compound **22b** were distinguishable and quantifiable to determine the 1:1.2 ratio between the two compounds.

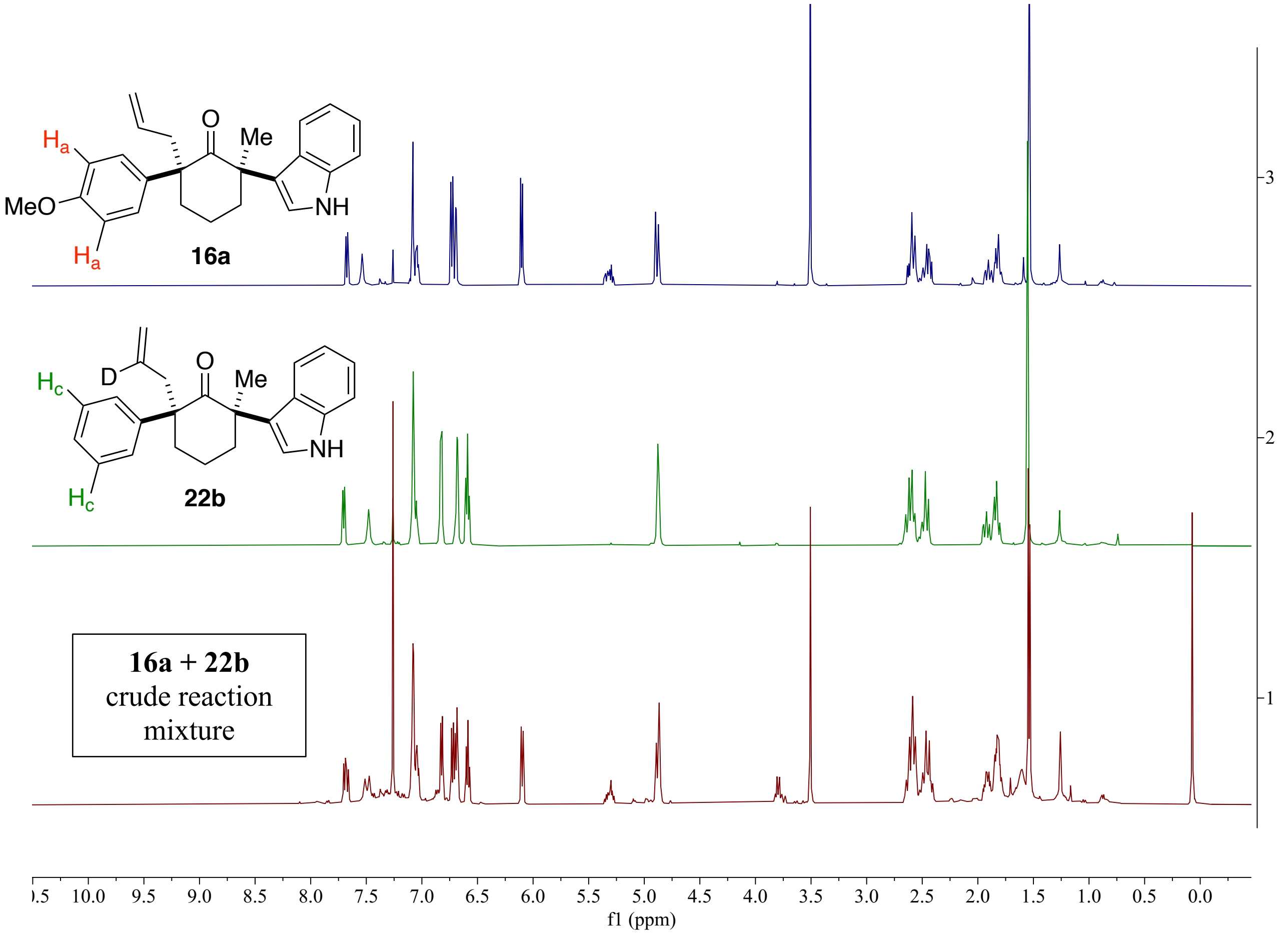

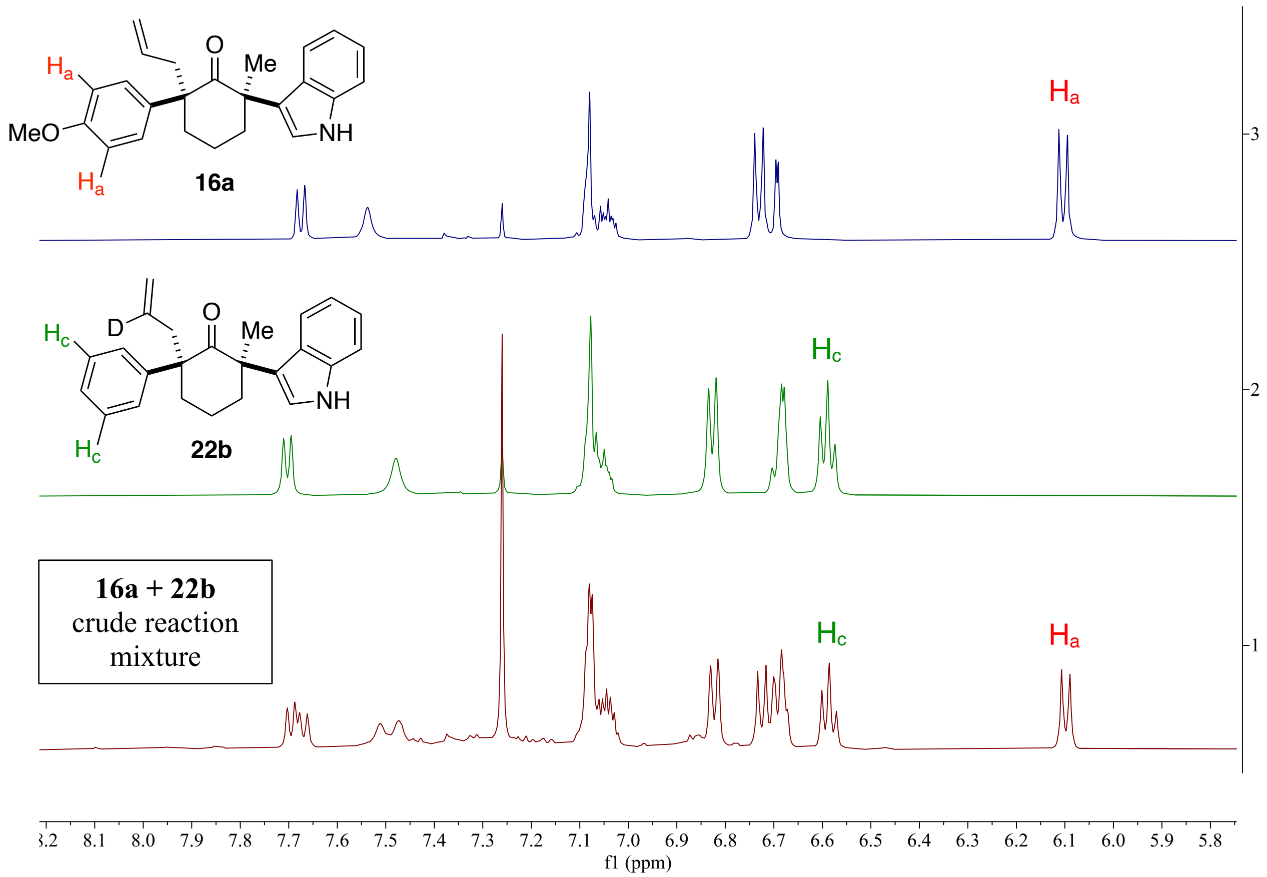
H
a
MeO
H
a
O
Me
NH
16a
H
a
D
H
c
O
Me
NH
H
c
22b
H
c
16a + 22b
crude reaction
mixture
H
c
H
a
f1 (ppm)

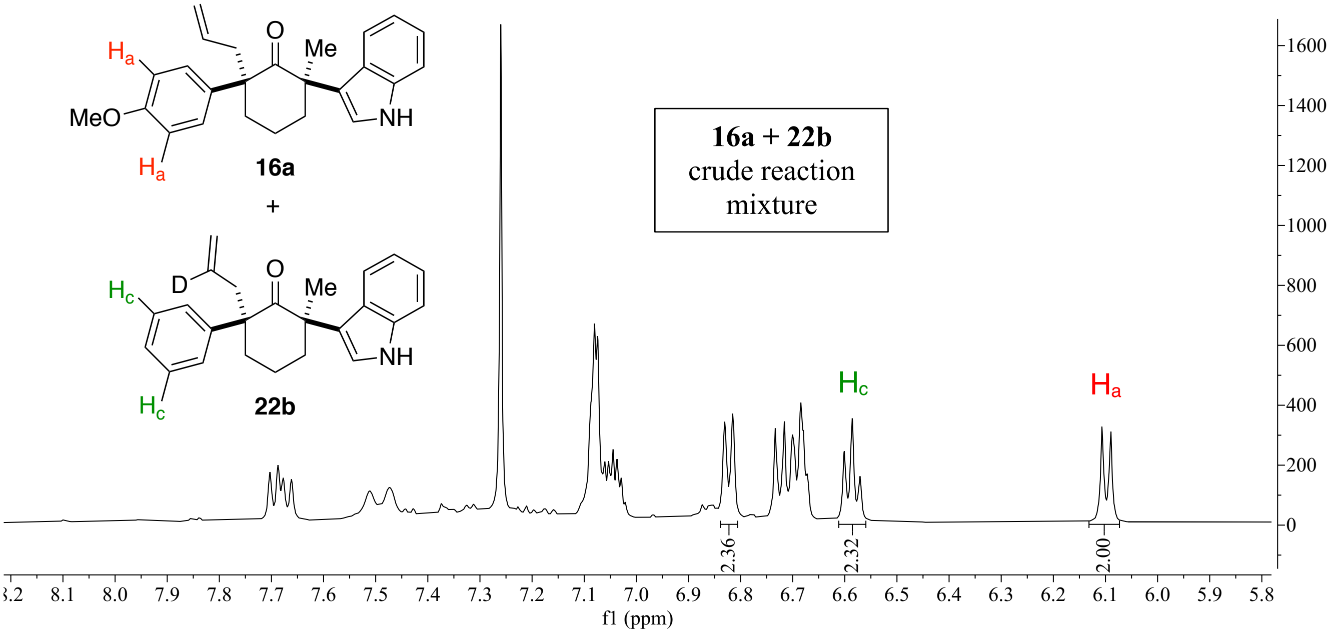
16a
+
22b
16a + 22b
crude reaction
mixture
H
c
H
a
2.36
2.32
2.00
f1 (ppm)

4. Isotopic Labeling

(±)-(2*R*,6*R*)-2-(allyl-1-d)-6-(1*H*-indol-3-yl)-6-methyl-2-phenylcyclohexan-1-one (23b)

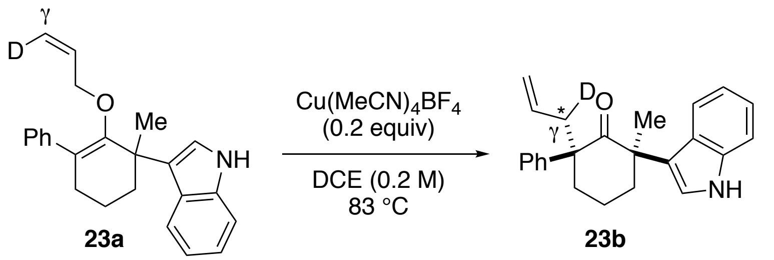

Procedure: α-Indolyl allylvinyl ether **23a** (50 mg, 0.145 mmol) was dissolved in DCE (0.7 mL, 0.2 M). $Cu(MeCN)_4$•BF_4 (9 mg, 0.029 mmol) was added. The reaction mixture was then heated to 83 °C in a preheated oil bath and stirred for 19 hours. Upon complete Claisen rearrangement as monitored by TLC, the crude reaction mixture was concentrated under vacuum and purified by column chromatography using 100% hexanes to 50:50 hexanes : CH_2Cl_2 to afford compound **23b** in 40% yield (20 mg, 0.058 mmol) as a white solid. ^{1}H NMR analysis of the crude reaction mixture indicated >20:1 dr. Attempts to unambiguously assign the relative stereochemistry of the deuterated γ-carbon were not successful.

Rf: 0.4 in 50:50 (Hexanes : CH_2Cl_2)

^{1}H NMR: (500 MHz, $CDCl_3$) δ = δ 7.70 (d, J = 8.7 Hz, 1H), 7.46 (bs, 1H), 7.10 – 7.01 (m, 3H), 6.82 (d, J = 8.2 Hz, 2H), 6.71 – 6.66 (m, 2H), 6.59 (t, J = 6.9 Hz, 2H), 5.34 – 5.25 (m, 1H), 4.89 (s, 1H), 4.88 – 4.84 (m, 1H), 2.66 – 2.55 (m, 2H), 2.52 – 2.42 (m, 2H), 1.97 – 1.88 (m, 1H), 1.87 – 1.79 (m, 2H), 1.55 (s, 3H).

^{13}C NMR: (125 MHz, $CDCl_3$) δ = 212.7, 139.6, 136.4, 134.7, 126.7, 126.2, 125.7, 125.3, 121.6, 121.3, 120.8, 119.2, 118.4, 117.4, 110.6, 55.1, 49.0, 39.2, 33.5, 27.4, 18.7, 18.6.

IR: f (cm^{-1}) = 2964 ,2927, 1687, 1493, 1447, 1371.

HRMS: (ESI-TOF) *m/z*: $(M+H)^+$ = 345.2072 calculated for $C_{24}H_{24}DNO$; Found 345.2074.

5. Concerning *cis* Stereochemistry

(±)-(2R,6R)-2-allyl-6-(1H-indol-3-yl)-2,6-dimethylcyclohexan-1-one (24b)

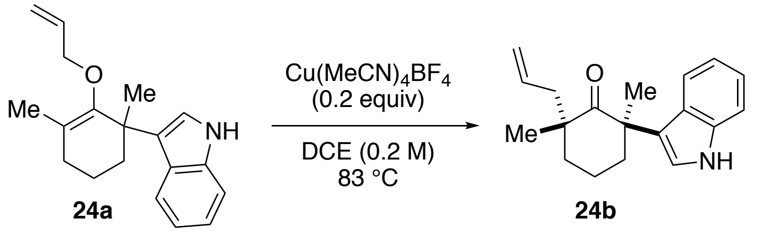

24a **24b**

Procedure: α-Indolyl allylvinyl ether **24a** (45 mg, 0.159 mmol) was dissolved in DCE (0.8 mL, 0.2 M) and $Cu(MeCN)_4BF_4$ (40 mg, 0.125 mmol) was added. The reaction mixture was then heated to 83 °C in a preheated oil bath and stirred for 17 hours. Upon complete Claisen rearrangement as monitored by TLC, the crude reaction mixture was concentrated under vacuum and purified by column chromatography using 100% hexanes to 95:5 hexanes : EtOAc to afford compound **24b** in 93% yield (42 mg, 0.149 mmol) as a tan solid. ^{1}H NMR analysis of the crude reaction mixture indicated >20:1 dr.

Rf: 0.4 in 50:50 (Hexanes : CH_2Cl_2)

^{1}H NMR: (500 MHz, $CDCl_3$) δ = 8.07 (bs, 1H), 7.64 (d, *J* = 8.1 Hz, 1H), 7.33 (d, *J* = 8.2 Hz, 1H), 7.16 (t, *J* = 8.1 Hz, 1H), 7.11 (d, *J* = 2.5 Hz, 1H), 7.06 (t, *J* = 8.1 Hz, 1H), 5.83 – 5.73 (m, 1H), 5.06 – 4.96 (m, 2H), 2.69 – 2.63 (m, 1H), 2.36 (dd, *J* = 13.8, 6.9 Hz, 1H), 2.26 – 2.16 (m, 2H), 1.86 – 1.73 (m, 3H), 1.69 – 1.62 (m, 1H), 1.47 (s, 3H), 0.68 (s, 3H).

^{13}C NMR: (125 MHz, $CDCl_3$) δ = 216.4, 136.7, 135.1, 126.1, 122.0, 120.8, 120.1, 119.6, 119.4, 117.5, 111.0, 48.5, 48.4, 44.0, 38.6, 37.8, 28.0, 24.2, 18.3.

IR: *f* (cm^{-1}) = 3313, 2966, 2915, 2860, 1682, 1456, 1426, 1338,1242, 1109, 991, 955, 913.

HRMS: (ESI-TOF) *m/z*: $(M+H)^+$ = 282.1853 calculated for $C_{19}H_{24}NO$; Found 282.1855.

X-Ray Structure: Crystal growth was performed via slow evaporation using 5% CH_2Cl_2 in hexanes.

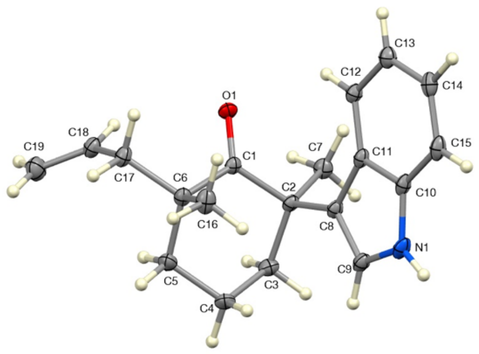

(±)-(2*R*,6*R*)-2-allyl-6-(1*H*-indol-3-yl)-2,6-dimethylcyclohexan-1-one (24b) and (±)-(2*S*,6*R*)-2-allyl-6-(1*H*-indol-3-yl)-2,6-dimethylcyclohexan-1-one (24c)

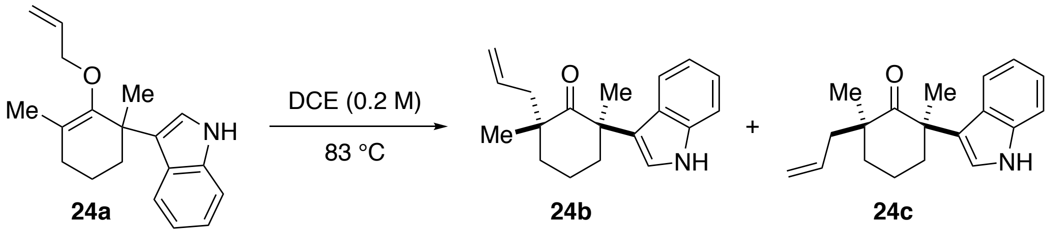

Procedure: α-Indolyl allylvinyl ether **24a** (100 mg, 0.355 mmol) was dissolved in DCE (1.7 mL, 0.2 M). The mixture was then heated to 83 °C in a preheated oil bath for 65 hours. Upon complete Claisen rearrangement as monitored by TLC, the crude reaction mixture was concentrated under vacuum and purified by column chromatography using 100% hexanes to 50:50 hexanes : CH_2Cl_2 to afford compound a chromatographically inseparable diastereomeric mixture of **24b** and **24c** in 89% yield (89 mg, 0.316 mmol) as a white solid. ^{1}H NMR analysis of the crude reaction mixture indicated 3.7:1 dr (**24b** : **24c**).

^{1}H NMR: (500 MHz, $CDCl_3$) δ 8.06 (s, 1.13 H), 7.67 (d, *J* = 8.1 Hz, 0.24 H), 7.64 (d, *J* = 8.1 Hz, 0.98 H), 7.33 – 7.31 (m, 1.19 H), 7.17 – 7.14 (m, 1.46 H), 7.12 – 7.11 (m, 1.0 H), 7.07 (ddd, *J* = 8.2, 6.9, 1.2 Hz, 1.23 H), 5.83 – 5.71 (m, 1.02 H), 5.35 – 5.29 (m, 0.26 H), 5.04 – 4.98 (m, 2.03 H), 4.76 – 4.79 (m, 0.25 H), 4.66 – 4.62 (m, 0.25 H), 2.67 – 2.63 (m, 1.25 H), 2.37 – 2.33 (m, 1H), 2.26 – 2.15 (m, 2.29 H), 1.97 – 1.71 (m, 4.52 H), 1.70 – 1.54 (m, 1.94 H), 1.51 (s, 0.75 H), 1.46 (d, *J* = 1.2 Hz, 3.0 H), 1.06 (s, 0.75 H), 0.68 (s, 3.0 H).

^{13}C NMR: (125 MHz, $CDCl_3$) δ = 216.9, 216.8, 136.9, 136.8, 135.2, 133.9, 126.1, 125.9, 122.0, 121.9, 121.1, 120.8, 120.5, 120.5, 120.4, 120.4, 119.6, 119.5, 119.3, 119.3, 119.2, 117.8, 117.7, 111.3, 111.3, 111.2, 111.2, 48.6, 48.6, 48.5, 48.5, 44.1, 42.7, 38.8, 38.7, 37.9, 37.8, 29.8, 28.0, 24.4, 24.3, 18.3, 18.2.

SYNTHESIS AND CHARACTERIZATION OF SUBSTRATES

2-(allyloxy)-3-methyl-3,4,5,6-tetrahydro-[1,1'-biphenyl]-3-ol (10)

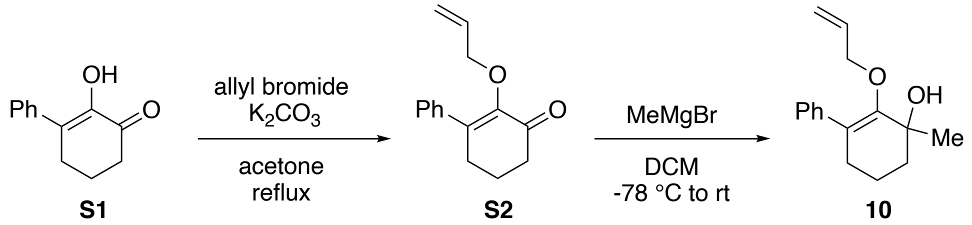

Procedure: Dione **S1** (2.90 g, 15.500 mmol) was dissolved in Na_2SO_4-dried acetone (70 mL, 0.2 M). Allylbromide (3.4 mL, 38.700 mmol) was added to the reaction mixture, followed by K_2CO_3 (9.00 g, 62.0 mmol). The solution was then warmed to reflux and stirred for 36 hours. Upon completion, the reaction mixture was cooled to 0 °C and quenched with DI water (30 mL). The aqueous layer was extracted with CH_2Cl_2 (3 x 30 mL) and the combined organic layers were washed with brine, dried over Na_2SO_4, and concentrated under vacuum. The crude product **S2** (1.54 g, 6.750 mmol) was taken onto the next step without further purification.

Ketone **S2** (1.54 g, 6.750 mmol) was dissolved in CH_2Cl_2 (33 mL, 0.2 M). After cooling the solution to -78 °C, Methylmagnesium bromide (3 mL, 8.770 mmol, 3.0 M in Et_2O) was then added dropwise. The reaction mixture was warmed to room temperature and stirred for 30 minutes. After cooling to 0 °C, the reaction was quenched with a saturated NH_4Cl solution (15 mL) and diluted with DI water (15 mL). The aqueous layer was extracted with CH_2Cl_2 (3 x 15 mL). The combined organic layers were washed with brine, dried over Na_2SO_4, and concentrated under vacuum. The crude material was purified by column chromatography using 100% hexanes to 90:10 hexanes : EtOAc to afford compound **10** in 81% yield (1.34 g, 5.484 mmol) as a yellow oil.

Rf: 0.5 in 80:20 (Hexanes : EtOAc)

^{1}H NMR: (500 MHz, $CDCl_3$) δ = 7.37 – 7.31 (m, 4H), 7.24 – 7.22 (m, 1H), 5.77 – 5.70 (m, 1H), 5.12 – 5.03 (m, 2H), 3.88 – 3.85 (m, 2H), 2.50 – 2.44 (m, 1H), 2.34 – 2.26 (m, 1H), 2.26 (s, 1H), 1.93 – 1.89 (m, 1H), 1.84 – 1.80 (m, 2H), 1.76 – 1.67 (m, 1H), 1.46 (s, 3H).

^{13}C NMR: (125 MHz, $CDCl_3$) δ = 153.6, 140.1, 134.5, 128.3, 128.1, 126.7, 121.2, 116.6, 74.2, 71.3, 38.1, 31.3, 27.4, 19.9.

IR: *f* (cm^{-1}) = 3446, 2970, 2934, 1706, 1645, 1444, 1386, 1267, 1130, 1040, 985.

HRMS: (ESI-TOF) *m/z*: $[(M-H_2O)+H)]^+$ = 227.1430 calculated for $C_{16}H_{19}O$; Found 227.1426.

3-(2-(allyloxy)-3-methyl-3,4,5,6-tetrahydro-[1,1'-biphenyl]-3-yl)-1*H*-indole (12)

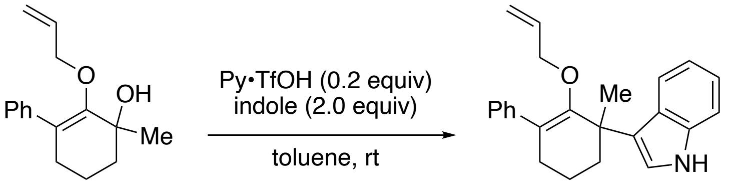

10 12

Procedure: α-Hydroxyl enol ether **10** (96 mg, 0.393 mmol) was dissolved in toluene (2.0 mL, 0.2 M). Indole (92 mg, 0.786 mmol) was added, followed by addition of Py•TfOH (18 mg, 0.079 mmol). The reaction mixture was stirred at room temperature for 24 hours, at which the starting material was fully consumed as monitored by TLC. The crude reaction mixture was concentrated under vacuum and then purified by column chromatography using 100% hexanes to 50:50 hexanes : CH_2Cl_2 to afford compound **12** in 93% yield (125 mg, 0.363 mmol) as an off-white solid.

Rf: 0.6 in 50:50 (Hexanes : CH_2Cl_2)

^{1}H NMR: (500 MHz, $CDCl_3$) δ = 7.91 (bs, 1H), 7.86 (d, *J* = 8.0 Hz, 1H), 7.45 (d, *J* = 6.9 Hz, 2H), 7.36 – 7.30 (m, 3H), 7.24 – 7.21 (m, 1H), 7.20 – 7.16 (m, 1H), 7.13 – 7.08 (m, 2H), 5.38 (ddt, *J* = 16.1, 10.7, 5.4 Hz, 1H), 4.79 – 4.77 (m, 1H), 4.77 – 4.74 (m, 1H), 3.66 – 3.63 (m, 1H), 3.59 – 3.57 (m, 1H), 2.59 (t, *J* = 6.2 Hz, 2H), 2.50 – 2.42 (m, 1H), 1.81 – 1.79 (m, 3H), 1.78 (s, 3H).

^{13}C NMR: (125 MHz, $CDCl_3$) δ = 156.9, 141.6, 137.0, 134.7, 128.5, 128.1, 126.2, 126.0, 123.6, 122.1, 121.5, 120.8, 120.0, 118.9, 115.4, 111.3, 73.8, 40.5, 38.8, 31.7, 25.1, 20.2.

IR: *f* (cm^{-1}) = 3415, 2930, 2864, 2833, 1693, 1642, 1491,1457, 1268, 1131, 990.

HRMS: (ESI-TOF) *m/z*: $(M+K)^+$ = 382.1567 calculated for $C_{24}H_{25}NOK$; Found 382.1566.

6-(allyloxy)-4'-methoxy-5-methyl-1,2,3,4-tetrahydro-[1,1'-biphenyl]-1-ol (15a)

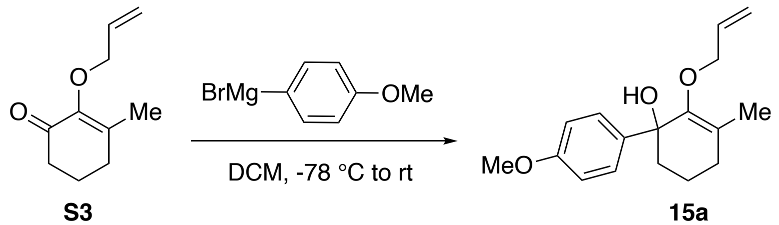

Procedure: Ketone **S3** (577 mg, 3.470 mmol) was dissolved in CH_2Cl_2 (17.0 mL, 0.2 M). After cooling the solution to -78 °C, 4-Methoxyphenylmagnesium bromide (5.2 mL, 5.21 mmol, 1.0 M in THF) was added dropwise. The reaction mixture was then warmed to room temperature and stirred for 1 hour. After cooling to 0 °C, the reaction was quenched with saturated NH_4Cl solution (8 mL) and diluted with DI water (8 mL). The aqueous layer was extracted with CH_2Cl_2 (3 x 10 mL) and the combined organic layers were washed with brine, dried over Na_2SO_4, and concentrated under vacuum. The crude material was purified by column chromatography using 100% hexanes to 90:10 hexanes : EtOAc to afford compound **15a** in 65% yield (621 mg, 2.264 mmol) as a colorless oil.

Rf: 0.4 in 80:20 (Hexanes : EtOAc)

^{1}H NMR: (400 MHz, $CDCl_3$) δ = 7.41 (d, *J* = 8.9 Hz, 2H), 6.86 (d, *J* = 8.9 Hz, 2H), 5.80 (ddt, *J* = 15.8, 10.5, 5.4 Hz, 1H), 5.15 (dq, *J* = 17.2, 1.7 Hz, 1H), 5.07 (dq, *J* = 10.5, 1.4 Hz, 1H), 4.16 (t, *J* = 1.5 Hz, 1H), 4.14 (t, *J* = 1.5 Hz, 1H), 3.80 (s, 3H), 2.70 (bs, 1H), 2.24 – 2.07 (m, 2H), 2.02 – 1.94 (m, 1H), 1.92 – 1.84 (m, 1H), 1.78 (s, 3H), 1.68 – 1.60 (m, 1H), 1.54 – 1.42 (m, 1H).

^{13}C NMR: (125 MHz, $CDCl_3$) δ = 158.4, 149.7, 138.8, 134.7, 127.3, 121.3, 116.3, 113.1, 75.4, 74.3, 55.2, 40.8, 31.0, 18.8, 16.8.

IR: *f* (cm^{-1}) = 3474. 2933, 2865, 2834, 1608, 1507, 1244, 1162, 1034.

HRMS: (ESI-TOF) *m/z*: $[(M-H_2O)+H)]^+$ = 257.1537 calculated for $C_{17}H_{21}O_2$; Found 257.1529.

6-(allyloxy)-3'-methoxy-5-methyl-1,2,3,4-tetrahydro-[1,1'-biphenyl]-1-ol (15b)

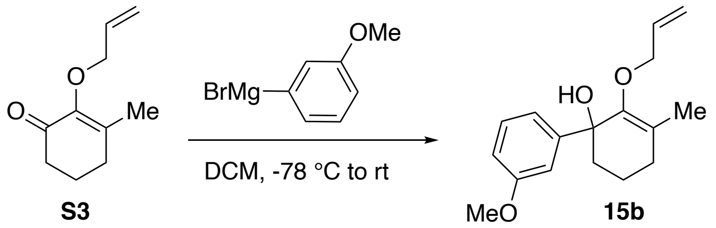

Procedure: Ketone **S3** (411 mg, 2.470 mmol) was dissolved in CH_2Cl_2 (12.0 mL, 0.2 M). After cooling the solution to -78 °C, 3-Methoxyphenylmagnesium bromide (3.2 mL, 3.20 mmol, 1.0 M in THF/toluene) was then added dropwise. The reaction mixture was then warmed to room temperature and stirred for 1 hour. After cooling to 0 °C, the reaction was quenched with saturated NH_4Cl solution (8 mL) and diluted with DI water (8 mL). The aqueous layer was extracted with CH_2Cl_2 (3 x 10 mL) and the combined organic layers were washed with brine, dried over Na_2SO_4, and concentrated under vacuum. The crude material was purified by column chromatography using 100% hexanes to 95:5 hexanes : EtOAc to afford compound **15b** in 66% yield (447 mg, 1.629 mmol) as a colorless oil.

Rf: 0.4 in 80:20 (Hexanes : EtOAc)

^{1}H NMR: (500 MHz, $CDCl_3$) δ = 7.24 (t, *J* = 7.9 Hz, 1H), 7.13 – 7.10 (m, 1H), 7.07 – 7.03 (m, 1H), 6.79 – 6.77 (m, 1H), 5.79 (ddt, *J* = 15,8, 10.6, 5.4 Hz, 1H), 5.15 (dq, *J* = 17.2, 1.7 Hz, 1H), 5.07 (dq, *J* = 10.5, 1.4 Hz, 1H), 4.15 (t, *J* = 1.5 Hz, 1H), 4.14 (t, *J* = 1.5 Hz, 1H), 3.82 (s, 3H), 2.76

(s, 1H), 2.22 – 2.10 (m, 2H), 2.02 – 1.96 (m, 1H), 1.93 – 1.87 (m, 1H), 1.78 (s, 3H), 1.68 – 1.62 (m, 1H), 1.56 – 1.52 (m, 1H).

^{13}C NMR: (125 MHz, $CDCl_3$) δ = 159.4, 149.6, 148.7, 134.7, 128.7, 121.5, 118.8, 116.4, 112.2, 111.9, 75.7, 74.4, 55.2, 40.6, 31.1, 18.8, 16.8.

IR: *f* (cm^{-1}) = 3484, 2935, 2866, 2833, 1671, 1598, 1582, 1483, 1315, 1157, 1083.

HRMS: (ESI-TOF) *m/z*: $[(M\text{-}H_2O)+H)]^+$ = 257.1537 calculated for $C_{17}H_{21}O_2$; Found 257.1529.

6-(allyloxy)-2'-methoxy-5-methyl-1,2,3,4-tetrahydro-[1,1'-biphenyl]-1-ol (15c)

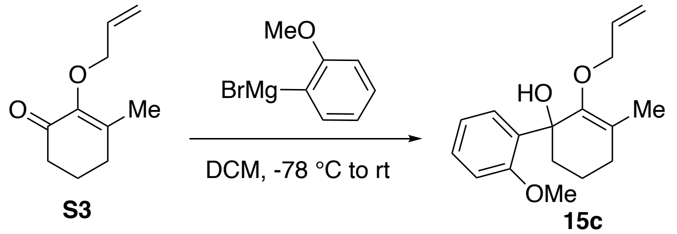

S3 **15c**

Procedure: Ketone **S3** (576 mg, 3.460 mmol) was dissolved in CH_2Cl_2 (17.0 mL, 0.2 M). After cooling the solution to -78 °C, 2-Methoxyphenylmagnesium bromide (5.2 mL, 5.180 mmol, 1.0 M in THF) was then added dropwise. The reaction mixture was then warmed to room temperature and stirred for 2 hours. After cooling to 0 °C, the reaction was quenched with a saturated NH_4Cl solution (8 mL) and diluted with DI water (8 mL). The aqueous layer was extracted with CH_2Cl_2 (3 x 10 mL) and the combined organic layers were washed with brine, dried over Na_2SO_4, and concentrated under vacuum. The crude material was purified by column chromatography using 100% hexanes to 95:5 hexanes : EtOAc to afford compound **15c** in 60% yield (568 mg, 2.070 mmol) as a colorless oil.

Rf: 0.4 in 80:20 (Hexanes : EtOAc)

^{1}H NMR: (500 MHz, $CDCl_3$) δ = 7.35 (dd, *J* = 7.7, 1.7 Hz, 1H), 7.22 (td, *J* = 8.1, 1.7 Hz, 1H), 6.94 (t, *J* = 7.5 Hz, 1H), 6.90 (d, *J* = 8.2 Hz, 1H), 5.85 (ddt, *J* = 15.9, 10.7, 5.5 Hz, 1H), 5.15 (dd,

J = 17.2, 1.8 Hz, 1H), 5.03 (dd, *J* = 10.5, 1.7 Hz, 1H), 4.38 – 4.33 (m, 1H), 4.18 – 4.12 (m, 2H), 3.87 (s, 3H), 2.16 – 2.06 (m, 3H), 2.03 – 1.96 (m, 1H), 1.78 (s, 3H), 1.76 – 1.68 (m, 1H), 1.46 – 1.38 (m, 1H).

^{13}C NMR: (125 MHz, $CDCl_3$) δ = 156.8, 148.9, 135.3, 133.3, 129.1, 127.9, 122.4, 120.6, 115.7, 111.3, 73.9, 55.4, 39.6, 31.1, 19.2, 16.9.

IR: *f* (cm^{-1}) = 3516, 2930, 2862, 2833, 1673, 1582, 1436, 1346, 1323, 1231, 1110, 1024.

HRMS: (ESI-TOF) *m/z*: $[(M-H_2O)+H)]^+$ = 257.1537 calculated for $C_{17}H_{21}O_2$; Found 257.1507.

2-(allyloxy)-1-(2,3-dihydrobenzo[*b*][1,4]dioxin-6-yl)-3-methylcyclohex-2-enol (15d)

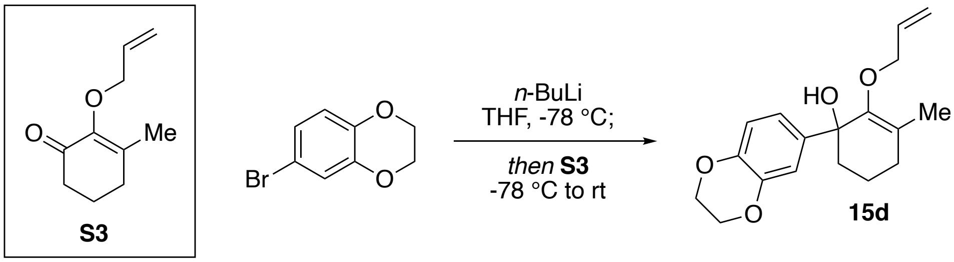

Procedure: 6-Bromo-2,3-dihydrobenzo[*b*][1,4]dioxine (0.60 mL, 4.250 mmol) was dissolved in THF (10 mL, 0.2 M). After cooling the solution to -78 °C, *n*-BuLi (1.7 mL, 2.5 M in hexanes, 4.250 mmol) was added dropwise. After stirring for 30 minutes, a solution of ketone **S3** (545 mg, 3.270 mmol) in THF (6.0 mL) was then added dropwise. The reaction mixture was then allowed to warm to room temperature and stirred for 2 hours. Upon consumption of **S3** as monitored by TLC, the reaction mixture was cooled to 0 °C, quenched with a saturated NH_4Cl solution (10 mL), and then diluted with water (10 mL). The aqueous layer was extracted with CH_2Cl_2 (3 x 10 mL). The combined organic layers were then washed with brine, dried over Na_2SO_4, and concentrated under vacuum. The crude material was purified with column chromatography using 100% hexanes

to 80:20 hexanes : EtOAc to afford compound **15d** in 39% yield (384 mg, 1.270 mmol) as a colorless oil.

Rf: 0.6 in 80:20 (Hexanes : EtOAc)

^{1}H NMR: (500 MHz, $CDCl_3$) δ = 7.02 (d, *J* = 2.1 Hz, 1H), 6.96 (dt, *J* = 8.4, 1.7 Hz, 1H), 6.80 (dd, *J* = 8.4, 1.7 Hz, 1H), 5.86 – 5.78 (m, 1H), 5.17 (dt, *J* = 17.2, 1.6 Hz, 1H), 5.08 (dt, *J* = 10.5, 1.5 Hz, 1H), 4.26 – 4.23 (m, 4H), 4.16 – 4.15 (m, 2H), 2.66 (d, *J* = 6.6 Hz, 1H), 2.20 – 2.06 (m, 2H), 1.99 – 1.93 (m, 1H), 1.89 – 1.84 (m, 1H), 1.76 (s, 3H), 1.67 – 1.59 (m, 1H), 1.56 – 1.46 (m, 1H).

^{13}C NMR: (125 MHz, $CDCl_3$) δ = 149.8, 142.9, 142.4, 140.4, 134.8, 121.2, 119.3, 116.5, 116.4, 115.3, 75.4, 74.4, 64.4, 64.4, 40.8, 31.1, 18.9, 16.8.

IR: *f* (cm^{-1}) = 3510, 2978, 2932, 2869, 1588, 1501, 1421, 1307, 1284, 1160, 1068, 990.

HRMS: (ESI-TOF) *m/z*: $[(M-H_2O)+H]^+$ = 285.1486 calculated for $C_{18}H_{21}O_3$; Found 285.1485.

6-(allyloxy)-5-methyl-4'-(methylthio)-1,2,3,4-tetrahydro-[1,1'-biphenyl]-1-ol (15e)

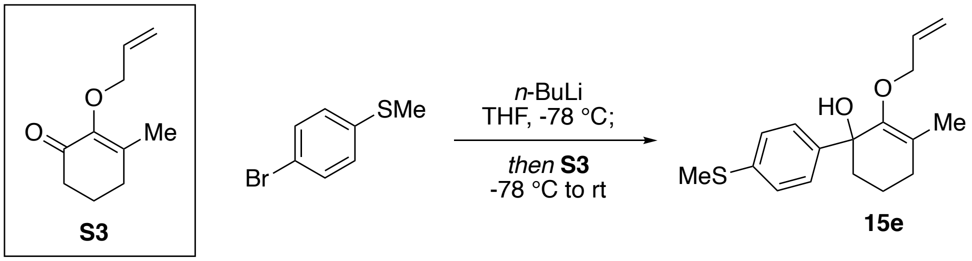

Procedure: 4-Bromophenyl methylsulfane (637 mg, 3.141 mmol) was dissolved in THF (12 mL). After cooling the solution to -78 °C, *n*-BuLi (1.3 mL, 2.5 M in hexanes, 3.140 mmol) was added dropwise. After stirring for 30 minutes, a solution of ketone **S3** (476 mg, 2.860 mmol) in THF (4.0 mL) was added dropwise. The reaction mixture was then allowed to warm to room temperature and stirred for 1 hour. Upon consumption of **S3** as monitored by TLC, the reaction mixture was cooled to 0 °C, quenched with a saturated NH_4Cl solution (15 mL), and then diluted with water

(10 mL). The aqueous layer was extracted with CH_2Cl_2 (3 x 10 mL). The combined organic layers were then washed with brine, dried over Na_2SO_4, and concentrated under vacuum. The crude material was purified with column chromatography using 100% hexanes to 90:10 hexanes : EtOAc to afford compound **15e** in 52% yield (435 mg, 1.485 mmol) as a colorless oil.

Rf: 0.4 in 80:20 (Hexanes : EtOAc)

^{1}H NMR: (500 MHz, $CDCl_3$) δ = 7.42 (d, *J* = 8.5 Hz, 2H), 7.22 (d, *J* = 8.3 Hz, 2H), 5.79 (ddt, *J* = 15.9, 10.4, 5.3 Hz, 1H), 5.14 (dd, *J* = 17.4, 1.6 Hz, 1H), 5.06 (d, *J* = 10.5 Hz, 1H), 4.15 (t, *J* = 1.3 Hz, 1H), 4.14 (t, *J* = 1.3 Hz, 1H), 2.71 (bs, 1H), 2.47 (s, 3H), 2.21 – 2.09 (m, 2H), 2.02 – 1.95 (m, 1H), 1.90 – 1.84 (m, 1H), 1.78 (s, 3H), 1.69 – 1.61 (m, 1H), 1.56 – 1.47 (m, 1H).

^{13}C NMR: (125 MHz, $CDCl_3$) δ = 149.6, 143.9, 136.6, 134.6, 126.7, 126.3, 121.5, 116.4, 75.5, 74.2, 40.9, 31.1, 18.8, 16.8, 16.0.

IR: *f* (cm^{-1}) = 3477, 2982, 2921, 2863, 2831, 1671, 1595, 1489, 1438, 1396, 1245, 1160, 1040, 922.

HRMS: (ESI-TOF) *m/z*: $[(M-H_2O)+H]^+$ = 273.1308 calculated for $C_{17}H_{21}OS$; Found 273.1306.

6-(allyloxy)-4'-chloro-5-methyl-1,2,3,4-tetrahydro-[1,1'-biphenyl]-1-ol (15g)

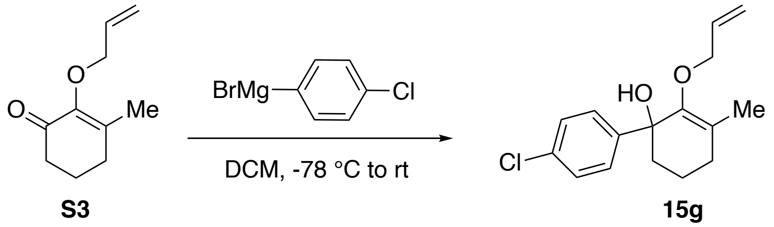

S3 15g

Procedure: Ketone **S3** (586 mg, 3.530 mmol) was dissolved in CH_2Cl_2 (17.0 mL, 0.2 M). After cooling the solution to -78 °C, 4-Chlorophenyl magnesium bromide (5.3 mL, 1.0 M in Et_2O, 5.290 mmol) was added dropwise. The reaction mixture was then warmed to room temperature and stirred for 1 hour. Upon consumption of **S3** as monitored by TLC, the reaction mixture was cooled

to 0 °C, quenched with a saturated NH_4Cl solution (15 mL), and then diluted with water (10 mL). The aqueous layer was extracted with CH_2Cl_2 (3 x 10 mL). The combined organic layers were then washed with brine, dried over Na_2SO_4, and concentrated under vacuum. The crude material was purified by column chromatography using 100% hexanes to 90:10 hexanes : EtOAc to afford compound **15g** in 75% yield (739 mg, 2.650 mmol) as a colorless oil.

Rf: 0.3 in 80:20 (Hexanes : EtOAc)

^{1}H NMR: (500 MHz, $CDCl_3$) δ = 7.46 – 7.41 (m, 2H), 7.31 – 7.26 (m, 2H), 5.84 – 5.73 (m, 1H), 5.18 – 5.06 (m, 2H), 4.14 (dq, *J* = 5.5, 1.5 Hz, 2H), 2.71 (s, 1H), 2.24 – 2.08 (m, 2H), 2.03 – 1.94 (m, 1H), 1.87 – 1.81 (m, 1H), 1.78 (s, 3H), 1.70 – 1.62 (m, 1H), 1.54 – 1.44 (m, 1H).

^{13}C NMR: (125 MHz, $CDCl_3$) δ = 149.3, 145.3, 134.5, 132.6, 127.9, 127.6, 122.0, 116.6, 75.4, 74.3, 40.8, 31.0, 18.7, 16.8.

IR: *f* (cm^{-1}) = 3549, 2936, 2866, 2833, 1671, 1574, 1487, 1398, 1329, 1161, 1087, 1041, 1012.

HRMS: (ESI-TOF) *m/z*: $[(M-H_2O)+H)]^+$ = 261.1046 calculated for $C_{16}H_{18}OCl$; Found 261.1041.

6-(allyloxy)-3'-fluoro-4'-methoxy-5-methyl-1,2,3,4-tetrahydro-[1,1'-biphenyl]-1-ol (15h)

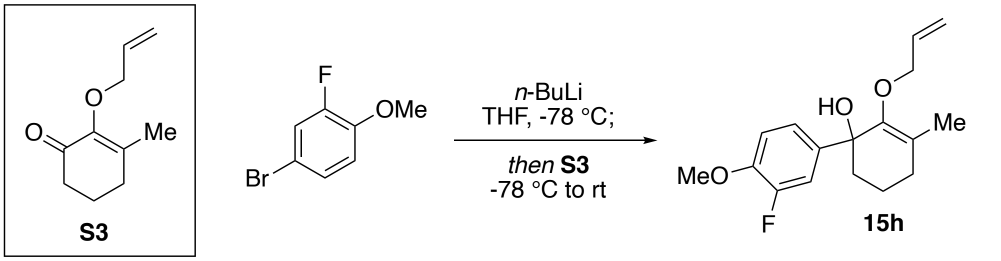

Procedure: 4-Bromo-2-fluoro-1-methoxybenzene (0.51 mL, 3.040 mmol) was dissolved in THF (15 mL). After cooling the solution to -78 °C, *n*-BuLi (1.6 mL, 2.5 M in hexanes, 3.040 mmol) was added dropwise. After stirring for 30 minutes, a solution of ketone **S3** (506 mg, 2.860 mmol) in THF (4.0 mL) was added dropwise. The reaction mixture was allowed to warm to room

temperature and stirred for 30 minutes. Upon consumption of **S3** as monitored by TLC, the reaction mixture was cooled to 0 °C, quenched with a saturated NH_4Cl solution (10 mL), and then diluted with water (10 mL). The aqueous layer was extracted with CH_2Cl_2 (3 x 10 mL). The combined organic layers were then washed with brine, dried over Na_2SO_4, and concentrated under vacuum. The crude material was purified with column chromatography using 100% hexanes to 80:20 hexanes : EtOAc to afford compound **15h** in 68% yield (602 mg, 1.941 mmol) as a yellow oil.

Rf: 0.6 in 80:20 (Hexanes : EtOAc)

^{1}H NMR: (500 MHz, $CDCl_3$) δ = 7.24 (dd, *J* = 12.8, 2.3 Hz, 1H), 7.20 – 7.16 (m, 1H), 6.90 (t, *J* = 8.6 Hz, 1H), 5.85 – 5.76 (m, 1H), 5.15 (dq, *J* = 17.2, 1.8 Hz, 1H), 5.09 (dq, *J* = 10.4, 1.4 Hz, 1H), 4.15 (dq, *J* = 5.5, 1.6 Hz, 2H), 3.88 (s, 3H), 2.70 (s, 1H), 2.22 – 2.09 (m, 2H), 2.01 – 1.94 (m, 1H), 1.88 – 1.82 (m, 1H), 1.77 (s, 3H), 1.69 – 1.62 (m, 1H), 1.53 – 1.46 (m, 1H).

^{13}C NMR: (125 MHz, $CDCl_3$) δ = 152.9, 150.9, 149.4, 146.4, 146.3, 140.2, 140.2, 134.6, 121.8, 121.7, 116.6, 114.4, 114.2, 112.7, 112.7, 75.2, 74.4, 56.3, 40.7, 31.0, 18.7, 16.8.

IR: *f* (cm^{-1}) = 3471, 2935, 2866, 1837, 1620, 1584, 1509, 1307, 1271, 1159, 1087, 1027.

HRMS: (ESI-TOF) *m/z*: $[(M-H_2O)+H]^+$ = 275.1442 calculated for $C_{17}H_{20}O_2F$; Found 275.1454.

6-(allyloxy)-5-methyl-4'-(trifluoromethyl)-1,2,3,4-tetrahydro-[1,1'-biphenyl]-1-ol (15i)

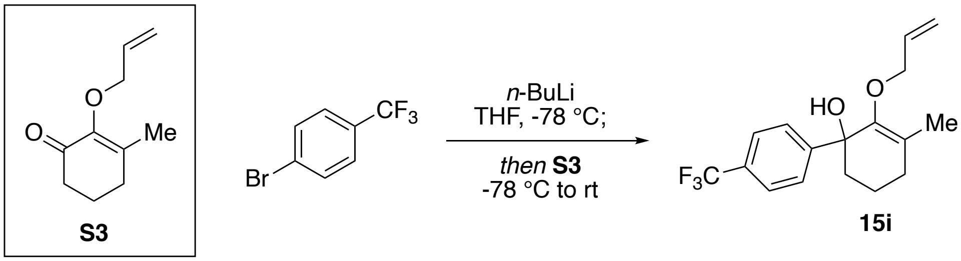

Procedure: 1-Bromo-4-(trifluoromethyl)benzene (0.6 mL, 4.080 mmol) was dissolved in THF (11 mL). After cooling the solution to -78 °C, *n*-BuLi (1.7 mL, 2.5 M in hexanes, 4.080 mmol)

was added dropwise. After stirring for 45 minutes, a solution of ketone **S3** (523 mg, 2.860 mmol) in THF (4.0 mL) was then added dropwise. The reaction mixture was allowed to warm to room temperature and stirred for 15 minutes. Upon consumption of **S3** as monitored by TLC, the reaction mixture was cooled to 0 °C, quenched with a saturated NH_4Cl solution (10 mL), and then diluted with water (10 mL). The aqueous layer was extracted with CH_2Cl_2 (3 x 10 mL). The combined organic layers were then washed with brine, dried over Na_2SO_4, and concentrated under vacuum. The crude material was purified with column chromatography using 100% hexanes to 90:10 hexanes : EtOAc to afford compound **15i** in 73% yield (716 mg, 2.292 mmol) as a yellow oil.

Rf: 0.5 in 80:20 (Hexanes : EtOAc)

^{1}H NMR: (500 MHz, $CDCl_3$) δ = 7.62 (d, *J* = 8.4 Hz, 2H), 7.58 (d, *J* = 8.5 Hz, 2H), 5.78 (ddt, *J* = 15.9, 10.6, 5.4 Hz, 1H), 5.12 (dq, *J* = 17.2, 1.6 Hz, 1H), 5.08 (dq, *J* = 10.5, 1.4 Hz, 1H), 4.15 (s, 1H), 4.14 (s, 1H), 2.71 (s, 1H), 2.24 – 2.13 (m, 2H), 2.02 (ddd, *J* = 12.9, 9.4, 3.2 Hz, 1H), 1.90 – 1.84 (m, 1H), 1.80 (s, 3H), 1.73 – 1.65 (m, 1H), 1.58 – 1.49 (m, 1H).

^{13}C NMR: (125 MHz, $CDCl_3$) δ = 151.0, 149.3, 134.5, 126.5, 124.8, 124.8, 122.3, 116.7, 75.7, 74.3, 41.0, 31.1, 18.7, 16.9.

IR: *f* (cm^{-1}) = 3458, 2937, 2868, 2836, 1617, 1409, 1322, 1160, 1119, 1087, 1016.

HRMS: (ESI-TOF) *m/z*: $[(M-H_2O)+H]^+$ = 295.1305 calculated for $C_{17}H_{18}OF_3$; Found 295.1313.

2-(allyloxy)-3-methyl-1-(9-methyl-9*H*-carbazol-3-yl)cyclohex-2-enol (15j)

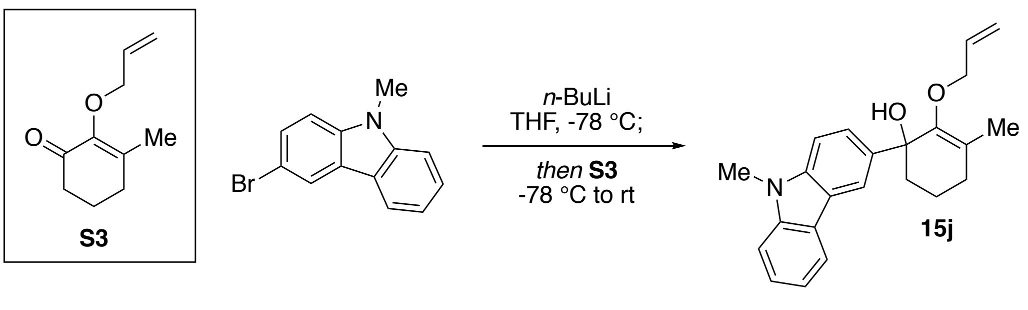

Procedure: 3-Bromo-9-methyl-9*H*-carbazole (851 mg, 3.280 mmol) was dissolved in THF (10 mL, 0.2 M). After cooling to -78 °C, *n*-BuLi (1.3 mL, 2.5 M in hexanes, 3.280 mmol) was added dropwise. After stirring for 30 minutes, a solution of ketone **S3** (420 mg, 2.519 mmol) in THF (4.0 mL) was added dropwise. The reaction mixture was then allowed to warm to room temperature and stirred for 1 hour. Upon consumption of **S3** as monitored by TLC, the reaction mixture was cooled to 0 °C, quenched with a saturated NH_4Cl solution (15 mL), and then diluted with water (10 mL). The aqueous layer was extracted with CH_2Cl_2 (3 x 10 mL). The combined organic layers were then washed with brine, dried over Na_2SO_4, and concentrated under vacuum. The crude material was purified with column chromatography using 100% hexanes to 80:20 hexanes : EtOAc to afford compound **15j** in 63% yield (549 mg, 1.580 mmol) as a colorless oil.

Rf: 0.7 in 80:20 (Hexanes : EtOAc)

^{1}H NMR: (500 MHz, $CDCl_3$) δ = 8.27 (d, *J* = 1.6 Hz, 1H), 8.13 (d, *J* = 7.7 Hz, 1H), 7.62 (dd, *J* = 8.5, 1.7 Hz, 1H), 7.47 (t, *J* = 7.6 Hz, 1H), 7.39 (d, *J* = 8.1 Hz, 1H), 7.35 (d, *J* = 8.5 Hz, 1H), 7.23 (d, *J* = 7.4 Hz, 1H), 5.81 – 5.73 (m, 1H), 5.12 (dd, *J* = 17.2, 1.6 Hz, 1H), 5.04 – 5.00 (m, 1H), 4.18 (d, *J* = 6.5 Hz, 2H), 3.85 (s, 3H), 2.90 (s, 1H), 2.23 (q, *J* = 5.8 Hz, 2H), 2.14 – 2.07 (m, 1H), 2.07 – 2.01 (m, 1H), 1.86 (s, 3H), 1.73 – 1.64 (m, 1H), 1.64 – 1.54 (m, 1H).

^{13}C NMR: (125 MHz, $CDCl_3$) δ = 150.1, 141.3, 140.2, 137.5, 134.7, 125.5, 124.4, 122.9, 122.3, 121.3, 120.3, 118.7, 117.9, 116.3, 108.4, 107.6, 76.1, 74.3, 41.4, 31.2, 29.1, 18.9, 16.9.

IR: *f* (cm^{-1}) = 3472, 2932, 2866, 2830, 1672, 1601, 1482, 1328, 1247, 1154, 990.

HRMS: (ESI-TOF) *m/z*: $[(M\text{-}H_2O)+H)]^+$ = 330.1853 calculated for $C_{23}H_{24}NO$; Found 330.1867.

2-(allyloxy)-1-(benzo[*b*]thiophen-2-yl)-3-methylcyclohex-2-enol (15k)

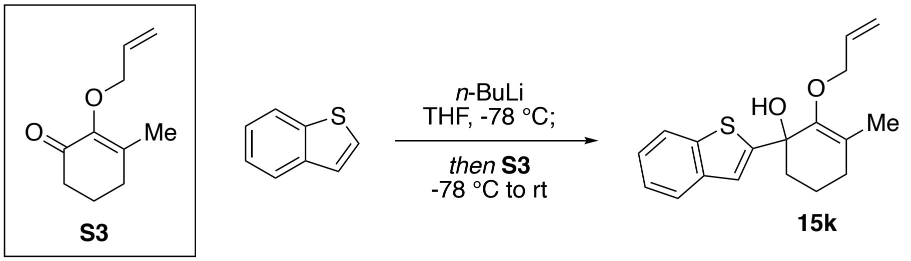

15k

Procedure: Benzothiophene (396 mg, 2.950 mmol) was dissolved in THF (15 mL, 0.2 M). After cooling the solution to -78 °C, *n*-BuLi (1.2 mL, 2.5 M in THF, 2.950 mmol) was added dropwise. After stirring for 1 hour, a solution of ketone **S3** (410 mg, 2.46 mmol) in THF (4.0 mL) was added dropwise. The reaction mixture was then allowed to warm to room temperature and stirred for 30 minutes. Upon consumption of **S3** as monitored by TLC, the reaction mixture was cooled to 0 °C, quenched with a saturated NH_4Cl solution (10 mL), and then diluted with water (10 mL). The aqueous layer was extracted with CH_2Cl_2 (3 x 10 mL). The combined organic layers were then washed with brine, dried over Na_2SO_4, and concentrated under vacuum. The crude material was purified with column chromatography using 100% hexanes to 95:5 hexanes : EtOAc to afford compound **15k** in 76% yield (562 mg, 1.871 mmol) as an orange oil.

Rf: 0.4 in 80:20 (Hexanes : EtOAc)

^{1}H NMR: (500 MHz, $CDCl_3$) δ = 7.82 – 7.78 (m, 1H), 7.71 – 7.67 (m, 1H), 7.35 – 7.27 (m, 2H), 7.17 (s, 1H), 5.89 – 5.80 (m, 1H), 5.21 (dq, *J* = 17.2, 1.7 Hz, 1H), 5.10 (dq, *J* = 10.5, 1.5 Hz, 1H), 4.24 (dq, *J* = 5.4, 1.4 Hz, 2H), 3.33 (s, 1H), 2.26 – 2.19 (m, 1H), 2.18 – 2.05 (m, 3H), 1.80 (s, 3H), 1.75 – 1.67 (m, 2H).

^{13}C NMR: (125 MHz, $CDCl_3$) δ = 153.3, 149.1, 139.7, 139.6, 134.4, 124.0, 123.8, 123.3, 122.3, 121.8, 120.7, 116.7, 75.0, 74.4, 39.7, 30.8, 18.8, 16.8.

IR: *f* (cm^{-1}) = 3434, 2911, 2865, 1673, 1456, 1434, 1331, 1246, 1156, 1039, 978.

HRMS: (ESI-TOF) *m/z*: $[(M-H_2O)+H)]^+$ = 283.1152 calculated for $C_{18}H_{19}OS$; Found 283.1157.

2-(allyloxy)-3-methyl-1-(3-methylthiophen-2-yl)cyclohex-2-enol (15l)

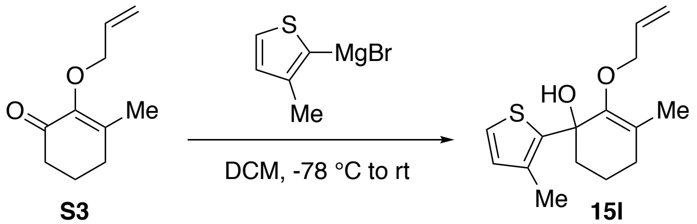

S3 **15l**

Procedure: Ketone **S3** (811 mg, 4.879 mmol) was dissolved in CH_2Cl_2 (24.0 mL, 0.2 M). After cooling the solution to -78 °C, 3-Methyl-2-thienylmagnesium bromide (14.6 mL, 0.5 M in THF, 7.319 mmol) was added dropwise. The solution was then warmed to room temperature and stirred for 2 hours. Upon consumption of **S3** as monitored by TLC, the reaction mixture was cooled to 0 °C, quenched with a saturated NH_4Cl solution (15 mL), and then diluted with water (10 mL). The aqueous layer was extracted with CH_2Cl_2 (3 x 10 mL). The combined organic layers were then washed with brine, dried over Na_2SO_4, and concentrated under vacuum. The crude material was purified by column chromatography using 100% hexanes to 70:30 hexanes : CH_2Cl_2 to afford compound **15l** in 61% yield (797 mg, 3.015 mmol) as a yellow oil.

Rf: 0.4 in 80:20 (Hexanes : EtOAc)

^{1}H NMR: (500 MHz, $CDCl_3$) δ = 7.04 (d, *J* = 5.1 Hz, 1H), 6.78 (d, *J* = 5.1 Hz, 1H), 5.88 – 5.79 (m, 1H), 5.20 (d, *J* = 17.2 Hz, 1H), 5.11 (d, *J* = 10.4, 1.7 Hz, 1H), 4.25 – 4.17 (m, 2H), 2.94 (s, 1H), 2.20 (s, 3H), 2.15 – 2.13 (m, 2H), 2.11 – 1.98 (m, 2H), 1.75 (s, 3H), 1.74 – 1.68 (m, 1H), 1.66 – 1.59 (m, 1H).

^{13}C NMR: (125 MHz, $CDCl_3$) δ = 149.3, 144.6, 134.8, 131.7, 131.4, 122.0, 121.5, 116.6, 74.3, 74.2, 39.2, 31.1, 19.1, 16.9, 14.6.

IR: *f* (cm^{-1}) = 3551, 2929, 2864, 1671, 1450, 1327, 1159, 1083, 1037, 923.

HRMS: (ESI-TOF) *m/z*:[(M-H_2O)+H)]$^+$ = 247.1151 calculated for $C_{15}H_{19}OS$; Found 247.1150.

5-methyl-6-((2-methylallyl)oxy)-1,2,3,4-tetrahydro-[1,1'-biphenyl]-1-ol (17a)

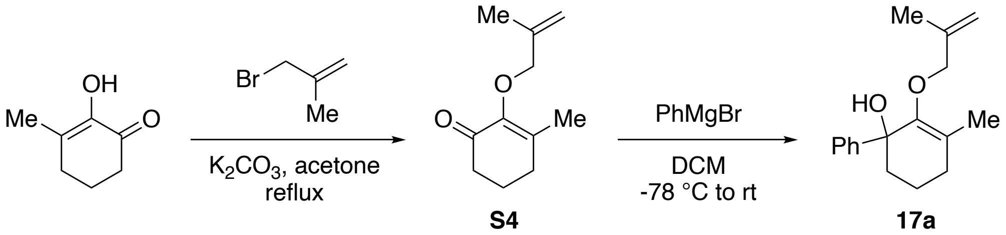

Procedure: 2-Hydroxy-3-methylcyclohex-2-en-1-one (756 mg, 5.980 mmol) was dissolved in Na_2SO_4-dried acetone (20 mL). 3-Bromo-2-methylprop-1-ene (1.5 mL, 14.900 mmol) was added to the reaction mixture, followed by K_2CO_3 (3.30 g, 23.900 mmol). The solution was then warmed to reflux and stirred for 30 hours. Upon completion, the reaction mixture was cooled to 0 °C and then quenched with DI water (10 mL). The aqueous layer was extracted with CH_2Cl_2 (3 x 15 mL). The combined organic layers were then washed with brine, dried over Na_2SO_4, and concentrated under vacuum. The crude material was purified with column chromatography using 100% hexanes to 95:5 hexanes : EtOAc to afford compound **S4** in 64% yield (688 mg, 3.820 mmol) as a colorless oil.

Rf: 0.7 in 80:20 (Hexanes : EtOAc)

^{1}H NMR: (500 MHz, $CDCl_3$) δ = 5.02 (s, 1H), 4.90 (s, 1H), 4.22 (s, 2H), 2.44 (t, *J* = 6.5 Hz, 2H), 2.38 (t, *J* = 5.9 Hz, 2H), 1.95 – 1.91 (m, 5H), 1.82 (s, 3H).

^{13}C NMR: (125 MHz, $CDCl_3$) δ = 194.7, 148.1, 145.9, 141.9, 112.7, 75.6, 38.8, 31.5, 22.2, 19.7, 17.8.

IR: *f* (cm^{-1}) = 3434, 2941, 2875, 1729, 1672, 1377, 1193, 1153, 1046, 992.

HRMS: (ESI-TOF) *m/z*: (M+H)$^+$ = 118.1223 calculated for $C_{11}H_{17}O_2$; Found 118.1213.

Ketone **S4** (600 mg, 3.330 mmol) was dissolved in CH_2Cl_2 (17 mL). After cooling the solution to -78 °C, phenylmagnesium bromide (1.5 mL, 3.0 M in Et_2O, 4.330 mmol) was added drop wise. The reaction mixture was then stirred at room temperature for 3 hours. Upon consumption of **S4** as monitored by TLC, the reaction mixture was cooled to 0 °C, quenched with a saturated NH_4Cl solution (12 mL), and then diluted with water (10 mL). The aqueous layer was extracted with CH_2Cl_2 (3 x 10 mL). The combined organic layers were then washed with brine, dried over Na_2SO_4, and concentrated under vacuum. The crude material was purified with column chromatography using 100% hexanes to 97:3 hexanes : EtOAc to afford compound **17a** in 73% yield (631 mg, 2.440 mmol) as a colorless oil.

Rf: 0.7 in 80:20 (Hexanes : EtOAc)

^{1}H NMR: (500 MHz, $CDCl_3$) δ = 7.51 (d, *J* = 8.3 Hz, 2H), 7.33 (t, *J* = 7.4 Hz, 2H), 7.24 (t, *J* = 7.3 Hz, 1H), 4.82 (s, 1H), 4.77 (s, 1H), 4.05 (q, *J* = 12.7 Hz, 2H), 2.78 (s, 1H), 2.20 – 2.14 (m, 2H), 2.01 (ddd, *J* = 13.0, 9.9, 3.2 Hz, 1H), 1.92 – 1.87 (m, 1H), 1.80 (s, 3H), 1.72 – 1.63 (m, 1H), 1.57 (s, 3H), 1.56 – 1.48 (m, 1H).

^{13}C NMR: (125 MHz, $CDCl_3$) δ = 149.8, 146.8, 142.5, 127.8, 126.7, 126.1, 121.6, 111.2, 76.8, 75.8, 40.9, 31.1, 19.3, 18.8, 16.7.

IR: *f* (cm^{-1}) = 3466, 2934, 2963, 1656, 1446, 1158, 1090, 992, 899.

HRMS: (ESI-TOF) *m/z*: $(M+H)^+$ = 259.1693 calculated for $C_{17}H_{23}O_2$; Found 259.1682.

5-methyl-6-((2-methyleneoctyl)oxy)-1,2,3,4-tetrahydro-[1,1'-biphenyl]-1-ol (17b)

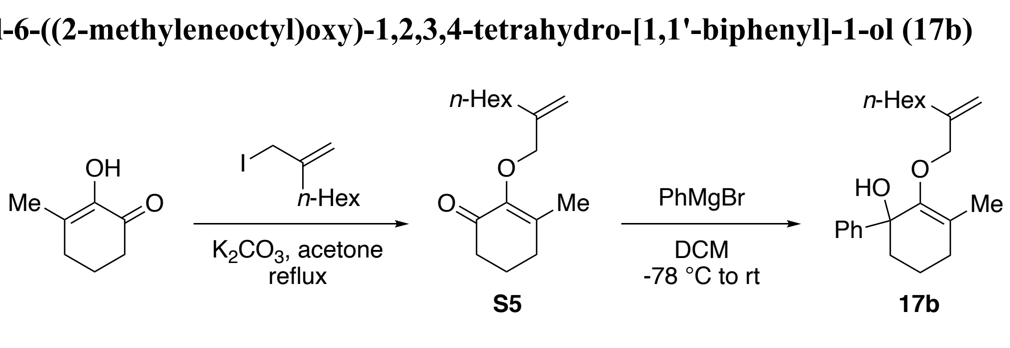

Procedure: 2-Hydroxy-3-methylcyclohex-2-en-1-one (286 mg, 2.273 mmol) was dissolved in acetone (12 mL). 2-(Iodomethyl)oct-1-ene (860 mL, 3.410 mmol) was added to the reaction mixture, followed by K_2CO_3 (942 mg, 6.820 mmol). The solution was then warmed to reflux and stirred for 30 hours. Upon completion, the reaction mixture was cooled to 0 °C and then quenched with DI water (10 mL). The aqueous layer was extracted with CH_2Cl_2 (3 x 15 mL) and the combined organic layers were washed with brine, dried over Na_2SO_4, and concentrated under vacuum. The crude material was purified with column chromatography using 100% hexanes to 70:30 hexanes : CH_2Cl_2 to afford compound **S5** in 70% yield (403 mg, 1.610 mmol) as a yellow oil. This compound was used immediately in the next step.

Ketone **S5** (303 mg, 1.210 mmol) was dissolved in CH_2Cl_2 (8 mL). After cooling the solution to -78 °C, phenylmagnesium bromide (0.7 mL, 3.0 M in Et_2O, 2.090 mmol) was added dropwise. The reaction mixture was then stirred at room temperature for 2 hours. Upon consumption of **S5** as monitored by TLC, the reaction mixture was cooled to 0 °C, quenched with a saturated NH_4Cl solution (10 mL), and then diluted with water (10 mL). The aqueous layer was extracted with CH_2Cl_2 (3 x 10 mL). The combined organic layers were then washed with brine, dried over Na_2SO_4, and concentrated under vacuum. The crude material was purified with column chromatography using 100% hexanes to 98:2 hexanes : EtOAc to afford compound **17b** in 61% yield (242 mg, 0.736 mmol) as a yellow oil.

Rf: 0.3 in 80:20 (Hexanes : EtOAc)

^{1}H NMR: (400 MHz, $CDCl_3$) δ = 7.51 – 7.47 (m, 2H), 7.32 (t, J = 7.5 Hz, 2H), 7.25 – 7.20 (m, 1H), 4.84 (s, 1H), 4.76 (s, 1H), 4.06 (q, J = 12.8 Hz, 2H), 2.77 (s, 1H), 2.16 (q, J = 5.7 Hz, 2H),

2.00 (ddd, J = 13.0, 9.8, 3.3 Hz, 1H), 1.92 – 1.81 (m, 3H), 1.79 (s, 3H), 1.71 – 1.61 (m, 1H), 1.55 – 1.48 (m, 1H), 1.31 – 1.14 (m, 8H), 0.86 (t, J = 7.1 Hz, 3H).

^{13}C NMR: (125 MHz, $CDCl_3$) δ = 149.9, 146.9, 146.9, 127.8, 126.8, 126.1, 121.6, 110.3, 76.0, 75.8, 40.9, 33.0, 31.7, 31.2, 29.0, 27.5, 22.6, 18.9, 16.8, 14.1.

IR: f (cm^{-1}) = 3531, 2926, 2856, 1650, 1490, 1447, 1330, 1159, 1090, 988, 900.

HRMS: (ESI-TOF) *m/z*: $[(M\text{-}H_2O)+H]^+$ = 311.2370 calculated for $C_{22}H_{31}O$; Found 311.2336.

6-((2-benzylallyl)oxy)-5-methyl-3,4-dihydro-[1,1'-biphenyl]-1(2*H*)-ol (17c)

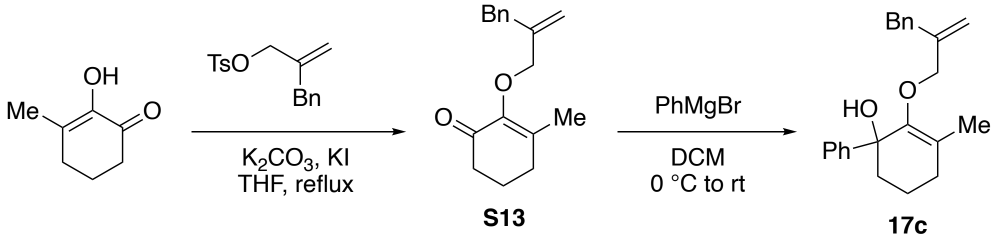

Procedure: 2-Hydroxy-3-methylcyclohex-2-en-1-one (594 mg, 4.708 mmol) was dissolved in THF (12 mL). 2-benzylallyl 4-methylbenzenesulfonate (1.42 g, 4.708 mmol) was added to the reaction mixture, followed by K_2CO_3 (1.30 g, 9.417 mmol) and KI (781 mg, 4.708 mmol). The solution was then warmed to reflux and stirred for 30 hours. Upon completion, the reaction mixture was cooled to 0 °C and then quenched with DI water (15 mL). The aqueous layer was extracted with EtOAc (3 x 15 mL) and the combined organic layers were washed with brine, dried over Na_2SO_4, and concentrated under vacuum. The crude material was purified with column chromatography using 100% hexanes to 99:1 hexanes : EtOAc to afford compound **S13** in 62% yield (741 mg, 2.890 mmol) as a colorless oil. This compound was used immediately in the next step.

Ketone **S13** (156 mg, 0.609 mmol) was dissolved in CH_2Cl_2 (3.0 mL, 0.2 M). After cooling the solution to 0 °C, phenylmagnesium bromide (0.3 mL, 3.0 M in Et_2O, 0.914 mmol) was added dropwise. The reaction mixture was then stirred for 90 minutes at 0 °C. Upon consumption of **S13** as monitored by TLC, the reaction mixture was quenched with a saturated NH_4Cl solution (5 mL), and then diluted with water (10 mL). The aqueous layer was extracted with CH_2Cl_2 (3 x 10 mL). The combined organic layers were then washed with brine, dried over Na_2SO_4, and concentrated under vacuum. The crude material was purified by column chromatography using 100% hexanes to 97:3 hexanes : EtOAc to afford compound **17c** in 74% yield (151 mg, 0.451 mmol) as a colorless oil.

Rf: 0.3 in 80:20 (Hexanes : EtOAc)

^{1}H NMR (500 MHz, $CDCl_3$) δ = 7.49 – 7.46 (m, 2H), 7.36 – 7.31 (m, 2H), 7.27 – 7.25 (m overlapped, 1H), 7.25 – 7.21 (m, 2H), 7.21 – 7.17 (m, 1H), 7.04 – 7.00 (m, 2H), 4.98 (s, 1H), 4.81 (s, 1H), 4.06 (s, 2H), 3.23 (s, 2H), 2.56 (s, 1H), 2.20 – 2.08 (m, 2H), 2.01 – 1.96 (m, 1H), 1.89 – 1.84 (m, 1H), 1.75 (s, 3H), 1.68 – 1.60 (m, 1H), 1.55 – 1.45 (m, 1H).

^{13}C NMR (125 MHz, $CDCl_3$) δ = 149.4, 146.7, 145.6, 138.7, 128.8, 128.3, 127.8, 126.8, 126.2, 126.1, 121.9, 112.9, 75.8, 75.2, 40.7, 39.8, 31.0, 18.7, 16.7.

IR: *f* (cm^{-1}) = 3548, 2933, 1448, 1157.

HRMS: (ESI-TOF) *m/z*: $[(M-H_2O)+H]^+$ = 317.1899 calculated for $C_{23}H_{25}O$; Found 317.1896.

6-((2-bromoallyl)oxy)-5-methyl-3,4-dihydro-[1,1'-biphenyl]-1(2*H*)-ol (17d)

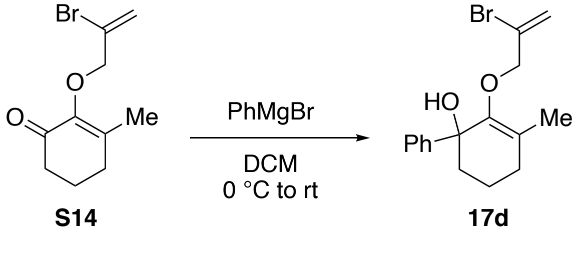

Ketone **S14** (500 mg, 2.030 mmol) was dissolved in CH_2Cl_2 (10 mL). After cooling the solution to 0 °C, phenylmagnesium bromide (0.9 mL, 3.0 M in Et_2O, 2.650 mmol) was added dropwise. The reaction mixture was then stirred at room temperature for 40 minutes. Upon consumption of **S14** as monitored by TLC, the reaction mixture was cooled to 0 °C, quenched with a saturated NH_4Cl solution (10 mL), and then diluted with water (10 mL). The aqueous layer was extracted with CH_2Cl_2 (3 x 10 mL). The combined organic layers were then washed with brine, dried over Na_2SO_4, and concentrated under vacuum. The crude material was purified with column chromatography using 100% hexanes to 97:3 hexanes : EtOAc to afford compound **17d** in 95% yield (632 mg, 1.960 mmol) as a colorless oil.

Rf: 0.6 in 80:20 (Hexanes : EtOAc)

^{1}H NMR: ^{1}H NMR (400 MHz, $CDCl_3$) δ 7.49 – 7.42 (m, 2H), 7.36 – 7.31 (m, 2H), 7.28 – 7.24 (m, 1H), 5.71 (q, *J* = 1.6 Hz, 1H), 5.45 (dd, *J* = 1.9, 1.0 Hz, 1H), 4.23 (s, 2H), 2.66 (s, 1H), 2.20 – 2.13 (m, 2H), 2.04 –1.97 (m, 1H), 1.93 – 1.87 (m, 1H), 1.80 (s, 3H), 1.72 – 1.62 (m, 1H), 1.59 – 1.49 (m, 1H).

^{13}C NMR: (125 MHz, $CDCl_3$) δ = 149.2, 146.3, 128.6, 127.9, 127.9, 126.9, 126.0, 126.0, 122.5, 117.1, 76.3, 75.9, 41.2, 31.1, 18.8, 16.8.

IR: *f* (cm^{-1}) = 3027, 1672, 1428, 698.

HRMS: (ESI-TOF) *m/z*: $[(M-H_2O)+H]^+$ = 305.0535 calculated for $C_{16}H_{18}BrO$; Found 305.0502.

2-(allyloxy)-3-ethyl-3,4,5,6-tetrahydro-[1,1'-biphenyl]-3-ol (19)

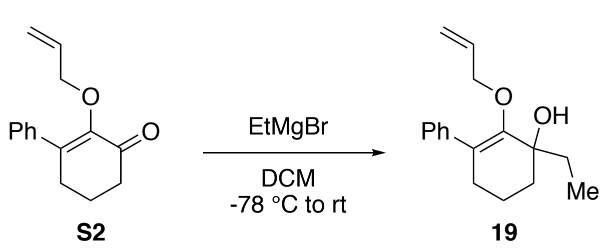

Procedure: Ketone **S2** (503 mg, 2.200 mmol) was dissolved in CH_2Cl_2 (11 mL, 0.2 M). After cooling the solution to -78 °C, ethylmagnesium bromide (1.1 mL, 3.0 M in Et_2O, 3.310 mmol) was added dropwise. The solution was then warmed to room temperature and stirred for 1 hour. Upon consumption of **S2** as monitored by TLC, the reaction mixture was cooled to 0 °C, quenched with a saturated NH_4Cl solution (10 mL), and then diluted with water (10 mL). The aqueous layer was extracted with CH_2Cl_2 (3 x 10 mL). The combined organic layers were then washed with brine, dried over Na_2SO_4, and concentrated under vacuum. The crude material was purified by column chromatography using 100% hexanes to 90:10 hexanes : EtOAc to afford compound **19** in 87% yield (495 mg, 1.920 mmol) as a colorless oil.

Rf: 0.7 in 90:10 (Hexanes : EtOAc)

^{1}H NMR: (500 MHz, $CDCl_3$) δ = 7.36 – 7.29 (m, 4H), 7.22 (tt, *J* = 6.5, 1.6 Hz, 1H), 5.77 – 5.68 (m, 1H), 5.09 (dq, *J* = 17.2, 1.6 Hz, 1H), 5.04 (dq, *J* = 10.4, 1.2 Hz, 1H), 3.86 (d, *J* = 1.3 Hz, 1H), 3.85 (d, *J* = 1.4 Hz, 1H), 2.53 – 2.47 (m, 1H), 2.29 – 2.22 (m, 1H), 2.03 (br s, 1H), 1.88 – 1.67 (m, 6H), 0.97 (t, *J* = 7.5 Hz, 3H).

^{13}C NMR: (125 MHz, $CDCl_3$) δ = 153.6, 140.3, 134.7, 128.4, 128.2, 126.7, 122.1, 116.4, 74.1, 73.8, 33.9, 32.3, 31.4, 19.4, 8.5.

IR: *f* (cm^{-1}) = 3466, 2965, 2935, 1645, 1292, 1442, 1336, 1260, 1127, 978.

HRMS: (ESI-TOF) *m/z*: $[(M\text{-}H_2O)+H]^+$ = 241.1587 calculated for $C_{17}H_{21}O$; Found 241.1586.

3-(2-(allyloxy)-4'-methoxy-3-methyl-3,4,5,6-tetrahydro-[1,1'-biphenyl]-3-yl)-1*H*-indole (21)

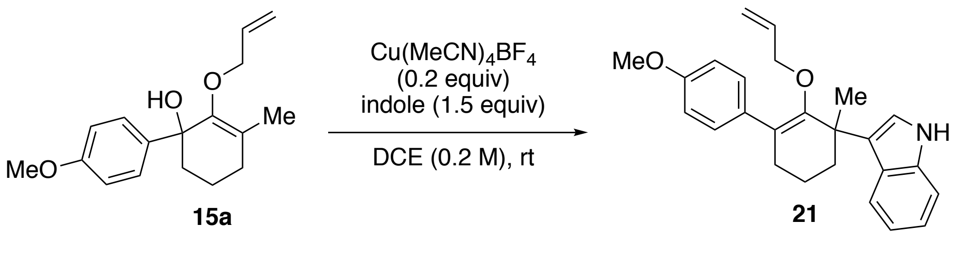

Procedure: α-Hydroxyl enol ether **15a** (171 mg, 0.624 mmol) was dissolved in DCE (3 mL, 0.2 M). Indole (110 mg, 0.936 mmol) was added, followed by addition of $Cu(MeCN)_4BF_4$ (39 mg, 0.124 mmol). The reaction mixture was stirred at room temperature for 17 hours, at which the starting material was fully consumed as monitored by TLC. The crude reaction mixture was concentrated under vacuum and then purified by column chromatography using 100% hexanes to 55:45 hexanes : CH_2Cl_2 to afford compound **21** in 80% yield (186 mg, 0.498 mmol) as a white solid.

Rf: 0.6 in 50:50 (Hexanes : CH_2Cl_2)

^{1}H NMR: (500 MHz, $CDCl_3$) δ = 7.92 (bs, 1H), 7.85 (d, *J* = 8.0 Hz, 1H), 7.40 – 7.37 (m, 2H), 7.34 (d, *J* = 8.1 Hz, 1H), 7.18 – 7.15 (m, 1H), 7.12 – 7.07 (m, 2H), 6.89 – 6.86 (m, 2H), 5.47 – 5.39 (m, 1H), 4.84 – 4.78 (m, 2H), 3.82 (s, 3H), 3.71 – 3.61 (m, 2H), 2.57 (t, *J* = 6.3 Hz, 2H), 2.49 – 2.41 (m, 1H), 1.82 – 1.74 (m, 3H), 1.71 (s, 3H).

^{13}C NMR: (125 MHz, $CDCl_3$) δ = 157.9, 156.4, 137.0, 134.9, 133.8, 129.5, 126.0, 123.7, 122.1, 121.4, 120.8, 119.3, 118.9, 115.4, 113.5, 111.3, 73.6, 55.2, 40.5, 38.9, 31.8, 25.0, 20.2.

IR: *ƒ* (cm^{-1}) = 3413, 2925, 1509, 1243, 1099, 1012.

HRMS: (ESI-TOF) *m/z*: $(M+H)^+$ = 374.2114 calculated for $C_{25}H_{28}NO_2$; Found 374.2110.

3-(2-((allyl-2-d)oxy)-3-methyl-3,4,5,6-tetrahydro-[1,1'-biphenyl]-3-yl)-1H-indole (22a)

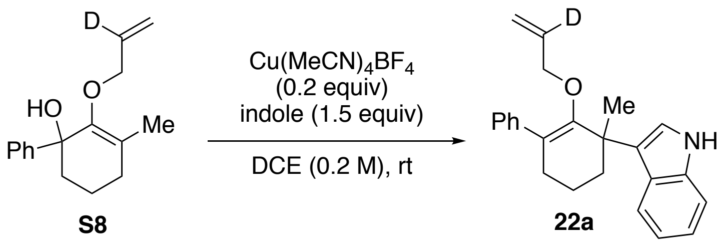

Procedure: α-Hydroxyl enol ether **S8** (112 mg, 0.457 mmol) was dissolved in DCE (2.5 mL, 0.2 M). Indole (81 mg, 0.685 mmol) was added, followed by addition of $Cu(MeCN)_4BF_4$ (29 mg,

0.091 mmol). The reaction mixture was stirred at room temperature for 24 hours, at which the starting material was fully consumed as monitored by TLC. The crude reaction mixture was concentrated under vacuum and then purified by column chromatography using 100% hexanes to 60:40 hexanes : CH_2Cl_2 to afford compound **22a** in 79% yield (123 mg, 0.357 mmol) as a white solid.

Rf: 0.5 in 50:50 (Hexanes : CH_2Cl_2)

^{1}H NMR: (500 MHz, $CDCl_3$) δ = 7.92 (bs, 1H), 7.86 (d, *J* = 7.9 Hz, 1H), 7.44 (dd, *J* = 8.2, 1.4 Hz, 2H), 7.37 – 7.30 (m, 3H), 7.22 – 7.19 (m, 1H), 7.19 – 7.15 (m, 1H), 7.11 – 7.07 (m, 2H), 4.78 – 4.76 (m, 1H), 4.76 – 4.74 (m, 1H), 3.65 (d, *J* = 12.8 Hz, 1H), 3.57 (d, *J* = 12.8 Hz, 1H), 2.58 (t, *J* = 6.2 Hz, 2H), 2.49 – 2.42 (m, 1H), 1.83 – 1.75 (m, 3H), 1.71 (s, 3H).

^{13}C NMR: (125 MHz, $CDCl_3$) δ =156.9, 141.6, 137.0, 128.5, 128.1, 126.2, 126.0, 123.6, 122.1, 121.5, 120.8, 120.0, 118.9, 115.3, 111.3, 73.7, 40.5, 38.8, 31.7, 25.1, 20.2.

IR: *f* (cm^{-1}) = 3413, 2926, 1696, 1597, 1491, 1132.

HRMS: (ESI-TOF) *m/z*: $(M+H)^+$ = 345.2071 calculated for $C_{24}H_{25}DNO$; Found 345.2075.

(*Z*)-3-(2-((allyl-3-*d*)oxy)-3-methyl-3,4,5,6-tetrahydro-[1,1'-biphenyl]-3-yl)-1*H*-indole (23a)

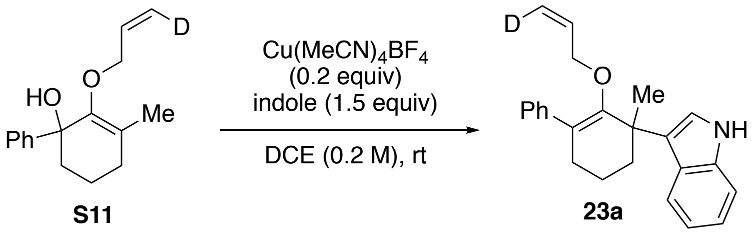

Procedure: α-Hydroxyl enol ether **S11** (2.02 g, 8.230 mmol) was dissolved in DCE (29 mL, 0.2 M). Indole (1.45 g, 12.350 mmol) was added, followed by addition of $Cu(MeCN)_4BF_4$ (367 mg, 1.170 mmol). The reaction mixture was stirred at room temperature for 18 hours, at which the starting material was fully consumed as monitored by TLC. The crude reaction mixture was

concentrated under vacuum and then purified by column chromatography using 100% hexanes to 55:45 hexanes : CH_2Cl_2 to afford compound **23a** in 86% yield (2.44 g, 7.080 mmol) as a white solid.

Rf: 0.6 in 50:50 (Hexanes : CH_2Cl_2)

^{1}H NMR: (400 MHz, $CDCl_3$) δ = 7.93 (s, 1H), 7.87 – 7.82 (m, 1H), 7.47 – 7.42 (m, 2H), 7.38 – 7.30 (m, 3H), 7.23 – 7.14 (m, 2H), 7.12 – 7.06 (m, 2H), 5.43 – 5.30 (m, 1H), 4.76 (dt, *J* = 10.4, 1.2 Hz, 1H), 3.70 – 3.52 (m, 2H), 2.61 – 2.53 (m, 1H), 2.51 – 2.37 (m, 1H), 1.83 – 1.75 (m, 3H), 1.70 (s, 3H).

^{13}C NMR: (125 MHz, $CDCl_3$) δ = 156.9, 141.6, 137.0, 134.6, 128.5, 128.1, 126.2, 126.0, 122.1, 121.5, 120.8, 120.0, 118.9, 111.3, 73.7, 40.4, 38.8, 31.7, 25.1, 20.2.

IR: *f* (cm^{-1}) = 2932, 2862, 1642, 1490, 1416, 1132, 1102.

HRMS: (ESI-TOF) *m/z*: $(M+H)^+$ = 345.2071 calculated for $C_{24}H_{24}DNO$; Found 345.2095.

3-(2-(allyloxy)-1,3-dimethylcyclohex-2-en-1-yl)-1*H*-indole (24a)

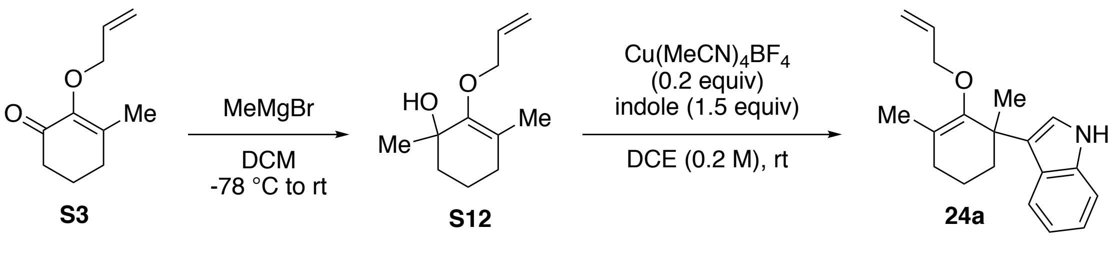

Procedure: Ketone **S3** (485 mg, 2.920 mmol) was dissolved in CH_2Cl_2 (15 mL, 0.2 M). After cooling to -78 °C, methylmagnesium bromide (1.1 mL, 3.0 M in Et_2O, 3.500 mmol) was then added dropwise. The reaction was then warmed to room temperature and stirred for 1 hour. Upon consumption of **S3** as monitored by TLC, the reaction mixture was cooled to 0 °C, quenched with a saturated NH_4Cl solution (10 mL), and then diluted with water (10 mL). The aqueous layer was extracted with CH_2Cl_2 (3 x 10 mL). The combined organic layers were then washed with brine,

dried over Na_2SO_4, and concentrated under vacuum. The crude material was purified by column chromatography using 100% hexanes to 97:3 hexanes : EtOAc to afford compound **S12** in 68% yield (360 mg, 1.980 mmol) as a colorless oil.

Rf: 0.4 in 90:10 (Hexanes : EtOAc)

^{1}H NMR: (500 MHz, $CDCl_3$) δ = 6.03 (ddt, *J* = 15.9, 10.7, 5.3 Hz, 1H), 5.37 – 5.33 (m, 1H), 5.22 – 5.18 (m, 1H), 4.41 – 4.35 (m, 1H), 4.31 – 4.26 (m, 1H), 2.06 – 1.98 (m, 2H), 1.95 – 1.92 (m, 1H), 1.81 – 1.74 (m, 1H), 1.72 – 1.66 (m, 2H), 1.65 (s, 3H), 1.58 – 2.56 (m, 1H), 1.36 (s, 3H).

^{13}C NMR: (125 MHz, $CDCl_3$) δ = 151.6, 134.9, 120.2, 116.5, 74.7, 71.2, 39.2, 31.2, 27.0, 19.3, 16.7.

IR: *f* (cm^{-1}) = 3448, 3932, 2885, 1671, 1450, 1367, 1256, 1143, 1106, 986.

HRMS: (ESI-TOF) *m/z*: $(M-H_2O)^+$ = 165.1307 calculated for $C_{11}H_{17}O$; Found 165.1273.

α-Hydroxyl enol ether **S12** (153 mg, 0.840 mmol) was dissolved in DCE (4.2 mL, 0.2 M). Indole (147 mg, 1.26 mmol) was added, followed by addition of $Cu(MeCN)_4BF_4$ (52 mg, 0.168 mmol). The reaction mixture was stirred at room temperature for 15 hours, at which the starting material was fully consumed as monitored by TLC. The crude reaction mixture was concentrated under vacuum and then purified by column chromatography using 100% hexanes to 50:50 hexanes : CH_2Cl_2 to afford compound **24a** in 97% yield (228 mg, 0.810 mmol) as a white solid.

Rf: 0.6 in 50:50 (Hexanes : CH_2Cl_2)

^{1}H NMR: (500 MHz, $CDCl_3$) δ = 7.90 (bs, 1H), 7.70 (d, *J* = 8.0 Hz, 1H), 7.33 (d, *J* = 8.1 Hz, 1H), 7.15 (t, *J* = 7.2 Hz, 1H), 7.05 (t, *J* = 7.6 Hz, 1H), 7.03 (s, 1H), 5.67 (ddt, *J* = 15.7, 10.5, 5.3 Hz, 1H), 5.02 (d, *J* = 17.3 Hz, 1H), 4.95 (d, *J* = 10.4 Hz, 1H), 4.00 (dd, *J* = 12.8, 5.3 Hz, 1H), 3.85 (dd,

J = 12.8, 5.3 Hz, 1H), 2.36 – 2.21 (m, 2H), 2.13 – 2.08 (m, 1H), 1.76 (s, 3H), 1.71 – 1.67 (m, 1H), 1.64 (s, 3H), 1.64 – 1.57 (m, 2H).

^{13}C NMR: (125 MHz, $CDCl_3$) δ = 153.9, 137.0, 135.1, 126.1, 123.7, 122.2, 121.4, 121.0, 118.8, 118.0, 115.4, 111.2, 73.8, 39.8, 39.5, 31.7, 25.4, 19.8, 17.3.

IR: *f* (cm^{-1}) = 3412, 2929, 2863, 1669, 1456, 1244, 1192, 1167, 1128, 1013.

HRMS: (ESI-TOF) *m/z*: $(M+H)^+$ = 282.1852 calculated for $C_{19}H_{23}NO$; Found 282.1850.

2-hydroxy-5,6-dihydro-[1,1'-biphenyl]-3(4*H*)-one (S1)

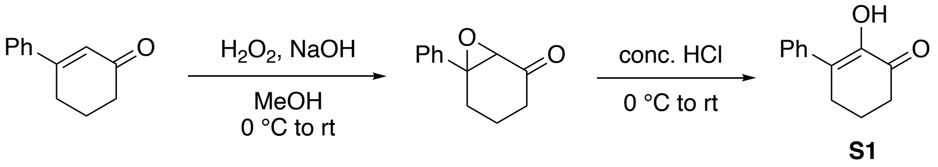

Procedure: 5,6-Dihydro-[1,1'-biphenyl]-3(4*H*)-one (2.0 g, 11.600 mmol) was dissolved in MeOH (20 mL). The solution was cooled to 0 °C. Hydrogen peroxide (6.6 mL, 113.900 mmol, 30% aqueous solution) was then added dropwise, followed by addition of aqueous NaOH (3.0 mL, 11.6 mmol, 4 M). After stirring at room temperature for 2 hours, the reaction mixture was then concentrated under vacuum to remove methanol. CH_2Cl_2 (15 mL) and DI H_2O (15 mL) were then added sequentially. The aqueous layer was extracted with CH_2Cl_2 (3 x 10 mL). The combined organic layers were then washed with brine, dried over Na_2SO_4, and concentrated under vacuum to yield crude epoxide material. In a round bottom flask equipped with stir bar, crude epoxide material was cooled to 0 °C. Ice cold concentrated aqueous HCl (10 mL, 12 M) was then added dropwise. The mixture was stirred at room temperature for 12 hours. After cooling to 0 °C, the reaction was diluted with CH_2Cl_2 (15 mL), followed by addition of DI H_2O (15 mL). The crude mixture was extracted with CH_2Cl_2 (3 x 15 mL). The combined organic layers were then washed

with saturated $NaHCO_3$ (15 mL), dried over Na_2SO_4, and concentrated under vacuum to afford known compound **S1** (1.12 g, 5.950 mmol) in 52% overall yield.

6-((Allyl-2-*d*)oxy)-5-methyl-3,4-dihydro-[1,1'-biphenyl]-1(2*H*)-ol (S8)

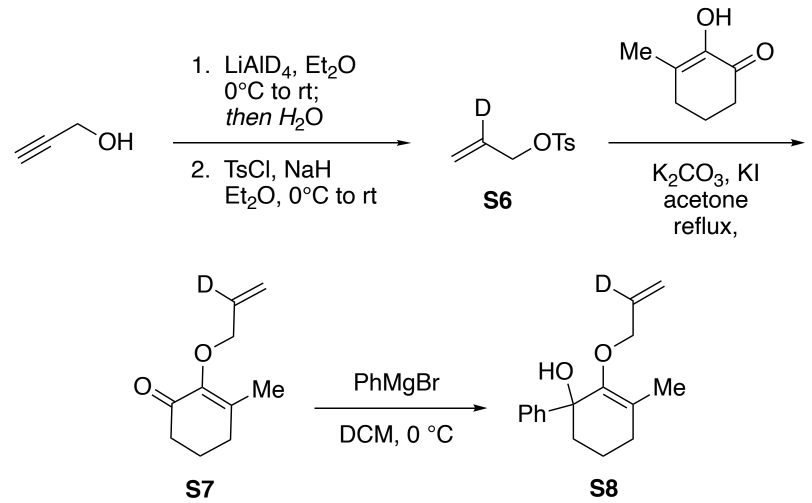

Procedure: Propargyl alcohol (250 mg, 4.460 mmol) was dissolved in Et_2O (3 mL). This solution was added dropwise to a suspension of $LiAlD_4$ (374 mg, 8.920 mmol) in Et_2O (10 mL) at 0 °C under N_2 atmosphere. The resulting mixture was allowed to warm to room temperature and stirred for 17 hours. After cooling to 0 °C, the reaction was slowly quenched with water (1 mL) dropwise. The mixture was further stirred for 3 hours at 0 °C, warmed to room temperature, and then filtered through celite. After washing the celite pad with Et_2O (15 mL), the combined filtrate was dried over Na_2SO_4. The organic solvent was removed carefully under vacuum in a rotary evaporator at 37 °C bath to afford colorless liquid, which was used immediately in the next step without further purification.

A solution of freshly prepared deuterated allyl alcohol in Et_2O (10 mL) was added dropwise into a suspension of NaH (102 mg, 4.460 mmol) in Et_2O (1 mL) at 0 °C. The mixture was stirred for 15 minutes, followed by addition of solution of tosyl chloride (760 mg, 4.040 mmol) in Et_2O

(10 mL). After stirring at room temperature for 18 hours, the reaction was quenched with saturated NH_4Cl solution (20 mL) and diluted with DI water (10 mL). The aqueous layer was extracted with Et_2O (3 x 20 mL). The combined organic layers were then washed with brine, dried over Na_2SO_4, and concentrated under vacuum. The crude material was purified with column chromatography using 100% hexanes to 96:4 hexanes : EtOAc to afford compound **S6** in 38% yield over 2 steps (353 mg, 1.660 mmol) as a colorless oil.

Rf: 0.5 in 80:20 (Hexanes : EtOAc)

^{1}H NMR: (500 MHz, $CDCl_3$) δ = 7.80 (d, *J* = 8.4 Hz, 2H), 7.34 (d, *J* = 8.4 Hz, 2H), 5.32 – 5.29 (m, 1H), 5.26 – 5.24 (m, 1H), 4.53 (s, 2H), 2.45 (s, 3H).

^{13}C NMR: (125 MHz, $CDCl_3$) δ = 144.8, 133.3, 129.8, 127.9, 120.2, 70.7, 21.6.

2-Hydroxy-3-methylcyclohex-2-en-1-one (209 mg, 1.660 mmol) was dissolved in acetone (15 mL). A solution of compound **S6** (353 mg, 1.66 mmol) in acetone (5 mL) was added, followed by addition of KI (275 mg, 1.657 mmol) and K_2CO_3 (457 mg, 3.314 mmol). The mixture was then warmed to reflux and stirred for 24 hours. After cooling to room temperature, acetone was removed from the reaction mixture under vacuum. The leftover residue was then redissolved in EtOAc. DI water (10 mL) was then added, and the aqueous layer was extracted with EtOAc (3 x 15 mL). The combined organic layers were then washed with brine, dried over Na_2SO_4, and concentrated under vacuum. The crude material was purified with column chromatography using 100% hexanes to 95:5 hexanes : EtOAc to afford compound **S7** in 41% yield (111 mg, 0.663 mmol) as a yellow oil.

Rf: 0.3 in 80:20 (Hexanes : EtOAc)

^{1}H NMR: (500 MHz, $CDCl_3$) δ = 5.28 – 5.26 (m, 1H), 5.19 – 5.16 (m, 1H), 4.33 (s, 2H), 2.46 – 2.42 (m, 2H), 2.38 (t, J = 5.5 Hz, 2H), 1.96 – 1.90 (m, 5H).

^{13}C NMR: (125 MHz, $CDCl_3$) δ = 194.9, 147.7, 146.5, 117.6, 72.8, 38.7, 31.5, 22.1, 17.9.

IR: *f* (cm^{-1}) = 2930, 1672, 1630, 1378, 1150.

HRMS: (ESI-TOF) *m/z*: $(M+H)^+$ = 168.1129 calculated for $C_{10}H_{14}DO_2$; Found 168.1124.

Ketone **S7** (100 mg, 0.598 mmol) was dissolved in CH_2Cl_2 (5 mL). After cooling to 0 °C, phenylmagnesium bromide (0.3 mL, 3.0 M in Et_2O, 0.780 mmol) was added dropwise. The reaction mixture was then stirred for 3 hours at 0 °C. Upon consumption of **S7** as monitored by TLC, the reaction mixture was cooled to 0 °C, quenched with a saturated NH_4Cl solution (5 mL), and then diluted with water (10 mL). The aqueous layer was extracted with CH_2Cl_2 (3 x 10 mL). The combined organic layers were then washed with brine, dried over Na_2SO_4, and concentrated under vacuum. The crude material was purified with column chromatography using 100% hexanes to 98:2 hexanes : EtOAc to afford compound **S8** in 84% yield (125mg, 0.509 mmol) as a colorless oil.

Rf: 0.5 in 80:20 (Hexanes : EtOAc)

^{1}H NMR: (500 MHz, $CDCl_3$) δ = 7.52 – 7.48 (m, 2H), 7.33 (t, J = 7.7 Hz, 2H), 7.26 – 7.22 (m, 1H), 5.13 – 5.10 (m, 1H), 5.07 – 5.04 (m, 1H), 4.14 (s, 2H), 2.73 (s, 1H), 2.23 – 2.13 (m, 2H), 2.04 – 1.98 (m, 1H), 1.93 – 1.87 (m, 1H), 1.79 (d, J = 1.0 Hz, 3H), 1.70 – 1.62 (m, 1H), 1.57 – 1.48 (m, 1H).

^{13}C NMR: (125 MHz, $CDCl_3$) δ = 149.7, 146.8, 127.8, 126.8, 126.1, 121.5, 116.3, 75.7, 74.2, 40.8, 31.1, 18.8, 16.8.

IR: *f* (cm^{-1}) = 3476, 2934, 1697, 1448, 1161

HRMS: (ESI-TOF) *m/z*: $(M+H)^+$ = 228.1493 calculated for $C_{16}H_{18}DO$; Found 228.1492.

(*Z*)-6-((allyl-3-*d*)oxy)-5-methyl-3,4-dihydro-[1,1'-biphenyl]-1(2*H*)-ol (S11)

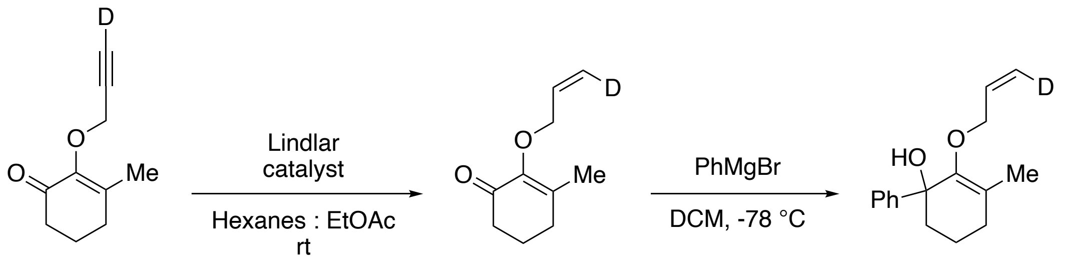

Procedure: A known ketone **S9** (1.66 g, 10.100 mmol) was dissolved in a 1:1 mixture of hexanes : EtOAc (100 mL, 0.1 M). Lindlar's catalyst (167 mg, 0.503 mmol) was added to the reaction mixture, which was then purged with hydrogen gas under balloon pressure. The reaction was stirred for 3 days, at which the starting material was fully consumed as monitored by TLC. After filtering the reaction mixture through a celite pad, the filtrate was concentrated under vacuum to obtain compound **S10** (1.66 g, 10.060 mmol) which was used in the next step without further purification.

Ketone **S10** (1.66 g, 10.060 mmol) was dissolved in CH_2Cl_2 (50 mL). After cooling to -78 °C, phenylmagnesium bromide (4.0 mL, 3.0 M in Et_2O, 11.920 mmol) was added dropwise. The reaction mixture was then stirred at room temperature for 30 minutes. Upon consumption of **S10** as monitored by TLC, the reaction mixture was cooled to 0 °C, quenched with a saturated NH_4Cl solution (10 mL), and then diluted with water (10 mL). The aqueous layer was extracted with CH_2Cl_2 (3 x 10 mL). The combined organic layers were then washed with brine, dried over Na_2SO_4, and concentrated under vacuum. The crude material was purified with column

chromatography using 100% hexanes to 95:5 hexanes : EtOAc to afford compound **S11** in 82% overall yield (2.02 g, 8.230 mmol) as a colorless oil.

Rf: 0.5 in 80:20 (Hexanes : EtOAc)

^{1}H NMR: (500 MHz, $CDCl_3$) δ = 7.50 (dd, *J* = 8.2, 1.3 Hz, 2H), 7.35 – 7.30 (m, 2H), 7.26 – 7.22 (m, 1H), 5.79 – 5.73 (m, 1H), 5.04 (dt, *J* = 10.5, 1.4 Hz, 1H), 4.15 (s, 1H), 4.14 (s, 1H), 2.72 (s, 1H), 2.22 – 2.11 (m, 2H), 2.04 – 1.97 (m, 1H), 1.93 – 1.87 (m, 1H), 1.79 (s, 3H), 1.69 – 1.62 (m, 1H), 1.55 – 1.47 (m, 1H).

^{13}C NMR: (125 MHz, $CDCl_3$) δ = 146.8, 134.6, 127.8, 126.8, 126.1, 125.8, 121.6, 116.0, 75.8, 74.3, 40.8, 31.1, 18.7, 16.8.

IR: *f* (cm^{-1}) = 3488, 2930, 2862, 1674, 1491, 1447, 1071.

HRMS: (ESI-TOF) *m/z*: $[(M\text{-}H_2O)+H]^+$ = 228.1493 calculated for $C_{16}H_{18}DO$; Found 228.1490.

(±)-(2R)-2-allyl-5-(1H-indol-3-yl)-5-methyl-2-phenylcyclopentan-1-one (S16)

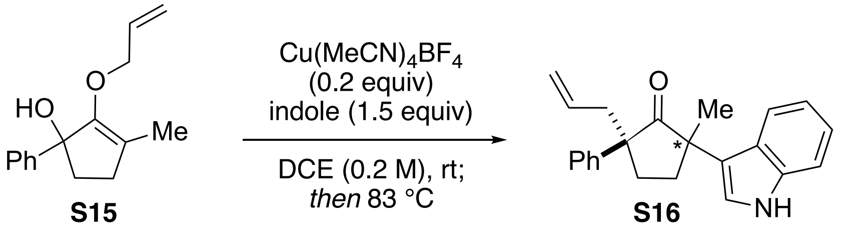

Procedure: α-Hydroxyl enol ether **S15** (150 mg, 0.652 mmol) was dissolved in DCE (3.2 mL, 0.2 M). Indole (115 mg, 0.978 mmol) was added, followed by addition of $Cu(MeCN)_4BF_4$ (41 mg, 0.130 mmol). The reaction mixture was stirred at room temperature for 22 hours, at which the starting material was fully consumed as monitored by TLC. The solution was then heated to 83 °C in a preheated oil bath for 22 hours. Upon complete Claisen rearrangement as monitored by TLC, the crude reaction mixture was concentrated under vacuum and purified by column chromatography using 100% hexanes to 95:5 hexanes : EtOAc to afford compound **S16** in 65%

yield (140 mg, 0.425 mmol) as a colorless oil. ^{1}H NMR analyses of the product indicated a 2.7:1 mixture of diastereomers, which were inseparable by column chromatography. The relative stereochemistry of the major diastereomer was not determined.

Rf: 0.4 in 50:50 (Hexanes : CH_2Cl_2)

^{1}H NMR: (500 MHz, $CDCl_3$) δ = 8.04 (s, 0.33 H), 7.82 – 7.79 (m, 1.28 H), 7.53 (d, J = 7.5 Hz, 0.83 H), 7.46 (t, J = 8.0 Hz, 3.04 H), 7.39 – 7.33 (m, 1.31 H), 7.28 – 7.17 (m, 4.80 H), 7.16 – 7.07 (m, 1.56 H), 6.98 (t, J = 7.5 Hz, 1.40 H), 6.59 (d, J = 2.5 Hz, 0.98 H), 5.54 (ddt, J = 17.1, 10.1, 7.2 Hz, 1.00 H), 5.43 (ddt, J = 17.1, 10.1, 7.1 Hz, 0.37 H), 5.09 – 5.01 (m, 2.08 H), 4.93 – 4.86 (m, 0.78 H), 2.73 – 2.67 (m, 1.84 H), 2.64 – 2.41 (m, 3.99 H), 2.28 – 2.15 (m, 2.12 H), 2.14 (m, 0.79 H), 1.60 (s, 3.17 H), 1.45 (s, 1.16 H).

^{13}C NMR: (125 MHz, $CDCl_3$) δ = 220.3, 220.2, 140.7, 140.2, 137.2, 136.9, 134.0, 133.8, 128.4, 128.31, 126.9, 126.7, 126.7, 126.6, 125.4, 125.1, 122.0, 121.8, 121.6, 121.5, 120.7, 120.3, 119.3, 119.1, 118.3, 117.9, 117.9, 117.7, 111.5, 111.3, 56.6, 56.3, 49.8, 44.8, 44.4, 34.2, 34.0, 30.5, 29.3, 25.5, 24.4.

X-RAY CRYSTALLOGRAPHY DATA

(±)-(2*R*,6*R*)-2-allyl-6-(1*H*-indol-3-yl)-6-methyl-2-phenylcyclohexan-1-one (*cis*-13)

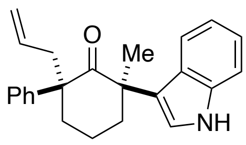

***cis*-13**

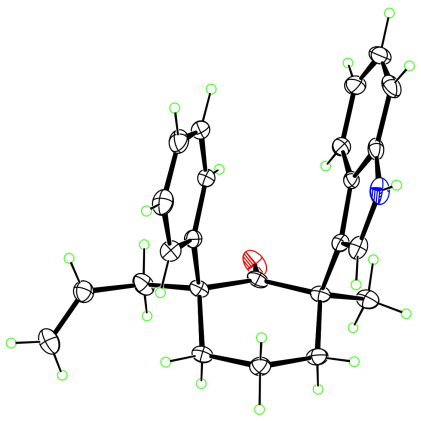

Sample Name: CCDC 2072233 / Stepherson2 (JRS2182 major)

Crystal data

$C_{24}H_{25}NO$	$Z = 4$
$M_r = 343.45$	$F(000) = 736$
Triclinic, *P*-1	$D_x = 1.205$ Mg m^{-3}
$a = 11.6991$ (5) Å	Mo *K*α radiation, λ = 0.71073 Å
$b = 11.8433$ (5) Å	Cell parameters from 9881 reflections
$c = 14.1600$ (6) Å	θ = 2.5–32.6°
α = 76.963 (2)°	μ = 0.07 mm^{-1}
β = 81.955 (2)°	$T = 90$ K
γ = 87.649 (2)°	Fragment, colourless
$V = 1892.48$ (14) Å^3	0.34 × 0.32 × 0.21 mm

Data collection

Bruker Kappa APEX-II DUO diffractometer	13781 independent reflections
Radiation source: fine-focus sealed tube	11494 reflections with $I > 2\sigma(I)$
TRIUMPH curved graphite	$R_{int} = 0.027$
ϕ and ω scans	$\theta_{max} = 32.6°$, $\theta_{min} = 1.5°$

Absorption correction: multi-scan *SADABS* (Sheldrick, 2004)	h = -17 – 16
T_{min} = 0.946, T_{max} = 0.985	k = -17 – 17
53713 measured reflections	l = -21 – 21

Refinement

Refinement on F^2	Primary atom site location: structure-invariant direct methods
Least-squares matrix: full	Secondary atom site location: difference Fourier map
$R[F^2 > 2\sigma(F^2)]$ = 0.041	Hydrogen site location: inferred from neighbouring sites
$wR(F^2)$ = 0.118	H atoms treated by a mixture of independent and constrained refinement
S = 1.03	$w = 1/[\sigma^2(F_o^2) + (0.0639P)^2 + 0.4073P]$ where $P = (F_o^2 + 2F_c^2)/3$
13781 reflections	$(\Delta/\sigma)_{max}$ = 0.001
477 parameters	$\Delta\rangle_{max}$ = 0.46 e Å^{-3}
0 restraints	$\Delta\rangle_{min}$ = -0.22 e Å^{-3}

Fractional atomic coordinates and isotropic or equivalent isotropic displacement parameters (Å^2)

	x	y	z	U_{iso}*/U_{eq}	
O1	0.04145 (5)	0.64563 (5)	0.74538 (5)	0.02006 (12)	
N1	0.27503 (6)	0.23907 (6)	0.77234 (6)	0.02032 (14)	
H1N	0.3279 (11)	0.1819 (11)	0.7758 (9)	0.024*	
C1	0.14134 (7)	0.61524 (6)	0.72633 (5)	0.01470 (13)	
C2	0.17205 (7)	0.53255 (7)	0.65624 (6)	0.01606 (13)	
C3	0.28016 (8)	0.57828 (8)	0.58394 (6)	0.02062 (15)	
H3A	0.3064	0.5193	0.5458	0.025*	
H3B	0.2584	0.6489	0.5374	0.025*	

C4	0.38069 (7)	0.60703 (8)	0.63219 (6)	0.02011 (15)	
H4A	0.4451	0.6390	0.5812	0.024*	
H4B	0.4086	0.5355	0.6740	0.024*	
C5	0.34238 (7)	0.69527 (7)	0.69418 (6)	0.01746 (14)	
H5A	0.3230	0.7691	0.6503	0.021*	
H5B	0.4081	0.7101	0.7266	0.021*	
C6	0.23765 (6)	0.65837 (6)	0.77317 (5)	0.01409 (13)	
C7	0.07117 (8)	0.52868 (8)	0.59796 (7)	0.02327 (17)	
H7A	0.0024	0.4987	0.6432	0.035*	
H7B	0.0919	0.4779	0.5522	0.035*	
H7C	0.0550	0.6070	0.5614	0.035*	
C8	0.19223 (7)	0.41237 (7)	0.71704 (6)	0.01449 (13)	
C9	0.28836 (7)	0.34363 (7)	0.70631 (6)	0.01759 (14)	
H9	0.3548	0.3654	0.6597	0.021*	
C10	0.16929 (7)	0.23824 (7)	0.82862 (6)	0.01753 (14)	
C11	0.11374 (7)	0.34559 (6)	0.79565 (6)	0.01464 (13)	
C12	0.00386 (7)	0.36637 (7)	0.84389 (6)	0.01675 (14)	
H12	-0.0365	0.4367	0.8226	0.020*	
C13	-0.04453 (8)	0.28275 (8)	0.92280 (6)	0.02066 (15)	
H13	-0.1179	0.2971	0.9563	0.025*	
C14	0.01275 (8)	0.17709 (8)	0.95427 (7)	0.02382 (17)	
H14	-0.0224	0.1215	1.0088	0.029*	
C15	0.11947 (8)	0.15304 (7)	0.90694 (7)	0.02341 (17)	
H15	0.1576	0.0810	0.9270	0.028*	
C16	0.19326 (7)	0.76402 (7)	0.81701 (6)	0.01701	

				(14)	
H16A	0.1246	0.7395	0.8658	0.020*	
H16B	0.2537	0.7847	0.8524	0.020*	
C17	0.16144 (7)	0.87116 (7)	0.74553 (6)	0.01908 (15)	
H17	0.0954	0.8681	0.7140	0.023*	
C18	0.21936 (9)	0.96958 (8)	0.72349 (8)	0.02711 (19)	
H18A	0.2858	0.9756	0.7536	0.033*	
H18B	0.1944	1.0341	0.6776	0.033*	
C19	0.26487 (7)	0.56144 (7)	0.86002 (5)	0.01438 (13)	
C20	0.37679 (7)	0.52031 (8)	0.87160 (6)	0.01963 (15)	
H20	0.4393	0.5500	0.8230	0.024*	
C21	0.39766 (8)	0.43608 (8)	0.95383 (7)	0.02292 (16)	
H21	0.4741	0.4083	0.9603	0.028*	
C22	0.30790 (8)	0.39245 (8)	1.02620 (6)	0.02172 (16)	
H22	0.3227	0.3355	1.0823	0.026*	
C23	0.19624 (8)	0.43283 (7)	1.01587 (6)	0.01956 (15)	
H23	0.1342	0.4034	1.0650	0.023*	
C24	0.17505 (7)	0.51633 (7)	0.93360 (6)	0.01640 (14)	
H24	0.0983	0.5432	0.9272	0.020*	
O2	0.48597 (5)	0.10069 (6)	0.74255 (6)	0.02530 (14)	
N2	0.83868 (6)	-0.21336 (6)	0.71645 (6)	0.02329 (15)	
H2N	0.9036 (12)	-0.2563 (11)	0.7194 (9)	0.028*	
C25	0.57962 (7)	0.08110 (7)	0.77189 (6)	0.01614 (14)	
C26	0.60769 (6)	-0.04158 (7)	0.83041 (6)	0.01509 (13)	
C27	0.65707 (7)	-0.03085 (8)	0.92363 (6)	0.01928	

				(15)	
H27A	0.5925	-0.0147	0.9721	0.023*	
H27B	0.6918	-0.1063	0.9521	0.023*	
C28	0.74793 (7)	0.06318 (7)	0.90780 (6)	0.01924 (15)	
H28A	0.7694	0.0690	0.9717	0.023*	
H28B	0.8181	0.0420	0.8675	0.023*	
C29	0.70164 (7)	0.17950 (7)	0.85695 (6)	0.01793 (14)	
H29A	0.7610	0.2392	0.8494	0.022*	
H29B	0.6330	0.2011	0.8988	0.022*	
C30	0.66817 (6)	0.17909 (7)	0.75554 (6)	0.01523 (13)	
C31	0.49545 (7)	-0.11229 (8)	0.86222 (6)	0.02054 (15)	
H31A	0.5111	-0.1862	0.9063	0.031*	
H31B	0.4669	-0.1272	0.8044	0.031*	
H31C	0.4371	-0.0684	0.8962	0.031*	
C32	0.69015 (6)	-0.10453 (6)	0.76684 (5)	0.01403 (13)	
C33	0.79448 (7)	-0.15426 (7)	0.78687 (6)	0.01880 (15)	
H33	0.8310	-0.1487	0.8415	0.023*	
C34	0.76312 (7)	-0.20291 (7)	0.64908 (6)	0.01988 (15)	
C35	0.66803 (7)	-0.13476 (6)	0.67779 (6)	0.01496 (13)	
C36	0.58003 (8)	-0.10808 (7)	0.61791 (6)	0.01956 (15)	
H36	0.5151	-0.0627	0.6355	0.023*	
C37	0.58960 (10)	-0.14912 (9)	0.53276 (7)	0.0284 (2)	
H37	0.5312	-0.1304	0.4913	0.034*	
C38	0.68421 (11)	-0.21790 (9)	0.50689 (7)	0.0339 (2)	
H38	0.6883	-0.2453	0.4484	0.041*	
C39	0.77126 (9)	-0.24658 (8)	0.56452 (8)	0.0298 (2)	

H39	0.8345	-0.2943	0.5474	0.036*	
C40	0.60975 (8)	0.29727 (7)	0.71532 (7)	0.02132 (16)	
H40A	0.5797	0.2939	0.6540	0.026*	
H40B	0.5433	0.3103	0.7634	0.026*	
C41	0.69096 (8)	0.39763 (8)	0.69533 (7)	0.02283 (16)	
H41	0.7473	0.4068	0.6390	0.027*	
C42	0.68947 (9)	0.47436 (8)	0.75095 (8)	0.02815 (19)	
H42A	0.6343	0.4679	0.8079	0.034*	
H42B	0.7435	0.5357	0.7338	0.034*	
C43	0.77113 (7)	0.16038 (7)	0.68089 (6)	0.01526 (13)	
C44	0.75088 (8)	0.12695 (7)	0.59611 (6)	0.01867 (14)	
H44	0.6739	0.1150	0.5866	0.022*	
C45	0.84152 (9)	0.11095 (8)	0.52554 (6)	0.02511 (18)	
H45	0.8264	0.0854	0.4696	0.030*	
C46	0.95425 (9)	0.13231 (8)	0.53663 (7)	0.02749 (19)	
H46	1.0164	0.1213	0.4885	0.033*	
C47	0.97531 (8)	0.16993 (8)	0.61873 (7)	0.02453 (17)	
H47	1.0520	0.1869	0.6257	0.029*	
C48	0.88483 (7)	0.18297 (7)	0.69086 (6)	0.01922 (15)	
H48	0.9004	0.2074	0.7472	0.023*	

Atomic displacement parameters (Å^2)

	U^{11}	U^{22}	U^{33}	U^{12}	U^{13}	U^{23}
O1	0.0154 (3)	0.0182 (3)	0.0277 (3)	0.0027 (2)	-0.0055 (2)	-0.0063 (2)
N1	0.0177 (3)	0.0156 (3)	0.0300 (4)	0.0051 (2)	-0.0067 (3)	-0.0090 (3)

C1	0.0160 (3)	0.0126 (3)	0.0153 (3)	0.0001 (2)	-0.0039 (2)	-0.0018 (2)
C2	0.0182 (3)	0.0160 (3)	0.0153 (3)	0.0006 (3)	-0.0054 (3)	-0.0046 (3)
C3	0.0256 (4)	0.0219 (4)	0.0143 (3)	-0.0015 (3)	-0.0006 (3)	-0.0048 (3)
C4	0.0188 (3)	0.0210 (4)	0.0198 (4)	-0.0027 (3)	0.0035 (3)	-0.0062 (3)
C5	0.0170 (3)	0.0173 (3)	0.0175 (3)	-0.0028 (3)	0.0005 (3)	-0.0040 (3)
C6	0.0142 (3)	0.0142 (3)	0.0143 (3)	-0.0004 (2)	-0.0022 (2)	-0.0040 (2)
C7	0.0272 (4)	0.0232 (4)	0.0230 (4)	0.0018 (3)	-0.0140 (3)	-0.0065 (3)
C8	0.0149 (3)	0.0145 (3)	0.0160 (3)	0.0015 (2)	-0.0046 (2)	-0.0063 (2)
C9	0.0161 (3)	0.0182 (3)	0.0212 (3)	0.0015 (3)	-0.0032 (3)	-0.0099 (3)
C10	0.0187 (3)	0.0143 (3)	0.0222 (4)	0.0014 (3)	-0.0077 (3)	-0.0067 (3)
C11	0.0159 (3)	0.0134 (3)	0.0171 (3)	0.0008 (2)	-0.0056 (3)	-0.0064 (2)
C12	0.0157 (3)	0.0167 (3)	0.0199 (3)	-0.0008 (3)	-0.0037 (3)	-0.0073 (3)
C13	0.0202 (4)	0.0227 (4)	0.0211 (4)	-0.0055 (3)	-0.0024 (3)	-0.0083 (3)
C14	0.0290 (4)	0.0209 (4)	0.0217 (4)	-0.0081 (3)	-0.0065 (3)	-0.0017 (3)
C15	0.0282 (4)	0.0153 (3)	0.0277 (4)	-0.0010 (3)	-0.0112 (3)	-0.0020 (3)
C16	0.0198 (3)	0.0153 (3)	0.0169 (3)	0.0005 (3)	-0.0028 (3)	-0.0054 (3)
C17	0.0214 (4)	0.0158 (3)	0.0214 (4)	0.0009 (3)	-0.0053 (3)	-0.0059 (3)
C18	0.0290 (4)	0.0150 (4)	0.0392 (5)	0.0000 (3)	-0.0119 (4)	-0.0055 (3)
C19	0.0149 (3)	0.0145 (3)	0.0147 (3)	-0.0004 (2)	-0.0034 (2)	-0.0045 (2)
C20	0.0154 (3)	0.0224 (4)	0.0203 (4)	0.0007 (3)	-0.0034	-0.0027

					(3)	(3)
C21	0.0189 (4)	0.0245 (4)	0.0249 (4)	0.0029 (3)	-0.0079 (3)	-0.0021 (3)
C22	0.0254 (4)	0.0195 (4)	0.0201 (4)	0.0007 (3)	-0.0077 (3)	-0.0016 (3)
C23	0.0220 (4)	0.0184 (3)	0.0175 (3)	-0.0024 (3)	-0.0021 (3)	-0.0023 (3)
C24	0.0153 (3)	0.0168 (3)	0.0174 (3)	-0.0005 (3)	-0.0025 (3)	-0.0043 (3)
O2	0.0158 (3)	0.0243 (3)	0.0419 (4)	0.0069 (2)	-0.0128 (3)	-0.0158 (3)
N2	0.0154 (3)	0.0170 (3)	0.0336 (4)	0.0051 (2)	0.0038 (3)	-0.0027 (3)
C25	0.0125 (3)	0.0199 (3)	0.0191 (3)	0.0036 (3)	-0.0029 (3)	-0.0109 (3)
C26	0.0112 (3)	0.0201 (3)	0.0152 (3)	0.0000 (2)	-0.0009 (2)	-0.0069 (3)
C27	0.0192 (3)	0.0256 (4)	0.0138 (3)	-0.0047 (3)	-0.0017 (3)	-0.0055 (3)
C28	0.0195 (3)	0.0232 (4)	0.0163 (3)	-0.0032 (3)	-0.0062 (3)	-0.0041 (3)
C29	0.0182 (3)	0.0214 (4)	0.0165 (3)	-0.0001 (3)	-0.0042 (3)	-0.0080 (3)
C30	0.0139 (3)	0.0168 (3)	0.0171 (3)	0.0031 (2)	-0.0047 (2)	-0.0071 (3)
C31	0.0139 (3)	0.0274 (4)	0.0217 (4)	-0.0038 (3)	0.0025 (3)	-0.0107 (3)
C32	0.0121 (3)	0.0146 (3)	0.0145 (3)	0.0011 (2)	-0.0002 (2)	-0.0025 (2)
C33	0.0134 (3)	0.0181 (3)	0.0220 (4)	0.0017 (3)	-0.0011 (3)	0.0004 (3)
C34	0.0203 (4)	0.0124 (3)	0.0238 (4)	-0.0015 (3)	0.0079 (3)	-0.0039 (3)
C35	0.0164 (3)	0.0124 (3)	0.0150 (3)	-0.0008 (2)	0.0018 (2)	-0.0032 (2)
C36	0.0239 (4)	0.0182 (3)	0.0172 (3)	-0.0044 (3)	-0.0036 (3)	-0.0040 (3)
C37	0.0413 (5)	0.0274 (4)	0.0181 (4)	-0.0144 (4)	-0.0036 (4)	-0.0060 (3)

C38	0.0525 (6)	0.0286 (5)	0.0215 (4)	-0.0191 (4)	0.0113 (4)	-0.0140 (4)
C39	0.0367 (5)	0.0189 (4)	0.0316 (5)	-0.0085 (3)	0.0179 (4)	-0.0131 (3)
C40	0.0207 (4)	0.0189 (4)	0.0277 (4)	0.0068 (3)	-0.0100 (3)	-0.0093 (3)
C41	0.0264 (4)	0.0185 (4)	0.0239 (4)	0.0037 (3)	-0.0065 (3)	-0.0042 (3)
C42	0.0296 (5)	0.0203 (4)	0.0364 (5)	0.0013 (3)	-0.0041 (4)	-0.0105 (4)
C43	0.0163 (3)	0.0142 (3)	0.0152 (3)	0.0023 (2)	-0.0029 (2)	-0.0029 (2)
C44	0.0255 (4)	0.0153 (3)	0.0153 (3)	-0.0018 (3)	-0.0033 (3)	-0.0029 (3)
C45	0.0389 (5)	0.0179 (4)	0.0168 (4)	-0.0046 (3)	0.0043 (3)	-0.0044 (3)
C46	0.0322 (5)	0.0188 (4)	0.0266 (4)	-0.0020 (3)	0.0115 (4)	-0.0041 (3)
C47	0.0189 (4)	0.0219 (4)	0.0292 (4)	0.0001 (3)	0.0032 (3)	-0.0022 (3)
C48	0.0162 (3)	0.0210 (4)	0.0201 (4)	0.0009 (3)	-0.0024 (3)	-0.0038 (3)

Geometric parameters (Å, º)

O1—C1	1.2202 (10)	O2—C25	1.2206 (9)
N1—C9	1.3728 (11)	N2—C34	1.3713 (13)
N1—C10	1.3736 (11)	N2—C33	1.3756 (12)
N1—H1N	0.896 (13)	N2—H2N	0.897 (13)
C1—C6	1.5427 (10)	C25—C30	1.5440 (11)
C1—C2	1.5436 (11)	C25—C26	1.5491 (11)
C2—C8	1.5163 (11)	C26—C32	1.5156 (10)
C2—C7	1.5396 (11)	C26—C31	1.5437 (11)
C2—C3	1.5443 (12)	C26—C27	1.5452 (11)
C3—C4	1.5260 (12)	C27—C28	1.5290 (12)
C3—H3A	0.9900	C27—H27A	0.9900
C3—H3B	0.9900	C27—H27B	0.9900

C4—C5	1.5261 (11)	C28—C29	1.5199 (12)
C4—H4A	0.9900	C28—H28A	0.9900
C4—H4B	0.9900	C28—H28B	0.9900
C5—C6	1.5437 (11)	C29—C30	1.5413 (11)
C5—H5A	0.9900	C29—H29A	0.9900
C5—H5B	0.9900	C29—H29B	0.9900
C6—C19	1.5409 (11)	C30—C43	1.5313 (11)
C6—C16	1.5580 (11)	C30—C40	1.5560 (11)
C7—H7A	0.9800	C31—H31A	0.9800
C7—H7B	0.9800	C31—H31B	0.9800
C7—H7C	0.9800	C31—H31C	0.9800
C8—C9	1.3725 (10)	C32—C33	1.3711 (10)
C8—C11	1.4436 (11)	C32—C35	1.4450 (11)
C9—H9	0.9500	C33—H33	0.9500
C10—C15	1.3966 (12)	C34—C39	1.3978 (13)
C10—C11	1.4164 (10)	C34—C35	1.4149 (11)
C11—C12	1.4084 (11)	C35—C36	1.4055 (11)
C12—C13	1.3855 (12)	C36—C37	1.3874 (12)
C12—H12	0.9500	C36—H36	0.9500
C13—C14	1.4059 (13)	C37—C38	1.4035 (17)
C13—H13	0.9500	C37—H37	0.9500
C14—C15	1.3827 (14)	C38—C39	1.3776 (18)
C14—H14	0.9500	C38—H38	0.9500
C15—H15	0.9500	C39—H39	0.9500
C16—C17	1.5034 (11)	C40—C41	1.5037 (13)
C16—H16A	0.9900	C40—H40A	0.9900
C16—H16B	0.9900	C40—H40B	0.9900
C17—C18	1.3254 (12)	C41—C42	1.3282 (13)
C17—H17	0.9500	C41—H41	0.9500
C18—H18A	0.9500	C42—H42A	0.9500
C18—H18B	0.9500	C42—H42B	0.9500
C19—C20	1.3966 (11)	C43—C44	1.3984 (11)
C19—C24	1.4013 (11)	C43—C48	1.4000 (11)

C20—C21	1.3950 (12)	C44—C45	1.3896 (12)
C20—H20	0.9500	C44—H44	0.9500
C21—C22	1.3872 (13)	C45—C46	1.3897 (15)
C21—H21	0.9500	C45—H45	0.9500
C22—C23	1.3883 (12)	C46—C47	1.3905 (14)
C22—H22	0.9500	C46—H46	0.9500
C23—C24	1.3920 (11)	C47—C48	1.3938 (12)
C23—H23	0.9500	C47—H47	0.9500
C24—H24	0.9500	C48—H48	0.9500
C9—N1—C10	108.85 (7)	C34—N2—C33	108.95 (7)
C9—N1—H1N	123.8 (8)	C34—N2—H2N	127.4 (8)
C10—N1—H1N	127.4 (8)	C33—N2—H2N	123.4 (8)
O1—C1—C6	120.05 (7)	O2—C25—C30	120.54 (7)
O1—C1—C2	120.36 (7)	O2—C25—C26	119.79 (7)
C6—C1—C2	119.58 (6)	C30—C25—C26	119.61 (6)
C8—C2—C7	109.23 (7)	C32—C26—C31	107.93 (6)
C8—C2—C1	108.24 (6)	C32—C26—C27	112.09 (6)
C7—C2—C1	109.71 (6)	C31—C26—C27	108.06 (6)
C8—C2—C3	111.80 (7)	C32—C26—C25	110.25 (6)
C7—C2—C3	108.86 (7)	C31—C26—C25	109.29 (6)
C1—C2—C3	108.98 (6)	C27—C26—C25	109.14 (6)
C4—C3—C2	114.40 (7)	C28—C27—C26	114.91 (7)
C4—C3—H3A	108.7	C28—C27—H27A	108.5
C2—C3—H3A	108.7	C26—C27—H27A	108.5
C4—C3—H3B	108.7	C28—C27—H27B	108.5
C2—C3—H3B	108.7	C26—C27—H27B	108.5
H3A—C3—H3B	107.6	H27A—C27—H27B	107.5
C3—C4—C5	110.16 (7)	C29—C28—C27	110.52 (7)
C3—C4—H4A	109.6	C29—C28—H28A	109.5
C5—C4—H4A	109.6	C27—C28—H28A	109.5
C3—C4—H4B	109.6	C29—C28—H28B	109.5
C5—C4—H4B	109.6	C27—C28—H28B	109.5

H4A—C4—H4B	108.1	H28A—C28—H28B	108.1
C4—C5—C6	114.75 (7)	C28—C29—C30	113.13 (6)
C4—C5—H5A	108.6	C28—C29—H29A	109.0
C6—C5—H5A	108.6	C30—C29—H29A	109.0
C4—C5—H5B	108.6	C28—C29—H29B	109.0
C6—C5—H5B	108.6	C30—C29—H29B	109.0
H5A—C5—H5B	107.6	H29A—C29—H29B	107.8
C19—C6—C1	108.01 (6)	C43—C30—C29	113.46 (6)
C19—C6—C5	113.99 (6)	C43—C30—C25	110.44 (6)
C1—C6—C5	109.16 (6)	C29—C30—C25	106.32 (6)
C19—C6—C16	106.28 (6)	C43—C30—C40	108.51 (7)
C1—C6—C16	109.98 (6)	C29—C30—C40	108.78 (6)
C5—C6—C16	109.35 (6)	C25—C30—C40	109.25 (6)
C2—C7—H7A	109.5	C26—C31—H31A	109.5
C2—C7—H7B	109.5	C26—C31—H31B	109.5
H7A—C7—H7B	109.5	H31A—C31—H31B	109.5
C2—C7—H7C	109.5	C26—C31—H31C	109.5
H7A—C7—H7C	109.5	H31A—C31—H31C	109.5
H7B—C7—H7C	109.5	H31B—C31—H31C	109.5
C9—C8—C11	106.01 (7)	C33—C32—C35	106.28 (7)
C9—C8—C2	126.61 (7)	C33—C32—C26	127.22 (7)
C11—C8—C2	127.36 (7)	C35—C32—C26	126.19 (6)
C8—C9—N1	110.50 (7)	C32—C33—N2	110.19 (7)
C8—C9—H9	124.7	C32—C33—H33	124.9
N1—C9—H9	124.7	N2—C33—H33	124.9
N1—C10—C15	129.70 (8)	N2—C34—C39	129.79 (8)
N1—C10—C11	107.75 (7)	N2—C34—C35	107.91 (7)
C15—C10—C11	122.53 (8)	C39—C34—C35	122.30 (9)
C12—C11—C10	118.36 (7)	C36—C35—C34	118.71 (7)
C12—C11—C8	134.71 (7)	C36—C35—C32	134.56 (7)

C10—C11—C8	106.89 (7)	C34—C35—C32	106.68 (7)
C13—C12—C11	119.07 (7)	C37—C36—C35	118.91 (9)
C13—C12—H12	120.5	C37—C36—H36	120.5
C11—C12—H12	120.5	C35—C36—H36	120.5
C12—C13—C14	121.38 (8)	C36—C37—C38	121.07 (9)
C12—C13—H13	119.3	C36—C37—H37	119.5
C14—C13—H13	119.3	C38—C37—H37	119.5
C15—C14—C13	120.89 (8)	C39—C38—C37	121.42 (8)
C15—C14—H14	119.6	C39—C38—H38	119.3
C13—C14—H14	119.6	C37—C38—H38	119.3
C14—C15—C10	117.74 (8)	C38—C39—C34	117.55 (9)
C14—C15—H15	121.1	C38—C39—H39	121.2
C10—C15—H15	121.1	C34—C39—H39	121.2
C17—C16—C6	116.35 (6)	C41—C40—C30	112.77 (7)
C17—C16—H16A	108.2	C41—C40—H40A	109.0
C6—C16—H16A	108.2	C30—C40—H40A	109.0
C17—C16—H16B	108.2	C41—C40—H40B	109.0
C6—C16—H16B	108.2	C30—C40—H40B	109.0
H16A—C16—H16B	107.4	H40A—C40—H40B	107.8
C18—C17—C16	124.37 (8)	C42—C41—C40	124.58 (9)
C18—C17—H17	117.8	C42—C41—H41	117.7
C16—C17—H17	117.8	C40—C41—H41	117.7
C17—C18—H18A	120.0	C41—C42—H42A	120.0
C17—C18—H18B	120.0	C41—C42—H42B	120.0
H18A—C18—H18B	120.0	H42A—C42—H42B	120.0
C20—C19—C24	118.04 (7)	C44—C43—C48	118.29 (7)
C20—C19—C6	122.78 (7)	C44—C43—C30	119.09 (7)
C24—C19—C6	119.10 (7)	C48—C43—C30	122.47 (7)
C21—C20—C19	120.62 (8)	C45—C44—C43	121.09 (8)
C21—C20—H20	119.7	C45—C44—H44	119.5
C19—C20—H20	119.7	C43—C44—H44	119.5
C22—C21—C20	120.69 (8)	C44—C45—C46	120.14 (8)

C22—C21—H21	119.7	C44—C45—H45	119.9
C20—C21—H21	119.7	C46—C45—H45	119.9
C21—C22—C23	119.34 (8)	C45—C46—C47	119.41 (8)
C21—C22—H22	120.3	C45—C46—H46	120.3
C23—C22—H22	120.3	C47—C46—H46	120.3
C22—C23—C24	120.10 (8)	C46—C47—C48	120.50 (9)
C22—C23—H23	120.0	C46—C47—H47	119.7
C24—C23—H23	120.0	C48—C47—H47	119.7
C23—C24—C19	121.21 (7)	C47—C48—C43	120.49 (8)
C23—C24—H24	119.4	C47—C48—H48	119.8
C19—C24—H24	119.4	C43—C48—H48	119.8
O1—C1—C2—C8	-102.96 (8)	O2—C25—C26—C32	105.02 (8)
C6—C1—C2—C8	76.40 (8)	C30—C25—C26—C32	-77.95 (8)
O1—C1—C2—C7	16.15 (10)	O2—C25—C26—C31	-13.47 (10)
C6—C1—C2—C7	-164.50 (7)	C30—C25—C26—C31	163.57 (6)
O1—C1—C2—C3	135.25 (8)	O2—C25—C26—C27	-131.45 (8)
C6—C1—C2—C3	-45.40 (9)	C30—C25—C26—C27	45.59 (9)
C8—C2—C3—C4	-69.60 (9)	C32—C26—C27—C28	77.46 (9)
C7—C2—C3—C4	169.64 (7)	C31—C26—C27—C28	-163.73 (7)
C1—C2—C3—C4	50.01 (9)	C25—C26—C27—C28	-44.98 (9)
C2—C3—C4—C5	-56.72 (9)	C26—C27—C28—C29	53.95 (9)
C3—C4—C5—C6	56.16 (9)	C27—C28—C29—C30	-60.35 (9)
O1—C1—C6—C19	99.74 (8)	C28—C29—C30—C43	-65.28 (9)
C2—C1—C6—	-79.61 (8)	C28—C29—	56.31 (8)

C19		C30—C25	
O1—C1—C6—C5	-135.82 (8)	C28—C29—C30—C40	173.86 (7)
C2—C1—C6—C5	44.82 (9)	O2—C25—C30—C43	-110.22 (8)
O1—C1—C6—C16	-15.85 (10)	C26—C25—C30—C43	72.77 (8)
C2—C1—C6—C16	164.80 (6)	O2—C25—C30—C29	126.29 (8)
C4—C5—C6—C19	71.97 (9)	C26—C25—C30—C29	-50.72 (8)
C4—C5—C6—C1	-48.89 (9)	O2—C25—C30—C40	9.06 (10)
C4—C5—C6—C16	-169.25 (7)	C26—C25—C30—C40	-167.95 (6)
C7—C2—C8—C9	114.50 (9)	C31—C26—C32—C33	-115.34 (9)
C1—C2—C8—C9	-126.09 (8)	C27—C26—C32—C33	3.55 (11)
C3—C2—C8—C9	-6.05 (10)	C25—C26—C32—C33	125.35 (8)
C7—C2—C8—C11	-63.53 (10)	C31—C26—C32—C35	57.41 (10)
C1—C2—C8—C11	55.88 (9)	C27—C26—C32—C35	176.29 (7)
C3—C2—C8—C11	175.93 (7)	C25—C26—C32—C35	-61.91 (10)
C11—C8—C9—N1	0.15 (9)	C35—C32—C33—N2	-0.03 (9)
C2—C8—C9—N1	-178.21 (7)	C26—C32—C33—N2	173.88 (7)
C10—N1—C9—C8	-0.64 (9)	C34—N2—C33—C32	-0.04 (10)
C9—N1—C10—C15	-177.53 (8)	C33—N2—C34—C39	179.17 (9)
C9—N1—C10—C11	0.86 (9)	C33—N2—C34—C35	0.09 (9)
N1—C10—C11—C12	-178.76 (7)	N2—C34—C35—C36	177.75 (7)

C15—C10—C11—C12	-0.22 (11)	C39—C34—C35—C36	-1.41 (12)
N1—C10—C11—C8	-0.76 (8)	N2—C34—C35—C32	-0.11 (9)
C15—C10—C11—C8	177.78 (7)	C39—C34—C35—C32	-179.27 (8)
C9—C8—C11—C12	177.89 (8)	C33—C32—C35—C36	-177.28 (9)
C2—C8—C11—C12	-3.76 (13)	C26—C32—C35—C36	8.73 (14)
C9—C8—C11—C10	0.37 (8)	C33—C32—C35—C34	0.09 (9)
C2—C8—C11—C10	178.72 (7)	C26—C32—C35—C34	-173.90 (7)
C10—C11—C12—C13	1.47 (11)	C34—C35—C36—C37	-0.19 (12)
C8—C11—C12—C13	-175.83 (8)	C32—C35—C36—C37	176.93 (9)
C11—C12—C13—C14	-1.26 (12)	C35—C36—C37—C38	1.09 (13)
C12—C13—C14—C15	-0.28 (13)	C36—C37—C38—C39	-0.43 (15)
C13—C14—C15—C10	1.51 (13)	C37—C38—C39—C34	-1.12 (14)
N1—C10—C15—C14	176.92 (8)	N2—C34—C39—C38	-176.91 (9)
C11—C10—C15—C14	-1.27 (12)	C35—C34—C39—C38	2.05 (13)
C19—C6—C16—C17	179.59 (7)	C43—C30—C40—C41	-58.37 (9)
C1—C6—C16—C17	-63.73 (9)	C29—C30—C40—C41	65.49 (9)
C5—C6—C16—C17	56.12 (9)	C25—C30—C40—C41	-178.84 (7)
C6—C16—C17—C18	-111.60 (10)	C30—C40—C41—C42	-105.25 (10)
C1—C6—C19—C20	128.53 (8)	C29—C30—C43—C44	162.79 (7)
C5—C6—C19—	7.03 (10)	C25—C30—	43.54 (9)

C20		C43—C44	
C16—C6—C19—C20	-113.49 (8)	C40—C30—C43—C44	-76.19 (9)
C1—C6—C19—C24	-54.99 (9)	C29—C30—C43—C48	-21.62 (11)
C5—C6—C19—C24	-176.48 (7)	C25—C30—C43—C48	-140.88 (8)
C16—C6—C19—C24	63.00 (9)	C40—C30—C43—C48	99.40 (8)
C24—C19—C20—C21	0.54 (12)	C48—C43—C44—C45	3.09 (12)
C6—C19—C20—C21	177.05 (8)	C30—C43—C44—C45	178.86 (8)
C19—C20—C21—C22	-0.72 (14)	C43—C44—C45—C46	-2.42 (13)
C20—C21—C22—C23	0.46 (14)	C44—C45—C46—C47	-0.10 (14)
C21—C22—C23—C24	-0.04 (13)	C45—C46—C47—C48	1.86 (14)
C22—C23—C24—C19	-0.13 (12)	C46—C47—C48—C43	-1.15 (13)
C20—C19—C24—C23	-0.12 (12)	C44—C43—C48—C47	-1.31 (12)
C6—C19—C24—C23	-176.77 (7)	C30—C43—C48—C47	-176.93 (8)

Hydrogen-bond geometry (Å, º)

D—H···*A*	*D*—H	H···*A*	*D*···*A*	*D*—H···*A*
N1—H1*N*···O2	0.896 (13)	2.093 (13)	2.9399 (9)	157.3 (11)
N2—H2*N*···O1^{i}	0.897 (13)	1.977 (13)	2.8675 (9)	172.2 (12)

Symmetry code: (i) *x*+1, *y*-1, *z*.

(±)-(2*R*,6*R*)-2-allyl-6-(5-methoxy-1*H*-indol-3-yl)-6-methyl-2-phenylcyclohexanone (14a)

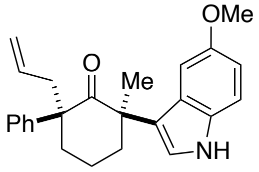

14a

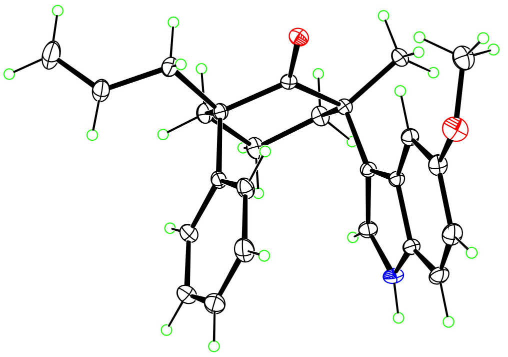

Sample Name: CCDC 2072234 / Satish3 (SCP1154)

Crystal data

$C_{25}H_{27}NO_2$	$F(000) = 800$
$M_r = 373.47$	$D_x = 1.245$ Mg m^{-3}
Monoclinic, $P2_1/c$	Mo $K\alpha$ radiation, $\lambda = 0.71073$ Å
$a = 8.6040$ (3) Å	Cell parameters from 9952 reflections
$b = 17.4999$ (6) Å	$\theta = 2.8–38.5°$
$c = 13.2548$ (4) Å	$\mu = 0.08$ mm^{-1}
$\beta = 93.3695$ (18)°	$T = 90$ K
$V = 1992.31$ (11) Å^3	Prism, colourless
$Z = 4$	0.19 × 0.12 × 0.12 mm

Data collection

Bruker Kappa APEX-II DUO diffractometer	12554 independent reflections
Radiation source: fine-focus sealed tube	8930 reflections with $I > 2\sigma(I)$
TRIUMPH curved graphite	$R_{int} = 0.058$
ϕ and ω scans	$\theta_{max} = 40.3°$, $\theta_{min} = 1.9°$
Absorption correction: multi-scan *SADABS* (Sheldrick, 2004)	$h = -15 – 15$
$T_{min} = 0.951$, $T_{max} = 0.991$	$k = -30 – 31$

68558 measured reflections	$l = -24 – 4$

Refinement

Refinement on F^2	0 restraints
Least-squares matrix: full	Hydrogen site location: mixed
$R[F^2 > 2\sigma(F^2)] = 0.048$	H atoms treated by a mixture of independent and constrained refinement
$wR(F^2) = 0.135$	$w = 1/[\sigma^2(F_o^2) + (0.0679P)^2 + 0.2809P]$ where $P = (F_o^2 + 2F_c^2)/3$
$S = 1.03$	$(\Delta/\sigma)_{max} = 0.001$
12554 reflections	$\Delta\rho_{max} = 0.64$ e Å^{-3}
258 parameters	$\Delta\rho_{min} = -0.31$ e Å^{-3}

Fractional atomic coordinates and isotropic or equivalent isotropic displacement parameters (Å^2)

	x	*y*	*z*	U_{iso}*/U_{eq}	
O1	0.61493 (6)	0.61385 (3)	0.81610 (4)	0.01349 (9)	
O2	0.32035 (7)	0.86754 (3)	0.84311 (5)	0.02081 (11)	
N1	0.60434 (8)	0.79651 (4)	0.49523 (5)	0.01674 (11)	
H1N	0.6284 (14)	0.8249 (7)	0.4424 (9)	0.020*	
C1	0.68032 (7)	0.61522 (3)	0.73667 (5)	0.00999 (9)	
C2	0.85990 (7)	0.61841 (4)	0.73721 (5)	0.01110 (10)	
C3	0.91129 (8)	0.55554 (4)	0.66423 (5)	0.01416 (11)	
H3A	1.025630	0.558195	0.659825	0.017*	
H3B	0.886003	0.504894	0.692232	0.017*	
C4	0.83364 (8)	0.56272 (4)	0.55831 (6)	0.01571 (12)	
H4A	0.871638	0.521594	0.514770	0.019*	
H4B	0.861558	0.612425	0.528625	0.019*	
C5	0.65698 (8)	0.55706 (4)	0.56203 (5)	0.01361 (11)	

H5A	0.630218	0.504275	0.581538	0.016*	
H5B	0.609636	0.566091	0.493180	0.016*	
C6	0.58274 (7)	0.61308 (4)	0.63545 (5)	0.01034 (10)	
C7	0.41698 (8)	0.58494 (4)	0.65238 (5)	0.01332 (11)	
H7A	0.365382	0.621311	0.695617	0.020*	
H7B	0.422157	0.534738	0.685158	0.020*	
H7C	0.357747	0.580855	0.587160	0.020*	
C8	0.57564 (7)	0.69427 (4)	0.59590 (5)	0.01093 (10)	
C9	0.63022 (8)	0.71962 (4)	0.50619 (5)	0.01487 (11)	
H9	0.678828	0.688287	0.458717	0.018*	
C10	0.53125 (8)	0.82269 (4)	0.57791 (5)	0.01380 (11)	
C11	0.51017 (7)	0.76029 (4)	0.64337 (5)	0.01091 (10)	
C12	0.43949 (8)	0.77328 (4)	0.73549 (5)	0.01222 (10)	
H12	0.424267	0.732562	0.781263	0.015*	
C13	0.39300 (8)	0.84703 (4)	0.75722 (6)	0.01472 (11)	
C14	0.41596 (9)	0.90836 (4)	0.69073 (6)	0.01822 (13)	
H14	0.383370	0.958263	0.708176	0.022*	
C15	0.48499 (9)	0.89688 (4)	0.60085 (6)	0.01744 (13)	
H15	0.500648	0.938082	0.555897	0.021*	
C16	0.24773 (9)	0.80808 (5)	0.89626 (6)	0.01945 (13)	
H16A	0.179576	0.778529	0.849006	0.029*	
H16B	0.185912	0.830309	0.948680	0.029*	
H16C	0.327642	0.774402	0.927741	0.029*	
C17	0.91170 (7)	0.69923 (4)	0.70833 (5)	0.01210 (10)	
C18	0.84565 (8)	0.76248 (4)	0.75409 (6)	0.01526	

				(12)	
H18	0.768558	0.754647	0.801488	0.018*	
C19	0.89063 (9)	0.83670 (4)	0.73156 (7)	0.02119 (15)	
H19	0.842940	0.878974	0.762413	0.025*	
C20	1.00562 (10)	0.84877 (5)	0.66372 (7)	0.02319 (16)	
H20	1.036392	0.899287	0.647843	0.028*	
C21	1.07508 (9)	0.78671 (5)	0.61941 (7)	0.02074 (14)	
H21	1.154604	0.794718	0.573760	0.025*	
C22	1.02850 (8)	0.71262 (4)	0.64168 (6)	0.01605 (12)	
H22	1.077035	0.670520	0.610987	0.019*	
C23	0.92792 (8)	0.60060 (4)	0.84597 (5)	0.01479 (11)	
H23A	0.891337	0.639883	0.892808	0.018*	
H23B	0.887874	0.550497	0.867471	0.018*	
C24	1.10247 (9)	0.59880 (5)	0.85368 (6)	0.01831 (13)	
H24	1.155152	0.641401	0.827268	0.022*	
C25	1.18957 (11)	0.54310 (5)	0.89403 (8)	0.02696 (18)	
H25A	1.141829	0.499391	0.921361	0.032*	
H25B	1.299842	0.546814	0.895667	0.032*	

Atomic displacement parameters (Å^2)

	U^{11}	U^{22}	U^{33}	U^{12}	U^{13}	U^{23}
O1	0.0148 (2)	0.0154 (2)	0.01056 (19)	-0.00062 (16)	0.00342 (15)	-0.00203 (16)
O2	0.0248 (3)	0.0145 (2)	0.0236 (3)	0.00147 (19)	0.0061 (2)	-0.0069 (2)
N1	0.0186 (3)	0.0181 (3)	0.0136 (2)	-0.0018 (2)	0.0018 (2)	0.0059 (2)
C1	0.0117 (2)	0.0079 (2)	0.0105 (2)	0.00023 (17)	0.00147 (18)	-0.00100 (18)

C2	0.0104 (2)	0.0113 (2)	0.0116 (2)	0.00071 (18)	0.00101 (18)	-0.00091 (19)
C3	0.0126 (2)	0.0138 (3)	0.0161 (3)	0.0030 (2)	0.0019 (2)	-0.0029 (2)
C4	0.0137 (3)	0.0189 (3)	0.0148 (3)	0.0022 (2)	0.0032 (2)	-0.0054 (2)
C5	0.0137 (2)	0.0144 (3)	0.0128 (3)	0.0005 (2)	0.0018 (2)	-0.0050 (2)
C6	0.0105 (2)	0.0106 (2)	0.0100 (2)	0.00004 (17)	0.00119 (17)	-0.00136 (18)
C7	0.0113 (2)	0.0133 (2)	0.0154 (3)	-0.00155 (19)	0.0014 (2)	-0.0015 (2)
C8	0.0115 (2)	0.0117 (2)	0.0097 (2)	-0.00026 (18)	0.00092 (18)	0.00038 (18)
C9	0.0155 (3)	0.0183 (3)	0.0110 (3)	-0.0004 (2)	0.0021 (2)	0.0020 (2)
C10	0.0135 (2)	0.0132 (3)	0.0144 (3)	-0.0019 (2)	-0.0013 (2)	0.0038 (2)
C11	0.0111 (2)	0.0101 (2)	0.0114 (2)	-0.00046 (18)	-0.00016 (18)	0.00081 (19)
C12	0.0132 (2)	0.0102 (2)	0.0133 (3)	0.00048 (18)	0.00082 (19)	-0.00052 (19)
C13	0.0150 (3)	0.0110 (2)	0.0181 (3)	0.0008 (2)	0.0000 (2)	-0.0031 (2)
C14	0.0192 (3)	0.0094 (2)	0.0256 (4)	0.0004 (2)	-0.0029 (3)	-0.0008 (2)
C15	0.0188 (3)	0.0109 (2)	0.0221 (3)	-0.0023 (2)	-0.0035 (2)	0.0050 (2)
C16	0.0178 (3)	0.0223 (3)	0.0186 (3)	0.0018 (2)	0.0039 (2)	-0.0042 (3)
C17	0.0098 (2)	0.0125 (2)	0.0139 (3)	-0.00043 (18)	0.00013 (18)	-0.0001 (2)
C18	0.0123 (2)	0.0128 (3)	0.0206 (3)	-0.0007 (2)	0.0003 (2)	-0.0028 (2)
C19	0.0169 (3)	0.0125 (3)	0.0338 (4)	-0.0010 (2)	-0.0027 (3)	-0.0019 (3)
C20	0.0187 (3)	0.0161 (3)	0.0341 (4)	-0.0051 (2)	-0.0043 (3)	0.0062 (3)
C21	0.0154 (3)	0.0230 (3)	0.0237 (4)	-0.0051	0.0002 (2)	0.0060 (3)

				(2)		
C22	0.0126 (2)	0.0186 (3)	0.0172 (3)	-0.0014 (2)	0.0023 (2)	0.0017 (2)
C23	0.0140 (3)	0.0165 (3)	0.0136 (3)	0.0016 (2)	-0.0014 (2)	0.0007 (2)
C24	0.0153 (3)	0.0189 (3)	0.0202 (3)	0.0015 (2)	-0.0037 (2)	0.0002 (2)
C25	0.0218 (4)	0.0243 (4)	0.0335 (5)	0.0055 (3)	-0.0098 (3)	0.0012 (3)

Geometric parameters (Å, º)

O1—C1	1.2227 (8)	C10—C11	1.4131 (9)
O2—C13	1.3782 (9)	C11—C12	1.4141 (9)
O2—C16	1.4218 (10)	C12—C13	1.3864 (9)
N1—C9	1.3703 (10)	C12—H12	0.9500
N1—C10	1.3737 (10)	C13—C14	1.4100 (11)
N1—H1N	0.893 (12)	C14—C15	1.3768 (12)
C1—C6	1.5408 (9)	C14—H14	0.9500
C1—C2	1.5457 (9)	C15—H15	0.9500
C2—C17	1.5380 (9)	C16—H16A	0.9800
C2—C3	1.5466 (9)	C16—H16B	0.9800
C2—C23	1.5553 (10)	C16—H16C	0.9800
C3—C4	1.5241 (10)	C17—C22	1.3964 (10)
C3—H3A	0.9900	C17—C18	1.3987 (10)
C3—H3B	0.9900	C18—C19	1.3928 (11)
C4—C5	1.5269 (10)	C18—H18	0.9500
C4—H4A	0.9900	C19—C20	1.3916 (13)
C4—H4B	0.9900	C19—H19	0.9500
C5—C6	1.5457 (9)	C20—C21	1.3866 (13)
C5—H5A	0.9900	C20—H20	0.9500
C5—H5B	0.9900	C21—C22	1.3940 (11)
C6—C8	1.5145 (9)	C21—H21	0.9500
C6—C7	1.5377 (9)	C22—H22	0.9500
C7—H7A	0.9800	C23—C24	1.4997 (10)

C7—H7B	0.9800	C23—H23A	0.9900
C7—H7C	0.9800	C23—H23B	0.9900
C8—C9	1.3772 (9)	C24—C25	1.3231 (11)
C8—C11	1.4456 (9)	C24—H24	0.9500
C9—H9	0.9500	C25—H25A	0.9500
C10—C15	1.3966 (10)	C25—H25B	0.9500
C13—O2—C16	116.91 (6)	C10—C11—C12	118.89 (6)
C9—N1—C10	108.79 (6)	C10—C11—C8	106.44 (6)
C9—N1—H1N	125.9 (8)	C12—C11—C8	134.65 (6)
C10—N1—H1N	125.3 (8)	C13—C12—C11	118.24 (6)
O1—C1—C6	119.64 (6)	C13—C12—H12	120.9
O1—C1—C2	120.48 (6)	C11—C12—H12	120.9
C6—C1—C2	119.89 (5)	O2—C13—C12	124.37 (7)
C17—C2—C1	109.65 (5)	O2—C13—C14	113.90 (6)
C17—C2—C3	113.63 (5)	C12—C13—C14	121.73 (7)
C1—C2—C3	107.10 (5)	C15—C14—C13	120.88 (7)
C17—C2—C23	108.43 (5)	C15—C14—H14	119.6
C1—C2—C23	108.58 (5)	C13—C14—H14	119.6
C3—C2—C23	109.35 (5)	C14—C15—C10	117.84 (7)
C4—C3—C2	113.05 (5)	C14—C15—H15	121.1
C4—C3—H3A	109.0	C10—C15—H15	121.1
C2—C3—H3A	109.0	O2—C16—H16A	109.5
C4—C3—H3B	109.0	O2—C16—H16B	109.5
C2—C3—H3B	109.0	H16A—C16—H16B	109.5
H3A—C3—H3B	107.8	O2—C16—H16C	109.5
C3—C4—C5	110.37 (6)	H16A—C16—H16C	109.5
C3—C4—H4A	109.6	H16B—C16—H16C	109.5
C5—C4—H4A	109.6	C22—C17—C18	117.87 (6)
C3—C4—H4B	109.6	C22—C17—C2	122.79 (6)
C5—C4—H4B	109.6	C18—C17—C2	119.26 (6)

H4A—C4—H4B	108.1	C19—C18—C17	121.33 (7)
C4—C5—C6	115.35 (6)	C19—C18—H18	119.3
C4—C5—H5A	108.4	C17—C18—H18	119.3
C6—C5—H5A	108.4	C20—C19—C18	119.81 (8)
C4—C5—H5B	108.4	C20—C19—H19	120.1
C6—C5—H5B	108.4	C18—C19—H19	120.1
H5A—C5—H5B	107.5	C21—C20—C19	119.70 (7)
C8—C6—C7	109.39 (5)	C21—C20—H20	120.2
C8—C6—C1	106.70 (5)	C19—C20—H20	120.2
C7—C6—C1	110.08 (5)	C20—C21—C22	120.16 (7)
C8—C6—C5	112.76 (5)	C20—C21—H21	119.9
C7—C6—C5	108.06 (5)	C22—C21—H21	119.9
C1—C6—C5	109.84 (5)	C21—C22—C17	121.11 (7)
C6—C7—H7A	109.5	C21—C22—H22	119.4
C6—C7—H7B	109.5	C17—C22—H22	119.4
H7A—C7—H7B	109.5	C24—C23—C2	112.84 (6)
C6—C7—H7C	109.5	C24—C23—H23A	109.0
H7A—C7—H7C	109.5	C2—C23—H23A	109.0
H7B—C7—H7C	109.5	C24—C23—H23B	109.0
C9—C8—C11	106.25 (6)	C2—C23—H23B	109.0
C9—C8—C6	126.31 (6)	H23A—C23—H23B	107.8
C11—C8—C6	127.44 (6)	C25—C24—C23	125.74 (8)
N1—C9—C8	110.26 (6)	C25—C24—H24	117.1
N1—C9—H9	124.9	C23—C24—H24	117.1
C8—C9—H9	124.9	C24—C25—H25A	120.0
N1—C10—C15	129.30 (7)	C24—C25—H25B	120.0
N1—C10—C11	108.27 (6)	H25A—C25—H25B	120.0
C15—C10—C11	122.42 (7)		
O1—C1—C2—C17	-105.47 (7)	C15—C10—C11—C8	-178.40 (6)
C6—C1—C2—C17	75.04 (7)	C9—C8—C11—C10	-0.47 (7)

O1—C1—C2—C3	130.81 (6)	C6—C8—C11—C10	179.92 (6)
C6—C1—C2—C3	-48.68 (7)	C9—C8—C11—C12	-178.59 (7)
O1—C1—C2—C23	12.83 (8)	C6—C8—C11—C12	1.80 (12)
C6—C1—C2—C23	-166.65 (5)	C10—C11—C12—C13	0.37 (10)
C17—C2—C3—C4	-65.90 (7)	C8—C11—C12—C13	178.31 (7)
C1—C2—C3—C4	55.34 (7)	C16—O2—C13—C12	-19.62 (11)
C23—C2—C3—C4	172.81 (6)	C16—O2—C13—C14	159.84 (7)
C2—C3—C4—C5	-59.94 (8)	C11—C12—C13—O2	178.79 (7)
C3—C4—C5—C6	53.68 (8)	C11—C12—C13—C14	-0.63 (10)
O1—C1—C6—C8	101.30 (7)	O2—C13—C14—C15	-179.03 (7)
C2—C1—C6—C8	-79.21 (7)	C12—C13—C14—C15	0.45 (11)
O1—C1—C6—C7	-17.31 (8)	C13—C14—C15—C10	0.01 (11)
C2—C1—C6—C7	162.19 (5)	N1—C10—C15—C14	-178.66 (7)
O1—C1—C6—C5	-136.18 (6)	C11—C10—C15—C14	-0.27 (11)
C2—C1—C6—C5	43.32 (7)	C1—C2—C17—C22	-136.83 (7)
C4—C5—C6—C8	74.85 (8)	C3—C2—C17—C22	-17.04 (9)
C4—C5—C6—C7	-164.12 (6)	C23—C2—C17—C22	104.77 (7)
C4—C5—C6—C1	-44.01 (8)	C1—C2—C17—C18	46.52 (8)
C7—C6—C8—C9	-121.64 (7)	C3—C2—C17—C18	166.32 (6)
C1—C6—C8—C9	119.31 (7)	C23—C2—C17—	-71.87 (7)

		C18	
C5—C6—C8—C9	-1.37 (9)	C22—C17—C18—C19	2.17 (11)
C7—C6—C8—C11	57.90 (8)	C2—C17—C18—C19	178.98 (7)
C1—C6—C8—C11	-61.16 (8)	C17—C18—C19—C20	-1.25 (12)
C5—C6—C8—C11	178.16 (6)	C18—C19—C20—C21	-0.30 (13)
C10—N1—C9—C8	-0.31 (8)	C19—C20—C21—C22	0.85 (13)
C11—C8—C9—N1	0.48 (8)	C20—C21—C22—C17	0.13 (12)
C6—C8—C9—N1	-179.90 (6)	C18—C17—C22—C21	-1.61 (11)
C9—N1—C10—C15	178.57 (7)	C2—C17—C22—C21	-178.30 (7)
C9—N1—C10—C11	0.00 (8)	C17—C2—C23—C24	-63.50 (7)
N1—C10—C11—C12	178.77 (6)	C1—C2—C23—C24	177.43 (6)
C15—C10—C11—C12	0.08 (10)	C3—C2—C23—C24	60.90 (8)
N1—C10—C11—C8	0.29 (7)	C2—C23—C24—C25	-128.82 (9)

Hydrogen-bond geometry (Å, º)

D—H···*A*	*D*—H	H···*A*	*D*···*A*	*D*—H···*A*
N1—H1*N*···O1^{i}	0.893 (12)	1.985 (12)	2.8517 (8)	163.1 (11)

Symmetry code: (i) *x*, -*y*+3/2, *z*-1/2.

(±)-(2*R*,6*R*)-2-allyl-6-(5-iodo-1*H*-indol-3-yl)-6-methyl-2-phenylcyclohexanone (14e)

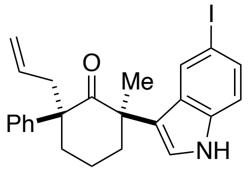

14e

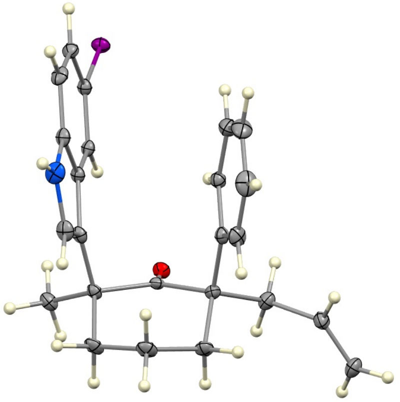

Sample Name: CCDC 2072235 / Satish8 (SCP1160)

Crystal data

$C_{24}H_{24}INO$	$F(000) = 944$
$M_r = 469.34$	$D_x = 1.526$ Mg m^{-3}
Monoclinic, $P2_1$	Mo $K\alpha$ radiation, $\lambda = 0.71073$ Å
$a = 9.3770$ (7) Å	Cell parameters from 9113 reflections
$b = 16.9386$ (13) Å	$\theta = 2.4–35.5°$
$c = 13.4807$ (10) Å	$\mu = 1.58$ mm^{-1}
$\beta = 107.382$ (1)°	$T = 90$ K
$V = 2043.4$ (3) Å^3	Needle fragment, colourless
$Z = 4$	0.20 × 0.15 × 0.08 mm

Data collection

Bruker Kappa APEX-II DUO diffractometer	20837 independent reflections
Radiation source: fine-focus sealed tube	17804 reflections with $I > 2\sigma(I)$
TRIUMPH curved graphite monochromator	$R_{int} = 0.035$
ϕ and ω scans	$\theta_{max} = 37.1°$, $\theta_{min} = 2.0°$
Absorption correction: multi-scan *SADABS* (Sheldrick, 2004)	$h = -15 – 15$

T_{min} = 0.813, T_{max} = 0.884	k = -28 – 28
84151 measured reflections	l = -22 – 22

Refinement

Refinement on F^2	Hydrogen site location: mixed
Least-squares matrix: full	H atoms treated by a mixture of independent and constrained refinement
$R[F^2 > 2\sigma(F^2)]$ = 0.030	$w = 1/[\sigma^2(F_o^2) + (0.0152P)^2 + 0.560P]$ where $P = (F_o^2 + 2F_c^2)/3$
$wR(F^2)$ = 0.052	$(\Delta/\sigma)_{max}$ = 0.003
S = 1.04	$\Delta\rho_{max}$ = 0.94 e Å^{-3}
20837 reflections	$\Delta\rho_{min}$ = -0.75 e Å^{-3}
495 parameters	Absolute structure: Flack x determined using 7466 quotients [(I+)-(I-)]/[(I+)+(I-)] (Parsons, Flack and Wagner, Acta Cryst. B69 (2013) 249-259). Crystal is a kryptoracemate.
3 restraints	Absolute structure parameter: 0.028 (4)

Fractional atomic coordinates and isotropic or equivalent isotropic displacement parameters (Å^2) for (SCP1160)

	x	y	z	U_{iso}*/U_{eq}
I1	0.89215 (2)	0.39988 (2)	0.66824 (2)	0.01463 (3)
O1	0.64888 (19)	0.19996 (10)	0.88144 (12)	0.0136 (3)
N1	1.2316 (3)	0.23110 (14)	1.0790 (2)	0.0182 (5)
H1N	1.317 (3)	0.228 (2)	1.114 (2)	0.022*
C1	0.7395 (2)	0.16411 (12)	0.95069 (17)	0.0111 (4)
C2	0.7678 (3)	0.07496 (15)	0.9391 (2)	0.0125 (5)
C3	0.7728 (3)	0.03338 (15)	1.04198 (19)	0.0165 (4)
H3A	0.671512	0.034193	1.050386	0.020*
H3B	0.801406	-0.022525	1.037951	0.020*
C4	0.8814 (3)	0.07097 (15)	1.13677 (19)	0.0178 (5)
H4A	0.983677	0.069350	1.130107	0.021*
H4B	0.881433	0.041031	1.199846	0.021*

C5	0.8362 (3)	0.15660 (14)	1.14710 (17)	0.0166 (4)
H5A	0.909555	0.180417	1.208475	0.020*
H5B	0.737662	0.156983	1.159948	0.020*
C6	0.8269 (3)	0.20835 (13)	1.05111 (17)	0.0123 (4)
C7	0.7447 (3)	0.28518 (14)	1.06035 (19)	0.0157 (4)
H7A	0.797418	0.311952	1.125417	0.024*
H7B	0.642442	0.272884	1.060053	0.024*
H7C	0.741794	0.319725	1.001458	0.024*
C8	0.9812 (3)	0.22697 (13)	1.04253 (17)	0.0123 (4)
C9	1.1147 (3)	0.20517 (15)	1.11125 (19)	0.0165 (5)
H9	1.124931	0.175930	1.173125	0.020*
C10	1.1764 (3)	0.27120 (14)	0.98723 (18)	0.0138 (4)
C11	1.0179 (3)	0.27130 (13)	0.96174 (17)	0.0114 (4)
C12	0.9347 (2)	0.30942 (12)	0.86974 (17)	0.0113 (4)
H12	0.828760	0.311633	0.850865	0.014*
C13	1.0115 (3)	0.34350 (13)	0.80770 (17)	0.0122 (4)
C14	1.1684 (3)	0.34228 (14)	0.83356 (18)	0.0142 (4)
H14	1.216855	0.366435	0.788790	0.017*
C15	1.2518 (3)	0.30603 (14)	0.92392 (19)	0.0156 (4)
H15	1.357786	0.304877	0.942418	0.019*
C16	0.9131 (3)	0.06555 (14)	0.90909 (19)	0.0129 (4)
C17	0.9343 (3)	0.11334 (13)	0.82993 (18)	0.0140 (4)
H17	0.860281	0.151409	0.798206	0.017*
C18	1.0597 (3)	0.10681 (15)	0.7965 (2)	0.0196 (5)
H18	1.071195	0.139984	0.742550	0.024*
C19	1.1688 (3)	0.05135 (17)	0.8424 (2)	0.0232 (5)
H19	1.255937	0.046616	0.820655	0.028*
C20	1.1488 (3)	0.00292 (17)	0.9204 (2)	0.0244 (5)
H20	1.223032	-0.035075	0.952015	0.029*
C21	1.0218 (3)	0.00931 (14)	0.9529 (2)	0.0181 (5)
H21	1.009090	-0.024993	1.005380	0.022*
C22	0.6337 (3)	0.04192 (14)	0.85004 (19)	0.0158 (4)
H22A	0.539136	0.059609	0.861295	0.019*

H22B	0.637720	0.064014	0.782929	0.019*
C23	0.6339 (3)	-0.04655 (14)	0.84390 (19)	0.0162 (4)
H23	0.722486	-0.071611	0.839639	0.019*
C24	0.5197 (3)	-0.09194 (15)	0.84401 (18)	0.0187 (5)
H24A	0.429293	-0.068925	0.848183	0.022*
H24B	0.527861	-0.147673	0.839914	0.022*
I2	0.07802 (2)	0.39469 (2)	0.31355 (2)	0.01835 (3)
O2	0.49969 (19)	0.19970 (10)	0.24508 (12)	0.0142 (3)
N2	0.5672 (3)	0.21892 (13)	0.65340 (15)	0.0186 (4)
H2N	0.584 (4)	0.208 (2)	0.7134 (18)	0.022*
C25	0.5689 (2)	0.16516 (12)	0.32421 (16)	0.0098 (4)
C26	0.5463 (3)	0.07566 (13)	0.33572 (17)	0.0113 (4)
C27	0.7031 (3)	0.03722 (14)	0.37037 (19)	0.0148 (4)
H27A	0.692118	-0.019480	0.384326	0.018*
H27B	0.747673	0.040930	0.312573	0.018*
C28	0.8100 (3)	0.07489 (15)	0.46714 (19)	0.0170 (4)
H28A	0.770281	0.067965	0.526861	0.020*
H28B	0.908383	0.048298	0.483986	0.020*
C29	0.8291 (3)	0.16270 (13)	0.44922 (18)	0.0150 (4)
H29A	0.883467	0.168321	0.396956	0.018*
H29B	0.892339	0.186113	0.514945	0.018*
C30	0.6826 (2)	0.21071 (13)	0.41209 (17)	0.0116 (4)
C31	0.7187 (3)	0.29047 (13)	0.37172 (18)	0.0141 (4)
H31A	0.628341	0.323148	0.351353	0.021*
H31B	0.754667	0.282000	0.311365	0.021*
H31C	0.796144	0.317296	0.426604	0.021*
C32	0.6108 (3)	0.22378 (13)	0.49786 (17)	0.0123 (4)
C33	0.6633 (4)	0.19753 (17)	0.5985 (2)	0.0170 (6)
H33	0.753293	0.168678	0.626356	0.020*
C34	0.4498 (3)	0.25944 (14)	0.58956 (18)	0.0154 (4)
C35	0.4735 (3)	0.26513 (14)	0.49022 (18)	0.0123 (4)
C36	0.3649 (3)	0.30370 (13)	0.40982 (17)	0.0126 (4)
H36	0.377445	0.309272	0.342803	0.015*

C37	0.2394 (3)	0.33332 (13)	0.43069 (19)	0.0144 (4)
C38	0.2158 (3)	0.32537 (15)	0.5290 (2)	0.0184 (5)
H38	0.127203	0.345578	0.539898	0.022*
C39	0.3212 (3)	0.28837 (15)	0.6088 (2)	0.0185 (5)
H39	0.306819	0.282640	0.675297	0.022*
C40	0.4552 (3)	0.06139 (13)	0.41175 (17)	0.0118 (4)
C41	0.4940 (3)	0.00467 (16)	0.4903 (2)	0.0167 (5)
H41	0.581551	-0.026161	0.499052	0.020*
C42	0.4063 (3)	-0.00734 (16)	0.5558 (2)	0.0200 (5)
H42	0.435082	-0.045901	0.609281	0.024*
C43	0.2773 (3)	0.03643 (16)	0.5439 (2)	0.0211 (5)
H43	0.217776	0.028076	0.588947	0.025*
C44	0.2362 (3)	0.09267 (15)	0.4654 (2)	0.0183 (5)
H44	0.148266	0.123166	0.456725	0.022*
C45	0.3237 (3)	0.10429 (15)	0.3997 (2)	0.0130 (5)
H45	0.293500	0.142148	0.345402	0.016*
C46	0.4572 (3)	0.04204 (14)	0.22795 (18)	0.0142 (4)
H46A	0.355341	0.064787	0.207200	0.017*
H46B	0.506569	0.058562	0.175826	0.017*
C47	0.4458 (3)	-0.04672 (13)	0.22738 (19)	0.0142 (4)
H47	0.417313	-0.071258	0.281920	0.017*
C48	0.4730 (2)	-0.09248 (15)	0.15596 (18)	0.0164 (5)
H48A	0.501749	-0.069778	0.100395	0.020*
H48B	0.463756	-0.148145	0.160076	0.020*

Atomic displacement parameters (Å^2) for (SCP1160)

	U^{11}	U^{22}	U^{33}	U^{12}	U^{13}	U^{23}
I1	0.01476 (5)	0.01703 (6)	0.01189 (5)	0.00116 (8)	0.00365 (4)	0.00327 (7)
O1	0.0144 (8)	0.0145 (7)	0.0107 (7)	0.0018 (6)	0.0019 (6)	0.0022 (6)
N1	0.0135 (11)	0.0209 (10)	0.0145 (11)	0.0017 (8)	-0.0046 (8)	0.0033 (8)
C1	0.0123 (9)	0.0121 (10)	0.0107 (9)	-0.0002 (7)	0.0062 (8)	0.0018 (7)

C2	0.0141 (12)	0.0119 (9)	0.0126 (11)	-0.0011 (8)	0.0054 (9)	0.0004 (8)
C3	0.0219 (12)	0.0156 (10)	0.0134 (10)	0.0002 (9)	0.0073 (9)	0.0048 (8)
C4	0.0232 (12)	0.0187 (11)	0.0121 (10)	0.0035 (9)	0.0063 (9)	0.0066 (8)
C5	0.0224 (12)	0.0190 (12)	0.0092 (9)	0.0037 (9)	0.0061 (9)	0.0035 (8)
C6	0.0146 (10)	0.0133 (9)	0.0085 (8)	0.0014 (7)	0.0028 (8)	0.0005 (7)
C7	0.0177 (11)	0.0173 (11)	0.0126 (10)	0.0037 (8)	0.0051 (9)	-0.0007 (8)
C8	0.0135 (10)	0.0130 (9)	0.0089 (9)	0.0021 (7)	0.0012 (8)	0.0002 (7)
C9	0.0165 (11)	0.0170 (11)	0.0118 (10)	0.0029 (9)	-0.0021 (9)	0.0010 (8)
C10	0.0122 (10)	0.0143 (10)	0.0122 (10)	0.0002 (8)	-0.0006 (8)	-0.0013 (7)
C11	0.0116 (9)	0.0113 (9)	0.0099 (9)	0.0004 (7)	0.0010 (8)	0.0002 (7)
C12	0.0104 (9)	0.0110 (9)	0.0112 (9)	0.0000 (7)	0.0010 (7)	-0.0003 (7)
C13	0.0126 (10)	0.0120 (9)	0.0108 (9)	0.0004 (7)	0.0016 (8)	-0.0001 (7)
C14	0.0127 (10)	0.0146 (10)	0.0157 (10)	-0.0015 (7)	0.0046 (8)	-0.0011 (8)
C15	0.0086 (9)	0.0163 (10)	0.0204 (11)	-0.0009 (8)	0.0018 (8)	-0.0015 (8)
C16	0.0143 (11)	0.0108 (9)	0.0131 (10)	0.0012 (8)	0.0034 (9)	-0.0003 (8)
C17	0.0162 (10)	0.0122 (9)	0.0136 (10)	0.0008 (8)	0.0045 (8)	-0.0002 (7)
C18	0.0206 (12)	0.0184 (11)	0.0223 (12)	-0.0009 (9)	0.0102 (10)	-0.0016 (9)
C19	0.0159 (12)	0.0252 (13)	0.0312 (14)	0.0017 (9)	0.0111 (11)	-0.0049 (11)
C20	0.0200 (13)	0.0204 (12)	0.0295 (14)	0.0094 (10)	0.0023 (11)	0.0007 (10)
C21	0.0228 (12)	0.0114 (10)	0.0200 (11)	0.0046 (8)	0.0061 (10)	0.0022 (8)

C22	0.0156 (11)	0.0140 (10)	0.0168 (10)	-0.0017 (8)	0.0033 (9)	0.0011 (8)
C23	0.0175 (11)	0.0153 (10)	0.0166 (11)	0.0004 (8)	0.0062 (9)	-0.0031 (8)
C24	0.0207 (10)	0.0127 (13)	0.0222 (10)	-0.0027 (9)	0.0057 (8)	0.0002 (9)
I2	0.01314 (6)	0.01896 (7)	0.02322 (7)	0.00158 (8)	0.00583 (5)	0.00077 (9)
O2	0.0140 (7)	0.0138 (7)	0.0118 (7)	0.0013 (6)	-0.0009 (6)	0.0021 (6)
N2	0.0264 (11)	0.0231 (10)	0.0063 (8)	0.0001 (8)	0.0049 (8)	0.0011 (7)
C25	0.0099 (9)	0.0103 (9)	0.0096 (9)	0.0018 (6)	0.0036 (7)	0.0006 (7)
C26	0.0109 (9)	0.0116 (9)	0.0108 (9)	0.0019 (7)	0.0024 (7)	0.0013 (7)
C27	0.0131 (10)	0.0141 (10)	0.0181 (11)	0.0049 (8)	0.0059 (9)	0.0044 (8)
C28	0.0098 (10)	0.0212 (11)	0.0180 (11)	0.0047 (8)	0.0014 (8)	0.0066 (9)
C29	0.0089 (9)	0.0203 (12)	0.0138 (10)	0.0009 (8)	0.0005 (8)	0.0042 (8)
C30	0.0105 (9)	0.0127 (9)	0.0106 (9)	0.0001 (7)	0.0018 (7)	0.0010 (7)
C31	0.0152 (10)	0.0133 (10)	0.0130 (9)	-0.0020 (8)	0.0032 (8)	0.0006 (7)
C32	0.0125 (10)	0.0142 (9)	0.0086 (9)	-0.0015 (7)	0.0010 (7)	0.0004 (7)
C33	0.0206 (15)	0.0181 (11)	0.0108 (12)	-0.0006 (10)	0.0023 (11)	0.0014 (9)
C34	0.0204 (11)	0.0158 (10)	0.0114 (9)	-0.0048 (8)	0.0070 (9)	-0.0034 (8)
C35	0.0152 (11)	0.0121 (10)	0.0100 (9)	-0.0027 (8)	0.0044 (8)	-0.0019 (7)
C36	0.0138 (10)	0.0135 (9)	0.0116 (9)	-0.0019 (7)	0.0054 (8)	-0.0020 (7)
C37	0.0141 (10)	0.0121 (9)	0.0170 (10)	-0.0022 (7)	0.0046 (8)	-0.0018 (7)
C38	0.0195 (12)	0.0180 (11)	0.0215 (12)	-0.0025 (9)	0.0117 (10)	-0.0051 (9)
C39	0.0275	0.0181	0.0140	-0.0035	0.0127	-0.0039

	(13)	(11)	(10)	(10)	(10)	(8)
C40	0.0126 (10)	0.0103 (9)	0.0129 (9)	-0.0010 (7)	0.0043 (8)	-0.0009 (7)
C41	0.0172 (12)	0.0145 (11)	0.0188 (11)	0.0019 (9)	0.0057 (9)	0.0039 (9)
C42	0.0240 (13)	0.0185 (11)	0.0183 (11)	-0.0049 (9)	0.0073 (10)	0.0047 (9)
C43	0.0226 (12)	0.0253 (12)	0.0200 (12)	-0.0083 (10)	0.0136 (10)	-0.0034 (9)
C44	0.0137 (11)	0.0194 (11)	0.0236 (12)	-0.0031 (8)	0.0082 (9)	-0.0054 (9)
C45	0.0112 (12)	0.0129 (10)	0.0153 (13)	-0.0005 (8)	0.0046 (10)	-0.0013 (9)
C46	0.0150 (10)	0.0132 (10)	0.0149 (10)	-0.0004 (8)	0.0052 (8)	-0.0023 (8)
C47	0.0149 (10)	0.0135 (9)	0.0154 (10)	-0.0029 (8)	0.0064 (8)	-0.0016 (8)
C48	0.0163 (9)	0.0126 (12)	0.0200 (9)	-0.0001 (8)	0.0048 (8)	-0.0034 (8)

Geometric parameters (Å, º) for (SCP1160)

I1—C13	2.108 (2)	I2—C37	2.107 (2)
O1—C1	1.220 (3)	O2—C25	1.220 (3)
N1—C9	1.367 (4)	N2—C34	1.363 (3)
N1—C10	1.370 (3)	N2—C33	1.375 (4)
N1—H1N	0.80 (2)	N2—H2N	0.80 (2)
C1—C2	1.549 (3)	C25—C30	1.543 (3)
C1—C6	1.550 (3)	C25—C26	1.545 (3)
C2—C16	1.541 (4)	C26—C40	1.537 (3)
C2—C3	1.543 (4)	C26—C27	1.547 (3)
C2—C22	1.560 (4)	C26—C46	1.552 (3)
C3—C4	1.516 (4)	C27—C28	1.527 (4)
C3—H3A	0.9900	C27—H27A	0.9900
C3—H3B	0.9900	C27—H27B	0.9900
C4—C5	1.529 (3)	C28—C29	1.526 (3)
C4—H4A	0.9900	C28—H28A	0.9900

C4—H4B	0.9900	C28—H28B	0.9900
C5—C6	1.544 (3)	C29—C30	1.545 (3)
C5—H5A	0.9900	C29—H29A	0.9900
C5—H5B	0.9900	C29—H29B	0.9900
C6—C8	1.519 (3)	C30—C32	1.518 (3)
C6—C7	1.536 (3)	C30—C31	1.532 (3)
C7—H7A	0.9800	C31—H31A	0.9800
C7—H7B	0.9800	C31—H31B	0.9800
C7—H7C	0.9800	C31—H31C	0.9800
C8—C9	1.367 (3)	C32—C33	1.372 (4)
C8—C11	1.447 (3)	C32—C35	1.442 (3)
C9—H9	0.9500	C33—H33	0.9500
C10—C15	1.392 (4)	C34—C39	1.396 (4)
C10—C11	1.422 (3)	C34—C35	1.424 (3)
C11—C12	1.409 (3)	C35—C36	1.407 (3)
C12—C13	1.382 (3)	C36—C37	1.383 (3)
C12—H12	0.9500	C36—H36	0.9500
C13—C14	1.407 (3)	C37—C38	1.414 (3)
C14—C15	1.380 (3)	C38—C39	1.376 (4)
C14—H14	0.9500	C38—H38	0.9500
C15—H15	0.9500	C39—H39	0.9500
C16—C21	1.391 (3)	C40—C41	1.395 (3)
C16—C17	1.400 (3)	C40—C45	1.398 (4)
C17—C18	1.384 (3)	C41—C42	1.390 (4)
C17—H17	0.9500	C41—H41	0.9500
C18—C19	1.391 (4)	C42—C43	1.387 (4)
C18—H18	0.9500	C42—H42	0.9500
C19—C20	1.390 (4)	C43—C44	1.390 (4)
C19—H19	0.9500	C43—H43	0.9500
C20—C21	1.391 (4)	C44—C45	1.390 (4)
C20—H20	0.9500	C44—H44	0.9500
C21—H21	0.9500	C45—H45	0.9500
C22—C23	1.501 (3)	C46—C47	1.507 (3)

C22—H22A	0.9900	C46—H46A	0.9900
C22—H22B	0.9900	C46—H46B	0.9900
C23—C24	1.318 (3)	C47—C48	1.319 (3)
C23—H23	0.9500	C47—H47	0.9500
C24—H24A	0.9500	C48—H48A	0.9500
C24—H24B	0.9500	C48—H48B	0.9500
C9—N1—C10	108.9 (2)	C34—N2—C33	109.2 (2)
C9—N1—H1N	123 (2)	C34—N2—H2N	129 (2)
C10—N1—H1N	128 (3)	C33—N2—H2N	121 (2)
O1—C1—C2	120.6 (2)	O2—C25—C30	119.78 (19)
O1—C1—C6	119.54 (19)	O2—C25—C26	120.38 (19)
C2—C1—C6	119.85 (19)	C30—C25—C26	119.83 (17)
C16—C2—C3	113.4 (2)	C40—C26—C25	109.95 (17)
C16—C2—C1	108.56 (19)	C40—C26—C27	113.59 (18)
C3—C2—C1	108.3 (2)	C25—C26—C27	107.29 (18)
C16—C2—C22	109.5 (2)	C40—C26—C46	108.39 (18)
C3—C2—C22	109.2 (2)	C25—C26—C46	108.52 (17)
C1—C2—C22	107.7 (2)	C27—C26—C46	108.99 (18)
C4—C3—C2	113.5 (2)	C28—C27—C26	113.83 (19)
C4—C3—H3A	108.9	C28—C27—H27A	108.8
C2—C3—H3A	108.9	C26—C27—H27A	108.8
C4—C3—H3B	108.9	C28—C27—H27B	108.8
C2—C3—H3B	108.9	C26—C27—H27B	108.8
H3A—C3—H3B	107.7	H27A—C27—H27B	107.7
C3—C4—C5	109.9 (2)	C29—C28—C27	110.49 (19)
C3—C4—H4A	109.7	C29—C28—H28A	109.6
C5—C4—H4A	109.7	C27—C28—H28A	109.6
C3—C4—H4B	109.7	C29—C28—H28B	109.6
C5—C4—H4B	109.7	C27—C28—H28B	109.6
H4A—C4—H4B	108.2	H28A—C28—H28B	108.1
C4—C5—C6	114.22 (19)	C28—C29—C30	115.42 (19)

C4—C5—H5A	108.7	C28—C29—H29A	108.4
C6—C5—H5A	108.7	C30—C29—H29A	108.4
C4—C5—H5B	108.7	C28—C29—H29B	108.4
C6—C5—H5B	108.7	C30—C29—H29B	108.4
H5A—C5—H5B	107.6	H29A—C29—H29B	107.5
C8—C6—C7	110.03 (19)	C32—C30—C31	109.71 (19)
C8—C6—C5	111.40 (19)	C32—C30—C25	107.21 (18)
C7—C6—C5	108.51 (18)	C31—C30—C25	110.10 (17)
C8—C6—C1	107.50 (17)	C32—C30—C29	112.56 (18)
C7—C6—C1	109.48 (18)	C31—C30—C29	108.04 (18)
C5—C6—C1	109.91 (18)	C25—C30—C29	109.22 (18)
C6—C7—H7A	109.5	C30—C31—H31A	109.5
C6—C7—H7B	109.5	C30—C31—H31B	109.5
H7A—C7—H7B	109.5	H31A—C31—H31B	109.5
C6—C7—H7C	109.5	C30—C31—H31C	109.5
H7A—C7—H7C	109.5	H31A—C31—H31C	109.5
H7B—C7—H7C	109.5	H31B—C31—H31C	109.5
C9—C8—C11	105.9 (2)	C33—C32—C35	106.1 (2)
C9—C8—C6	126.4 (2)	C33—C32—C30	126.6 (2)
C11—C8—C6	127.71 (19)	C35—C32—C30	127.31 (19)
N1—C9—C8	110.9 (2)	C32—C33—N2	110.3 (3)
N1—C9—H9	124.6	C32—C33—H33	124.8
C8—C9—H9	124.6	N2—C33—H33	124.8
N1—C10—C15	129.8 (2)	N2—C34—C39	129.7 (2)
N1—C10—C11	107.7 (2)	N2—C34—C35	107.8 (2)
C15—C10—C11	122.5 (2)	C39—C34—C35	122.5 (2)
C12—C11—C10	118.5 (2)	C36—C35—C34	118.4 (2)
C12—C11—C8	135.0 (2)	C36—C35—C32	134.9 (2)
C10—C11—C8	106.51 (19)	C34—C35—C32	106.6 (2)
C13—C12—C11	118.2 (2)	C37—C36—C35	118.4 (2)
C13—C12—H12	120.9	C37—C36—H36	120.8

C11—C12—H12	120.9	C35—C36—H36	120.8
C12—C13—C14	122.6 (2)	C36—C37—C38	122.5 (2)
C12—C13—I1	119.68 (16)	C36—C37—I2	119.40 (17)
C14—C13—I1	117.71 (16)	C38—C37—I2	118.10 (18)
C15—C14—C13	120.0 (2)	C39—C38—C37	120.0 (2)
C15—C14—H14	120.0	C39—C38—H38	120.0
C13—C14—H14	120.0	C37—C38—H38	120.0
C14—C15—C10	118.2 (2)	C38—C39—C34	118.2 (2)
C14—C15—H15	120.9	C38—C39—H39	120.9
C10—C15—H15	120.9	C34—C39—H39	120.9
C21—C16—C17	117.8 (2)	C41—C40—C45	117.9 (2)
C21—C16—C2	123.5 (2)	C41—C40—C26	122.9 (2)
C17—C16—C2	118.6 (2)	C45—C40—C26	119.2 (2)
C18—C17—C16	122.0 (2)	C42—C41—C40	120.9 (2)
C18—C17—H17	119.0	C42—C41—H41	119.6
C16—C17—H17	119.0	C40—C41—H41	119.6
C17—C18—C19	119.5 (2)	C43—C42—C41	120.6 (2)
C17—C18—H18	120.2	C43—C42—H42	119.7
C19—C18—H18	120.2	C41—C42—H42	119.7
C20—C19—C18	119.2 (2)	C42—C43—C44	119.2 (2)
C20—C19—H19	120.4	C42—C43—H43	120.4
C18—C19—H19	120.4	C44—C43—H43	120.4
C19—C20—C21	120.9 (2)	C45—C44—C43	120.0 (2)
C19—C20—H20	119.5	C45—C44—H44	120.0
C21—C20—H20	119.5	C43—C44—H44	120.0
C16—C21—C20	120.5 (2)	C44—C45—C40	121.3 (3)
C16—C21—H21	119.8	C44—C45—H45	119.3
C20—C21—H21	119.8	C40—C45—H45	119.3
C23—C22—C2	112.8 (2)	C47—C46—C26	112.86 (19)
C23—C22—H22A	109.0	C47—C46—H46A	109.0
C2—C22—H22A	109.0	C26—C46—H46A	109.0
C23—C22—H22B	109.0	C47—C46—H46B	109.0
C2—C22—H22B	109.0	C26—C46—H46B	109.0

H22A—C22—H22B	107.8	H46A—C46—H46B	107.8
C24—C23—C22	124.6 (2)	C48—C47—C46	124.1 (2)
C24—C23—H23	117.7	C48—C47—H47	117.9
C22—C23—H23	117.7	C46—C47—H47	117.9
C23—C24—H24A	120.0	C47—C48—H48A	120.0
C23—C24—H24B	120.0	C47—C48—H48B	120.0
H24A—C24—H24B	120.0	H48A—C48—H48B	120.0
O1—C1—C2—C16	101.1 (3)	O2—C25—C26—C40	-106.5 (2)
C6—C1—C2—C16	-79.0 (3)	C30—C25—C26—C40	75.2 (2)
O1—C1—C2—C3	-135.4 (2)	O2—C25—C26—C27	129.6 (2)
C6—C1—C2—C3	44.5 (3)	C30—C25—C26—C27	-48.8 (3)
O1—C1—C2—C22	-17.3 (3)	O2—C25—C26—C46	11.9 (3)
C6—C1—C2—C22	162.57 (19)	C30—C25—C26—C46	-166.42 (19)
C16—C2—C3—C4	67.9 (3)	C40—C26—C27—C28	-68.2 (3)
C1—C2—C3—C4	-52.7 (3)	C25—C26—C27—C28	53.6 (2)
C22—C2—C3—C4	-169.8 (2)	C46—C26—C27—C28	170.87 (19)
C2—C3—C4—C5	60.7 (3)	C26—C27—C28—C29	-58.0 (3)
C3—C4—C5—C6	-57.3 (3)	C27—C28—C29—C30	53.8 (3)
C4—C5—C6—C8	-72.5 (3)	O2—C25—C30—C32	104.6 (2)
C4—C5—C6—C7	166.3 (2)	C26—C25—C30—C32	-77.1 (2)
C4—C5—C6—C1	46.6 (3)	O2—C25—C30—C31	-14.7 (3)

O1—C1—C6—C8	-100.5 (2)	C26—C25—C30—C31	163.64 (19)
C2—C1—C6—C8	79.5 (2)	O2—C25—C30—C29	-133.2 (2)
O1—C1—C6—C7	19.0 (3)	C26—C25—C30—C29	45.2 (3)
C2—C1—C6—C7	-161.0 (2)	C28—C29—C30—C32	73.1 (3)
O1—C1—C6—C5	138.0 (2)	C28—C29—C30—C31	-165.6 (2)
C2—C1—C6—C5	-41.9 (3)	C28—C29—C30—C25	-45.9 (3)
C7—C6—C8—C9	117.6 (3)	C31—C30—C32—C33	-120.5 (3)
C5—C6—C8—C9	-2.8 (3)	C25—C30—C32—C33	119.9 (3)
C1—C6—C8—C9	-123.2 (2)	C29—C30—C32—C33	-0.2 (3)
C7—C6—C8—C11	-61.9 (3)	C31—C30—C32—C35	60.3 (3)
C5—C6—C8—C11	177.7 (2)	C25—C30—C32—C35	-59.2 (3)
C1—C6—C8—C11	57.3 (3)	C29—C30—C32—C35	-179.3 (2)
C10—N1—C9—C8	0.2 (3)	C35—C32—C33—N2	0.7 (3)
C11—C8—C9—N1	-1.1 (3)	C30—C32—C33—N2	-178.6 (2)
C6—C8—C9—N1	179.3 (2)	C34—N2—C33—C32	0.1 (3)
C9—N1—C10—C15	-177.9 (3)	C33—N2—C34—C39	175.6 (3)
C9—N1—C10—C11	0.8 (3)	C33—N2—C34—C35	-0.9 (3)
N1—C10—C11—C12	180.0 (2)	N2—C34—C35—C36	179.0 (2)
C15—C10—C11—C12	-1.2 (3)	C39—C34—C35—C36	2.1 (4)
N1—C10—C11—	-1.5 (3)	N2—C34—C35—	1.3 (3)

C8		C32	
C15—C10—C11—C8	177.4 (2)	C39—C34—C35—C32	-175.5 (2)
C9—C8—C11—C12	179.8 (3)	C33—C32—C35—C36	-178.3 (3)
C6—C8—C11—C12	-0.7 (4)	C30—C32—C35—C36	1.0 (4)
C9—C8—C11—C10	1.5 (3)	C33—C32—C35—C34	-1.2 (3)
C6—C8—C11—C10	-178.9 (2)	C30—C32—C35—C34	178.0 (2)
C10—C11—C12—C13	1.3 (3)	C34—C35—C36—C37	-0.9 (3)
C8—C11—C12—C13	-176.8 (2)	C32—C35—C36—C37	175.9 (3)
C11—C12—C13—C14	-0.8 (3)	C35—C36—C37—C38	-0.8 (3)
C11—C12—C13—I1	179.17 (16)	C35—C36—C37—I2	178.01 (17)
C12—C13—C14—C15	0.0 (3)	C36—C37—C38—C39	1.3 (4)
I1—C13—C14—C15	-179.90 (17)	I2—C37—C38—C39	-177.53 (19)
C13—C14—C15—C10	0.1 (3)	C37—C38—C39—C34	0.0 (4)
N1—C10—C15—C14	179.0 (2)	N2—C34—C39—C38	-177.7 (3)
C11—C10—C15—C14	0.4 (4)	C35—C34—C39—C38	-1.7 (4)
C3—C2—C16—C21	16.2 (3)	C25—C26—C40—C41	-134.6 (2)
C1—C2—C16—C21	136.6 (2)	C27—C26—C40—C41	-14.4 (3)
C22—C2—C16—C21	-106.0 (3)	C46—C26—C40—C41	106.9 (2)
C3—C2—C16—C17	-167.1 (2)	C25—C26—C40—C45	48.3 (3)
C1—C2—C16—C17	-46.6 (3)	C27—C26—C40—C45	168.6 (2)

C22—C2—C16—C17	70.7 (3)	C46—C26—C40—C45	-70.1 (3)
C21—C16—C17—C18	-1.2 (4)	C45—C40—C41—C42	-1.5 (4)
C2—C16—C17—C18	-178.1 (2)	C26—C40—C41—C42	-178.6 (2)
C16—C17—C18—C19	0.0 (4)	C40—C41—C42—C43	0.6 (4)
C17—C18—C19—C20	0.6 (4)	C41—C42—C43—C44	0.1 (4)
C18—C19—C20—C21	0.0 (4)	C42—C43—C44—C45	0.3 (4)
C17—C16—C21—C20	1.8 (4)	C43—C44—C45—C40	-1.2 (4)
C2—C16—C21—C20	178.5 (2)	C41—C40—C45—C44	1.8 (4)
C19—C20—C21—C16	-1.2 (4)	C26—C40—C45—C44	179.0 (2)
C16—C2—C22—C23	72.5 (3)	C40—C26—C46—C47	-67.9 (2)
C3—C2—C22—C23	-52.2 (3)	C25—C26—C46—C47	172.70 (19)
C1—C2—C22—C23	-169.7 (2)	C27—C26—C46—C47	56.2 (3)
C2—C22—C23—C24	127.0 (3)	C26—C46—C47—C48	-132.4 (2)

Hydrogen-bond geometry (Å, º) for (SCP1160)

D—H···*A*	*D*—H	H···*A*	*D*···*A*	*D*—H···*A*
N1—H1*N*···O2^{i}	0.80 (2)	2.11 (2)	2.873 (3)	158 (3)
C12—H12···O1	0.95	2.65	3.301 (3)	127
N2—H2*N*···O1	0.80 (2)	2.17 (2)	2.955 (3)	170 (3)
C38—H38···I1^{ii}	0.95	3.31	4.211 (3)	159

Symmetry codes: (i) *x*+1, *y*, *z*+1; (ii) *x*-1, *y*, *z*.

(±)-(2R,6R)-2-allyl-6-(7-bromo-1H-indol-3-yl)-6-methyl-2-phenylcyclohexanone (14f)

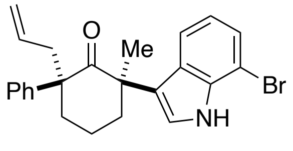

14f

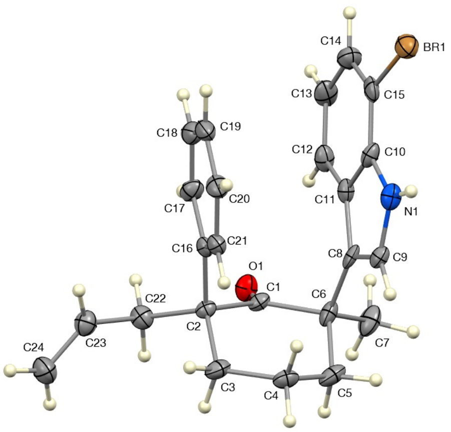

Sample Name: CCDC 2072236 / Satish9 (SCP1180)

Crystal data

$C_{24}H_{24}BrNO$	$F(000) = 436$
$M_r = 422.35$	$D_x = 1.377$ Mg m^{-3}
Monoclinic, $P2_1$	Mo $K\alpha$ radiation, $\lambda = 0.71073$ Å
$a = 8.0027$ (17) Å	Cell parameters from 2056 reflections
$b = 10.497$ (2) Å	$\theta = 2.6–20.0°$
$c = 12.356$ (3) Å	$\mu = 2.03$ mm^{-1}
$\beta = 101.045$ (5)°	$T = 90$ K
$V = 1018.8$ (4) Å^3	Fragment, colourless
$Z = 2$	0.12 × 0.12 × 0.10 mm

Data collection

Bruker Kappa APEX-II DUO diffractometer	5255 independent reflections
Radiation source: fine-focus sealed tube	3371 reflections with $I > 2\sigma(I)$
TRIUMPH curved graphite monochromator	$R_{int} = 0.106$
ϕ and ω scans	$\theta_{max} = 28.7°$, $\theta_{min} = 1.7°$
Absorption correction: multi-scan *SADABS* (Sheldrick, 2004)	$h = -10 – 10$

T_{min} = 0.745, T_{max} = 0.823	k = -14 – 14
17401 measured reflections	l = -16 – 16

Refinement

Refinement on F^2	Hydrogen site location: mixed
Least-squares matrix: full	H atoms treated by a mixture of independent and constrained refinement
$R[F^2 > 2\sigma(F^2)]$ = 0.053	$w = 1/[\sigma^2(F_o^2)]$ where $P = (F_o^2 + 2F_c^2)/3$
$wR(F^2)$ = 0.092	$(\Delta/\sigma)_{max}$ < 0.001
S = 0.97	$\Delta\rho_{max}$ = 0.42 e Å^{-3}
5255 reflections	$\Delta\rho_{min}$ = -0.55 e Å^{-3}
248 parameters	Absolute structure: Flack x determined using 1112 quotients [(I+)-(I-)]/[(I+)+(I-)] (Parsons, Flack and Wagner, Acta Cryst. B69 (2013) 249-259).
2 restraints	Absolute structure parameter: 0.005 (10)

Fractional atomic coordinates and isotropic or equivalent isotropic displacement parameters (Å^2) for (SCP1180)

	x	y	z	U_{iso}*/U_{eq}
Br1	0.08689 (7)	0.79180 (8)	0.18579 (5)	0.02932 (17)
O1	0.9713 (5)	0.5143 (4)	0.3712 (3)	0.0260 (10)
N1	0.2938 (6)	0.5643 (5)	0.3288 (4)	0.0220 (12)
H1N	0.190 (4)	0.552 (6)	0.325 (5)	0.026*
C1	0.8470 (7)	0.4447 (6)	0.3614 (4)	0.0180 (13)
C2	0.8093 (7)	0.3476 (6)	0.2659 (5)	0.0166 (14)
C3	0.7588 (8)	0.2178 (7)	0.3094 (5)	0.0249 (16)
H3A	0.708045	0.164441	0.245518	0.030*
H3B	0.863641	0.174528	0.347500	0.030*
C4	0.6338 (7)	0.2243 (6)	0.3885 (5)	0.0235 (15)
H4A	0.521025	0.252060	0.347530	0.028*
H4B	0.620559	0.138303	0.418680	0.028*

C5	0.6944 (7)	0.3160 (7)	0.4826 (4)	0.0263 (18)
H5A	0.799990	0.281673	0.528259	0.032*
H5B	0.607193	0.320055	0.529516	0.032*
C6	0.7296 (7)	0.4522 (6)	0.4462 (5)	0.0214 (14)
C7	0.8183 (8)	0.5285 (7)	0.5481 (5)	0.0320 (17)
H7A	0.834016	0.616903	0.526597	0.048*
H7B	0.929527	0.490332	0.577504	0.048*
H7C	0.747754	0.526612	0.604681	0.048*
C8	0.5690 (7)	0.5210 (6)	0.3926 (4)	0.0173 (13)
C9	0.4059 (7)	0.4803 (6)	0.3870 (4)	0.0185 (13)
H9	0.374685	0.403845	0.419185	0.022*
C10	0.3829 (7)	0.6636 (6)	0.2952 (5)	0.0206 (14)
C11	0.5588 (7)	0.6424 (6)	0.3363 (5)	0.0192 (14)
C12	0.6751 (8)	0.7348 (6)	0.3165 (5)	0.0241 (15)
H12	0.793384	0.724743	0.344642	0.029*
C13	0.6152 (9)	0.8404 (6)	0.2558 (5)	0.0299 (17)
H13	0.694413	0.902579	0.241513	0.036*
C14	0.4406 (9)	0.8599 (6)	0.2138 (5)	0.0303 (16)
H14	0.403375	0.933955	0.171745	0.036*
C15	0.3250 (7)	0.7711 (7)	0.2341 (4)	0.0240 (17)
C16	0.6704 (7)	0.4083 (5)	0.1768 (5)	0.0181 (13)
C17	0.7057 (8)	0.5226 (6)	0.1287 (5)	0.0241 (14)
H17	0.815255	0.559825	0.149794	0.029*
C18	0.5844 (8)	0.5830 (7)	0.0509 (5)	0.0291 (17)
H18	0.611539	0.661007	0.019272	0.035*
C19	0.4244 (8)	0.5310 (6)	0.0187 (5)	0.0290 (17)
H19	0.340735	0.573152	-0.034082	0.035*
C20	0.3872 (8)	0.4159 (6)	0.0646 (5)	0.0271 (16)
H20	0.277349	0.379274	0.042769	0.033*
C21	0.5095 (7)	0.3537 (6)	0.1423 (4)	0.0205 (14)
H21	0.483458	0.274231	0.171897	0.025*
C22	0.9749 (7)	0.3291 (5)	0.2192 (5)	0.0240 (16)
H22A	1.012221	0.413049	0.195967	0.029*

H22B	1.065620	0.296597	0.278727	0.029*
C23	0.9553 (8)	0.2397 (6)	0.1235 (5)	0.0265 (15)
H23	0.867836	0.257493	0.061761	0.032*
C24	1.0498 (9)	0.1379 (6)	0.1182 (6)	0.0355 (18)
H24A	1.138482	0.116885	0.178348	0.043*
H24B	1.029197	0.085643	0.054322	0.043*

Atomic displacement parameters (Å^2) for (SCP1180)

	U^{11}	U^{22}	U^{33}	U^{12}	U^{13}	U^{23}
Br1	0.0295 (3)	0.0332 (3)	0.0248 (3)	0.0049 (5)	0.0041 (2)	-0.0009 (5)
O1	0.023 (2)	0.030 (3)	0.026 (2)	-0.006 (2)	0.0072 (19)	-0.003 (2)
N1	0.016 (3)	0.029 (3)	0.021 (3)	-0.007 (3)	0.004 (2)	-0.005 (2)
C1	0.020 (3)	0.020 (3)	0.013 (3)	-0.002 (3)	0.001 (3)	0.003 (3)
C2	0.016 (3)	0.017 (3)	0.015 (3)	-0.002 (3)	0.001 (2)	0.001 (3)
C3	0.023 (4)	0.029 (4)	0.022 (4)	0.000 (3)	0.001 (3)	0.010 (3)
C4	0.025 (4)	0.021 (3)	0.024 (3)	-0.003 (3)	0.003 (3)	0.007 (3)
C5	0.024 (3)	0.035 (5)	0.019 (3)	-0.005 (3)	0.001 (2)	0.011 (3)
C6	0.019 (3)	0.034 (4)	0.013 (3)	-0.005 (3)	0.006 (3)	-0.001 (3)
C7	0.029 (4)	0.049 (5)	0.017 (3)	-0.006 (3)	0.003 (3)	-0.004 (3)
C8	0.019 (3)	0.027 (4)	0.007 (3)	-0.008 (3)	0.005 (2)	-0.002 (3)
C9	0.020 (3)	0.023 (3)	0.013 (3)	-0.005 (3)	0.004 (2)	-0.001 (3)
C10	0.022 (4)	0.026 (4)	0.016 (3)	-0.008 (3)	0.007 (3)	-0.001 (3)
C11	0.019 (3)	0.022 (3)	0.018 (3)	-0.003 (3)	0.007 (3)	-0.006 (3)
C12	0.024 (4)	0.022 (3)	0.029 (4)	-0.006 (3)	0.012 (3)	-0.007 (3)
C13	0.034 (4)	0.021 (4)	0.041 (4)	-0.008 (3)	0.021 (3)	0.000 (3)
C14	0.042 (4)	0.023 (4)	0.028 (4)	0.001 (3)	0.012 (3)	0.003 (3)
C15	0.028 (3)	0.028 (5)	0.019 (3)	0.005 (3)	0.008 (2)	-0.010 (3)
C16	0.023 (3)	0.015 (3)	0.017 (3)	0.003 (3)	0.006 (3)	-0.003 (3)
C17	0.032 (4)	0.019 (3)	0.022 (3)	-0.006 (3)	0.006 (3)	0.000 (3)
C18	0.040 (5)	0.027 (4)	0.021 (4)	0.006 (3)	0.006 (3)	0.000 (3)
C19	0.036 (4)	0.029 (4)	0.022 (4)	0.015 (4)	0.006 (3)	0.000 (3)
C20	0.026 (4)	0.033 (4)	0.020 (3)	0.007 (3)	-0.001 (3)	-0.002 (3)

C21	0.023 (3)	0.018 (3)	0.020 (3)	0.003 (3)	0.006 (3)	-0.001 (3)
C22	0.022 (3)	0.024 (4)	0.025 (3)	0.000 (3)	0.003 (3)	-0.003 (3)
C23	0.024 (3)	0.030 (4)	0.025 (3)	-0.002 (3)	0.006 (3)	-0.001 (3)
C24	0.043 (4)	0.030 (4)	0.035 (4)	0.007 (4)	0.010 (3)	-0.004 (3)

Geometric parameters (Å, º) for (SCP1180)

Br1—C15	1.897 (6)	C10—C15	1.387 (9)
O1—C1	1.221 (7)	C10—C11	1.420 (8)
N1—C9	1.361 (7)	C11—C12	1.398 (9)
N1—C10	1.371 (8)	C12—C13	1.372 (8)
N1—H1N	0.83 (3)	C12—H12	0.9500
C1—C6	1.538 (8)	C13—C14	1.409 (9)
C1—C2	1.545 (8)	C13—H13	0.9500
C2—C16	1.544 (8)	C14—C15	1.370 (9)
C2—C3	1.546 (8)	C14—H14	0.9500
C2—C22	1.556 (8)	C16—C17	1.391 (8)
C3—C4	1.528 (9)	C16—C21	1.399 (8)
C3—H3A	0.9900	C17—C18	1.383 (8)
C3—H3B	0.9900	C17—H17	0.9500
C4—C5	1.516 (8)	C18—C19	1.378 (8)
C4—H4A	0.9900	C18—H18	0.9500
C4—H4B	0.9900	C19—C20	1.391 (9)
C5—C6	1.541 (9)	C19—H19	0.9500
C5—H5A	0.9900	C20—C21	1.395 (8)
C5—H5B	0.9900	C20—H20	0.9500
C6—C8	1.512 (8)	C21—H21	0.9500
C6—C7	1.545 (8)	C22—C23	1.494 (8)
C7—H7A	0.9800	C22—H22A	0.9900
C7—H7B	0.9800	C22—H22B	0.9900
C7—H7C	0.9800	C23—C24	1.317 (8)
C8—C9	1.363 (7)	C23—H23	0.9500
C8—C11	1.447 (8)	C24—H24A	0.9500
C9—H9	0.9500	C24—H24B	0.9500

C9—N1—C10	108.9 (5)	N1—C10—C15	130.1 (6)
C9—N1—H1N	119 (4)	N1—C10—C11	108.1 (5)
C10—N1—H1N	132 (4)	C15—C10—C11	121.8 (6)
O1—C1—C6	119.6 (5)	C12—C11—C10	118.4 (6)
O1—C1—C2	120.5 (5)	C12—C11—C8	135.9 (6)
C6—C1—C2	119.9 (5)	C10—C11—C8	105.7 (5)
C16—C2—C1	106.3 (5)	C13—C12—C11	118.8 (6)
C16—C2—C3	114.3 (5)	C13—C12—H12	120.6
C1—C2—C3	110.0 (5)	C11—C12—H12	120.6
C16—C2—C22	109.6 (5)	C12—C13—C14	122.5 (6)
C1—C2—C22	108.0 (5)	C12—C13—H13	118.8
C3—C2—C22	108.5 (4)	C14—C13—H13	118.8
C4—C3—C2	115.5 (5)	C15—C14—C13	119.3 (6)
C4—C3—H3A	108.4	C15—C14—H14	120.4
C2—C3—H3A	108.4	C13—C14—H14	120.4
C4—C3—H3B	108.4	C14—C15—C10	119.2 (6)
C2—C3—H3B	108.4	C14—C15—Br1	122.5 (5)
H3A—C3—H3B	107.5	C10—C15—Br1	118.4 (5)
C5—C4—C3	111.5 (5)	C17—C16—C21	118.2 (5)
C5—C4—H4A	109.3	C17—C16—C2	118.9 (5)
C3—C4—H4A	109.3	C21—C16—C2	122.9 (5)
C5—C4—H4B	109.3	C18—C17—C16	121.3 (6)
C3—C4—H4B	109.3	C18—C17—H17	119.3
H4A—C4—H4B	108.0	C16—C17—H17	119.3
C4—C5—C6	114.5 (5)	C19—C18—C17	120.5 (6)
C4—C5—H5A	108.6	C19—C18—H18	119.7
C6—C5—H5A	108.6	C17—C18—H18	119.7
C4—C5—H5B	108.6	C18—C19—C20	119.1 (6)
C6—C5—H5B	108.6	C18—C19—H19	120.5
H5A—C5—H5B	107.6	C20—C19—H19	120.5
C8—C6—C1	107.8 (5)	C19—C20—C21	120.7 (6)
C8—C6—C5	112.5 (5)	C19—C20—H20	119.7

C1—C6—C5	108.7 (5)	C21—C20—H20	119.7
C8—C6—C7	109.2 (5)	C20—C21—C16	120.1 (6)
C1—C6—C7	109.6 (5)	C20—C21—H21	120.0
C5—C6—C7	109.0 (5)	C16—C21—H21	120.0
C6—C7—H7A	109.5	C23—C22—C2	113.8 (5)
C6—C7—H7B	109.5	C23—C22—H22A	108.8
H7A—C7—H7B	109.5	C2—C22—H22A	108.8
C6—C7—H7C	109.5	C23—C22—H22B	108.8
H7A—C7—H7C	109.5	C2—C22—H22B	108.8
H7B—C7—H7C	109.5	H22A—C22—H22B	107.7
C9—C8—C11	106.6 (5)	C24—C23—C22	125.0 (6)
C9—C8—C6	126.8 (5)	C24—C23—H23	117.5
C11—C8—C6	126.6 (5)	C22—C23—H23	117.5
N1—C9—C8	110.6 (5)	C23—C24—H24A	120.0
N1—C9—H9	124.7	C23—C24—H24B	120.0
C8—C9—H9	124.7	H24A—C24—H24B	120.0
O1—C1—C2—C16	-99.9 (6)	C15—C10—C11—C8	-178.7 (5)
C6—C1—C2—C16	81.3 (6)	C9—C8—C11—C12	176.9 (7)
O1—C1—C2—C3	135.8 (6)	C6—C8—C11—C12	-3.6 (11)
C6—C1—C2—C3	-42.9 (7)	C9—C8—C11—C10	-2.6 (6)
O1—C1—C2—C22	17.6 (7)	C6—C8—C11—C10	176.9 (5)
C6—C1—C2—C22	-161.2 (5)	C10—C11—C12—C13	-1.8 (9)
C16—C2—C3—C4	-75.2 (6)	C8—C11—C12—C13	178.8 (6)
C1—C2—C3—C4	44.2 (7)	C11—C12—C13—C14	0.8 (9)
C22—C2—C3—C4	162.2 (5)	C12—C13—C14—C15	0.2 (9)

C2—C3—C4—C5	-52.1 (7)	C13—C14—C15—C10	-0.3 (9)
C3—C4—C5—C6	55.8 (7)	C13—C14—C15—Br1	177.6 (5)
O1—C1—C6—C8	105.0 (6)	N1—C10—C15—C14	178.0 (6)
C2—C1—C6—C8	-76.2 (7)	C11—C10—C15—C14	-0.6 (9)
O1—C1—C6—C5	-132.7 (6)	N1—C10—C15—Br1	-0.1 (9)
C2—C1—C6—C5	46.0 (6)	C11—C10—C15—Br1	-178.7 (4)
O1—C1—C6—C7	-13.7 (8)	C1—C2—C16—C17	61.2 (7)
C2—C1—C6—C7	165.1 (5)	C3—C2—C16—C17	-177.3 (5)
C4—C5—C6—C8	68.2 (7)	C22—C2—C16—C17	-55.3 (7)
C4—C5—C6—C1	-51.1 (6)	C1—C2—C16—C21	-118.5 (6)
C4—C5—C6—C7	-170.6 (5)	C3—C2—C16—C21	3.0 (8)
C1—C6—C8—C9	128.1 (6)	C22—C2—C16—C21	125.0 (6)
C5—C6—C8—C9	8.3 (8)	C21—C16—C17—C18	1.6 (9)
C7—C6—C8—C9	-112.9 (7)	C2—C16—C17—C18	-178.1 (6)
C1—C6—C8—C11	-51.2 (8)	C16—C17—C18—C19	-0.1 (10)
C5—C6—C8—C11	-171.1 (5)	C17—C18—C19—C20	-0.8 (10)
C7—C6—C8—C11	67.8 (7)	C18—C19—C20—C21	0.2 (10)
C10—N1—C9—C8	-0.3 (7)	C19—C20—C21—C16	1.4 (9)
C11—C8—C9—N1	1.8 (7)	C17—C16—C21—C20	-2.3 (8)
C6—C8—C9—N1	-177.6 (5)	C2—C16—C21—	177.5 (5)

		C20	
C9—N1—C10—C15	179.9 (6)	C16—C2—C22—C23	-63.0 (6)
C9—N1—C10—C11	-1.4 (7)	C1—C2—C22—C23	-178.4 (5)
N1—C10—C11—C12	-177.2 (5)	C3—C2—C22—C23	62.4 (6)
C15—C10—C11—C12	1.7 (9)	C2—C22—C23—C24	-124.6 (7)
N1—C10—C11—C8	2.4 (6)		

Hydrogen-bond geometry (Å, º) for (SCP1180)

D—H···*A*	*D*—H	H···*A*	*D*···*A*	*D*—H···*A*
N1—H1*N*···O1^{i}	0.83 (3)	1.99 (3)	2.780 (6)	160 (6)

Symmetry code: (i) *x*-1, *y*, *z*.

(±)-(2*R*,6*R*)-2-allyl-6-methyl-6-(1-methyl-1*H*-indol-3-yl)-2-phenylcyclohexanone (14i)

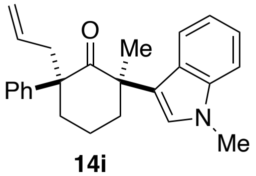

14i

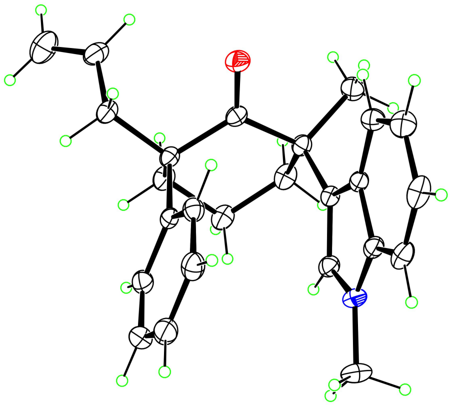

Sample Name: CCDC 2072237 / Malone9 (JAM7082)

Crystal data

$C_{25}H_{27}NO$	$F(000) = 1536$
$M_r = 357.47$	$D_x = 1.217$ Mg m^{-3}
Monoclinic, $P2_1/c$	Mo $K\alpha$ radiation, $\lambda = 0.71073$ Å
$a = 17.1415$ (6) Å	Cell parameters from 6189 reflections
$b = 16.1745$ (5) Å	$\theta = 2.3–32.3°$
$c = 14.1312$ (5) Å	$\mu = 0.07$ mm^{-1}
$\beta = 95.090$ (2)°	$T = 120$ K
$V = 3902.5$ (2) Å^3	Needle fragment, colourless
$Z = 8$	0.37 × 0.30 × 0.23 mm

Data collection

Bruker Kappa APEX-II diffractometer	14937 independent reflections
Radiation source: fine-focus sealed tube	9936 reflections with $I > 2\sigma(I)$
TRIUMPH curved graphite	$R_{int} = 0.039$
ϕ and ω scans	$\theta_{max} = 33.3°$, $\theta_{min} = 1.7°$
Absorption correction: multi-scan *SADABS* (Sheldrick, 2004)	$h = -25 – 26$
$T_{min} = 0.908$, $T_{max} = 0.983$	$k = -24 – 17$
35033 measured reflections	$l = -21 – 19$

Refinement

Refinement on F^2	0 restraints
Least-squares matrix: full	Hydrogen site location: inferred from neighbouring sites
$R[F^2 > 2\sigma(F^2)] = 0.054$	H-atom parameters constrained
$wR(F^2) = 0.144$	$w = 1/[\sigma^2(F_o^2) + (0.0696P)^2 + 0.2882P]$ where $P = (F_o^2 + 2F_c^2)/3$
$S = 1.02$	$(\Delta/\sigma)_{max} = 0.001$
14937 reflections	$\Delta\rangle_{max} = 0.44$ e Å^{-3}
491 parameters	$\Delta\rangle_{min} = -0.28$ e Å^{-3}

Fractional atomic coordinates and isotropic or equivalent isotropic displacement parameters (Å^2)

	x	*y*	*z*	U_{iso}*/U_{eq}	
O1	0.31704 (5)	0.77643 (5)	0.15354 (6)	0.02353 (18)	
N1	0.15786 (6)	0.82203 (6)	0.44207 (7)	0.02020 (19)	
C1	0.33275 (6)	0.76792 (7)	0.23863 (8)	0.0166 (2)	
C2	0.33309 (6)	0.84363 (7)	0.30592 (8)	0.0167 (2)	
C3	0.40194 (7)	0.83773 (8)	0.38394 (9)	0.0224 (2)	
H3A	0.4501	0.8565	0.3565	0.027*	
H3B	0.3919	0.8768	0.4354	0.027*	
C4	0.41762 (7)	0.75264 (8)	0.42791 (8)	0.0221 (2)	
H4A	0.4664	0.7542	0.4710	0.027*	
H4B	0.3740	0.7371	0.4658	0.027*	
C5	0.42551 (7)	0.68884 (8)	0.35057 (8)	0.0211 (2)	
H5A	0.4374	0.6344	0.3805	0.025*	
H5B	0.4701	0.7042	0.3142	0.025*	
C6	0.35108 (6)	0.68117 (7)	0.28145 (7)	0.0164 (2)	
C7	0.34414 (7)	0.92342 (7)	0.25000 (9)	0.0231 (2)	
H7A	0.3022	0.9282	0.1985	0.035*	
H7B	0.3949	0.9220	0.2232	0.035*	
H7C	0.3424	0.9711	0.2926	0.035*	

C8	0.25352 (6)	0.84337 (7)	0.34521 (8)	0.0159 (2)	
C9	0.23697 (7)	0.82247 (7)	0.43541 (8)	0.0187 (2)	
H9	0.2752	0.8100	0.4862	0.022*	
C10	0.12088 (7)	0.84277 (7)	0.35542 (8)	0.0176 (2)	
C11	0.17877 (6)	0.85707 (7)	0.29203 (8)	0.0155 (2)	
C12	0.15368 (7)	0.87756 (7)	0.19761 (8)	0.0197 (2)	
H12	0.1908	0.8863	0.1525	0.024*	
C13	0.07435 (7)	0.88479 (8)	0.17103 (9)	0.0236 (2)	
H13	0.0575	0.8997	0.1075	0.028*	
C14	0.01824 (7)	0.87069 (8)	0.23567 (9)	0.0248 (3)	
H14	-0.0358	0.8766	0.2155	0.030*	
C15	0.04072 (7)	0.84827 (7)	0.32833 (9)	0.0220 (2)	
H15	0.0030	0.8370	0.3720	0.026*	
C16	0.11904 (8)	0.79645 (9)	0.52440 (9)	0.0307 (3)	
H16A	0.1576	0.7726	0.5720	0.046*	
H16B	0.0790	0.7550	0.5053	0.046*	
H16C	0.0942	0.8445	0.5515	0.046*	
C17	0.28027 (7)	0.64877 (7)	0.32890 (8)	0.0165 (2)	
C18	0.20540 (7)	0.65968 (7)	0.28391 (8)	0.0190 (2)	
H18	0.1992	0.6884	0.2251	0.023*	
C19	0.13973 (7)	0.62951 (8)	0.32307 (9)	0.0243 (2)	
H19	0.0891	0.6390	0.2920	0.029*	
C20	0.14790 (8)	0.58561 (8)	0.40735 (10)	0.0288 (3)	
H20	0.1031	0.5646	0.4343	0.035*	
C21	0.22186 (8)	0.57271 (8)	0.45192 (9)	0.0274 (3)	
H21	0.2278	0.5422	0.5095	0.033*	
C22	0.28749 (8)	0.60383 (7)	0.41357 (8)	0.0225 (2)	
H22	0.3379	0.5945	0.4452	0.027*	
C23	0.36610 (7)	0.61857 (7)	0.20170 (8)	0.0201 (2)	
H23A	0.3711	0.5626	0.2300	0.024*	
H23B	0.3198	0.6182	0.1546	0.024*	
C24	0.43697 (8)	0.63556 (8)	0.15094 (9)	0.0254 (3)	
H24	0.4376	0.6850	0.1147	0.030*	

C25	0.49898 (9)	0.58703 (10)	0.15262 (11)	0.0371 (3)	
H25A	0.5005	0.5371	0.1881	0.045*	
H25B	0.5421	0.6021	0.1183	0.045*	
O2	0.18585 (5)	0.36974 (5)	0.40590 (6)	0.02223 (18)	
N2	0.15024 (6)	0.11116 (6)	0.62363 (7)	0.01892 (19)	
C26	0.21678 (6)	0.34586 (6)	0.48181 (8)	0.0161 (2)	
C27	0.16845 (6)	0.33557 (7)	0.56822 (8)	0.0159 (2)	
C28	0.21408 (7)	0.37105 (7)	0.65830 (8)	0.0201 (2)	
H28A	0.2100	0.4321	0.6563	0.024*	
H28B	0.1886	0.3519	0.7145	0.024*	
C29	0.30042 (7)	0.34756 (8)	0.67146 (8)	0.0208 (2)	
H29A	0.3054	0.2875	0.6841	0.025*	
H29B	0.3261	0.3774	0.7270	0.025*	
C30	0.34129 (7)	0.36908 (7)	0.58319 (8)	0.0203 (2)	
H30A	0.3973	0.3538	0.5943	0.024*	
H30B	0.3385	0.4296	0.5731	0.024*	
C31	0.30583 (6)	0.32545 (7)	0.49250 (8)	0.0166 (2)	
C32	0.09098 (7)	0.38307 (7)	0.55094 (9)	0.0225 (2)	
H32A	0.0597	0.3591	0.4963	0.034*	
H32B	0.1019	0.4413	0.5380	0.034*	
H32C	0.0619	0.3792	0.6074	0.034*	
C33	0.15176 (6)	0.24380 (7)	0.57470 (8)	0.01556 (19)	
C34	0.17183 (6)	0.19059 (7)	0.64835 (8)	0.0177 (2)	
H34	0.1971	0.2064	0.7081	0.021*	
C35	0.11643 (7)	0.11142 (7)	0.53173 (8)	0.0183 (2)	
C36	0.11489 (6)	0.19407 (7)	0.49905 (8)	0.0160 (2)	
C37	0.08394 (7)	0.20993 (8)	0.40543 (8)	0.0199 (2)	
H37	0.0807	0.2649	0.3819	0.024*	
C38	0.05828 (7)	0.14444 (8)	0.34808 (9)	0.0257 (3)	
H38	0.0381	0.1549	0.2844	0.031*	
C39	0.06150 (8)	0.06297 (9)	0.38196	0.0294 (3)	

			(10)		
H39	0.0439	0.0191	0.3408	0.035*	
C40	0.08984 (8)	0.04551 (8)	0.47405 (9)	0.0258 (3)	
H40	0.0912	-0.0096	0.4975	0.031*	
C41	0.16704 (8)	0.03764 (8)	0.68085 (9)	0.0284 (3)	
H41A	0.1193	0.0044	0.6819	0.043*	
H41B	0.1854	0.0539	0.7458	0.043*	
H41C	0.2077	0.0051	0.6535	0.043*	
C42	0.31739 (6)	0.23126 (7)	0.49497 (8)	0.0171 (2)	
C43	0.27796 (7)	0.18284 (7)	0.42458 (8)	0.0201 (2)	
H43	0.2466	0.2090	0.3744	0.024*	
C44	0.28348 (8)	0.09714 (8)	0.42619 (10)	0.0260 (3)	
H44	0.2551	0.0652	0.3783	0.031*	
C45	0.33037 (8)	0.05845 (8)	0.49766 (10)	0.0301 (3)	
H45	0.3332	-0.0002	0.5001	0.036*	
C46	0.37315 (8)	0.10551 (8)	0.56551 (10)	0.0291 (3)	
H46	0.4069	0.0791	0.6132	0.035*	
C47	0.36704 (7)	0.19130 (8)	0.56426 (9)	0.0229 (2)	
H47	0.3969	0.2230	0.6110	0.027*	
C48	0.34150 (7)	0.36162 (8)	0.40406 (8)	0.0215 (2)	
H48A	0.3140	0.3373	0.3460	0.026*	
H48B	0.3324	0.4221	0.4019	0.026*	
C49	0.42729 (8)	0.34548 (9)	0.40369 (9)	0.0296 (3)	
H49	0.4435	0.2896	0.3992	0.035*	
C50	0.48206 (9)	0.40238 (12)	0.40913 (11)	0.0452 (4)	
H50A	0.4683	0.4590	0.4138	0.054*	
H50B	0.5355	0.3869	0.4084	0.054*	

Atomic displacement parameters (Å^2)

	U^{11}	U^{22}	U^{33}	U^{12}	U^{13}	U^{23}
O1	0.0276 (5)	0.0263 (4)	0.0166 (4)	0.0036 (3)	0.0019 (3)	0.0003 (3)
N1	0.0215 (5)	0.0237 (5)	0.0163 (4)	0.0006 (4)	0.0068 (4)	0.0002 (4)
C1	0.0119 (4)	0.0205 (5)	0.0176 (5)	0.0007 (4)	0.0028 (4)	-0.0013 (4)
C2	0.0159 (5)	0.0165 (5)	0.0178 (5)	-0.0005 (4)	0.0018 (4)	-0.0021 (4)
C3	0.0180 (5)	0.0244 (6)	0.0244 (6)	0.0003 (4)	-0.0012 (4)	-0.0063 (5)
C4	0.0194 (5)	0.0267 (6)	0.0192 (5)	0.0038 (4)	-0.0041 (4)	-0.0049 (4)
C5	0.0174 (5)	0.0245 (6)	0.0206 (5)	0.0039 (4)	-0.0019 (4)	-0.0038 (4)
C6	0.0162 (5)	0.0180 (5)	0.0149 (5)	0.0019 (4)	0.0005 (4)	-0.0023 (4)
C7	0.0212 (5)	0.0203 (6)	0.0288 (6)	-0.0027 (4)	0.0076 (5)	0.0015 (5)
C8	0.0161 (5)	0.0154 (5)	0.0163 (5)	0.0013 (4)	0.0020 (4)	-0.0014 (4)
C9	0.0206 (5)	0.0195 (5)	0.0161 (5)	0.0023 (4)	0.0013 (4)	-0.0016 (4)
C10	0.0184 (5)	0.0158 (5)	0.0192 (5)	-0.0006 (4)	0.0047 (4)	-0.0030 (4)
C11	0.0158 (5)	0.0146 (5)	0.0164 (5)	0.0005 (4)	0.0026 (4)	-0.0017 (4)
C12	0.0204 (5)	0.0210 (5)	0.0176 (5)	-0.0012 (4)	0.0014 (4)	0.0002 (4)
C13	0.0231 (6)	0.0257 (6)	0.0211 (5)	0.0000 (5)	-0.0035 (4)	-0.0029 (5)
C14	0.0174 (5)	0.0242 (6)	0.0319 (6)	0.0022 (4)	-0.0022 (5)	-0.0101 (5)
C15	0.0169 (5)	0.0218 (6)	0.0280 (6)	-0.0012 (4)	0.0058 (4)	-0.0080 (5)
C16	0.0351 (7)	0.0373 (8)	0.0218 (6)	-0.0015 (6)	0.0141 (5)	0.0032 (5)
C17	0.0192 (5)	0.0145 (5)	0.0159 (5)	0.0013 (4)	0.0021 (4)	-0.0026 (4)

C18	0.0201 (5)	0.0170 (5)	0.0196 (5)	-0.0001 (4)	-0.0003 (4)	-0.0014 (4)
C19	0.0207 (6)	0.0203 (6)	0.0321 (6)	-0.0012 (4)	0.0041 (5)	-0.0032 (5)
C20	0.0314 (7)	0.0204 (6)	0.0367 (7)	-0.0031 (5)	0.0154 (5)	-0.0020 (5)
C21	0.0403 (7)	0.0200 (6)	0.0231 (6)	0.0018 (5)	0.0094 (5)	0.0027 (5)
C22	0.0280 (6)	0.0194 (5)	0.0200 (5)	0.0046 (5)	0.0014 (4)	0.0004 (4)
C23	0.0205 (5)	0.0214 (6)	0.0183 (5)	0.0028 (4)	0.0020 (4)	-0.0041 (4)
C24	0.0262 (6)	0.0301 (7)	0.0206 (5)	0.0029 (5)	0.0064 (5)	-0.0030 (5)
C25	0.0273 (7)	0.0414 (8)	0.0437 (8)	0.0064 (6)	0.0090 (6)	-0.0098 (7)
O2	0.0208 (4)	0.0250 (4)	0.0204 (4)	0.0009 (3)	-0.0011 (3)	0.0069 (3)
N2	0.0200 (5)	0.0152 (4)	0.0219 (5)	0.0015 (3)	0.0038 (4)	0.0045 (4)
C26	0.0173 (5)	0.0122 (5)	0.0186 (5)	-0.0021 (4)	0.0006 (4)	0.0006 (4)
C27	0.0164 (5)	0.0139 (5)	0.0174 (5)	0.0010 (4)	0.0010 (4)	0.0002 (4)
C28	0.0230 (6)	0.0183 (5)	0.0191 (5)	-0.0011 (4)	0.0021 (4)	-0.0039 (4)
C29	0.0214 (5)	0.0238 (6)	0.0170 (5)	-0.0040 (4)	0.0005 (4)	-0.0031 (4)
C30	0.0185 (5)	0.0213 (5)	0.0207 (5)	-0.0042 (4)	-0.0002 (4)	-0.0009 (4)
C31	0.0160 (5)	0.0176 (5)	0.0160 (5)	-0.0007 (4)	0.0015 (4)	0.0020 (4)
C32	0.0198 (5)	0.0203 (6)	0.0279 (6)	0.0047 (4)	0.0040 (4)	0.0022 (5)
C33	0.0142 (5)	0.0155 (5)	0.0172 (5)	-0.0004 (4)	0.0023 (4)	0.0005 (4)
C34	0.0175 (5)	0.0182 (5)	0.0174 (5)	0.0001 (4)	0.0020 (4)	0.0011 (4)
C35	0.0173 (5)	0.0177 (5)	0.0207 (5)	-0.0010 (4)	0.0065 (4)	0.0004 (4)
C36	0.0137 (5)	0.0171 (5)	0.0174 (5)	-0.0009 (4)	0.0036 (4)	0.0001 (4)
C37	0.0160 (5)	0.0246 (6)	0.0190 (5)	-0.0020 (4)	0.0007 (4)	0.0013 (4)

C38	0.0207 (6)	0.0353 (7)	0.0208 (6)	-0.0061 (5)	0.0004 (4)	-0.0046 (5)
C39	0.0281 (6)	0.0288 (7)	0.0315 (7)	-0.0099 (5)	0.0044 (5)	-0.0113 (5)
C40	0.0267 (6)	0.0199 (6)	0.0320 (6)	-0.0054 (5)	0.0089 (5)	-0.0036 (5)
C41	0.0355 (7)	0.0197 (6)	0.0307 (7)	0.0049 (5)	0.0066 (5)	0.0103 (5)
C42	0.0149 (5)	0.0181 (5)	0.0187 (5)	0.0018 (4)	0.0040 (4)	0.0016 (4)
C43	0.0180 (5)	0.0222 (6)	0.0209 (5)	0.0019 (4)	0.0058 (4)	-0.0022 (4)
C44	0.0240 (6)	0.0227 (6)	0.0329 (7)	-0.0004 (5)	0.0120 (5)	-0.0071 (5)
C45	0.0322 (7)	0.0182 (6)	0.0426 (8)	0.0056 (5)	0.0183 (6)	0.0015 (5)
C46	0.0281 (6)	0.0269 (7)	0.0333 (7)	0.0106 (5)	0.0077 (5)	0.0084 (5)
C47	0.0200 (5)	0.0250 (6)	0.0237 (6)	0.0044 (4)	0.0016 (4)	0.0029 (5)
C48	0.0192 (5)	0.0238 (6)	0.0218 (5)	-0.0003 (4)	0.0037 (4)	0.0051 (4)
C49	0.0215 (6)	0.0424 (8)	0.0255 (6)	0.0043 (5)	0.0064 (5)	0.0099 (5)
C50	0.0264 (7)	0.0675 (12)	0.0423 (9)	-0.0105 (7)	0.0066 (6)	0.0160 (8)

Geometric parameters (Å, º)

O1—C1	1.2165 (13)	O2—C26	1.2165 (13)
N1—C9	1.3676 (15)	N2—C34	1.3736 (15)
N1—C10	1.3696 (15)	N2—C35	1.3743 (15)
N1—C16	1.4508 (15)	N2—C41	1.4524 (15)
C1—C6	1.5491 (16)	C26—C27	1.5443 (16)
C1—C2	1.5501 (15)	C26—C31	1.5560 (15)
C2—C8	1.5175 (15)	C27—C33	1.5160 (15)
C2—C7	1.5338 (16)	C27—C32	1.5349 (16)
C2—C3	1.5450 (16)	C27—C28	1.5443 (15)
C3—C4	1.5241 (17)	C28—C29	1.5233 (17)
C3—H3A	0.9900	C28—H28A	0.9900
C3—H3B	0.9900	C28—H28B	0.9900
C4—C5	1.5179 (16)	C29—C30	1.5241 (17)

C4—H4A	0.9900	C29—H29A	0.9900
C4—H4B	0.9900	C29—H29B	0.9900
C5—C6	1.5415 (15)	C30—C31	1.5400 (16)
C5—H5A	0.9900	C30—H30A	0.9900
C5—H5B	0.9900	C30—H30B	0.9900
C6—C17	1.5303 (16)	C31—C42	1.5363 (16)
C6—C23	1.5534 (15)	C31—C48	1.5535 (16)
C7—H7A	0.9800	C32—H32A	0.9800
C7—H7B	0.9800	C32—H32B	0.9800
C7—H7C	0.9800	C32—H32C	0.9800
C8—C9	1.3725 (15)	C33—C34	1.3707 (15)
C8—C11	1.4439 (15)	C33—C36	1.4385 (15)
C9—H9	0.9500	C34—H34	0.9500
C10—C15	1.3962 (16)	C35—C40	1.3938 (17)
C10—C11	1.4136 (15)	C35—C36	1.4139 (15)
C11—C12	1.4048 (15)	C36—C37	1.4046 (15)
C12—C13	1.3832 (17)	C37—C38	1.3817 (17)
C12—H12	0.9500	C37—H37	0.9500
C13—C14	1.4025 (18)	C38—C39	1.402 (2)
C13—H13	0.9500	C38—H38	0.9500
C14—C15	1.3802 (18)	C39—C40	1.3774 (19)
C14—H14	0.9500	C39—H39	0.9500
C15—H15	0.9500	C40—H40	0.9500
C16—H16A	0.9800	C41—H41A	0.9800
C16—H16B	0.9800	C41—H41B	0.9800
C16—H16C	0.9800	C41—H41C	0.9800
C17—C18	1.3920 (16)	C42—C43	1.3931 (16)
C17—C22	1.3961 (16)	C42—C47	1.3974 (16)
C18—C19	1.3864 (17)	C43—C44	1.3894 (17)
C18—H18	0.9500	C43—H43	0.9500
C19—C20	1.3830 (19)	C44—C45	1.383 (2)
C19—H19	0.9500	C44—H44	0.9500
C20—C21	1.380 (2)	C45—C46	1.383 (2)

C20—H20	0.9500	C45—H45	0.9500
C21—C22	1.3859 (18)	C46—C47	1.3915 (18)
C21—H21	0.9500	C46—H46	0.9500
C22—H22	0.9500	C47—H47	0.9500
C23—C24	1.4901 (18)	C48—C49	1.4940 (17)
C23—H23A	0.9900	C48—H48A	0.9900
C23—H23B	0.9900	C48—H48B	0.9900
C24—C25	1.3199 (19)	C49—C50	1.312 (2)
C24—H24	0.9500	C49—H49	0.9500
C25—H25A	0.9500	C50—H50A	0.9500
C25—H25B	0.9500	C50—H50B	0.9500
C9—N1—C10	108.68 (9)	C34—N2—C35	108.60 (9)
C9—N1—C16	125.78 (10)	C34—N2—C41	126.06 (10)
C10—N1—C16	125.30 (10)	C35—N2—C41	125.08 (10)
O1—C1—C6	120.53 (10)	O2—C26—C27	120.60 (10)
O1—C1—C2	120.35 (10)	O2—C26—C31	120.00 (10)
C6—C1—C2	119.10 (9)	C27—C26—C31	119.39 (9)
C8—C2—C7	110.30 (9)	C33—C27—C32	109.54 (9)
C8—C2—C3	113.15 (9)	C33—C27—C26	105.70 (8)
C7—C2—C3	107.35 (9)	C32—C27—C26	109.72 (9)
C8—C2—C1	105.53 (9)	C33—C27—C28	113.49 (9)
C7—C2—C1	110.04 (9)	C32—C27—C28	108.23 (9)
C3—C2—C1	110.49 (9)	C26—C27—C28	110.11 (9)
C4—C3—C2	116.27 (10)	C29—C28—C27	115.05 (9)
C4—C3—H3A	108.2	C29—C28—H28A	108.5
C2—C3—H3A	108.2	C27—C28—H28A	108.5
C4—C3—H3B	108.2	C29—C28—H28B	108.5
C2—C3—H3B	108.2	C27—C28—H28B	108.5
H3A—C3—H3B	107.4	H28A—C28—H28B	107.5
C5—C4—C3	110.13 (10)	C28—C29—C30	110.81 (10)
C5—C4—H4A	109.6	C28—C29—H29A	109.5
C3—C4—H4A	109.6	C30—C29—H29A	109.5

C5—C4—H4B	109.6	C28—C29—H29B	109.5
C3—C4—H4B	109.6	C30—C29—H29B	109.5
H4A—C4—H4B	108.1	H29A—C29—H29B	108.1
C4—C5—C6	112.91 (9)	C29—C30—C31	113.60 (9)
C4—C5—H5A	109.0	C29—C30—H30A	108.8
C6—C5—H5A	109.0	C31—C30—H30A	108.8
C4—C5—H5B	109.0	C29—C30—H30B	108.8
C6—C5—H5B	109.0	C31—C30—H30B	108.8
H5A—C5—H5B	107.8	H30A—C30—H30B	107.7
C17—C6—C5	113.20 (9)	C42—C31—C30	113.26 (9)
C17—C6—C1	109.88 (9)	C42—C31—C48	109.42 (9)
C5—C6—C1	107.60 (9)	C30—C31—C48	110.03 (9)
C17—C6—C23	106.48 (9)	C42—C31—C26	109.64 (9)
C5—C6—C23	109.33 (9)	C30—C31—C26	107.16 (9)
C1—C6—C23	110.36 (9)	C48—C31—C26	107.14 (9)
C2—C7—H7A	109.5	C27—C32—H32A	109.5
C2—C7—H7B	109.5	C27—C32—H32B	109.5
H7A—C7—H7B	109.5	H32A—C32—H32B	109.5
C2—C7—H7C	109.5	C27—C32—H32C	109.5
H7A—C7—H7C	109.5	H32A—C32—H32C	109.5
H7B—C7—H7C	109.5	H32B—C32—H32C	109.5
C9—C8—C11	105.91 (10)	C34—C33—C36	106.10 (10)
C9—C8—C2	127.38 (10)	C34—C33—C27	128.65 (10)
C11—C8—C2	126.45 (10)	C36—C33—C27	125.13 (9)
N1—C9—C8	110.68 (10)	C33—C34—N2	110.51 (10)
N1—C9—H9	124.7	C33—C34—H34	124.7
C8—C9—H9	124.7	N2—C34—H34	124.7
N1—C10—C15	128.82 (11)	N2—C35—C40	129.71 (11)
N1—C10—C11	108.14 (10)	N2—C35—C36	107.80 (10)
C15—C10—C11	123.02 (11)	C40—C35—C36	122.45 (11)

C12—C11—C10	117.87 (10)	C37—C36—C35	118.35 (10)
C12—C11—C8	135.51 (10)	C37—C36—C33	134.60 (10)
C10—C11—C8	106.59 (9)	C35—C36—C33	106.96 (9)
C13—C12—C11	119.25 (11)	C38—C37—C36	119.11 (11)
C13—C12—H12	120.4	C38—C37—H37	120.4
C11—C12—H12	120.4	C36—C37—H37	120.4
C12—C13—C14	121.59 (11)	C37—C38—C39	121.31 (12)
C12—C13—H13	119.2	C37—C38—H38	119.3
C14—C13—H13	119.2	C39—C38—H38	119.3
C15—C14—C13	120.72 (11)	C40—C39—C38	121.03 (12)
C15—C14—H14	119.6	C40—C39—H39	119.5
C13—C14—H14	119.6	C38—C39—H39	119.5
C14—C15—C10	117.51 (11)	C39—C40—C35	117.73 (12)
C14—C15—H15	121.2	C39—C40—H40	121.1
C10—C15—H15	121.2	C35—C40—H40	121.1
N1—C16—H16A	109.5	N2—C41—H41A	109.5
N1—C16—H16B	109.5	N2—C41—H41B	109.5
H16A—C16—H16B	109.5	H41A—C41—H41B	109.5
N1—C16—H16C	109.5	N2—C41—H41C	109.5
H16A—C16—H16C	109.5	H41A—C41—H41C	109.5
H16B—C16—H16C	109.5	H41B—C41—H41C	109.5
C18—C17—C22	117.74 (11)	C43—C42—C47	117.84 (11)
C18—C17—C6	119.43 (10)	C43—C42—C31	119.20 (10)
C22—C17—C6	122.71 (10)	C47—C42—C31	122.95 (10)
C19—C18—C17	121.43 (11)	C44—C43—C42	121.42 (11)
C19—C18—H18	119.3	C44—C43—H43	119.3
C17—C18—H18	119.3	C42—C43—H43	119.3
C20—C19—C18	120.04 (12)	C45—C44—C43	119.83 (12)
C20—C19—H19	120.0	C45—C44—H44	120.1
C18—C19—H19	120.0	C43—C44—H44	120.1
C21—C20—C19	119.29 (12)	C46—C45—C44	119.69 (12)

C21—C20—H20	120.4	C46—C45—H45	120.2
C19—C20—H20	120.4	C44—C45—H45	120.2
C20—C21—C22	120.74 (12)	C45—C46—C47	120.36 (12)
C20—C21—H21	119.6	C45—C46—H46	119.8
C22—C21—H21	119.6	C47—C46—H46	119.8
C21—C22—C17	120.72 (12)	C46—C47—C42	120.70 (12)
C21—C22—H22	119.6	C46—C47—H47	119.6
C17—C22—H22	119.6	C42—C47—H47	119.6
C24—C23—C6	115.07 (10)	C49—C48—C31	113.26 (10)
C24—C23—H23A	108.5	C49—C48—H48A	108.9
C6—C23—H23A	108.5	C31—C48—H48A	108.9
C24—C23—H23B	108.5	C49—C48—H48B	108.9
C6—C23—H23B	108.5	C31—C48—H48B	108.9
H23A—C23—H23B	107.5	H48A—C48—H48B	107.7
C25—C24—C23	124.84 (13)	C50—C49—C48	125.21 (15)
C25—C24—H24	117.6	C50—C49—H49	117.4
C23—C24—H24	117.6	C48—C49—H49	117.4
C24—C25—H25A	120.0	C49—C50—H50A	120.0
C24—C25—H25B	120.0	C49—C50—H50B	120.0
H25A—C25—H25B	120.0	H50A—C50—H50B	120.0
O1—C1—C2—C8	96.86 (12)	O2—C26—C27—C33	100.89 (11)
C6—C1—C2—C8	-81.95 (11)	C31—C26—C27—C33	-79.17 (11)
O1—C1—C2—C7	-22.13 (14)	O2—C26—C27—C32	-17.14 (14)
C6—C1—C2—C7	159.07 (9)	C31—C26—C27—C32	162.81 (9)
O1—C1—C2—C3	-140.49 (11)	O2—C26—C27—C28	-136.16 (11)
C6—C1—C2—C3	40.70 (13)	C31—C26—C27—C28	43.78 (13)
C8—C2—C3—C4	76.39 (13)	C33—C27—	73.65 (12)

		C28—C29	
C7—C2—C3—C4	-161.68 (10)	C32—C27—C28—C29	-164.53 (10)
C1—C2—C3—C4	-41.68 (13)	C26—C27—C28—C29	-44.60 (13)
C2—C3—C4—C5	52.46 (14)	C27—C28—C29—C30	53.46 (13)
C3—C4—C5—C6	-60.50 (13)	C28—C29—C30—C31	-59.33 (13)
C4—C5—C6—C17	-64.77 (13)	C29—C30—C31—C42	-66.52 (13)
C4—C5—C6—C1	56.84 (12)	C29—C30—C31—C48	170.67 (10)
C4—C5—C6—C23	176.72 (10)	C29—C30—C31—C26	54.51 (12)
O1—C1—C6—C17	-103.16 (12)	O2—C26—C31—C42	-105.08 (11)
C2—C1—C6—C17	75.64 (12)	C27—C26—C31—C42	74.98 (12)
O1—C1—C6—C5	133.18 (11)	O2—C26—C31—C30	131.63 (11)
C2—C1—C6—C5	-48.01 (12)	C27—C26—C31—C30	-48.31 (12)
O1—C1—C6—C23	13.97 (14)	O2—C26—C31—C48	13.58 (14)
C2—C1—C6—C23	-167.23 (9)	C27—C26—C31—C48	-166.37 (9)
C7—C2—C8—C9	-134.28 (12)	C32—C27—C33—C34	-121.22 (12)
C3—C2—C8—C9	-14.01 (16)	C26—C27—C33—C34	120.63 (12)
C1—C2—C8—C9	106.91 (12)	C28—C27—C33—C34	-0.14 (16)
C7—C2—C8—C11	52.54 (14)	C32—C27—C33—C36	63.18 (13)
C3—C2—C8—C11	172.80 (10)	C26—C27—C33—C36	-54.97 (13)
C1—C2—C8—C11	-66.27 (13)	C28—C27—C33—C36	-175.73 (10)

C10—N1—C9—C8	0.06 (13)	C36—C33—C34—N2	0.21 (12)
C16—N1—C9—C8	174.79 (11)	C27—C33—C34—N2	-176.04 (10)
C11—C8—C9—N1	0.11 (13)	C35—N2—C34—C33	0.93 (13)
C2—C8—C9—N1	-174.19 (10)	C41—N2—C34—C33	175.25 (11)
C9—N1—C10—C15	178.39 (11)	C34—N2—C35—C40	175.73 (12)
C16—N1—C10—C15	3.62 (19)	C41—N2—C35—C40	1.34 (19)
C9—N1—C10—C11	-0.21 (13)	C34—N2—C35—C36	-1.70 (12)
C16—N1—C10—C11	-174.98 (11)	C41—N2—C35—C36	-176.09 (10)
N1—C10—C11—C12	178.59 (10)	N2—C35—C36—C37	178.88 (10)
C15—C10—C11—C12	-0.11 (16)	C40—C35—C36—C37	1.23 (16)
N1—C10—C11—C8	0.28 (12)	N2—C35—C36—C33	1.80 (12)
C15—C10—C11—C8	-178.42 (10)	C40—C35—C36—C33	-175.85 (11)
C9—C8—C11—C12	-178.11 (12)	C34—C33—C36—C37	-177.62 (12)
C2—C8—C11—C12	-3.7 (2)	C27—C33—C36—C37	-1.20 (19)
C9—C8—C11—C10	-0.23 (12)	C34—C33—C36—C35	-1.23 (12)
C2—C8—C11—C10	174.14 (10)	C27—C33—C36—C35	175.19 (10)
C10—C11—C12—C13	1.48 (16)	C35—C36—C37—C38	-1.79 (16)
C8—C11—C12—C13	179.18 (12)	C33—C36—C37—C38	174.29 (12)
C11—C12—C13—C14	-1.17 (18)	C36—C37—C38—C39	0.96 (18)
C12—C13—	-0.58 (19)	C37—C38—	0.5 (2)

C14—C15		C39—C40	
C13—C14—C15—C10	1.91 (17)	C38—C39—C40—C35	-1.11 (19)
N1—C10—C15—C14	180.00 (11)	N2—C35—C40—C39	-176.87 (12)
C11—C10—C15—C14	-1.59 (17)	C36—C35—C40—C39	0.22 (18)
C5—C6—C17—C18	162.48 (10)	C30—C31—C42—C43	170.06 (10)
C1—C6—C17—C18	42.16 (13)	C48—C31—C42—C43	-66.80 (12)
C23—C6—C17—C18	-77.37 (12)	C26—C31—C42—C43	50.44 (13)
C5—C6—C17—C22	-21.51 (15)	C30—C31—C42—C47	-11.01 (15)
C1—C6—C17—C22	-141.84 (10)	C48—C31—C42—C47	112.13 (12)
C23—C6—C17—C22	98.64 (12)	C26—C31—C42—C47	-130.63 (11)
C22—C17—C18—C19	2.13 (16)	C47—C42—C43—C44	4.15 (16)
C6—C17—C18—C19	178.33 (10)	C31—C42—C43—C44	-176.86 (10)
C17—C18—C19—C20	-1.73 (18)	C42—C43—C44—C45	-1.51 (17)
C18—C19—C20—C21	0.34 (19)	C43—C44—C45—C46	-1.81 (18)
C19—C20—C21—C22	0.57 (19)	C44—C45—C46—C47	2.37 (19)
C20—C21—C22—C17	-0.13 (19)	C45—C46—C47—C42	0.37 (19)
C18—C17—C22—C21	-1.20 (17)	C43—C42—C47—C46	-3.57 (17)
C6—C17—C22—C21	-177.27 (11)	C31—C42—C47—C46	177.49 (11)
C17—C6—C23—C24	-175.63 (10)	C42—C31—C48—C49	-60.28 (13)
C5—C6—C23—C24	-53.01 (13)	C30—C31—C48—C49	64.75 (13)

C1—C6—C23—C24	65.16 (13)	C26—C31—C48—C49	-179.08 (10)
C6—C23—C24—C25	115.29 (15)	C31—C48—C49—C50	-115.23 (15)

(±)-(2*R*,6*R*)-2-allyl-6-(1*H*-indol-3-yl)-2-(4-methoxyphenyl)-6-methylcyclohexanone (16a)

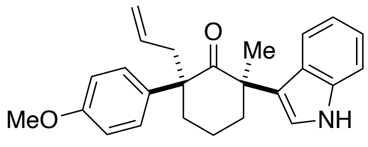

16a

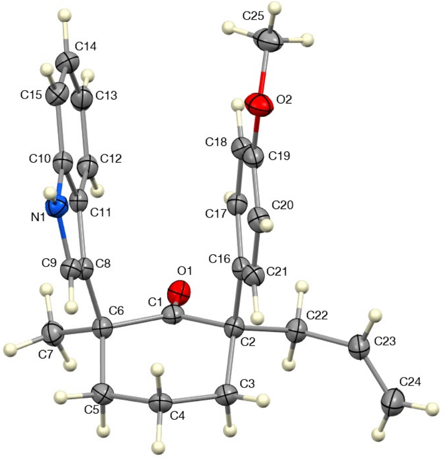

Sample Name: CCDC 2072238 / Satish10 (SCP1234P2)

Crystal data

$C_{25}H_{27}NO_2$	$F(000) = 800$
$M_r = 373.47$	$D_x = 1.229$ Mg m^{-3}
Monoclinic, $P2_1/n$	Cu $K\alpha$ radiation, $\lambda = 1.54184$ Å
$a = 12.1038$ (11) Å	Cell parameters from 7341 reflections
$b = 13.2693$ (11) Å	$\theta = 4.6$–$69.8°$
$c = 12.8176$ (11) Å	$\mu = 0.60$ mm^{-1}
$\beta = 101.283$ (5)°	$T = 90$ K
$V = 2018.8$ (3) Å^3	Plate, colourless
$Z = 4$	0.19 × 0.18 × 0.04 mm

Data collection

Bruker Kappa APEX-II DUO diffractometer	3791 independent reflections
Radiation source: IμS microfocus	3089 reflections with $I > 2\sigma(I)$
QUAZAR multilayer optics	$R_{int} = 0.086$
ϕ and ω scans	$\theta_{max} = 70.2°$, $\theta_{min} = 4.6°$
Absorption correction: multi-scan *SADABS* (Sheldrick, 2004)	$h = -14 - 14$

T_{min} = 0.727, T_{max} = 0.976	k = -15 – 5
23180 measured reflections	l = -14 – 5

Refinement

Refinement on F^2	0 restraints
Least-squares matrix: full	Hydrogen site location: mixed
$R[F^2 > 2\sigma(F^2)]$ = 0.047	H atoms treated by a mixture of independent and constrained refinement
$wR(F^2)$ = 0.137	$w = 1/[\sigma^2(F_o^2) + (0.0754P)^2 + 0.4441P]$ where $P = (F_o^2 + 2F_c^2)/3$
S = 1.06	$(\Delta/\sigma)_{max}$ < 0.001
3791 reflections	$\Delta\rho_{max}$ = 0.27 e Å^{-3}
258 parameters	$\Delta\rho_{min}$ = -0.27 e Å^{-3}

Fractional atomic coordinates and isotropic or equivalent isotropic displacement parameters (Å^2) for (SCP1234P2)

	x	y	z	U_{iso}*/U_{eq}
O1	0.41436 (9)	0.25404 (9)	0.27801 (9)	0.0249 (3)
O2	0.87750 (11)	0.49857 (9)	0.56711 (9)	0.0309 (3)
N1	0.73484 (12)	0.17582 (10)	0.61379 (11)	0.0231 (3)
H1N	0.8022 (18)	0.1756 (15)	0.6579 (16)	0.028*
C1	0.50865 (14)	0.21840 (12)	0.28991 (12)	0.0213 (3)
C2	0.59924 (13)	0.26939 (12)	0.23880 (12)	0.0213 (3)
C3	0.66083 (14)	0.18790 (12)	0.18694 (12)	0.0231 (4)
H3A	0.608690	0.160939	0.123875	0.028*
H3B	0.725083	0.219378	0.161888	0.028*
C4	0.70469 (14)	0.10067 (12)	0.26080 (12)	0.0228 (4)
H4A	0.742072	0.050648	0.222049	0.027*
H4B	0.761056	0.125962	0.321766	0.027*
C5	0.60806 (14)	0.05058 (12)	0.30133 (12)	0.0235 (4)
H5A	0.639316	-0.003631	0.351590	0.028*
H5B	0.557252	0.018607	0.240404	0.028*
C6	0.53804 (13)	0.12190 (12)	0.35742 (12)	0.0213 (3)

C7	0.42897 (14)	0.06744 (12)	0.36761 (13)	0.0253 (4)
H7A	0.447136	0.000663	0.398959	0.038*
H7B	0.380960	0.060068	0.297006	0.038*
H7C	0.389081	0.106770	0.413374	0.038*
C8	0.60080 (14)	0.15516 (11)	0.46695 (12)	0.0204 (3)
C9	0.70839 (14)	0.13110 (12)	0.51548 (12)	0.0220 (3)
H9	0.757816	0.089375	0.485470	0.026*
C10	0.64484 (14)	0.23253 (12)	0.62957 (13)	0.0227 (4)
C11	0.55780 (14)	0.21973 (11)	0.53999 (12)	0.0212 (3)
C12	0.45603 (14)	0.27149 (12)	0.53772 (13)	0.0239 (4)
H12	0.394886	0.263772	0.479268	0.029*
C13	0.44634 (15)	0.33382 (13)	0.62193 (13)	0.0269 (4)
H13	0.377631	0.368816	0.620752	0.032*
C14	0.53532 (16)	0.34664 (13)	0.70894 (13)	0.0282 (4)
H14	0.526424	0.391022	0.764869	0.034*
C15	0.63564 (15)	0.29573 (13)	0.71454 (13)	0.0260 (4)
H15	0.695967	0.303338	0.773749	0.031*
C16	0.67605 (14)	0.33066 (11)	0.32607 (12)	0.0216 (4)
C17	0.62695 (14)	0.39352 (12)	0.39104 (12)	0.0225 (3)
H17	0.547125	0.398012	0.379085	0.027*
C18	0.69024 (14)	0.44999 (12)	0.47276 (13)	0.0243 (4)
H18	0.654162	0.491058	0.516778	0.029*
C19	0.80700 (15)	0.44551 (12)	0.48913 (13)	0.0251 (4)
C20	0.85821 (14)	0.38486 (12)	0.42432 (13)	0.0246 (4)
H20	0.938076	0.382122	0.435204	0.029*
C21	0.79373 (14)	0.32804 (12)	0.34357 (13)	0.0236 (4)
H21	0.830053	0.286944	0.299759	0.028*
C22	0.53937 (14)	0.34221 (12)	0.15007 (13)	0.0240 (4)
H22A	0.482295	0.303961	0.099470	0.029*
H22B	0.499496	0.395059	0.182815	0.029*
C23	0.61929 (15)	0.39152 (12)	0.09007 (13)	0.0265 (4)
H23	0.672790	0.437538	0.127702	0.032*
C24	0.62064 (18)	0.37567 (15)	-0.01071 (15)	0.0376 (5)

H24A	0.568371	0.330147	-0.050853	0.045*
H24B	0.673881	0.409712	-0.043535	0.045*
C25	0.82837 (17)	0.57720 (14)	0.61845 (15)	0.0345 (4)
H25A	0.790478	0.625126	0.565043	0.052*
H25B	0.887435	0.612153	0.668668	0.052*
H25C	0.773403	0.548445	0.656951	0.052*

Atomic displacement parameters (Å^2) for (SCP1234P2)

	U^{11}	U^{22}	U^{33}	U^{12}	U^{13}	U^{23}
O1	0.0245 (6)	0.0268 (6)	0.0233 (6)	0.0003 (5)	0.0047 (5)	0.0030 (4)
O2	0.0359 (7)	0.0257 (6)	0.0289 (6)	-0.0017 (5)	0.0008 (5)	-0.0067 (5)
N1	0.0242 (7)	0.0240 (7)	0.0209 (7)	0.0010 (5)	0.0036 (6)	0.0003 (5)
C1	0.0258 (8)	0.0201 (8)	0.0173 (7)	-0.0016 (6)	0.0027 (6)	-0.0027 (6)
C2	0.0260 (8)	0.0199 (8)	0.0192 (7)	-0.0009 (6)	0.0076 (6)	0.0021 (6)
C3	0.0283 (8)	0.0217 (8)	0.0201 (7)	-0.0009 (6)	0.0069 (7)	-0.0019 (6)
C4	0.0282 (8)	0.0197 (8)	0.0219 (7)	0.0011 (6)	0.0086 (7)	-0.0025 (6)
C5	0.0293 (8)	0.0186 (8)	0.0225 (8)	-0.0011 (6)	0.0052 (7)	-0.0016 (6)
C6	0.0255 (8)	0.0187 (8)	0.0207 (7)	-0.0025 (6)	0.0069 (6)	0.0007 (6)
C7	0.0275 (8)	0.0229 (8)	0.0256 (8)	-0.0039 (6)	0.0054 (7)	0.0018 (6)
C8	0.0267 (8)	0.0169 (7)	0.0196 (7)	-0.0025 (6)	0.0091 (6)	0.0023 (6)
C9	0.0272 (8)	0.0194 (7)	0.0208 (7)	0.0001 (6)	0.0079 (6)	0.0007 (6)
C10	0.0277 (8)	0.0197 (8)	0.0222 (8)	-0.0006 (6)	0.0085 (7)	0.0034 (6)
C11	0.0266 (8)	0.0180 (7)	0.0203 (7)	-0.0024 (6)	0.0077 (7)	0.0035 (6)
C12	0.0274 (8)	0.0229 (8)	0.0219 (8)	0.0006 (6)	0.0061 (7)	0.0044 (6)
C13	0.0323 (9)	0.0231 (8)	0.0282 (8)	0.0049 (7)	0.0132 (7)	0.0052 (6)

C14	0.0397 (10)	0.0240 (8)	0.0234 (8)	0.0023 (7)	0.0127 (7)	-0.0004 (6)
C15	0.0325 (9)	0.0256 (8)	0.0203 (8)	-0.0010 (7)	0.0061 (7)	0.0008 (6)
C16	0.0285 (8)	0.0163 (7)	0.0205 (7)	-0.0018 (6)	0.0058 (6)	0.0031 (6)
C17	0.0260 (8)	0.0205 (8)	0.0222 (7)	-0.0009 (6)	0.0077 (6)	0.0032 (6)
C18	0.0340 (9)	0.0187 (8)	0.0214 (7)	0.0009 (6)	0.0083 (7)	0.0009 (6)
C19	0.0327 (9)	0.0193 (8)	0.0219 (8)	-0.0026 (6)	0.0022 (7)	0.0023 (6)
C20	0.0256 (8)	0.0208 (8)	0.0270 (8)	-0.0010 (6)	0.0044 (7)	0.0027 (6)
C21	0.0273 (8)	0.0204 (8)	0.0240 (8)	0.0013 (6)	0.0072 (7)	0.0010 (6)
C22	0.0278 (8)	0.0228 (8)	0.0218 (8)	-0.0004 (6)	0.0057 (7)	0.0023 (6)
C23	0.0333 (9)	0.0216 (8)	0.0247 (8)	-0.0028 (7)	0.0062 (7)	0.0036 (6)
C24	0.0521 (12)	0.0342 (10)	0.0290 (9)	-0.0108 (9)	0.0134 (9)	0.0018 (7)
C25	0.0454 (11)	0.0259 (9)	0.0291 (9)	0.0030 (8)	-0.0003 (8)	-0.0064 (7)

Geometric parameters (Å, º) for (SCP1234P2)

O1—C1	1.217 (2)	C10—C11	1.409 (2)
O2—C19	1.374 (2)	C11—C12	1.406 (2)
O2—C25	1.424 (2)	C12—C13	1.383 (2)
N1—C10	1.371 (2)	C12—H12	0.9500
N1—C9	1.373 (2)	C13—C14	1.401 (3)
N1—H1N	0.90 (2)	C13—H13	0.9500
C1—C2	1.540 (2)	C14—C15	1.379 (3)
C1—C6	1.547 (2)	C14—H14	0.9500
C2—C3	1.536 (2)	C15—H15	0.9500
C2—C16	1.539 (2)	C16—C17	1.391 (2)
C2—C22	1.559 (2)	C16—C21	1.398 (2)
C3—C4	1.523 (2)	C17—C18	1.390 (2)

C3—H3A	0.9900	C17—H17	0.9500
C3—H3B	0.9900	C18—C19	1.389 (2)
C4—C5	1.522 (2)	C18—H18	0.9500
C4—H4A	0.9900	C19—C20	1.387 (3)
C4—H4B	0.9900	C20—C21	1.390 (2)
C5—C6	1.539 (2)	C20—H20	0.9500
C5—H5A	0.9900	C21—H21	0.9500
C5—H5B	0.9900	C22—C23	1.499 (2)
C6—C8	1.525 (2)	C22—H22A	0.9900
C6—C7	1.533 (2)	C22—H22B	0.9900
C7—H7A	0.9800	C23—C24	1.312 (3)
C7—H7B	0.9800	C23—H23	0.9500
C7—H7C	0.9800	C24—H24A	0.9500
C8—C9	1.367 (2)	C24—H24B	0.9500
C8—C11	1.439 (2)	C25—H25A	0.9800
C9—H9	0.9500	C25—H25B	0.9800
C10—C15	1.396 (2)	C25—H25C	0.9800
C19—O2—C25	117.09 (14)	C12—C11—C10	118.07 (15)
C10—N1—C9	108.86 (13)	C12—C11—C8	134.77 (14)
C10—N1—H1N	124.0 (13)	C10—C11—C8	107.10 (14)
C9—N1—H1N	126.7 (13)	C13—C12—C11	118.94 (15)
O1—C1—C2	120.29 (14)	C13—C12—H12	120.5
O1—C1—C6	119.93 (15)	C11—C12—H12	120.5
C2—C1—C6	119.78 (13)	C12—C13—C14	121.64 (16)
C3—C2—C16	114.26 (13)	C12—C13—H13	119.2
C3—C2—C1	108.72 (13)	C14—C13—H13	119.2
C16—C2—C1	107.58 (12)	C15—C14—C13	120.92 (16)
C3—C2—C22	108.44 (13)	C15—C14—H14	119.5
C16—C2—C22	109.34 (12)	C13—C14—H14	119.5
C1—C2—C22	108.34 (13)	C14—C15—C10	117.22 (15)
C4—C3—C2	113.92 (13)	C14—C15—H15	121.4
C4—C3—H3A	108.8	C10—C15—H15	121.4

C2—C3—H3A	108.8	C17—C16—C21	117.38 (14)
C4—C3—H3B	108.8	C17—C16—C2	118.92 (14)
C2—C3—H3B	108.8	C21—C16—C2	123.70 (15)
H3A—C3—H3B	107.7	C18—C17—C16	122.50 (15)
C5—C4—C3	110.26 (13)	C18—C17—H17	118.8
C5—C4—H4A	109.6	C16—C17—H17	118.8
C3—C4—H4A	109.6	C19—C18—C17	119.04 (16)
C5—C4—H4B	109.6	C19—C18—H18	120.5
C3—C4—H4B	109.6	C17—C18—H18	120.5
H4A—C4—H4B	108.1	O2—C19—C20	116.50 (15)
C4—C5—C6	114.89 (13)	O2—C19—C18	123.82 (16)
C4—C5—H5A	108.5	C20—C19—C18	119.68 (15)
C6—C5—H5A	108.5	C19—C20—C21	120.60 (16)
C4—C5—H5B	108.5	C19—C20—H20	119.7
C6—C5—H5B	108.5	C21—C20—H20	119.7
H5A—C5—H5B	107.5	C20—C21—C16	120.78 (16)
C8—C6—C7	109.88 (13)	C20—C21—H21	119.6
C8—C6—C5	112.79 (13)	C16—C21—H21	119.6
C7—C6—C5	108.18 (13)	C23—C22—C2	112.98 (13)
C8—C6—C1	107.10 (12)	C23—C22—H22A	109.0
C7—C6—C1	109.28 (13)	C2—C22—H22A	109.0
C5—C6—C1	109.57 (13)	C23—C22—H22B	109.0
C6—C7—H7A	109.5	C2—C22—H22B	109.0
C6—C7—H7B	109.5	H22A—C22—H22B	107.8
H7A—C7—H7B	109.5	C24—C23—C22	124.66 (16)
C6—C7—H7C	109.5	C24—C23—H23	117.7
H7A—C7—H7C	109.5	C22—C23—H23	117.7
H7B—C7—H7C	109.5	C23—C24—H24A	120.0
C9—C8—C11	106.06 (13)	C23—C24—H24B	120.0
C9—C8—C6	127.59 (15)	H24A—C24—H24B	120.0
C11—C8—C6	126.35 (14)	O2—C25—H25A	109.5
C8—C9—N1	110.31 (15)	O2—C25—H25B	109.5

C8—C9—H9	124.8	H25A—C25—H25B	109.5
N1—C9—H9	124.8	O2—C25—H25C	109.5
N1—C10—C15	129.17 (15)	H25A—C25—H25C	109.5
N1—C10—C11	107.62 (14)	H25B—C25—H25C	109.5
C15—C10—C11	123.18 (15)		
O1—C1—C2—C3	-135.15 (15)	C15—C10—C11—C8	176.10 (15)
C6—C1—C2—C3	45.34 (17)	C9—C8—C11—C12	178.14 (17)
O1—C1—C2—C16	100.62 (16)	C6—C8—C11—C12	-1.4 (3)
C6—C1—C2—C16	-78.89 (16)	C9—C8—C11—C10	1.07 (17)
O1—C1—C2—C22	-17.5 (2)	C6—C8—C11—C10	-178.44 (14)
C6—C1—C2—C22	163.00 (13)	C10—C11—C12—C13	1.2 (2)
C16—C2—C3—C4	68.58 (17)	C8—C11—C12—C13	-175.65 (17)
C1—C2—C3—C4	-51.59 (17)	C11—C12—C13—C14	0.1 (2)
C22—C2—C3—C4	-169.19 (13)	C12—C13—C14—C15	-1.2 (3)
C2—C3—C4—C5	58.28 (17)	C13—C14—C15—C10	0.9 (3)
C3—C4—C5—C6	-55.71 (17)	N1—C10—C15—C14	178.26 (16)
C4—C5—C6—C8	-72.41 (17)	C11—C10—C15—C14	0.5 (2)
C4—C5—C6—C7	165.84 (13)	C3—C2—C16—C17	-168.61 (13)
C4—C5—C6—C1	46.78 (17)	C1—C2—C16—C17	-47.81 (18)
O1—C1—C6—C8	-99.88 (17)	C22—C2—C16—	69.65 (18)

		C17	
C2—C1—C6—C8	79.63 (17)	C3—C2—C16—C21	12.2 (2)
O1—C1—C6—C7	19.10 (19)	C1—C2—C16—C21	133.02 (15)
C2—C1—C6—C7	-161.39 (13)	C22—C2—C16—C21	-109.52 (17)
O1—C1—C6—C5	137.47 (15)	C21—C16—C17—C18	-1.9 (2)
C2—C1—C6—C5	-43.02 (17)	C2—C16—C17—C18	178.87 (14)
C7—C6—C8—C9	122.78 (17)	C16—C17—C18—C19	1.4 (2)
C5—C6—C8—C9	2.0 (2)	C25—O2—C19—C20	166.45 (15)
C1—C6—C8—C9	-118.63 (17)	C25—O2—C19—C18	-13.4 (2)
C7—C6—C8—C11	-57.8 (2)	C17—C18—C19—O2	179.64 (14)
C5—C6—C8—C11	-178.60 (14)	C17—C18—C19—C20	-0.2 (2)
C1—C6—C8—C11	60.8 (2)	O2—C19—C20—C21	179.72 (14)
C11—C8—C9—N1	0.34 (18)	C18—C19—C20—C21	-0.4 (2)
C6—C8—C9—N1	179.84 (14)	C19—C20—C21—C16	-0.1 (2)
C10—N1—C9—C8	-1.66 (18)	C17—C16—C21—C20	1.2 (2)
C9—N1—C10—C15	-175.72 (16)	C2—C16—C21—C20	-179.60 (14)
C9—N1—C10—C11	2.30 (18)	C3—C2—C22—C23	-58.63 (17)
N1—C10—C11—C12	-179.71 (14)	C16—C2—C22—C23	66.55 (17)
C15—C10—C11—C12	-1.5 (2)	C1—C2—C22—C23	-176.47 (13)
N1—C10—C11—C8	-2.07 (17)	C2—C22—C23—C24	113.6 (2)

Hydrogen-bond geometry (Å, º) for (SCP1234P2)

D—H···*A*	*D*—H	H···*A*	*D*···*A*	*D*—H···*A*
N1—H1*N*···O1^{i}	0.90 (2)	2.07 (2)	2.8685 (18)	148.5 (18)

Symmetry code: (i) *x*+1/2, -*y*+1/2, *z*+1/2.

(±)-(2*R*,6*R*)-2-allyl-2-(4-chlorophenyl)-6-(1*H*-indol-3-yl)-6-methylcyclohexanone (16g)

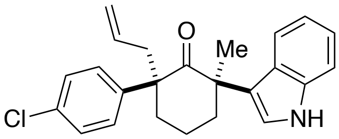

16g

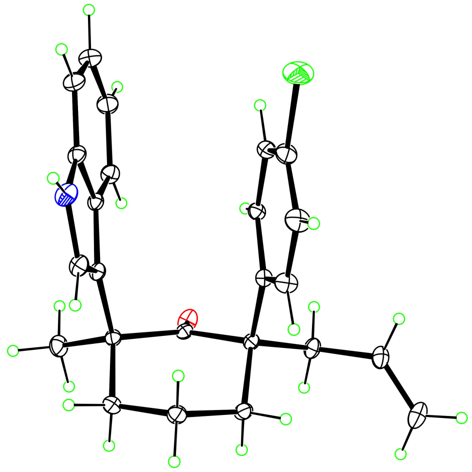

Sample Name: CCDC 2072239 / Satish5 (SCP1164)

Crystal data

$C_{24}H_{24}ClNO$	D_x = 1.256 Mg m^{-3}
M_r = 377.89	Mo *K*α radiation, λ = 0.71073 Å
Orthorhombic, $P2_12_12_1$	Cell parameters from 9867 reflections
a = 8.3903 (4) Å	θ = 2.4–41.1°
b = 13.9407 (7) Å	μ = 0.20 mm^{-1}
c = 17.0863 (9) Å	*T* = 90 K
V = 1998.53 (17) Å^3	Needle, colourless
Z = 4	0.49 × 0.17 × 0.14 mm
F(000) = 800	

Data collection

Bruker Kappa APEX-II DUO diffractometer	13267 independent reflections
Radiation source: fine-focus sealed tube	11932 reflections with $I > 2\sigma(I)$
TRIUMPH curved graphite	R_{int} = 0.039
ϕ and ω scans	θ_{max} = 41.2°, θ_{min} = 1.9°
Absorption correction: multi-scan *SADABS* (Sheldrick, 2004)	*h* = -15 – 12

T_{min} = 0.926, T_{max} = 0.972	k = -25 – 25
54330 measured reflections	l = -31 – 31

Refinement

Refinement on F^2	Hydrogen site location: mixed
Least-squares matrix: full	H atoms treated by a mixture of independent and constrained refinement
$R[F^2 > 2\sigma(F^2)]$ = 0.034	$w = 1/[\sigma^2(F_o^2) + (0.0488P)^2 + 0.0978P]$ where $P = (F_o^2 + 2F_c^2)/3$
$wR(F^2)$ = 0.089	$(\Delta/\sigma)_{max}$ = 0.001
S = 1.06	$\Delta\rho_{max}$ = 0.47 e Å^{-3}
13267 reflections	$\Delta\rho_{min}$ = -0.20 e Å^{-3}
249 parameters	Absolute structure: Refined as an inversion twin.
0 restraints	Flack parameter: 0.46 (3)

Fractional atomic coordinates and isotropic or equivalent isotropic displacement parameters (Å^2)

	x	y	z	U_{iso}*/U_{eq}	
Cl1	0.98730 (3)	0.74053 (2)	0.25485 (2)	0.02717 (6)	
O1	0.33757 (7)	0.45783 (5)	0.43247 (4)	0.01544 (11)	
N1	0.98728 (9)	0.47066 (6)	0.43931 (5)	0.01625 (12)	
H1N	1.086 (2)	0.4707 (12)	0.4295 (10)	0.020*	
C1	0.44897 (9)	0.42920 (6)	0.39294 (4)	0.00960 (11)	
C2	0.45728 (9)	0.45284 (5)	0.30473 (4)	0.00973 (11)	
C3	0.48002 (10)	0.35682 (6)	0.26143 (5)	0.01226 (12)	
H3A	0.382919	0.317325	0.268192	0.015*	
H3B	0.492972	0.369695	0.204809	0.015*	
C4	0.62352 (11)	0.30029 (6)	0.29042 (5)	0.01378 (13)	

H4A	0.721724	0.338362	0.282264	0.017*	
H4B	0.633428	0.240080	0.260086	0.017*	
C5	0.60538 (11)	0.27679 (6)	0.37717 (5)	0.01435 (13)	
H5A	0.702331	0.242719	0.394994	0.017*	
H5B	0.514066	0.232520	0.383660	0.017*	
C6	0.57888 (9)	0.36529 (6)	0.43029 (4)	0.01037 (11)	
C7	0.52008 (11)	0.32914 (7)	0.51028 (5)	0.01592 (14)	
H7A	0.601531	0.287820	0.533796	0.024*	
H7B	0.421371	0.292555	0.503285	0.024*	
H7C	0.499876	0.384026	0.544716	0.024*	
C8	0.73008 (9)	0.42340 (6)	0.44205 (4)	0.01017 (11)	
C9	0.88031 (10)	0.40240 (6)	0.41491 (5)	0.01366 (13)	
H9	0.906440	0.348272	0.383665	0.016*	
C10	0.90829 (9)	0.53794 (6)	0.48303 (5)	0.01328 (12)	
C11	0.74593 (9)	0.51049 (6)	0.48701 (5)	0.01056 (11)	
C12	0.63916 (10)	0.57019 (6)	0.52728 (5)	0.01358 (13)	
H12	0.529901	0.553085	0.531552	0.016*	
C13	0.69463 (13)	0.65429 (7)	0.56073 (6)	0.01846 (15)	
H13	0.622476	0.695003	0.587801	0.022*	
C14	0.85641 (14)	0.68036 (7)	0.55521 (6)	0.02162 (17)	
H14	0.891440	0.738568	0.578446	0.026*	
C15	0.96527 (12)	0.62277 (7)	0.51658 (6)	0.01936 (16)	
H15	1.074496	0.640187	0.512992	0.023*	
C16	0.59151 (9)	0.52448 (6)	0.28970 (4)	0.01007 (11)	
C17	0.60419	0.60515 (6)	0.33776 (4)	0.01121	

	(10)			(11)	
H17	0.528024	0.614473	0.378192	0.013*	
C18	0.72512 (11)	0.67208 (6)	0.32803 (5)	0.01392 (13)	
H18	0.733978	0.725662	0.362109	0.017*	
C19	0.83265 (11)	0.65879 (7)	0.26736 (5)	0.01590 (14)	
C20	0.81990 (12)	0.58208 (7)	0.21628 (6)	0.01794 (15)	
H20	0.892700	0.575235	0.174063	0.022*	
C21	0.69899 (11)	0.51512 (7)	0.22759 (5)	0.01487 (13)	
H21	0.689365	0.462397	0.192687	0.018*	
C22	0.29695 (10)	0.49856 (6)	0.27846 (5)	0.01309 (13)	
H22A	0.209099	0.452738	0.288380	0.016*	
H22B	0.276481	0.556917	0.309908	0.016*	
C23	0.29893 (11)	0.52453 (7)	0.19329 (5)	0.01587 (13)	
H23	0.365802	0.576015	0.177513	0.019*	
C24	0.21409 (13)	0.48081 (9)	0.13848 (6)	0.02168 (17)	
H24A	0.145906	0.429024	0.152094	0.026*	
H24B	0.221434	0.501234	0.085555	0.026*	

Atomic displacement parameters (Å^2)

	U^{11}	U^{22}	U^{33}	U^{12}	U^{13}	U^{23}
Cl1	0.02417 (11)	0.02881 (12)	0.02854 (12)	-0.01574 (9)	0.00391 (9)	0.00336 (9)
O1	0.0084 (2)	0.0252 (3)	0.0128 (2)	0.0023 (2)	0.00200 (18)	-0.0005 (2)
N1	0.0067 (2)	0.0227 (3)	0.0193 (3)	-0.0004 (2)	0.0003 (2)	-0.0004 (3)
C1	0.0070 (2)	0.0114 (3)	0.0104 (2)	-0.0013 (2)	0.00018 (19)	-0.0006 (2)
C2	0.0087 (2)	0.0109 (3)	0.0096 (2)	0.0001 (2)	-0.0001	-0.0004

					(2)	(2)
C3	0.0124 (3)	0.0121 (3)	0.0123 (3)	0.0002 (2)	-0.0017 (2)	-0.0024 (2)
C4	0.0148 (3)	0.0119 (3)	0.0146 (3)	0.0028 (2)	-0.0009 (2)	-0.0028 (2)
C5	0.0166 (3)	0.0106 (3)	0.0159 (3)	0.0009 (2)	-0.0023 (3)	-0.0003 (2)
C6	0.0093 (3)	0.0110 (3)	0.0108 (3)	-0.0007 (2)	-0.0005 (2)	0.0010 (2)
C7	0.0164 (3)	0.0181 (3)	0.0133 (3)	-0.0026 (3)	0.0009 (3)	0.0049 (3)
C8	0.0078 (2)	0.0122 (3)	0.0105 (2)	0.0006 (2)	-0.0002 (2)	0.0003 (2)
C9	0.0093 (3)	0.0169 (3)	0.0147 (3)	0.0025 (2)	-0.0001 (2)	-0.0007 (2)
C10	0.0096 (3)	0.0160 (3)	0.0142 (3)	-0.0019 (2)	-0.0017 (2)	0.0015 (2)
C11	0.0089 (3)	0.0121 (3)	0.0106 (2)	-0.0002 (2)	-0.0007 (2)	0.0008 (2)
C12	0.0138 (3)	0.0145 (3)	0.0124 (3)	0.0011 (2)	0.0006 (2)	-0.0013 (2)
C13	0.0243 (4)	0.0152 (3)	0.0159 (3)	0.0014 (3)	-0.0018 (3)	-0.0034 (3)
C14	0.0278 (5)	0.0157 (4)	0.0214 (4)	-0.0047 (3)	-0.0070 (3)	-0.0020 (3)
C15	0.0170 (3)	0.0192 (4)	0.0218 (4)	-0.0070 (3)	-0.0060 (3)	0.0021 (3)
C16	0.0095 (3)	0.0110 (3)	0.0098 (2)	0.0006 (2)	0.0010 (2)	0.0000 (2)
C17	0.0123 (3)	0.0107 (3)	0.0107 (3)	-0.0001 (2)	0.0010 (2)	0.0001 (2)
C18	0.0165 (3)	0.0123 (3)	0.0129 (3)	-0.0029 (2)	-0.0009 (2)	0.0013 (2)
C19	0.0142 (3)	0.0167 (3)	0.0168 (3)	-0.0046 (3)	0.0012 (3)	0.0038 (3)
C20	0.0167 (3)	0.0200 (4)	0.0171 (3)	-0.0034 (3)	0.0070 (3)	0.0009 (3)
C21	0.0157 (3)	0.0156 (3)	0.0134 (3)	-0.0017 (3)	0.0053 (2)	-0.0015 (2)
C22	0.0100 (3)	0.0167 (3)	0.0125 (3)	0.0024 (2)	-0.0017	0.0002 (2)

					(2)	
C23	0.0146 (3)	0.0182 (3)	0.0148 (3)	0.0019 (3)	-0.0028 (2)	0.0030 (3)
C24	0.0199 (4)	0.0295 (5)	0.0157 (3)	0.0010 (4)	-0.0063 (3)	0.0016 (3)

Geometric parameters (Å, º)

Cl1—C19	1.7401 (9)	C10—C15	1.3985 (13)
O1—C1	1.2202 (10)	C10—C11	1.4166 (11)
N1—C10	1.3701 (12)	C11—C12	1.4031 (11)
N1—C9	1.3728 (12)	C12—C13	1.3849 (13)
N1—H1N	0.843 (17)	C12—H12	0.9500
C1—C2	1.5444 (10)	C13—C14	1.4083 (16)
C1—C6	1.5457 (11)	C13—H13	0.9500
C2—C16	1.5271 (11)	C14—C15	1.3836 (16)
C2—C3	1.5413 (11)	C14—H14	0.9500
C2—C22	1.5548 (11)	C15—H15	0.9500
C3—C4	1.5219 (12)	C16—C17	1.3965 (11)
C3—H3A	0.9900	C16—C21	1.3987 (11)
C3—H3B	0.9900	C17—C18	1.3884 (12)
C4—C5	1.5255 (12)	C17—H17	0.9500
C4—H4A	0.9900	C18—C19	1.3867 (13)
C4—H4B	0.9900	C18—H18	0.9500
C5—C6	1.5477 (12)	C19—C20	1.3845 (14)
C5—H5A	0.9900	C20—C21	1.3920 (13)
C5—H5B	0.9900	C20—H20	0.9500
C6—C8	1.5186 (11)	C21—H21	0.9500
C6—C7	1.5378 (11)	C22—C23	1.4997 (12)
C7—H7A	0.9800	C22—H22A	0.9900
C7—H7B	0.9800	C22—H22B	0.9900
C7—H7C	0.9800	C23—C24	1.3250 (14)
C8—C9	1.3746 (11)	C23—H23	0.9500
C8—C11	1.4427 (11)	C24—H24A	0.9500
C9—H9	0.9500	C24—H24B	0.9500

C10—N1—C9	108.90 (7)	N1—C10—C15	129.57 (8)
C10—N1—H1N	125.6 (12)	N1—C10—C11	107.84 (7)
C9—N1—H1N	125.5 (12)	C15—C10—C11	122.52 (8)
O1—C1—C2	120.33 (7)	C12—C11—C10	118.51 (8)
O1—C1—C6	120.01 (7)	C12—C11—C8	134.53 (7)
C2—C1—C6	119.61 (6)	C10—C11—C8	106.89 (7)
C16—C2—C3	113.33 (6)	C13—C12—C11	119.34 (8)
C16—C2—C1	109.70 (6)	C13—C12—H12	120.3
C3—C2—C1	106.78 (6)	C11—C12—H12	120.3
C16—C2—C22	108.75 (6)	C12—C13—C14	120.98 (9)
C3—C2—C22	108.94 (6)	C12—C13—H13	119.5
C1—C2—C22	109.28 (6)	C14—C13—H13	119.5
C4—C3—C2	113.04 (6)	C15—C14—C13	121.23 (9)
C4—C3—H3A	109.0	C15—C14—H14	119.4
C2—C3—H3A	109.0	C13—C14—H14	119.4
C4—C3—H3B	109.0	C14—C15—C10	117.42 (9)
C2—C3—H3B	109.0	C14—C15—H15	121.3
H3A—C3—H3B	107.8	C10—C15—H15	121.3
C3—C4—C5	110.40 (7)	C17—C16—C21	118.18 (7)
C3—C4—H4A	109.6	C17—C16—C2	118.94 (6)
C5—C4—H4A	109.6	C21—C16—C2	122.83 (7)
C3—C4—H4B	109.6	C18—C17—C16	121.76 (7)
C5—C4—H4B	109.6	C18—C17—H17	119.1
H4A—C4—H4B	108.1	C16—C17—H17	119.1
C4—C5—C6	114.39 (7)	C19—C18—C17	118.37 (8)
C4—C5—H5A	108.7	C19—C18—H18	120.8
C6—C5—H5A	108.7	C17—C18—H18	120.8
C4—C5—H5B	108.7	C20—C19—C18	121.61 (8)
C6—C5—H5B	108.7	C20—C19—Cl1	119.08 (7)
H5A—C5—H5B	107.6	C18—C19—Cl1	119.31 (7)
C8—C6—C7	108.98 (6)	C19—C20—C21	119.12 (8)
C8—C6—C1	109.64 (6)	C19—C20—H20	120.4

C7—C6—C1	109.24 (6)	C21—C20—H20	120.4
C8—C6—C5	112.51 (7)	C20—C21—C16	120.84 (8)
C7—C6—C5	107.82 (7)	C20—C21—H21	119.6
C1—C6—C5	108.58 (6)	C16—C21—H21	119.6
C6—C7—H7A	109.5	C23—C22—C2	111.69 (7)
C6—C7—H7B	109.5	C23—C22—H22A	109.3
H7A—C7—H7B	109.5	C2—C22—H22A	109.3
C6—C7—H7C	109.5	C23—C22—H22B	109.3
H7A—C7—H7C	109.5	C2—C22—H22B	109.3
H7B—C7—H7C	109.5	H22A—C22—H22B	107.9
C9—C8—C11	105.92 (7)	C24—C23—C22	124.67 (9)
C9—C8—C6	127.43 (7)	C24—C23—H23	117.7
C11—C8—C6	126.61 (7)	C22—C23—H23	117.7
N1—C9—C8	110.45 (8)	C23—C24—H24A	120.0
N1—C9—H9	124.8	C23—C24—H24B	120.0
C8—C9—H9	124.8	H24A—C24—H24B	120.0
O1—C1—C2—C16	109.70 (8)	N1—C10—C11—C8	-0.94 (9)
C6—C1—C2—C16	-72.94 (8)	C15—C10—C11—C8	176.29 (8)
O1—C1—C2—C3	-127.12 (8)	C9—C8—C11—C12	177.59 (9)
C6—C1—C2—C3	50.24 (8)	C6—C8—C11—C12	-4.63 (14)
O1—C1—C2—C22	-9.44 (10)	C9—C8—C11—C10	0.89 (9)
C6—C1—C2—C22	167.92 (7)	C6—C8—C11—C10	178.67 (7)
C16—C2—C3—C4	65.90 (9)	C10—C11—C12—C13	0.94 (12)
C1—C2—C3—C4	-54.99 (8)	C8—C11—C12—C13	-175.46 (9)
C22—C2—C3—C4	-172.90 (7)	C11—C12—C13—C14	-0.32 (14)

C2—C3—C4—C5	60.21 (9)	C12—C13—C14—C15	-0.29 (15)
C3—C4—C5—C6	-55.83 (9)	C13—C14—C15—C10	0.23 (15)
O1—C1—C6—C8	-105.94 (8)	N1—C10—C15—C14	177.02 (9)
C2—C1—C6—C8	76.69 (8)	C11—C10—C15—C14	0.44 (14)
O1—C1—C6—C7	13.42 (10)	C3—C2—C16—C17	-166.25 (7)
C2—C1—C6—C7	-163.95 (7)	C1—C2—C16—C17	-47.02 (9)
O1—C1—C6—C5	130.77 (8)	C22—C2—C16—C17	72.45 (9)
C2—C1—C6—C5	-46.60 (9)	C3—C2—C16—C21	16.54 (10)
C4—C5—C6—C8	-74.32 (9)	C1—C2—C16—C21	135.77 (8)
C4—C5—C6—C7	165.49 (7)	C22—C2—C16—C21	-104.77 (9)
C4—C5—C6—C1	47.24 (9)	C21—C16—C17—C18	-3.80 (12)
C7—C6—C8—C9	115.78 (9)	C2—C16—C17—C18	178.85 (7)
C1—C6—C8—C9	-124.70 (9)	C16—C17—C18—C19	1.78 (13)
C5—C6—C8—C9	-3.75 (11)	C17—C18—C19—C20	1.18 (14)
C7—C6—C8—C11	-61.54 (10)	C17—C18—C19—Cl1	-178.95 (7)
C1—C6—C8—C11	57.98 (10)	C18—C19—C20—C21	-1.97 (15)
C5—C6—C8—C11	178.93 (7)	Cl1—C19—C20—C21	178.16 (8)
C10—N1—C9—C8	-0.07 (10)	C19—C20—C21—C16	-0.16 (14)
C11—C8—C9—N1	-0.52 (10)	C17—C16—C21—C20	2.96 (13)
C6—C8—C9—N1	-178.28 (7)	C2—C16—C21—	-179.80 (8)

		C20	
C9—N1—C10—C15	-176.34 (9)	C16—C2—C22—C23	59.38 (9)
C9—N1—C10—C11	0.64 (10)	C3—C2—C22—C23	-64.58 (9)
N1—C10—C11—C12	-178.27 (8)	C1—C2—C22—C23	179.11 (7)
C15—C10—C11—C12	-1.03 (13)	C2—C22—C23—C24	110.70 (11)

Hydrogen-bond geometry (Å, º)

D—H···*A*	*D*—H	H···*A*	*D*···*A*	*D*—H···*A*
N1—H1*N*···O1^{i}	0.843 (17)	2.122 (17)	2.9469 (10)	166.3 (16)

Symmetry code: (i) *x*+1, *y*, *z*.

(±)-(2*S*,6*R*)-2-allyl-6-(1*H*-indol-3-yl)-6-methyl-2-(3-methylthiophen-2-yl)cyclohexanone (16l)

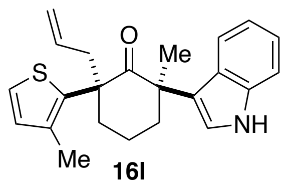

16l

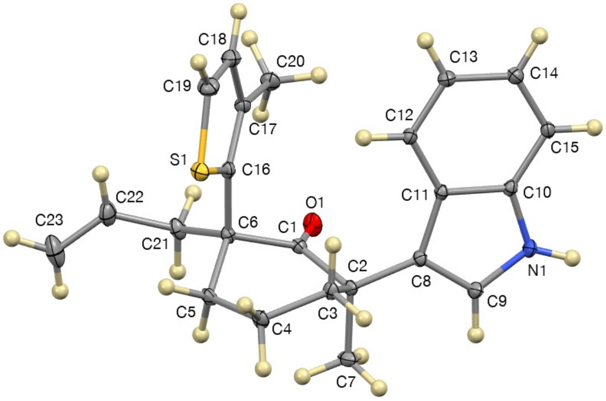

Sample Name: CCDC 2072240 / Malone11 (JAM7151)

Crystal data

$C_{23}H_{25}NOS$	D_x = 1.280 Mg m^{-3}
M_r = 363.50	Mo *K*α radiation, λ = 0.71073 Å
Orthorhombic, *Pbca*	Cell parameters from 9883 reflections
a = 14.4511 (8) Å	θ = 2.8–36.0°
b = 8.2265 (5) Å	μ = 0.18 mm^{-1}
c = 31.7402 (17) Å	*T* = 90 K
V = 3773.3 (4) Å^3	Lath, colourless
Z = 8	0.25 × 0.17 × 0.05 mm
F(000) = 1552	

Data collection

Bruker Kappa APEX-II DUO diffractometer	9145 independent reflections
Radiation source: fine-focus sealed tube	7094 reflections with *I* > 2σ(*I*)
TRIUMPH curved graphite monochromator	R_{int} = 0.053
ϕ and ω scans	$θ_{max}$ = 36.3°, $θ_{min}$ = 1.9°
Absorption correction: multi-scan *SADABS* (Sheldrick, 2004)	*h* = -23 – 24

T_{min} = 0.908, T_{max} = 0.991	k = -12 – 13
53537 measured reflections	l = -52 – 52

Refinement

Refinement on F^2	0 restraints
Least-squares matrix: full	Hydrogen site location: mixed
$R[F^2 > 2\sigma(F^2)]$ = 0.044	H atoms treated by a mixture of independent and constrained refinement
$wR(F^2)$ = 0.113	$w = 1/[\sigma^2(F_o^2) + (0.0535P)^2 + 1.0833P]$ where $P = (F_o^2 + 2F_c^2)/3$
S = 1.05	$(\Delta/\sigma)_{max}$ = 0.001
9145 reflections	$\Delta\rho_{max}$ = 0.61 e Å^{-3}
240 parameters	$\Delta\rho_{min}$ = -0.30 e Å^{-3}

Fractional atomic coordinates and isotropic or equivalent isotropic displacement parameters (Å^2) for (Malone11)

	x	y	z	U_{iso}*/U_{eq}
S1	0.62527 (2)	0.33869 (3)	0.54993 (2)	0.01381 (5)
O1	0.40811 (5)	0.37396 (9)	0.66589 (2)	0.01664 (14)
N1	0.52967 (6)	0.77135 (10)	0.74832 (2)	0.01306 (14)
H1N	0.5251 (10)	0.8341 (18)	0.7713 (4)	0.016*
C1	0.44079 (6)	0.44872 (11)	0.63647 (3)	0.01020 (14)
C2	0.46315 (6)	0.63079 (11)	0.64105 (3)	0.01005 (14)
C3	0.52961 (6)	0.68457 (11)	0.60575 (3)	0.01169 (15)
H3A	0.542011	0.802379	0.608704	0.014*
H3B	0.589146	0.626457	0.609093	0.014*
C4	0.49139 (7)	0.65152 (11)	0.56145 (3)	0.01362 (16)
H4A	0.543453	0.650827	0.541174	0.016*
H4B	0.449368	0.741426	0.553457	0.016*
C5	0.43843 (6)	0.48920 (12)	0.55779 (3)	0.01245 (15)
H5A	0.455121	0.438055	0.530593	0.015*
H5B	0.371374	0.513554	0.556848	0.015*
C6	0.45545 (6)	0.36382 (11)	0.59326 (3)	0.01052 (14)

C7	0.36930 (7)	0.71978 (13)	0.63687 (3)	0.01623 (17)
H7A	0.327937	0.684964	0.659618	0.024*
H7B	0.341245	0.693350	0.609611	0.024*
H7C	0.379248	0.837394	0.638748	0.024*
C8	0.50437 (6)	0.66359 (11)	0.68401 (3)	0.01002 (14)
C9	0.47126 (6)	0.76857 (12)	0.71412 (3)	0.01257 (15)
H9	0.415977	0.830332	0.711663	0.015*
C10	0.60173 (6)	0.66564 (11)	0.74135 (3)	0.01029 (14)
C11	0.58875 (6)	0.59600 (10)	0.70098 (3)	0.00922 (13)
C12	0.65466 (6)	0.48399 (11)	0.68622 (3)	0.01113 (14)
H12	0.647943	0.435745	0.659203	0.013*
C13	0.72964 (6)	0.44466 (12)	0.71155 (3)	0.01328 (15)
H13	0.774024	0.368337	0.701743	0.016*
C14	0.74103 (7)	0.51594 (12)	0.75151 (3)	0.01347 (15)
H14	0.793330	0.487778	0.768113	0.016*
C15	0.67738 (6)	0.62629 (11)	0.76704 (3)	0.01201 (15)
H15	0.684672	0.673696	0.794131	0.014*
C16	0.55258 (6)	0.29095 (11)	0.59184 (3)	0.01049 (14)
C17	0.59391 (7)	0.17758 (11)	0.61788 (3)	0.01250 (15)
C18	0.68413 (7)	0.13303 (13)	0.60330 (3)	0.01662 (17)
H18	0.722326	0.056588	0.617467	0.020*
C19	0.71015 (7)	0.21008 (14)	0.56732 (3)	0.01833 (18)
H19	0.767923	0.194557	0.553609	0.022*
C20	0.55762 (7)	0.10633 (12)	0.65830 (3)	0.01628 (17)
H20A	0.584140	0.165188	0.682277	0.024*
H20B	0.575054	-0.008610	0.660007	0.024*
H20C	0.490047	0.116001	0.658944	0.024*
C21	0.38253 (7)	0.22610 (12)	0.58908 (3)	0.01494 (16)
H21A	0.387642	0.153386	0.613824	0.018*
H21B	0.319939	0.275004	0.589476	0.018*
C22	0.39272 (8)	0.12666 (14)	0.54984 (3)	0.02032 (19)
H22	0.450237	0.073711	0.545190	0.024*
C23	0.32680 (10)	0.10740 (18)	0.52109 (4)	0.0293 (3)

H23A	0.268426	0.158612	0.524732	0.035*
H23B	0.338049	0.042424	0.496878	0.035*

Atomic displacement parameters (Å^2) for (Malone11)

	U^{11}	U^{22}	U^{33}	U^{12}	U^{13}	U^{23}
S1	0.01396 (10)	0.01713 (11)	0.01035 (9)	0.00127 (8)	0.00215 (7)	0.00106 (7)
O1	0.0223 (3)	0.0175 (3)	0.0102 (3)	-0.0049 (3)	0.0031 (2)	0.0022 (2)
N1	0.0162 (3)	0.0139 (3)	0.0091 (3)	0.0029 (3)	0.0001 (2)	-0.0031 (2)
C1	0.0096 (3)	0.0121 (3)	0.0089 (3)	0.0002 (3)	-0.0006 (2)	0.0005 (3)
C2	0.0110 (3)	0.0113 (3)	0.0079 (3)	0.0015 (3)	0.0005 (2)	0.0005 (3)
C3	0.0157 (4)	0.0111 (3)	0.0083 (3)	-0.0015 (3)	0.0010 (3)	0.0010 (3)
C4	0.0195 (4)	0.0131 (4)	0.0082 (3)	-0.0007 (3)	-0.0005 (3)	0.0023 (3)
C5	0.0147 (4)	0.0146 (4)	0.0080 (3)	0.0001 (3)	-0.0019 (3)	0.0013 (3)
C6	0.0115 (3)	0.0117 (3)	0.0084 (3)	-0.0014 (3)	-0.0009 (2)	0.0003 (3)
C7	0.0156 (4)	0.0199 (4)	0.0132 (4)	0.0072 (3)	-0.0012 (3)	0.0005 (3)
C8	0.0113 (3)	0.0106 (3)	0.0081 (3)	0.0004 (3)	0.0010 (2)	-0.0001 (3)
C9	0.0142 (4)	0.0139 (4)	0.0097 (3)	0.0033 (3)	0.0007 (3)	-0.0009 (3)
C10	0.0127 (3)	0.0095 (3)	0.0086 (3)	-0.0004 (3)	0.0009 (3)	-0.0006 (2)
C11	0.0105 (3)	0.0091 (3)	0.0081 (3)	-0.0006 (3)	0.0006 (2)	-0.0001 (2)
C12	0.0129 (4)	0.0105 (3)	0.0101 (3)	0.0009 (3)	0.0002 (3)	-0.0013 (3)
C13	0.0135 (4)	0.0127 (4)	0.0136 (3)	0.0025 (3)	-0.0011 (3)	-0.0007 (3)
C14	0.0139 (4)	0.0128 (4)	0.0137 (4)	0.0004 (3)	-0.0030	0.0001 (3)

					(3)	
C15	0.0152 (4)	0.0109 (3)	0.0099 (3)	-0.0010 (3)	-0.0025 (3)	-0.0008 (3)
C16	0.0122 (3)	0.0108 (3)	0.0084 (3)	-0.0006 (3)	0.0000 (3)	-0.0005 (3)
C17	0.0159 (4)	0.0107 (3)	0.0109 (3)	0.0006 (3)	-0.0016 (3)	-0.0003 (3)
C18	0.0172 (4)	0.0169 (4)	0.0158 (4)	0.0047 (3)	-0.0016 (3)	-0.0004 (3)
C19	0.0148 (4)	0.0230 (5)	0.0172 (4)	0.0053 (4)	0.0018 (3)	-0.0010 (4)
C20	0.0227 (4)	0.0136 (4)	0.0126 (4)	-0.0002 (3)	-0.0011 (3)	0.0033 (3)
C21	0.0152 (4)	0.0167 (4)	0.0129 (4)	-0.0056 (3)	-0.0024 (3)	0.0006 (3)
C22	0.0247 (5)	0.0173 (4)	0.0189 (4)	-0.0069 (4)	-0.0033 (3)	-0.0036 (3)
C23	0.0340 (6)	0.0351 (7)	0.0188 (5)	-0.0146 (5)	-0.0068 (4)	-0.0023 (4)

Geometric parameters (Å, º) for (Malone11)

S1—C19	1.7113 (11)	C9—H9	0.9500
S1—C16	1.7399 (9)	C10—C15	1.4019 (12)
O1—C1	1.2139 (11)	C10—C11	1.4161 (12)
N1—C10	1.3746 (12)	C11—C12	1.4055 (12)
N1—C9	1.3753 (12)	C12—C13	1.3873 (13)
N1—H1N	0.897 (14)	C12—H12	0.9500
C1—C2	1.5392 (13)	C13—C14	1.4072 (13)
C1—C6	1.5536 (12)	C13—H13	0.9500
C2—C8	1.5123 (12)	C14—C15	1.3831 (13)
C2—C3	1.5405 (12)	C14—H14	0.9500
C2—C7	1.5470 (13)	C15—H15	0.9500
C3—C4	1.5352 (12)	C16—C17	1.3818 (13)
C3—H3A	0.9900	C17—C18	1.4312 (14)
C3—H3B	0.9900	C17—C20	1.5048 (13)
C4—C5	1.5435 (13)	C18—C19	1.3592 (15)

C4—H4A	0.9900	C18—H18	0.9500
C4—H4B	0.9900	C19—H19	0.9500
C5—C6	1.5466 (12)	C20—H20A	0.9800
C5—H5A	0.9900	C20—H20B	0.9800
C5—H5B	0.9900	C20—H20C	0.9800
C6—C16	1.5270 (12)	C21—C22	1.4973 (14)
C6—C21	1.5529 (13)	C21—H21A	0.9900
C7—H7A	0.9800	C21—H21B	0.9900
C7—H7B	0.9800	C22—C23	1.3287 (16)
C7—H7C	0.9800	C22—H22	0.9500
C8—C9	1.3740 (12)	C23—H23A	0.9500
C8—C11	1.4442 (12)	C23—H23B	0.9500
C19—S1—C16	92.67 (5)	N1—C10—C15	130.03 (8)
C10—N1—C9	109.10 (7)	N1—C10—C11	107.54 (8)
C10—N1—H1N	123.4 (9)	C15—C10—C11	122.43 (8)
C9—N1—H1N	127.4 (9)	C12—C11—C10	118.48 (8)
O1—C1—C2	120.13 (8)	C12—C11—C8	134.46 (8)
O1—C1—C6	120.30 (8)	C10—C11—C8	107.06 (7)
C2—C1—C6	119.49 (7)	C13—C12—C11	119.29 (8)
C8—C2—C1	109.97 (7)	C13—C12—H12	120.4
C8—C2—C3	111.04 (7)	C11—C12—H12	120.4
C1—C2—C3	109.98 (7)	C12—C13—C14	121.10 (9)
C8—C2—C7	109.76 (7)	C12—C13—H13	119.5
C1—C2—C7	105.56 (7)	C14—C13—H13	119.5
C3—C2—C7	110.39 (7)	C15—C14—C13	121.13 (8)
C4—C3—C2	113.02 (8)	C15—C14—H14	119.4
C4—C3—H3A	109.0	C13—C14—H14	119.4
C2—C3—H3A	109.0	C14—C15—C10	117.57 (8)
C4—C3—H3B	109.0	C14—C15—H15	121.2
C2—C3—H3B	109.0	C10—C15—H15	121.2
H3A—C3—H3B	107.8	C17—C16—C6	130.14 (8)
C3—C4—C5	113.62 (7)	C17—C16—S1	110.41 (7)

C3—C4—H4A	108.8	C6—C16—S1	119.26 (6)
C5—C4—H4A	108.8	C16—C17—C18	111.91 (8)
C3—C4—H4B	108.8	C16—C17—C20	128.48 (9)
C5—C4—H4B	108.8	C18—C17—C20	119.57 (8)
H4A—C4—H4B	107.7	C19—C18—C17	113.86 (9)
C4—C5—C6	116.32 (7)	C19—C18—H18	123.1
C4—C5—H5A	108.2	C17—C18—H18	123.1
C6—C5—H5A	108.2	C18—C19—S1	111.15 (8)
C4—C5—H5B	108.2	C18—C19—H19	124.4
C6—C5—H5B	108.2	S1—C19—H19	124.4
H5A—C5—H5B	107.4	C17—C20—H20A	109.5
C16—C6—C5	112.74 (7)	C17—C20—H20B	109.5
C16—C6—C21	109.57 (8)	H20A—C20—H20B	109.5
C5—C6—C21	108.45 (7)	C17—C20—H20C	109.5
C16—C6—C1	109.14 (7)	H20A—C20—H20C	109.5
C5—C6—C1	108.73 (7)	H20B—C20—H20C	109.5
C21—C6—C1	108.11 (7)	C22—C21—C6	113.76 (8)
C2—C7—H7A	109.5	C22—C21—H21A	108.8
C2—C7—H7B	109.5	C6—C21—H21A	108.8
H7A—C7—H7B	109.5	C22—C21—H21B	108.8
C2—C7—H7C	109.5	C6—C21—H21B	108.8
H7A—C7—H7C	109.5	H21A—C21—H21B	107.7
H7B—C7—H7C	109.5	C23—C22—C21	124.47 (12)
C9—C8—C11	106.07 (7)	C23—C22—H22	117.8
C9—C8—C2	127.02 (8)	C21—C22—H22	117.8
C11—C8—C2	126.87 (7)	C22—C23—H23A	120.0
C8—C9—N1	110.22 (8)	C22—C23—H23B	120.0
C8—C9—H9	124.9	H23A—C23—H23B	120.0
N1—C9—H9	124.9		

O1—C1—C2—C8	40.12 (11)	N1—C10—C11—C8	-0.67 (10)
C6—C1—C2—C8	-143.12 (7)	C15—C10—C11—C8	179.71 (8)
O1—C1—C2—C3	162.70 (8)	C9—C8—C11—C12	-179.80 (10)
C6—C1—C2—C3	-20.53 (10)	C2—C8—C11—C12	-2.18 (16)
O1—C1—C2—C7	-78.22 (10)	C9—C8—C11—C10	0.14 (10)
C6—C1—C2—C7	98.55 (8)	C2—C8—C11—C10	177.76 (8)
C8—C2—C3—C4	179.19 (7)	C10—C11—C12—C13	0.33 (13)
C1—C2—C3—C4	57.24 (10)	C8—C11—C12—C13	-179.73 (10)
C7—C2—C3—C4	-58.85 (10)	C11—C12—C13—C14	-0.50 (14)
C2—C3—C4—C5	-38.55 (11)	C12—C13—C14—C15	0.66 (15)
C3—C4—C5—C6	-17.44 (12)	C13—C14—C15—C10	-0.62 (14)
C4—C5—C6—C16	-69.81 (10)	N1—C10—C15—C14	-179.05 (9)
C4—C5—C6—C21	168.68 (8)	C11—C10—C15—C14	0.47 (13)
C4—C5—C6—C1	51.35 (10)	C5—C6—C16—C17	179.47 (9)
O1—C1—C6—C16	-91.12 (10)	C21—C6—C16—C17	-59.65 (12)
C2—C1—C6—C16	92.12 (9)	C1—C6—C16—C17	58.54 (12)
O1—C1—C6—C5	145.53 (9)	C5—C6—C16—S1	-6.03 (10)
C2—C1—C6—C5	-31.23 (10)	C21—C6—C16—S1	114.85 (7)
O1—C1—C6—C21	27.98 (11)	C1—C6—C16—S1	-126.96 (7)
C2—C1—C6—C21	-148.78 (8)	C19—S1—C16—C17	-0.51 (8)

C1—C2—C8—C9	-122.08 (10)	C19—S1—C16—C6	-176.03 (7)
C3—C2—C8—C9	115.96 (10)	C6—C16—C17—C18	175.32 (9)
C7—C2—C8—C9	-6.37 (13)	S1—C16—C17—C18	0.44 (10)
C1—C2—C8—C11	60.78 (11)	C6—C16—C17—C20	-7.34 (16)
C3—C2—C8—C11	-61.18 (11)	S1—C16—C17—C20	177.78 (8)
C7—C2—C8—C11	176.49 (9)	C16—C17—C18—C19	-0.10 (13)
C11—C8—C9—N1	0.45 (11)	C20—C17—C18—C19	-177.71 (9)
C2—C8—C9—N1	-177.17 (8)	C17—C18—C19—S1	-0.29 (12)
C10—N1—C9—C8	-0.90 (11)	C16—S1—C19—C18	0.46 (9)
C9—N1—C10—C15	-179.46 (9)	C16—C6—C21—C22	-57.43 (10)
C9—N1—C10—C11	0.96 (10)	C5—C6—C21—C22	66.01 (11)
N1—C10—C11—C12	179.28 (8)	C1—C6—C21—C22	-176.26 (8)
C15—C10—C11—C12	-0.33 (13)	C6—C21—C22—C23	-122.38 (12)

Hydrogen-bond geometry (Å, º) for (Malone11)

D—H···*A*	*D*—H	H···*A*	*D*···*A*	*D*—H···*A*
N1—H1*N*···O1^{i}	0.897 (14)	2.238 (14)	2.9891 (11)	141.1 (12)

Symmetry code: (i) -*x*+1, *y*+1/2, -*z*+3/2.

Document origin: *publCIF* [Westrip, S. P. (2010). *J. Apply. Cryst.*, **43**, 920-925].

(±)-(2*R*,6*R*)-2-(1*H*-indol-3-yl)-2-methyl-6-(2-methylallyl)-6-phenylcyclohexanone (18a)

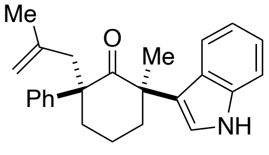

18a

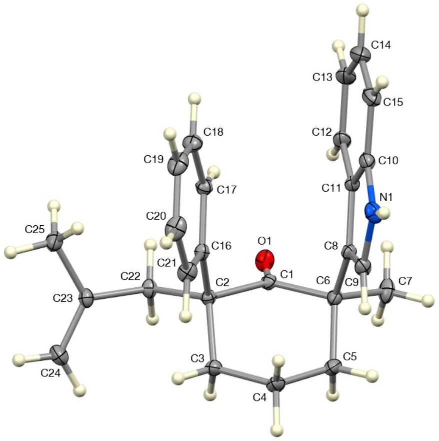

Sample Name: CCDC 2072241 / Malone13 (JAM7208)

Crystal data

$C_{25}H_{27}NO$	$F(000) = 768$
$M_r = 357.47$	$D_x = 1.230$ Mg m^{-3}
Monoclinic, $P2_1/c$	Mo $K\alpha$ radiation, $\lambda = 0.71073$ Å
$a = 8.783$ (1) Å	Cell parameters from 9892 reflections
$b = 16.3404$ (19) Å	$\theta = 2.4$–$41.1°$
$c = 13.6725$ (15) Å	$\mu = 0.07$ mm^{-1}
$\beta = 100.283$ (6)°	$T = 90$ K
$V = 1930.7$ (4) Å^3	Lath fragment, colourless
$Z = 4$	0.43 × 0.32 × 0.08 mm

Data collection

Bruker Kappa APEX-II DUO diffractometer	12870 independent reflections
Radiation source: fine-focus sealed tube	10328 reflections with $I > 2\sigma(I)$
TRIUMPH curved graphite monochromator	$R_{int} = 0.046$
ϕ and ω scans	$\theta_{max} = 41.3°$, $\theta_{min} = 2.0°$
Absorption correction: multi-scan *SADABS* (Sheldrick, 2004)	$h = -15 - 16$

T_{min} = 0.912, T_{max} = 0.994	k = -30 – 25
76658 measured reflections	l = -25 – 24

Refinement

Refinement on F^2	0 restraints
Least-squares matrix: full	Hydrogen site location: mixed
$R[F^2 > 2\sigma(F^2)]$ = 0.043	H atoms treated by a mixture of independent and constrained refinement
$wR(F^2)$ = 0.129	$w = 1/[\sigma^2(F_o^2) + (0.0682P)^2 + 0.2364P]$ where $P = (F_o^2 + 2F_c^2)/3$
S = 1.04	$(\Delta/\sigma)_{max}$ = 0.001
12870 reflections	$\Delta\rho_{max}$ = 0.72 e Å^{-3}
249 parameters	$\Delta\rho_{min}$ = -0.26 e Å^{-3}

Fractional atomic coordinates and isotropic or equivalent isotropic displacement parameters (Å^2) for (Malone13)

	x	y	z	U_{iso}*/U_{eq}
O1	0.69364 (5)	0.12144 (3)	0.50907 (3)	0.01706 (7)
N1	0.73472 (6)	0.34947 (3)	0.80723 (4)	0.01645 (8)
H1N	0.7170 (12)	0.3798 (6)	0.8571 (8)	0.020*
C1	0.63711 (6)	0.13973 (3)	0.58131 (4)	0.01175 (7)
C2	0.45976 (6)	0.15150 (3)	0.57207 (3)	0.01105 (7)
C3	0.40735 (6)	0.09252 (3)	0.64805 (4)	0.01514 (8)
H3A	0.296120	0.101819	0.648597	0.018*
H3B	0.419102	0.035526	0.625984	0.018*
C4	0.49738 (7)	0.10226 (4)	0.75374 (4)	0.01801 (9)
H4A	0.460629	0.061396	0.797675	0.022*
H4B	0.478563	0.157458	0.778956	0.022*
C5	0.67051 (7)	0.09057 (4)	0.75609 (4)	0.01828 (9)
H5A	0.688970	0.032913	0.739264	0.022*
H5B	0.725819	0.100366	0.824750	0.022*
C6	0.74094 (6)	0.14675 (3)	0.68480 (4)	0.01337 (8)
C7	0.90592 (7)	0.11648 (4)	0.68069 (5)	0.02008 (10)

H7A	0.950502	0.150004	0.633470	0.030*
H7B	0.901852	0.059196	0.659140	0.030*
H7C	0.970287	0.120990	0.746831	0.030*
C8	0.74923 (6)	0.23492 (3)	0.71793 (3)	0.01160 (7)
C9	0.71007 (6)	0.26653 (3)	0.80290 (4)	0.01452 (8)
H9	0.671366	0.235443	0.851843	0.017*
C10	0.79165 (6)	0.37339 (3)	0.72456 (4)	0.01465 (8)
C11	0.80421 (6)	0.30282 (3)	0.66674 (3)	0.01231 (7)
C12	0.85814 (6)	0.31124 (4)	0.57631 (4)	0.01738 (9)
H12	0.869472	0.264686	0.536623	0.021*
C13	0.89433 (8)	0.38863 (5)	0.54633 (5)	0.02412 (12)
H13	0.929409	0.395010	0.484943	0.029*
C14	0.88025 (8)	0.45783 (4)	0.60475 (6)	0.02650 (13)
H14	0.905635	0.510184	0.582059	0.032*
C15	0.82994 (7)	0.45128 (4)	0.69506 (5)	0.02176 (11)
H15	0.821924	0.498006	0.735180	0.026*
C16	0.42055 (5)	0.24132 (3)	0.58732 (3)	0.01095 (7)
C17	0.47836 (6)	0.30062 (3)	0.53009 (4)	0.01339 (8)
H17	0.548381	0.284774	0.488014	0.016*
C18	0.43571 (7)	0.38215 (3)	0.53340 (4)	0.01776 (9)
H18	0.476833	0.421583	0.494177	0.021*
C19	0.33242 (8)	0.40610 (4)	0.59437 (4)	0.02036 (10)
H19	0.302315	0.461814	0.596581	0.024*
C20	0.27365 (7)	0.34800 (4)	0.65197 (4)	0.02006 (10)
H20	0.203726	0.364129	0.693985	0.024*
C21	0.31699 (6)	0.26617 (3)	0.64826 (4)	0.01575 (8)
H21	0.275753	0.226833	0.687552	0.019*
C22	0.38064 (6)	0.12636 (3)	0.46512 (4)	0.01315 (8)
H22A	0.428198	0.158054	0.416682	0.016*
H22B	0.402062	0.067741	0.455264	0.016*
C23	0.20693 (6)	0.13961 (3)	0.44269 (4)	0.01388 (8)
C24	0.11008 (7)	0.08049 (4)	0.45943 (5)	0.02106 (10)
H24A	0.001467	0.088935	0.443378	0.025*

H24B	0.149706	0.029923	0.487402	0.025*
C25	0.14642 (7)	0.22018 (4)	0.39841 (4)	0.01774 (9)
H25A	0.034534	0.216093	0.374970	0.027*
H25B	0.197645	0.233731	0.342421	0.027*
H25C	0.167694	0.263141	0.449015	0.027*

Atomic displacement parameters (Å^2) for (Malone13)

	U^{11}	U^{22}	U^{33}	U^{12}	U^{13}	U^{23}
O1	0.01518 (16)	0.02234 (19)	0.01415 (15)	0.00339 (13)	0.00396 (12)	-0.00166 (13)
N1	0.01591 (18)	0.01832 (19)	0.01539 (17)	-0.00193 (14)	0.00352 (14)	-0.00564 (14)
C1	0.01217 (17)	0.01089 (16)	0.01200 (16)	0.00095 (13)	0.00159 (13)	0.00037 (13)
C2	0.01157 (17)	0.01114 (17)	0.01039 (15)	-0.00006 (13)	0.00183 (12)	-0.00031 (12)
C3	0.0166 (2)	0.01475 (19)	0.01369 (18)	-0.00376 (15)	0.00175 (14)	0.00184 (14)
C4	0.0207 (2)	0.0200 (2)	0.01285 (18)	-0.00553 (18)	0.00145 (15)	0.00472 (16)
C5	0.0205 (2)	0.0159 (2)	0.0164 (2)	-0.00206 (17)	-0.00206 (16)	0.00584 (16)
C6	0.01322 (18)	0.01341 (18)	0.01256 (17)	0.00128 (14)	-0.00017 (13)	0.00097 (13)
C7	0.0149 (2)	0.0214 (2)	0.0221 (2)	0.00640 (17)	-0.00162 (17)	-0.00245 (18)
C8	0.01125 (17)	0.01364 (17)	0.00977 (15)	-0.00061 (13)	0.00150 (12)	0.00035 (13)
C9	0.01433 (19)	0.0188 (2)	0.01063 (16)	-0.00269 (15)	0.00277 (13)	-0.00113 (14)
C10	0.01227 (18)	0.01441 (19)	0.01629 (18)	-0.00170 (14)	-0.00013 (14)	-0.00073 (15)
C11	0.01082 (17)	0.01489 (18)	0.01089 (16)	-0.00185 (13)	0.00101 (12)	0.00104 (13)
C12	0.0148 (2)	0.0252 (2)	0.01228 (17)	-0.00525 (17)	0.00261 (14)	0.00225 (16)
C13	0.0201 (3)	0.0322 (3)	0.0193 (2)	-0.0101	0.00134	0.0095 (2)

				(2)	(18)	
C14	0.0222 (3)	0.0230 (3)	0.0316 (3)	-0.0088 (2)	-0.0027 (2)	0.0112 (2)
C15	0.0179 (2)	0.0150 (2)	0.0300 (3)	-0.00353 (17)	-0.00219 (19)	0.00141 (19)
C16	0.01114 (17)	0.01185 (17)	0.00978 (15)	0.00059 (13)	0.00167 (12)	-0.00127 (12)
C17	0.01330 (18)	0.01258 (18)	0.01433 (17)	-0.00023 (14)	0.00256 (14)	-0.00007 (14)
C18	0.0206 (2)	0.01213 (19)	0.0194 (2)	-0.00013 (16)	0.00043 (17)	0.00006 (16)
C19	0.0250 (3)	0.0147 (2)	0.0195 (2)	0.00585 (18)	-0.00102 (18)	-0.00440 (17)
C20	0.0220 (2)	0.0215 (2)	0.0169 (2)	0.00777 (19)	0.00387 (17)	-0.00493 (17)
C21	0.0167 (2)	0.0182 (2)	0.01327 (18)	0.00296 (16)	0.00517 (15)	-0.00144 (15)
C22	0.01333 (18)	0.01403 (18)	0.01177 (16)	0.00048 (14)	0.00141 (13)	-0.00279 (14)
C23	0.01302 (18)	0.01522 (19)	0.01264 (17)	-0.00076 (14)	0.00019 (13)	-0.00234 (14)
C24	0.0168 (2)	0.0206 (2)	0.0242 (2)	-0.00555 (18)	-0.00061 (18)	0.00034 (19)
C25	0.0160 (2)	0.0187 (2)	0.0171 (2)	0.00166 (17)	-0.00080 (16)	0.00084 (16)

Geometric parameters (Å, º) for (Malone13)

O1—C1	1.2193 (6)	C12—C13	1.3836 (9)
N1—C10	1.3719 (7)	C12—H12	0.9500
N1—C9	1.3722 (8)	C13—C14	1.4026 (12)
N1—H1N	0.879 (10)	C13—H13	0.9500
C1—C6	1.5447 (7)	C14—C15	1.3879 (11)
C1—C2	1.5521 (7)	C14—H14	0.9500
C2—C16	1.5302 (7)	C15—H15	0.9500
C2—C3	1.5462 (7)	C16—C17	1.3968 (7)
C2—C22	1.5579 (7)	C16—C21	1.3989 (7)

C3—C4	1.5267 (8)	C17—C18	1.3867 (8)
C3—H3A	0.9900	C17—H17	0.9500
C3—H3B	0.9900	C18—C19	1.3933 (9)
C4—C5	1.5272 (9)	C18—H18	0.9500
C4—H4A	0.9900	C19—C20	1.3903 (10)
C4—H4B	0.9900	C19—H19	0.9500
C5—C6	1.5463 (8)	C20—C21	1.3936 (8)
C5—H5A	0.9900	C20—H20	0.9500
C5—H5B	0.9900	C21—H21	0.9500
C6—C8	1.5082 (7)	C22—C23	1.5171 (8)
C6—C7	1.5416 (8)	C22—H22A	0.9900
C7—H7A	0.9800	C22—H22B	0.9900
C7—H7B	0.9800	C23—C24	1.3338 (8)
C7—H7C	0.9800	C23—C25	1.5058 (8)
C8—C9	1.3702 (7)	C24—H24A	0.9500
C8—C11	1.4404 (7)	C24—H24B	0.9500
C9—H9	0.9500	C25—H25A	0.9800
C10—C15	1.3943 (8)	C25—H25B	0.9800
C10—C11	1.4134 (8)	C25—H25C	0.9800
C11—C12	1.4075 (7)		
C10—N1—C9	108.89 (4)	C12—C11—C10	118.92 (5)
C10—N1—H1N	128.3 (7)	C12—C11—C8	134.16 (5)
C9—N1—H1N	122.8 (7)	C10—C11—C8	106.88 (4)
O1—C1—C6	120.07 (5)	C13—C12—C11	118.74 (6)
O1—C1—C2	120.82 (4)	C13—C12—H12	120.6
C6—C1—C2	119.03 (4)	C11—C12—H12	120.6
C16—C2—C3	113.90 (4)	C12—C13—C14	121.32 (6)
C16—C2—C1	110.78 (4)	C12—C13—H13	119.3
C3—C2—C1	106.43 (4)	C14—C13—H13	119.3
C16—C2—C22	107.90 (4)	C15—C14—C13	121.26 (6)
C3—C2—C22	109.26 (4)	C15—C14—H14	119.4
C1—C2—C22	108.47 (4)	C13—C14—H14	119.4

C4—C3—C2	113.89 (4)	C14—C15—C10	117.34 (6)
C4—C3—H3A	108.8	C14—C15—H15	121.3
C2—C3—H3A	108.8	C10—C15—H15	121.3
C4—C3—H3B	108.8	C17—C16—C21	118.18 (5)
C2—C3—H3B	108.8	C17—C16—C2	118.42 (4)
H3A—C3—H3B	107.7	C21—C16—C2	123.07 (4)
C3—C4—C5	110.63 (5)	C18—C17—C16	121.39 (5)
C3—C4—H4A	109.5	C18—C17—H17	119.3
C5—C4—H4A	109.5	C16—C17—H17	119.3
C3—C4—H4B	109.5	C17—C18—C19	119.86 (5)
C5—C4—H4B	109.5	C17—C18—H18	120.1
H4A—C4—H4B	108.1	C19—C18—H18	120.1
C4—C5—C6	114.92 (4)	C20—C19—C18	119.62 (5)
C4—C5—H5A	108.5	C20—C19—H19	120.2
C6—C5—H5A	108.5	C18—C19—H19	120.2
C4—C5—H5B	108.5	C19—C20—C21	120.20 (5)
C6—C5—H5B	108.5	C19—C20—H20	119.9
H5A—C5—H5B	107.5	C21—C20—H20	119.9
C8—C6—C7	108.80 (4)	C20—C21—C16	120.74 (5)
C8—C6—C1	109.57 (4)	C20—C21—H21	119.6
C7—C6—C1	110.36 (4)	C16—C21—H21	119.6
C8—C6—C5	112.40 (4)	C23—C22—C2	114.59 (4)
C7—C6—C5	108.48 (5)	C23—C22—H22A	108.6
C1—C6—C5	107.22 (4)	C2—C22—H22A	108.6
C6—C7—H7A	109.5	C23—C22—H22B	108.6
C6—C7—H7B	109.5	C2—C22—H22B	108.6
H7A—C7—H7B	109.5	H22A—C22—H22B	107.6
C6—C7—H7C	109.5	C24—C23—C25	120.81 (5)
H7A—C7—H7C	109.5	C24—C23—C22	120.74 (5)
H7B—C7—H7C	109.5	C25—C23—C22	118.43 (5)
C9—C8—C11	106.16 (5)	C23—C24—H24A	120.0
C9—C8—C6	127.50 (5)	C23—C24—H24B	120.0
C11—C8—C6	126.33 (4)	H24A—C24—	120.0

		H24B	
C8—C9—N1	110.32 (5)	C23—C25—H25A	109.5
C8—C9—H9	124.8	C23—C25—H25B	109.5
N1—C9—H9	124.8	H25A—C25—H25B	109.5
N1—C10—C15	129.83 (6)	C23—C25—H25C	109.5
N1—C10—C11	107.74 (5)	H25A—C25—H25C	109.5
C15—C10—C11	122.41 (5)	H25B—C25—H25C	109.5
O1—C1—C2—C16	-111.78 (5)	N1—C10—C11—C8	1.00 (6)
C6—C1—C2—C16	71.63 (5)	C15—C10—C11—C8	-177.66 (5)
O1—C1—C2—C3	123.92 (5)	C9—C8—C11—C12	-178.75 (6)
C6—C1—C2—C3	-52.68 (5)	C6—C8—C11—C12	2.62 (9)
O1—C1—C2—C22	6.47 (6)	C9—C8—C11—C10	-1.16 (5)
C6—C1—C2—C22	-170.13 (4)	C6—C8—C11—C10	-179.80 (5)
C16—C2—C3—C4	-68.83 (6)	C10—C11—C12—C13	-1.15 (8)
C1—C2—C3—C4	53.52 (6)	C8—C11—C12—C13	176.20 (6)
C22—C2—C3—C4	170.44 (5)	C11—C12—C13—C14	0.89 (9)
C2—C3—C4—C5	-57.06 (6)	C12—C13—C14—C15	0.22 (10)
C3—C4—C5—C6	55.12 (6)	C13—C14—C15—C10	-1.01 (9)
O1—C1—C6—C8	111.96 (5)	N1—C10—C15—C14	-177.62 (6)
C2—C1—C6—C8	-71.42 (5)	C11—C10—C15—C14	0.72 (9)
O1—C1—C6—C7	-7.82 (7)	C3—C2—C16—	172.73 (4)

		C17	
C2—C1—C6—C7	168.80 (4)	C1—C2—C16—C17	52.80 (6)
O1—C1—C6—C5	-125.79 (5)	C22—C2—C16—C17	-65.79 (5)
C2—C1—C6—C5	50.84 (6)	C3—C2—C16—C21	-14.01 (7)
C4—C5—C6—C8	70.54 (6)	C1—C2—C16—C21	-133.94 (5)
C4—C5—C6—C7	-169.11 (5)	C22—C2—C16—C21	107.47 (5)
C4—C5—C6—C1	-49.93 (6)	C21—C16—C17—C18	0.34 (8)
C7—C6—C8—C9	-116.54 (6)	C2—C16—C17—C18	173.93 (5)
C1—C6—C8—C9	122.73 (5)	C16—C17—C18—C19	-0.37 (8)
C5—C6—C8—C9	3.63 (7)	C17—C18—C19—C20	0.41 (9)
C7—C6—C8—C11	61.81 (6)	C18—C19—C20—C21	-0.42 (9)
C1—C6—C8—C11	-58.92 (6)	C19—C20—C21—C16	0.40 (9)
C5—C6—C8—C11	-178.03 (5)	C17—C16—C21—C20	-0.35 (8)
C11—C8—C9—N1	0.91 (6)	C2—C16—C21—C20	-173.62 (5)
C6—C8—C9—N1	179.53 (5)	C16—C2—C22—C23	-56.32 (5)
C10—N1—C9—C8	-0.31 (6)	C3—C2—C22—C23	67.99 (6)
C9—N1—C10—C15	178.07 (6)	C1—C2—C22—C23	-176.38 (4)
C9—N1—C10—C11	-0.45 (6)	C2—C22—C23—C24	-90.63 (6)
N1—C10—C11—C12	179.02 (5)	C2—C22—C23—C25	91.07 (6)
C15—C10—C11—C12	0.36 (8)		

Hydrogen-bond geometry (Å, º) for (Malone13)

D—H···*A*	*D*—H	H···*A*	*D*···*A*	*D*—H···*A*
N1—H1*N*···O1^{i}	0.879 (10)	2.125 (10)	2.8858 (7)	144.6 (9)

Symmetry code: (i) *x*, -*y*+1/2, *z*+1/2.

(±)-(2*R*,6*R*)-2-allyl-6-ethyl-6-(1*H*-indol-3-yl)-2-phenylcyclohexanone (*cis*-20)

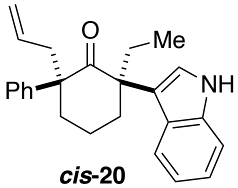

***cis*-20**

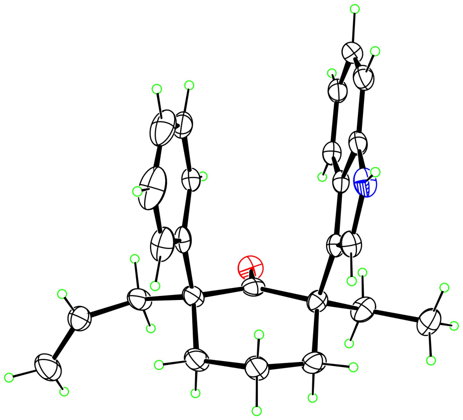

Sample Name: CCDC 2072242 / Malone12 (JAM7191-F1)

Crystal data

$C_{25}H_{27}NO$	D_x = 1.175 Mg m^{-3}
M_r = 357.47	Cu *K*α radiation, λ = 1.54184 Å
Orthorhombic, $P2_12_12_1$	Cell parameters from 8430 reflections
a = 8.0496 (2) Å	θ = 5.0–68.0°
b = 14.2007 (5) Å	μ = 0.54 mm^{-1}
c = 17.6818 (5) Å	*T* = 90 K
V = 2021.21 (10) Å^3	Needle, colourless
Z = 4	0.21 × 0.05 × 0.03 mm
F(000) = 768	

Data collection

Bruker Kappa APEX-II DUO diffractometer	3671 independent reflections
Radiation source: IμS microfocus	3481 reflections with $I > 2\sigma(I)$
QUAZAR multilayer optics monochromator	R_{int} = 0.038
ϕ and ω scans	θ_{max} = 68.8°, θ_{min} = 4.0°
Absorption correction: multi-scan *SADABS* (Sheldrick, 2004)	*h* = -8 – 9

T_{min} = 0.904, T_{max} = 0.984	k = -17 – 17
21140 measured reflections	l = -21 – 18

Refinement

Refinement on F^2	Hydrogen site location: mixed
Least-squares matrix: full	H atoms treated by a mixture of independent and constrained refinement
$R[F^2 > 2\sigma(F^2)]$ = 0.038	$w = 1/[\sigma^2(F_o^2) + (0.0534P)^2 + 0.5113P]$ where $P = (F_o^2 + 2F_c^2)/3$
$wR(F^2)$ = 0.098	$(\Delta/\sigma)_{max}$ < 0.001
S = 1.03	$\Delta\rho_{max}$ = 0.50 e Å^{-3}
3671 reflections	$\Delta\rho_{min}$ = -0.17 e Å^{-3}
249 parameters	Absolute structure: Refined as an inversion twin.
0 restraints	Absolute structure parameter: 0.5 (4)

Fractional atomic coordinates and isotropic or equivalent isotropic displacement parameters (Å^2) for (Malone12)

	x	y	z	U_{iso}*/U_{eq}
O1	0.37700 (18)	0.49243 (12)	0.61460 (9)	0.0337 (4)
N1	1.0284 (2)	0.50079 (16)	0.58114 (11)	0.0331 (5)
H1N	1.137 (4)	0.5032 (19)	0.5913 (16)	0.040*
C1	0.4920 (3)	0.46250 (15)	0.65213 (13)	0.0265 (5)
C2	0.5151 (3)	0.49557 (16)	0.73426 (12)	0.0287 (5)
C3	0.5565 (4)	0.40960 (19)	0.78336 (15)	0.0403 (6)
H3A	0.583165	0.431229	0.835190	0.048*
H3B	0.457250	0.368644	0.786541	0.048*
C4	0.7017 (3)	0.35199 (18)	0.75328 (14)	0.0397 (6)
H4A	0.802663	0.391727	0.751058	0.048*
H4B	0.724122	0.298471	0.787677	0.048*
C5	0.6607 (3)	0.31528 (17)	0.67474 (15)	0.0365 (6)
H5A	0.566104	0.271018	0.678818	0.044*
H5B	0.757311	0.279322	0.655611	0.044*

C6	0.6161 (3)	0.39211 (15)	0.61633 (13)	0.0269 (5)
C7	0.5325 (3)	0.34596 (17)	0.54689 (16)	0.0363 (6)
H7A	0.431336	0.312341	0.563824	0.044*
H7B	0.497326	0.396112	0.511440	0.044*
C8	0.6435 (4)	0.27725 (18)	0.50502 (15)	0.0421 (6)
H8A	0.743444	0.310028	0.487407	0.063*
H8B	0.583474	0.251400	0.461505	0.063*
H8C	0.675486	0.225907	0.539080	0.063*
C9	0.7662 (3)	0.44830 (15)	0.59205 (12)	0.0241 (4)
C10	0.9271 (3)	0.43570 (17)	0.61446 (13)	0.0296 (5)
H10	0.963521	0.388228	0.648493	0.036*
C11	0.9345 (3)	0.55902 (16)	0.53648 (13)	0.0286 (5)
C12	0.7681 (3)	0.52723 (15)	0.54045 (11)	0.0239 (4)
C13	0.6473 (3)	0.57619 (16)	0.49869 (12)	0.0280 (5)
H13	0.534960	0.555735	0.498938	0.034*
C14	0.6934 (3)	0.65393 (17)	0.45749 (13)	0.0350 (6)
H14	0.611948	0.686957	0.429054	0.042*
C15	0.8596 (4)	0.68559 (18)	0.45666 (14)	0.0412 (6)
H15	0.887660	0.740666	0.428977	0.049*
C16	0.9810 (3)	0.63798 (18)	0.49525 (14)	0.0374 (6)
H16	1.093354	0.658334	0.493842	0.045*
C17	0.6476 (3)	0.57321 (16)	0.73657 (12)	0.0281 (5)
C18	0.6411 (3)	0.64608 (16)	0.68368 (12)	0.0277 (5)
H18	0.554526	0.646242	0.647176	0.033*
C19	0.7568 (3)	0.71804 (19)	0.68273 (15)	0.0401 (6)
H19	0.750569	0.765945	0.645330	0.048*
C20	0.8810 (4)	0.7200 (2)	0.7362 (2)	0.0544 (8)
H20	0.961474	0.768925	0.735761	0.065*
C21	0.8874 (4)	0.6504 (3)	0.7902 (2)	0.0589 (9)
H21	0.971717	0.652328	0.827697	0.071*
C22	0.7723 (4)	0.5771 (2)	0.79078 (15)	0.0439 (7)
H22	0.779244	0.529553	0.828449	0.053*
C23	0.3464 (3)	0.53725 (18)	0.76184 (14)	0.0352 (6)

H23A	0.323654	0.596236	0.733843	0.042*
H23B	0.256309	0.492161	0.749939	0.042*
C24	0.3445 (3)	0.55733 (18)	0.84397 (15)	0.0387 (6)
H24	0.425278	0.599770	0.863292	0.046*
C25	0.2374 (4)	0.5199 (2)	0.89224 (16)	0.0515 (8)
H25A	0.155102	0.477184	0.874724	0.062*
H25B	0.242886	0.535725	0.944383	0.062*

Atomic displacement parameters (Å^2) for (Malone12)

	U^{11}	U^{22}	U^{33}	U^{12}	U^{13}	U^{23}
O1	0.0170 (7)	0.0398 (9)	0.0443 (9)	0.0015 (7)	-0.0028 (7)	0.0011 (8)
N1	0.0159 (9)	0.0482 (12)	0.0353 (10)	-0.0011 (9)	-0.0002 (8)	-0.0079 (9)
C1	0.0176 (10)	0.0252 (11)	0.0368 (12)	-0.0025 (9)	0.0032 (9)	0.0057 (9)
C2	0.0254 (11)	0.0315 (12)	0.0292 (11)	0.0087 (10)	0.0059 (9)	0.0076 (9)
C3	0.0438 (15)	0.0376 (14)	0.0396 (13)	0.0171 (12)	0.0138 (11)	0.0144 (11)
C4	0.0463 (15)	0.0366 (13)	0.0362 (12)	0.0205 (12)	0.0097 (11)	0.0126 (10)
C5	0.0354 (13)	0.0265 (12)	0.0476 (14)	0.0071 (11)	0.0091 (11)	0.0060 (10)
C6	0.0214 (11)	0.0262 (11)	0.0330 (11)	0.0024 (9)	-0.0003 (9)	-0.0024 (9)
C7	0.0280 (12)	0.0326 (12)	0.0484 (14)	-0.0041 (10)	-0.0050 (11)	-0.0070 (11)
C8	0.0500 (16)	0.0341 (13)	0.0422 (14)	0.0049 (12)	-0.0047 (13)	-0.0083 (11)
C9	0.0198 (10)	0.0282 (11)	0.0241 (9)	0.0020 (9)	-0.0004 (9)	-0.0047 (8)
C10	0.0233 (11)	0.0375 (13)	0.0282 (11)	0.0055 (10)	-0.0011 (9)	-0.0026 (10)
C11	0.0243 (11)	0.0342 (12)	0.0273 (11)	-0.0041 (9)	0.0058 (9)	-0.0090 (10)
C12	0.0217	0.0273	0.0228 (9)	0.0001 (8)	0.0020 (9)	-0.0078

	(10)	(11)				(8)
C13	0.0278 (11)	0.0302 (11)	0.0259 (10)	0.0016 (10)	0.0008 (9)	-0.0061 (9)
C14	0.0466 (15)	0.0322 (12)	0.0264 (11)	0.0079 (11)	0.0062 (11)	-0.0013 (9)
C15	0.0598 (17)	0.0306 (12)	0.0333 (12)	-0.0060 (12)	0.0184 (13)	-0.0024 (10)
C16	0.0364 (13)	0.0384 (13)	0.0375 (13)	-0.0119 (11)	0.0126 (11)	-0.0126 (11)
C17	0.0253 (11)	0.0354 (12)	0.0236 (10)	0.0107 (10)	0.0016 (9)	-0.0065 (9)
C18	0.0264 (11)	0.0314 (11)	0.0254 (10)	0.0025 (10)	0.0035 (9)	-0.0046 (9)
C19	0.0405 (14)	0.0361 (13)	0.0437 (14)	-0.0055 (11)	0.0136 (12)	-0.0139 (11)
C20	0.0355 (15)	0.0556 (18)	0.072 (2)	-0.0083 (14)	0.0028 (15)	-0.0322 (17)
C21	0.0410 (16)	0.071 (2)	0.0646 (19)	0.0116 (16)	-0.0224 (15)	-0.0335 (18)
C22	0.0441 (15)	0.0508 (16)	0.0367 (13)	0.0163 (13)	-0.0132 (12)	-0.0123 (12)
C23	0.0276 (12)	0.0354 (13)	0.0426 (13)	0.0092 (10)	0.0121 (11)	0.0105 (10)
C24	0.0389 (14)	0.0353 (13)	0.0419 (13)	0.0113 (12)	0.0095 (12)	0.0029 (11)
C25	0.0520 (17)	0.0544 (17)	0.0480 (15)	0.0179 (15)	0.0211 (14)	0.0123 (13)

Geometric parameters (Å, º) for (Malone12)

O1—C1	1.216 (3)	C11—C16	1.389 (3)
N1—C10	1.366 (3)	C11—C12	1.415 (3)
N1—C11	1.371 (3)	C12—C13	1.405 (3)
N1—H1N	0.89 (3)	C13—C14	1.374 (3)
C1—C2	1.538 (3)	C13—H13	0.9500
C1—C6	1.549 (3)	C14—C15	1.411 (4)
C2—C17	1.535 (3)	C14—H14	0.9500
C2—C3	1.535 (3)	C15—C16	1.370 (4)

C2—C23	1.560 (3)	C15—H15	0.9500
C3—C4	1.523 (3)	C16—H16	0.9500
C3—H3A	0.9900	C17—C22	1.389 (3)
C3—H3B	0.9900	C17—C18	1.396 (3)
C4—C5	1.520 (4)	C18—C19	1.383 (4)
C4—H4A	0.9900	C18—H18	0.9500
C4—H4B	0.9900	C19—C20	1.376 (4)
C5—C6	1.545 (3)	C19—H19	0.9500
C5—H5A	0.9900	C20—C21	1.375 (5)
C5—H5B	0.9900	C20—H20	0.9500
C6—C9	1.510 (3)	C21—C22	1.393 (5)
C6—C7	1.546 (3)	C21—H21	0.9500
C7—C8	1.517 (4)	C22—H22	0.9500
C7—H7A	0.9900	C23—C24	1.480 (4)
C7—H7B	0.9900	C23—H23A	0.9900
C8—H8A	0.9800	C23—H23B	0.9900
C8—H8B	0.9800	C24—C25	1.325 (4)
C8—H8C	0.9800	C24—H24	0.9500
C9—C10	1.366 (3)	C25—H25A	0.9500
C9—C12	1.445 (3)	C25—H25B	0.9500
C10—H10	0.9500		
C10—N1—C11	109.12 (18)	C9—C10—N1	110.6 (2)
C10—N1—H1N	121.5 (18)	C9—C10—H10	124.7
C11—N1—H1N	129.3 (19)	N1—C10—H10	124.7
O1—C1—C2	120.1 (2)	N1—C11—C16	129.9 (2)
O1—C1—C6	119.6 (2)	N1—C11—C12	107.5 (2)
C2—C1—C6	120.34 (18)	C16—C11—C12	122.6 (2)
C17—C2—C3	113.9 (2)	C13—C12—C11	118.1 (2)
C17—C2—C1	109.19 (17)	C13—C12—C9	135.1 (2)
C3—C2—C1	108.52 (19)	C11—C12—C9	106.78 (19)
C17—C2—C23	108.91 (18)	C14—C13—C12	119.3 (2)
C3—C2—C23	108.32 (18)	C14—C13—H13	120.3

C1—C2—C23	107.81 (18)	C12—C13—H13	120.3
C4—C3—C2	113.37 (19)	C13—C14—C15	121.2 (2)
C4—C3—H3A	108.9	C13—C14—H14	119.4
C2—C3—H3A	108.9	C15—C14—H14	119.4
C4—C3—H3B	108.9	C16—C15—C14	120.9 (2)
C2—C3—H3B	108.9	C16—C15—H15	119.5
H3A—C3—H3B	107.7	C14—C15—H15	119.5
C5—C4—C3	109.7 (2)	C15—C16—C11	117.9 (2)
C5—C4—H4A	109.7	C15—C16—H16	121.1
C3—C4—H4A	109.7	C11—C16—H16	121.1
C5—C4—H4B	109.7	C22—C17—C18	117.4 (2)
C3—C4—H4B	109.7	C22—C17—C2	123.3 (2)
H4A—C4—H4B	108.2	C18—C17—C2	119.3 (2)
C4—C5—C6	114.8 (2)	C19—C18—C17	122.1 (2)
C4—C5—H5A	108.6	C19—C18—H18	119.0
C6—C5—H5A	108.6	C17—C18—H18	119.0
C4—C5—H5B	108.6	C20—C19—C18	119.7 (3)
C6—C5—H5B	108.6	C20—C19—H19	120.1
H5A—C5—H5B	107.5	C18—C19—H19	120.1
C9—C6—C5	112.17 (19)	C21—C20—C19	119.3 (3)
C9—C6—C7	110.28 (19)	C21—C20—H20	120.3
C5—C6—C7	109.4 (2)	C19—C20—H20	120.4
C9—C6—C1	106.94 (17)	C20—C21—C22	121.2 (3)
C5—C6—C1	109.42 (18)	C20—C21—H21	119.4
C7—C6—C1	108.49 (18)	C22—C21—H21	119.4
C8—C7—C6	113.8 (2)	C17—C22—C21	120.3 (3)
C8—C7—H7A	108.8	C17—C22—H22	119.9
C6—C7—H7A	108.8	C21—C22—H22	119.9
C8—C7—H7B	108.8	C24—C23—C2	112.9 (2)
C6—C7—H7B	108.8	C24—C23—H23A	109.0
H7A—C7—H7B	107.7	C2—C23—H23A	109.0
C7—C8—H8A	109.5	C24—C23—H23B	109.0
C7—C8—H8B	109.5	C2—C23—H23B	109.0

H8A—C8—H8B	109.5	H23A—C23—H23B	107.8
C7—C8—H8C	109.5	C25—C24—C23	124.2 (3)
H8A—C8—H8C	109.5	C25—C24—H24	117.9
H8B—C8—H8C	109.5	C23—C24—H24	117.9
C10—C9—C12	105.9 (2)	C24—C25—H25A	120.0
C10—C9—C6	127.4 (2)	C24—C25—H25B	120.0
C12—C9—C6	126.69 (19)	H25A—C25—H25B	120.0
O1—C1—C2—C17	-99.0 (2)	N1—C11—C12—C13	-179.81 (18)
C6—C1—C2—C17	80.1 (2)	C16—C11—C12—C13	2.2 (3)
O1—C1—C2—C3	136.3 (2)	N1—C11—C12—C9	1.8 (2)
C6—C1—C2—C3	-44.6 (3)	C16—C11—C12—C9	-176.1 (2)
O1—C1—C2—C23	19.2 (3)	C10—C9—C12—C13	-179.3 (2)
C6—C1—C2—C23	-161.70 (19)	C6—C9—C12—C13	0.3 (4)
C17—C2—C3—C4	-69.1 (3)	C10—C9—C12—C11	-1.3 (2)
C1—C2—C3—C4	52.7 (3)	C6—C9—C12—C11	178.3 (2)
C23—C2—C3—C4	169.5 (2)	C11—C12—C13—C14	-1.7 (3)
C2—C3—C4—C5	-60.6 (3)	C9—C12—C13—C14	176.0 (2)
C3—C4—C5—C6	57.0 (3)	C12—C13—C14—C15	-0.3 (3)
C4—C5—C6—C9	72.4 (3)	C13—C14—C15—C16	1.9 (4)
C4—C5—C6—C7	-164.8 (2)	C14—C15—C16—C11	-1.4 (4)
C4—C5—C6—C1	-46.1 (3)	N1—C11—C16—C15	-178.1 (2)

O1—C1—C6—C9	98.7 (2)	C12—C11—C16—C15	-0.6 (3)
C2—C1—C6—C9	-80.5 (2)	C3—C2—C17—C22	-14.8 (3)
O1—C1—C6—C5	-139.6 (2)	C1—C2—C17—C22	-136.3 (2)
C2—C1—C6—C5	41.3 (3)	C23—C2—C17—C22	106.2 (2)
O1—C1—C6—C7	-20.3 (3)	C3—C2—C17—C18	167.5 (2)
C2—C1—C6—C7	160.6 (2)	C1—C2—C17—C18	46.1 (3)
C9—C6—C7—C8	61.4 (3)	C23—C2—C17—C18	-71.4 (2)
C5—C6—C7—C8	-62.4 (3)	C22—C17—C18—C19	2.3 (3)
C1—C6—C7—C8	178.2 (2)	C2—C17—C18—C19	-179.9 (2)
C5—C6—C9—C10	1.9 (3)	C17—C18—C19—C20	-1.4 (4)
C7—C6—C9—C10	-120.4 (2)	C18—C19—C20—C21	-0.4 (4)
C1—C6—C9—C10	121.8 (2)	C19—C20—C21—C22	1.2 (4)
C5—C6—C9—C12	-177.7 (2)	C18—C17—C22—C21	-1.4 (4)
C7—C6—C9—C12	60.1 (3)	C2—C17—C22—C21	-179.1 (2)
C1—C6—C9—C12	-57.7 (3)	C20—C21—C22—C17	-0.3 (4)
C12—C9—C10—N1	0.3 (2)	C17—C2—C23—C24	-71.0 (3)
C6—C9—C10—N1	-179.3 (2)	C3—C2—C23—C24	53.4 (3)
C11—N1—C10—C9	0.9 (3)	C1—C2—C23—C24	170.6 (2)
C10—N1—C11—C16	176.1 (2)	C2—C23—C24—C25	-121.4 (3)

C10—N1—C11—C12	-1.7 (2)		

(±)-(2*R*,6*R*)-2-(allyl-2-*d*)-6-(1*H*-indol-3-yl)-6-methyl-2-phenylcyclohexan-1-one (22b)

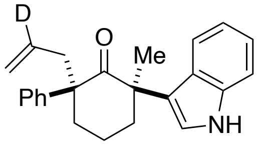

22b

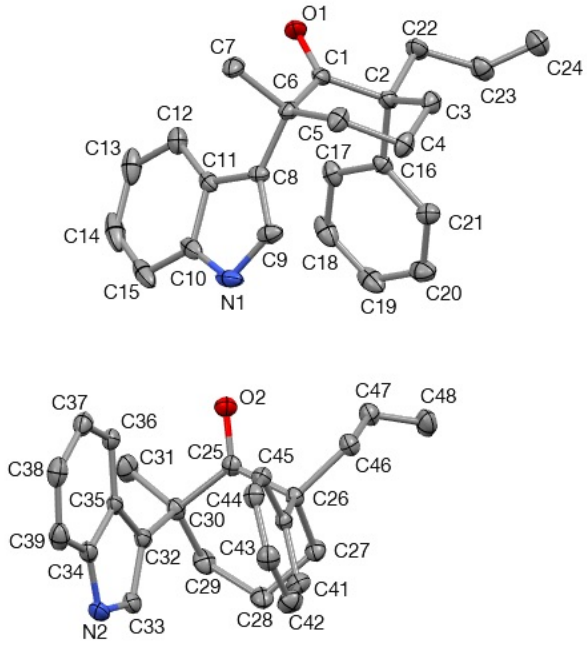

Sample Name: CCDC 2072247 / Satish6 (SCP1234P1)

Crystal data

$C_{24}H_{24}DNO$	$Z = 4$
$M_r = 344.45$	$F(000) = 736$
Triclinic, $P\bar{1}$	$D_x = 1.210$ Mg m^{-3}
$a = 11.6922$ (8) Å	Mo $K\alpha$ radiation, $\lambda = 0.71073$ Å
$b = 11.8408$ (8) Å	Cell parameters from 9926 reflections
$c = 14.1595$ (9) Å	$\theta = 2.5–30.2°$
$\alpha = 77.005$ (2)°	$\mu = 0.07$ mm^{-1}
$\beta = 81.879$ (2)°	$T = 90$ K
$\gamma = 87.592$ (2)°	Plate fragment
$V = 1890.8$ (2) Å^3	0.27 × 0.21 × 0.15 mm

Data collection

Bruker Kappa APEX-II diffractometer	17482 independent reflections

Radiation source: fine-focus sealed tube	10344 reflections with $I > 2\sigma(I)$
TRIUMPH curved graphite monochromator	R_{int} = 0.071
ϕ and ω scans	θ_{max} = 35.7°, θ_{min} = 1.8°
Absorption correction: multi-scan *SADABS* (Sheldrick, 2004)	h = -19 – 19
T_{min} = 0.933, T_{max} = 0.989	k = -19 – 19
115822 measured reflections	l = -23 – 23

Refinement

Refinement on F^2	0 restraints
Least-squares matrix: full	Hydrogen site location: mixed
$R[F^2 > 2\sigma(F^2)]$ = 0.059	H atoms treated by a mixture of independent and constrained refinement
$wR(F^2)$ = 0.157	$w = 1/[\sigma^2(F_o^2) + (0.0656P)^2 + 0.5202P]$ where $P = (F_o^2 + 2F_c^2)/3$
S = 1.01	$(\Delta/\sigma)_{max}$ < 0.001
17482 reflections	$\Delta\rho_{max}$ = 0.51 e Å^{-3}
477 parameters	$\Delta\rho_{min}$ = -0.25 e Å^{-3}

Fractional atomic coordinates and isotropic or equivalent isotropic displacement parameters (Å^2) for (SCP1234P1)

	x	y	z	U_{iso}*/U_{eq}
O1	-0.01374 (8)	0.60045 (8)	0.24266 (8)	0.0303 (2)
N1	0.33880 (10)	0.28638 (9)	0.21666 (9)	0.0286 (2)
H1N	0.4034 (15)	0.2463 (15)	0.2181 (12)	0.034*
C1	0.07986 (10)	0.58075 (10)	0.27215 (9)	0.0195 (2)
C2	0.16848 (10)	0.67869 (10)	0.25575 (8)	0.0180 (2)
C3	0.20162 (11)	0.67913 (11)	0.35694 (9)	0.0216 (2)
H3A	0.260989	0.738883	0.349397	0.026*
H3B	0.132777	0.700801	0.398719	0.026*
C4	0.24790 (11)	0.56309 (11)	0.40784 (9)	0.0230 (2)
H4A	0.269164	0.569079	0.471770	0.028*

H4B	0.318272	0.541796	0.367592	0.028*
C5	0.15708 (11)	0.46919 (11)	0.42360 (8)	0.0233 (2)
H5A	0.092419	0.485400	0.472076	0.028*
H5B	0.191817	0.393679	0.452000	0.028*
C6	0.10772 (9)	0.45840 (10)	0.33046 (8)	0.0181 (2)
C7	-0.00432 (10)	0.38765 (12)	0.36236 (9)	0.0247 (2)
H7A	0.011462	0.313865	0.406415	0.037*
H7B	-0.032792	0.372581	0.304552	0.037*
H7C	-0.062856	0.431546	0.396402	0.037*
C8	0.19038 (9)	0.39502 (9)	0.26695 (8)	0.01647 (19)
C9	0.29455 (10)	0.34562 (10)	0.28708 (9)	0.0229 (2)
H9	0.330994	0.351371	0.341654	0.027*
C10	0.26357 (11)	0.29671 (10)	0.14924 (10)	0.0243 (2)
C11	0.16829 (10)	0.36507 (9)	0.17795 (8)	0.0177 (2)
C12	0.08040 (11)	0.39145 (11)	0.11829 (9)	0.0235 (2)
H12	0.015156	0.436649	0.135899	0.028*
C13	0.09045 (15)	0.35034 (13)	0.03303 (10)	0.0344 (3)
H13	0.032253	0.369156	-0.008655	0.041*
C14	0.18491 (16)	0.28167 (13)	0.00758 (11)	0.0425 (4)
H14	0.189140	0.254020	-0.050824	0.051*
C15	0.27169 (14)	0.25324 (12)	0.06498 (11)	0.0362 (4)
H15	0.335066	0.205571	0.047801	0.043*
C16	0.27126 (10)	0.66000 (10)	0.18100 (8)	0.0179 (2)
C17	0.25106 (12)	0.62679 (10)	0.09632 (9)	0.0223 (2)
H17	0.174058	0.614814	0.086800	0.027*
C18	0.34195 (14)	0.61099 (11)	0.02574 (9)	0.0302 (3)
H18	0.326993	0.585491	-0.030292	0.036*
C19	0.45400 (13)	0.63228 (11)	0.03682 (10)	0.0329 (3)
H19	0.516168	0.621442	-0.011417	0.040*
C20	0.47525 (12)	0.66958 (12)	0.11884 (10)	0.0291 (3)
H20	0.551929	0.686560	0.125736	0.035*
C21	0.38472 (10)	0.68223 (11)	0.19101 (9)	0.0224 (2)
H21	0.400276	0.706202	0.247565	0.027*

C22	0.10981 (11)	0.79692 (11)	0.21548 (10)	0.0253 (2)
H22A	0.079959	0.793525	0.154144	0.030*
H22B	0.043175	0.809907	0.263489	0.030*
C23	0.19076 (12)	0.89718 (11)	0.19565 (10)	0.0266 (3)
D23	0.247151	0.906235	0.139259	0.032*
C24	0.18943 (13)	0.97377 (12)	0.25078 (12)	0.0326 (3)
H24A	0.134302	0.967562	0.307772	0.039*
H24B	0.243615	1.035037	0.233426	0.039*
O2	0.54175 (7)	0.14549 (7)	0.24514 (7)	0.02345 (18)
N2	0.77506 (9)	-0.26126 (9)	0.27225 (9)	0.0244 (2)
H2N	0.8257 (14)	-0.3182 (15)	0.2769 (12)	0.029*
C25	0.64162 (10)	0.11478 (9)	0.22617 (8)	0.0173 (2)
C26	0.73779 (9)	0.15770 (9)	0.27296 (8)	0.01647 (19)
C27	0.84256 (10)	0.19439 (10)	0.19407 (8)	0.0202 (2)
H27A	0.823264	0.268185	0.150133	0.024*
H27B	0.908297	0.209222	0.226517	0.024*
C28	0.88084 (11)	0.10611 (11)	0.13225 (9)	0.0235 (2)
H28A	0.945475	0.137870	0.081348	0.028*
H28B	0.908583	0.034552	0.174143	0.028*
C29	0.78063 (11)	0.07752 (11)	0.08398 (9)	0.0244 (2)
H29A	0.806992	0.018418	0.045899	0.029*
H29B	0.758956	0.148147	0.037396	0.029*
C30	0.67247 (10)	0.03209 (10)	0.15611 (8)	0.0188 (2)
C31	0.57177 (12)	0.02813 (12)	0.09791 (10)	0.0275 (3)
H31A	0.503193	-0.002817	0.143067	0.041*
H31B	0.593016	-0.021899	0.051533	0.041*
H31C	0.554901	0.106576	0.062042	0.041*
C32	0.69255 (10)	-0.08820 (10)	0.21712 (8)	0.0173 (2)
C33	0.78838 (10)	-0.15704 (10)	0.20639 (9)	0.0213 (2)
H33	0.854905	-0.135481	0.159711	0.026*
C34	0.66934 (10)	-0.26199 (10)	0.32866 (9)	0.0207 (2)
C35	0.61388 (10)	-0.15462 (9)	0.29563 (8)	0.0167 (2)
C36	0.50412 (10)	-0.13386 (10)	0.34382 (9)	0.0194 (2)

H36	0.463683	-0.063551	0.322396	0.023*
C37	0.45561 (11)	-0.21696 (11)	0.42282 (9)	0.0238 (2)
H37	0.382229	-0.202329	0.456493	0.029*
C38	0.51292 (12)	-0.32267 (11)	0.45418 (10)	0.0275 (3)
H38	0.477790	-0.378329	0.508642	0.033*
C39	0.61951 (12)	-0.34657 (11)	0.40686 (10)	0.0269 (3)
H39	0.657736	-0.418557	0.427030	0.032*
C40	0.76492 (10)	0.06118 (9)	0.35992 (8)	0.01656 (19)
C41	0.87662 (10)	0.01982 (11)	0.37186 (9)	0.0222 (2)
H41	0.939289	0.049225	0.323289	0.027*
C42	0.89746 (11)	-0.06386 (11)	0.45389 (10)	0.0261 (3)
H42	0.974023	-0.091551	0.460425	0.031*
C43	0.80765 (11)	-0.10729 (11)	0.52625 (9)	0.0244 (2)
H43	0.822410	-0.164055	0.582404	0.029*
C44	0.69636 (11)	-0.06713 (10)	0.51584 (9)	0.0217 (2)
H44	0.634230	-0.096358	0.565034	0.026*
C45	0.67520 (10)	0.01596 (10)	0.43350 (8)	0.0186 (2)
H45	0.598300	0.042581	0.427044	0.022*
C46	0.69351 (10)	0.26381 (10)	0.31676 (9)	0.0200 (2)
H46A	0.624821	0.239511	0.365672	0.024*
H46B	0.754116	0.284455	0.351988	0.024*
C47	0.66182 (11)	0.37073 (10)	0.24541 (9)	0.0224 (2)
D47	0.595897	0.367604	0.213847	0.027*
C48	0.71940 (13)	0.46897 (11)	0.22350 (11)	0.0310 (3)
H48A	0.785748	0.475137	0.253700	0.037*
H48B	0.694438	0.533418	0.177544	0.037*

Atomic displacement parameters (Å^2) for (SCP1234P1)

	U^{11}	U^{22}	U^{33}	U^{12}	U^{13}	U^{23}
O1	0.0192 (4)	0.0290 (5)	0.0505 (6)	0.0091 (4)	-0.0161 (4)	-0.0202 (4)
N1	0.0187 (5)	0.0191 (5)	0.0418 (7)	0.0058 (4)	0.0064 (4)	-0.0015 (4)
C1	0.0163 (5)	0.0239 (5)	0.0220 (5)	0.0047 (4)	-0.0035	-0.0134

					(4)	(4)
C2	0.0165 (5)	0.0205 (5)	0.0195 (5)	0.0037 (4)	-0.0055 (4)	-0.0083 (4)
C3	0.0225 (6)	0.0262 (6)	0.0191 (5)	-0.0013 (4)	-0.0044 (4)	-0.0102 (4)
C4	0.0239 (6)	0.0286 (6)	0.0175 (5)	-0.0051 (5)	-0.0069 (4)	-0.0040 (4)
C5	0.0232 (6)	0.0311 (6)	0.0160 (5)	-0.0057 (5)	-0.0019 (4)	-0.0057 (4)
C6	0.0146 (5)	0.0244 (5)	0.0168 (5)	-0.0008 (4)	-0.0010 (4)	-0.0079 (4)
C7	0.0173 (5)	0.0336 (6)	0.0254 (6)	-0.0046 (5)	0.0030 (4)	-0.0140 (5)
C8	0.0138 (5)	0.0174 (5)	0.0167 (5)	0.0011 (4)	0.0004 (4)	-0.0023 (4)
C9	0.0177 (5)	0.0213 (5)	0.0253 (6)	0.0014 (4)	-0.0007 (4)	0.0023 (4)
C10	0.0258 (6)	0.0136 (5)	0.0289 (6)	-0.0027 (4)	0.0112 (5)	-0.0040 (4)
C11	0.0209 (5)	0.0140 (4)	0.0166 (5)	-0.0015 (4)	0.0028 (4)	-0.0031 (4)
C12	0.0295 (6)	0.0205 (5)	0.0211 (5)	-0.0058 (5)	-0.0045 (5)	-0.0044 (4)
C13	0.0521 (9)	0.0321 (7)	0.0207 (6)	-0.0188 (6)	-0.0046 (6)	-0.0064 (5)
C14	0.0681 (11)	0.0341 (8)	0.0259 (7)	-0.0255 (7)	0.0165 (7)	-0.0174 (6)
C15	0.0461 (8)	0.0214 (6)	0.0376 (8)	-0.0105 (6)	0.0225 (7)	-0.0148 (6)
C16	0.0207 (5)	0.0161 (5)	0.0167 (5)	0.0033 (4)	-0.0036 (4)	-0.0034 (4)
C17	0.0320 (6)	0.0167 (5)	0.0181 (5)	-0.0012 (4)	-0.0043 (4)	-0.0028 (4)
C18	0.0501 (8)	0.0197 (6)	0.0186 (6)	-0.0055 (5)	0.0056 (5)	-0.0050 (4)
C19	0.0413 (8)	0.0204 (6)	0.0306 (7)	-0.0026 (5)	0.0157 (6)	-0.0046 (5)
C20	0.0242 (6)	0.0244 (6)	0.0343 (7)	-0.0001 (5)	0.0051 (5)	-0.0029 (5)

C21	0.0205 (5)	0.0231 (5)	0.0228 (5)	0.0016 (4)	-0.0023 (4)	-0.0042 (4)
C22	0.0248 (6)	0.0222 (6)	0.0331 (7)	0.0085 (5)	-0.0120 (5)	-0.0111 (5)
C23	0.0313 (7)	0.0217 (6)	0.0277 (6)	0.0061 (5)	-0.0088 (5)	-0.0053 (5)
C24	0.0341 (7)	0.0235 (6)	0.0415 (8)	0.0014 (5)	-0.0039 (6)	-0.0109 (6)
O2	0.0191 (4)	0.0211 (4)	0.0313 (5)	0.0033 (3)	-0.0070 (3)	-0.0067 (3)
N2	0.0213 (5)	0.0190 (5)	0.0366 (6)	0.0066 (4)	-0.0089 (4)	-0.0119 (4)
C25	0.0194 (5)	0.0148 (4)	0.0172 (5)	0.0003 (4)	-0.0048 (4)	-0.0012 (4)
C26	0.0175 (5)	0.0166 (5)	0.0158 (5)	-0.0002 (4)	-0.0027 (4)	-0.0045 (4)
C27	0.0214 (5)	0.0201 (5)	0.0186 (5)	-0.0029 (4)	-0.0002 (4)	-0.0044 (4)
C28	0.0228 (6)	0.0251 (6)	0.0218 (5)	-0.0030 (4)	0.0046 (4)	-0.0077 (4)
C29	0.0316 (6)	0.0255 (6)	0.0163 (5)	-0.0018 (5)	-0.0007 (4)	-0.0062 (4)
C30	0.0225 (5)	0.0189 (5)	0.0169 (5)	0.0008 (4)	-0.0068 (4)	-0.0055 (4)
C31	0.0335 (7)	0.0277 (6)	0.0261 (6)	0.0022 (5)	-0.0169 (5)	-0.0085 (5)
C32	0.0184 (5)	0.0183 (5)	0.0179 (5)	0.0013 (4)	-0.0058 (4)	-0.0080 (4)
C33	0.0211 (5)	0.0217 (5)	0.0246 (6)	0.0016 (4)	-0.0044 (4)	-0.0122 (4)
C34	0.0219 (5)	0.0170 (5)	0.0265 (6)	0.0019 (4)	-0.0094 (4)	-0.0086 (4)
C35	0.0181 (5)	0.0160 (5)	0.0192 (5)	0.0011 (4)	-0.0069 (4)	-0.0080 (4)
C36	0.0185 (5)	0.0196 (5)	0.0226 (5)	-0.0008 (4)	-0.0053 (4)	-0.0086 (4)
C37	0.0232 (6)	0.0269 (6)	0.0238 (6)	-0.0059 (5)	-0.0031 (4)	-0.0100 (5)
C38	0.0342 (7)	0.0234 (6)	0.0255 (6)	-0.0091	-0.0079	-0.0026

				(5)	(5)	(5)
C39	0.0328 (7)	0.0167 (5)	0.0332 (7)	-0.0002 (5)	-0.0147 (5)	-0.0034 (5)
C40	0.0181 (5)	0.0168 (5)	0.0164 (5)	-0.0011 (4)	-0.0039 (4)	-0.0058 (4)
C41	0.0180 (5)	0.0254 (6)	0.0227 (5)	0.0001 (4)	-0.0034 (4)	-0.0039 (4)
C42	0.0220 (6)	0.0276 (6)	0.0284 (6)	0.0027 (5)	-0.0093 (5)	-0.0026 (5)
C43	0.0298 (6)	0.0211 (5)	0.0226 (6)	0.0000 (5)	-0.0087 (5)	-0.0020 (4)
C44	0.0251 (6)	0.0203 (5)	0.0191 (5)	-0.0034 (4)	-0.0023 (4)	-0.0029 (4)
C45	0.0179 (5)	0.0185 (5)	0.0201 (5)	-0.0006 (4)	-0.0034 (4)	-0.0051 (4)
C46	0.0232 (5)	0.0180 (5)	0.0198 (5)	0.0002 (4)	-0.0035 (4)	-0.0059 (4)
C47	0.0261 (6)	0.0193 (5)	0.0235 (6)	0.0011 (4)	-0.0055 (4)	-0.0069 (4)
C48	0.0338 (7)	0.0174 (5)	0.0438 (8)	0.0002 (5)	-0.0132 (6)	-0.0065 (5)

Geometric parameters (Å, º) for (SCP1234P1)

O1—C1	1.2208 (14)	O2—C25	1.2196 (14)
N1—C10	1.3695 (19)	N2—C33	1.3692 (17)
N1—C9	1.3756 (17)	N2—C34	1.3729 (17)
N1—H1N	0.875 (18)	N2—H2N	0.876 (17)
C1—C2	1.5441 (17)	C25—C26	1.5400 (16)
C1—C6	1.5443 (17)	C25—C30	1.5423 (16)
C2—C16	1.5287 (16)	C26—C40	1.5395 (15)
C2—C3	1.5375 (16)	C26—C27	1.5417 (16)
C2—C22	1.5576 (16)	C26—C46	1.5609 (15)
C3—C4	1.5174 (18)	C27—C28	1.5233 (16)
C3—H3A	0.9900	C27—H27A	0.9900
C3—H3B	0.9900	C27—H27B	0.9900
C4—C5	1.5278 (17)	C28—C29	1.5226 (18)

C4—H4A	0.9900	C28—H28A	0.9900
C4—H4B	0.9900	C28—H28B	0.9900
C5—C6	1.5446 (16)	C29—C30	1.5417 (17)
C5—H5A	0.9900	C29—H29A	0.9900
C5—H5B	0.9900	C29—H29B	0.9900
C6—C8	1.5176 (15)	C30—C32	1.5185 (16)
C6—C7	1.5417 (16)	C30—C31	1.5375 (16)
C7—H7A	0.9800	C31—H31A	0.9800
C7—H7B	0.9800	C31—H31B	0.9800
C7—H7C	0.9800	C31—H31C	0.9800
C8—C9	1.3675 (16)	C32—C33	1.3689 (16)
C8—C11	1.4425 (16)	C32—C35	1.4419 (16)
C9—H9	0.9500	C33—H33	0.9500
C10—C15	1.3918 (19)	C34—C39	1.3916 (18)
C10—C11	1.4163 (16)	C34—C35	1.4158 (16)
C11—C12	1.4024 (17)	C35—C36	1.4052 (16)
C12—C13	1.3884 (18)	C36—C37	1.3834 (17)
C12—H12	0.9500	C36—H36	0.9500
C13—C14	1.398 (2)	C37—C38	1.4059 (19)
C13—H13	0.9500	C37—H37	0.9500
C14—C15	1.373 (3)	C38—C39	1.379 (2)
C14—H14	0.9500	C38—H38	0.9500
C15—H15	0.9500	C39—H39	0.9500
C16—C17	1.3957 (16)	C40—C41	1.3948 (16)
C16—C21	1.3959 (17)	C40—C45	1.4001 (16)
C17—C18	1.3894 (18)	C41—C42	1.3900 (17)
C17—H17	0.9500	C41—H41	0.9500
C18—C19	1.381 (2)	C42—C43	1.3861 (19)
C18—H18	0.9500	C42—H42	0.9500
C19—C20	1.388 (2)	C43—C44	1.3827 (18)
C19—H19	0.9500	C43—H43	0.9500
C20—C21	1.3918 (17)	C44—C45	1.3905 (16)
C20—H20	0.9500	C44—H44	0.9500

C21—H21	0.9500	C45—H45	0.9500
C22—C23	1.5012 (19)	C46—C47	1.5006 (17)
C22—H22A	0.9900	C46—H46A	0.9900
C22—H22B	0.9900	C46—H46B	0.9900
C23—C24	1.3213 (19)	C47—C48	1.3218 (18)
C23—D23	0.9500	C47—D47	0.9500
C24—H24A	0.9500	C48—H48A	0.9500
C24—H24B	0.9500	C48—H48B	0.9500
C10—N1—C9	109.12 (10)	C33—N2—C34	108.88 (10)
C10—N1—H1N	127.0 (11)	C33—N2—H2N	125.2 (11)
C9—N1—H1N	123.8 (11)	C34—N2—H2N	125.9 (11)
O1—C1—C2	120.47 (11)	O2—C25—C26	120.02 (10)
O1—C1—C6	119.80 (11)	O2—C25—C30	120.45 (10)
C2—C1—C6	119.67 (9)	C26—C25—C30	119.53 (9)
C16—C2—C3	113.64 (9)	C40—C26—C25	108.19 (9)
C16—C2—C1	110.44 (9)	C40—C26—C27	114.02 (9)
C3—C2—C1	106.25 (9)	C25—C26—C27	109.16 (9)
C16—C2—C22	108.43 (10)	C40—C26—C46	106.15 (9)
C3—C2—C22	108.82 (9)	C25—C26—C46	110.04 (9)
C1—C2—C22	109.17 (9)	C27—C26—C46	109.22 (9)
C4—C3—C2	113.23 (10)	C28—C27—C26	114.69 (9)
C4—C3—H3A	108.9	C28—C27—H27A	108.6
C2—C3—H3A	108.9	C26—C27—H27A	108.6
C4—C3—H3B	108.9	C28—C27—H27B	108.6
C2—C3—H3B	108.9	C26—C27—H27B	108.6
H3A—C3—H3B	107.7	H27A—C27—H27B	107.6
C3—C4—C5	110.44 (10)	C29—C28—C27	110.22 (10)
C3—C4—H4A	109.6	C29—C28—H28A	109.6
C5—C4—H4A	109.6	C27—C28—H28A	109.6
C3—C4—H4B	109.6	C29—C28—H28B	109.6
C5—C4—H4B	109.6	C27—C28—H28B	109.6
H4A—C4—H4B	108.1	H28A—C28—	108.1

		H28B	
C4—C5—C6	114.98 (10)	C28—C29—C30	114.36 (10)
C4—C5—H5A	108.5	C28—C29—H29A	108.7
C6—C5—H5A	108.5	C30—C29—H29A	108.7
C4—C5—H5B	108.5	C28—C29—H29B	108.7
C6—C5—H5B	108.5	C30—C29—H29B	108.7
H5A—C5—H5B	107.5	H29A—C29—H29B	107.6
C8—C6—C7	107.80 (9)	C32—C30—C31	109.19 (10)
C8—C6—C1	110.36 (9)	C32—C30—C29	111.81 (10)
C7—C6—C1	109.46 (10)	C31—C30—C29	108.91 (10)
C8—C6—C5	112.02 (10)	C32—C30—C25	108.15 (9)
C7—C6—C5	108.04 (9)	C31—C30—C25	109.75 (10)
C1—C6—C5	109.10 (9)	C29—C30—C25	109.01 (9)
C6—C7—H7A	109.5	C30—C31—H31A	109.5
C6—C7—H7B	109.5	C30—C31—H31B	109.5
H7A—C7—H7B	109.5	H31A—C31—H31B	109.5
C6—C7—H7C	109.5	C30—C31—H31C	109.5
H7A—C7—H7C	109.5	H31A—C31—H31C	109.5
H7B—C7—H7C	109.5	H31B—C31—H31C	109.5
C9—C8—C11	106.57 (10)	C33—C32—C35	106.03 (10)
C9—C8—C6	127.11 (11)	C33—C32—C30	126.61 (11)
C11—C8—C6	126.05 (10)	C35—C32—C30	127.33 (10)
C8—C9—N1	109.99 (12)	C32—C33—N2	110.59 (11)
C8—C9—H9	125.0	C32—C33—H33	124.7
N1—C9—H9	125.0	N2—C33—H33	124.7
N1—C10—C15	129.99 (13)	N2—C34—C39	129.85 (11)
N1—C10—C11	107.78 (11)	N2—C34—C35	107.64 (11)
C15—C10—C11	122.22 (14)	C39—C34—C35	122.49 (11)
C12—C11—C10	118.68 (11)	C36—C35—C34	118.25 (11)
C12—C11—C8	134.73 (10)	C36—C35—C32	134.86 (10)
C10—C11—C8	106.55 (11)	C34—C35—C32	106.85 (10)

C13—C12—C11	118.82 (13)	C37—C36—C35	119.25 (11)
C13—C12—H12	120.6	C37—C36—H36	120.4
C11—C12—H12	120.6	C35—C36—H36	120.4
C12—C13—C14	121.00 (15)	C36—C37—C38	121.20 (12)
C12—C13—H13	119.5	C36—C37—H37	119.4
C14—C13—H13	119.5	C38—C37—H37	119.4
C15—C14—C13	121.58 (13)	C39—C38—C37	120.82 (12)
C15—C14—H14	119.2	C39—C38—H38	119.6
C13—C14—H14	119.2	C37—C38—H38	119.6
C14—C15—C10	117.66 (13)	C38—C39—C34	117.95 (12)
C14—C15—H15	121.2	C38—C39—H39	121.0
C10—C15—H15	121.2	C34—C39—H39	121.0
C17—C16—C21	118.32 (11)	C41—C40—C45	117.69 (10)
C17—C16—C2	119.16 (10)	C41—C40—C26	122.97 (10)
C21—C16—C2	122.38 (10)	C45—C40—C26	119.26 (10)
C18—C17—C16	120.95 (12)	C42—C41—C40	120.88 (11)
C18—C17—H17	119.5	C42—C41—H41	119.6
C16—C17—H17	119.5	C40—C41—H41	119.6
C19—C18—C17	120.17 (12)	C43—C42—C41	120.66 (12)
C19—C18—H18	119.9	C43—C42—H42	119.7
C17—C18—H18	119.9	C41—C42—H42	119.7
C18—C19—C20	119.62 (12)	C44—C43—C42	119.31 (11)
C18—C19—H19	120.2	C44—C43—H43	120.3
C20—C19—H19	120.2	C42—C43—H43	120.3
C19—C20—C21	120.30 (13)	C43—C44—C45	120.13 (11)
C19—C20—H20	119.8	C43—C44—H44	119.9
C21—C20—H20	119.8	C45—C44—H44	119.9
C20—C21—C16	120.55 (12)	C44—C45—C40	121.33 (11)
C20—C21—H21	119.7	C44—C45—H45	119.3
C16—C21—H21	119.7	C40—C45—H45	119.3
C23—C22—C2	112.76 (10)	C47—C46—C26	116.40 (9)
C23—C22—H22A	109.0	C47—C46—H46A	108.2
C2—C22—H22A	109.0	C26—C46—H46A	108.2

C23—C22—H22B	109.0	C47—C46—H46B	108.2
C2—C22—H22B	109.0	C26—C46—H46B	108.2
H22A—C22—H22B	107.8	H46A—C46—H46B	107.3
C24—C23—C22	124.88 (13)	C48—C47—C46	124.45 (12)
C24—C23—D23	117.6	C48—C47—D47	117.8
C22—C23—D23	117.6	C46—C47—D47	117.8
C23—C24—H24A	120.0	C47—C48—H48A	120.0
C23—C24—H24B	120.0	C47—C48—H48B	120.0
H24A—C24—H24B	120.0	H48A—C48—H48B	120.0
O1—C1—C2—C16	-109.96 (12)	O2—C25—C26—C40	99.70 (12)
C6—C1—C2—C16	72.89 (12)	C30—C25—C26—C40	-79.76 (12)
O1—C1—C2—C3	126.37 (12)	O2—C25—C26—C27	-135.70 (11)
C6—C1—C2—C3	-50.78 (12)	C30—C25—C26—C27	44.83 (13)
O1—C1—C2—C22	9.17 (15)	O2—C25—C26—C46	-15.86 (14)
C6—C1—C2—C22	-167.98 (10)	C30—C25—C26—C46	164.67 (9)
C16—C2—C3—C4	-65.28 (13)	C40—C26—C27—C28	72.16 (13)
C1—C2—C3—C4	56.37 (12)	C25—C26—C27—C28	-48.95 (13)
C22—C2—C3—C4	173.81 (10)	C46—C26—C27—C28	-169.29 (10)
C2—C3—C4—C5	-60.36 (13)	C26—C27—C28—C29	56.25 (14)
C3—C4—C5—C6	53.82 (14)	C27—C28—C29—C30	-56.76 (14)
O1—C1—C6—C8	104.91 (12)	C28—C29—C30—C32	-69.55 (13)
C2—C1—C6—C8	-77.93 (12)	C28—C29—C30—C31	169.70 (10)

O1—C1—C6—C7	-13.59 (14)	C28—C29—C30—C25	49.98 (13)
C2—C1—C6—C7	163.58 (9)	O2—C25—C30—C32	-103.07 (12)
O1—C1—C6—C5	-131.61 (11)	C26—C25—C30—C32	76.39 (12)
C2—C1—C6—C5	45.56 (13)	O2—C25—C30—C31	15.97 (15)
C4—C5—C6—C8	77.59 (13)	C26—C25—C30—C31	-164.58 (10)
C4—C5—C6—C7	-163.82 (11)	O2—C25—C30—C29	135.16 (11)
C4—C5—C6—C1	-44.90 (14)	C26—C25—C30—C29	-45.38 (13)
C7—C6—C8—C9	-115.41 (13)	C31—C30—C32—C33	114.34 (13)
C1—C6—C8—C9	125.09 (12)	C29—C30—C32—C33	-6.25 (15)
C5—C6—C8—C9	3.32 (16)	C25—C30—C32—C33	-126.27 (12)
C7—C6—C8—C11	57.77 (14)	C31—C30—C32—C35	-63.51 (14)
C1—C6—C8—C11	-61.73 (14)	C29—C30—C32—C35	175.91 (10)
C5—C6—C8—C11	176.50 (10)	C25—C30—C32—C35	55.88 (14)
C11—C8—C9—N1	-0.23 (13)	C35—C32—C33—N2	0.08 (13)
C6—C8—C9—N1	174.02 (11)	C30—C32—C33—N2	-178.14 (10)
C10—N1—C9—C8	0.11 (14)	C34—N2—C33—C32	-0.62 (13)
C9—N1—C10—C15	179.13 (12)	C33—N2—C34—C39	-177.44 (12)
C9—N1—C10—C11	0.05 (13)	C33—N2—C34—C35	0.91 (13)
N1—C10—C11—C12	177.93 (10)	N2—C34—C35—C36	-178.85 (10)
C15—C10—	-1.23 (17)	C39—C34—	-0.36 (16)

C11—C12		C35—C36	
N1—C10—C11—C8	-0.19 (12)	N2—C34—C35—C32	-0.84 (12)
C15—C10—C11—C8	-179.35 (11)	C39—C34—C35—C32	177.65 (11)
C9—C8—C11—C12	-177.42 (13)	C33—C32—C35—C36	178.00 (12)
C6—C8—C11—C12	8.3 (2)	C30—C32—C35—C36	-3.8 (2)
C9—C8—C11—C10	0.25 (12)	C33—C32—C35—C34	0.47 (12)
C6—C8—C11—C10	-174.07 (10)	C30—C32—C35—C34	178.67 (10)
C10—C11—C12—C13	-0.46 (17)	C34—C35—C36—C37	1.66 (16)
C8—C11—C12—C13	177.00 (12)	C32—C35—C36—C37	-175.65 (12)
C11—C12—C13—C14	1.40 (19)	C35—C36—C37—C38	-1.51 (17)
C12—C13—C14—C15	-0.7 (2)	C36—C37—C38—C39	-0.01 (19)
C13—C14—C15—C10	-1.0 (2)	C37—C38—C39—C34	1.31 (18)
N1—C10—C15—C14	-177.03 (13)	N2—C34—C39—C38	177.01 (12)
C11—C10—C15—C14	1.93 (18)	C35—C34—C39—C38	-1.12 (18)
C3—C2—C16—C17	162.86 (10)	C25—C26—C40—C41	128.55 (11)
C1—C2—C16—C17	43.58 (14)	C27—C26—C40—C41	6.91 (15)
C22—C2—C16—C17	-76.00 (13)	C46—C26—C40—C41	-113.37 (12)
C3—C2—C16—C21	-21.44 (15)	C25—C26—C40—C45	-54.74 (13)
C1—C2—C16—C21	-140.72 (11)	C27—C26—C40—C45	-176.38 (10)
C22—C2—C16—C21	99.70 (12)	C46—C26—C40—C45	63.34 (12)

C21—C16—C17—C18	2.93 (17)	C45—C40—C41—C42	0.34 (17)
C2—C16—C17—C18	178.80 (11)	C26—C40—C41—C42	177.10 (11)
C16—C17—C18—C19	-2.42 (19)	C40—C41—C42—C43	-0.6 (2)
C17—C18—C19—C20	0.0 (2)	C41—C42—C43—C44	0.46 (19)
C18—C19—C20—C21	1.9 (2)	C42—C43—C44—C45	0.01 (18)
C19—C20—C21—C16	-1.36 (19)	C43—C44—C45—C40	-0.31 (18)
C17—C16—C21—C20	-1.04 (17)	C41—C40—C45—C44	0.13 (17)
C2—C16—C21—C20	-176.78 (11)	C26—C40—C45—C44	-176.75 (10)
C16—C2—C22—C23	-58.61 (13)	C40—C26—C46—C47	179.55 (10)
C3—C2—C22—C23	65.46 (13)	C25—C26—C46—C47	-63.60 (13)
C1—C2—C22—C23	-178.98 (10)	C27—C26—C46—C47	56.20 (13)
C2—C22—C23—C24	-105.18 (15)	C26—C46—C47—C48	-111.88 (14)

Hydrogen-bond geometry (Å, º) for (SCP1234P1)

D—H···*A*	*D*—H	H···*A*	*D*···*A*	*D*—H···*A*
N1—H1*N*···O2	0.875 (18)	1.999 (18)	2.8657 (14)	170.4 (16)
N2—H2*N*···O1^{i}	0.876 (17)	2.119 (17)	2.9379 (14)	155.5 (15)

Symmetry code: (i) *x*+1, *y*-1, *z*.

(±)-(2R,6R)-2-allyl-6-(1H-indol-3-yl)-2,6-dimethylcyclohexanone (24b)

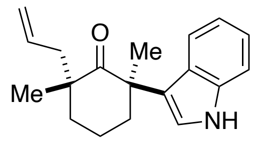

24b

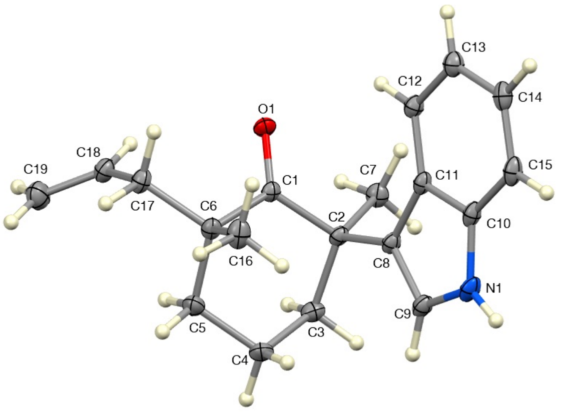

Sample Name: CCDC 2072248 / Malone14 (JAM7277)

Crystal data

$C_{19}H_{23}NO$	D_x = 1.226 Mg m^{-3}
M_r = 281.38	Mo $K\alpha$ radiation, λ = 0.71073 Å
Orthorhombic, $Pca2_1$	Cell parameters from 9889 reflections
a = 16.0164 (12) Å	θ = 2.4–33.7°
b = 8.5137 (6) Å	μ = 0.08 mm^{-1}
c = 11.1772 (8) Å	T = 90 K
V = 1524.11 (19) Å^3	Plate fragment, tan
Z = 4	0.28 × 0.15 × 0.11 mm
F(000) = 608	

Data collection

Bruker Kappa APEX-II DUO diffractometer	5832 independent reflections
Radiation source: fine-focus sealed tube	5435 reflections with $I > 2\sigma(I)$
TRIUMPH curved graphite monochromator	R_{int} = 0.044
ϕ and ω scans	θ_{max} = 33.8°, θ_{min} = 2.4°
Absorption correction: multi-scan *SADABS* (Sheldrick, 2004)	h = -25 – 23

T_{min} = 0.939, T_{max} = 0.992	k = -13 – 13
33513 measured reflections	l = -16 – 17

Refinement

Refinement on F^2	Hydrogen site location: mixed
Least-squares matrix: full	H atoms treated by a mixture of independent and constrained refinement
$R[F^2 > 2\sigma(F^2)]$ = 0.035	$w = 1/[\sigma^2(F_o^2) + (0.0578P)^2 + 0.0881P]$ where $P = (F_o^2 + 2F_c^2)/3$
$wR(F^2)$ = 0.092	$(\Delta/\sigma)_{max}$ < 0.001
S = 1.06	$\Delta\rho_{max}$ = 0.40 e Å^{-3}
5832 reflections	$\Delta\rho_{min}$ = -0.19 e Å^{-3}
196 parameters	Absolute structure: Refined as an inversion twin.
1 restraint	Absolute structure parameter: 0.0 (10)

Fractional atomic coordinates and isotropic or equivalent isotropic displacement parameters (Å^2) for (Malone14)

	x	y	z	U_{iso}*/U_{eq}
O1	0.51859 (5)	0.62747 (10)	0.50773 (9)	0.01644 (17)
N1	0.19558 (6)	0.44331 (13)	0.47811 (11)	0.0185 (2)
H1N	0.1424 (12)	0.433 (2)	0.4711 (18)	0.022*
C1	0.45470 (6)	0.68319 (13)	0.46577 (11)	0.01237 (18)
C2	0.40284 (6)	0.58877 (13)	0.37396 (11)	0.01327 (19)
C3	0.37801 (7)	0.69961 (15)	0.27040 (12)	0.0172 (2)
H3A	0.428423	0.723690	0.222551	0.021*
H3B	0.338129	0.644068	0.217575	0.021*
C4	0.33847 (8)	0.85388 (14)	0.31115 (13)	0.0192 (2)
H4A	0.325469	0.919505	0.240443	0.023*
H4B	0.285547	0.831747	0.353680	0.023*
C5	0.39761 (7)	0.94297 (14)	0.39401 (13)	0.0188 (2)
H5A	0.369428	1.039744	0.421968	0.023*
H5B	0.447554	0.974994	0.347886	0.023*

C6	0.42596 (6)	0.84739 (13)	0.50388 (12)	0.01465 (19)
C7	0.45678 (7)	0.45478 (14)	0.32329 (12)	0.0172 (2)
H7A	0.428282	0.407116	0.254723	0.026*
H7B	0.465555	0.375104	0.385310	0.026*
H7C	0.510844	0.496800	0.297583	0.026*
C8	0.32726 (6)	0.51856 (13)	0.43606 (11)	0.01401 (19)
C9	0.24498 (7)	0.52948 (14)	0.40210 (12)	0.0168 (2)
H9	0.225151	0.588157	0.335622	0.020*
C10	0.24495 (7)	0.37188 (13)	0.56277 (12)	0.0161 (2)
C11	0.32877 (7)	0.41744 (13)	0.53971 (11)	0.0139 (2)
C12	0.39193 (7)	0.36035 (13)	0.61531 (11)	0.0154 (2)
H12	0.448584	0.388450	0.602033	0.018*
C13	0.37049 (8)	0.26246 (14)	0.70954 (12)	0.0181 (2)
H13	0.412936	0.224957	0.761595	0.022*
C14	0.28708 (8)	0.21751 (15)	0.72963 (12)	0.0194 (2)
H14	0.274215	0.149586	0.794493	0.023*
C15	0.22383 (7)	0.27066 (14)	0.65653 (12)	0.0190 (2)
H15	0.167590	0.239491	0.669441	0.023*
C16	0.35368 (8)	0.82863 (16)	0.59461 (13)	0.0211 (2)
H16A	0.372162	0.762926	0.661553	0.032*
H16B	0.305902	0.778848	0.555074	0.032*
H16C	0.337190	0.932217	0.624664	0.032*
C17	0.49745 (7)	0.93477 (14)	0.56934 (12)	0.0169 (2)
H17A	0.475307	1.035615	0.599972	0.020*
H17B	0.514519	0.871205	0.639361	0.020*
C18	0.57367 (7)	0.96861 (15)	0.49549 (12)	0.0179 (2)
H18	0.605483	0.881906	0.467480	0.021*
C19	0.59944 (9)	1.11191 (17)	0.46684 (15)	0.0257 (3)
H19A	0.569022	1.201115	0.493441	0.031*
H19B	0.648291	1.125279	0.419733	0.031*

Atomic displacement parameters (Å^2) for (Malone14)

	U^{11}	U^{22}	U^{33}	U^{12}	U^{13}	U^{23}
O1	0.0104 (3)	0.0175 (4)	0.0214 (4)	0.0014 (3)	-0.0015 (3)	0.0000 (3)
N1	0.0088 (4)	0.0218 (5)	0.0248 (6)	-0.0020 (3)	0.0010 (4)	-0.0051 (4)
C1	0.0095 (4)	0.0139 (4)	0.0136 (4)	-0.0006 (3)	0.0021 (3)	-0.0001 (4)
C2	0.0100 (4)	0.0144 (4)	0.0154 (5)	0.0002 (3)	0.0002 (3)	-0.0023 (4)
C3	0.0148 (4)	0.0206 (5)	0.0162 (5)	0.0008 (4)	-0.0017 (4)	0.0008 (4)
C4	0.0141 (5)	0.0191 (5)	0.0244 (6)	0.0028 (4)	-0.0048 (4)	0.0017 (5)
C5	0.0158 (5)	0.0151 (5)	0.0256 (6)	0.0018 (4)	-0.0026 (4)	0.0011 (4)
C6	0.0112 (4)	0.0143 (4)	0.0185 (5)	0.0005 (3)	0.0020 (4)	-0.0025 (4)
C7	0.0142 (4)	0.0173 (5)	0.0199 (6)	0.0011 (3)	0.0036 (4)	-0.0034 (4)
C8	0.0095 (4)	0.0152 (4)	0.0172 (5)	-0.0004 (3)	0.0003 (3)	-0.0028 (4)
C9	0.0110 (4)	0.0184 (5)	0.0211 (5)	-0.0004 (4)	-0.0015 (4)	-0.0035 (4)
C10	0.0111 (4)	0.0168 (4)	0.0204 (5)	-0.0021 (3)	0.0029 (4)	-0.0065 (4)
C11	0.0104 (4)	0.0142 (4)	0.0171 (5)	-0.0013 (3)	0.0024 (3)	-0.0040 (4)
C12	0.0127 (4)	0.0165 (5)	0.0169 (5)	-0.0010 (3)	0.0014 (4)	-0.0023 (4)
C13	0.0175 (5)	0.0176 (5)	0.0193 (6)	-0.0019 (4)	0.0027 (4)	-0.0020 (4)
C14	0.0216 (5)	0.0165 (5)	0.0200 (6)	-0.0048 (4)	0.0065 (4)	-0.0036 (4)
C15	0.0151 (5)	0.0185 (5)	0.0233 (6)	-0.0048 (4)	0.0064 (4)	-0.0063 (4)
C16	0.0164 (5)	0.0212 (5)	0.0257 (6)	-0.0012 (4)	0.0079 (4)	-0.0067 (5)
C17	0.0150 (4)	0.0174 (4)	0.0184 (6)	-0.0020	0.0015 (4)	-0.0048

				(4)		(4)
C18	0.0140 (4)	0.0195 (5)	0.0202 (5)	-0.0019 (4)	-0.0002 (4)	-0.0028 (4)
C19	0.0211 (6)	0.0229 (6)	0.0332 (7)	-0.0043 (4)	0.0019 (5)	0.0030 (5)

Geometric parameters (Å, º) for (Malone14)

O1—C1	1.2216 (13)	C8—C9	1.3746 (15)
N1—C9	1.3734 (17)	C8—C11	1.4435 (17)
N1—C10	1.3749 (17)	C9—H9	0.9500
N1—H1N	0.86 (2)	C10—C15	1.3984 (18)
C1—C6	1.5322 (16)	C10—C11	1.4210 (15)
C1—C2	1.5456 (16)	C11—C12	1.4049 (17)
C2—C8	1.5181 (15)	C12—C13	1.3863 (17)
C2—C7	1.5389 (16)	C12—H12	0.9500
C2—C3	1.5455 (17)	C13—C14	1.4078 (17)
C3—C4	1.5275 (18)	C13—H13	0.9500
C3—H3A	0.9900	C14—C15	1.3779 (19)
C3—H3B	0.9900	C14—H14	0.9500
C4—C5	1.5265 (18)	C15—H15	0.9500
C4—H4A	0.9900	C16—H16A	0.9800
C4—H4B	0.9900	C16—H16B	0.9800
C5—C6	1.5415 (18)	C16—H16C	0.9800
C5—H5A	0.9900	C17—C18	1.5016 (17)
C5—H5B	0.9900	C17—H17A	0.9900
C6—C16	1.5473 (17)	C17—H17B	0.9900
C6—C17	1.5492 (17)	C18—C19	1.3271 (18)
C7—H7A	0.9800	C18—H18	0.9500
C7—H7B	0.9800	C19—H19A	0.9500
C7—H7C	0.9800	C19—H19B	0.9500
C9—N1—C10	109.30 (10)	C9—C8—C11	106.15 (10)
C9—N1—H1N	124.7 (13)	C9—C8—C2	127.70 (11)
C10—N1—H1N	125.9 (13)	C11—C8—C2	126.04 (10)

O1—C1—C6	119.95 (10)	N1—C9—C8	110.20 (11)
O1—C1—C2	120.20 (10)	N1—C9—H9	124.9
C6—C1—C2	119.85 (9)	C8—C9—H9	124.9
C8—C2—C7	108.91 (9)	N1—C10—C15	130.48 (11)
C8—C2—C3	112.19 (9)	N1—C10—C11	107.33 (11)
C7—C2—C3	108.75 (10)	C15—C10—C11	122.19 (12)
C8—C2—C1	109.25 (9)	C12—C11—C10	118.48 (11)
C7—C2—C1	109.16 (9)	C12—C11—C8	134.52 (10)
C3—C2—C1	108.54 (9)	C10—C11—C8	107.01 (10)
C4—C3—C2	114.10 (11)	C13—C12—C11	119.12 (11)
C4—C3—H3A	108.7	C13—C12—H12	120.4
C2—C3—H3A	108.7	C11—C12—H12	120.4
C4—C3—H3B	108.7	C12—C13—C14	121.31 (12)
C2—C3—H3B	108.7	C12—C13—H13	119.3
H3A—C3—H3B	107.6	C14—C13—H13	119.3
C5—C4—C3	110.55 (10)	C15—C14—C13	120.93 (12)
C5—C4—H4A	109.5	C15—C14—H14	119.5
C3—C4—H4A	109.5	C13—C14—H14	119.5
C5—C4—H4B	109.5	C14—C15—C10	117.96 (11)
C3—C4—H4B	109.5	C14—C15—H15	121.0
H4A—C4—H4B	108.1	C10—C15—H15	121.0
C4—C5—C6	113.82 (10)	C6—C16—H16A	109.5
C4—C5—H5A	108.8	C6—C16—H16B	109.5
C6—C5—H5A	108.8	H16A—C16—H16B	109.5
C4—C5—H5B	108.8	C6—C16—H16C	109.5
C6—C5—H5B	108.8	H16A—C16—H16C	109.5
H5A—C5—H5B	107.7	H16B—C16—H16C	109.5
C1—C6—C5	110.41 (10)	C18—C17—C6	115.68 (11)
C1—C6—C16	108.22 (9)	C18—C17—H17A	108.4
C5—C6—C16	110.87 (10)	C6—C17—H17A	108.4
C1—C6—C17	110.33 (9)	C18—C17—H17B	108.4

C5—C6—C17	109.91 (9)	C6—C17—H17B	108.4
C16—C6—C17	107.04 (10)	H17A—C17—H17B	107.4
C2—C7—H7A	109.5	C19—C18—C17	124.18 (12)
C2—C7—H7B	109.5	C19—C18—H18	117.9
H7A—C7—H7B	109.5	C17—C18—H18	117.9
C2—C7—H7C	109.5	C18—C19—H19A	120.0
H7A—C7—H7C	109.5	C18—C19—H19B	120.0
H7B—C7—H7C	109.5	H19A—C19—H19B	120.0
O1—C1—C2—C8	102.30 (12)	C1—C2—C8—C11	-56.11 (14)
C6—C1—C2—C8	-77.91 (12)	C10—N1—C9—C8	-0.93 (14)
O1—C1—C2—C7	-16.70 (15)	C11—C8—C9—N1	0.52 (13)
C6—C1—C2—C7	163.10 (10)	C2—C8—C9—N1	176.91 (11)
O1—C1—C2—C3	-135.09 (11)	C9—N1—C10—C15	-178.43 (12)
C6—C1—C2—C3	44.70 (13)	C9—N1—C10—C11	0.93 (13)
C8—C2—C3—C4	70.47 (13)	N1—C10—C11—C12	179.51 (10)
C7—C2—C3—C4	-169.00 (10)	C15—C10—C11—C12	-1.07 (17)
C1—C2—C3—C4	-50.34 (12)	N1—C10—C11—C8	-0.60 (13)
C2—C3—C4—C5	57.91 (14)	C15—C10—C11—C8	178.83 (11)
C3—C4—C5—C6	-55.91 (14)	C9—C8—C11—C12	179.92 (13)
O1—C1—C6—C5	135.93 (11)	C2—C8—C11—C12	3.5 (2)
C2—C1—C6—C5	-43.86 (13)	C9—C8—C11—C10	0.05 (13)
O1—C1—C6—C16	-102.56 (12)	C2—C8—C11—C10	-176.41 (10)

C2—C1—C6—C16	77.65 (13)	C10—C11—C12—C13	-0.20 (17)
O1—C1—C6—C17	14.25 (15)	C8—C11—C12—C13	179.94 (12)
C2—C1—C6—C17	-165.54 (10)	C11—C12—C13—C14	0.98 (18)
C4—C5—C6—C1	47.75 (13)	C12—C13—C14—C15	-0.53 (19)
C4—C5—C6—C16	-72.17 (13)	C13—C14—C15—C10	-0.71 (18)
C4—C5—C6—C17	169.69 (10)	N1—C10—C15—C14	-179.21 (12)
C7—C2—C8—C9	-112.67 (13)	C11—C10—C15—C14	1.51 (17)
C3—C2—C8—C9	7.78 (16)	C1—C6—C17—C18	63.84 (14)
C1—C2—C8—C9	128.18 (12)	C5—C6—C17—C18	-58.14 (13)
C7—C2—C8—C11	63.04 (14)	C16—C6—C17—C18	-178.62 (11)
C3—C2—C8—C11	-176.52 (11)	C6—C17—C18—C19	114.89 (15)

Hydrogen-bond geometry (Å, º) for (Malone14)

D—H···*A*	*D*—H	H···*A*	*D*···*A*	*D*—H···*A*
N1—H1*N*···O1^{i}	0.86 (2)	2.09 (2)	2.9168 (13)	161.4 (19)

Symmetry code: (i) *x*-1/2, -*y*+1, *z*.

^{1}H AND ^{13}C NMR SPECTRA

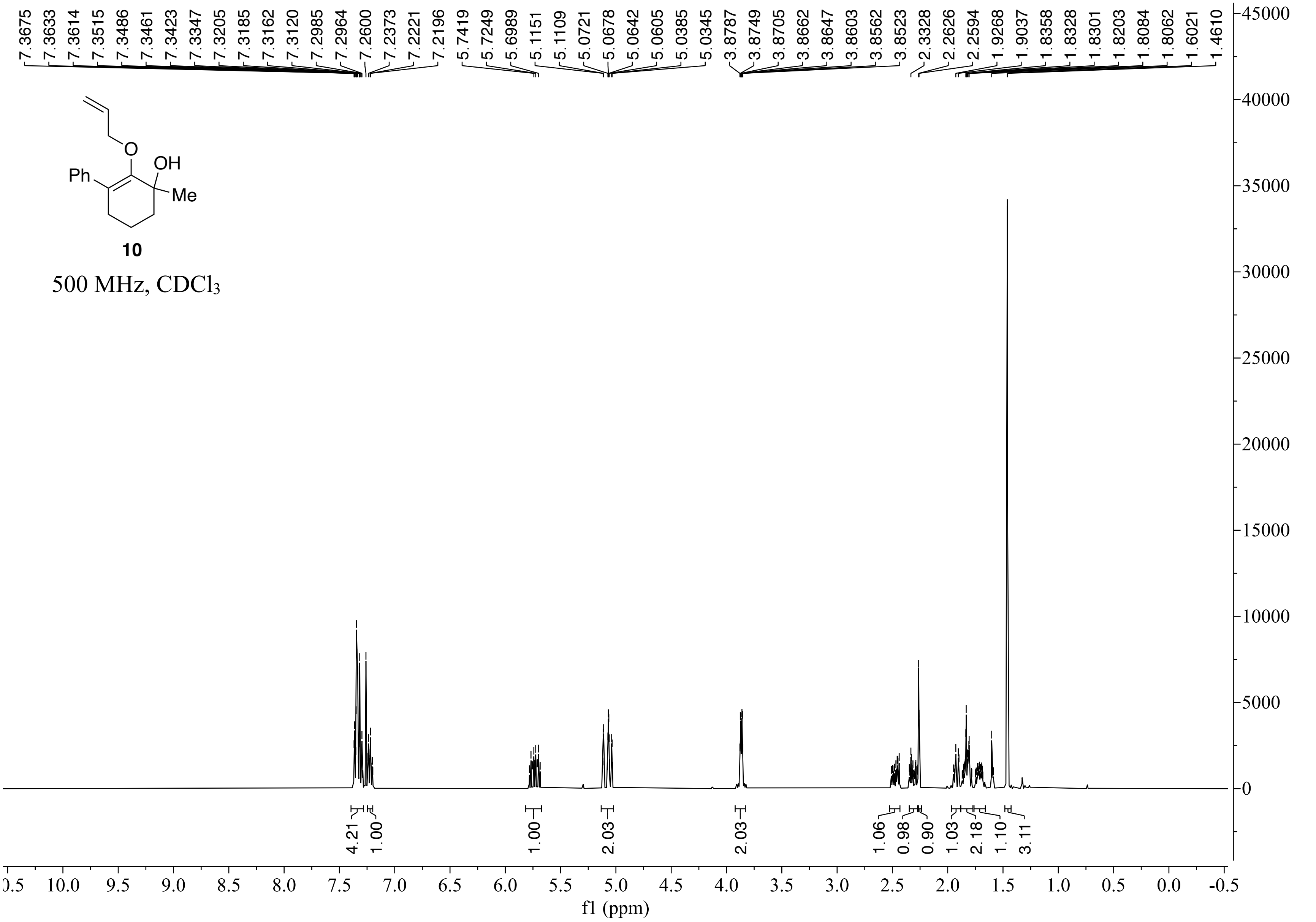
7.3675 7.3633 7.3614 7.3515 7.3486 7.3461 7.3423 7.3347 7.3205 7.3185 7.3162 7.3120 7.2985 7.2964 7.2600 7.2373 7.2221 7.2196 5.7419 5.7249 5.6989 5.1151 5.1109 5.0721 5.0678 5.0642 5.0605 5.0385 5.0345 3.8787 3.8749 3.8705 3.8662 3.8647 3.8603 3.8562 3.8523 2.3328 2.2626 2.2594 1.9268 1.9037 1.8358 1.8328 1.8301 1.8203 1.8084 1.8062 1.6021 1.4610
O
OH
Ph
Me
10
500 MHz, CDCl$_3$
4.21 1.00 1.00 2.03 2.03 1.06 0.98 0.90 1.03 2.18 1.10 3.11
f1 (ppm)

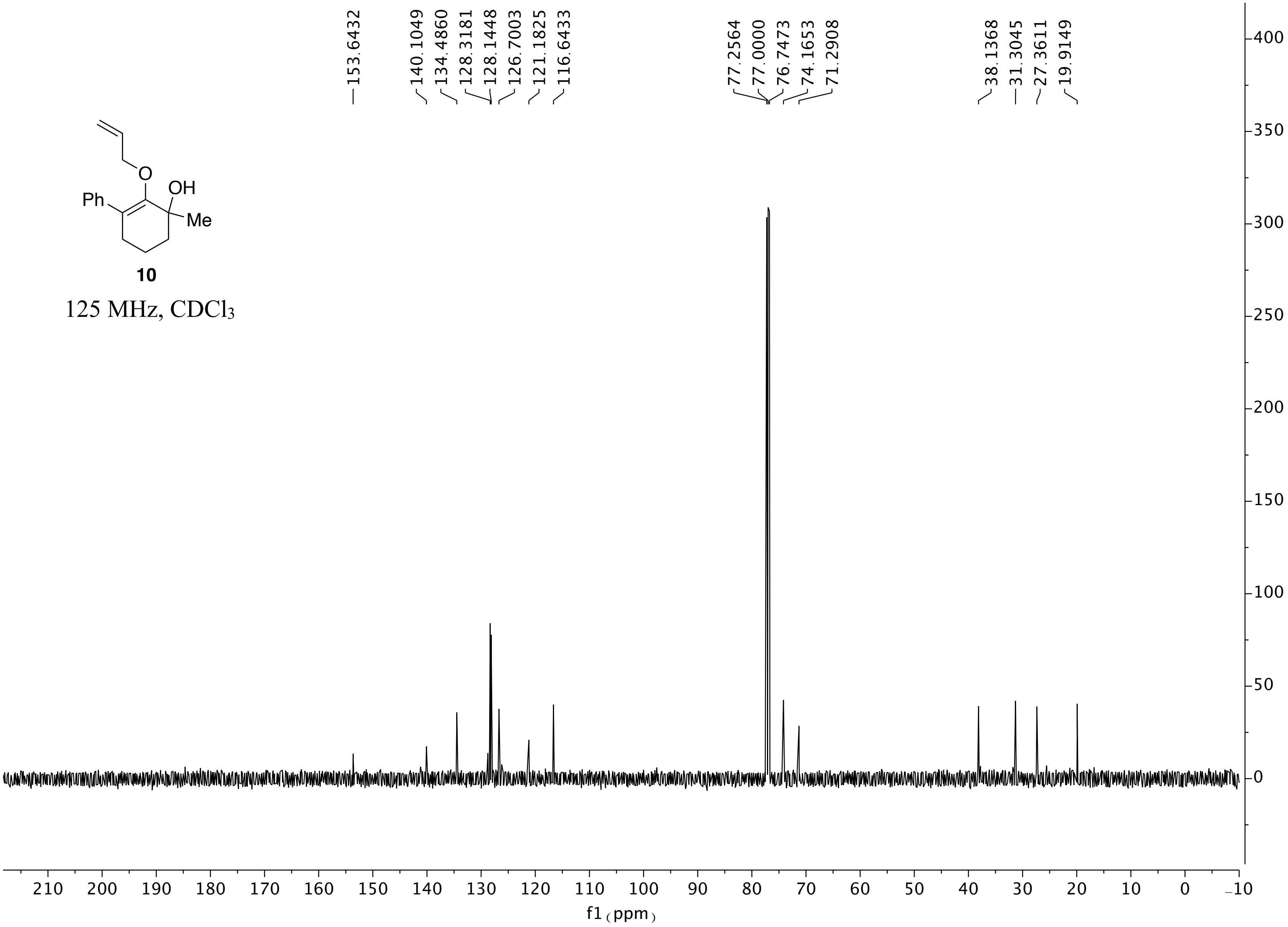
Ph
O
OH
Me
10
125 MHz, CDCl3
153.6432
140.1049
134.4860
128.3181
128.1448
126.7003
121.1825
116.6433
77.2564
77.0000
76.7473
74.1653
71.2908
38.1368
31.3045
27.3611
19.9149
400
350
300
250
200
150
100
50
0
210 200 190 180 170 160 150 140 130 120 110 100 90 80 70 60 50 40 30 20 10 0 -10
f1 (ppm)

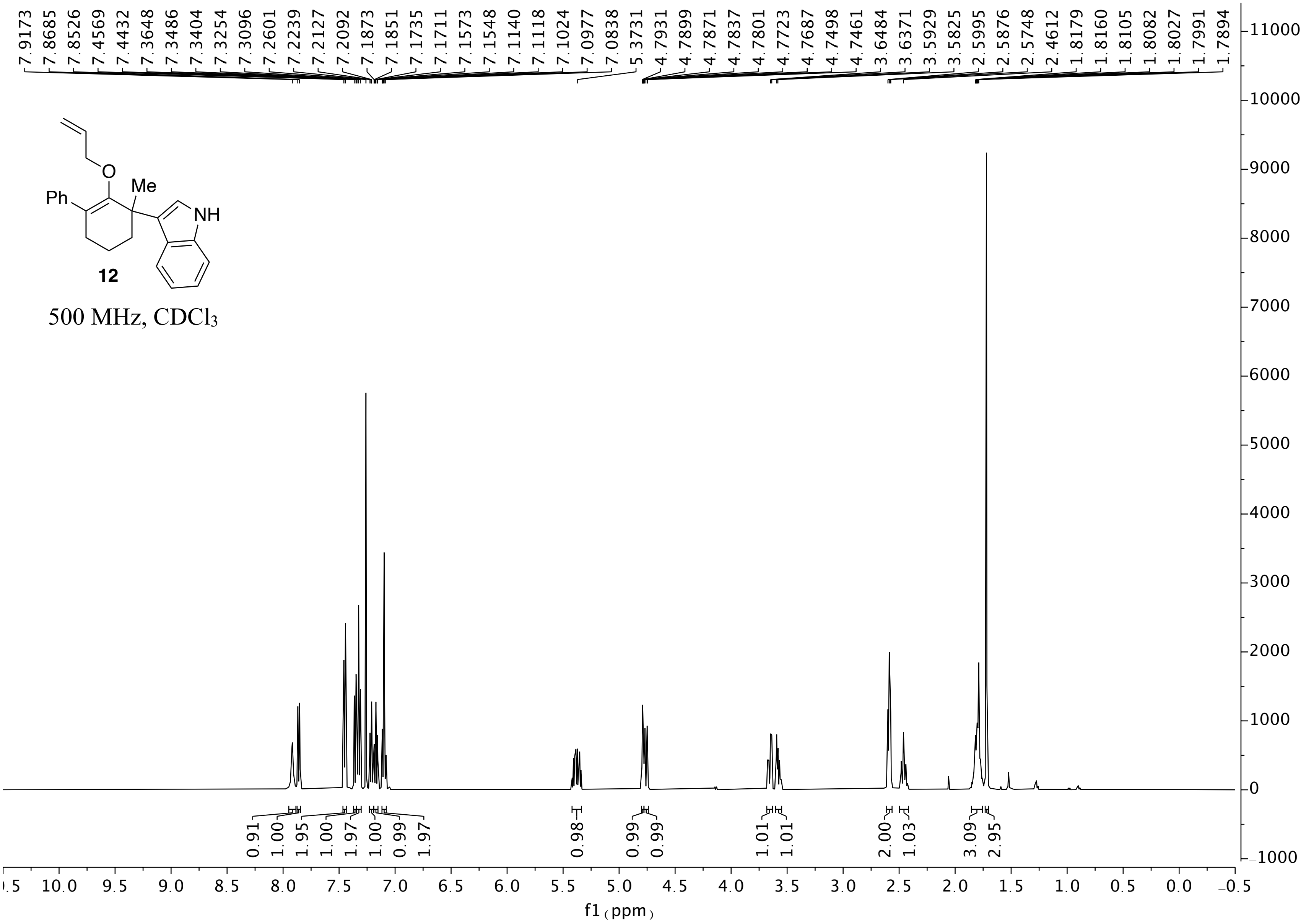

Me
O
Ph
NH
12
500 MHz, $CDCl_3$
f1 (ppm)

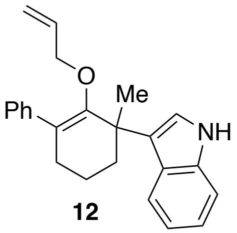

12

125 MHz, $CDCl_3$

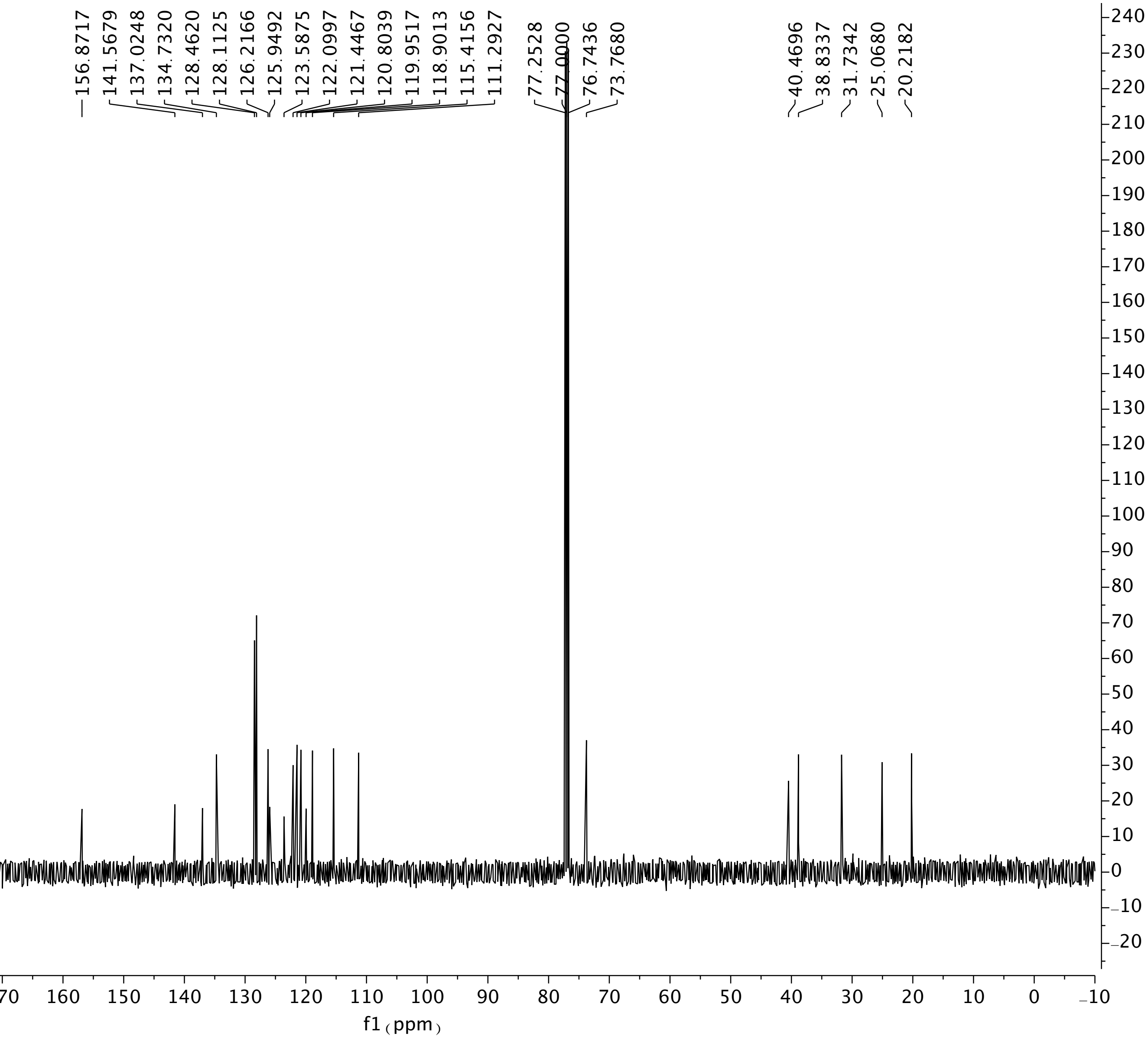

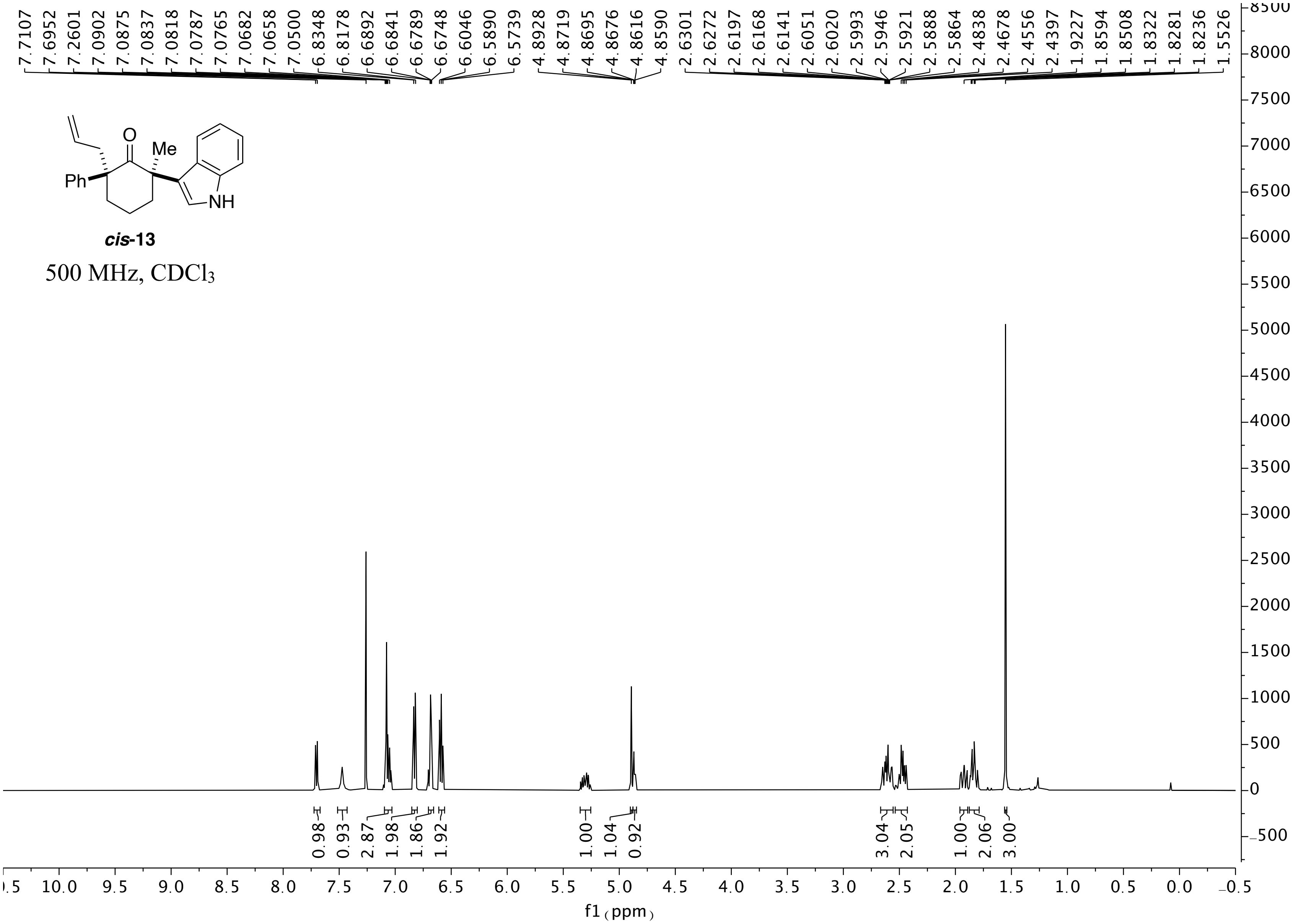

Me
O
Ph
NH
cis-13
500 MHz, $CDCl_3$
7.7107
7.6952
7.2601
7.0902
7.0875
7.0837
7.0818
7.0787
7.0765
7.0682
7.0658
7.0500
6.8348
6.8178
6.6892
6.6841
6.6789
6.6748
6.6046
6.5890
6.5739
4.8928
4.8719
4.8695
4.8676
4.8616
4.8590
2.6301
2.6272
2.6197
2.6168
2.6141
2.6051
2.6020
2.5993
2.5946
2.5921
2.5888
2.5864
2.4838
2.4678
2.4556
2.4397
1.9227
1.8594
1.8508
1.8322
1.8281
1.8236
1.5526
0.98
0.93
2.87
1.98
1.86
1.92
1.00
1.04
0.92
3.04
2.05
1.00
2.06
3.00
f1 (ppm)

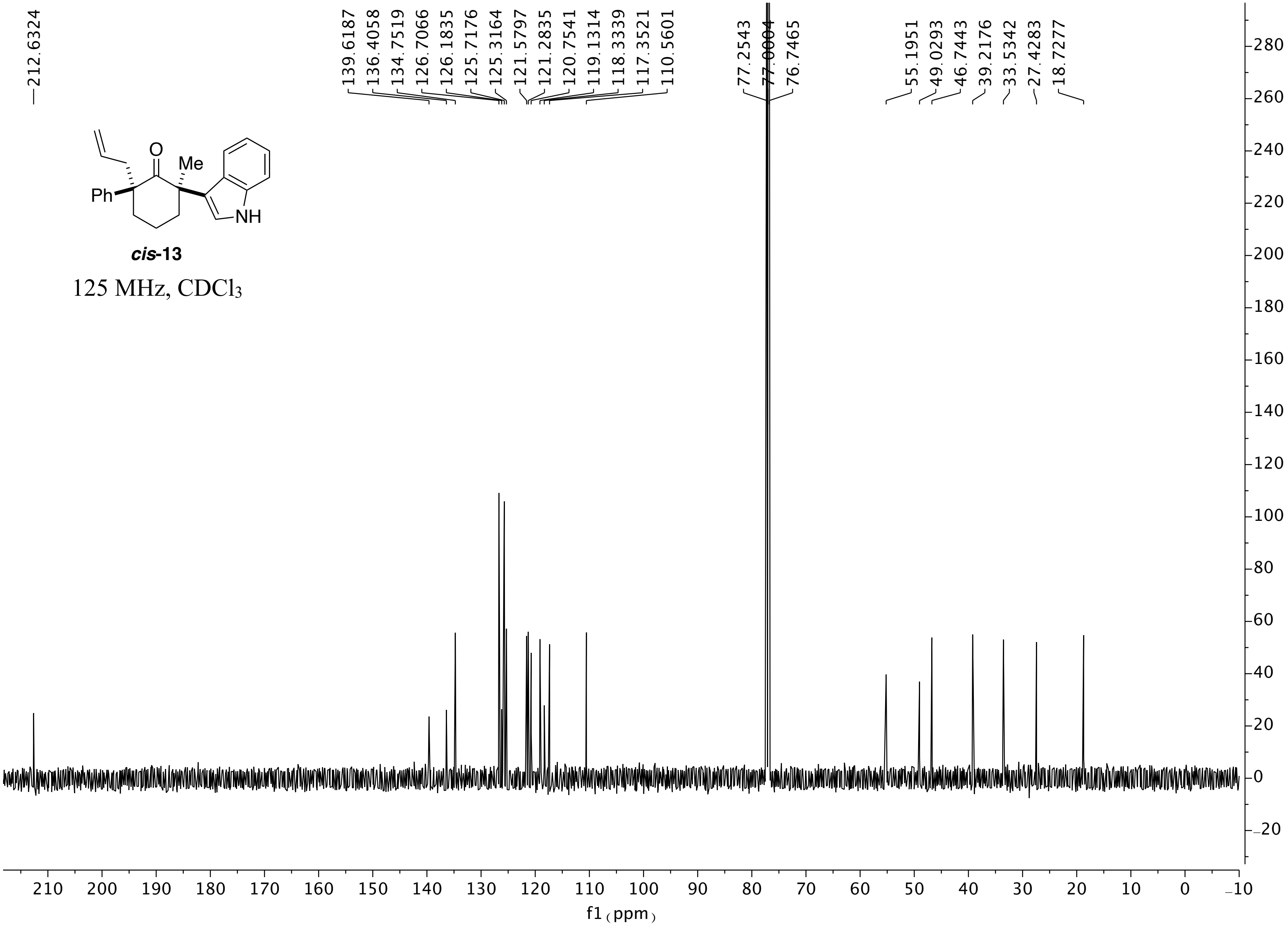

***cis*-13**

125 MHz, $CDCl_3$

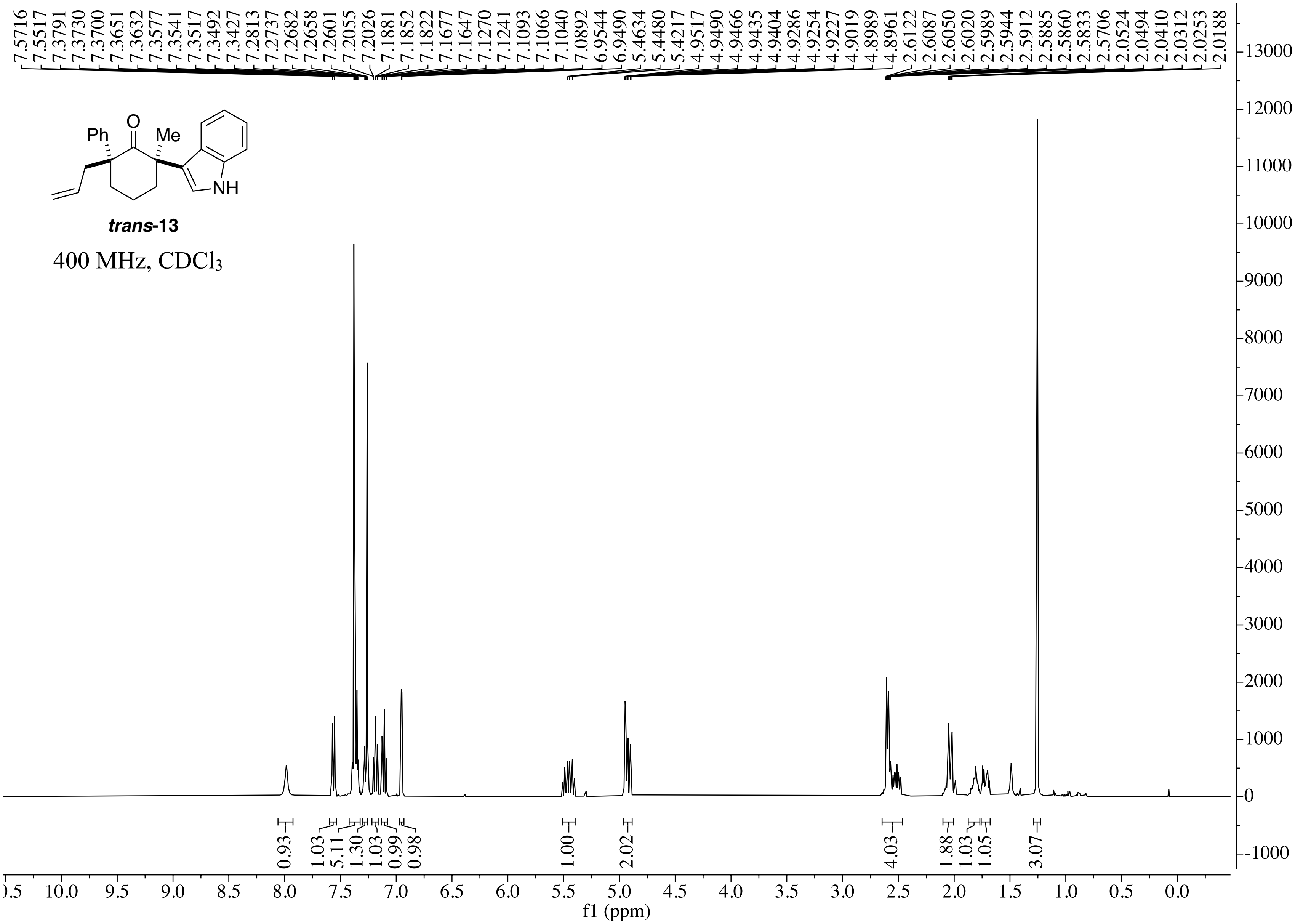
7.5716
7.5517
7.3791
7.3730
7.3700
7.3651
7.3632
7.3577
7.3541
7.3517
7.3492
7.3427
7.2813
7.2737
7.2682
7.2658
7.2601
7.2055
7.2026
7.1881
7.1852
7.1822
7.1677
7.1647
7.1270
7.1241
7.1093
7.1066
7.1040
7.0892
6.9544
6.9490
5.4634
5.4480
5.4217
4.9517
4.9490
4.9466
4.9435
4.9404
4.9286
4.9254
4.9227
4.9019
4.8989
4.8961
2.6122
2.6087
2.6050
2.6020
2.5989
2.5944
2.5912
2.5885
2.5860
2.5833
2.5706
2.0524
2.0494
2.0410
2.0312
2.0253
2.0188
Ph
O
Me
NH
trans-13
400 MHz, CDCl3
0.93
1.03
5.11
1.30
1.03
0.99
0.98
1.00
2.02
4.03
1.88
1.03
1.05
3.07
f1 (ppm)

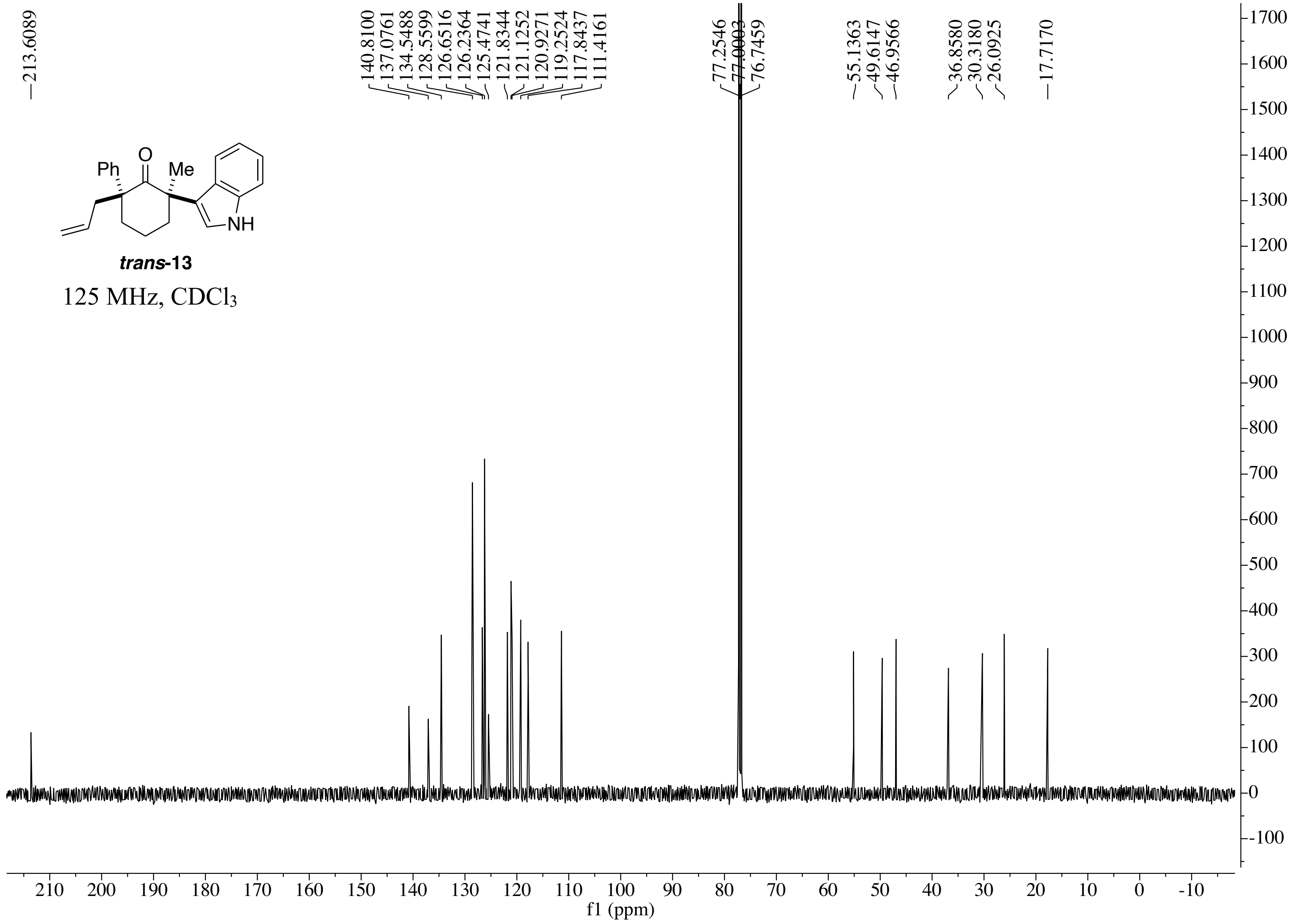

213.6089
140.8100
137.0761
134.5488
128.5599
126.6516
126.2364
125.4741
121.8344
121.1252
120.9271
119.2524
117.8437
111.4161
77.2546
77.0003
76.7459
55.1363
49.6147
46.9566
36.8580
30.3180
26.0925
17.7170
Ph
O
Me
NH
trans-13
125 MHz, CDCl3
f1 (ppm)

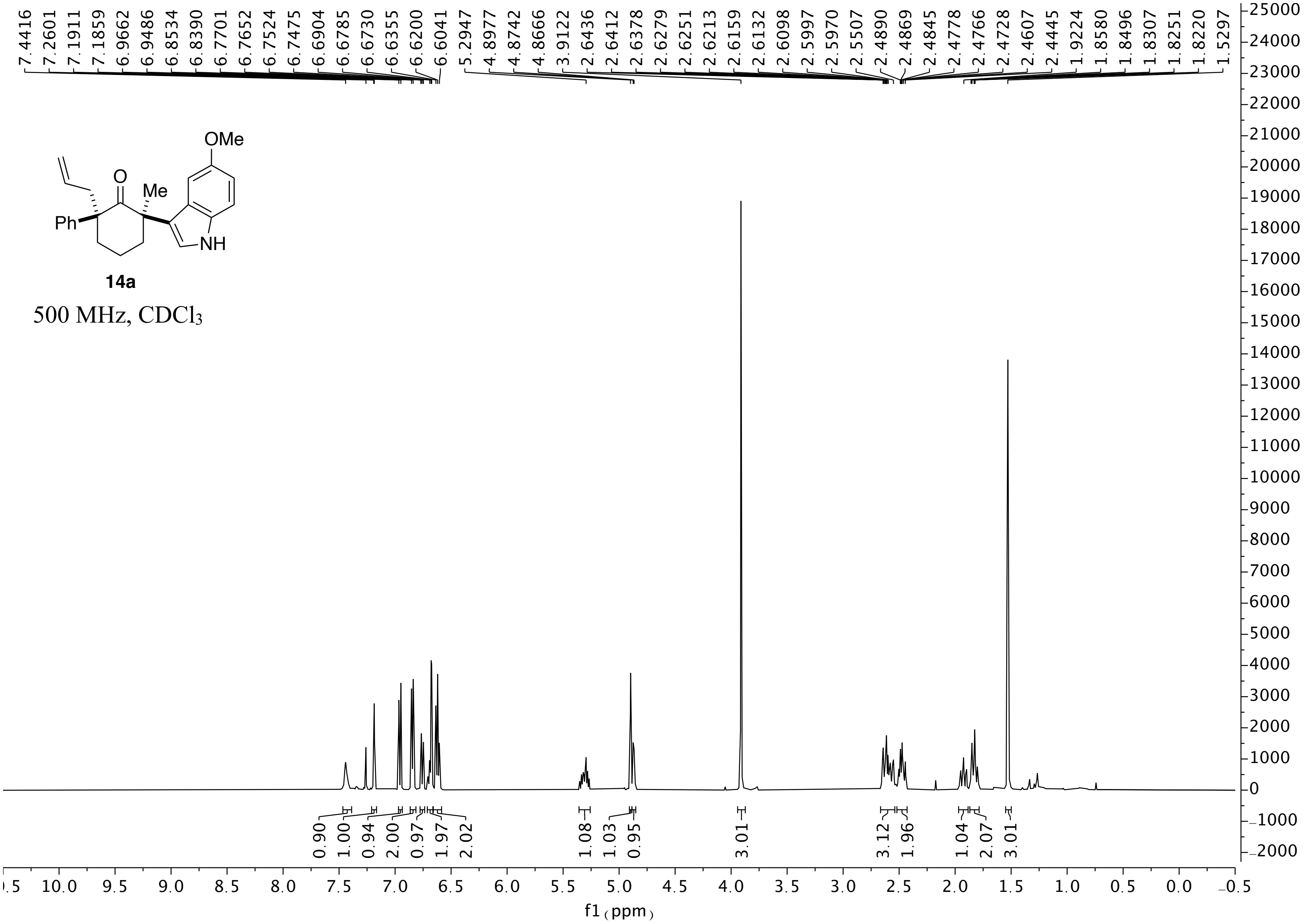

7.4416
7.2601
7.1911
7.1859
6.9662
6.9486
6.8534
6.8390
6.7701
6.7652
6.7524
6.7475
6.6904
6.6785
6.6730
6.6355
6.6200
6.6041
5.2947
4.8977
4.8742
4.8666
3.9122
2.6436
2.6412
2.6378
2.6279
2.6251
2.6213
2.6159
2.6132
2.6098
2.5997
2.5970
2.5507
2.4890
2.4869
2.4845
2.4778
2.4766
2.4728
2.4607
2.4445
1.9224
1.8580
1.8496
1.8307
1.8251
1.8220
1.5297
OMe
O
Me
Ph
NH
14a
500 MHz, CDCl3
0.90
1.00
0.94
2.00
0.97
1.97
2.02
1.08
1.03
0.95
3.01
3.12
1.96
1.04
2.07
3.01
f1 (ppm)

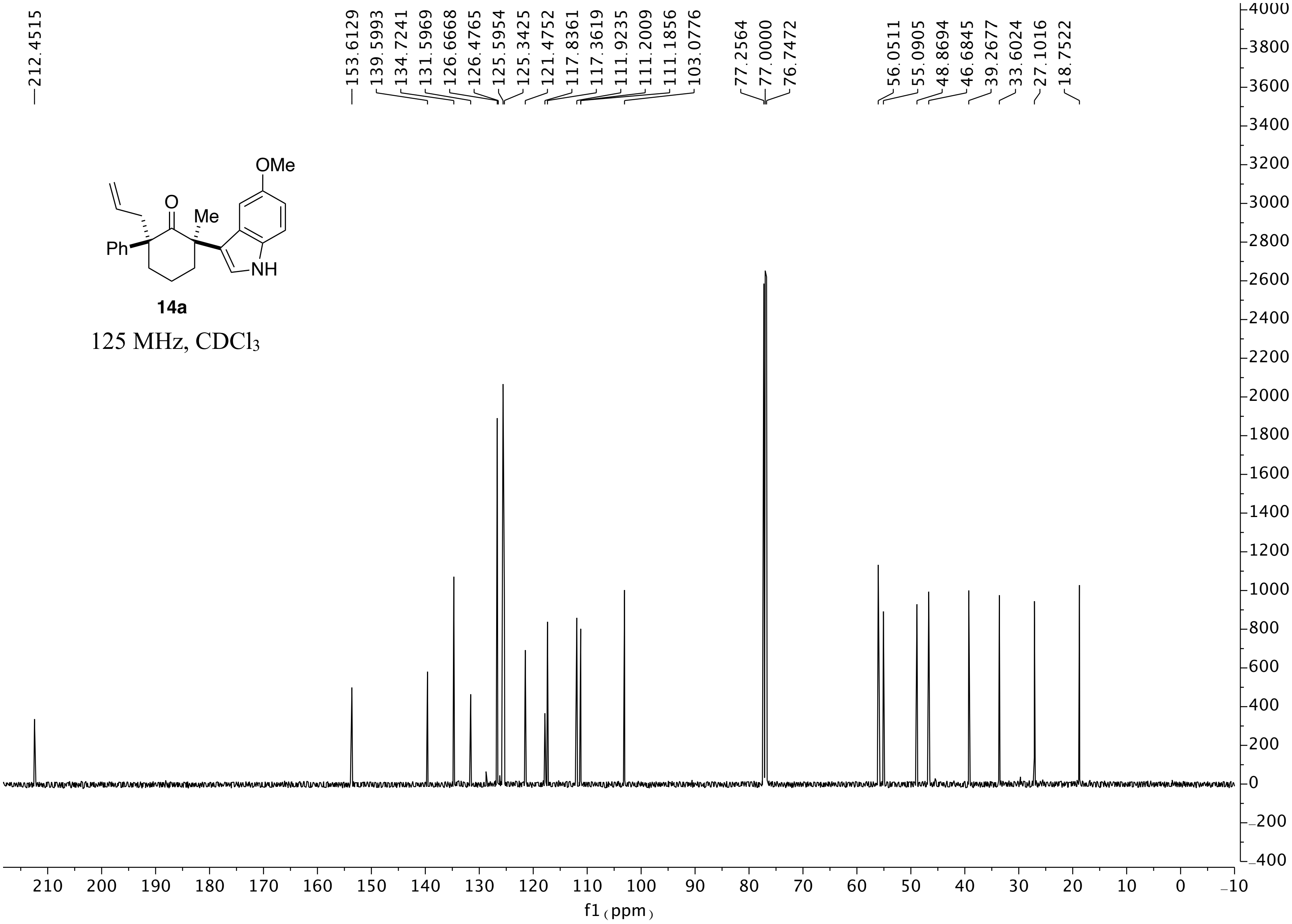

OMe
O
Me
Ph
NH
14a
125 MHz, CDCl3
212.4515
153.6129
139.5993
134.7241
131.5969
126.6668
126.4765
125.5954
125.3425
121.4752
117.8361
117.3619
111.9235
111.2009
111.1856
103.0776
77.2564
77.0000
76.7472
56.0511
55.0905
48.8694
46.6845
39.2677
33.6024
27.1016
18.7522
f1 (ppm)

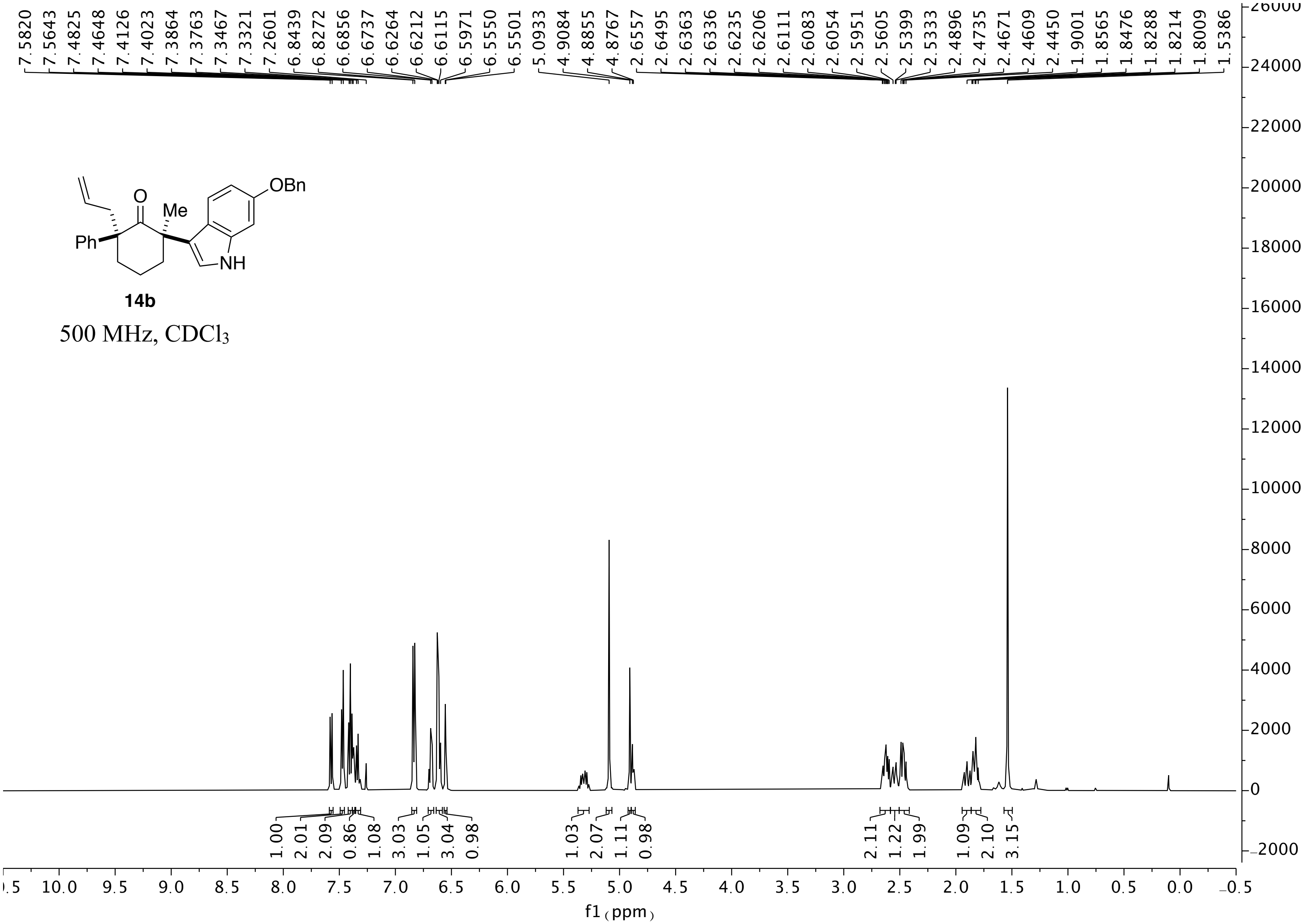

14b

500 MHz, $CDCl_3$

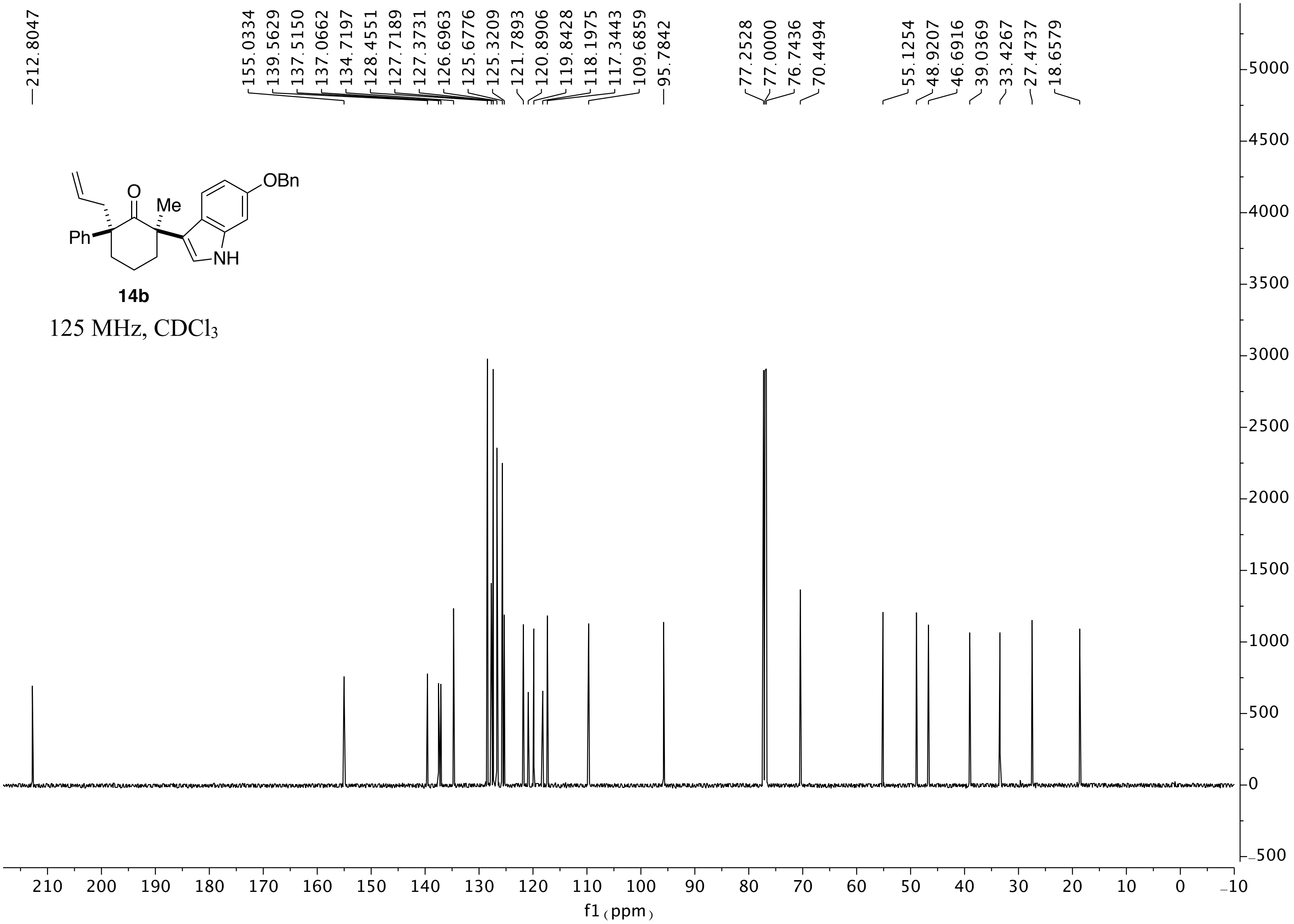

212.8047
155.0334
139.5629
137.5150
137.0662
134.7197
128.4551
127.7189
127.3731
126.6963
125.6776
125.3209
121.7893
120.8906
119.8428
118.1975
117.3443
109.6859
95.7842
77.2528
77.0000
76.7436
70.4494
55.1254
48.9207
46.6916
39.0369
33.4267
27.4737
18.6579
OBn
O
Me
Ph
NH
14b
125 MHz, CDCl3
f1 (ppm)

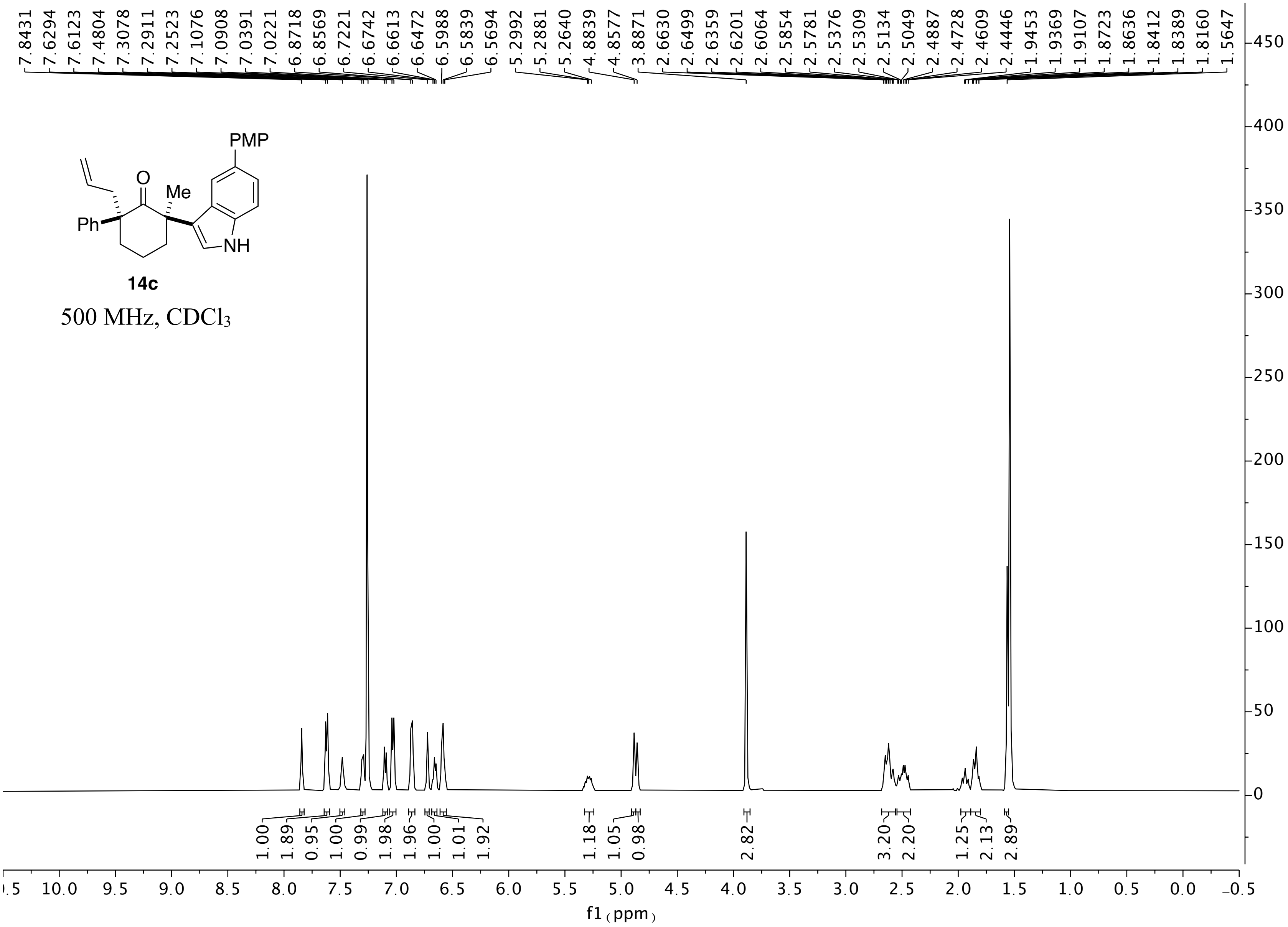
PMP
O
Me
Ph
NH
14c
500 MHz, CDCl3
f1 (ppm)

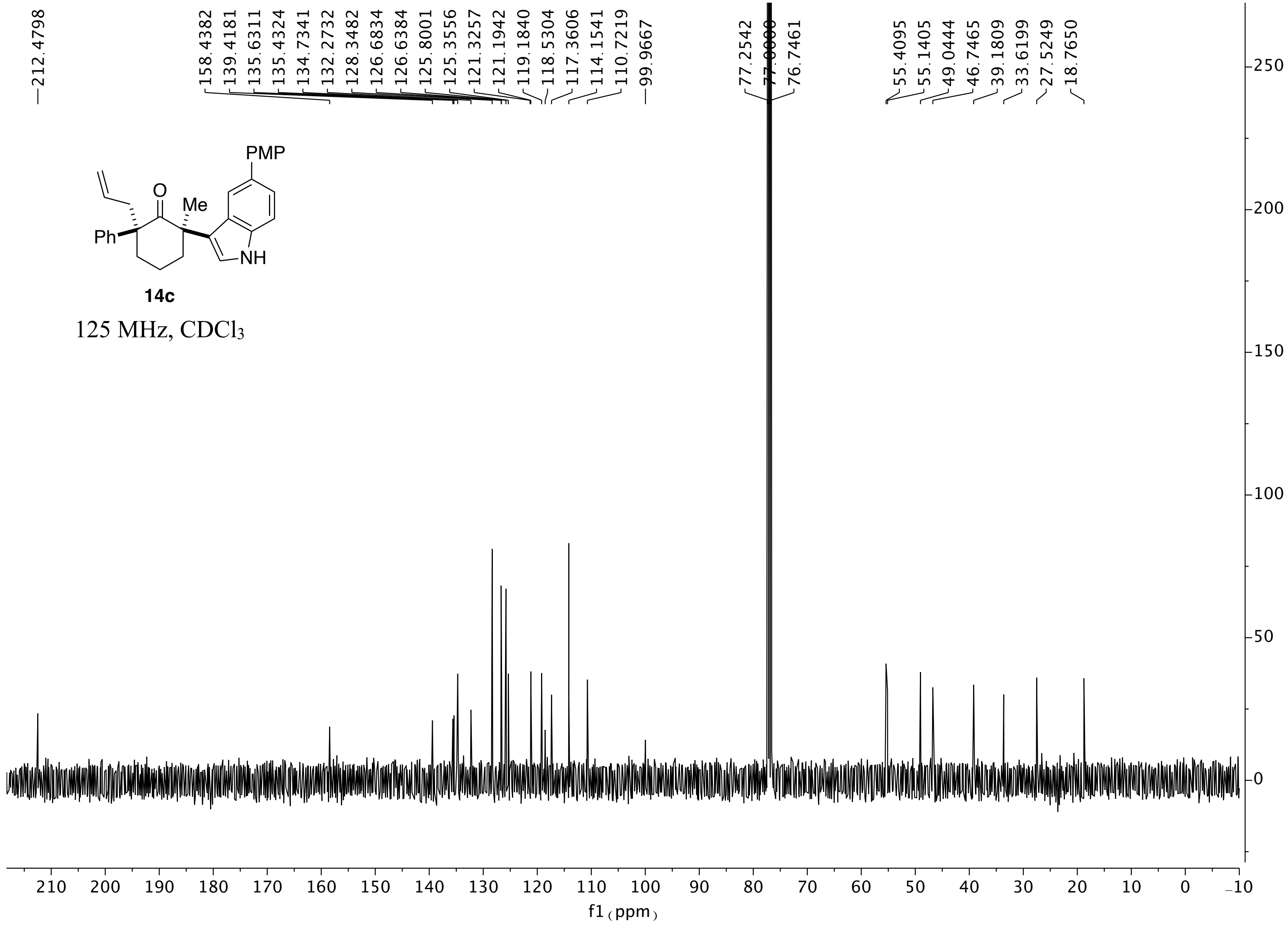

14c

125 MHz, $CDCl_3$

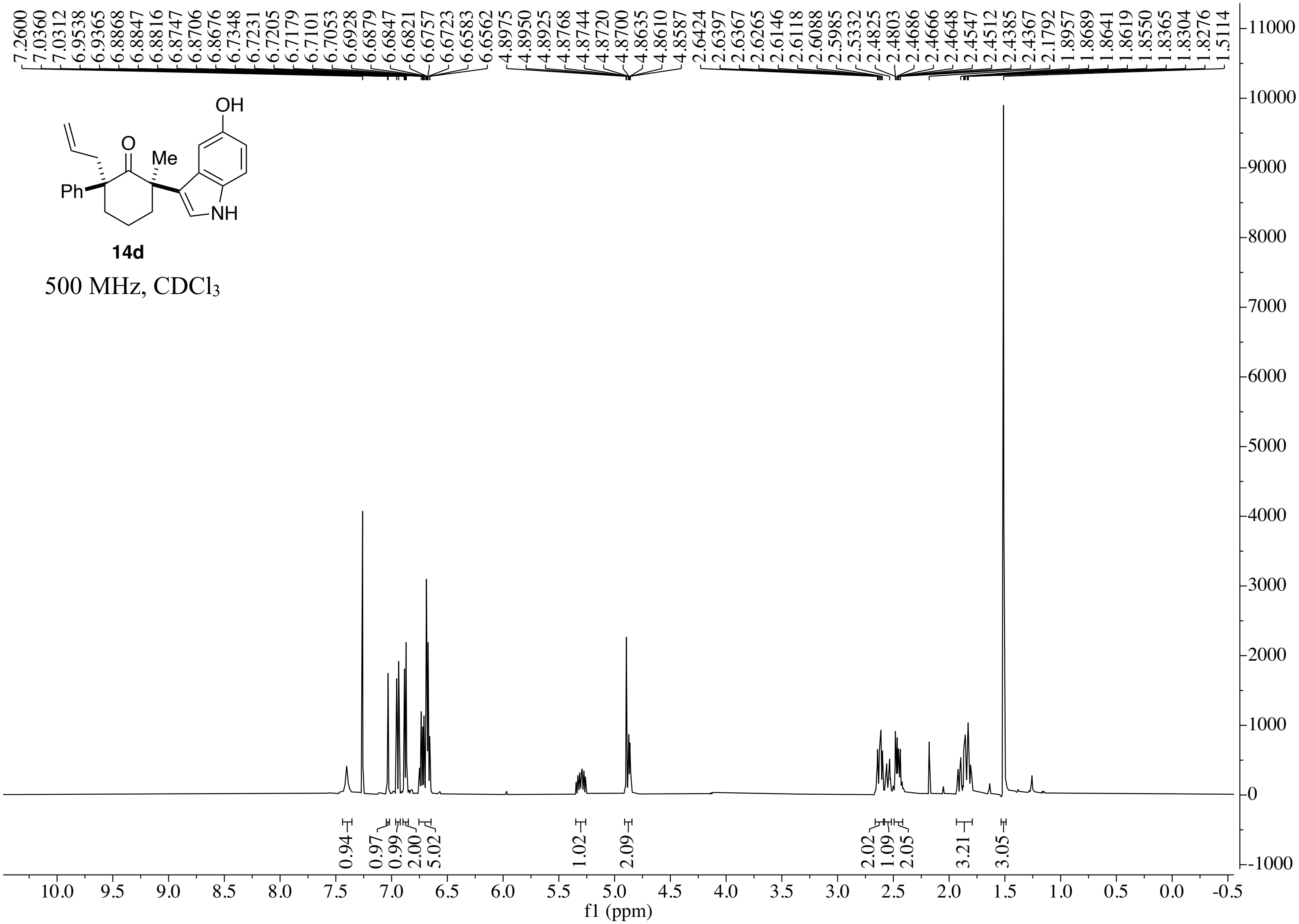

7.2600
7.0360
7.0312
6.9538
6.9365
6.8868
6.8847
6.8816
6.8747
6.8706
6.8676
6.7348
6.7231
6.7205
6.7179
6.7101
6.7053
6.6928
6.6879
6.6847
6.6821
6.6757
6.6723
6.6583
6.6562
4.8975
4.8950
4.8925
4.8768
4.8744
4.8720
4.8700
4.8635
4.8610
4.8587
2.6424
2.6397
2.6367
2.6265
2.6146
2.6118
2.6088
2.5985
2.5332
2.4825
2.4803
2.4686
2.4666
2.4648
2.4547
2.4512
2.4385
2.4367
2.1792
1.8957
1.8689
1.8641
1.8619
1.8550
1.8365
1.8304
1.8276
1.5114
OH
O
Me
Ph
NH
14d
500 MHz, $CDCl_3$
0.94
0.97
0.99
2.00
5.02
1.02
2.09
2.02
1.09
2.05
3.21
3.05
f1 (ppm)

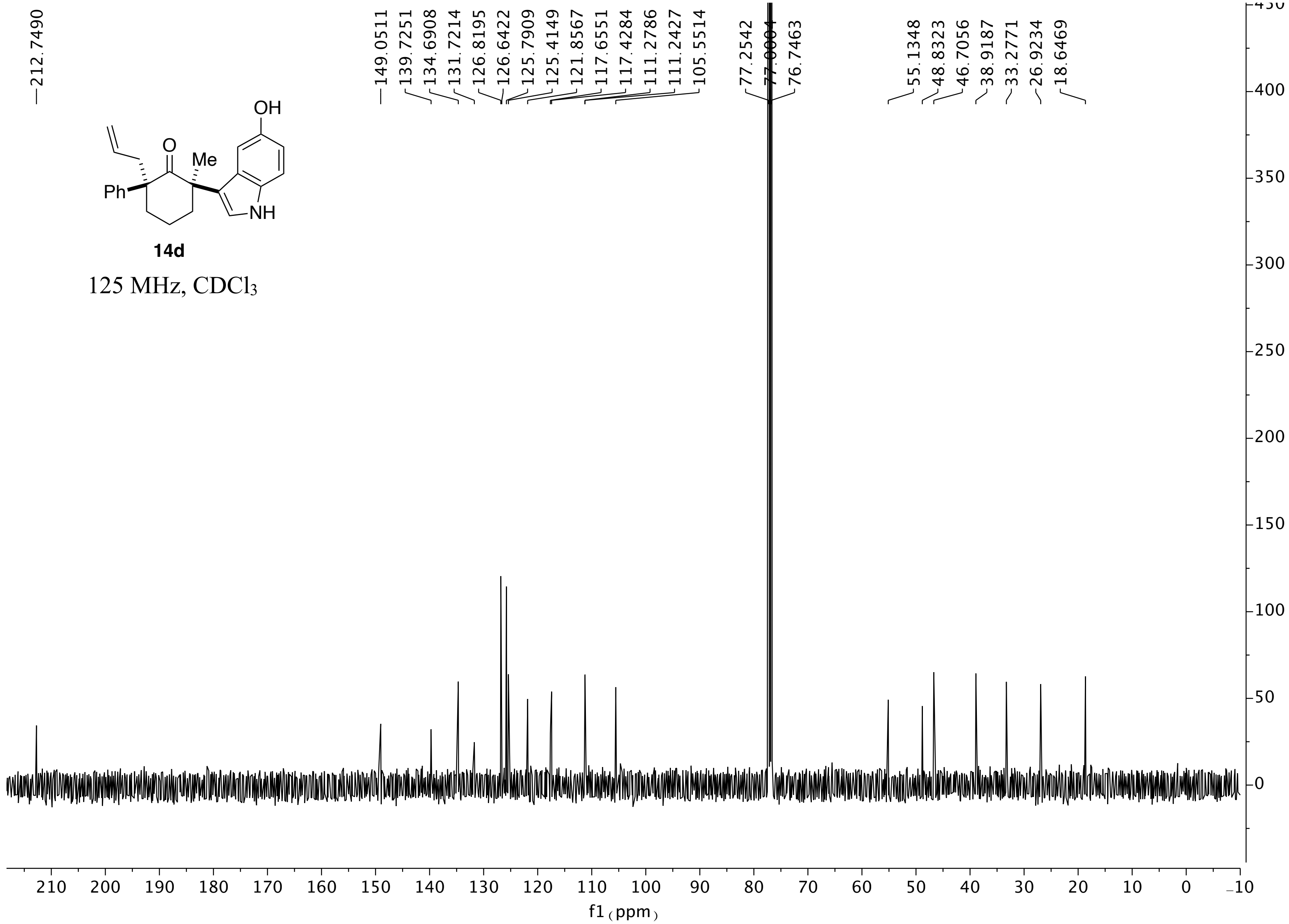
212.7490
149.0511
139.7251
134.6908
131.7214
126.8195
126.6422
125.7909
125.4149
121.8567
117.6551
117.4284
111.2786
111.2427
105.5514
77.2542
77.0004
76.7463
55.1348
48.8323
46.7056
38.9187
33.2771
26.9234
18.6469
OH
O
Me
Ph
NH
14d
125 MHz, CDCl3
f1 (ppm)

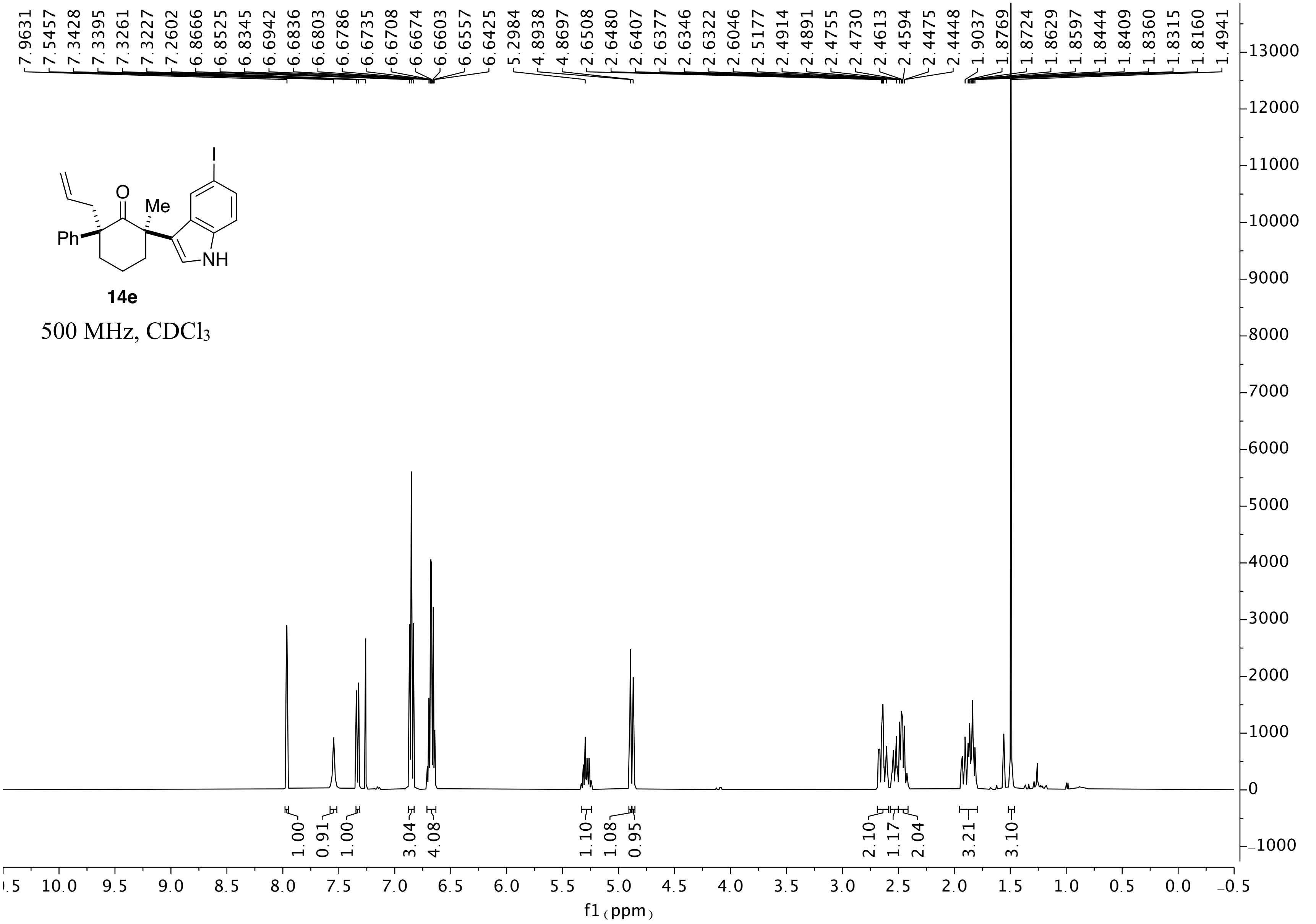
7.9631 7.5457 7.3428 7.3395 7.3261 7.3227 7.2602 6.8666 6.8525 6.8345 6.6942 6.6836 6.6803 6.6786 6.6735 6.6708 6.6674 6.6603 6.6557 6.6425 5.2984 4.8938 4.8697 2.6508 2.6480 2.6407 2.6377 2.6346 2.6322 2.6046 2.5177 2.4914 2.4891 2.4755 2.4730 2.4613 2.4594 2.4475 2.4448 1.9037 1.8769 1.8724 1.8629 1.8597 1.8444 1.8409 1.8360 1.8315 1.8160 1.4941
I
O
Me
Ph
NH
14e
500 MHz, CDCl3
1.00 0.91 1.00 3.04 4.08 1.10 1.08 0.95 2.10 1.17 2.04 3.21 3.10
f1 (ppm)

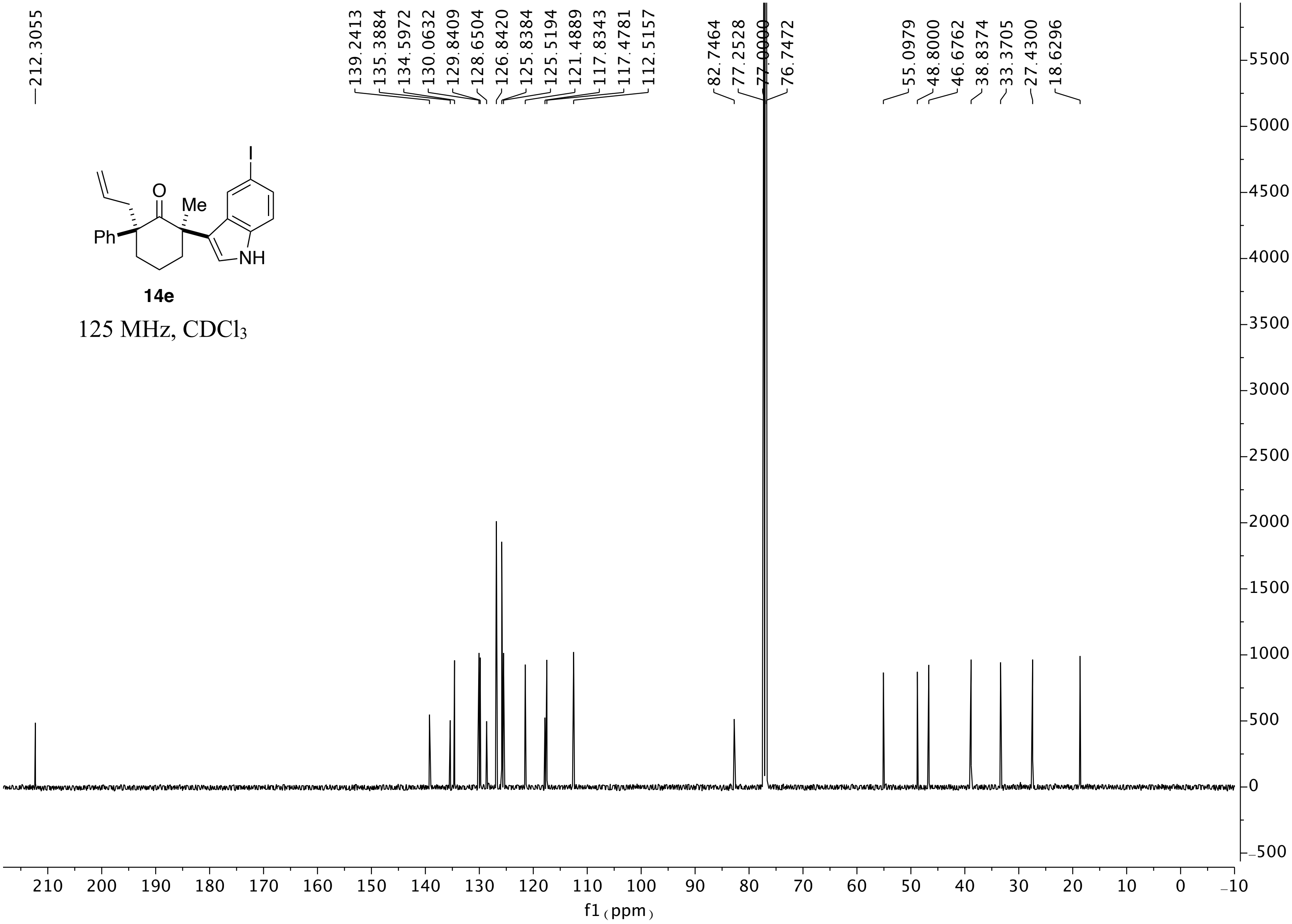
212.3055
139.2413
135.3884
134.5972
130.0632
129.8409
128.6504
126.8420
125.8384
125.5194
121.4889
117.8343
117.4781
112.5157
82.7464
77.2528
77.0000
76.7472
55.0979
48.8000
46.6762
38.8374
33.3705
27.4300
18.6296
O
Me
I
Ph
NH
14e
125 MHz, CDCl3
f1 (ppm)

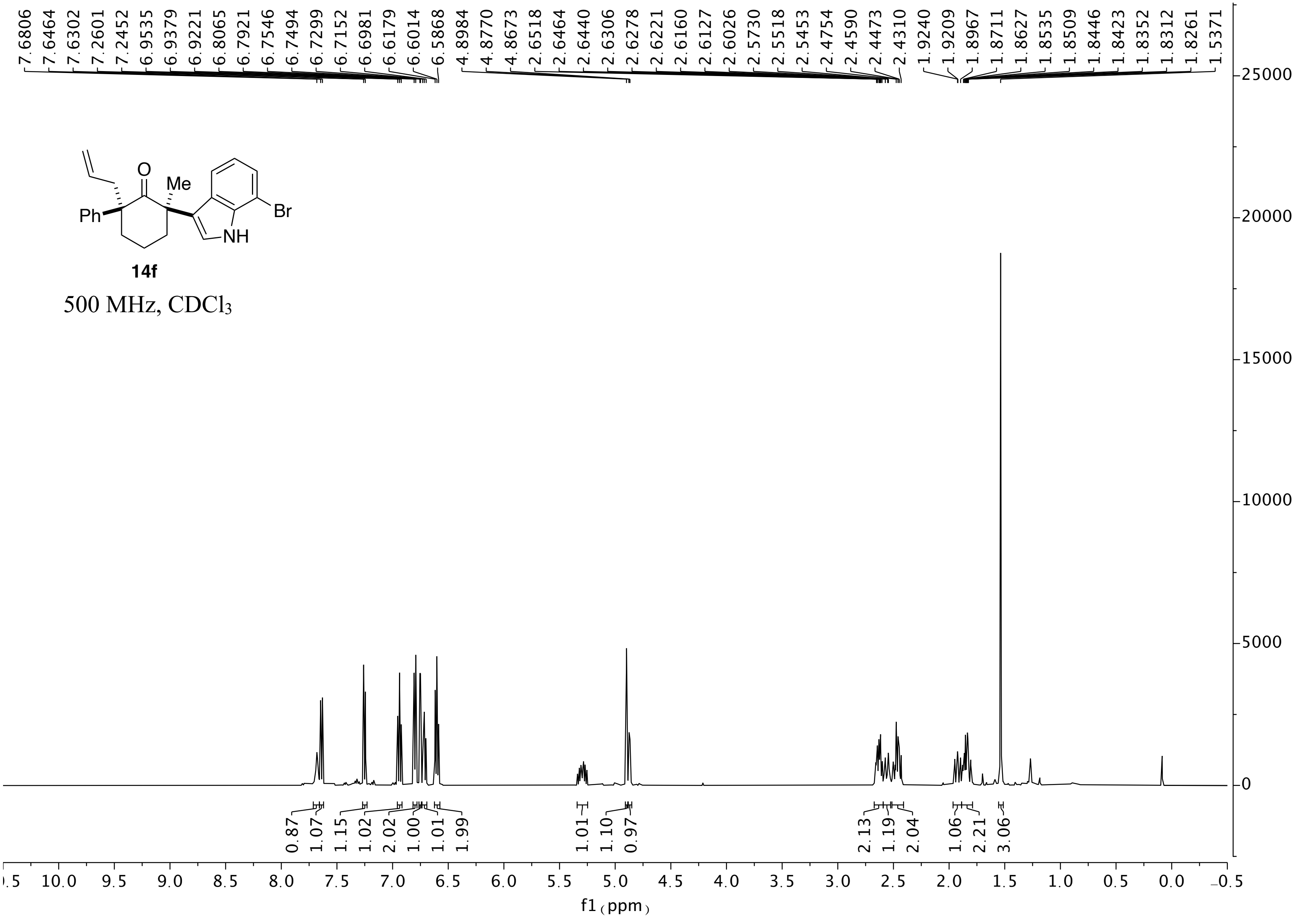
7.6806 7.6464 7.6302 7.2601 7.2452 6.9535 6.9379 6.9221 6.8065 6.7921 6.7546 6.7494 6.7299 6.7152 6.6981 6.6179 6.6014 6.5868
4.8984 4.8770 4.8673
2.6518 2.6464 2.6440 2.6306 2.6278 2.6221 2.6160 2.6127 2.6026 2.5730 2.5518 2.5453 2.4754 2.4590 2.4473 2.4310
1.9240 1.9209 1.8967 1.8711 1.8627 1.8535 1.8509 1.8446 1.8423 1.8352 1.8312 1.8261 1.5371
O
Me
Ph
Br
NH
14f
500 MHz, $CDCl_3$
0.87 1.07 1.15 1.02 2.02 2.00 1.01 1.99
1.01 1.10 0.97
2.13 1.19 2.04
1.06 2.21 3.06
25000
20000
15000
10000
5000
0
10.0 9.5 9.0 8.5 8.0 7.5 7.0 6.5 6.0 5.5 5.0 4.5 4.0 3.5 3.0 2.5 2.0 1.5 1.0 0.5 0.0 -0.5
f1 (ppm)

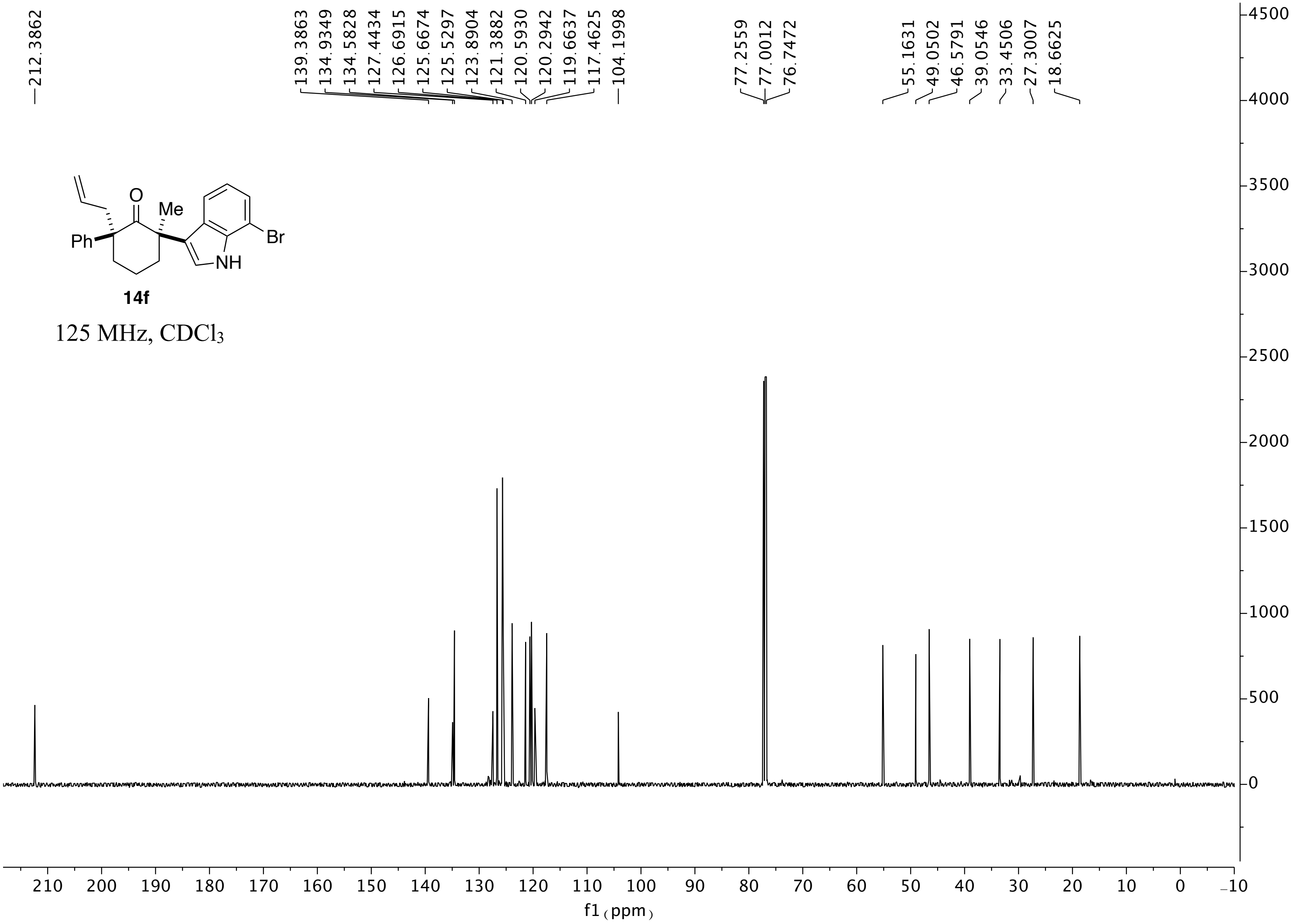

14f

125 MHz, CDCl3

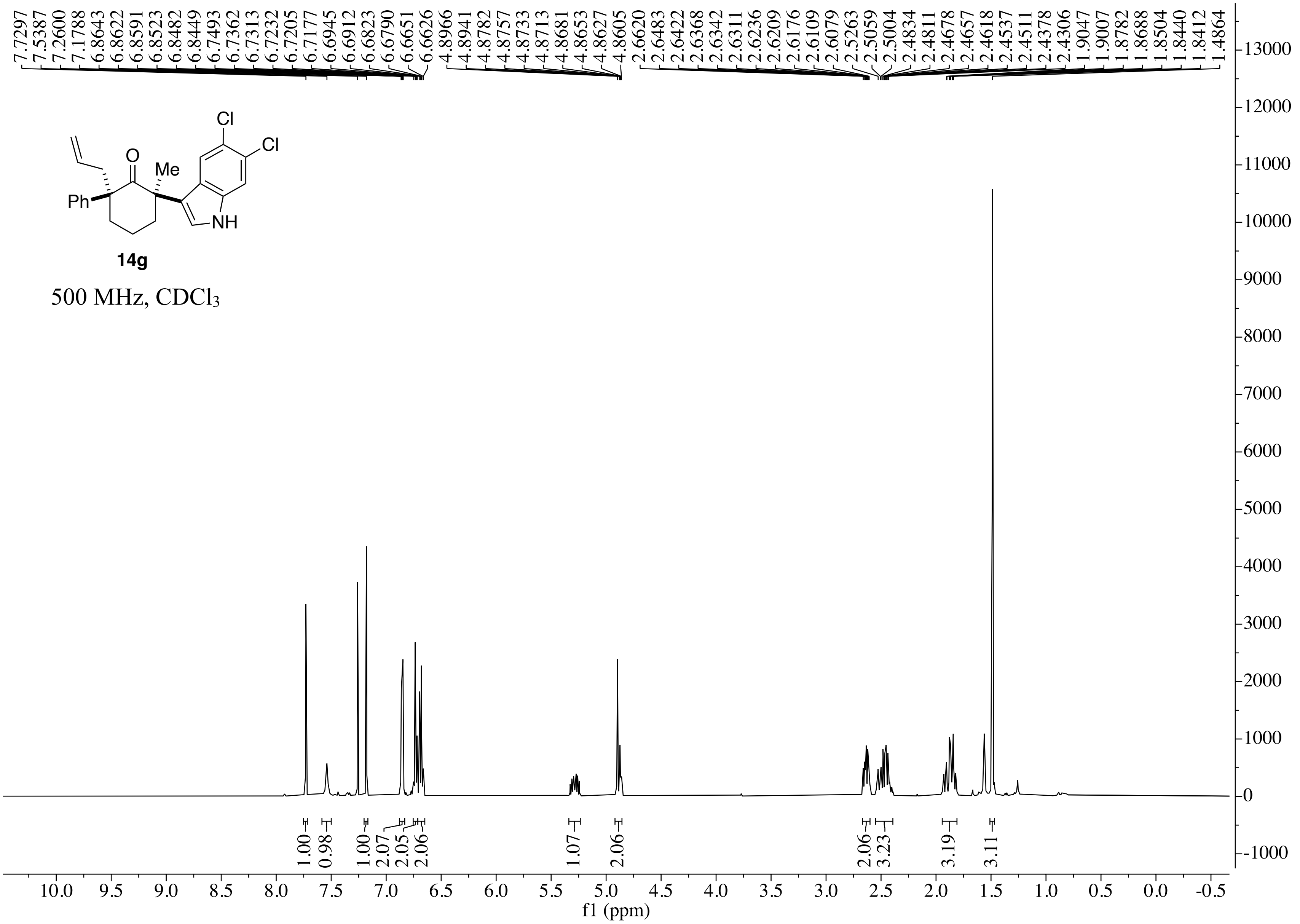
7.7297 7.5387 7.2600 7.1788 6.8643 6.8622 6.8591 6.8523 6.8482 6.8449 6.7493 6.7362 6.7313 6.7232 6.7205 6.7177 6.6945 6.6912 6.6823 6.6790 6.6651 6.6626
4.8966 4.8941 4.8782 4.8757 4.8733 4.8713 4.8681 4.8653 4.8627 4.8605
2.6620 2.6483 2.6422 2.6368 2.6342 2.6311 2.6236 2.6209 2.6176 2.6109 2.6079 2.5263 2.5059 2.5004 2.4834 2.4811 2.4678 2.4657 2.4618 2.4537 2.4511 2.4378 2.4306 1.9047 1.9007 1.8782 1.8688 1.8504 1.8440 1.8412 1.4864
Cl
Cl
O
Me
Ph
NH
14g
500 MHz, CDCl3
1.00 0.98 1.00 2.07 2.05 2.06 1.07 2.06 2.06 3.23 3.19 3.11
f1 (ppm)

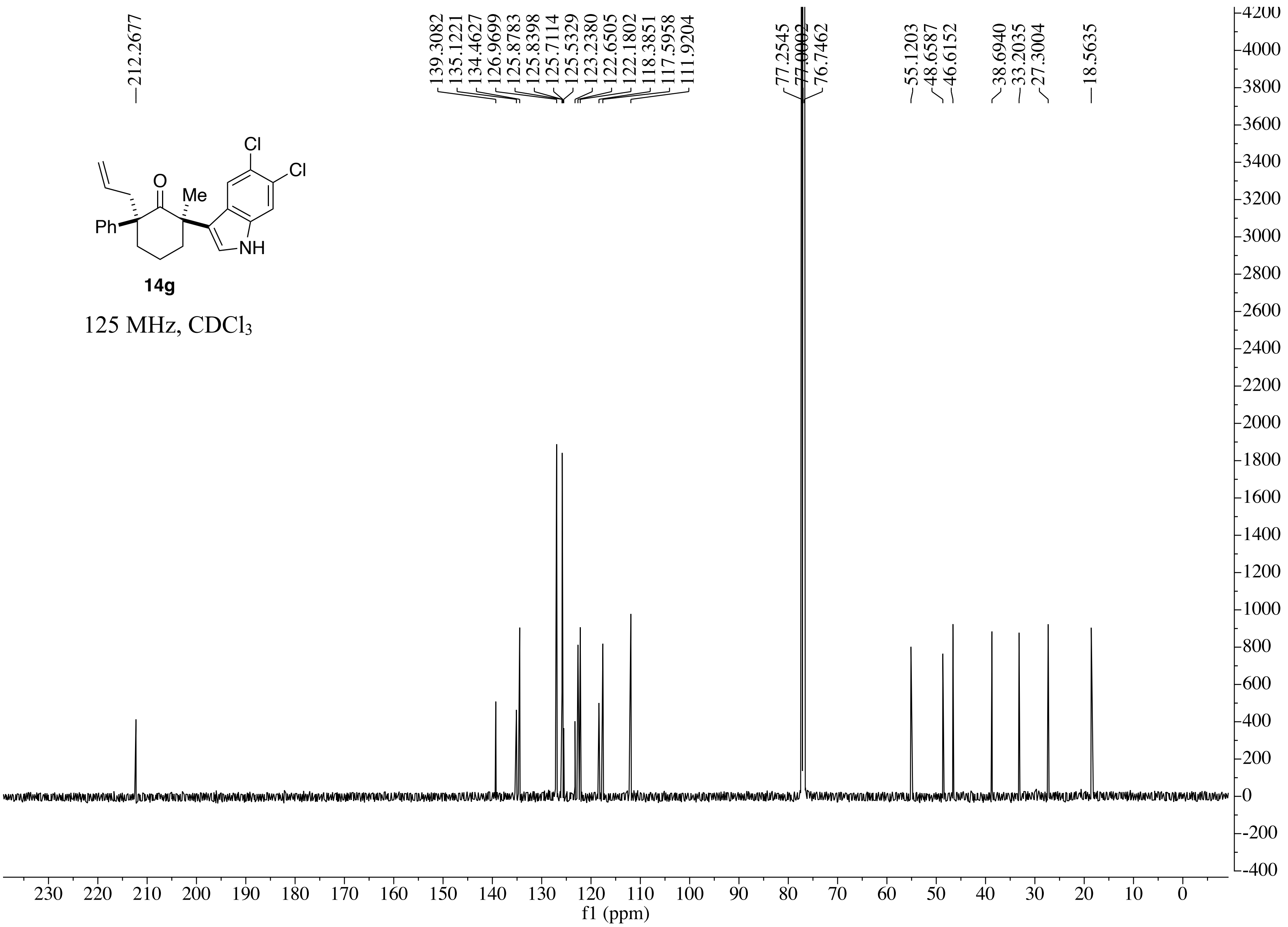
212.2677
139.3082
135.1221
134.4627
126.9699
125.8783
125.8398
125.7114
125.5329
123.2380
122.6505
122.1802
118.3851
117.5958
111.9204
77.2545
77.0002
76.7462
55.1203
48.6587
46.6152
38.6940
33.2035
27.3004
18.5635
Cl
Cl
O
Me
Ph
NH
14g
125 MHz, CDCl3
f1 (ppm)

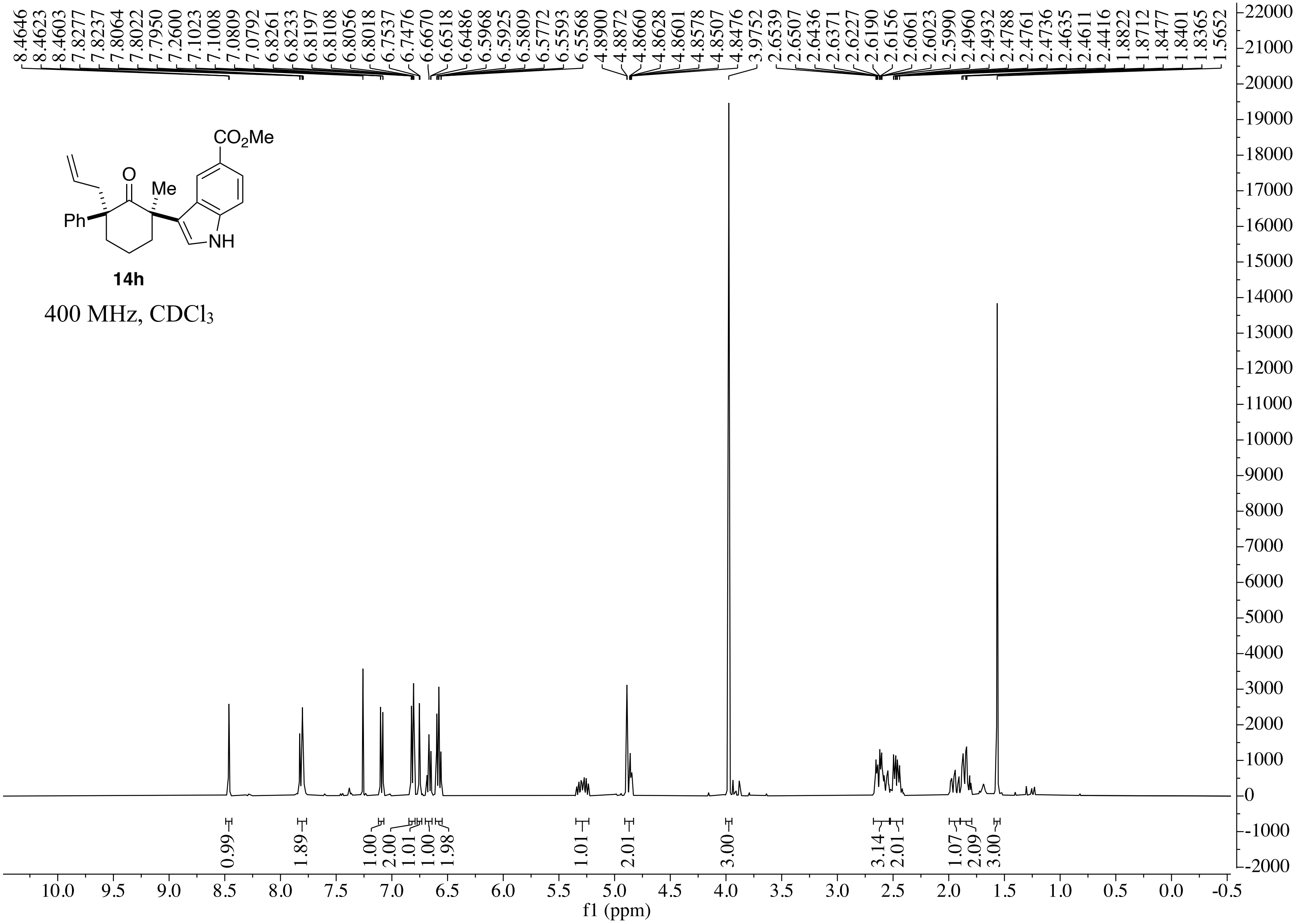
8.4646
8.4623
8.4603
7.8277
7.8237
7.8064
7.8022
7.7950
7.2600
7.1023
7.1008
7.0809
7.0792
6.8261
6.8233
6.8197
6.8108
6.8056
6.8018
6.7537
6.7476
6.6670
6.6518
6.6486
6.5968
6.5925
6.5809
6.5772
6.5593
6.5568
4.8900
4.8872
4.8660
4.8628
4.8601
4.8578
4.8507
4.8476
3.9752
2.6539
2.6507
2.6436
2.6371
2.6227
2.6190
2.6156
2.6061
2.6023
2.5990
2.4960
2.4932
2.4788
2.4761
2.4736
2.4635
2.4611
2.4416
1.8822
1.8712
1.8477
1.8401
1.8365
1.5652
CO2Me
O
Me
Ph
NH
14h
400 MHz, CDCl3
0.99
1.89
1.00
2.00
1.01
1.00
1.98
1.01
2.01
3.00
3.14
2.01
1.07
2.09
3.00
f1 (ppm)

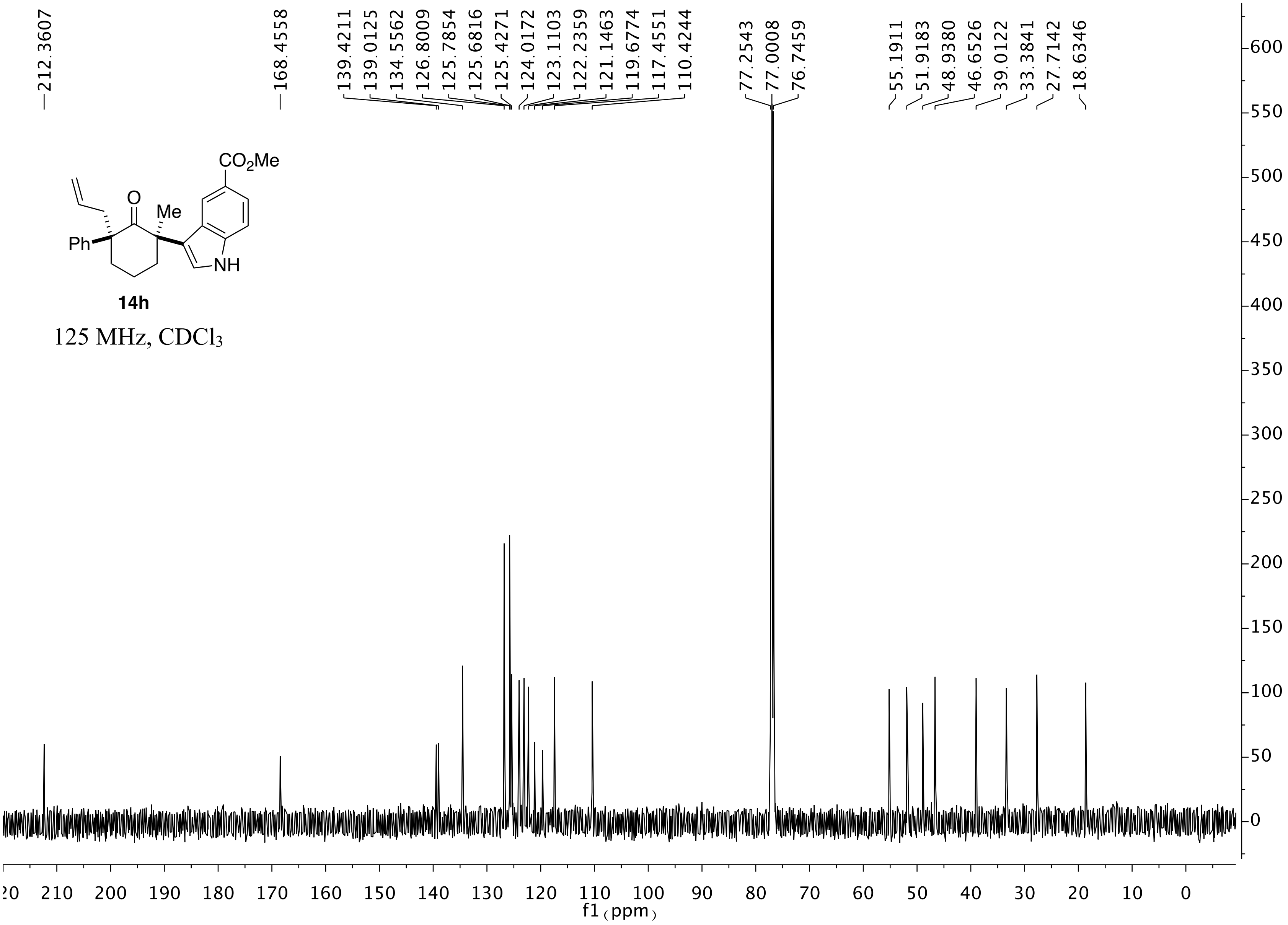
212.3607
168.4558
139.4211
139.0125
134.5562
126.8009
125.7854
125.6816
125.4271
124.0172
123.1103
122.2359
121.1463
119.6774
117.4551
110.4244
77.2543
77.0008
76.7459
55.1911
51.9183
48.9380
46.6526
39.0122
33.3841
27.7142
18.6346
CO2Me
O
Me
Ph
NH
14h
125 MHz, CDCl3
f1 (ppm)

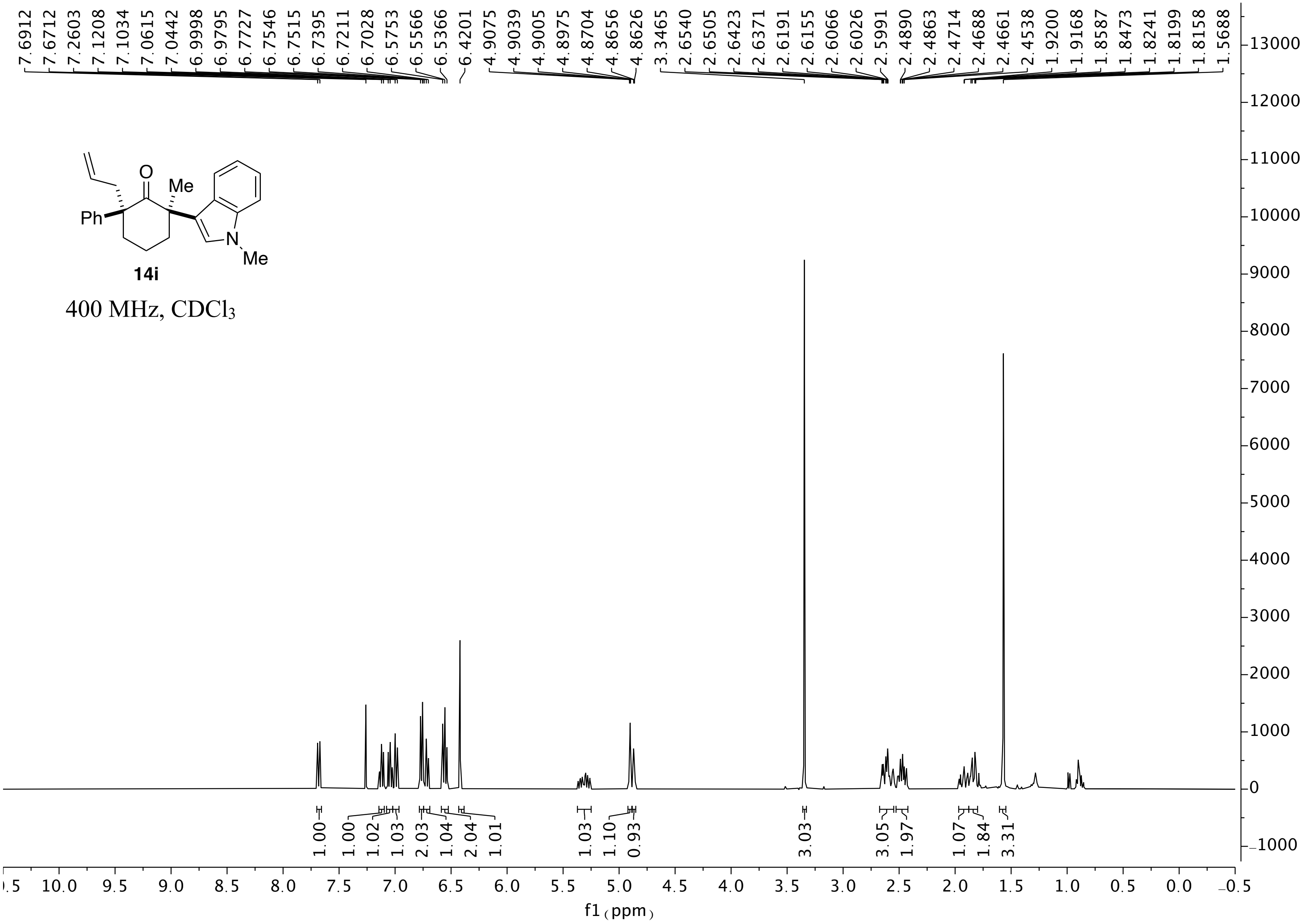

14i
400 MHz, CDCl3
f1 (ppm)

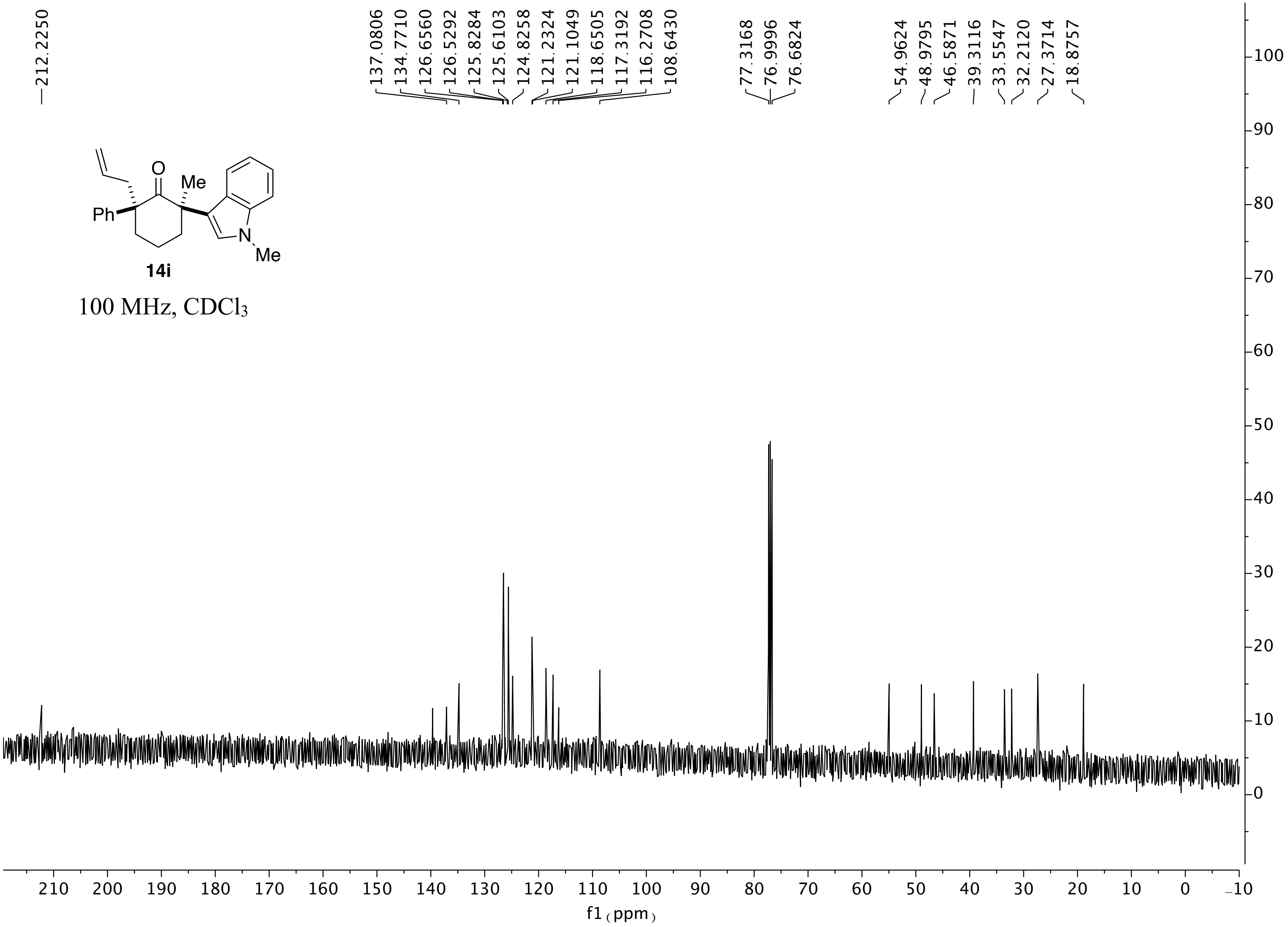
212.2250
137.0806
134.7710
126.6560
126.5292
125.8284
125.6103
124.8258
121.2324
121.1049
118.6505
117.3192
116.2708
108.6430
77.3168
76.9996
76.6824
54.9624
48.9795
46.5871
39.3116
33.5547
32.2120
27.3714
18.8757
O
Me
Ph
N
Me
14i
100 MHz, CDCl3
f1 (ppm)

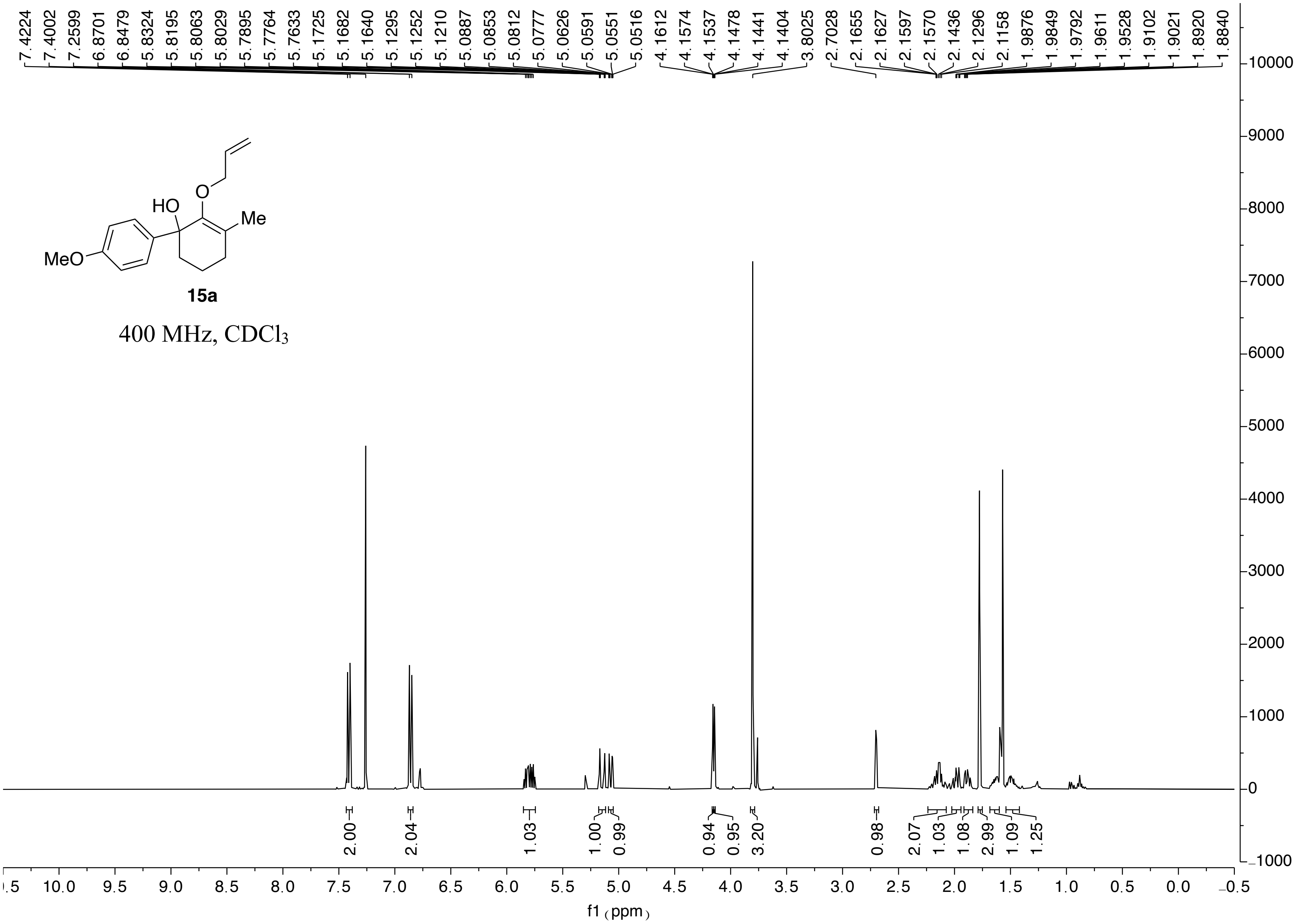

15a

400 MHz, $CDCl_3$

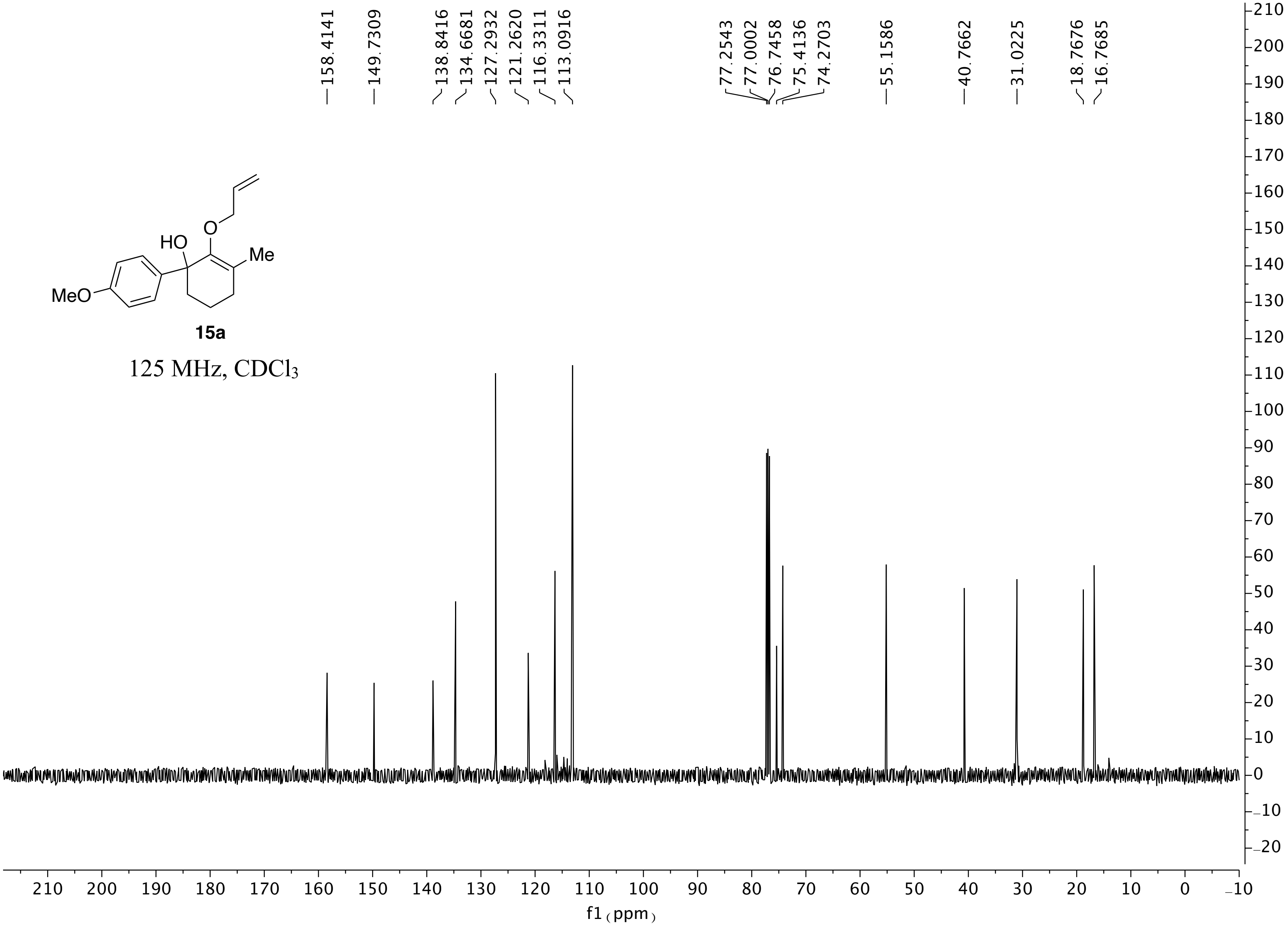
158.4141
149.7309
138.8416
134.6681
127.2932
121.2620
116.3311
113.0916
77.2543
77.0002
76.7458
75.4136
74.2703
55.1586
40.7662
31.0225
18.7676
16.7685
HO
O
Me
MeO
15a
125 MHz, CDCl3
f1 (ppm)

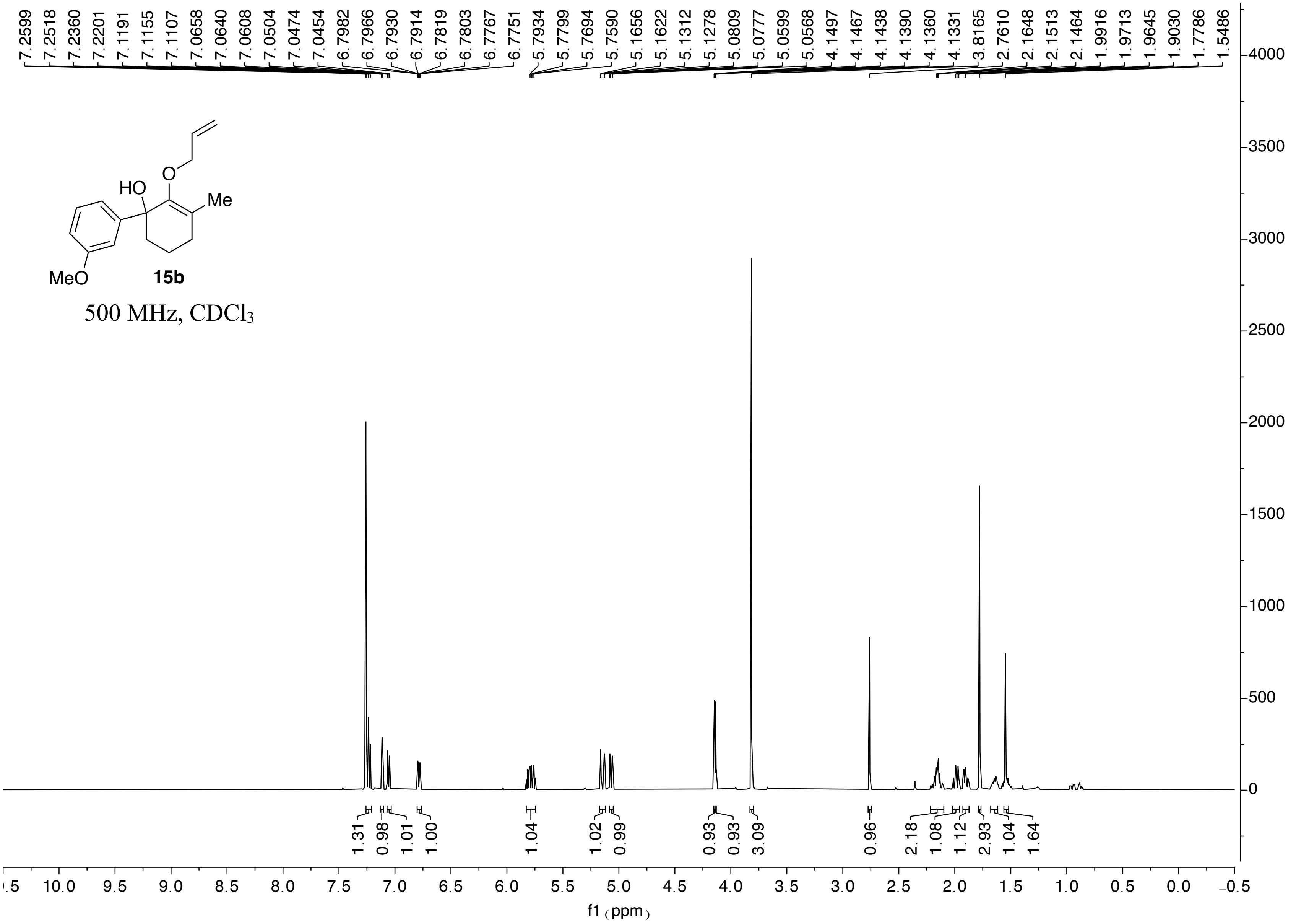

HO
O
Me
MeO
15b
500 MHz, CDCl3
f1 (ppm)

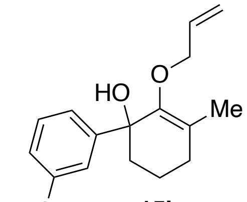

15b

125 MHz, $CDCl_3$

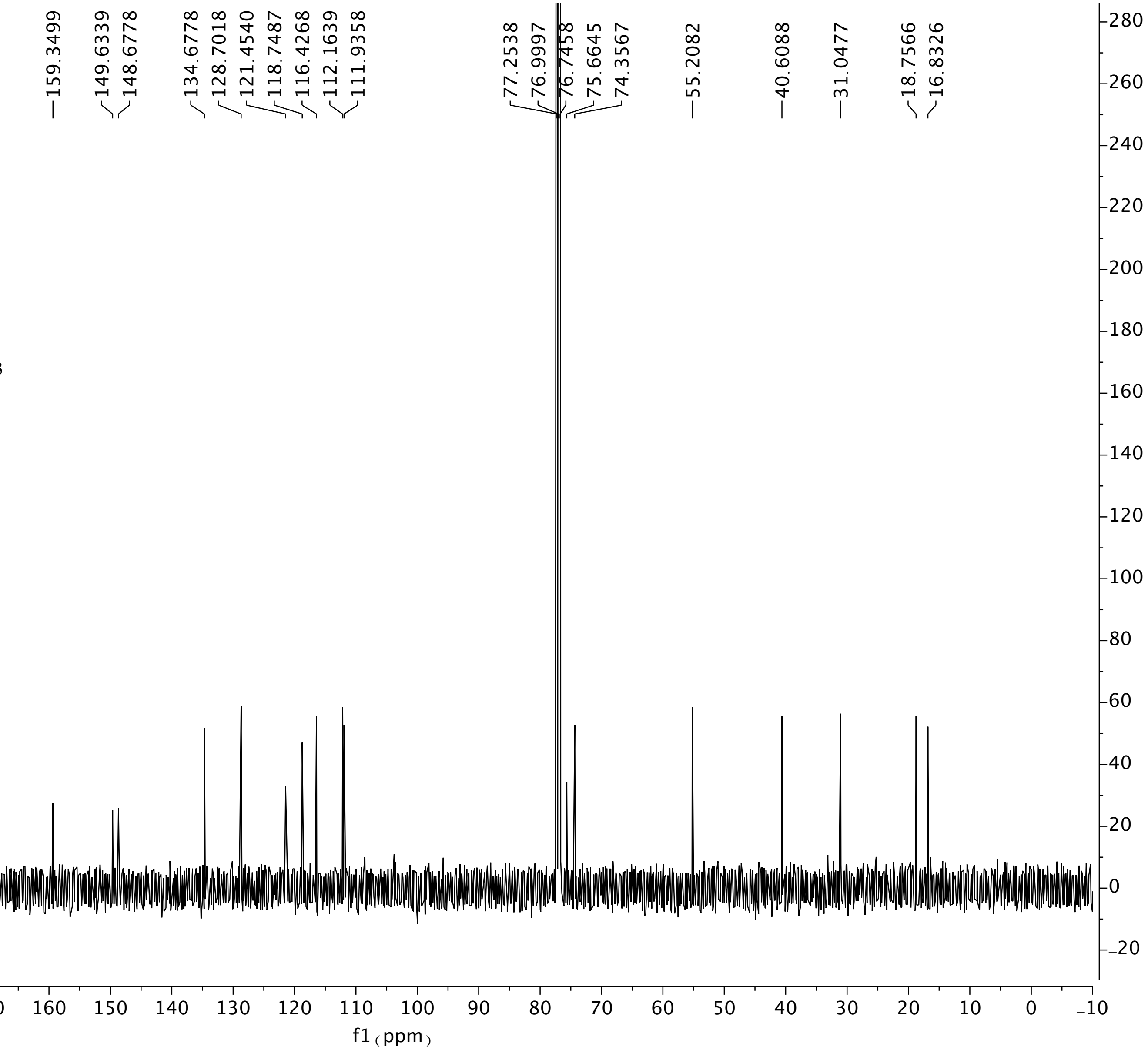

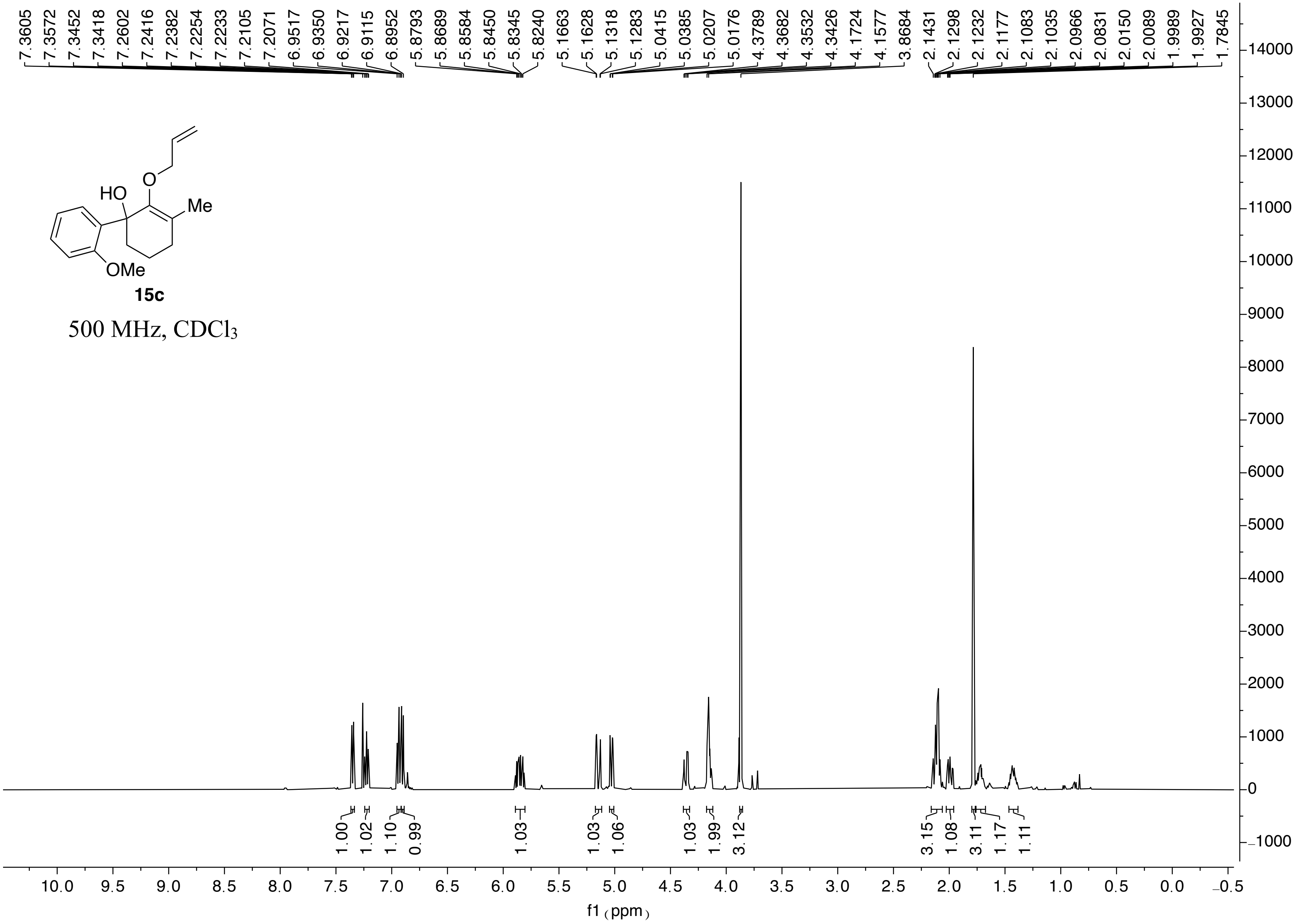

15c

500 MHz, $CDCl_3$

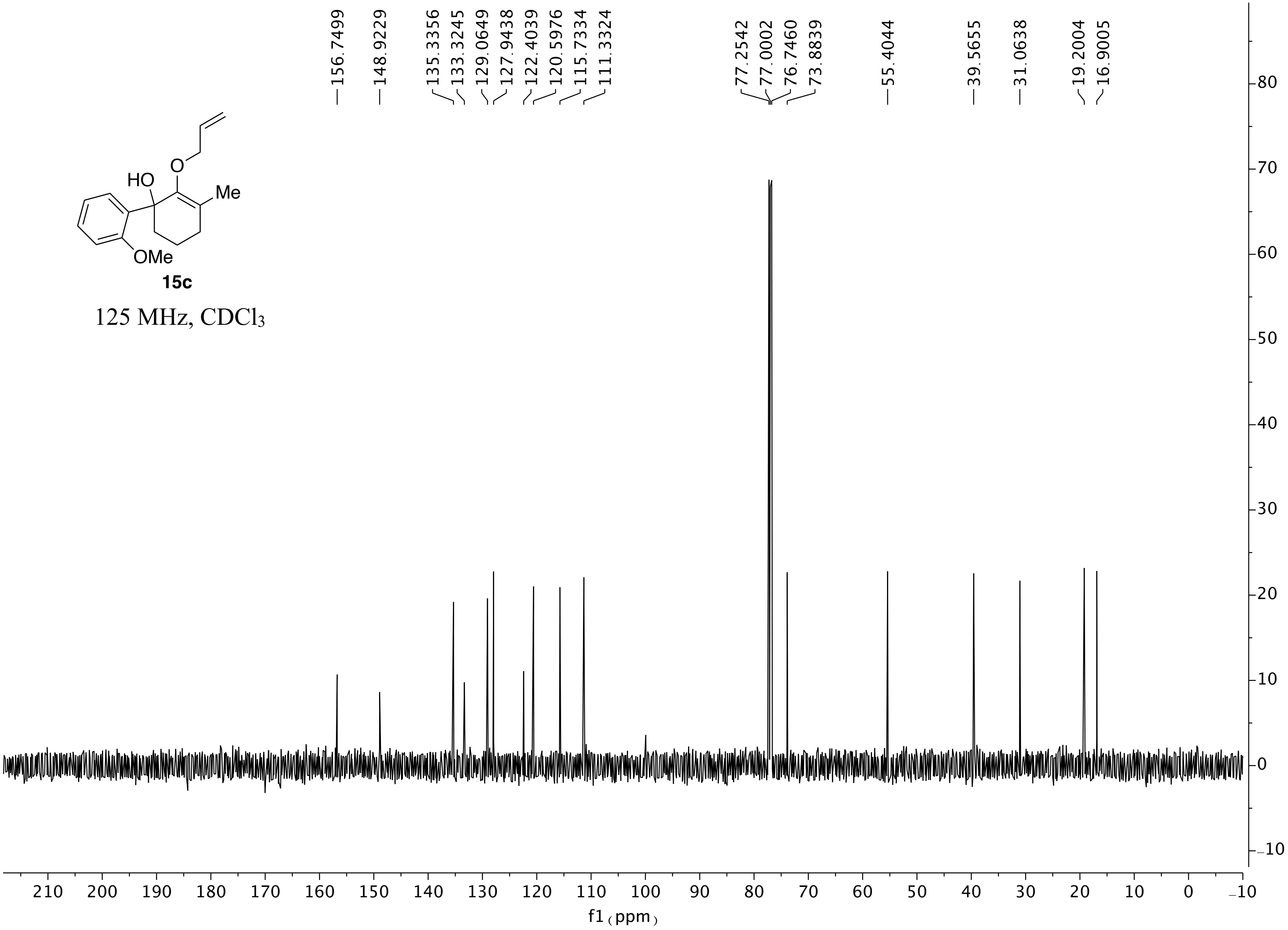
HO
O
Me
OMe
15c
125 MHz, CDCl3
156.7499
148.9229
135.3356
133.3245
129.0649
127.9438
122.4039
120.5976
115.7334
111.3324
77.2542
77.0002
76.7460
73.8839
55.4044
39.5655
31.0638
19.2004
16.9005
f1 (ppm)

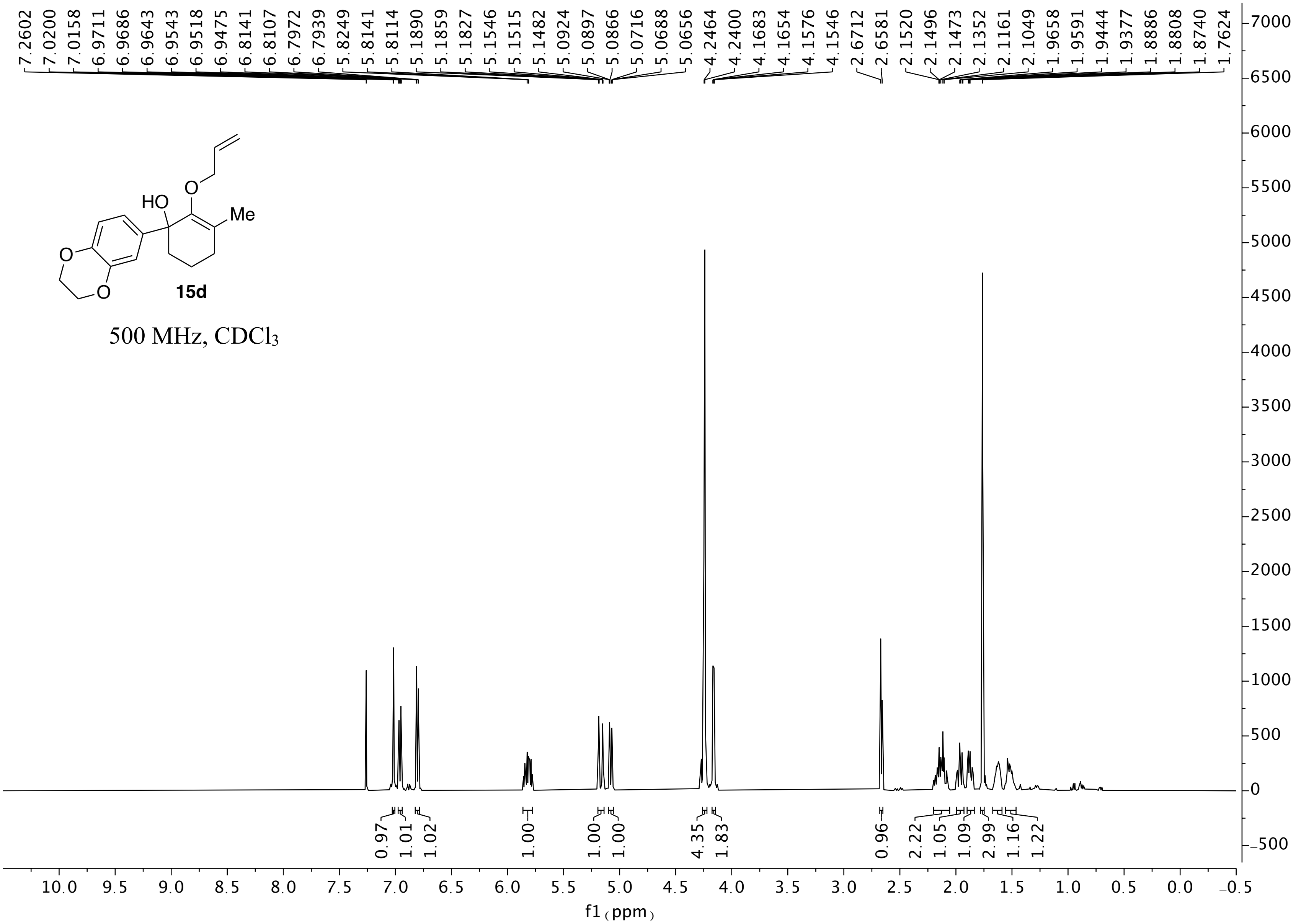
7.2602
7.0200
7.0158
6.9711
6.9686
6.9643
6.9543
6.9518
6.9475
6.8141
6.8107
6.7972
6.7939
5.8249
5.8141
5.8114
5.1890
5.1859
5.1827
5.1546
5.1515
5.1482
5.0924
5.0897
5.0866
5.0716
5.0688
5.0656
4.2464
4.2400
4.1683
4.1654
4.1576
4.1546
2.6712
2.6581
2.1520
2.1496
2.1473
2.1352
2.1161
2.1049
1.9658
1.9591
1.9444
1.9377
1.8886
1.8808
1.8740
1.7624
HO
O
Me
O
O
15d
500 MHz, CDCl3
0.97
1.01
1.02
1.00
1.00
1.00
4.35
1.83
0.96
2.22
1.05
1.09
2.99
1.16
1.22
f1 (ppm)

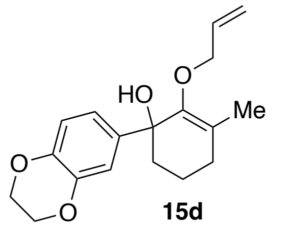

15d

125 MHz, $CDCl_3$

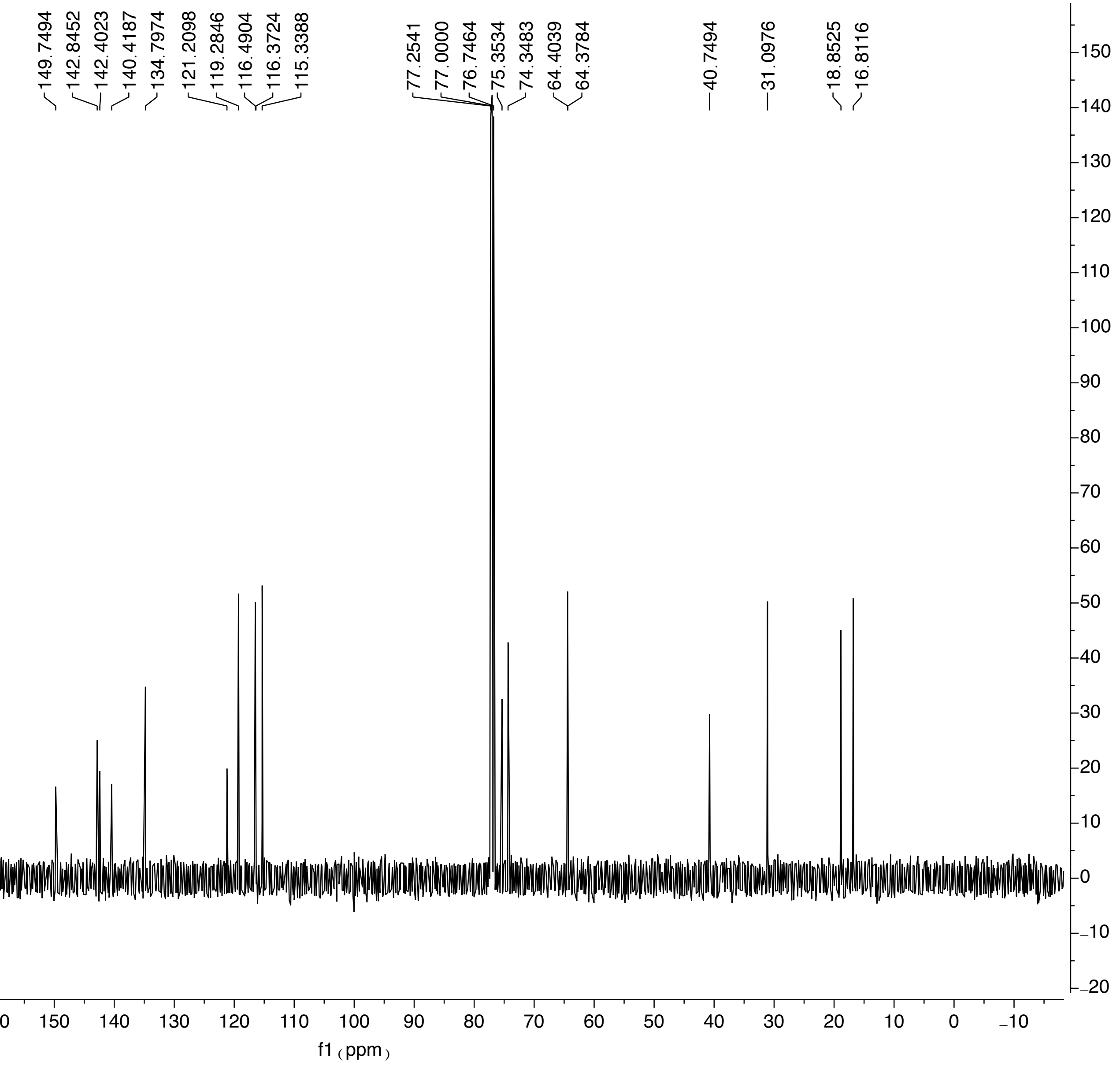

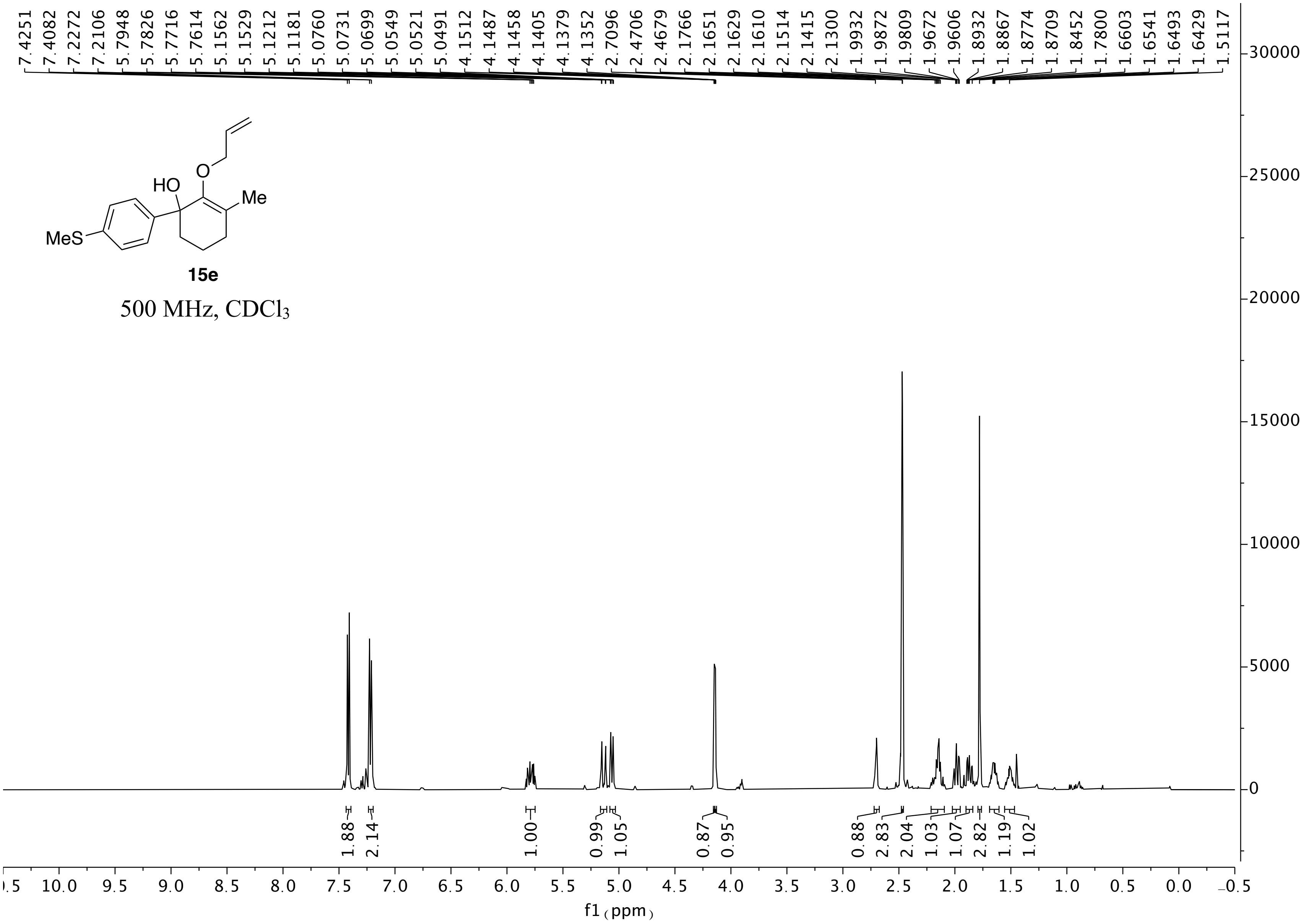
7.4251
7.4082
7.2272
7.2106
5.7948
5.7826
5.7716
5.7614
5.1562
5.1529
5.1212
5.1181
5.0760
5.0731
5.0699
5.0549
5.0521
5.0491
4.1512
4.1487
4.1458
4.1405
4.1379
4.1352
2.7096
2.4706
2.4679
2.1766
2.1651
2.1629
2.1610
2.1514
2.1415
2.1300
1.9932
1.9872
1.9809
1.9672
1.9606
1.8932
1.8867
1.8774
1.8709
1.8452
1.7800
1.6603
1.6541
1.6493
1.6429
1.5117
MeS
HO
O
Me
15e
500 MHz, CDCl3
1.88
2.14
1.00
0.99
1.05
0.87
0.95
0.88
2.83
2.04
1.03
1.07
2.82
1.19
1.02
f1 (ppm)

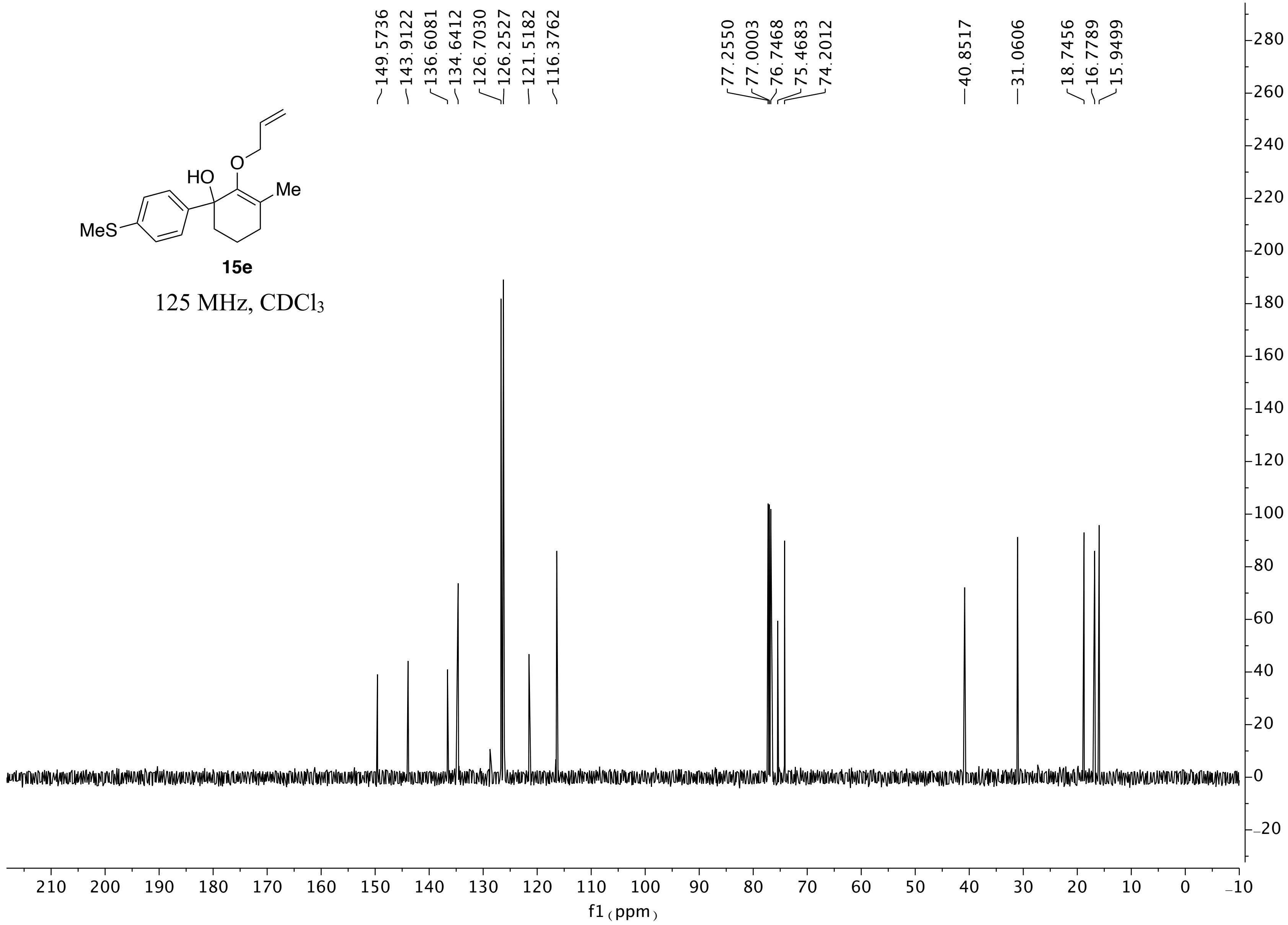

149.5736
143.9122
136.6081
134.6412
126.7030
126.2527
121.5182
116.3762
77.2550
77.0003
76.7468
75.4683
74.2012
40.8517
31.0606
18.7456
16.7789
15.9499
HO
O
Me
MeS
15e
125 MHz, CDCl3
f1 (ppm)

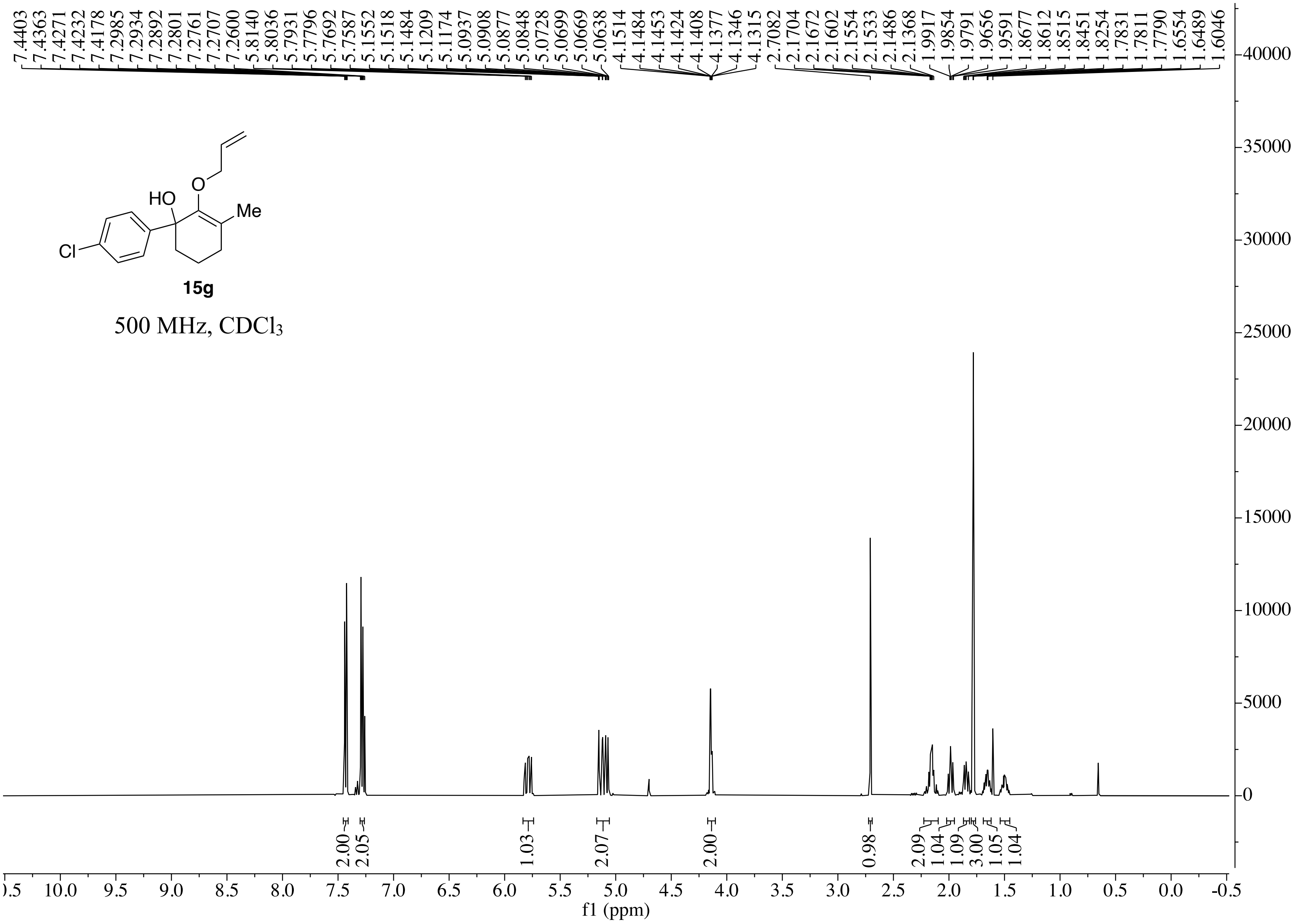
7.4403
7.4363
7.4271
7.4232
7.4178
7.2985
7.2934
7.2892
7.2801
7.2761
7.2707
7.2600
5.8140
5.8036
5.7931
5.7796
5.7692
5.7587
5.1552
5.1518
5.1484
5.1209
5.1174
5.0937
5.0908
5.0877
5.0848
5.0728
5.0699
5.0669
5.0638
4.1514
4.1484
4.1453
4.1424
4.1408
4.1377
4.1346
4.1315
2.7082
2.1704
2.1672
2.1602
2.1554
2.1533
2.1486
2.1368
1.9917
1.9854
1.9791
1.9656
1.9591
1.8677
1.8612
1.8515
1.8451
1.8254
1.7831
1.7811
1.7790
1.6554
1.6489
1.6046
HO
O
Me
Cl
15g
500 MHz, CDCl3
2.00
2.05
1.03
2.07
2.00
0.98
2.09
1.04
1.09
3.00
1.05
1.04
f1 (ppm)
10.0 9.5 9.0 8.5 8.0 7.5 7.0 6.5 6.0 5.5 5.0 4.5 4.0 3.5 3.0 2.5 2.0 1.5 1.0 0.5 0.0 -0.5
40000 35000 30000 25000 20000 15000 10000 5000 0

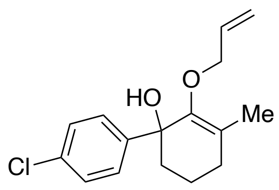

15g

125 MHz, $CDCl_3$

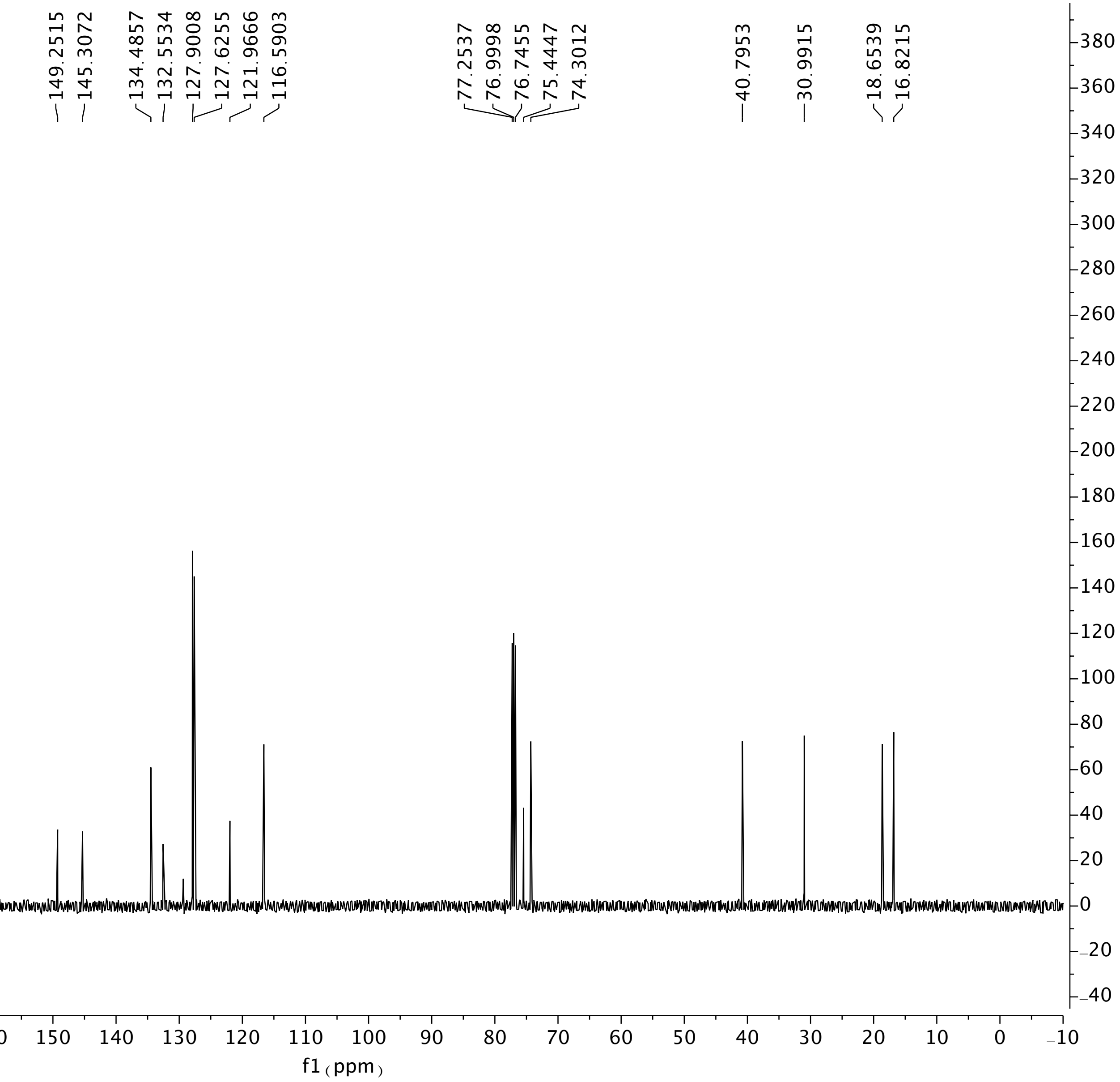

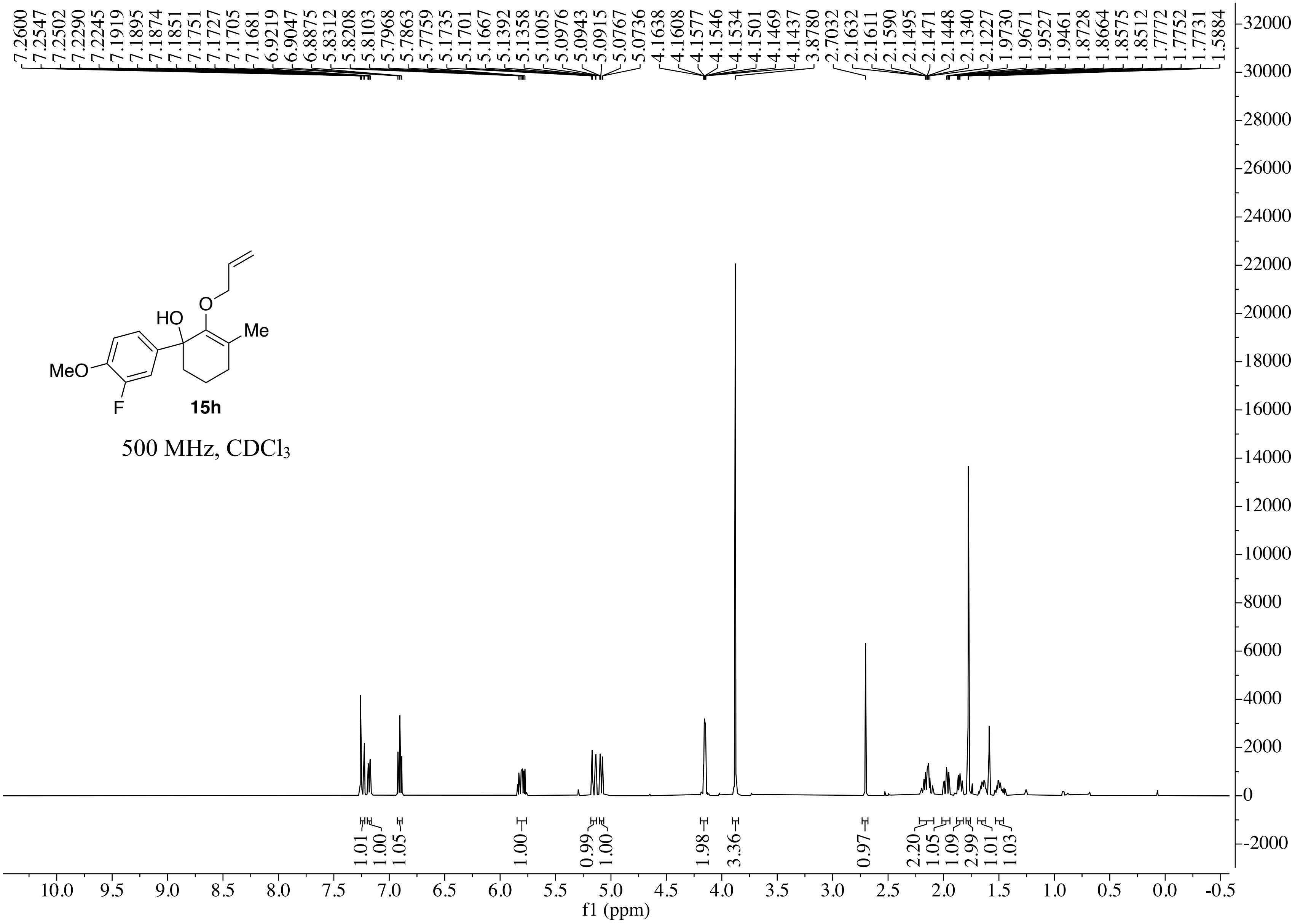

7.2600
7.2547
7.2502
7.2290
7.2245
7.1919
7.1895
7.1874
7.1851
7.1751
7.1727
7.1705
7.1681
6.9219
6.9047
6.8875
5.8312
5.8208
5.8103
5.7968
5.7863
5.7759
5.1735
5.1701
5.1667
5.1392
5.1358
5.1005
5.0976
5.0943
5.0915
5.0767
5.0736
4.1638
4.1608
4.1577
4.1546
4.1534
4.1501
4.1469
4.1437
3.8780
2.7032
2.1632
2.1611
2.1590
2.1495
2.1471
2.1448
2.1340
2.1227
1.9730
1.9671
1.9527
1.9461
1.8728
1.8664
1.8575
1.8512
1.7772
1.7752
1.7731
1.5884
HO
O
Me
MeO
F
15h
500 MHz, CDCl3
1.01
1.00
1.05
1.00
0.99
1.00
1.98
3.36
0.97
2.20
1.05
1.09
2.99
1.01
1.03
10.0
9.5
9.0
8.5
8.0
7.5
7.0
6.5
6.0
5.5
5.0
4.5
4.0
3.5
3.0
2.5
2.0
1.5
1.0
0.5
0.0
-0.5
f1 (ppm)
32000
30000
28000
26000
24000
22000
20000
18000
16000
14000
12000
10000
8000
6000
4000
2000
0
-2000

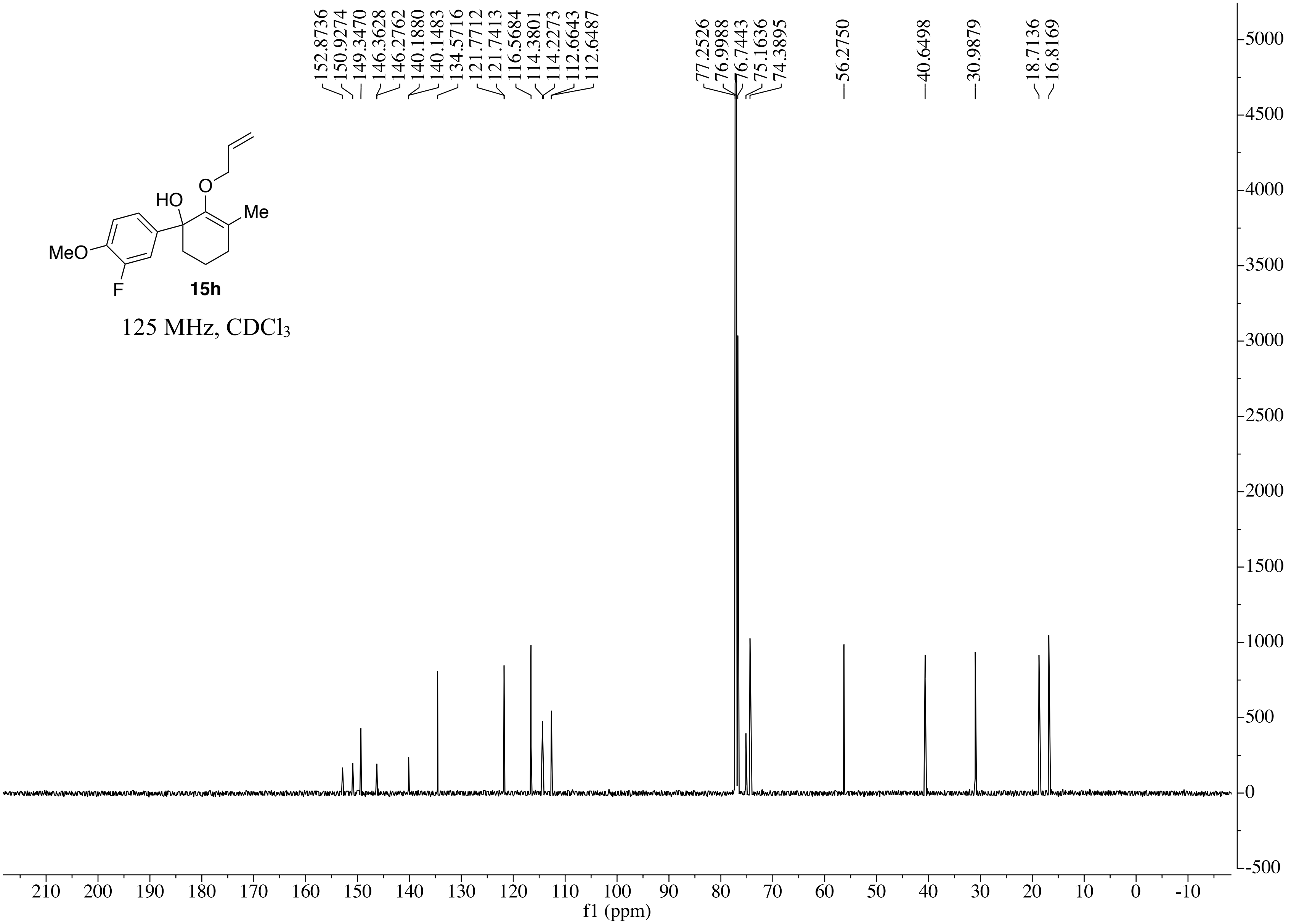

HO
O
Me
MeO
F
15h
125 MHz, $CDCl_3$
152.8736
150.9274
149.3470
146.3628
146.2762
140.1880
140.1483
134.5716
121.7712
121.7413
116.5684
114.3801
114.2273
112.6643
112.6487
77.2526
76.9988
76.7443
75.1636
74.3895
56.2750
40.6498
30.9879
18.7136
16.8169
5000
4500
4000
3500
3000
2500
2000
1500
1000
500
0
-500
210
200
190
180
170
160
150
140
130
120
110
100
90
80
70
60
50
40
30
20
10
0
-10
f1 (ppm)

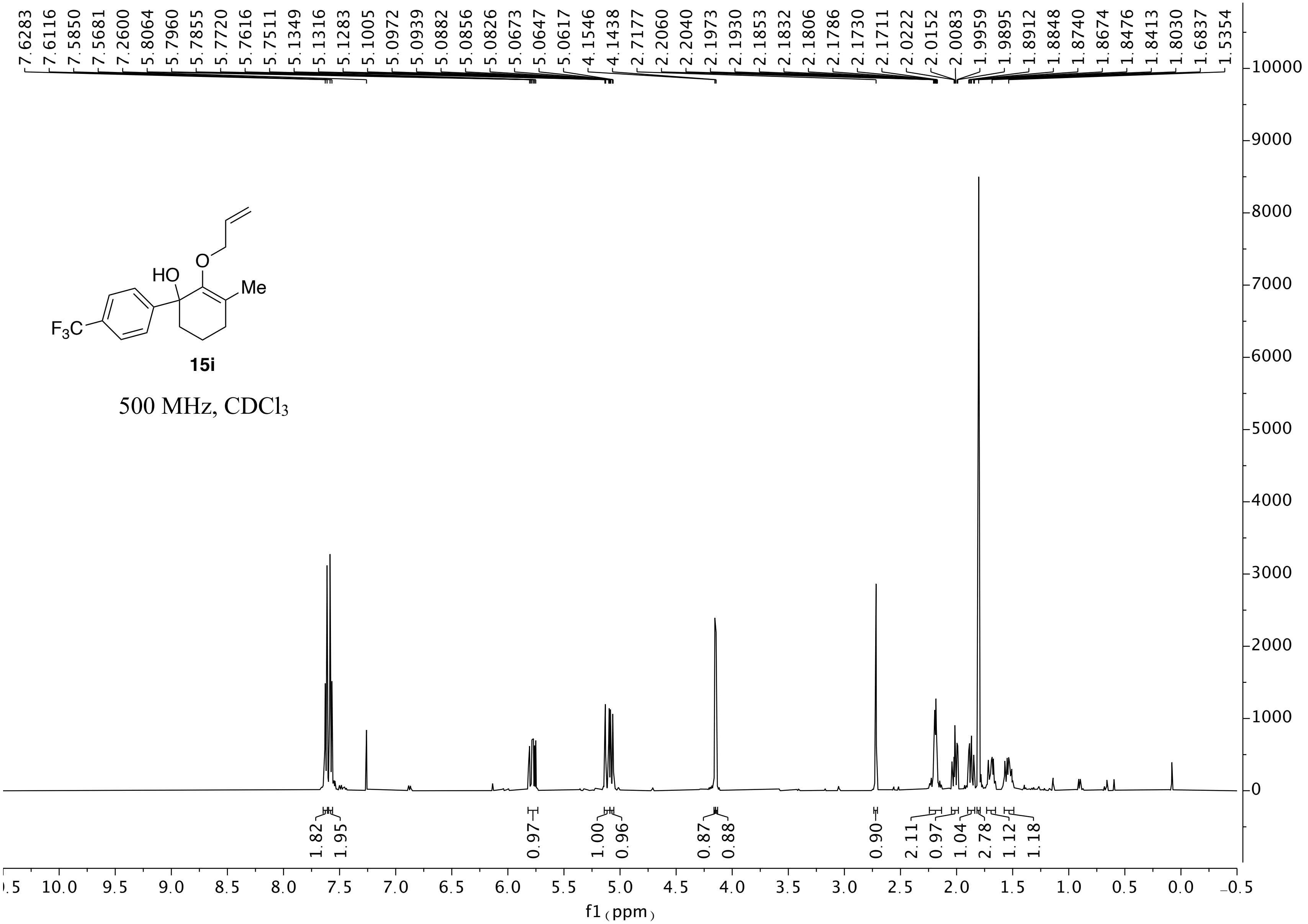

15i

500 MHz, $CDCl_3$

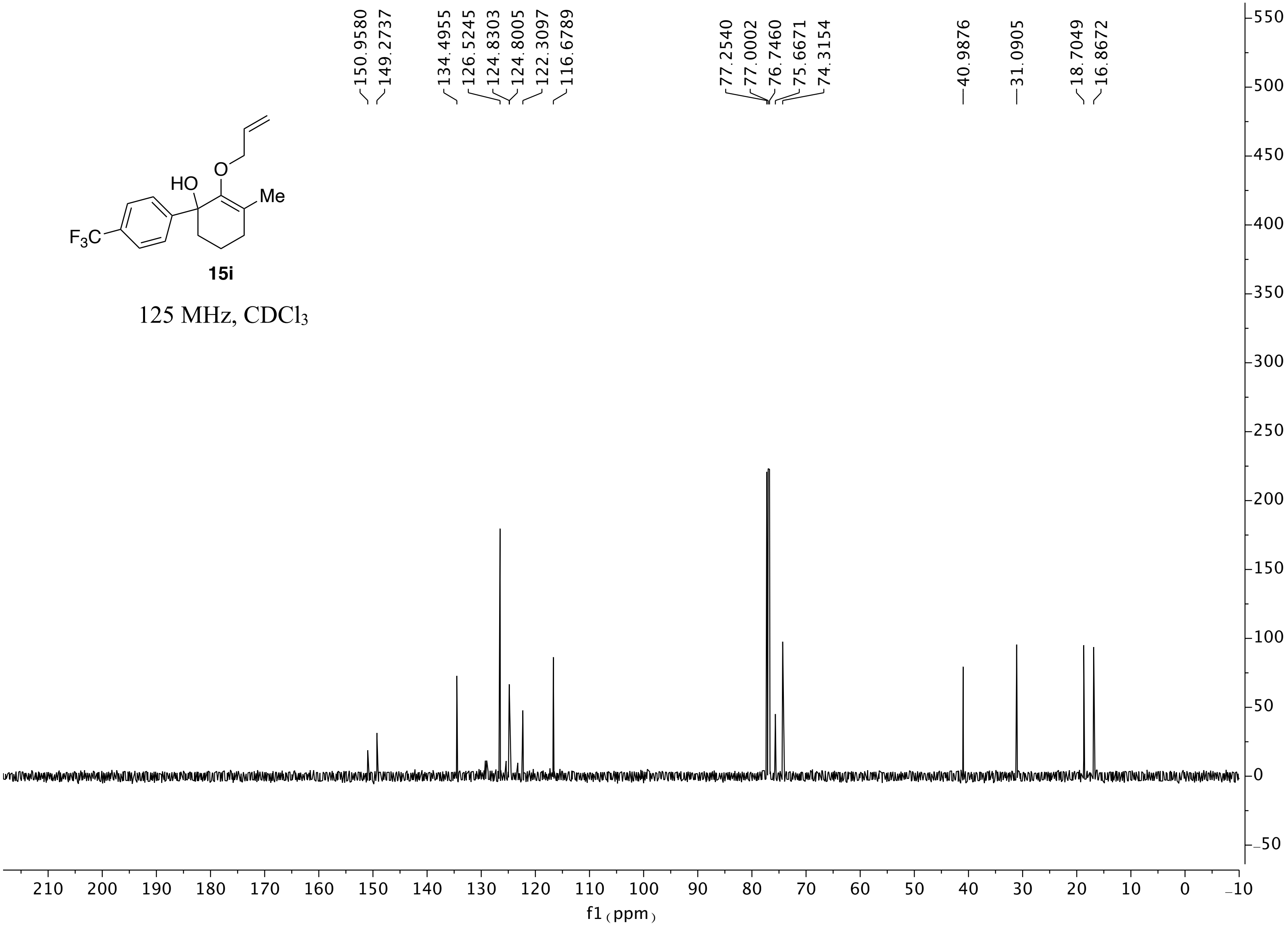
150.9580
149.2737
134.4955
126.5245
124.8303
124.8005
122.3097
116.6789
77.2540
77.0002
76.7460
75.6671
74.3154
40.9876
31.0905
18.7049
16.8672
HO
O
Me
F_3C
15i
125 MHz, $CDCl_3$
f1 (ppm)

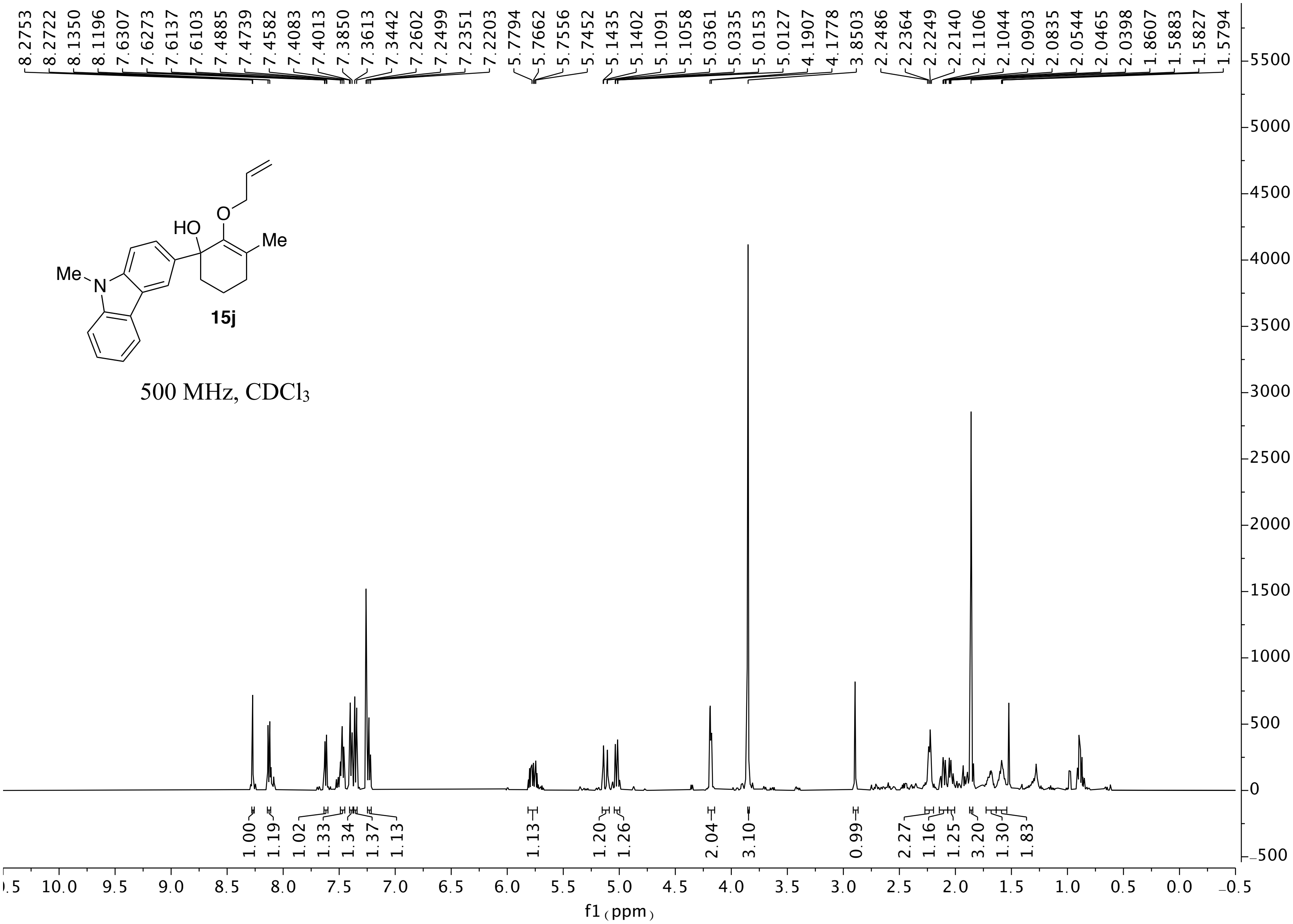
8.2753
8.2722
8.1350
8.1196
7.6307
7.6273
7.6137
7.6103
7.4885
7.4739
7.4582
7.4083
7.4013
7.3850
7.3613
7.3442
7.2602
7.2499
7.2351
7.2203
5.7794
5.7662
5.7556
5.7452
5.1435
5.1402
5.1091
5.1058
5.0361
5.0335
5.0153
5.0127
4.1907
4.1778
3.8503
2.2486
2.2364
2.2249
2.2140
2.1106
2.1044
2.0903
2.0835
2.0544
2.0465
2.0398
1.8607
1.5883
1.5827
1.5794
HO
O
Me
Me
N
15j
500 MHz, CDCl3
1.00
1.19
1.02
1.33
1.34
1.37
1.13
1.13
1.20
1.26
2.04
3.10
0.99
2.27
1.16
1.25
3.20
1.30
1.83
f1 (ppm)

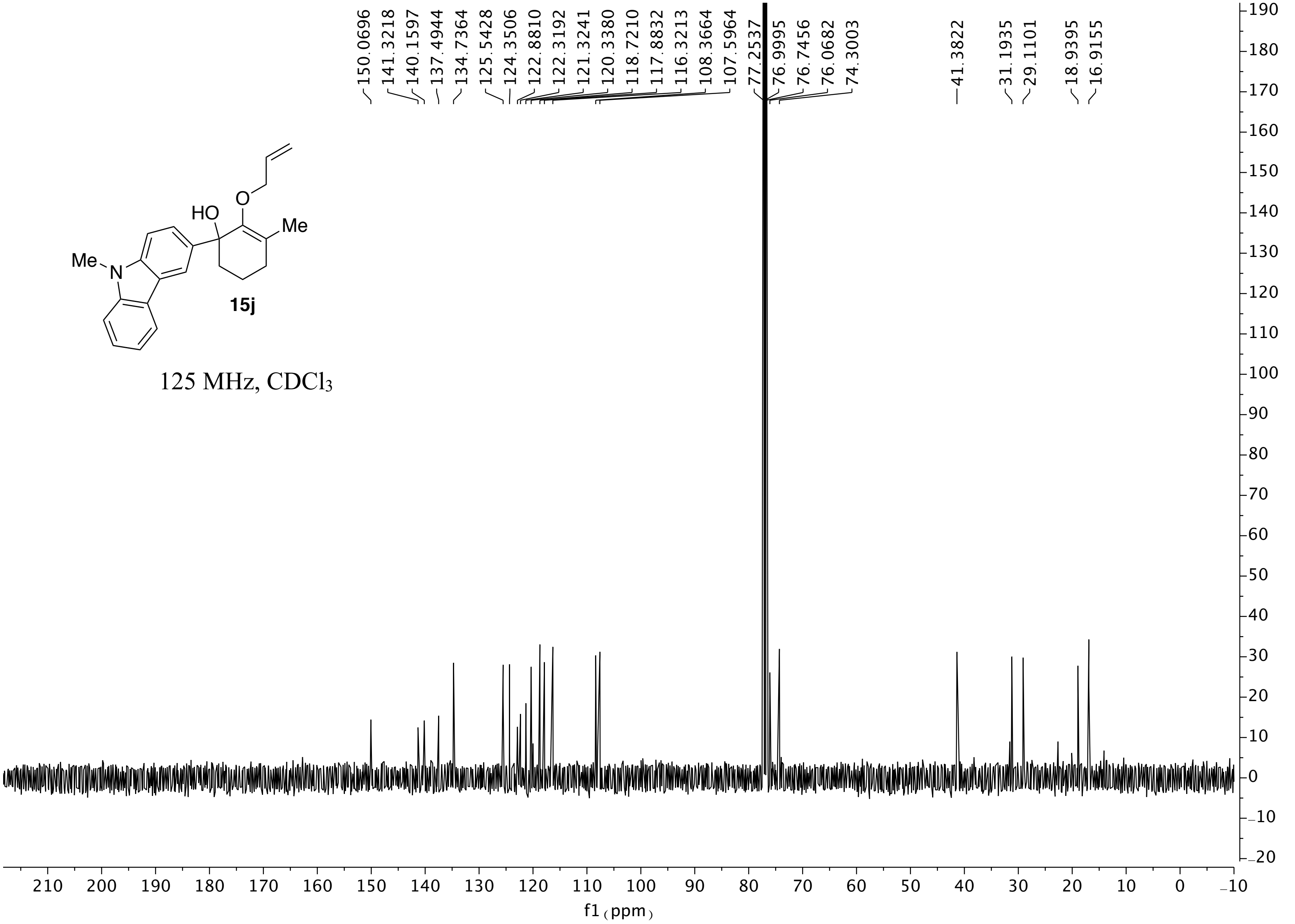
HO
O
Me
Me
N
15j
125 MHz, CDCl3
150.0696
141.3218
140.1597
137.4944
134.7364
125.5428
124.3506
122.8810
122.3192
121.3241
120.3380
118.7210
117.8832
116.3213
108.3664
107.5964
77.2537
76.9995
76.7456
76.0682
74.3003
41.3822
31.1935
29.1101
18.9395
16.9155
f1 (ppm)

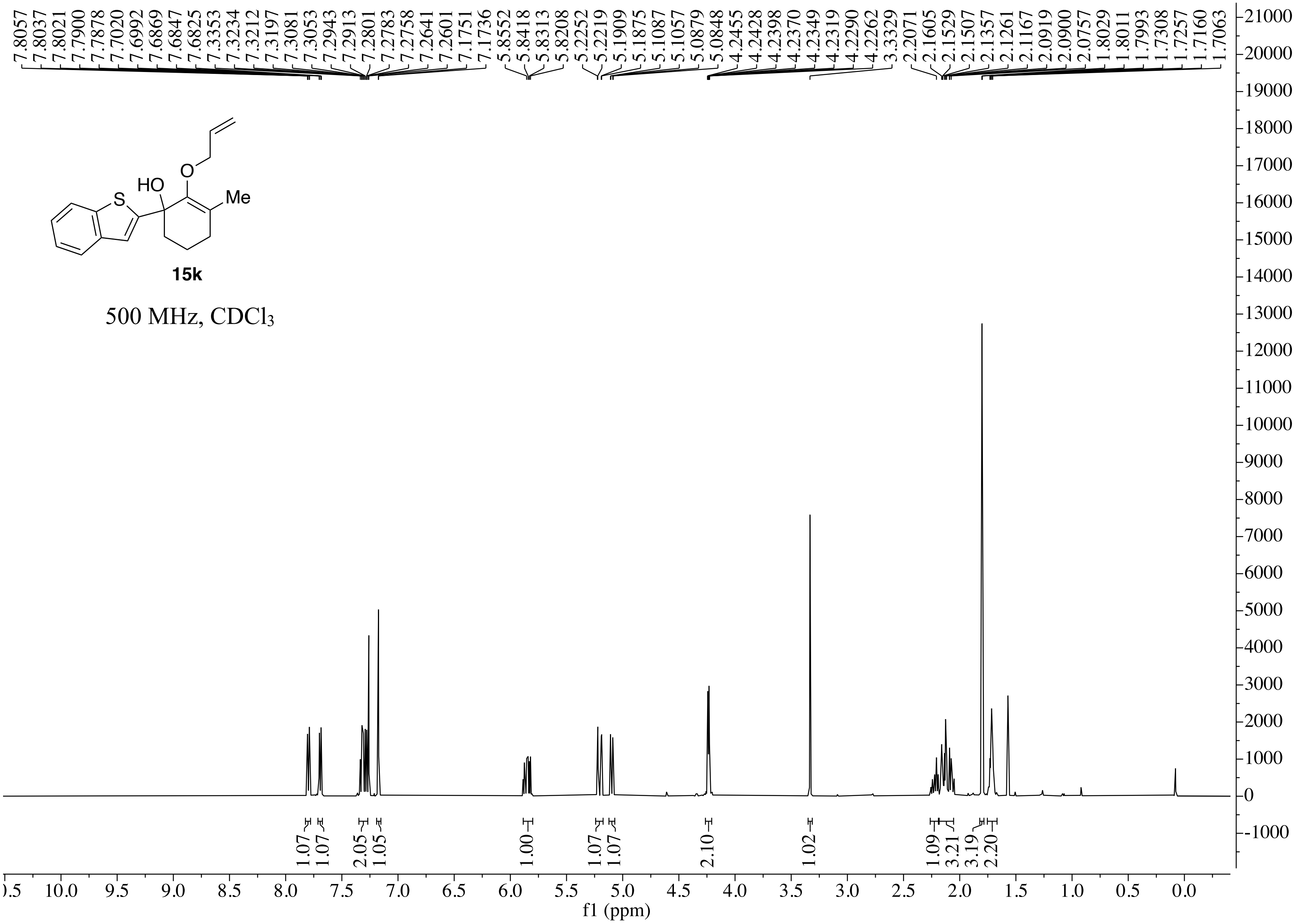
7.8057
7.8037
7.8021
7.7900
7.7878
7.7020
7.6992
7.6869
7.6847
7.6825
7.3353
7.3234
7.3212
7.3197
7.3081
7.3053
7.2943
7.2913
7.2801
7.2783
7.2758
7.2641
7.2601
7.1751
7.1736
5.8552
5.8418
5.8313
5.8208
5.2252
5.2219
5.1909
5.1875
5.1087
5.1057
5.0879
5.0848
4.2455
4.2428
4.2398
4.2370
4.2349
4.2319
4.2290
4.2262
3.3329
2.2071
2.1605
2.1529
2.1507
2.1357
2.1261
2.1167
2.0919
2.0900
2.0757
1.8029
1.8011
1.7993
1.7308
1.7257
1.7160
1.7063
HO
O
S
Me
15k
500 MHz, $CDCl_3$
1.07
1.07
2.05
1.05
1.00
1.07
1.07
2.10
1.02
1.09
3.21
3.19
2.20
f1 (ppm)

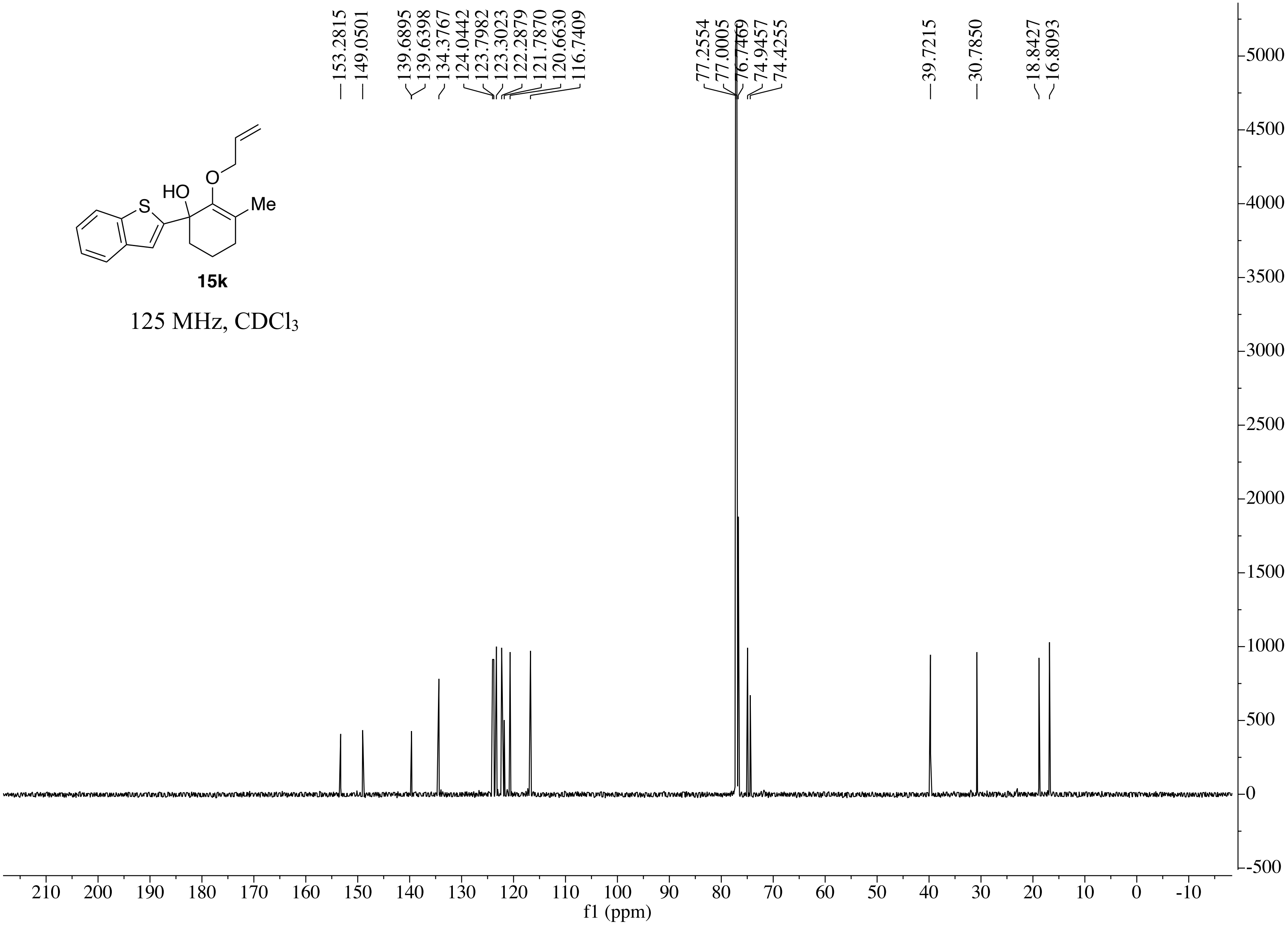
153.2815
149.0501
139.6895
139.6398
134.3767
124.0442
123.7982
123.3023
122.2879
121.7870
120.6630
116.7409
77.2554
77.0005
76.7469
74.9457
74.4255
39.7215
30.7850
18.8427
16.8093
HO
O
S
Me
15k
125 MHz, $CDCl_3$
f1 (ppm)

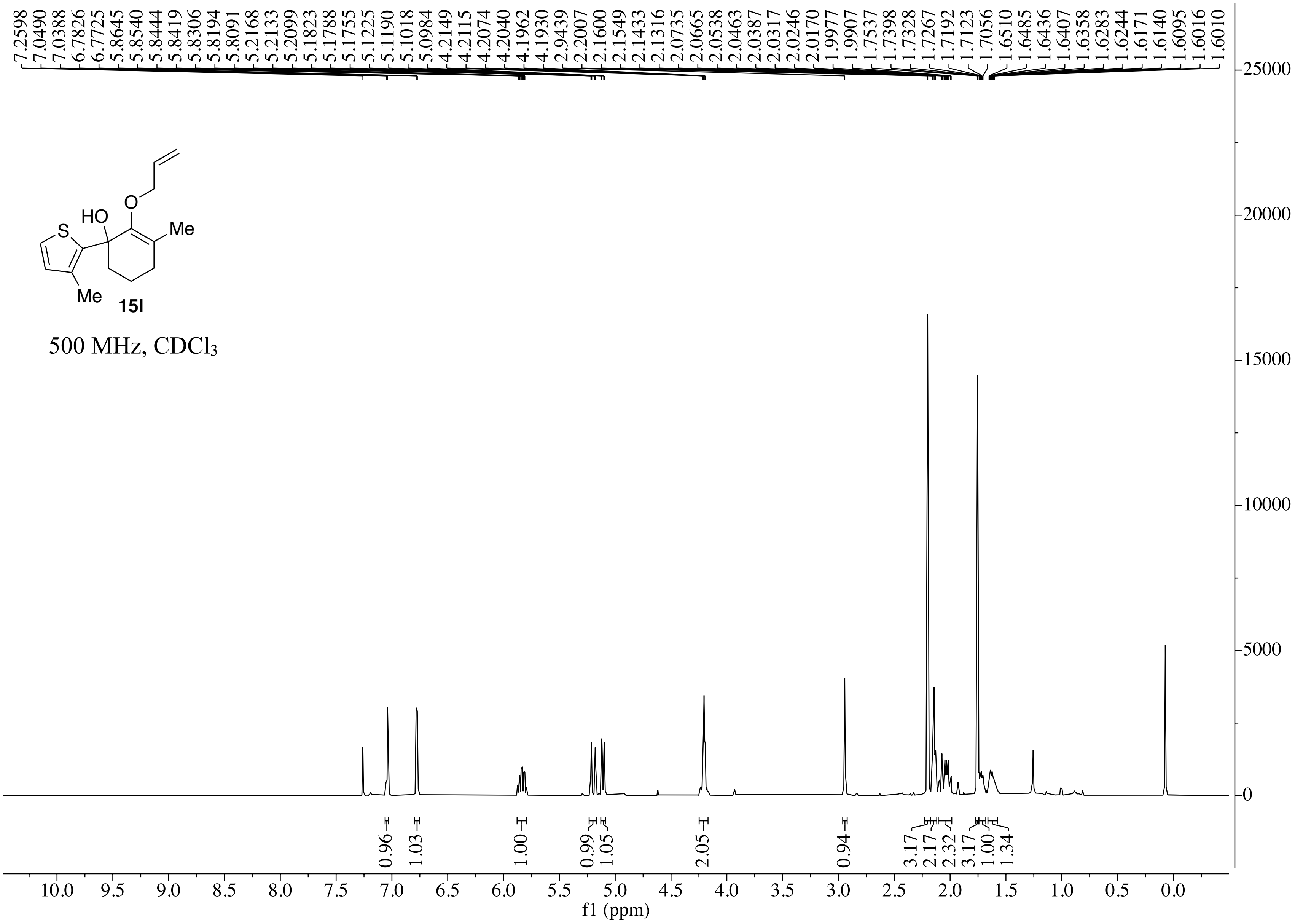
7.2598
7.0490
7.0388
6.7826
6.7725
5.8645
5.8540
5.8444
5.8419
5.8306
5.8194
5.8091
5.2168
5.2133
5.2099
5.1823
5.1788
5.1755
5.1225
5.1190
5.1018
5.0984
4.2149
4.2115
4.2074
4.2040
4.1962
4.1930
2.9439
2.2007
2.1600
2.1549
2.1433
2.1316
2.0735
2.0665
2.0538
2.0463
2.0387
2.0317
2.0246
2.0170
1.9977
1.9907
1.7537
1.7398
1.7328
1.7267
1.7192
1.7123
1.7056
1.6510
1.6485
1.6436
1.6407
1.6358
1.6283
1.6244
1.6171
1.6140
1.6095
1.6016
1.6010
HO
O
S
Me
Me
15l
500 MHz, CDCl3
0.96
1.03
1.00
0.99
1.05
2.05
0.94
3.17
2.17
2.32
3.17
1.00
1.34
f1 (ppm)

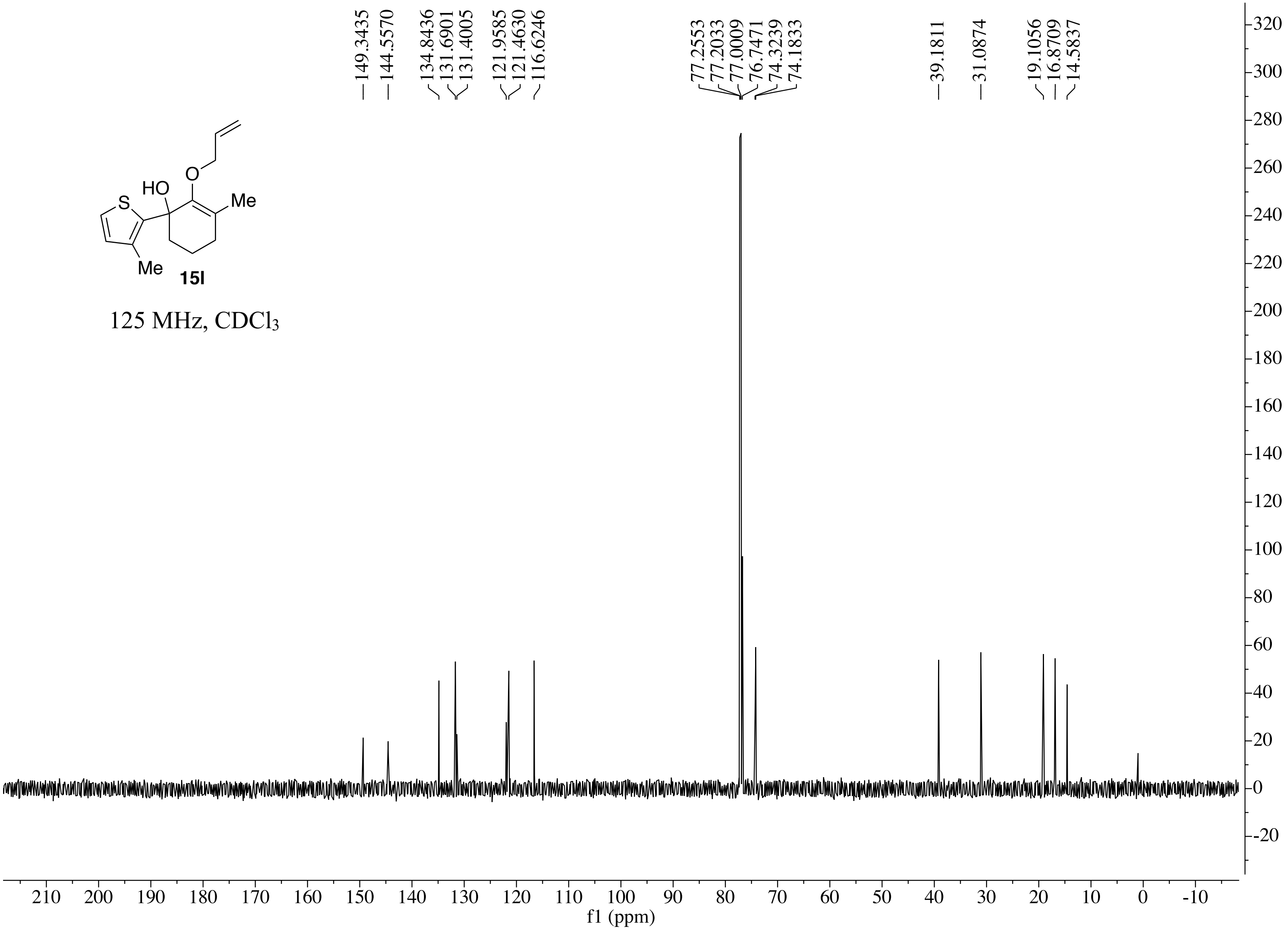
149.3435
144.5570
134.8436
131.6901
131.4005
121.9585
121.4630
116.6246
77.2553
77.2033
77.0009
76.7471
74.3239
74.1833
39.1811
31.0874
19.1056
16.8709
14.5837
HO
O
S
Me
Me
15l
125 MHz, CDCl3
f1 (ppm)

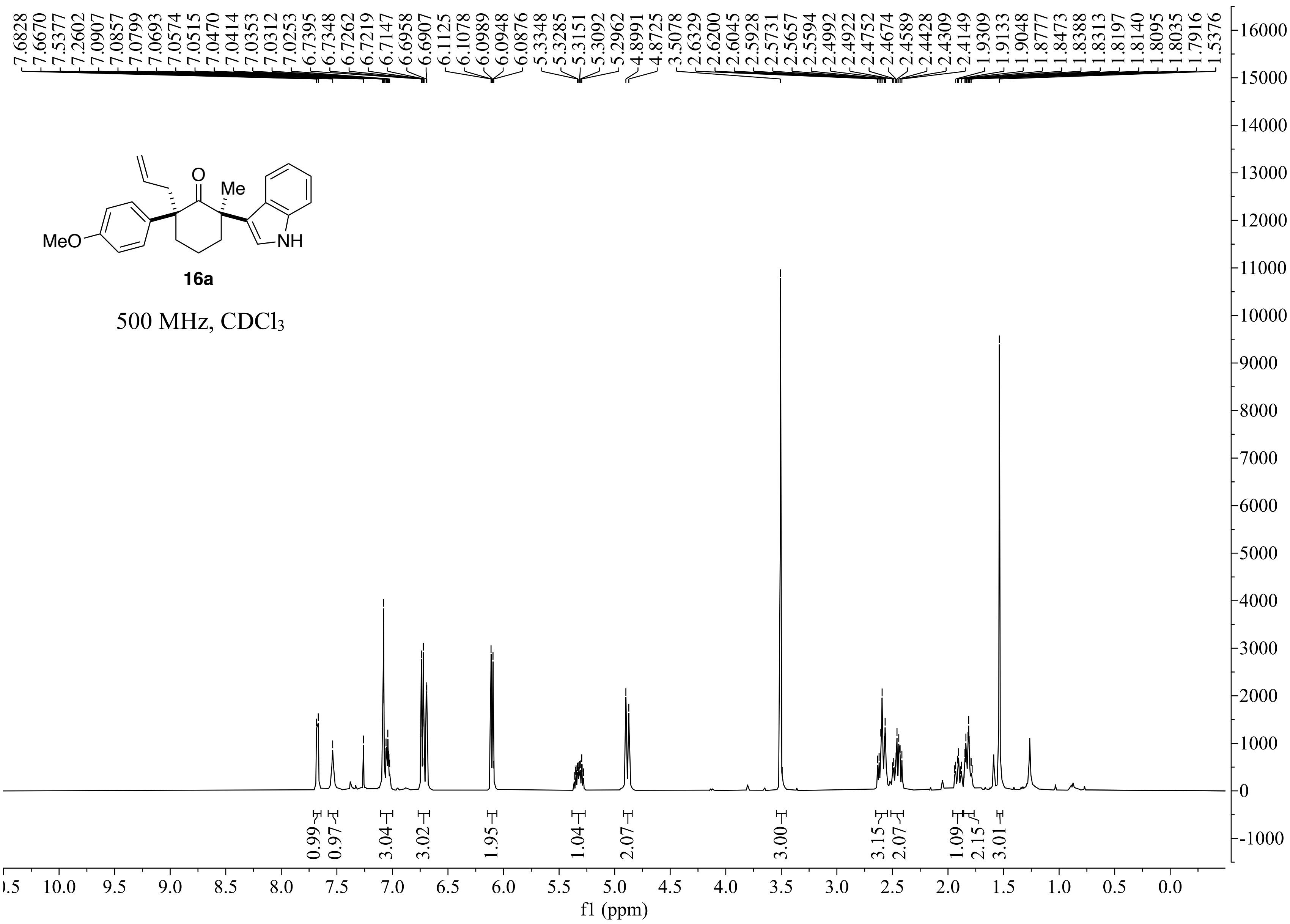
7.6828 7.6670 7.5377 7.2602 7.0907 7.0857 7.0799 7.0693 7.0574 7.0515 7.0470 7.0414 7.0353 7.0312 7.0253 6.7395 6.7348 6.7262 6.7219 6.7147 6.6958 6.6907 6.1125 6.1078 6.0989 6.0948 6.0876 5.3348 5.3285 5.3151 5.3092 5.2962 4.8991 4.8725 3.5078 2.6329 2.6200 2.6045 2.5928 2.5731 2.5657 2.5594 2.4992 2.4922 2.4752 2.4674 2.4589 2.4428 2.4309 2.4149 1.9309 1.9133 1.9048 1.8777 1.8473 1.8388 1.8313 1.8197 1.8140 1.8095 1.8035 1.7916 1.5376
O
Me
MeO
NH
16a
500 MHz, CDCl3
0.99 0.97 3.04 3.02 1.95 1.04 2.07 3.00 3.15 2.07 1.09 2.15 3.01
f1 (ppm)

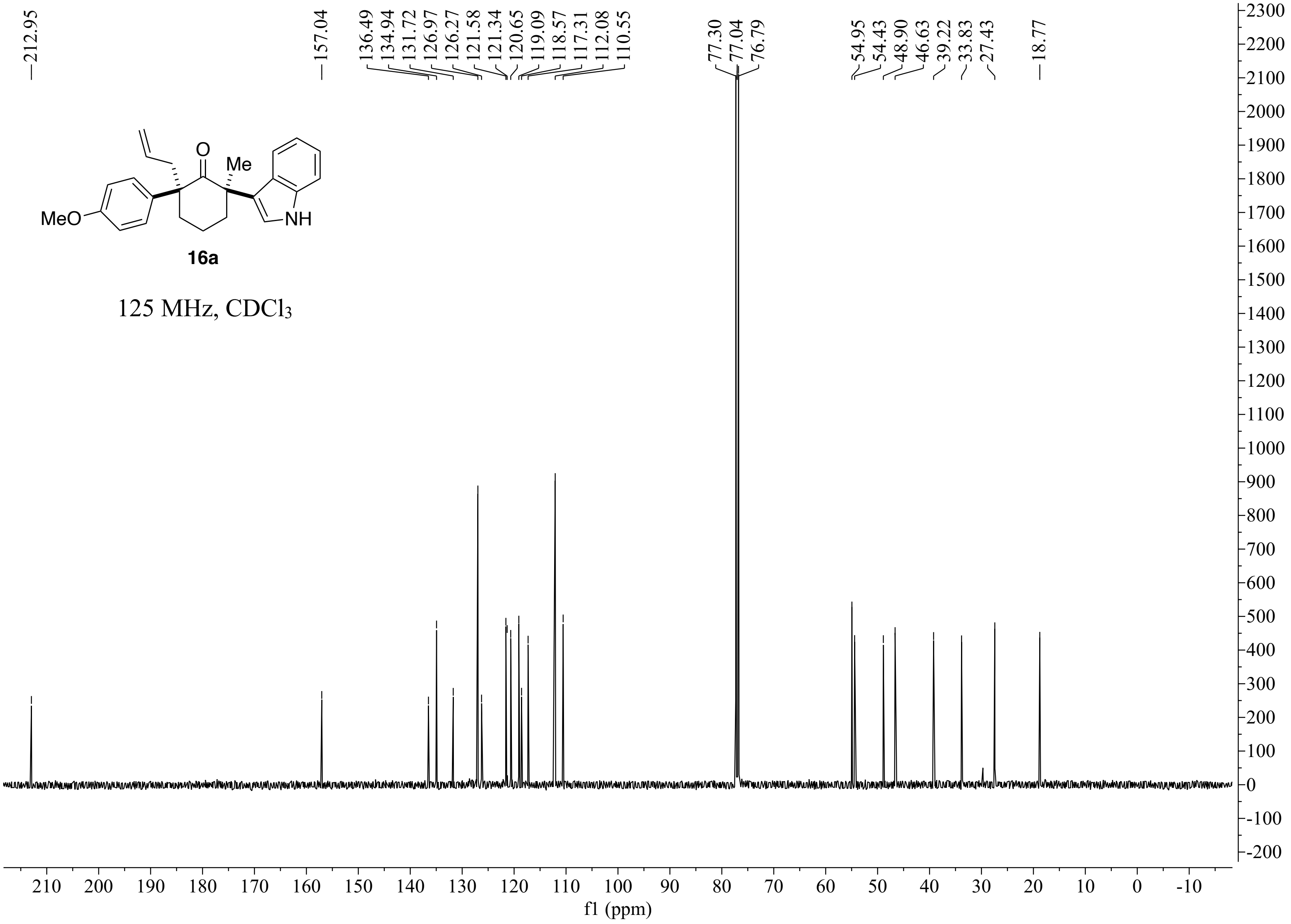

212.95
157.04
136.49
134.94
131.72
126.97
126.27
121.58
121.34
120.65
119.09
118.57
117.31
112.08
110.55
77.30
77.04
76.79
54.95
54.43
48.90
46.63
39.22
33.83
27.43
18.77
O
Me
MeO
NH
16a
125 MHz, CDCl3
f1 (ppm)

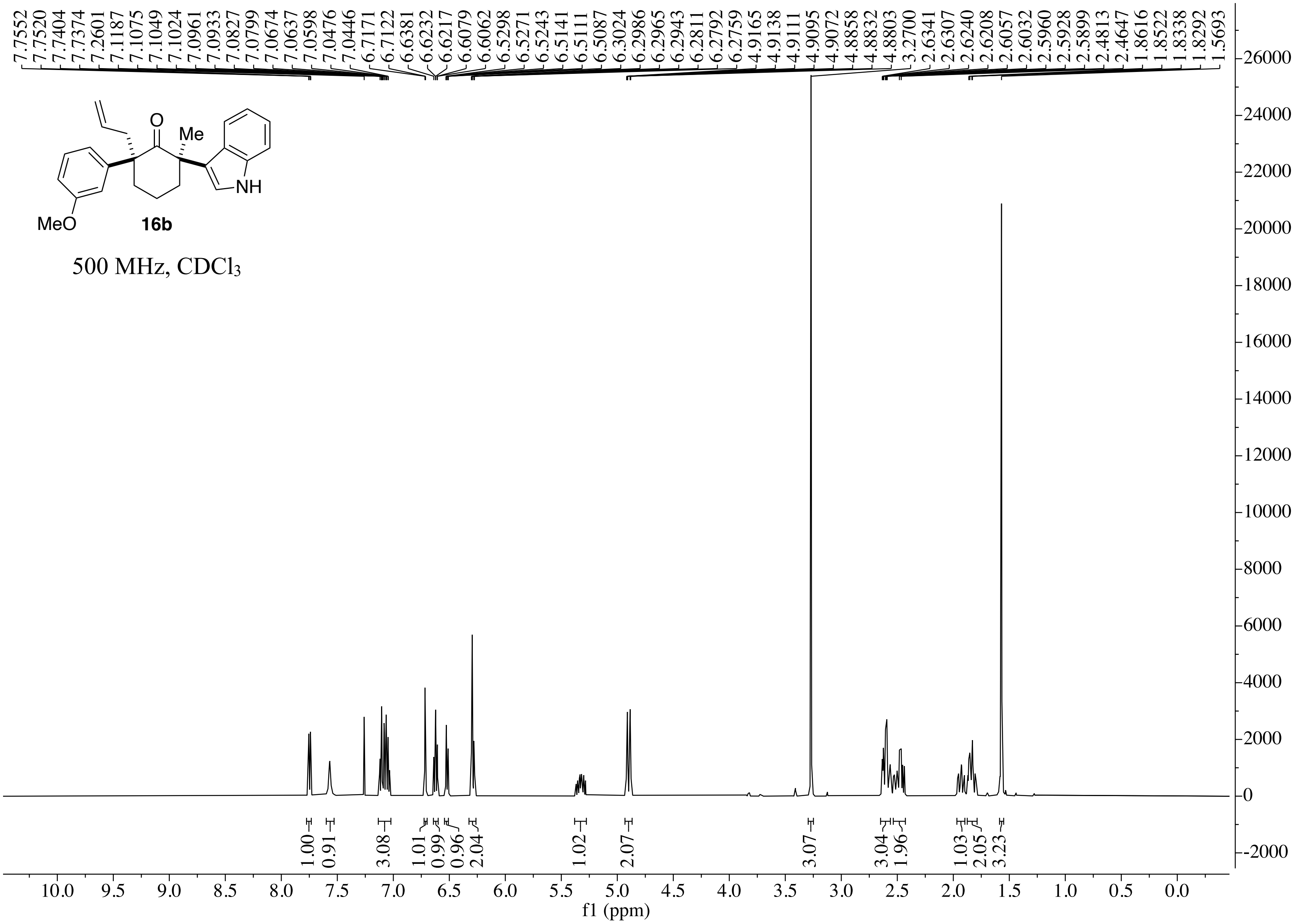
Me
O
NH
MeO
16b
500 MHz, CDCl3
7.7552
7.7520
7.7404
7.7374
7.2601
7.1187
7.1075
7.1049
7.1024
7.0961
7.0933
7.0827
7.0799
7.0674
7.0637
7.0598
7.0476
7.0446
6.7171
6.7122
6.6381
6.6232
6.6217
6.6079
6.6062
6.5298
6.5271
6.5243
6.5141
6.5111
6.5087
6.3024
6.2986
6.2965
6.2943
6.2811
6.2792
6.2759
4.9165
4.9138
4.9111
4.9095
4.9072
4.8858
4.8832
4.8803
3.2700
2.6341
2.6307
2.6240
2.6208
2.6057
2.6032
2.5960
2.5928
2.5899
2.4813
2.4647
1.8616
1.8522
1.8338
1.8292
1.5693
1.00
0.91
3.08
1.01
0.99
0.96
2.04
1.02
2.07
3.07
3.04
1.96
1.03
2.05
3.23
10.0 9.5 9.0 8.5 8.0 7.5 7.0 6.5 6.0 5.5 5.0 4.5 4.0 3.5 3.0 2.5 2.0 1.5 1.0 0.5 0.0
f1 (ppm)
26000 24000 22000 20000 18000 16000 14000 12000 10000 8000 6000 4000 2000 0 -2000

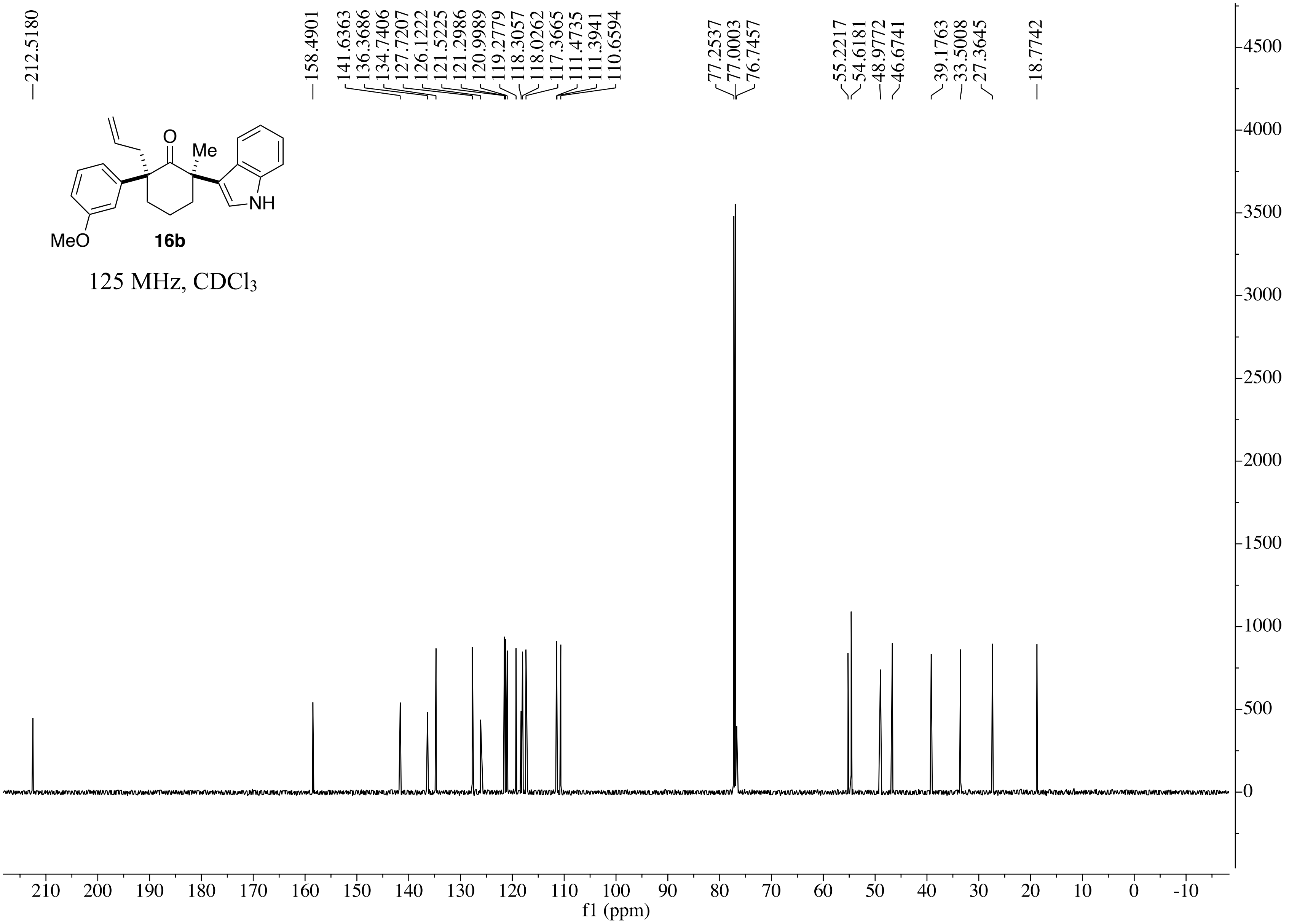
212.5180
158.4901
141.6363
136.3686
134.7406
127.7207
126.1222
121.5225
121.2986
120.9989
119.2779
118.3057
118.0262
117.3665
111.4735
111.3941
110.6594
77.2537
77.0003
76.7457
55.2217
54.6181
48.9772
46.6741
39.1763
33.5008
27.3645
18.7742
O
Me
NH
MeO
16b
125 MHz, CDCl3
f1 (ppm)

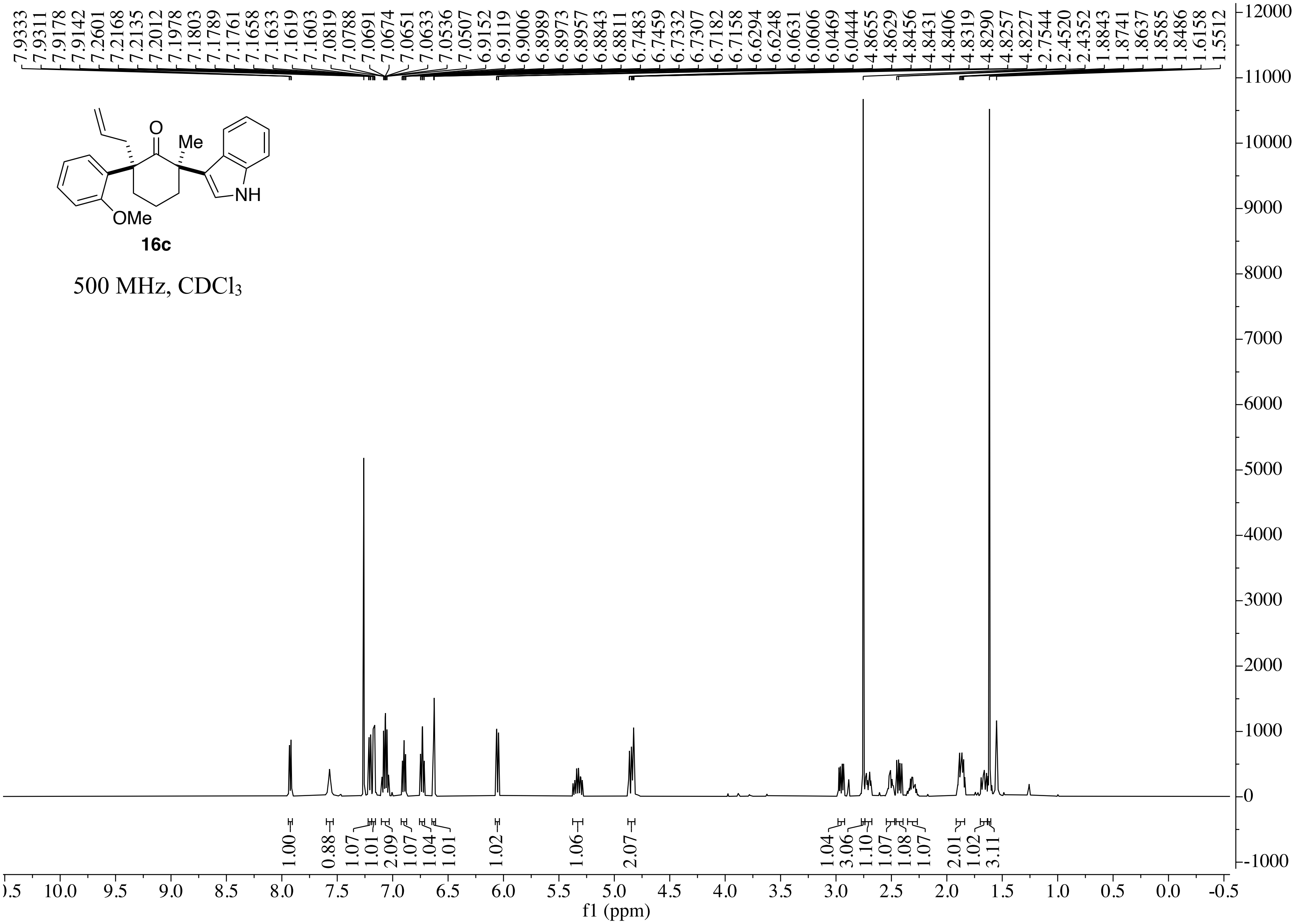
7.9333
7.9311
7.9178
7.9142
7.2601
7.2168
7.2135
7.2012
7.1978
7.1803
7.1789
7.1761
7.1658
7.1633
7.1619
7.1603
7.0819
7.0788
7.0691
7.0674
7.0651
7.0633
7.0536
7.0507
6.9152
6.9119
6.9006
6.8989
6.8973
6.8957
6.8843
6.8811
6.7483
6.7459
6.7332
6.7307
6.7182
6.7158
6.6294
6.6248
6.0631
6.0606
6.0469
6.0444
4.8655
4.8629
4.8456
4.8431
4.8406
4.8319
4.8290
4.8257
4.8227
2.7544
2.4520
2.4352
1.8843
1.8741
1.8637
1.8585
1.8486
1.6158
1.5512
O
Me
NH
OMe
16c
500 MHz, $CDCl_3$
1.00
0.88
1.07
1.01
2.09
1.07
1.04
1.01
1.02
1.06
2.07
1.04
3.06
1.10
1.07
1.08
1.07
2.01
1.02
3.11
10.0
9.5
9.0
8.5
8.0
7.5
7.0
6.5
6.0
5.5
5.0
4.5
4.0
3.5
3.0
2.5
2.0
1.5
1.0
0.5
0.0
-0.5
f1 (ppm)
12000
11000
10000
9000
8000
7000
6000
5000
4000
3000
2000
1000
0
-1000

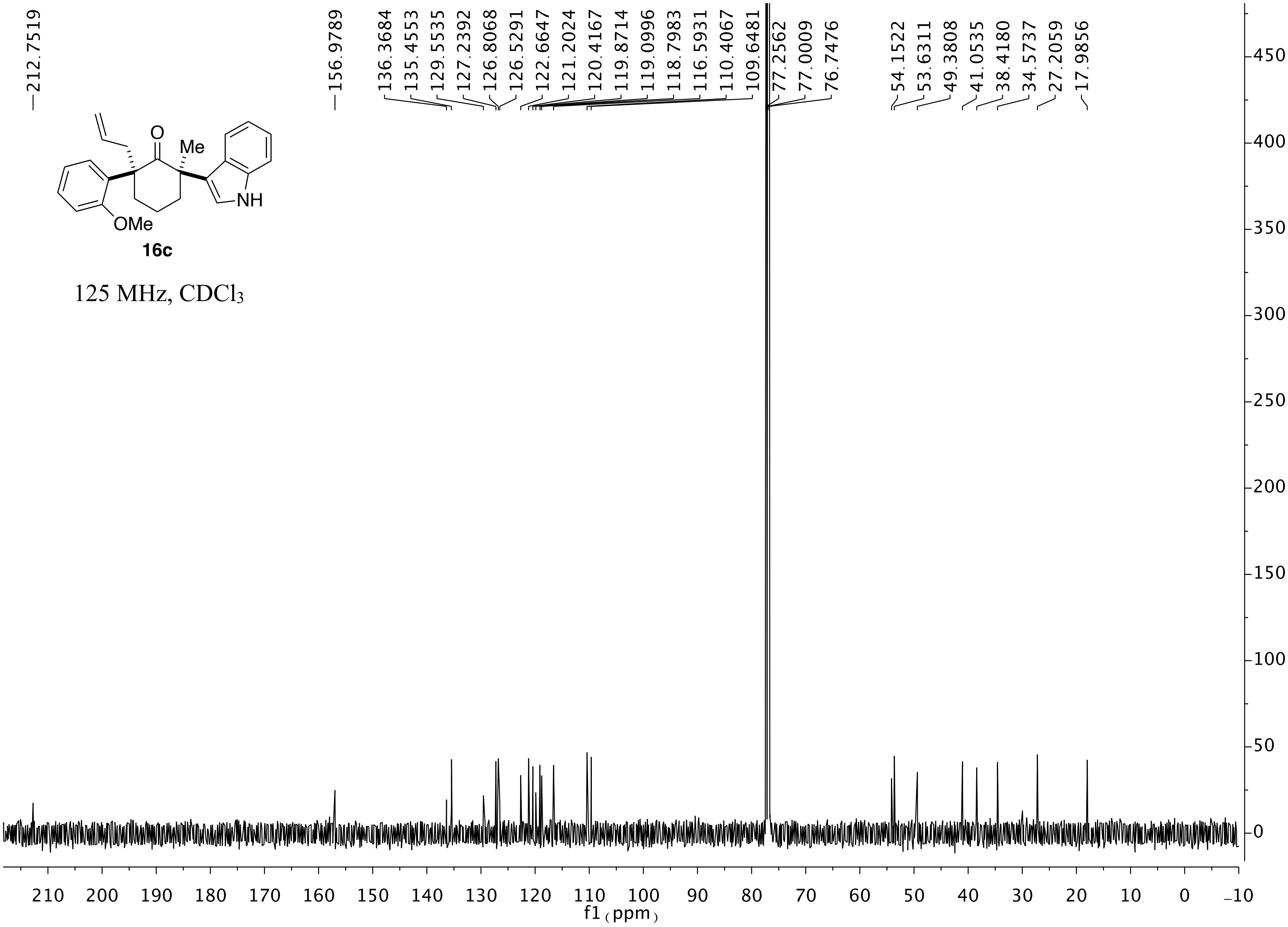

16c

125 MHz, $CDCl_3$

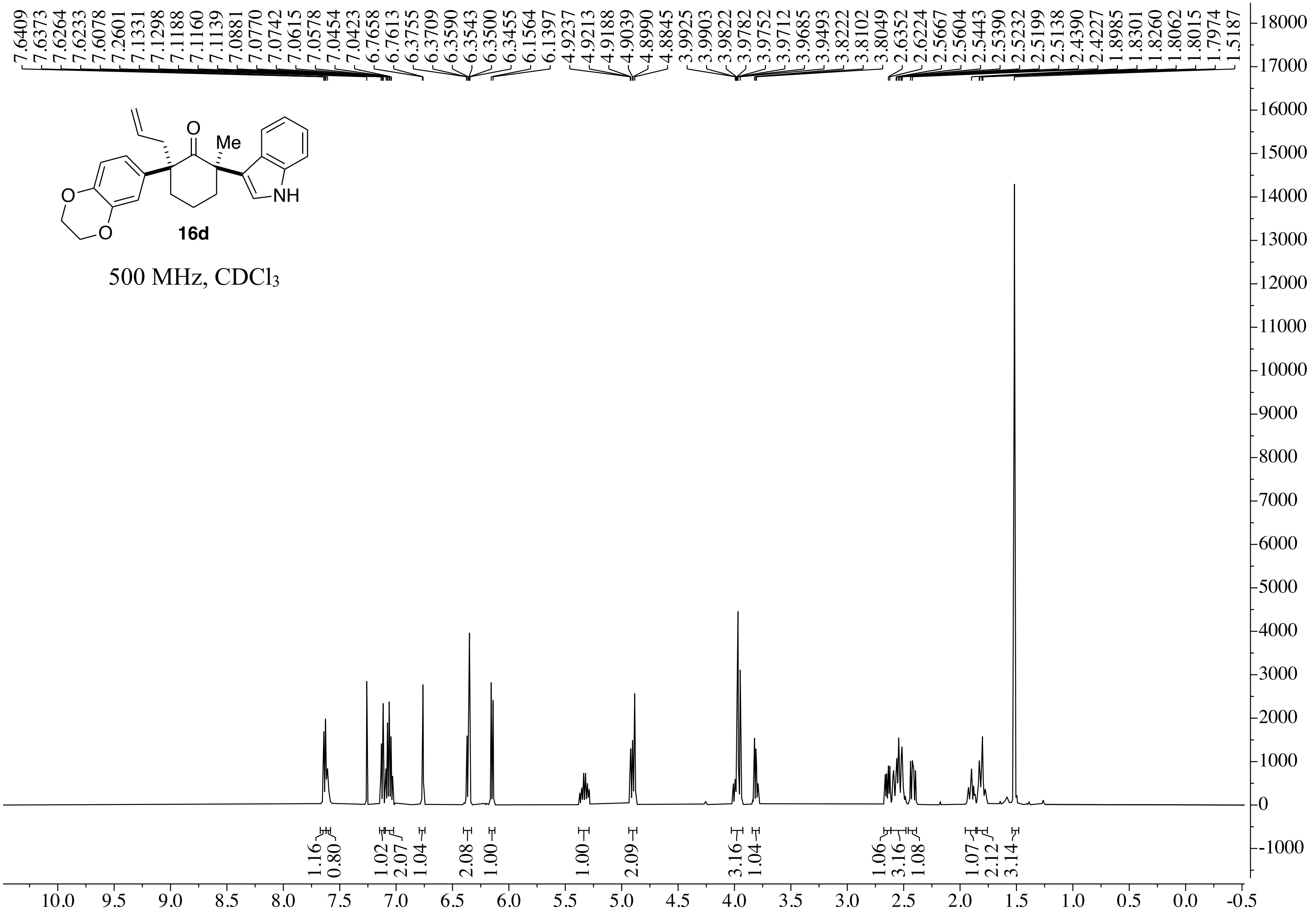
16d
500 MHz, CDCl3
f1 (ppm)

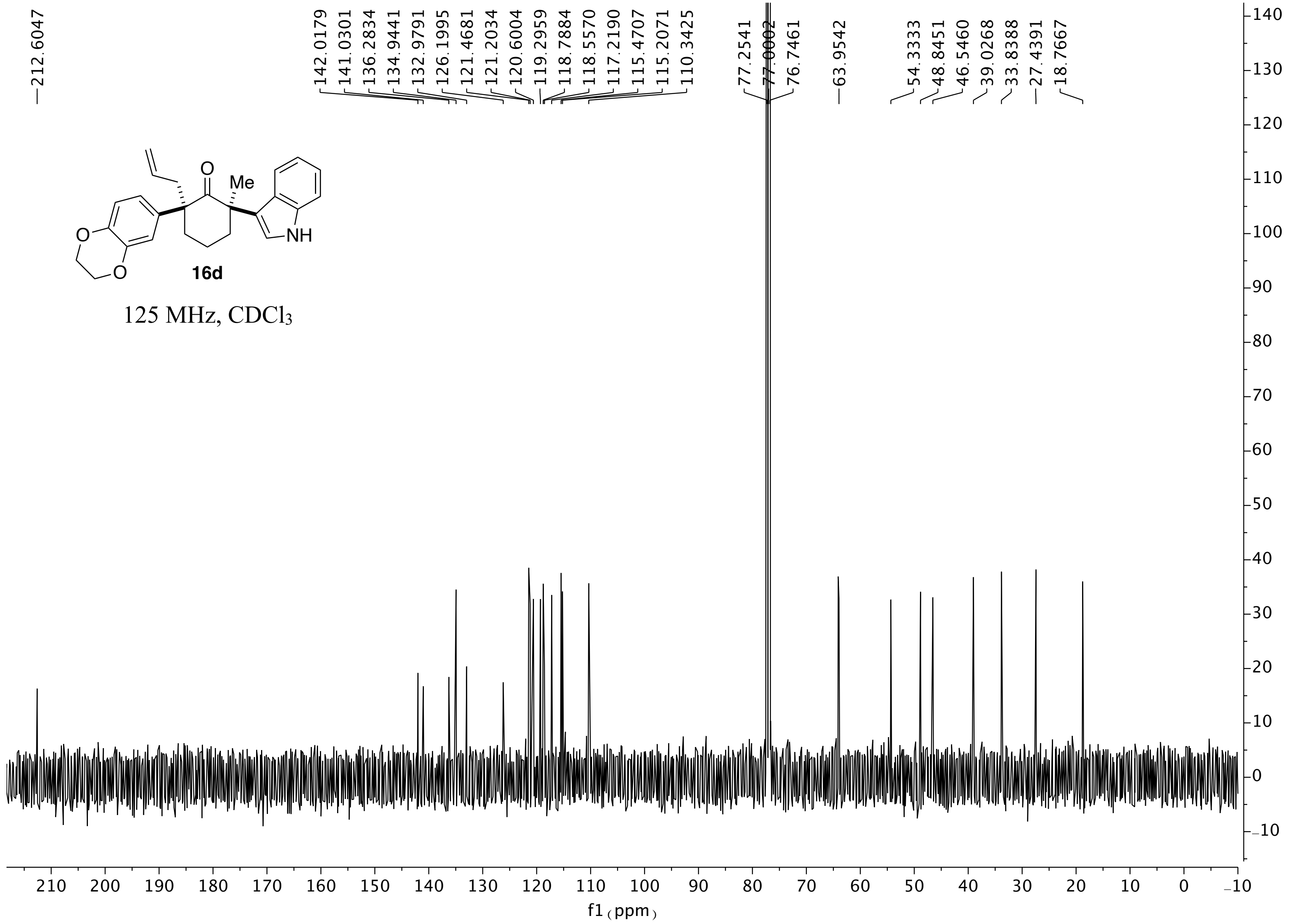

212.6047
142.0179
141.0301
136.2834
134.9441
132.9791
126.1995
121.4681
121.2034
120.6004
119.2959
118.7884
118.5570
117.2190
115.4707
115.2071
110.3425
77.2541
77.0002
76.7461
63.9542
54.3333
48.8451
46.5460
39.0268
33.8388
27.4391
18.7667
O
Me
NH
O
O
16d
125 MHz, CDCl3
f1 (ppm)

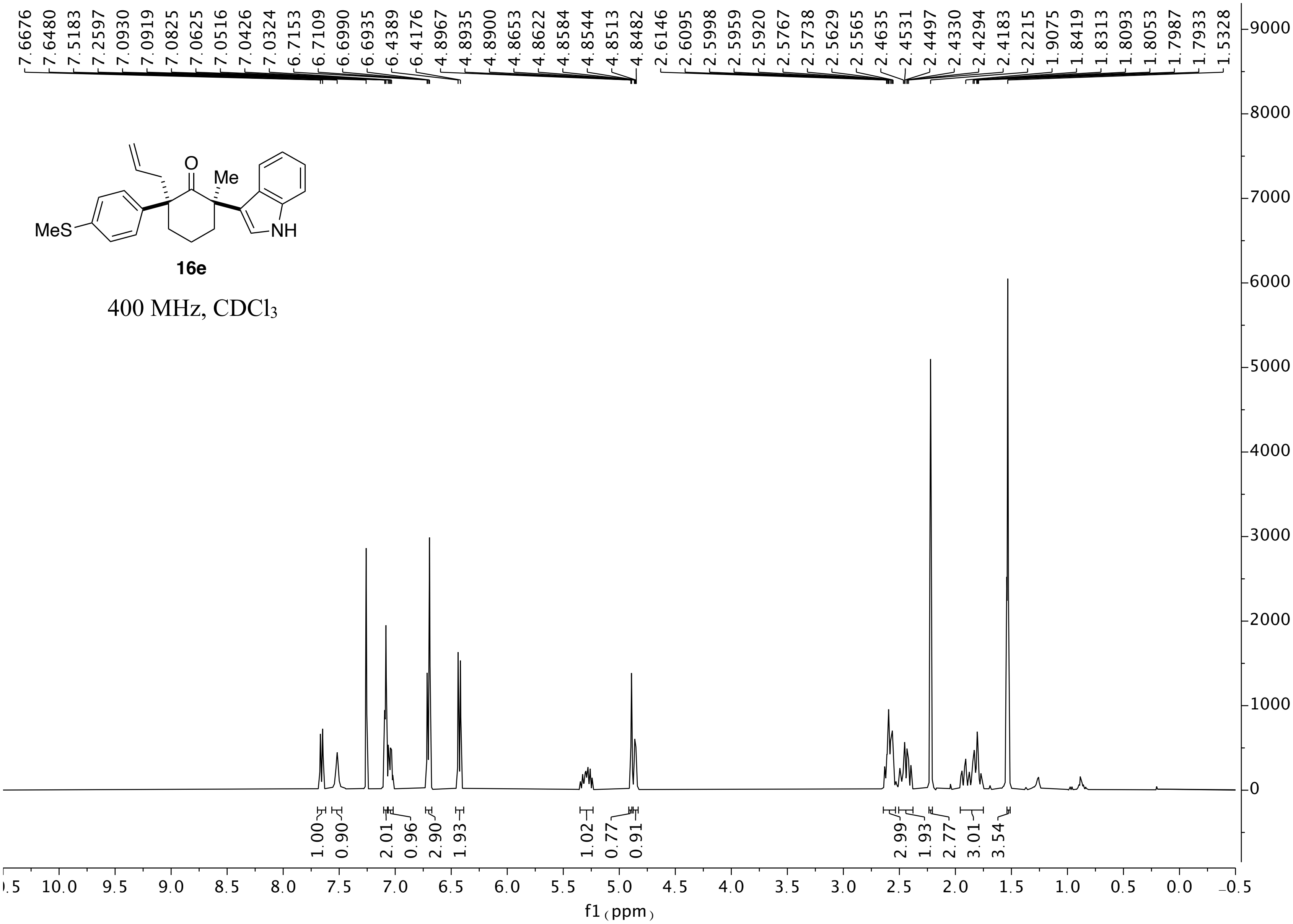
7.6676 7.6480 7.5183 7.2597 7.0930 7.0919 7.0825 7.0625 7.0516 7.0426 7.0324 6.7153 6.7109 6.6990 6.6935 6.4389 6.4176 4.8967 4.8935 4.8900 4.8653 4.8622 4.8584 4.8544 4.8513 4.8482 2.6146 2.6095 2.5998 2.5959 2.5920 2.5767 2.5738 2.5629 2.5565 2.4635 2.4531 2.4497 2.4330 2.4294 2.4183 2.2215 1.9075 1.8419 1.8313 1.8093 1.8053 1.7987 1.7933 1.5328
O
Me
MeS
NH
16e
400 MHz, CDCl3
1.00 0.90 2.01 0.96 2.90 1.93 1.02 0.77 0.91 2.99 1.93 2.77 3.01 3.54
f1 (ppm)

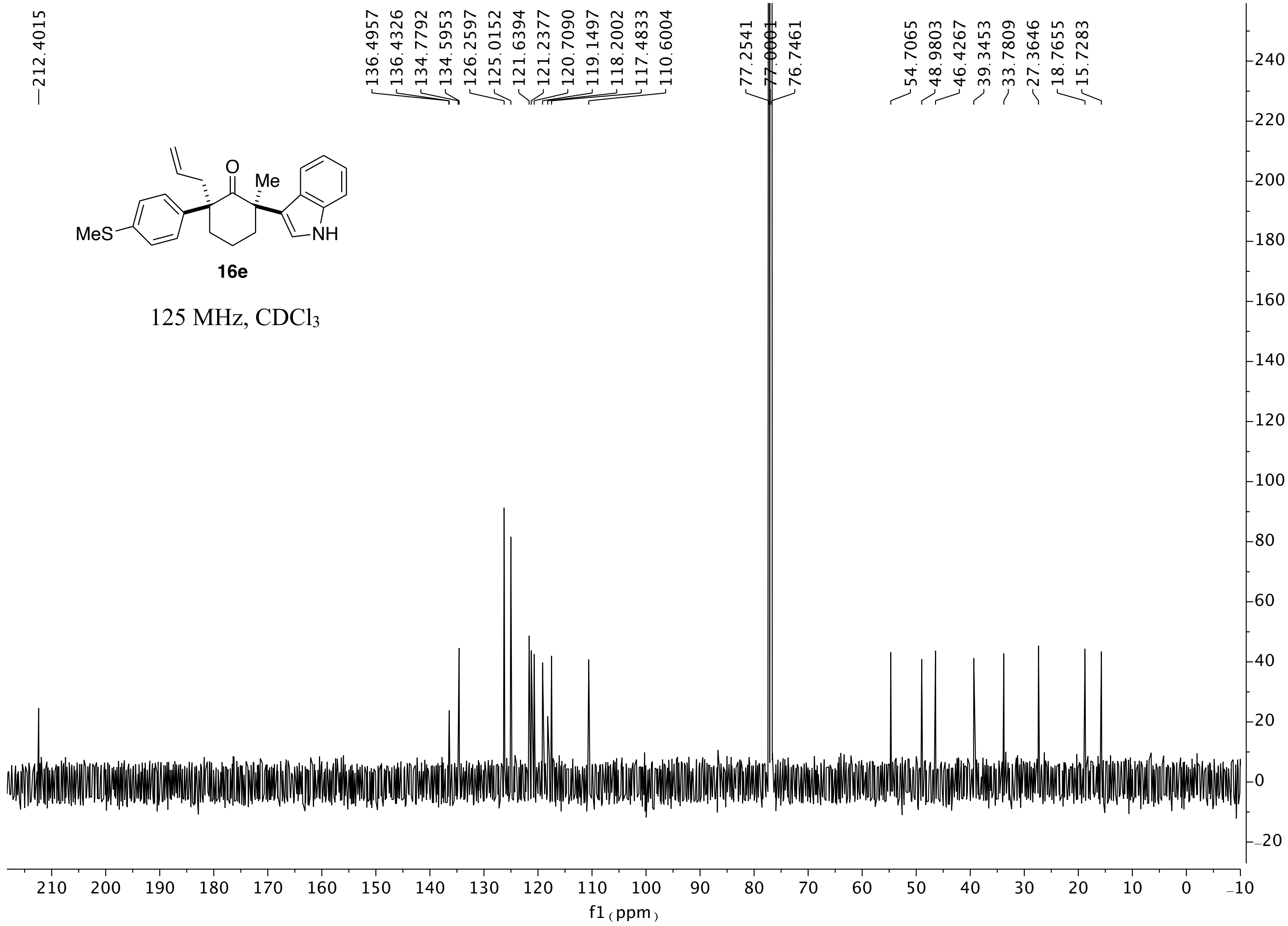

212.4015
136.4957
136.4326
134.7792
134.5953
126.2597
125.0152
121.6394
121.2377
120.7090
119.1497
118.2002
117.4833
110.6004
77.2541
77.0001
76.7461
54.7065
48.9803
46.4267
39.3453
33.7809
27.3646
18.7655
15.7283
MeS
O
Me
NH
16e
125 MHz, CDCl3
f1 (ppm)

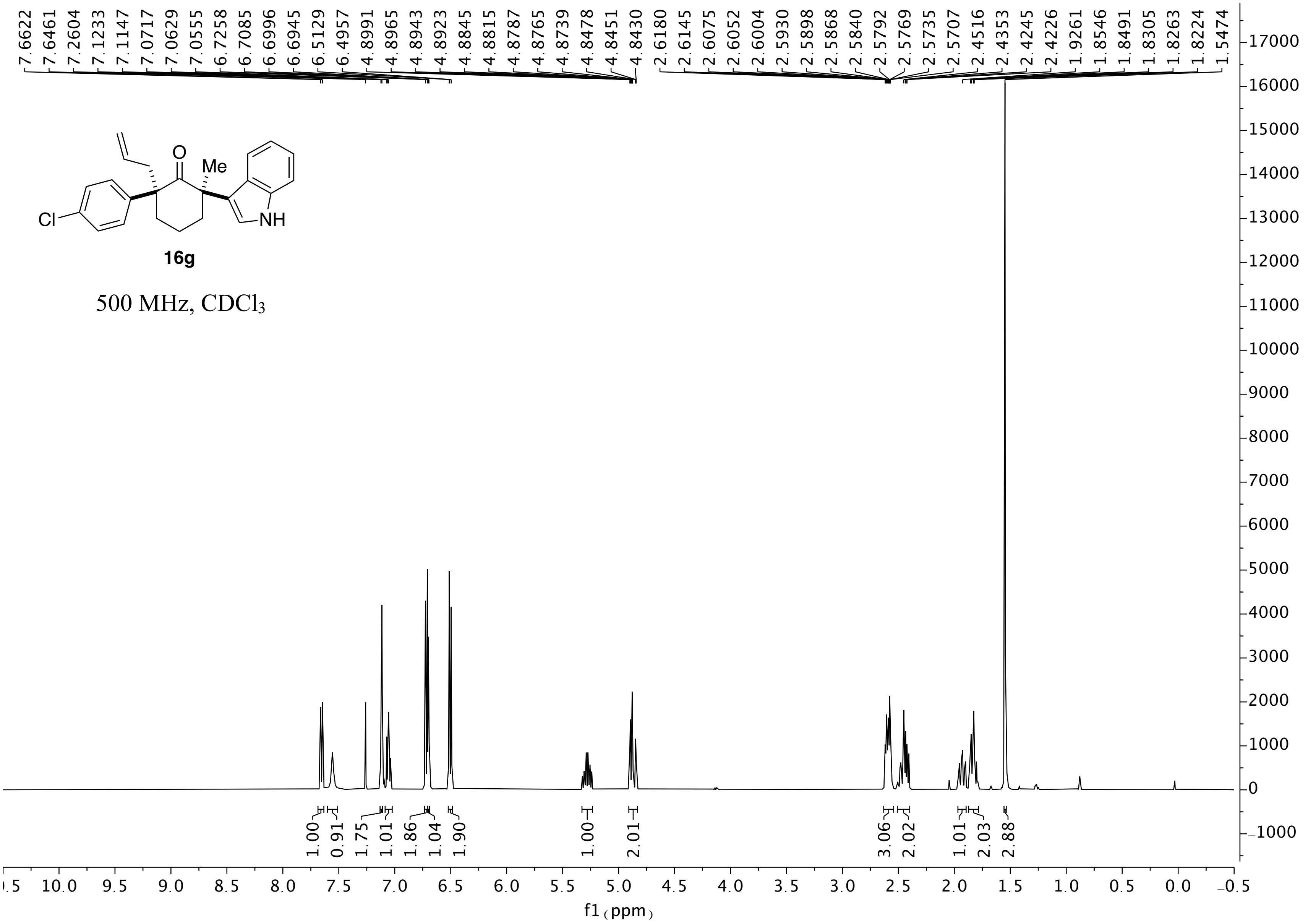
7.6622
7.6461
7.2604
7.1233
7.1147
7.0717
7.0629
7.0555
6.7258
6.7085
6.6996
6.6945
6.5129
6.4957
4.8991
4.8965
4.8943
4.8923
4.8845
4.8815
4.8787
4.8765
4.8739
4.8478
4.8451
4.8430
2.6180
2.6145
2.6075
2.6052
2.6004
2.5930
2.5898
2.5868
2.5840
2.5792
2.5769
2.5735
2.5707
2.4516
2.4353
2.4245
2.4226
1.9261
1.8546
1.8491
1.8305
1.8263
1.8224
1.5474
O
Me
Cl
NH
16g
500 MHz, CDCl3
1.00
0.91
1.75
1.01
1.86
1.04
1.90
1.00
2.01
3.06
2.02
1.01
2.03
2.88
f1 (ppm)

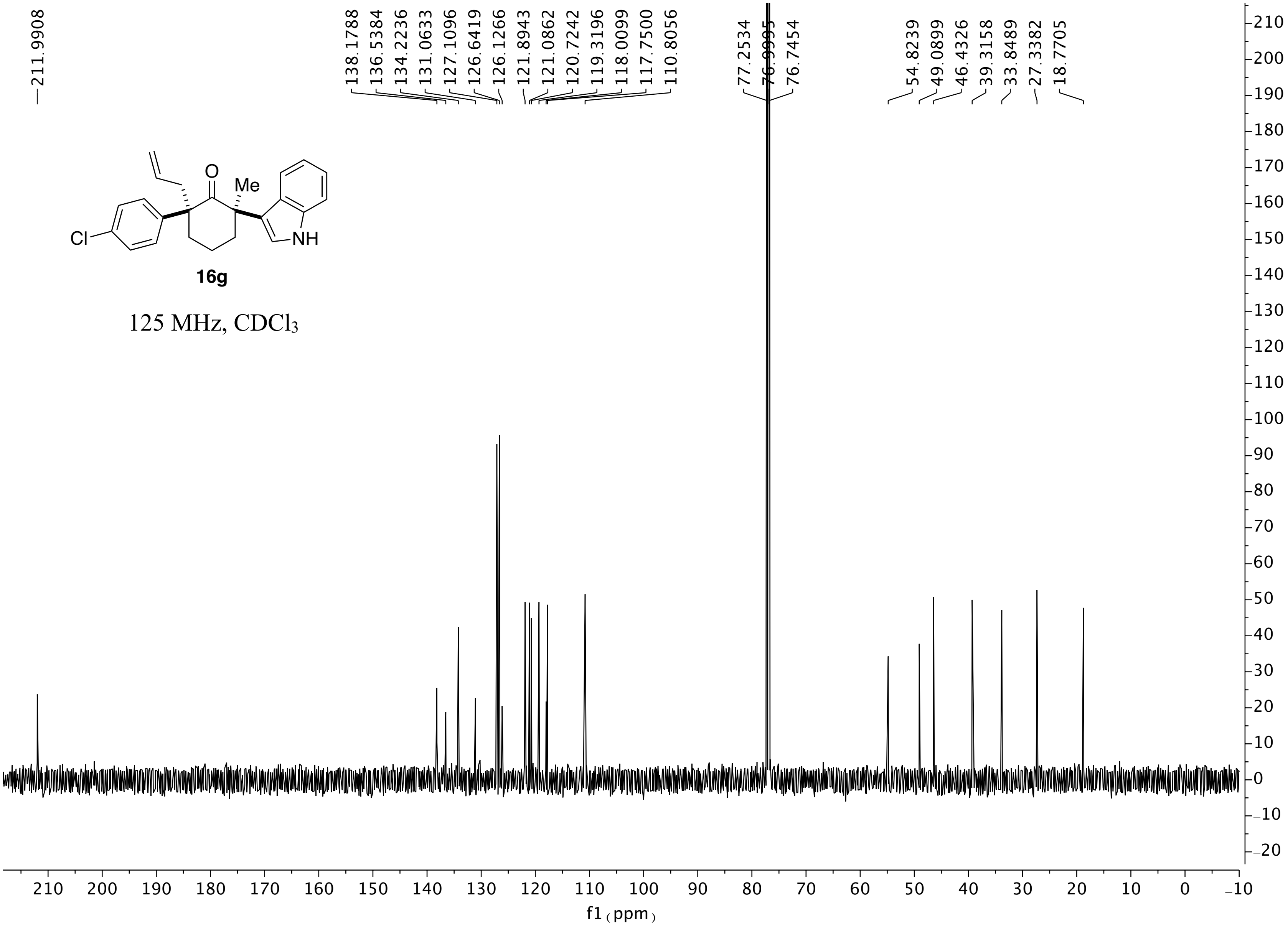
211.9908
138.1788
136.5384
134.2236
131.0633
127.1096
126.6419
126.1266
121.8943
121.0862
120.7242
119.3196
118.0099
117.7500
110.8056
77.2534
76.9995
76.7454
54.8239
49.0899
46.4326
39.3158
33.8489
27.3382
18.7705
O
Me
Cl
NH
16g
125 MHz, CDCl3
f1 (ppm)

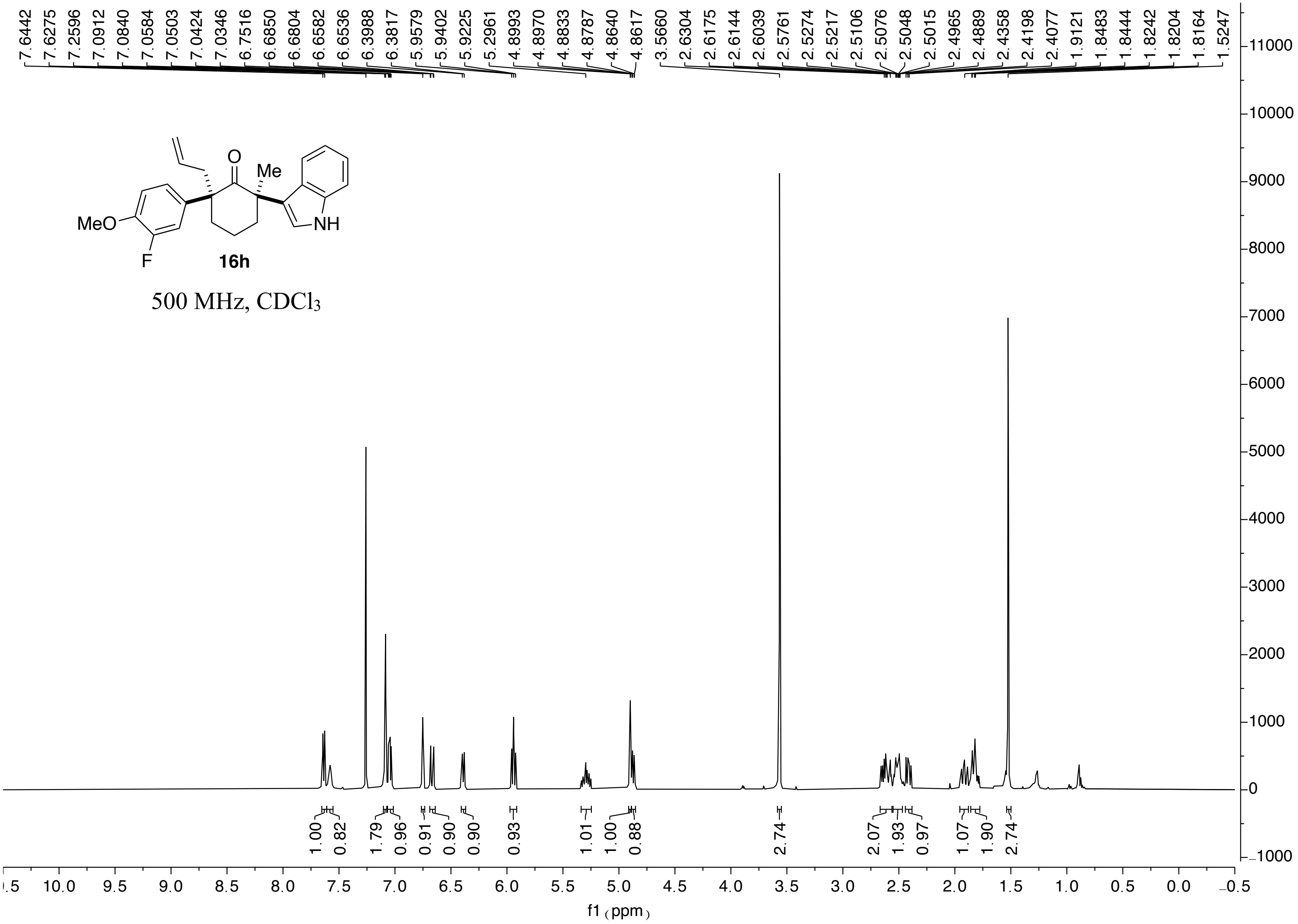
7.6442 7.6275 7.2596 7.0912 7.0840 7.0584 7.0503 7.0424 7.0346 6.7516 6.6850 6.6804 6.6582 6.6536 6.3988 6.3817 5.9579 5.9402 5.9225 5.2961 4.8993 4.8970 4.8833 4.8787 4.8640 4.8617
3.5660 2.6304 2.6175 2.6144 2.6039 2.5761 2.5274 2.5217 2.5106 2.5076 2.5048 2.5015 2.4965 2.4889 2.4358 2.4198 2.4077 1.9121 1.8483 1.8444 1.8242 1.8204 1.8164 1.5247
O
Me
MeO
NH
F
16h
500 MHz, CDCl3
1.00 0.82 1.79 0.96 0.91 0.90 0.90 0.93 1.01 1.00 0.88 2.74 2.07 1.93 0.97 1.07 1.90 2.74
f1 (ppm)

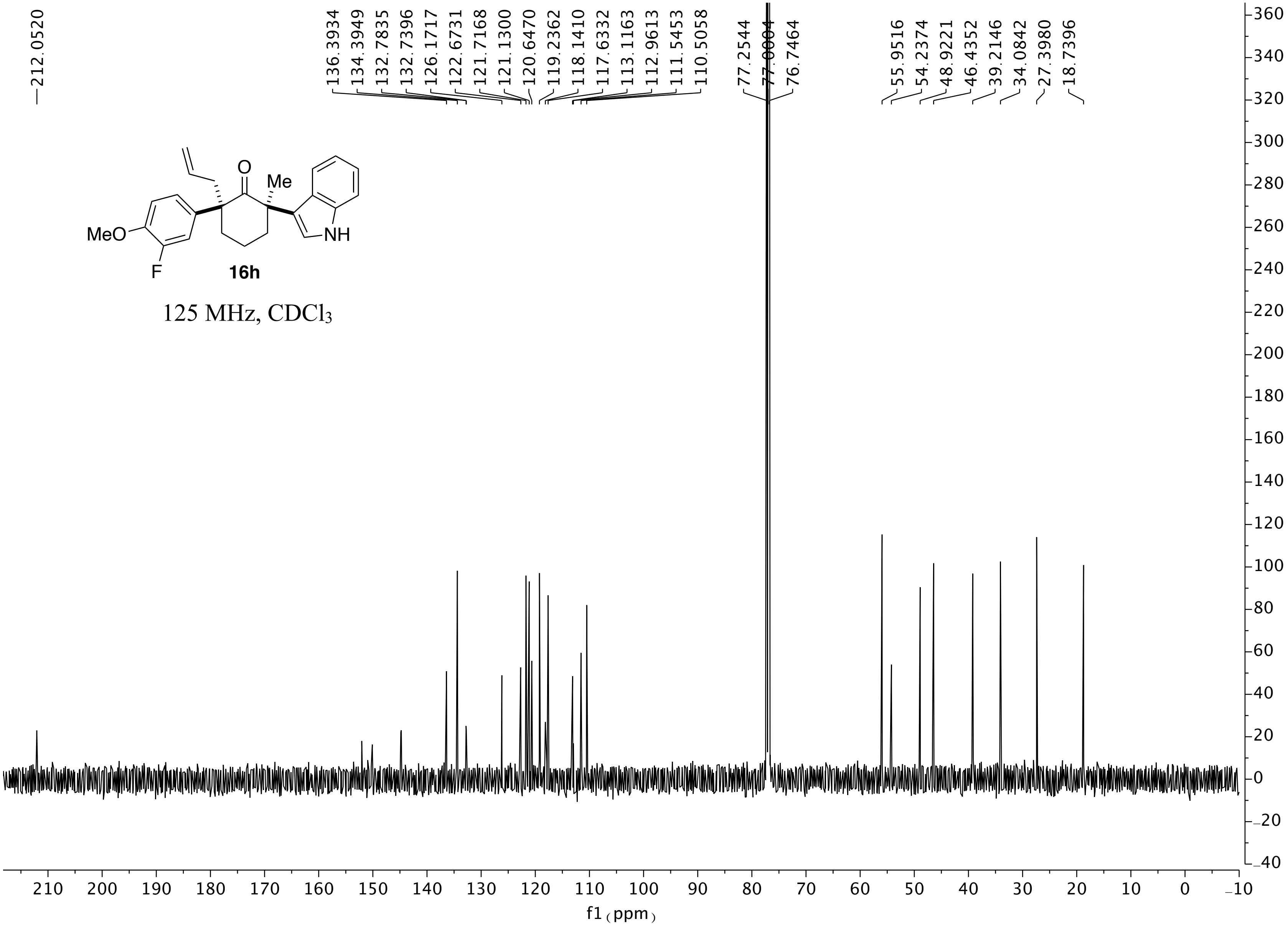
212.0520
136.3934
134.3949
132.7835
132.7396
126.1717
122.6731
121.7168
121.1300
120.6470
119.2362
118.1410
117.6332
113.1163
112.9613
111.5453
110.5058
77.2544
77.0004
76.7464
55.9516
54.2374
48.9221
46.4352
39.2146
34.0842
27.3980
18.7396
Me
O
MeO
F
NH
16h
125 MHz, CDCl3
f1 (ppm)

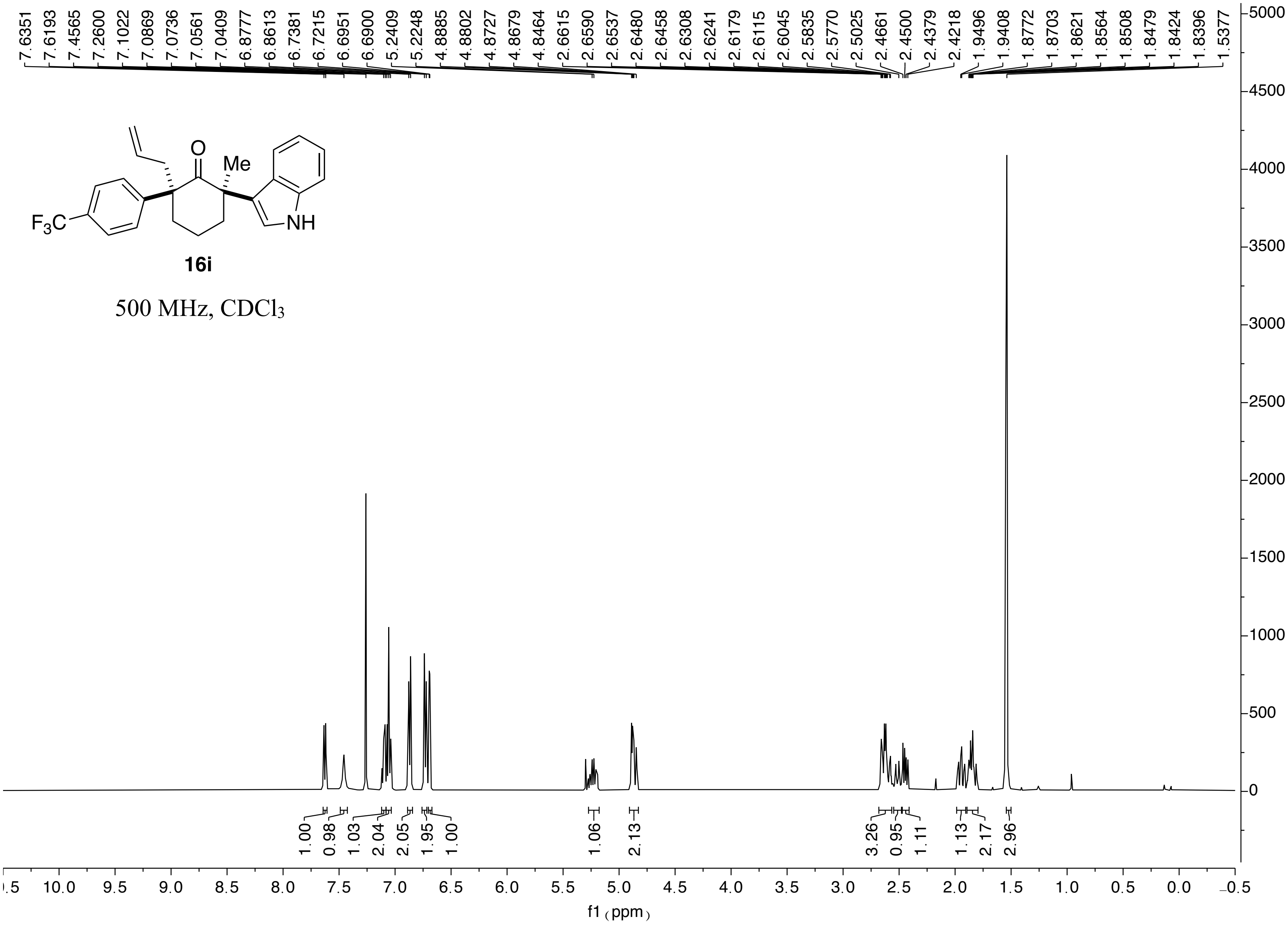

16i

500 MHz, $CDCl_3$

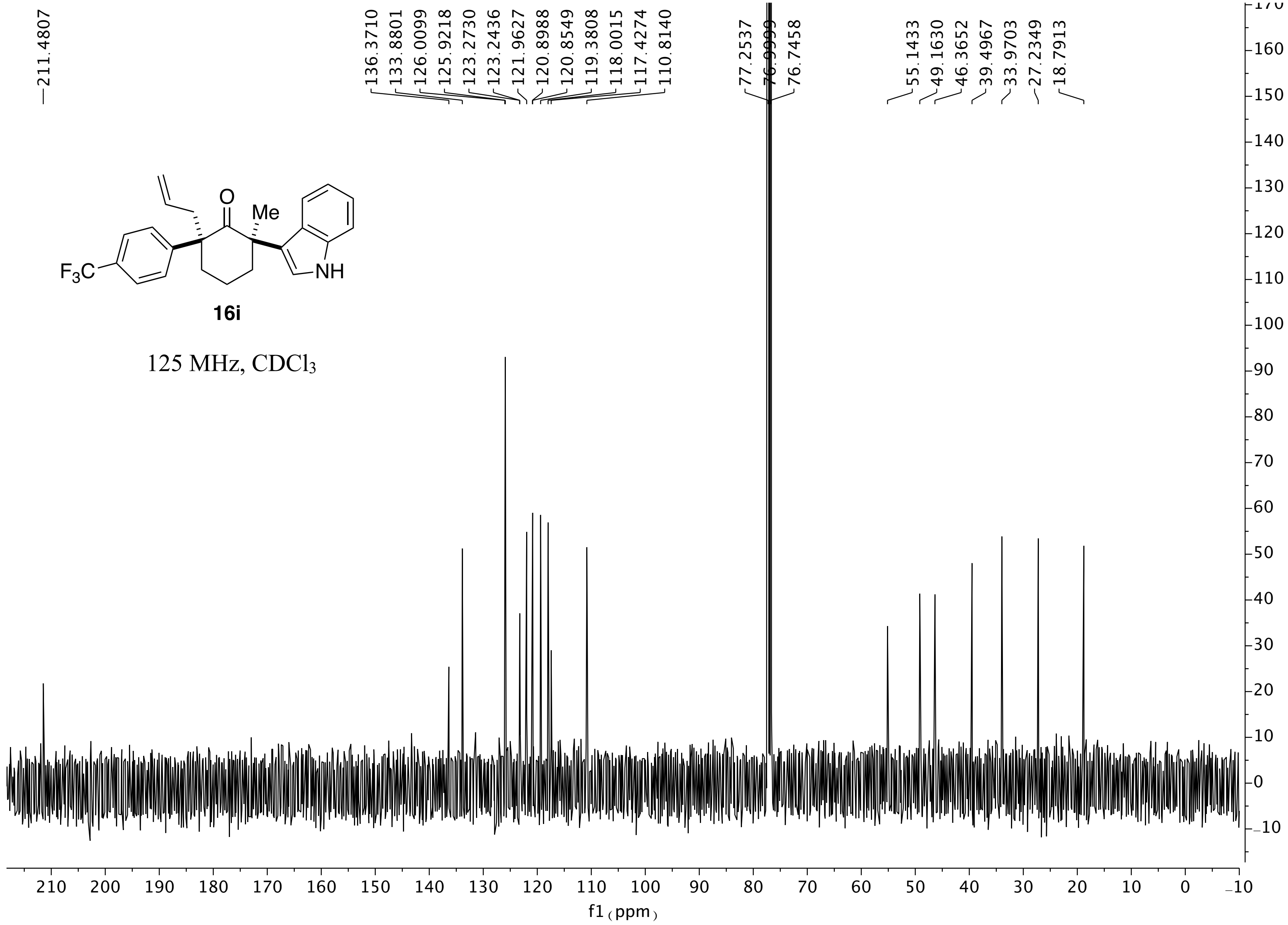
211.4807
136.3710
133.8801
126.0099
125.9218
123.2730
123.2436
121.9627
120.8988
120.8549
119.3808
118.0015
117.4274
110.8140
77.2537
76.9999
76.7458
55.1433
49.1630
46.3652
39.4967
33.9703
27.2349
18.7913
Me
O
F3C
NH
16i
125 MHz, CDCl3
f1 (ppm)

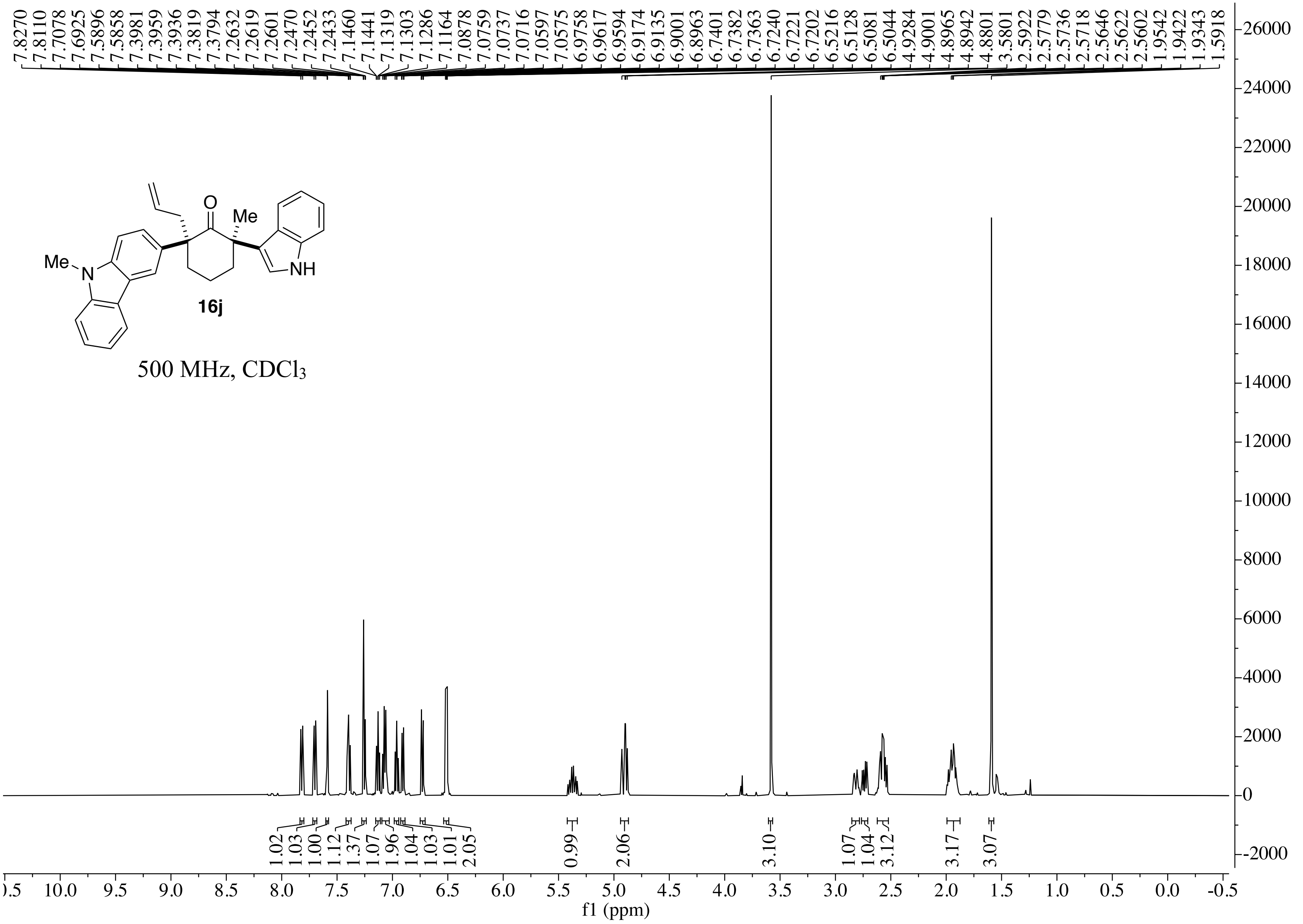

7.8270
7.8110
7.7078
7.6925
7.5896
7.5858
7.3981
7.3959
7.3936
7.3819
7.3794
7.2632
7.2619
7.2601
7.2470
7.2452
7.2433
7.1460
7.1441
7.1319
7.1303
7.1286
7.1164
7.0878
7.0759
7.0737
7.0716
7.0597
7.0575
6.9758
6.9617
6.9594
6.9174
6.9135
6.9001
6.8963
6.7401
6.7382
6.7363
6.7240
6.7221
6.7202
6.5216
6.5128
6.5081
6.5044
4.9284
4.9001
4.8965
4.8942
4.8801
3.5801
2.5922
2.5779
2.5736
2.5718
2.5646
2.5622
2.5602
1.9542
1.9422
1.9343
1.5918
O
Me
Me
N
NH
16j
500 MHz, $CDCl_3$
1.02
1.03
1.00
1.12
1.37
1.07
1.96
1.04
1.03
1.01
2.05
0.99
2.06
3.10
1.07
1.04
3.12
3.17
3.07
f1 (ppm)

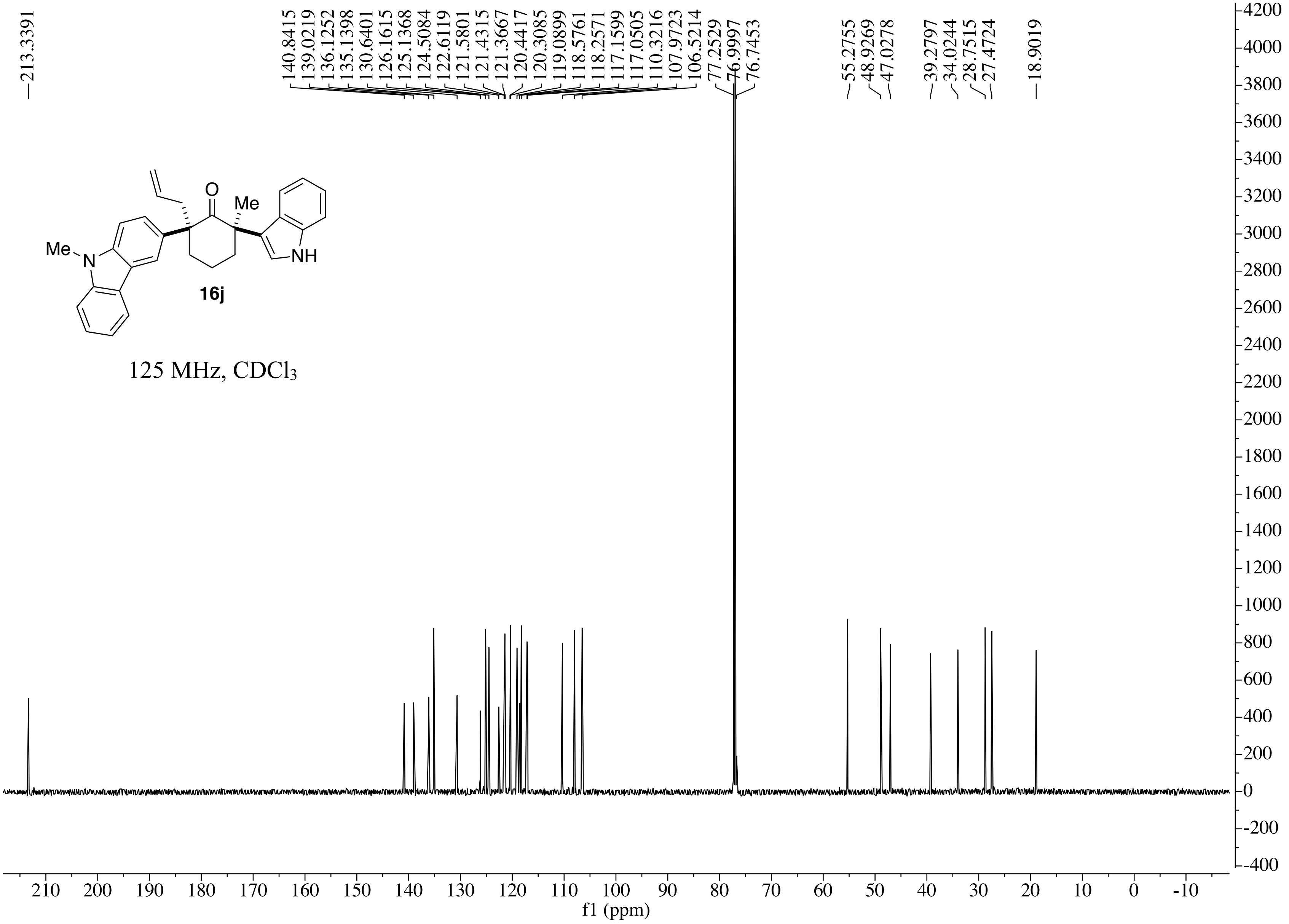
213.3391
140.8415
139.0219
136.1252
135.1398
130.6401
126.1615
125.1368
124.5084
122.6119
121.5801
121.4315
121.3667
120.4417
120.3085
119.0899
118.5761
118.2571
117.1599
117.0505
110.3216
107.9723
106.5214
77.2529
76.9997
76.7453
55.2755
48.9269
47.0278
39.2797
34.0244
28.7515
27.4724
18.9019
O
Me
Me
N
NH
16j
125 MHz, CDCl3
f1 (ppm)

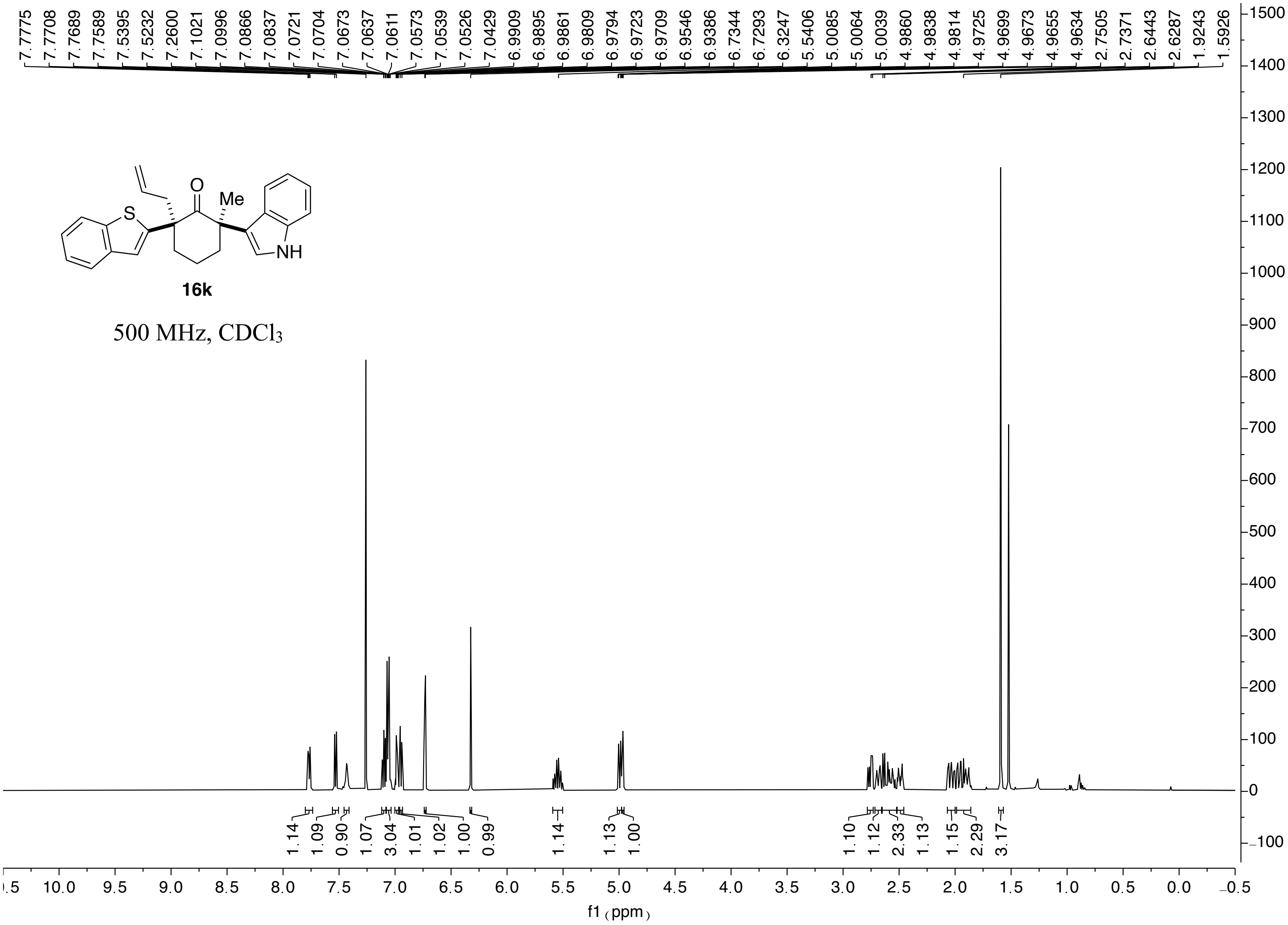
7.7775
7.7708
7.7689
7.7589
7.5395
7.5232
7.2600
7.1021
7.0996
7.0866
7.0837
7.0721
7.0704
7.0673
7.0637
7.0611
7.0573
7.0539
7.0526
7.0429
6.9909
6.9895
6.9861
6.9809
6.9794
6.9723
6.9709
6.9546
6.9386
6.7344
6.7293
6.3247
5.5406
5.0085
5.0064
5.0039
4.9860
4.9838
4.9814
4.9725
4.9699
4.9673
4.9655
4.9634
2.7505
2.7371
2.6443
2.6287
1.9243
1.5926
O
Me
S
NH
16k
500 MHz, CDCl3
1.14
1.09
0.90
1.07
3.04
1.01
1.02
1.00
0.99
1.14
1.13
1.00
1.10
1.12
2.33
1.13
1.15
2.29
3.17
f1 (ppm)

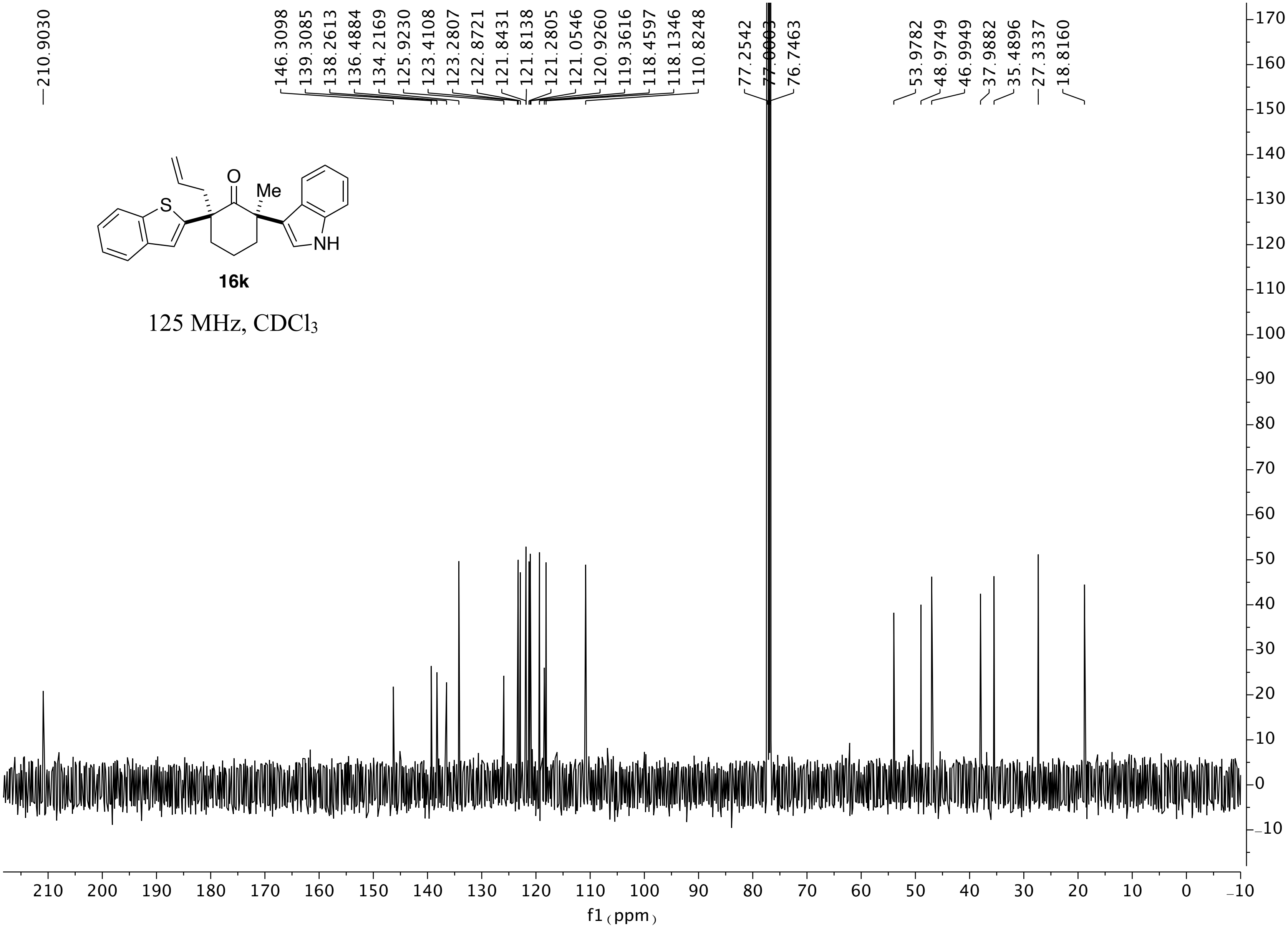

210.9030
146.3098
139.3085
138.2613
136.4884
134.2169
125.9230
123.4108
123.2807
122.8721
121.8431
121.8138
121.2805
121.0546
120.9260
119.3616
118.4597
118.1346
110.8248
77.2542
77.0003
76.7463
53.9782
48.9749
46.9949
37.9882
35.4896
27.3337
18.8160
S
O
Me
NH
16k
125 MHz, $CDCl_3$
210 200 190 180 170 160 150 140 130 120 110 100 90 80 70 60 50 40 30 20 10 0 -10
f1 (ppm)
170 160 150 140 130 120 110 100 90 80 70 60 50 40 30 20 10 0 -10

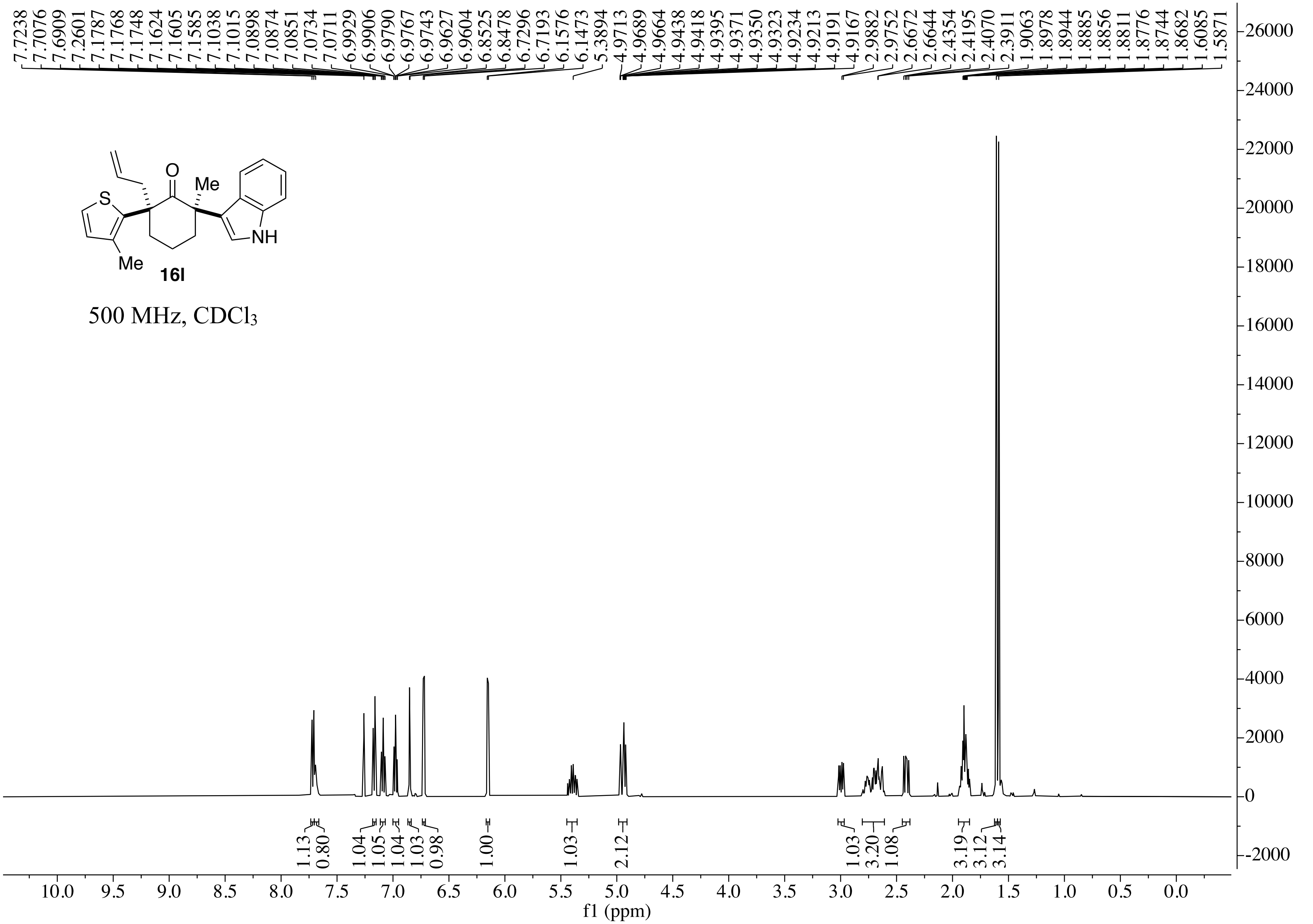

O
Me
S
NH
Me
16l
500 MHz, $CDCl_3$
7.7238
7.7076
7.6909
7.2601
7.1787
7.1768
7.1748
7.1624
7.1605
7.1585
7.1038
7.1015
7.0898
7.0874
7.0851
7.0734
7.0711
6.9929
6.9906
6.9790
6.9767
6.9743
6.9627
6.9604
6.8525
6.8478
6.7296
6.7193
6.1576
6.1473
5.3894
4.9713
4.9689
4.9664
4.9438
4.9418
4.9395
4.9371
4.9350
4.9323
4.9234
4.9213
4.9191
4.9167
2.9882
2.9752
2.6672
2.6644
2.4354
2.4195
2.4070
2.3911
1.9063
1.8978
1.8944
1.8885
1.8856
1.8811
1.8776
1.8744
1.8682
1.6085
1.5871
1.13
0.80
1.04
1.05
1.04
1.03
0.98
1.00
1.03
2.12
1.03
3.20
1.08
3.19
3.12
3.14
10.0
9.5
9.0
8.5
8.0
7.5
7.0
6.5
6.0
5.5
5.0
4.5
4.0
3.5
3.0
2.5
2.0
1.5
1.0
0.5
0.0
f1 (ppm)
26000
24000
22000
20000
18000
16000
14000
12000
10000
8000
6000
4000
2000
0
-2000

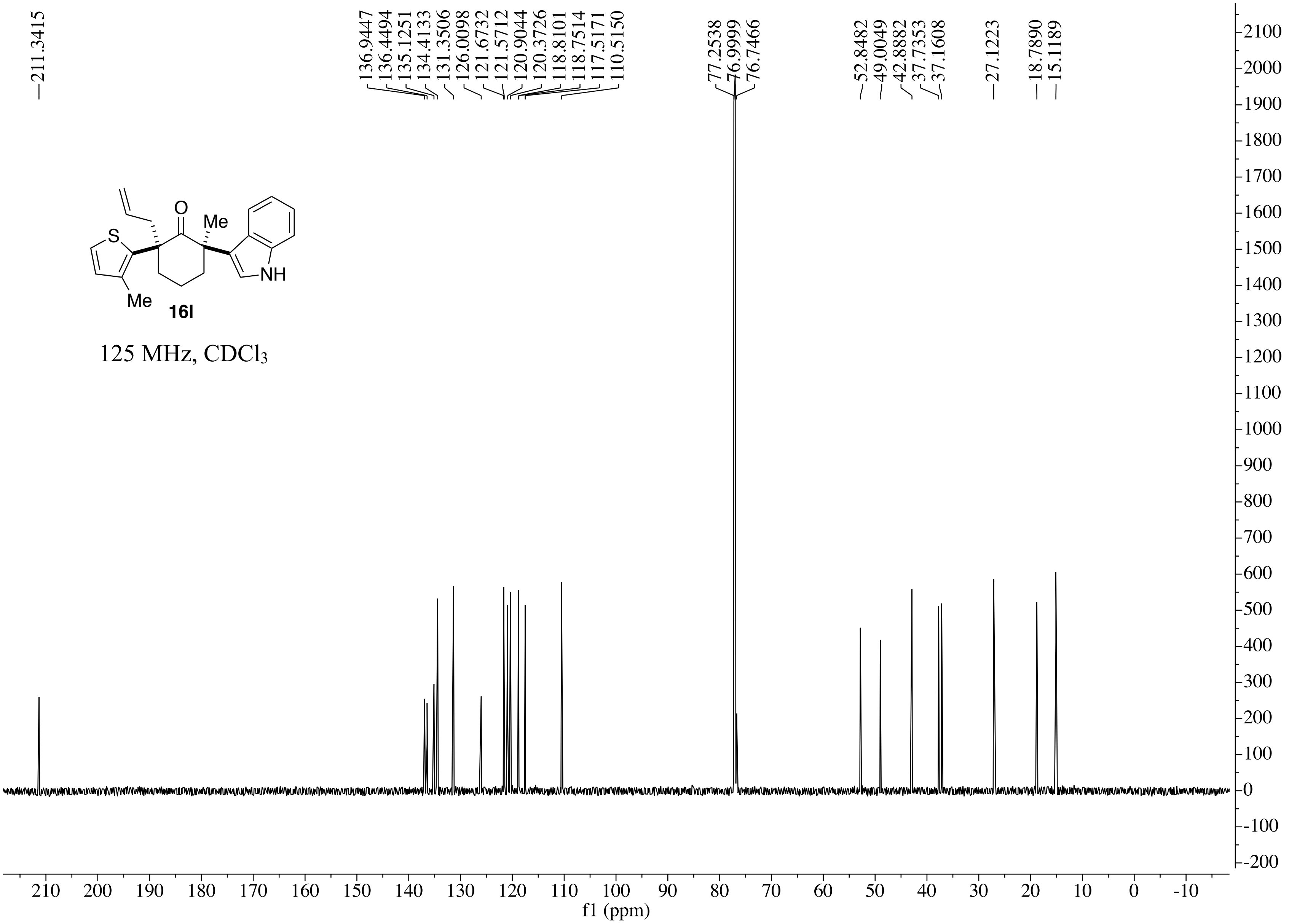
211.3415
136.9447
136.4494
135.1251
134.4133
131.3506
126.0098
121.6732
121.5712
120.9044
120.3726
118.8101
118.7514
117.5171
110.5150
77.2538
76.9999
76.7466
52.8482
49.0049
42.8882
37.7353
37.1608
27.1223
18.7890
15.1189
S
O
Me
NH
Me
16l
125 MHz, CDCl3
f1 (ppm)

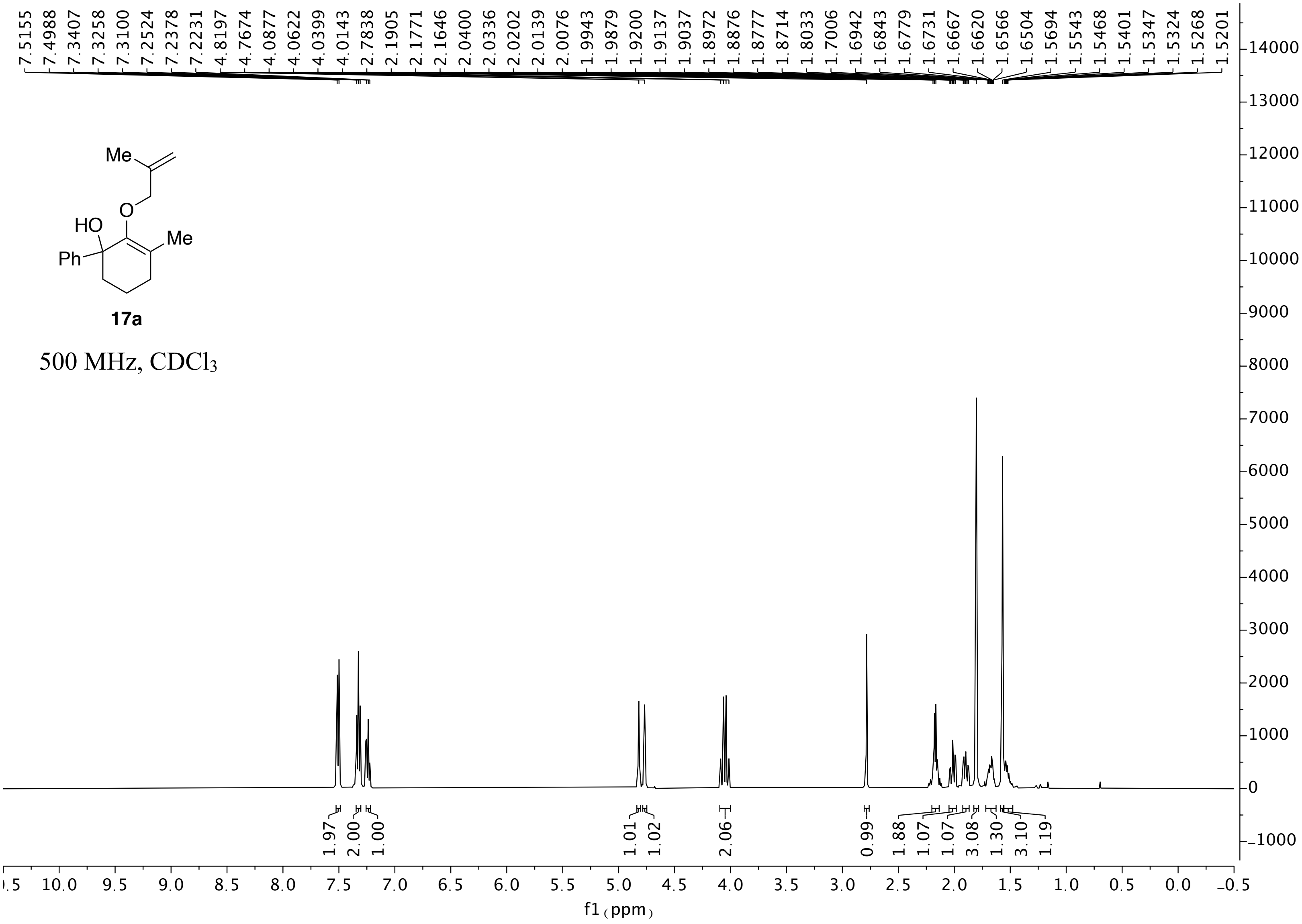

7.5155
7.4988
7.3407
7.3258
7.3100
7.2524
7.2378
7.2231
4.8197
4.7674
4.0877
4.0622
4.0399
4.0143
2.7838
2.1905
2.1771
2.1646
2.0400
2.0336
2.0202
2.0139
2.0076
1.9943
1.9879
1.9200
1.9137
1.9037
1.8972
1.8876
1.8777
1.8714
1.8033
1.7006
1.6942
1.6843
1.6779
1.6731
1.6667
1.6620
1.6566
1.6504
1.5694
1.5543
1.5468
1.5401
1.5347
1.5324
1.5268
1.5201
Me
O
HO
Me
Ph
17a
500 MHz, CDCl3
1.97
2.00
1.00
1.01
1.02
2.06
0.99
1.88
1.07
1.07
3.08
1.30
3.10
1.19
f1 (ppm)

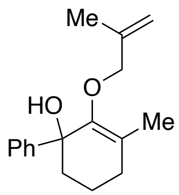

17a

125 MHz, CDCl3

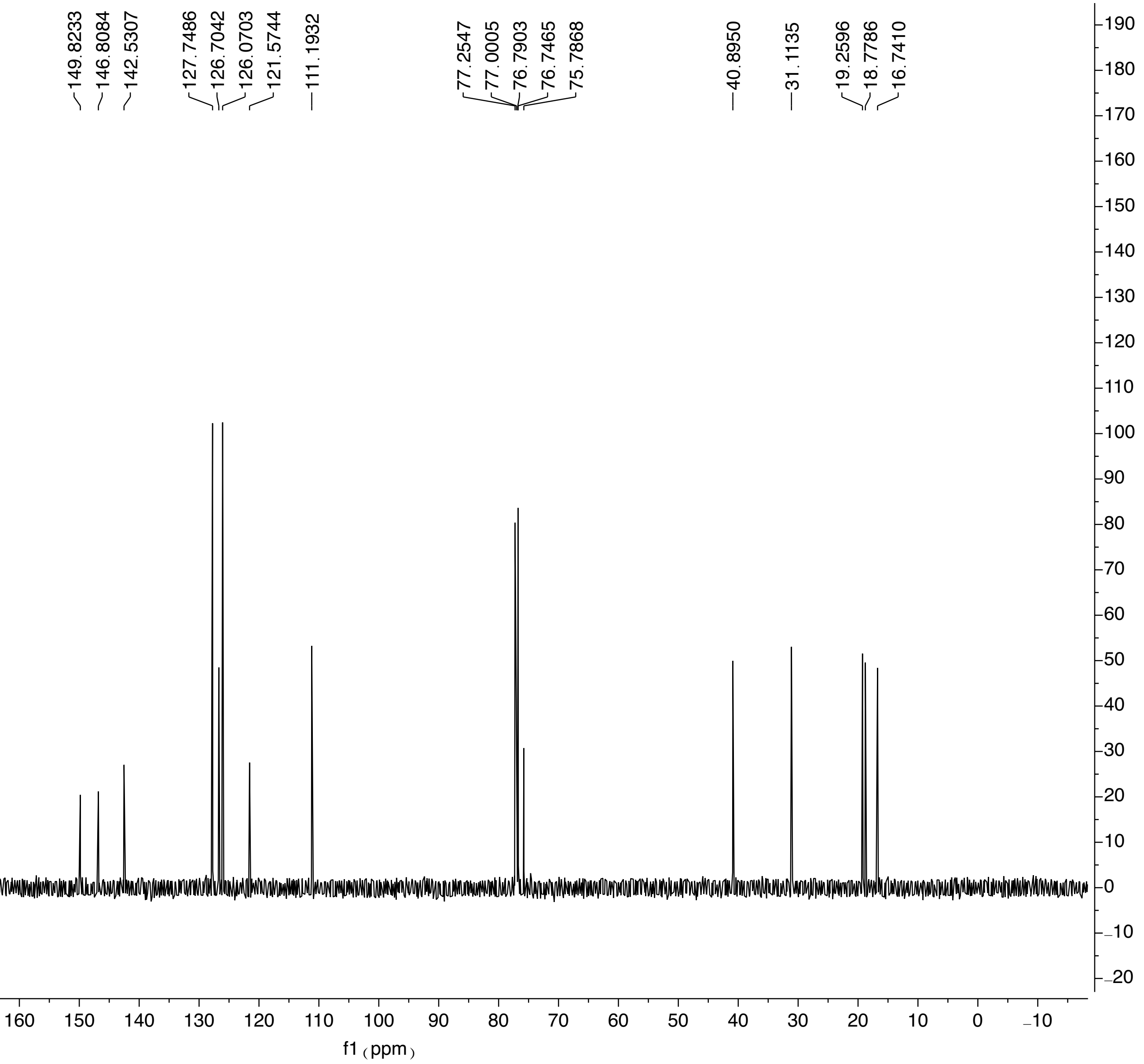

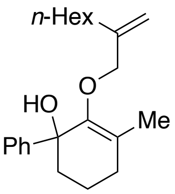

17b

400 MHz, $CDCl_3$

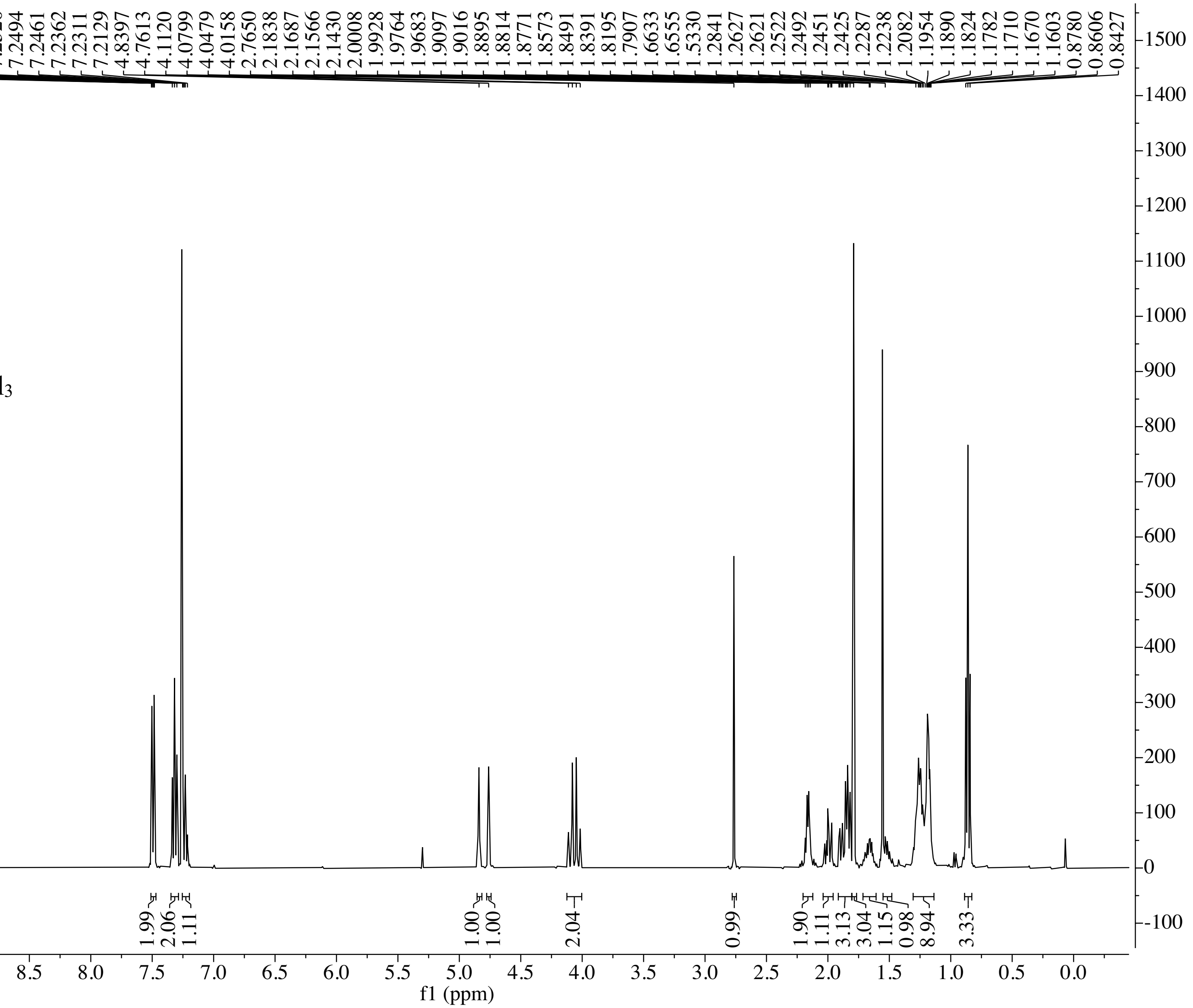

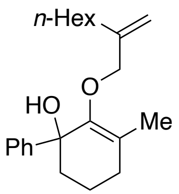

17b

125 MHz, $CDCl_3$

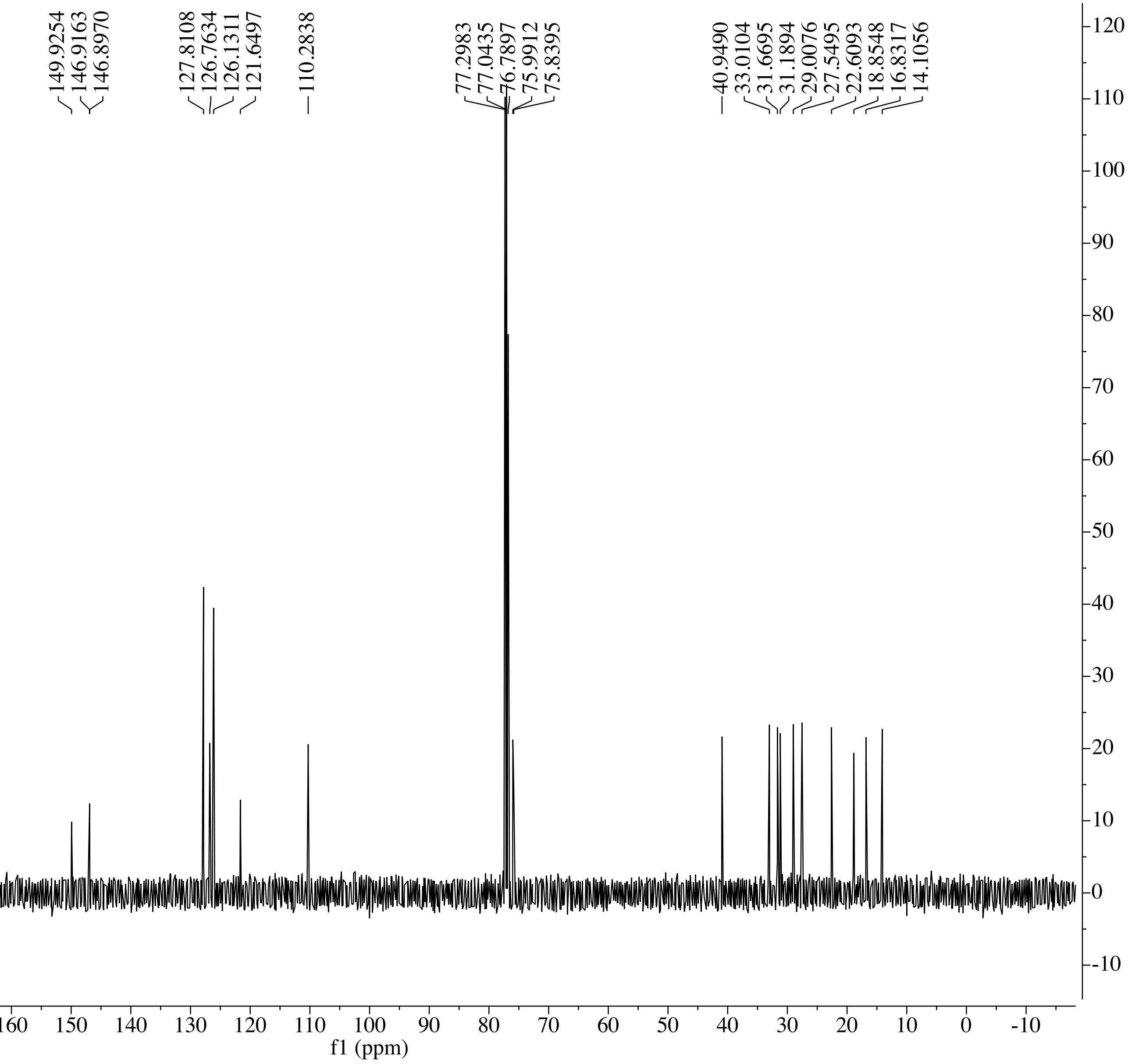

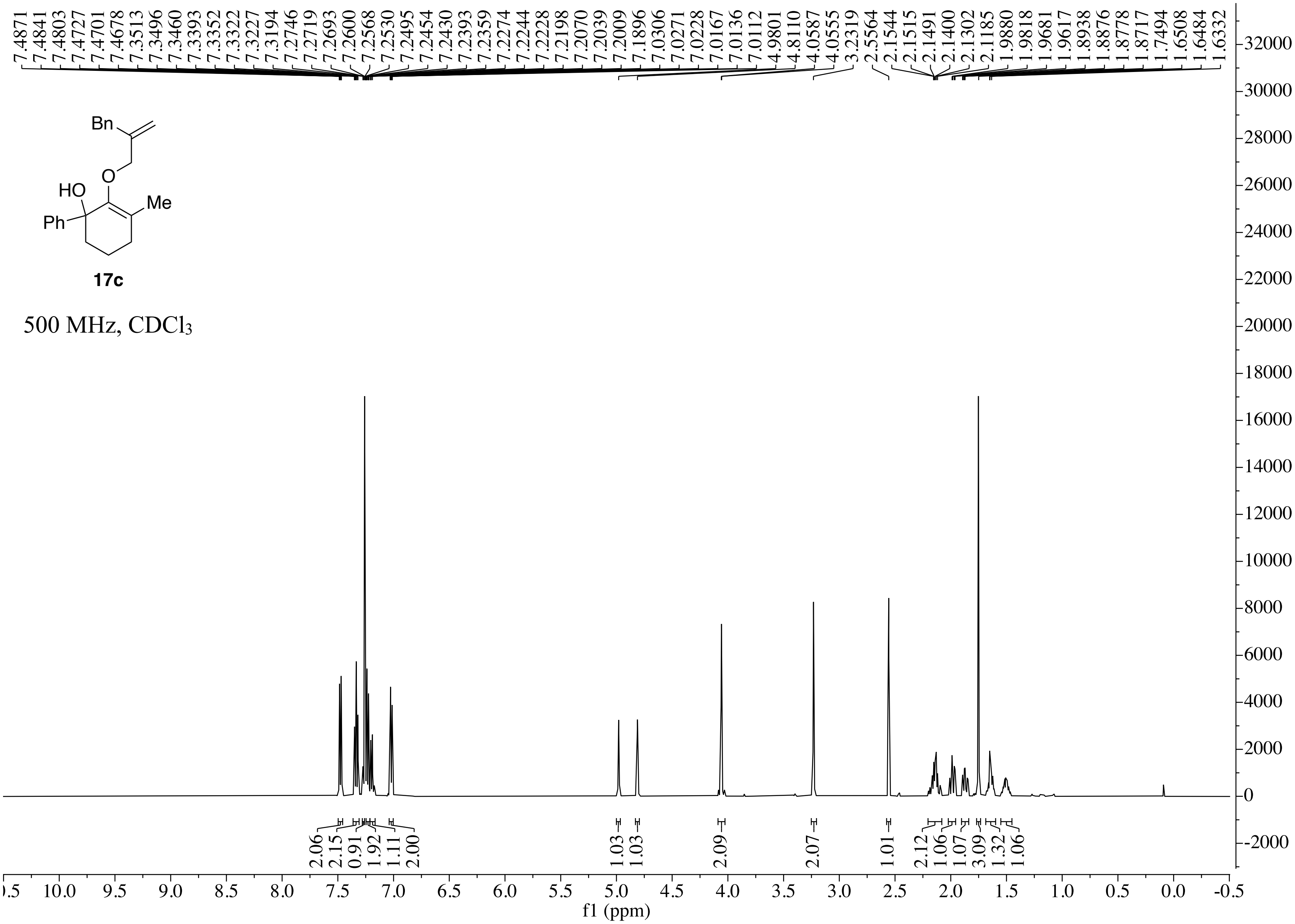

17c

500 MHz, $CDCl_3$

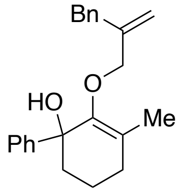

17c

125 MHz, $CDCl_3$

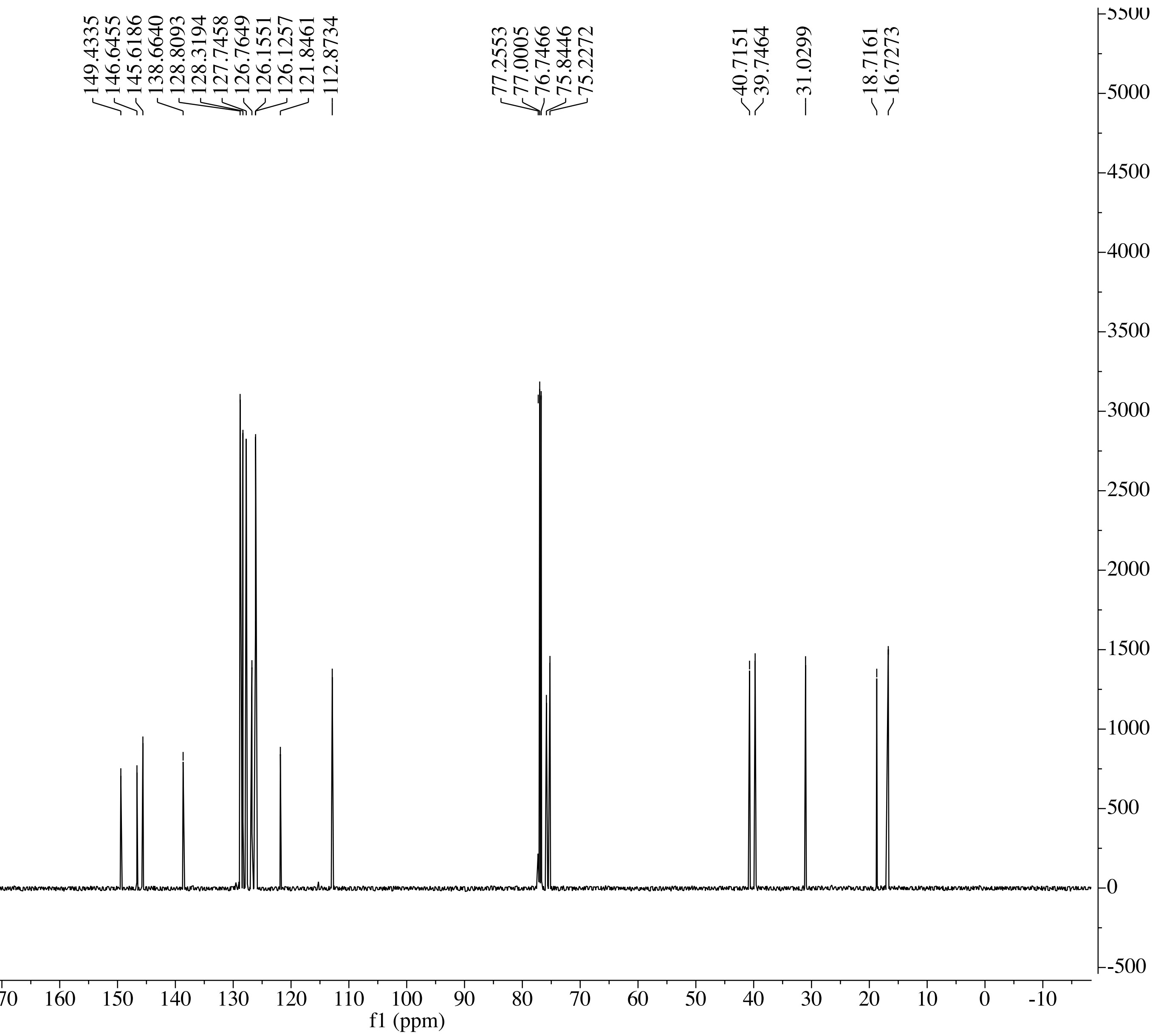

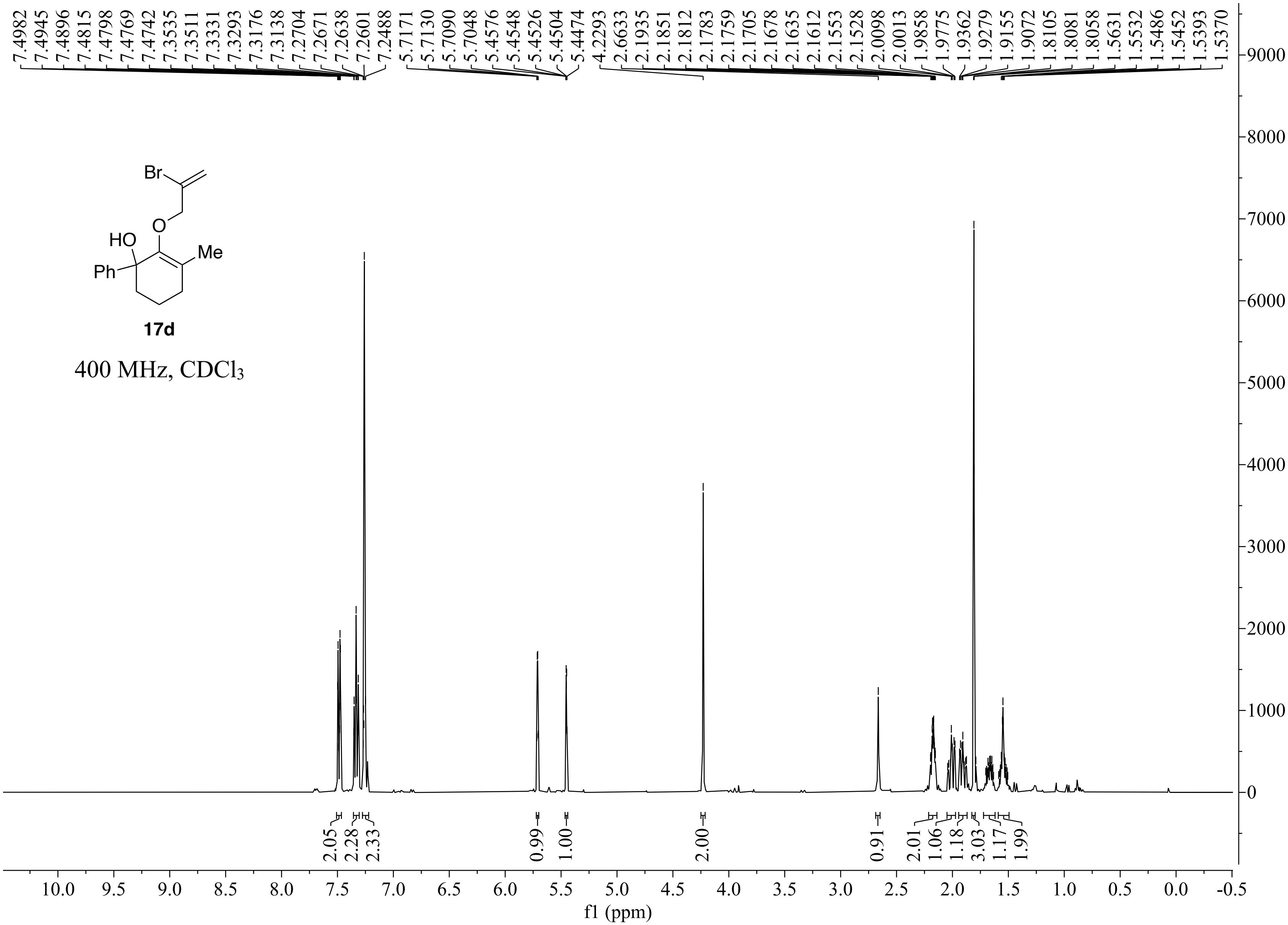

7.4982
7.4945
7.4896
7.4815
7.4798
7.4769
7.4742
7.3535
7.3511
7.3331
7.3293
7.3176
7.3138
7.2704
7.2671
7.2638
7.2601
7.2488
5.7171
5.7130
5.7090
5.7048
5.4576
5.4548
5.4526
5.4504
5.4474
4.2293
2.6633
2.1935
2.1851
2.1812
2.1783
2.1759
2.1705
2.1678
2.1635
2.1612
2.1553
2.1528
2.0098
2.0013
1.9858
1.9775
1.9362
1.9279
1.9155
1.9072
1.8105
1.8081
1.8058
1.5631
1.5532
1.5486
1.5452
1.5393
1.5370
Br
O
HO
Me
Ph
17d
400 MHz, $CDCl_3$
2.05
2.28
2.33
0.99
1.00
2.00
0.91
2.01
1.06
1.18
3.03
1.17
1.99
f1 (ppm)

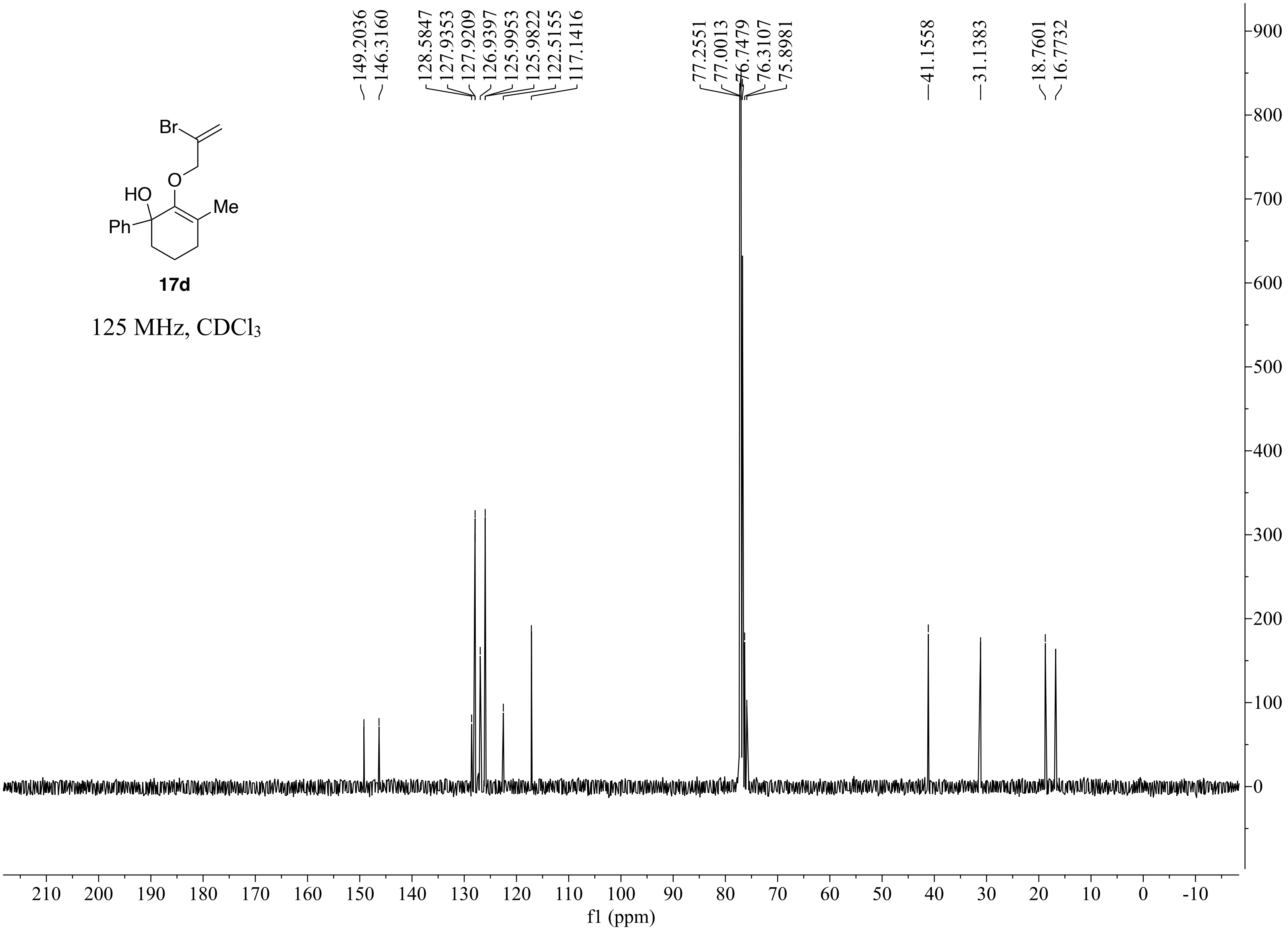

Br
HO
O
Me
Ph
17d
125 MHz, CDCl3
149.2036
146.3160
128.5847
127.9353
127.9209
126.9397
125.9953
125.9822
122.5155
117.1416
77.2551
77.0013
76.7479
76.3107
75.8981
41.1558
31.1383
18.7601
16.7732
f1 (ppm)

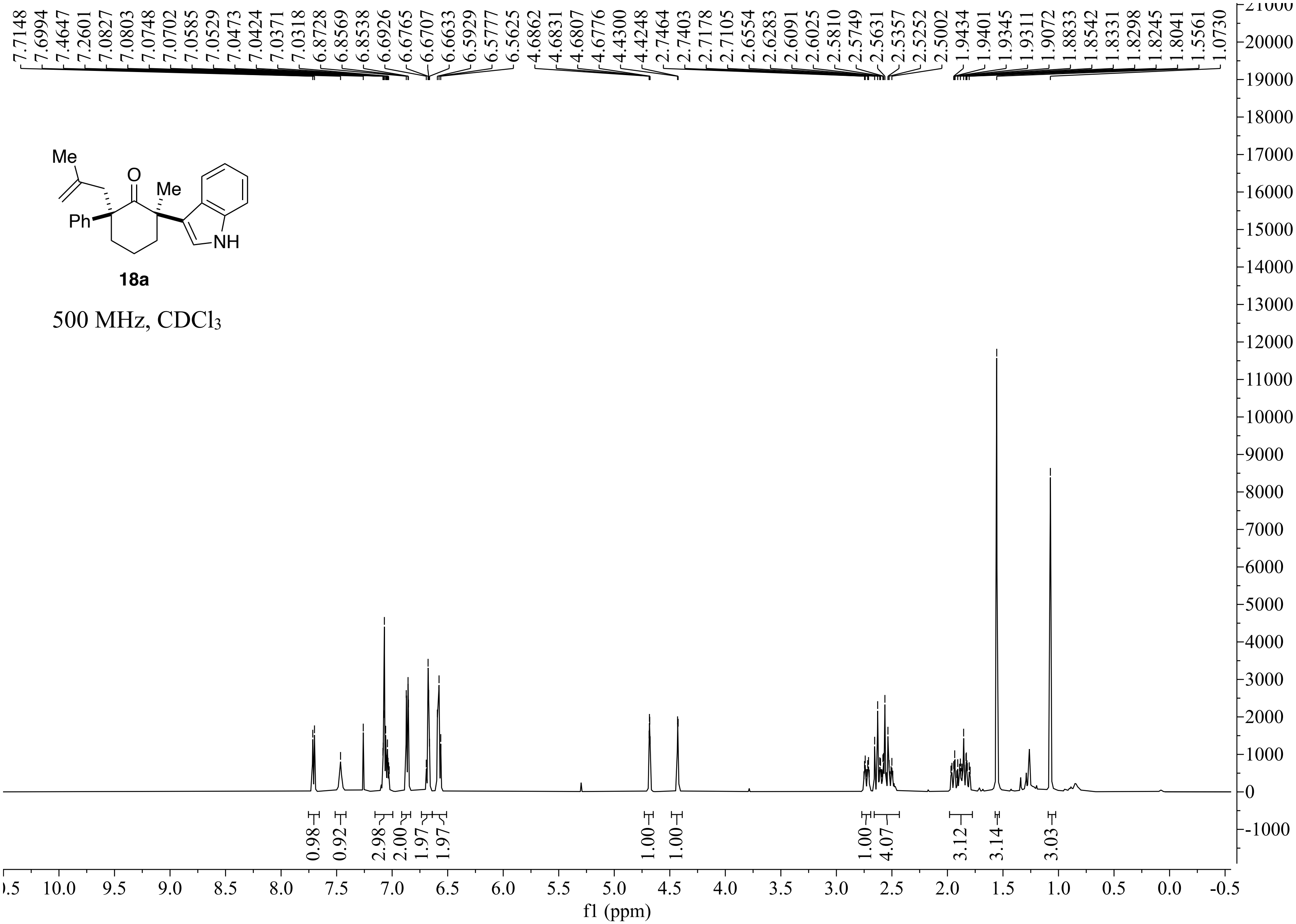
Me
O
Me
Ph
NH
18a
500 MHz, CDCl3
7.7148 7.6994 7.4647 7.2601 7.0827 7.0803 7.0748 7.0702 7.0585 7.0529 7.0473 7.0424 7.0371 7.0318 6.8728 6.8569 6.8538 6.6926 6.6765 6.6707 6.6633 6.5929 6.5777 6.5625 4.6862 4.6831 4.6807 4.6776 4.4300 4.4248 2.7464 2.7403 2.7178 2.7105 2.6554 2.6283 2.6091 2.6025 2.5810 2.5749 2.5631 2.5357 2.5252 2.5002 1.9434 1.9401 1.9345 1.9311 1.9072 1.8833 1.8542 1.8331 1.8298 1.8245 1.8041 1.5561 1.0730
0.98 0.92 2.98 2.00 1.97 1.97 1.00 1.00 1.00 4.07 3.12 3.14 3.03
f1 (ppm)

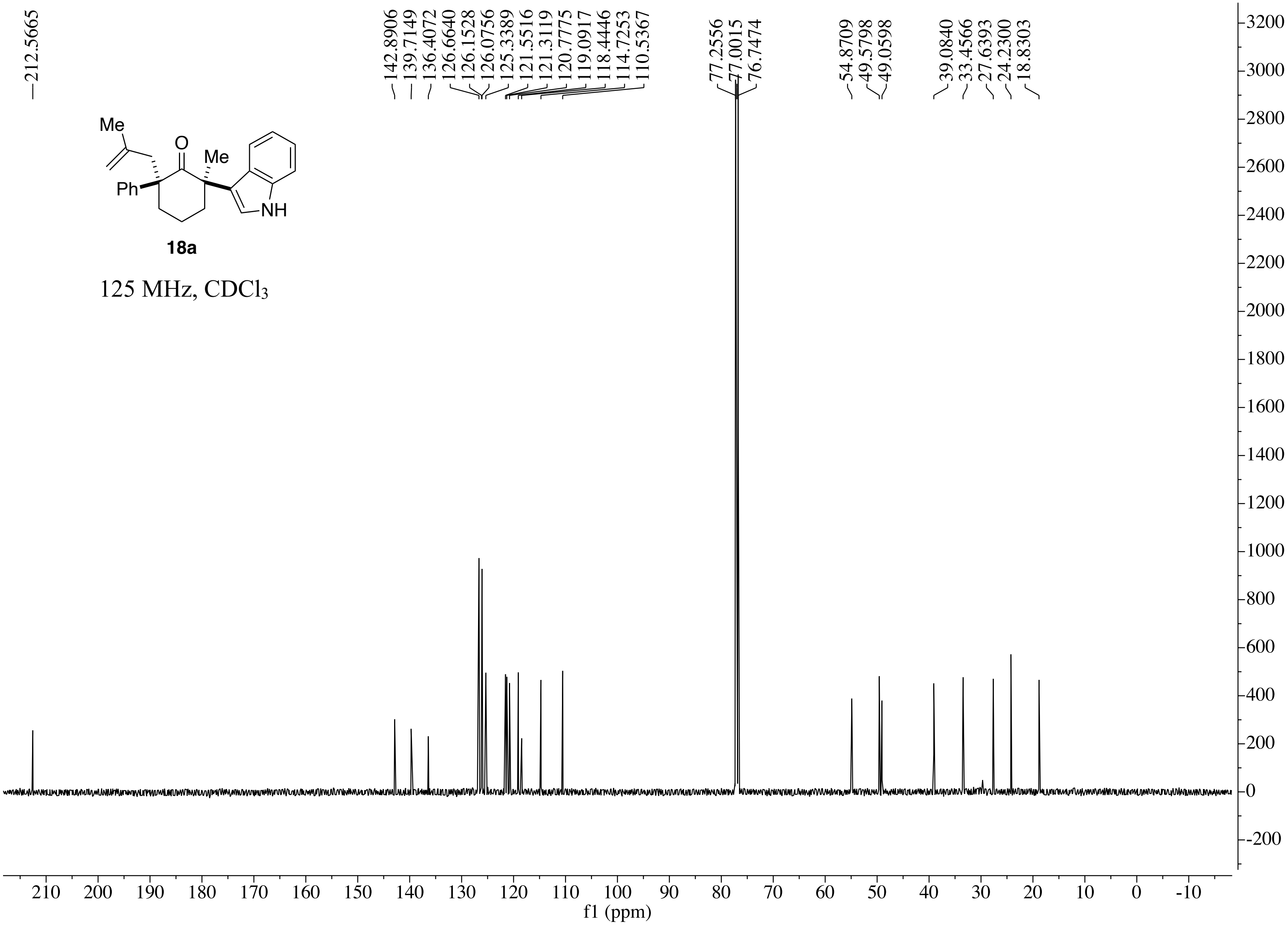

212.5665
142.8906
139.7149
136.4072
126.6640
126.1528
126.0756
125.3389
121.5516
121.3119
120.7775
119.0917
118.4446
114.7253
110.5367
77.2556
77.0015
76.7474
54.8709
49.5798
49.0598
39.0840
33.4566
27.6393
24.2300
18.8303
Me
O
Me
Ph
NH
18a
125 MHz, $CDCl_3$
f1 (ppm)

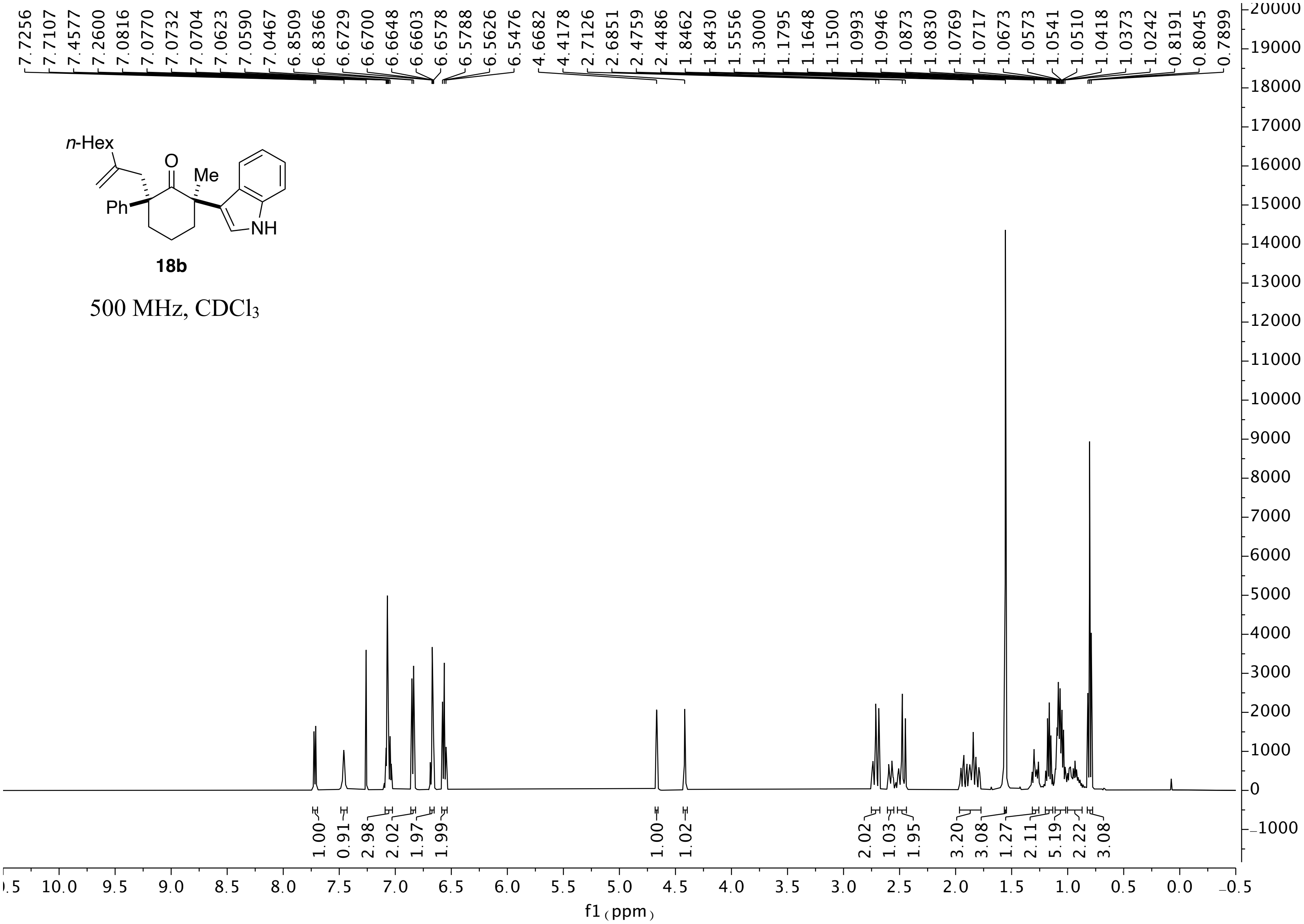
n-Hex
O
Me
Ph
NH
18b
500 MHz, CDCl3
7.7256
7.7107
7.4577
7.2600
7.0816
7.0770
7.0732
7.0704
7.0623
7.0590
7.0467
6.8509
6.8366
6.6729
6.6700
6.6648
6.6603
6.6578
6.5788
6.5626
6.5476
4.6682
4.4178
2.7126
2.6851
2.4759
2.4486
1.8462
1.8430
1.5556
1.3000
1.1795
1.1648
1.1500
1.0993
1.0946
1.0873
1.0830
1.0769
1.0717
1.0673
1.0573
1.0541
1.0510
1.0418
1.0373
1.0242
0.8191
0.8045
0.7899
1.00
0.91
2.98
2.02
1.97
1.99
1.00
1.02
2.02
1.03
1.95
3.20
3.08
1.27
2.11
5.19
2.22
3.08
f1 (ppm)

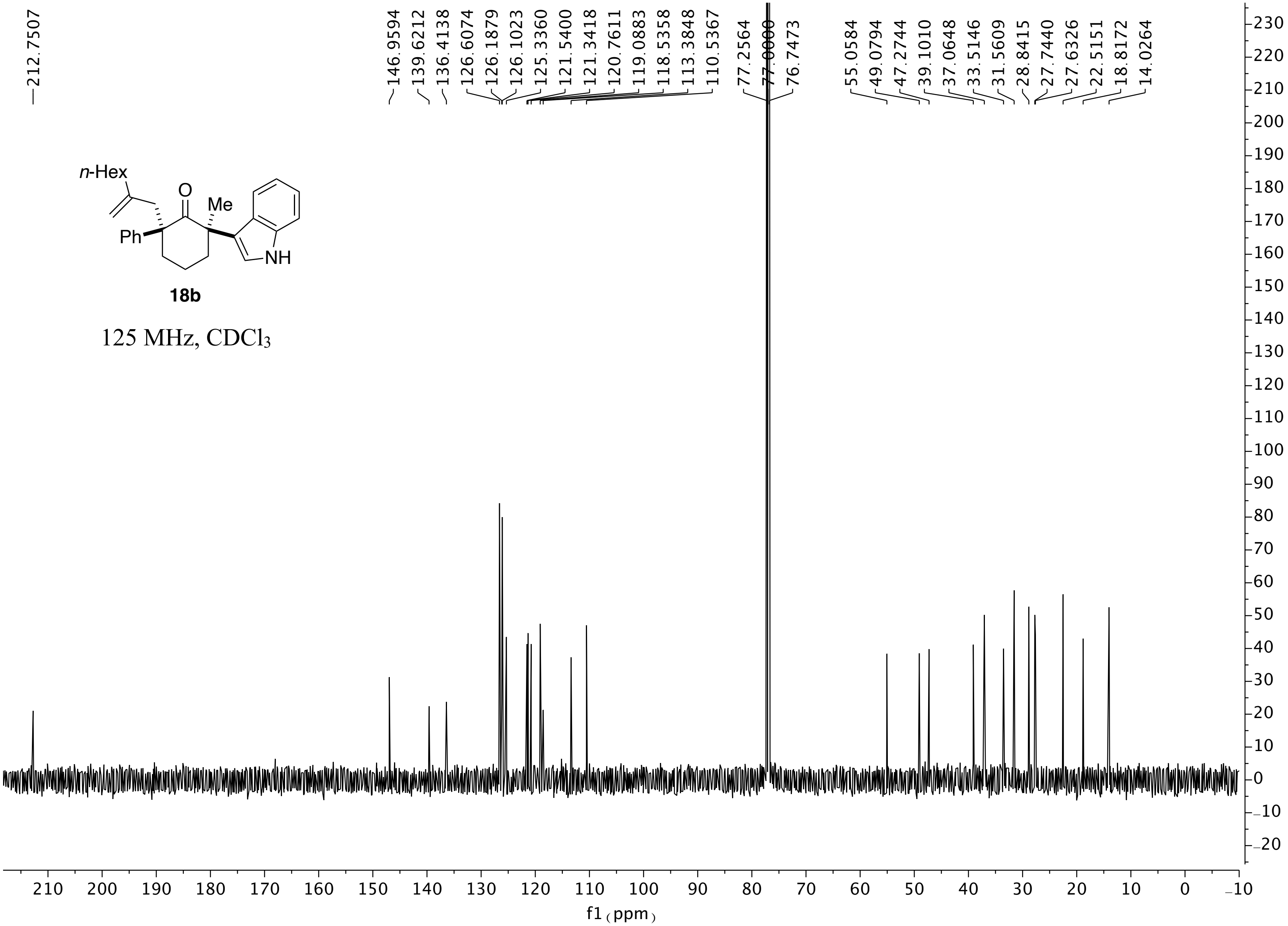
212.7507
146.9594
139.6212
136.4138
126.6074
126.1879
126.1023
125.3360
121.5400
121.3418
120.7611
119.0883
118.5358
113.3848
110.5367
77.2564
77.0000
76.7473
55.0584
49.0794
47.2744
39.1010
37.0648
33.5146
31.5609
28.8415
27.7440
27.6326
22.5151
18.8172
14.0264
n-Hex
O
Me
Ph
NH
18b
125 MHz, CDCl3
f1 (ppm)

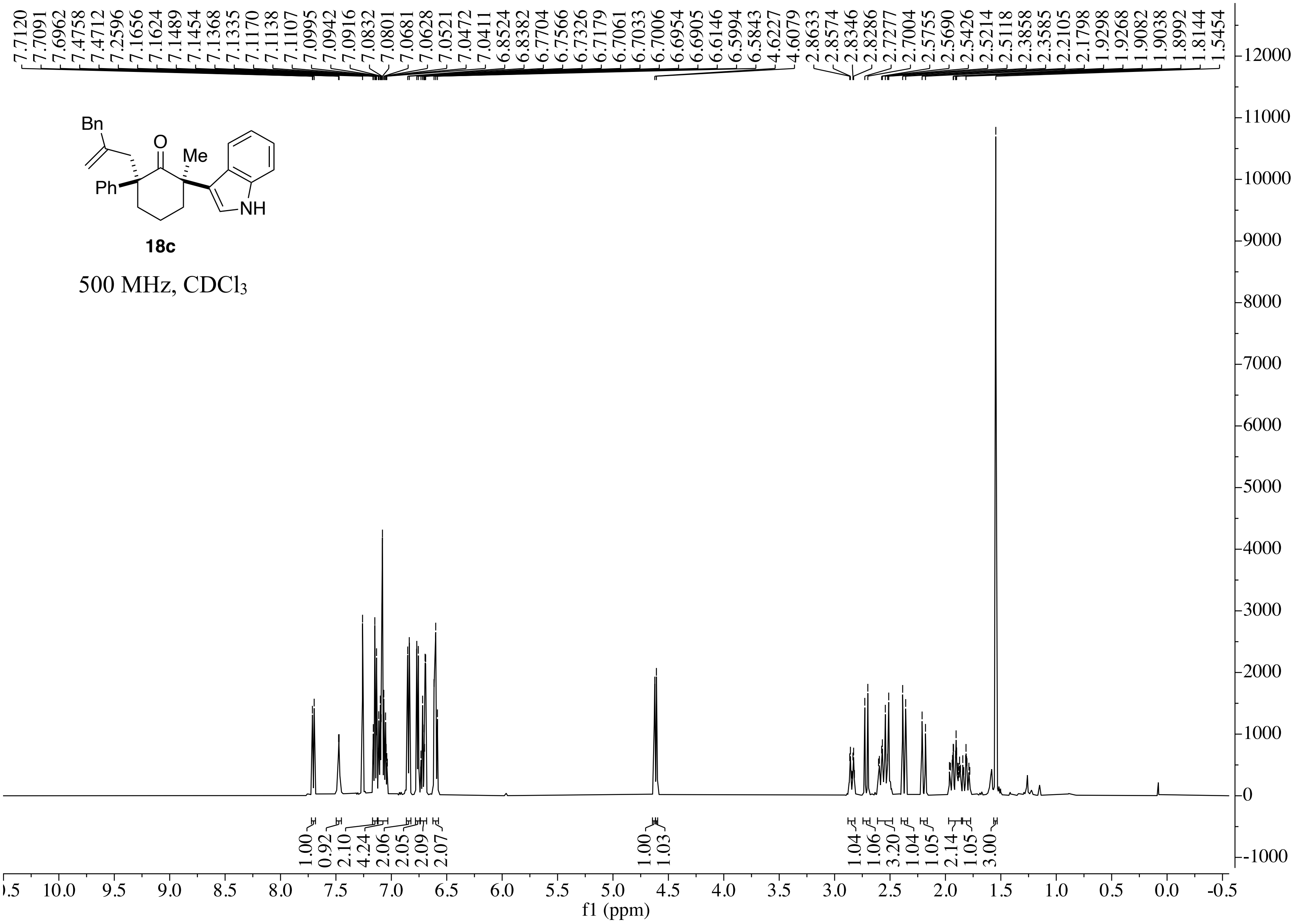

Bn
O
Me
Ph
NH
18c
500 MHz, CDCl3
f1 (ppm)

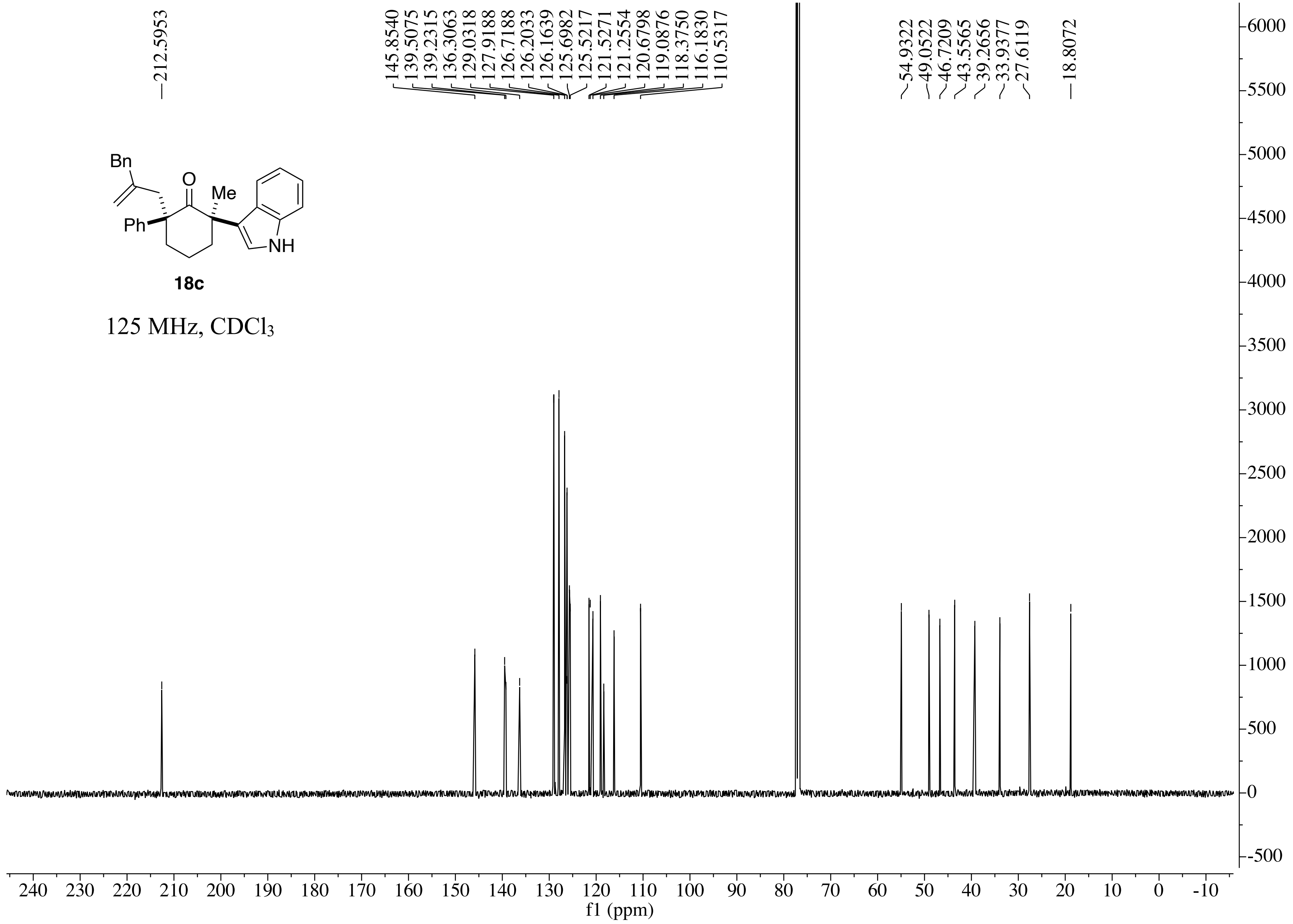
212.5953
145.8540
139.5075
139.2315
136.3063
129.0318
127.9188
126.7188
126.2033
126.1639
125.6982
125.5217
121.5271
121.2554
120.6798
119.0876
118.3750
116.1830
110.5317
54.9322
49.0522
46.7209
43.5565
39.2656
33.9377
27.6119
18.8072
Bn
O
Me
Ph
NH
18c
125 MHz, CDCl3
f1 (ppm)

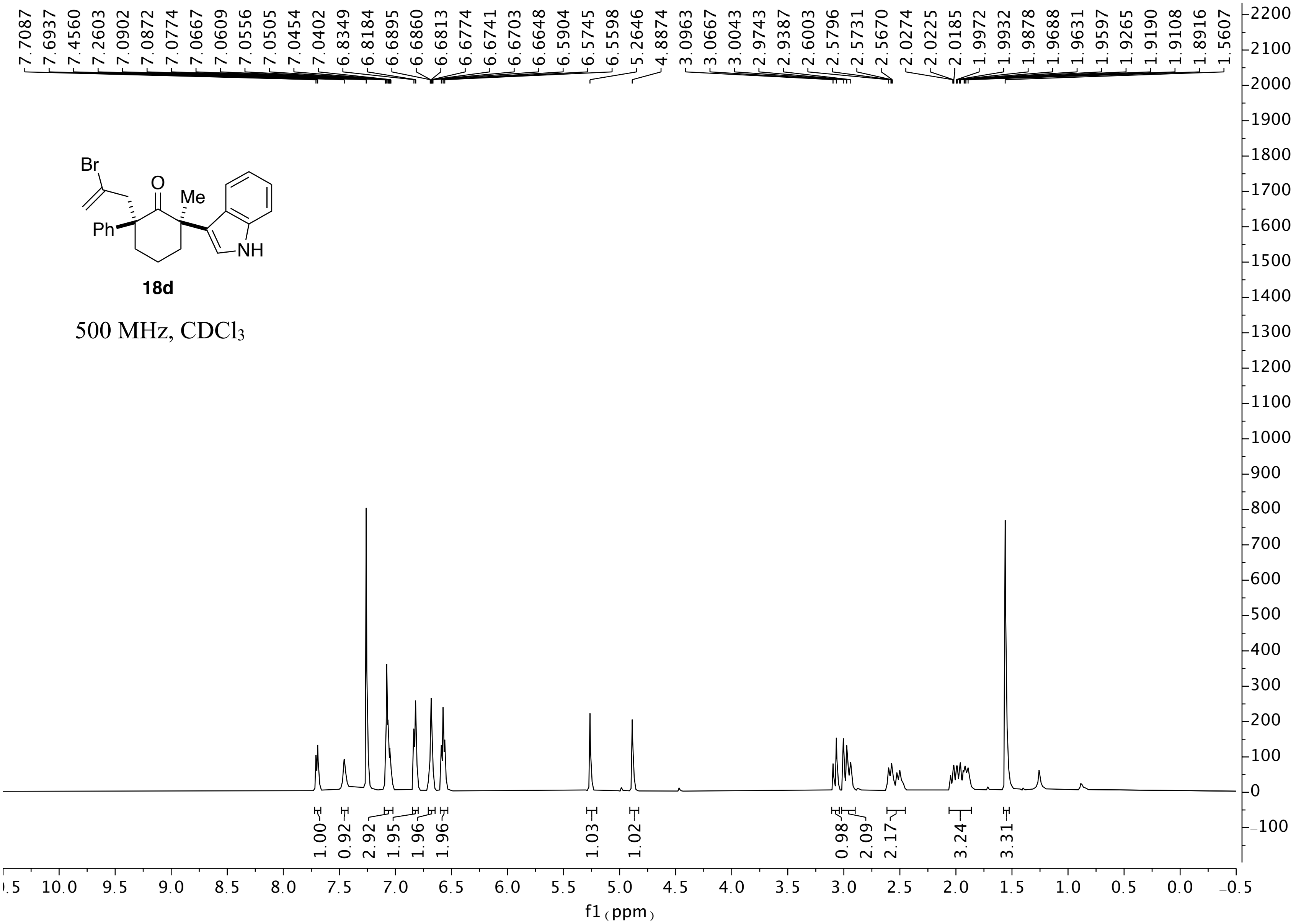

18d

500 MHz, $CDCl_3$

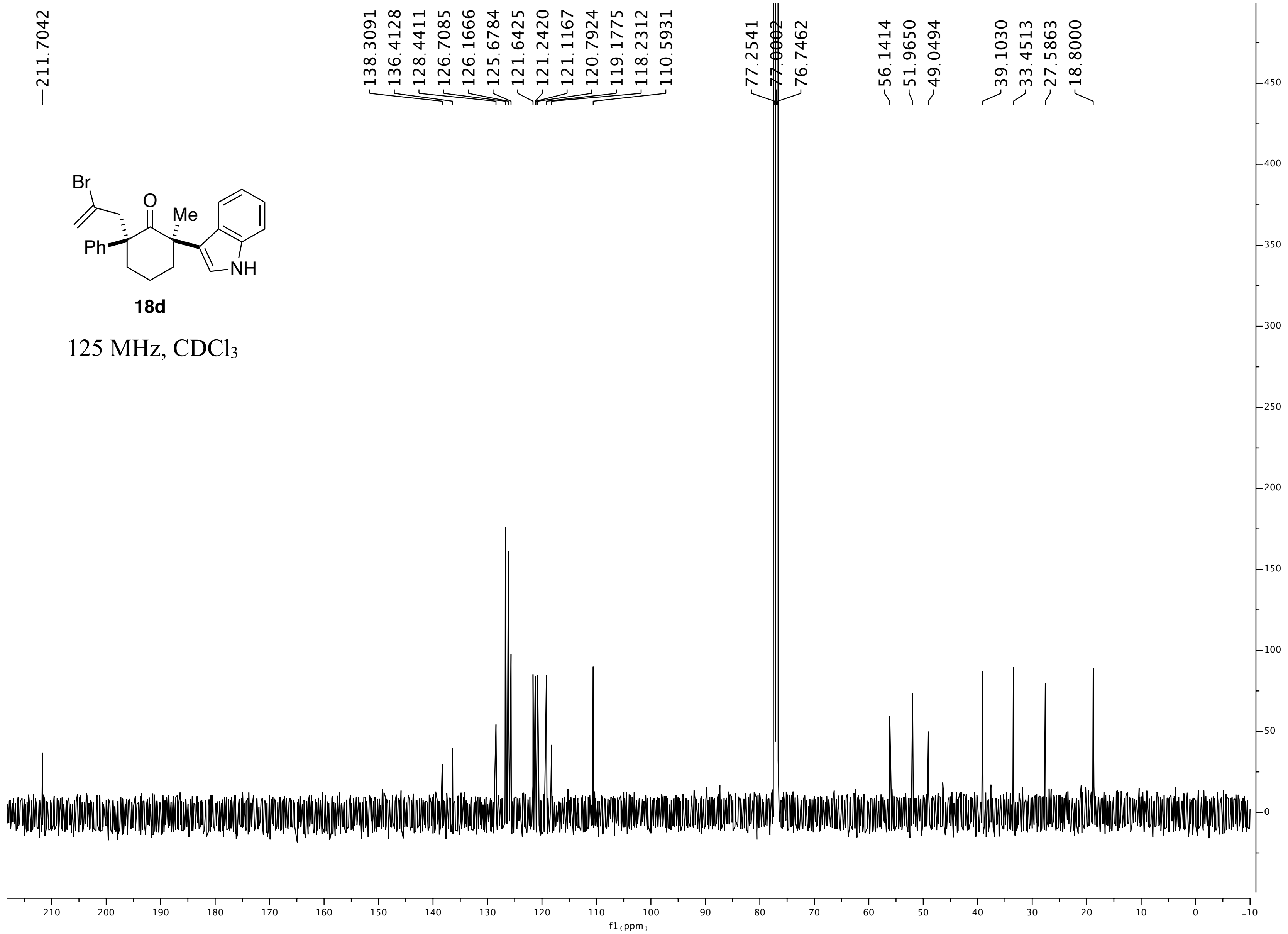
211.7042
138.3091
136.4128
128.4411
126.7085
126.1666
125.6784
121.6425
121.2420
121.1167
120.7924
119.1775
118.2312
110.5931
77.2541
77.0002
76.7462
56.1414
51.9650
49.0494
39.1030
33.4513
27.5863
18.8000
Br
O
Me
Ph
NH
18d
125 MHz, CDCl3
f1 (ppm)

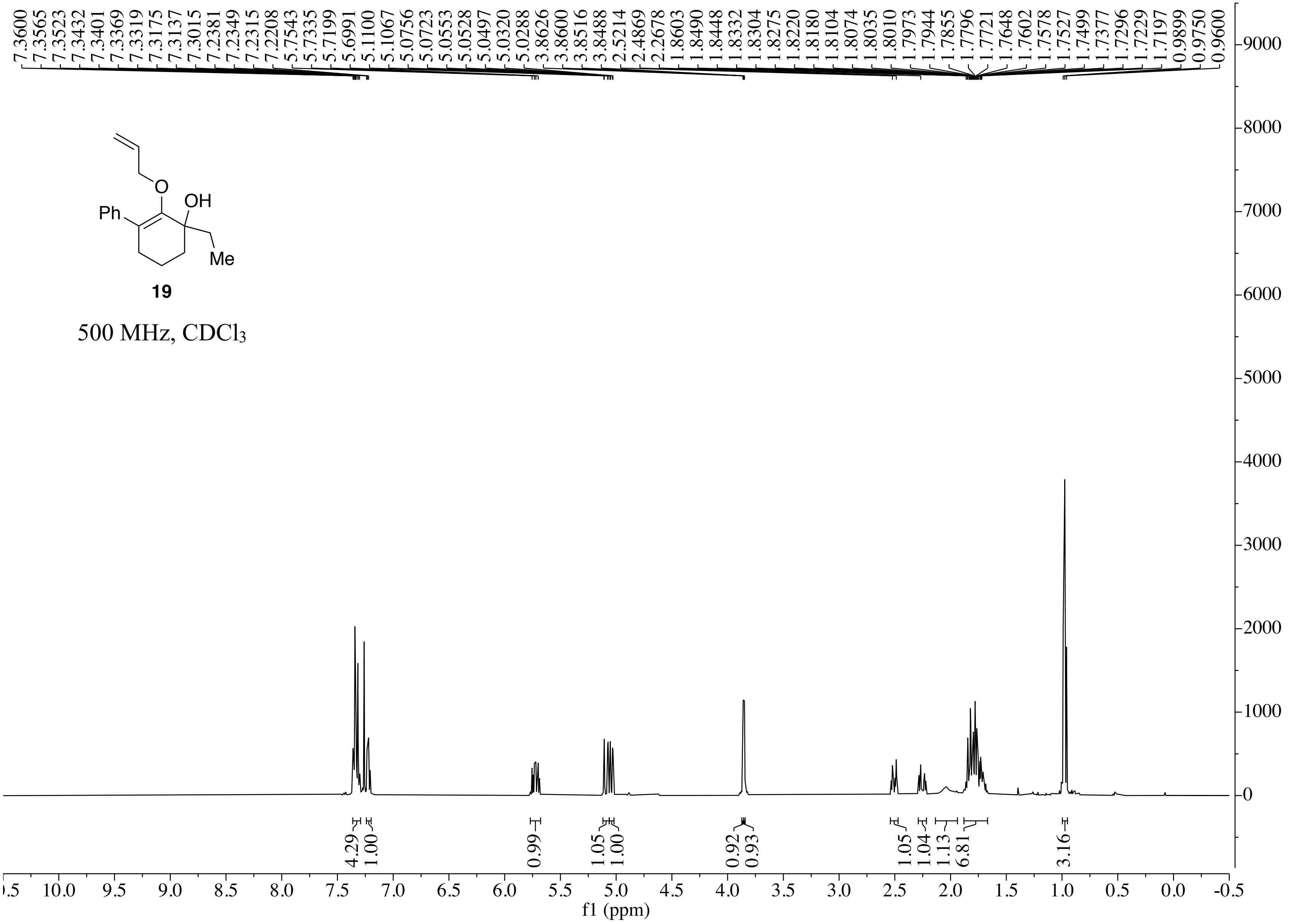

Ph
OH
O
Me
19
500 MHz, $CDCl_3$
f1 (ppm)

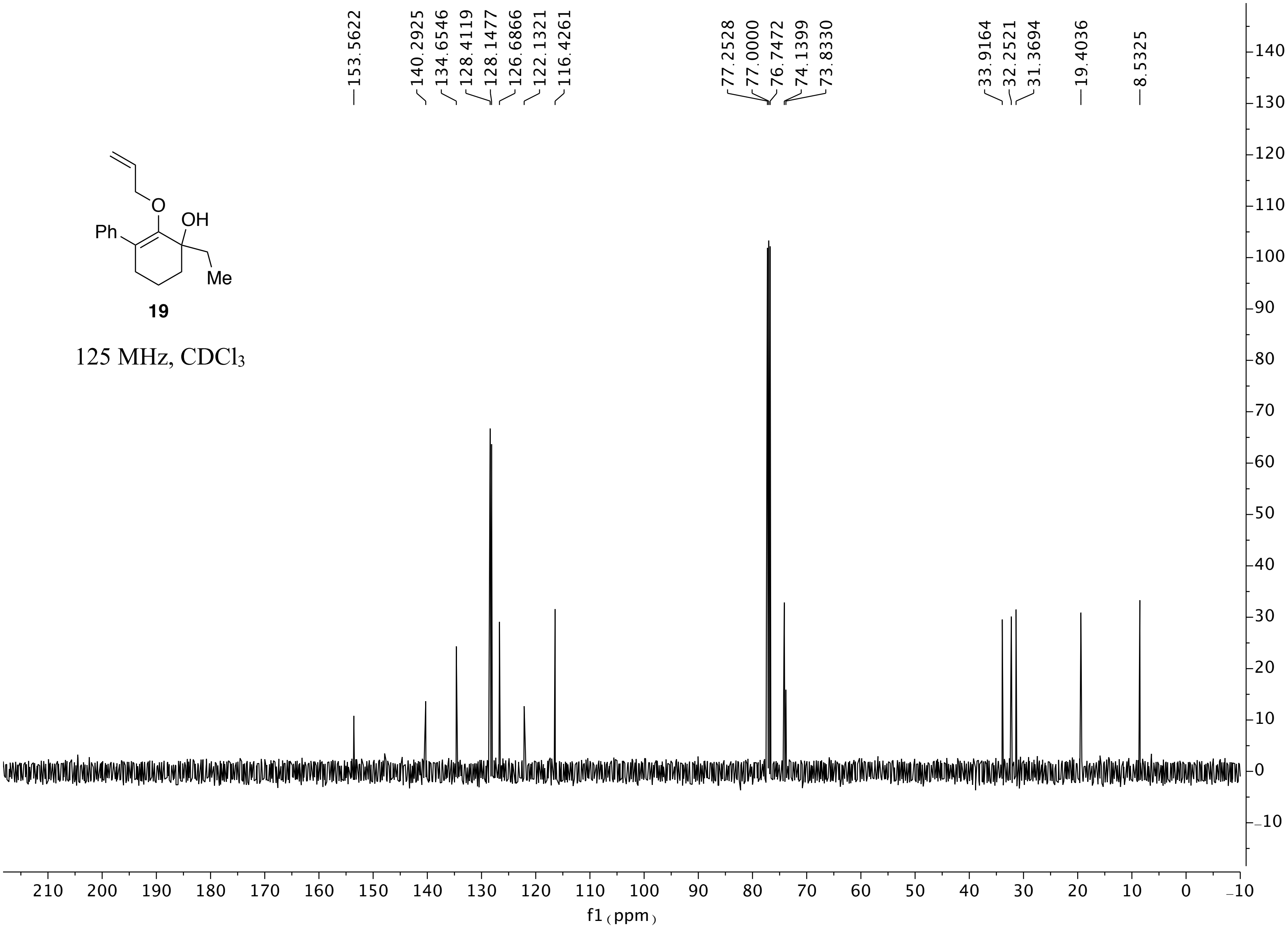

19

125 MHz, $CDCl_3$

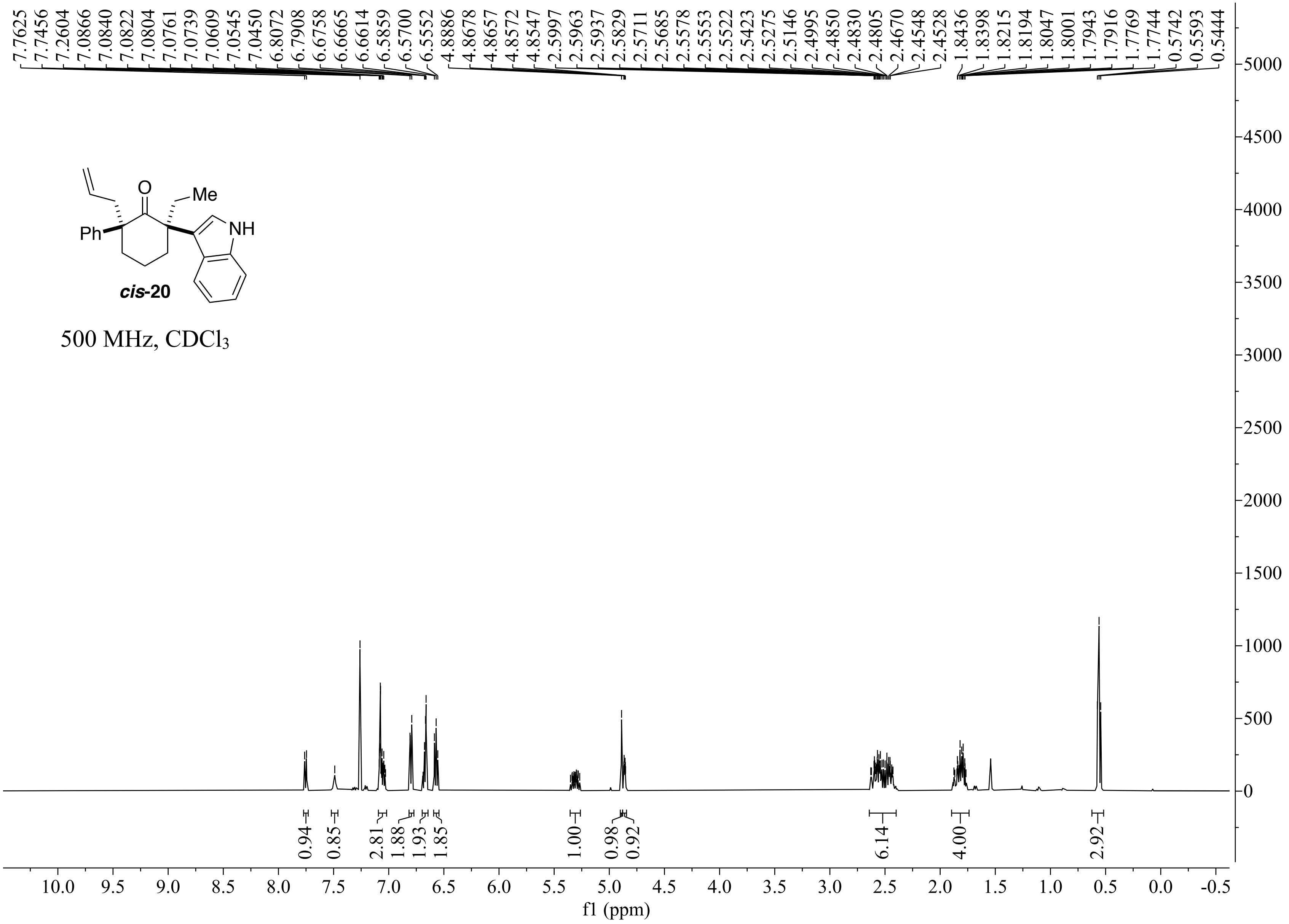

***cis*-20**

500 MHz, $CDCl_3$

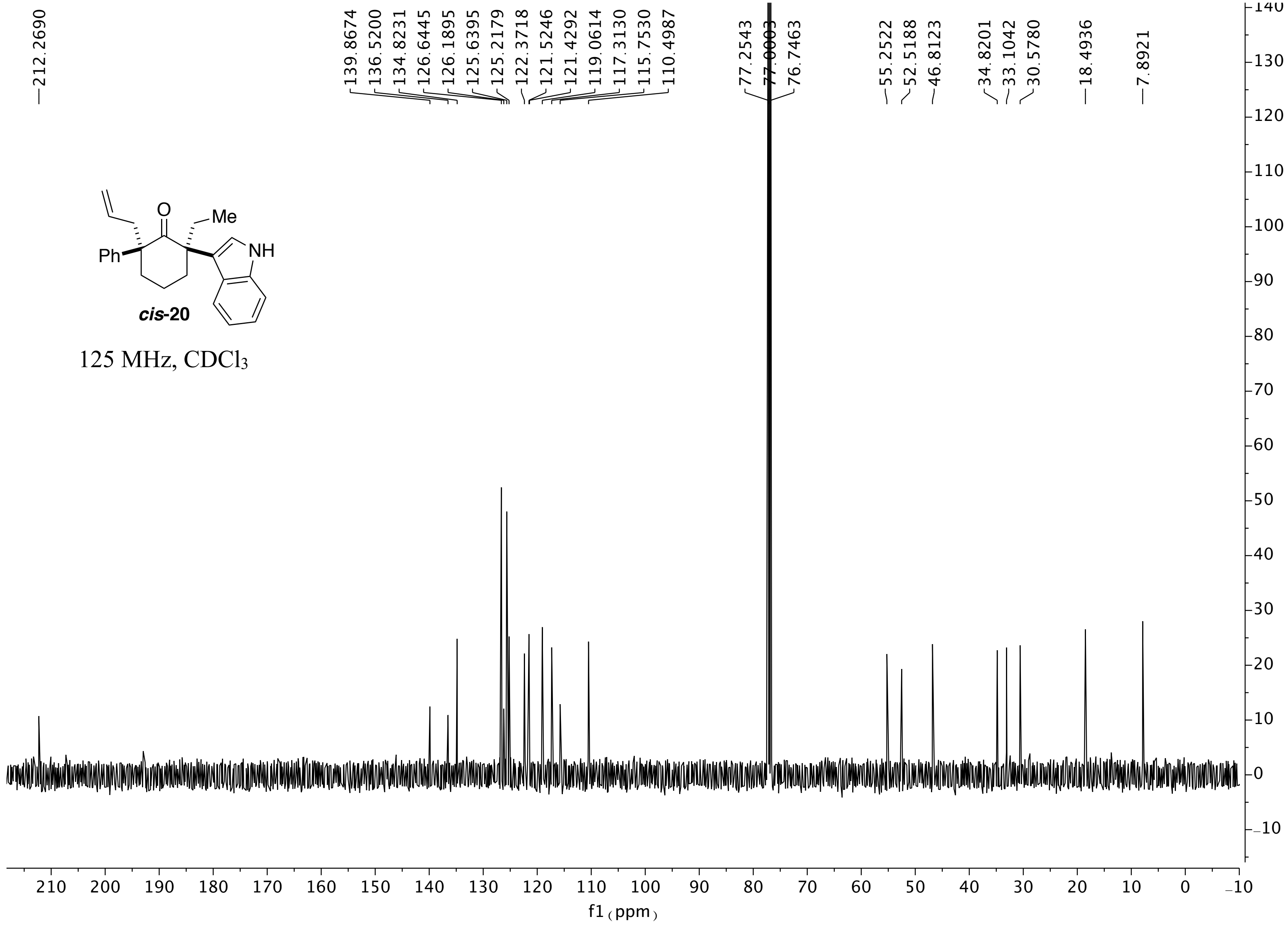
212.2690
139.8674
136.5200
134.8231
126.6445
126.1895
125.6395
125.2179
122.3718
121.5246
121.4292
119.0614
117.3130
115.7530
110.4987
77.2543
77.0003
76.7463
55.2522
52.5188
46.8123
34.8201
33.1042
30.5780
18.4936
7.8921
O
Me
Ph
NH
cis-20
125 MHz, CDCl3
f1 (ppm)

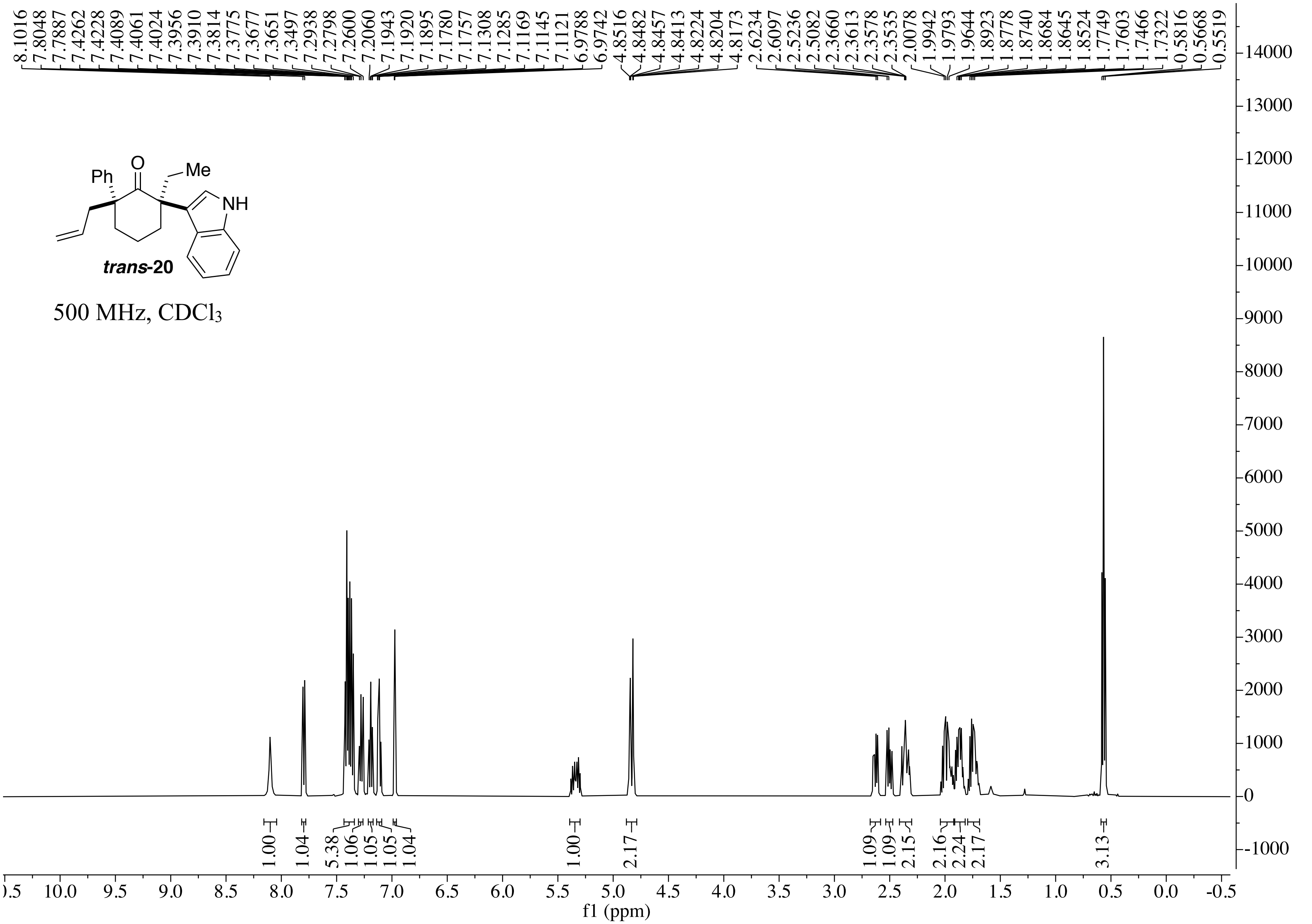

Ph
O
Me
NH
trans-20
500 MHz, $CDCl_3$
8.1016
7.8048
7.7887
7.4262
7.4228
7.4089
7.4061
7.4024
7.3956
7.3910
7.3814
7.3775
7.3677
7.3651
7.3497
7.2938
7.2798
7.2600
7.2060
7.1943
7.1920
7.1895
7.1780
7.1757
7.1308
7.1285
7.1169
7.1145
7.1121
6.9788
6.9742
4.8516
4.8482
4.8457
4.8413
4.8224
4.8204
4.8173
2.6234
2.6097
2.5236
2.5082
2.3660
2.3613
2.3578
2.3535
2.0078
1.9942
1.9793
1.9644
1.8923
1.8778
1.8740
1.8684
1.8645
1.8524
1.7749
1.7603
1.7466
1.7322
0.5816
0.5668
0.5519
1.00
1.04
5.38
1.06
1.05
1.05
1.04
1.00
2.17
1.09
1.09
2.15
2.16
2.24
2.17
3.13
f1 (ppm)

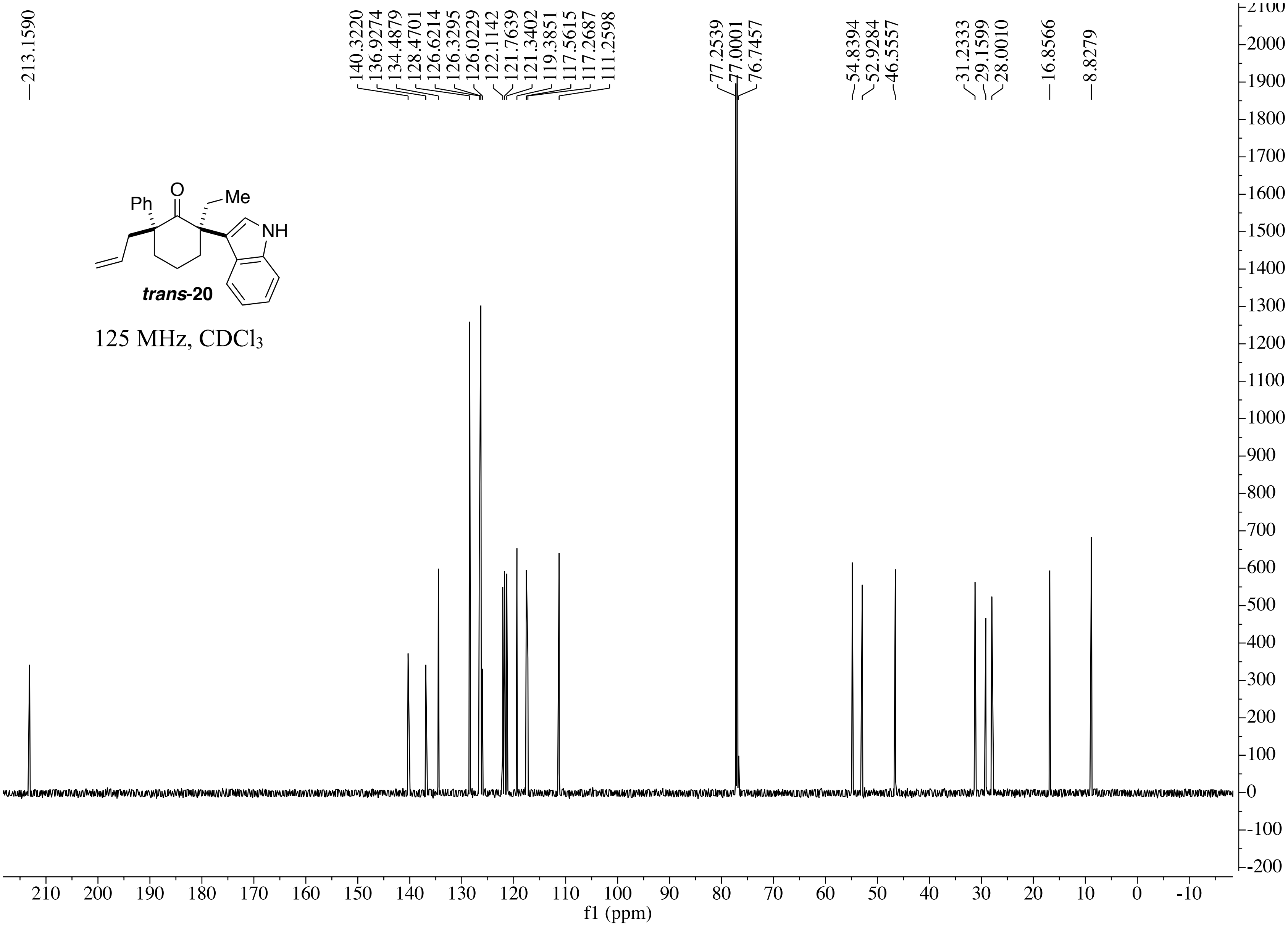

213.1590
140.3220
136.9274
134.4879
128.4701
126.6214
126.3295
126.0229
122.1142
121.7639
121.3402
119.3851
117.5615
117.2687
111.2598
77.2539
77.0001
76.7457
54.8394
52.9284
46.5557
31.2333
29.1599
28.0010
16.8566
8.8279
Ph
O
Me
NH
trans-20
125 MHz, CDCl3
f1 (ppm)

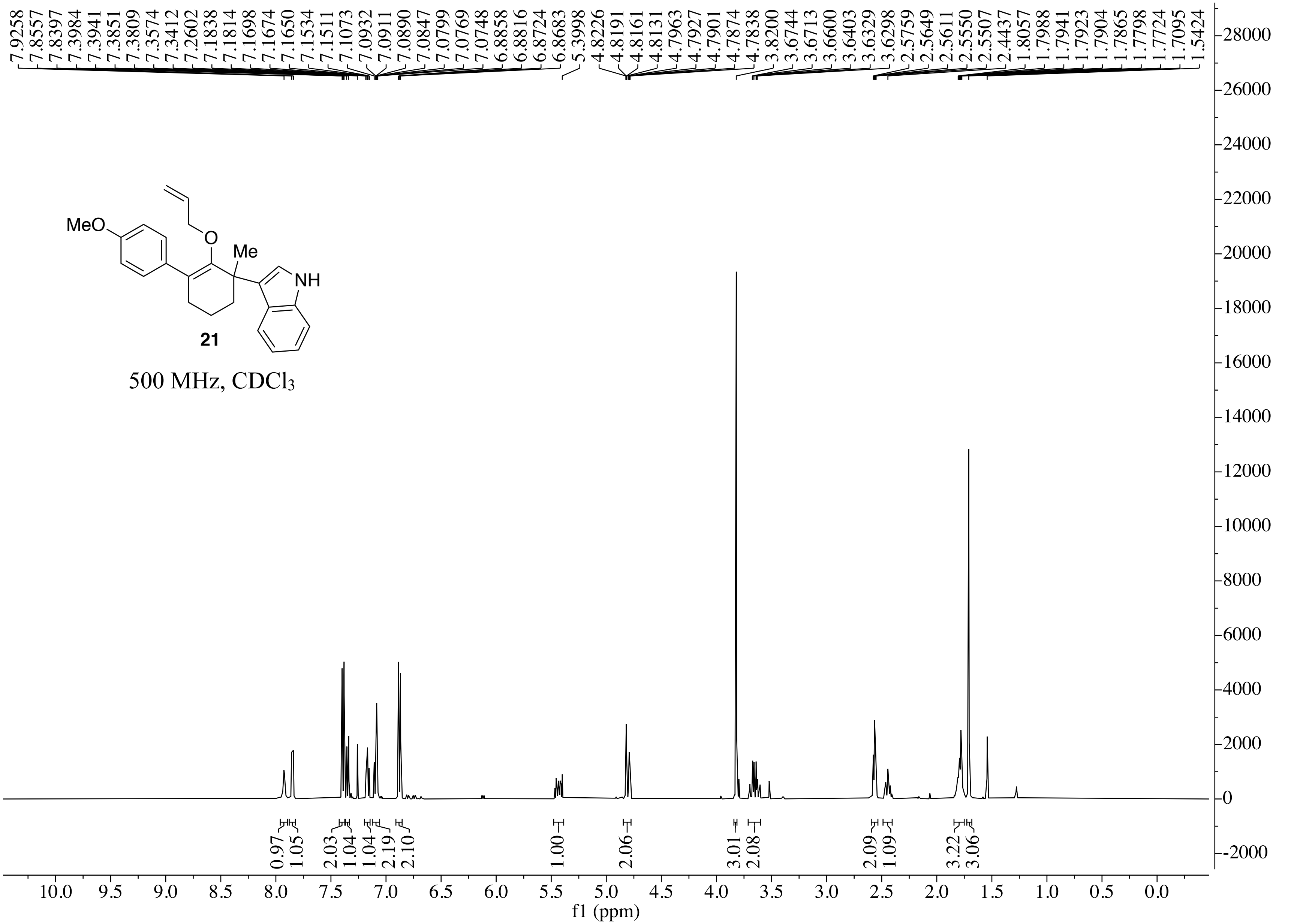
7.9258
7.8557
7.8397
7.3984
7.3941
7.3851
7.3809
7.3574
7.3412
7.2602
7.1838
7.1814
7.1698
7.1674
7.1650
7.1534
7.1511
7.1073
7.0932
7.0911
7.0890
7.0847
7.0799
7.0769
7.0748
6.8858
6.8816
6.8724
6.8683
5.3998
4.8226
4.8191
4.8161
4.8131
4.7963
4.7927
4.7901
4.7874
4.7838
3.8200
3.6744
3.6713
3.6600
3.6403
3.6329
3.6298
2.5759
2.5649
2.5611
2.5550
2.5507
2.4437
1.8057
1.7988
1.7941
1.7923
1.7904
1.7865
1.7798
1.7724
1.7095
1.5424
MeO
O
Me
NH
21
500 MHz, CDCl3
0.97
1.05
2.03
1.04
1.04
2.19
2.10
1.00
2.06
3.01
2.08
2.09
1.09
3.22
3.06
28000
26000
24000
22000
20000
18000
16000
14000
12000
10000
8000
6000
4000
2000
0
-2000
10.0
9.5
9.0
8.5
8.0
7.5
7.0
6.5
6.0
5.5
5.0
4.5
4.0
3.5
3.0
2.5
2.0
1.5
1.0
0.5
0.0
f1 (ppm)

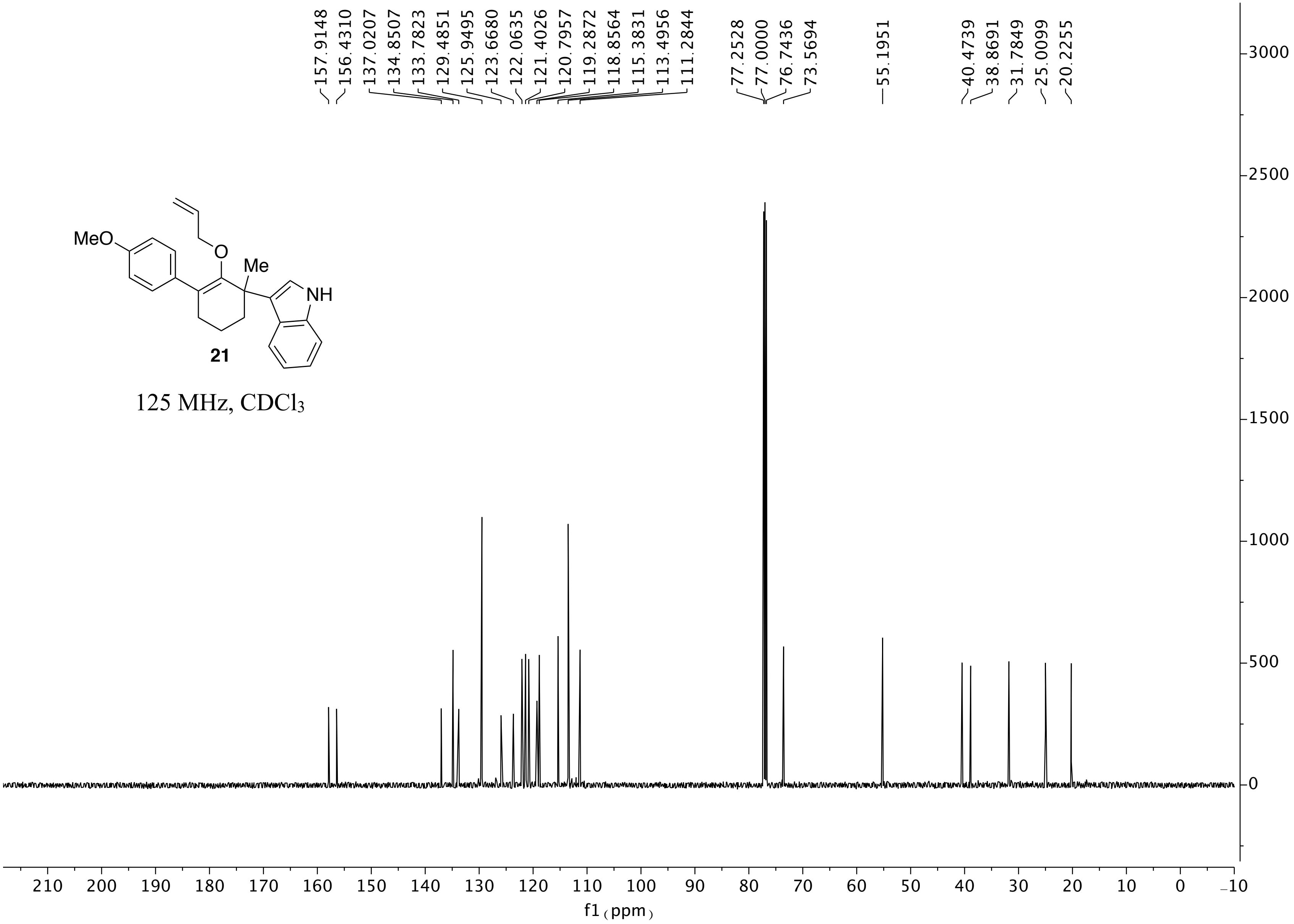

21

125 MHz, $CDCl_3$

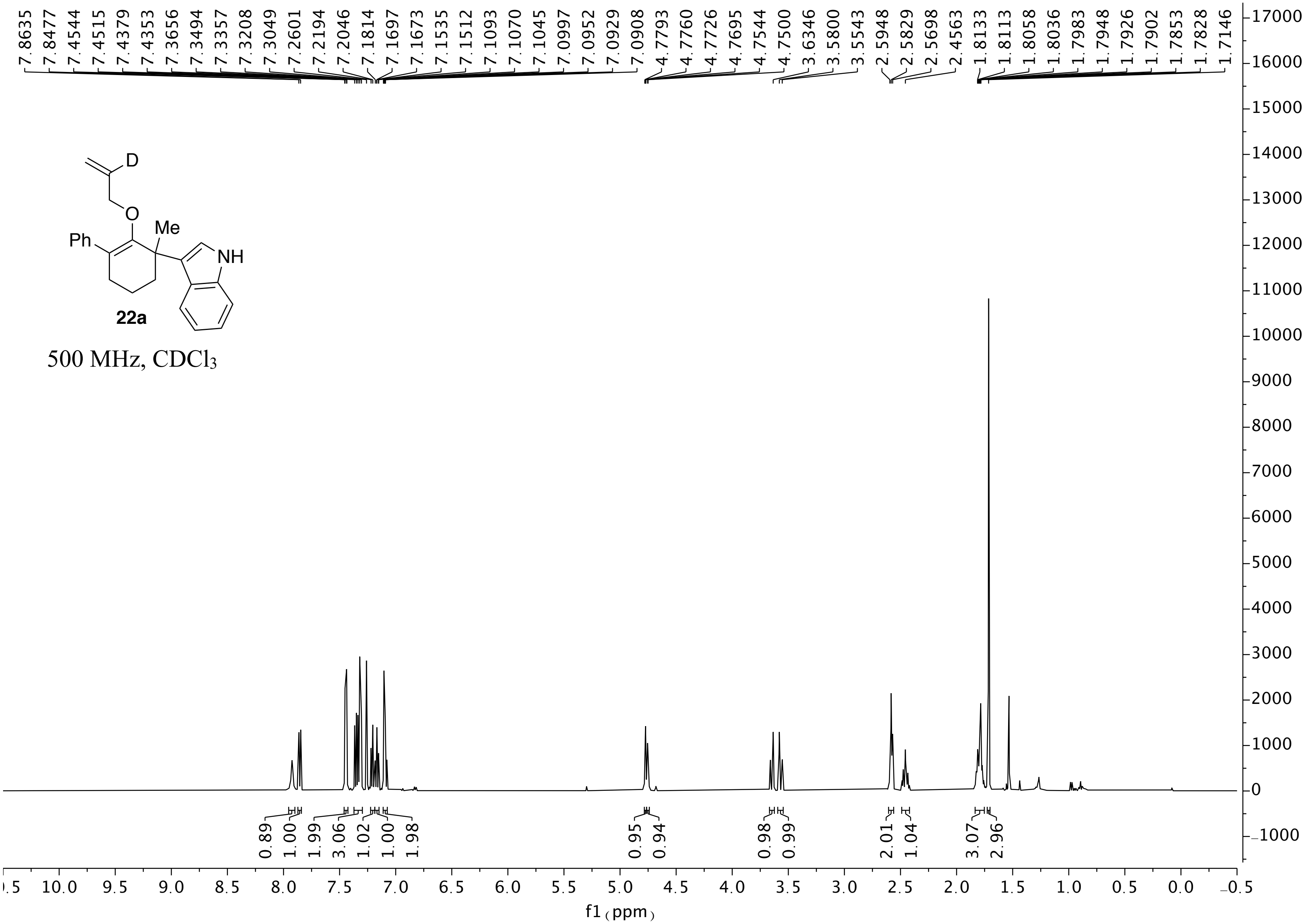
7.8635
7.8477
7.4544
7.4515
7.4379
7.4353
7.3656
7.3494
7.3357
7.3208
7.3049
7.2601
7.2194
7.2046
7.1814
7.1697
7.1673
7.1535
7.1512
7.1093
7.1070
7.1045
7.0997
7.0952
7.0929
7.0908
4.7793
4.7760
4.7726
4.7695
4.7544
4.7500
3.6346
3.5800
3.5543
2.5948
2.5829
2.5698
2.4563
1.8133
1.8113
1.8058
1.8036
1.7983
1.7948
1.7926
1.7902
1.7853
1.7828
1.7146
D
O
Me
Ph
NH
22a
500 MHz, CDCl3
0.89
1.00
1.99
3.06
1.02
1.00
1.98
0.95
0.94
0.98
0.99
2.01
1.04
3.07
2.96
f1 (ppm)

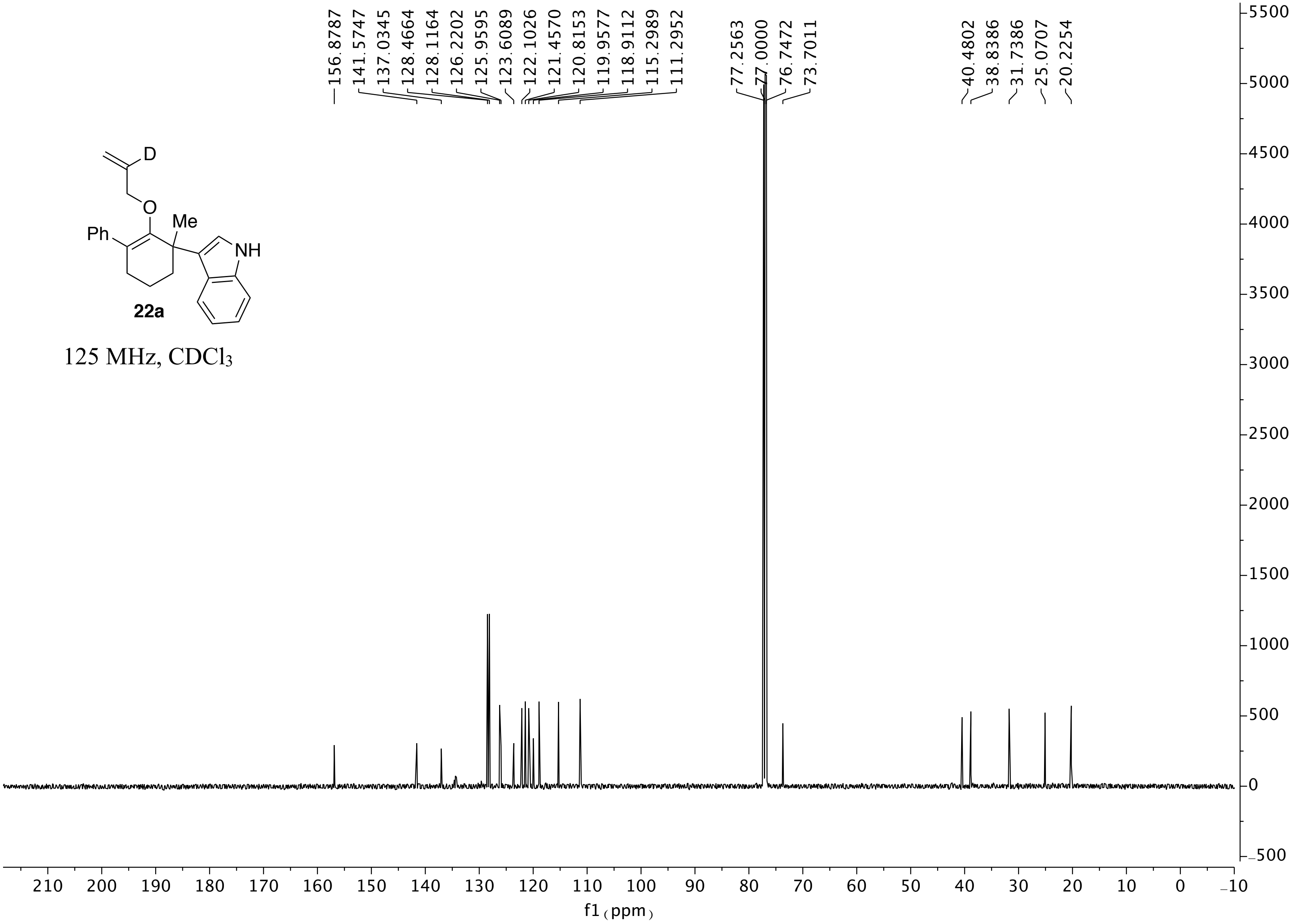

156.8787
141.5747
137.0345
128.4664
128.1164
126.2202
125.9595
123.6089
122.1026
121.4570
120.8153
119.9577
118.9112
115.2989
111.2952
77.2563
77.0000
76.7472
73.7011
40.4802
38.8386
31.7386
25.0707
20.2254
D
O
Me
Ph
NH
22a
125 MHz, CDCl3
f1 (ppm)

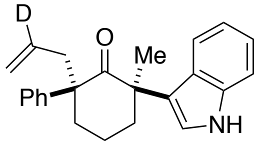

22b

500 MHz, $CDCl_3$

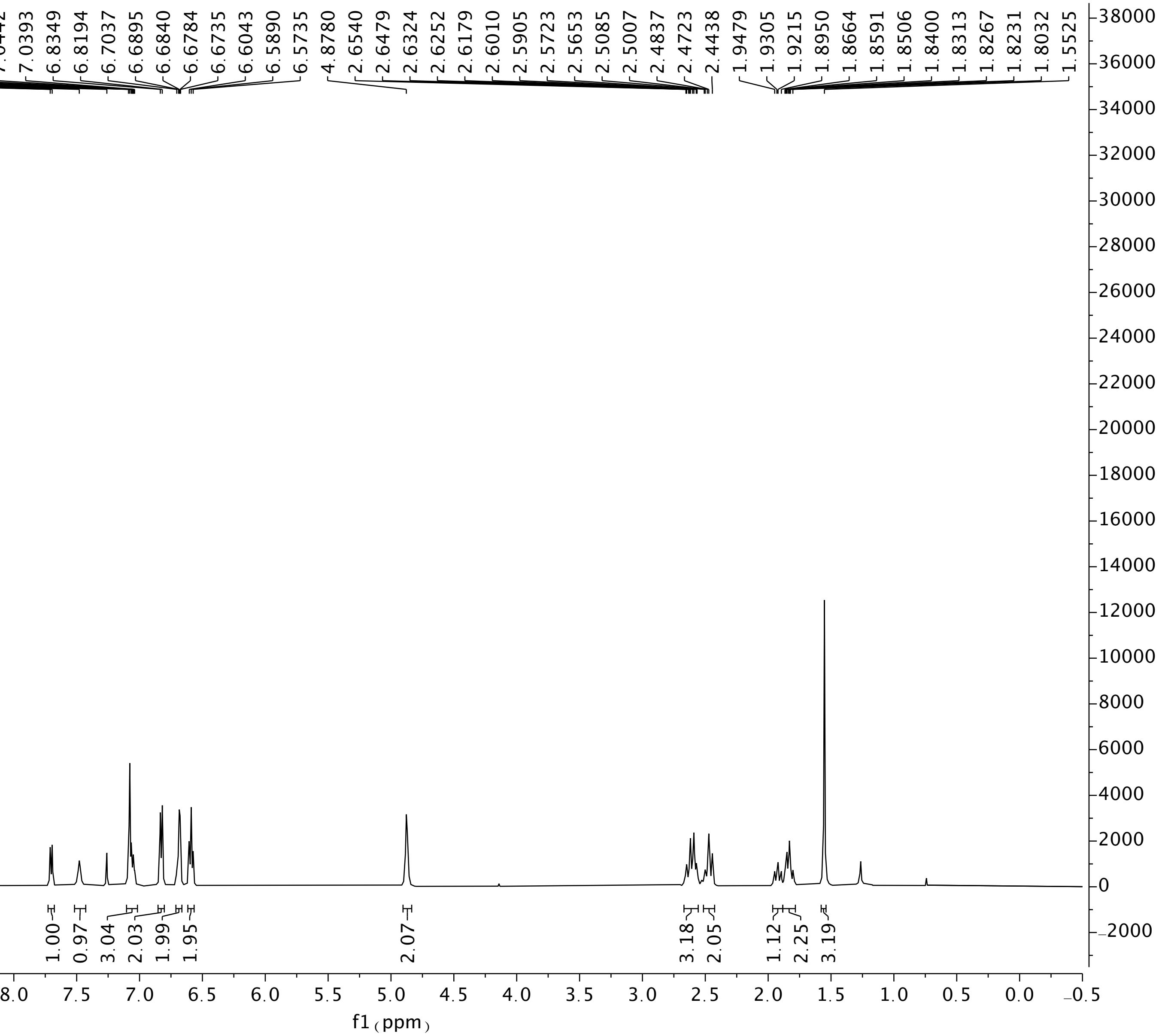

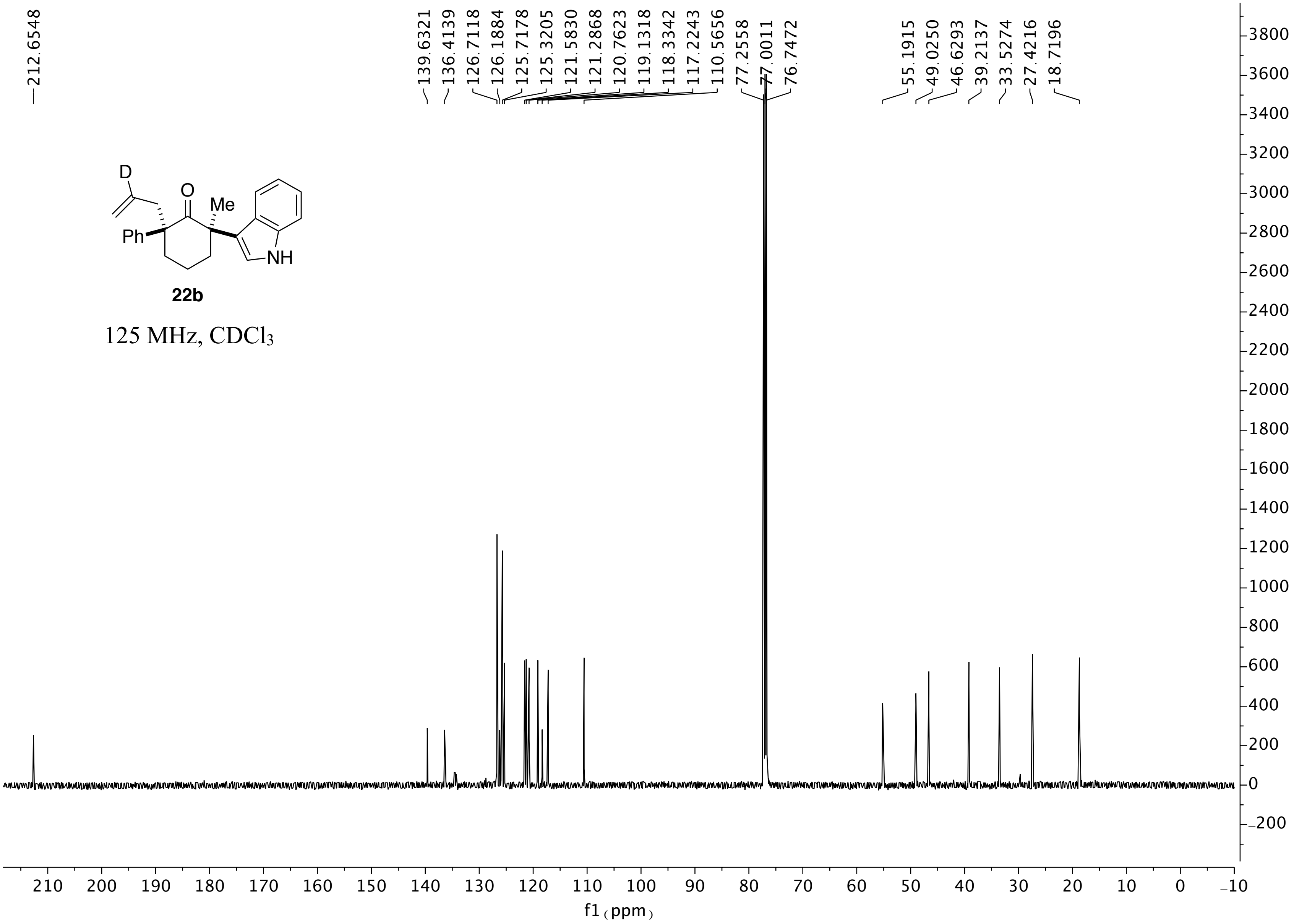
212.6548
139.6321
136.4139
126.7118
126.1884
125.7178
125.3205
121.5830
121.2868
120.7623
119.1318
118.3342
117.2243
110.5656
77.2558
77.0011
76.7472
55.1915
49.0250
46.6293
39.2137
33.5274
27.4216
18.7196
D
O
Me
Ph
NH
22b
125 MHz, CDCl3
f1 (ppm)

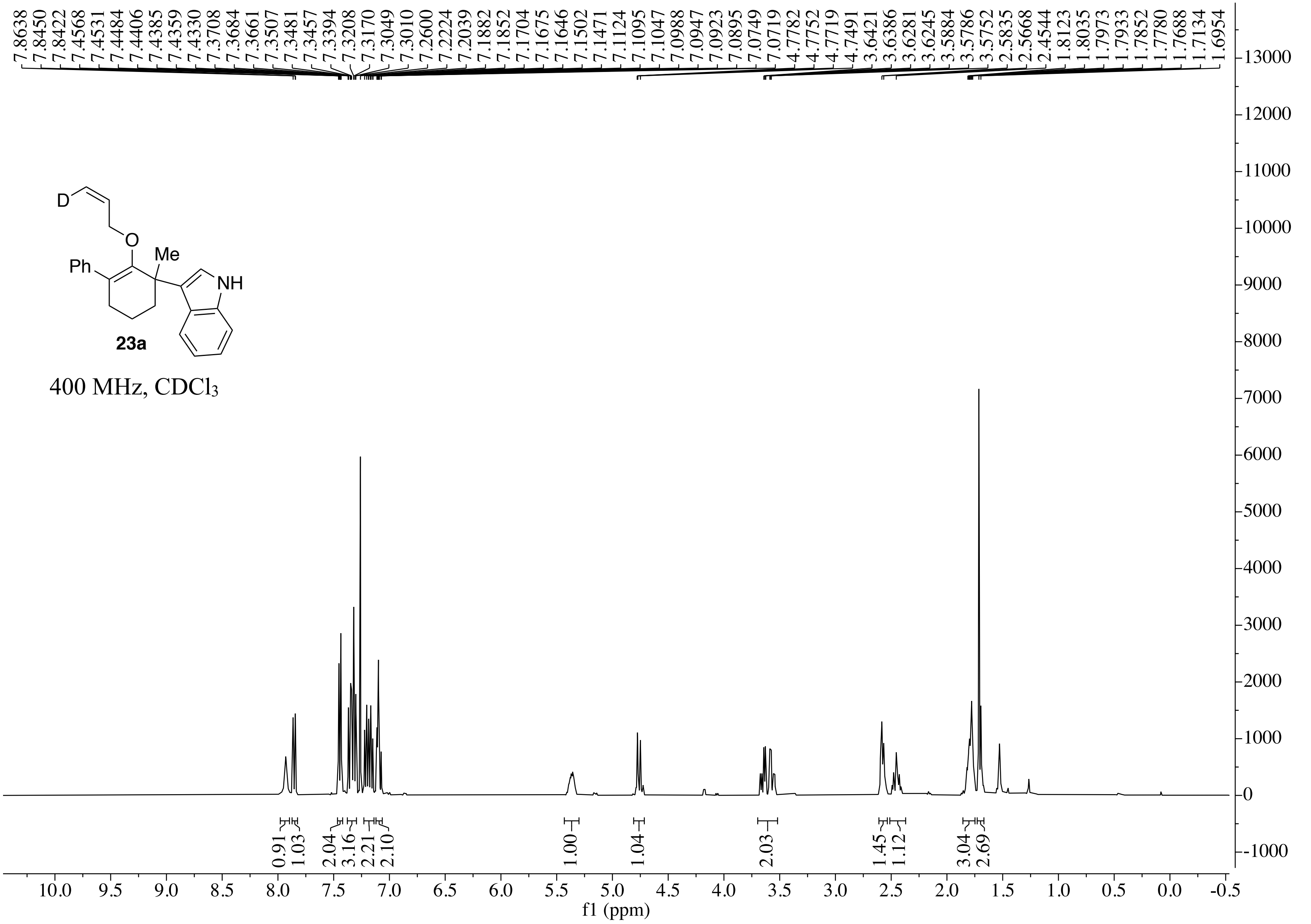
7.8638
7.8450
7.8422
7.4568
7.4531
7.4484
7.4406
7.4385
7.4359
7.4330
7.3708
7.3684
7.3661
7.3507
7.3481
7.3457
7.3394
7.3208
7.3170
7.3049
7.3010
7.2600
7.2224
7.2039
7.1882
7.1852
7.1704
7.1675
7.1646
7.1502
7.1471
7.1124
7.1095
7.1047
7.0988
7.0947
7.0923
7.0895
7.0749
7.0719
4.7782
4.7752
4.7719
4.7491
3.6421
3.6386
3.6281
3.6245
3.5884
3.5786
3.5752
2.5835
2.5668
2.4544
1.8123
1.8035
1.7973
1.7933
1.7852
1.7780
1.7688
1.7134
1.6954
D
O
Me
Ph
NH
23a
400 MHz, $CDCl_3$
0.91
1.03
2.04
3.16
2.21
2.10
1.00
1.04
2.03
1.45
1.12
3.04
2.69
f1 (ppm)

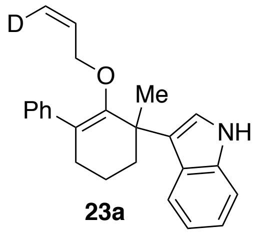

23a

125 MHz, $CDCl_3$

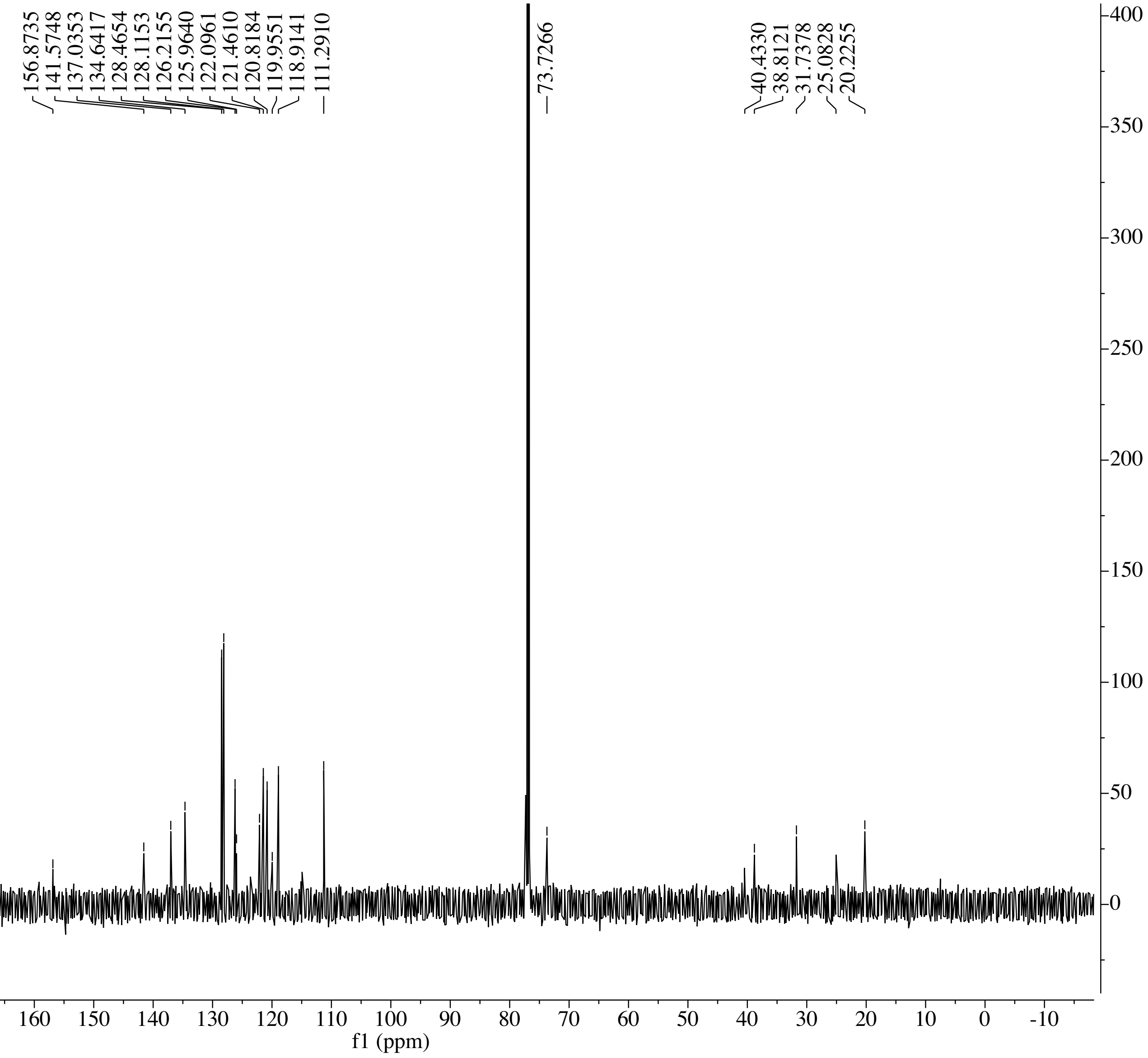

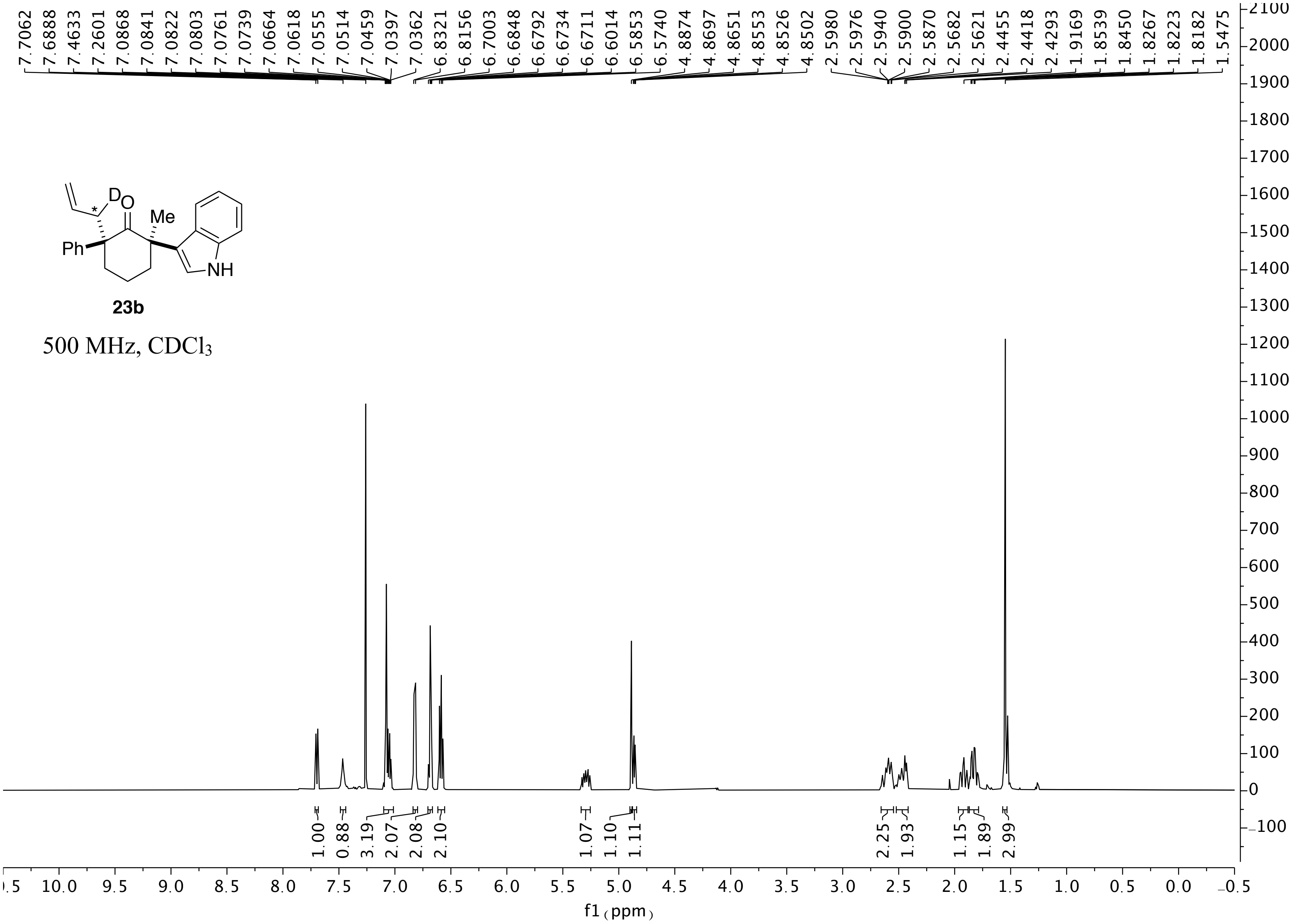
7.7062
7.6888
7.4633
7.2601
7.0868
7.0841
7.0822
7.0803
7.0761
7.0739
7.0664
7.0618
7.0555
7.0514
7.0459
7.0397
7.0362
6.8321
6.8156
6.7003
6.6848
6.6792
6.6734
6.6711
6.6014
6.5853
6.5740
4.8874
4.8697
4.8651
4.8553
4.8526
4.8502
2.5980
2.5976
2.5940
2.5900
2.5870
2.5682
2.5621
2.4455
2.4418
2.4293
1.9169
1.8539
1.8450
1.8267
1.8223
1.8182
1.5475
D
O
Me
Ph
NH
*
23b
500 MHz, CDCl3
1.00
0.88
3.19
2.07
2.08
2.10
1.07
1.10
1.11
2.25
1.93
1.15
1.89
2.99
f1 (ppm)

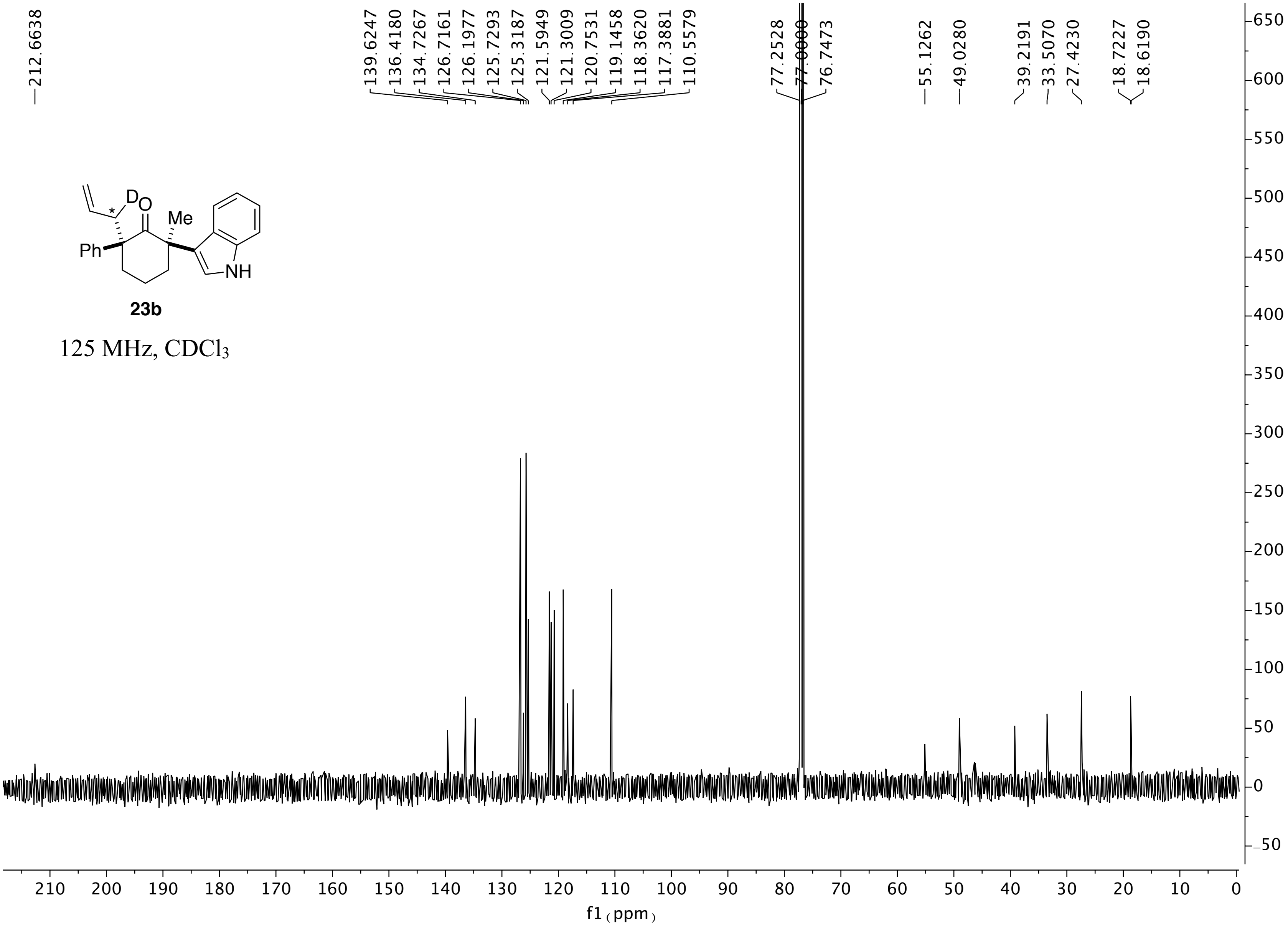

23b

125 MHz, $CDCl_3$

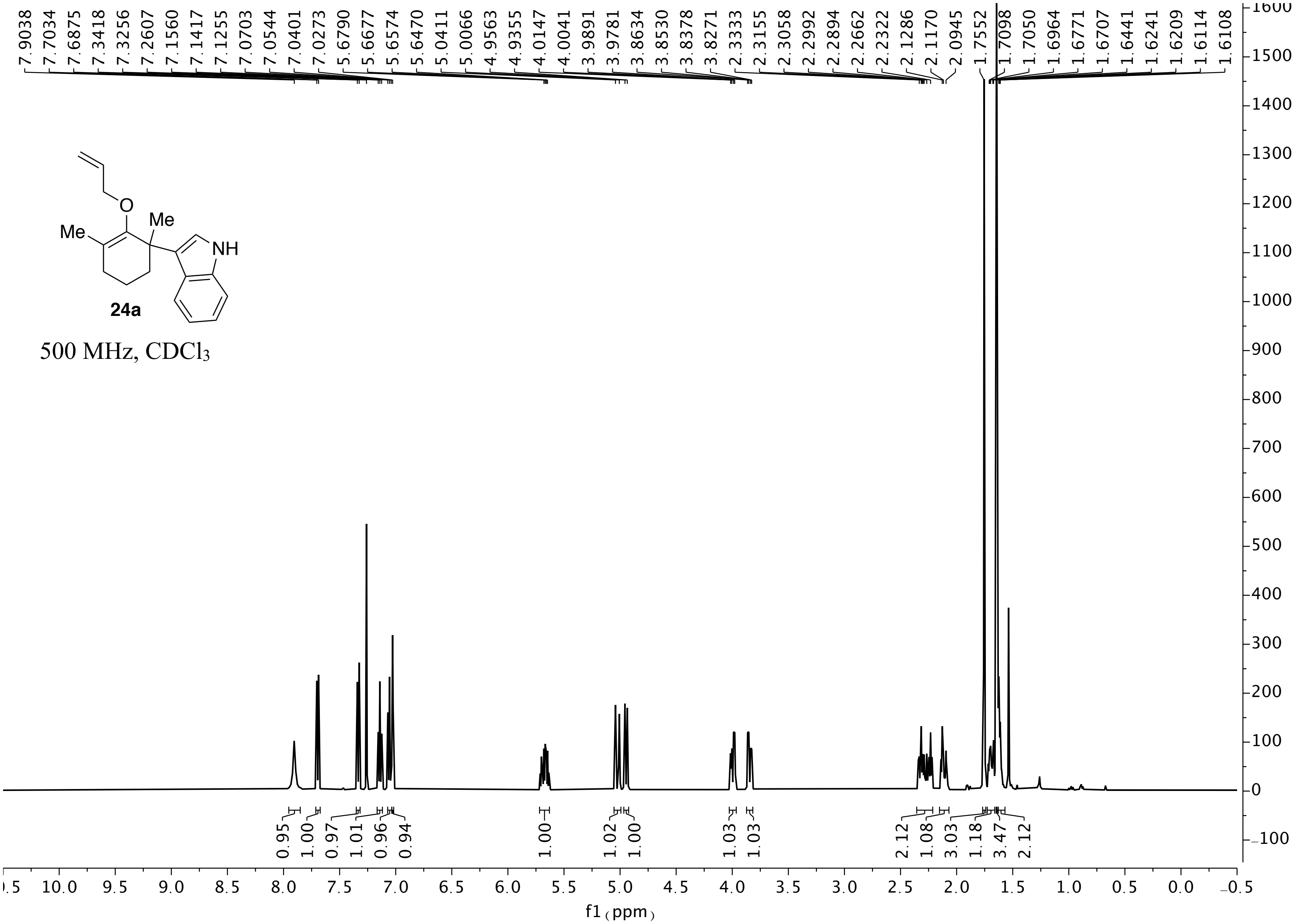
7.9038
7.7034
7.6875
7.3418
7.3256
7.2607
7.1560
7.1417
7.1255
7.0703
7.0544
7.0401
7.0273
5.6790
5.6677
5.6574
5.6470
5.0411
5.0066
4.9563
4.9355
4.0147
4.0041
3.9891
3.9781
3.8634
3.8530
3.8378
3.8271
2.3333
2.3155
2.3058
2.2992
2.2894
2.2662
2.2322
2.1286
2.1170
2.0945
1.7552
1.7098
1.7050
1.6964
1.6771
1.6707
1.6441
1.6241
1.6209
1.6114
1.6108
O
Me
Me
NH
24a
500 MHz, CDCl3
0.95
1.00
0.97
1.01
0.96
0.94
1.00
1.02
1.00
1.03
1.03
2.12
1.08
3.03
1.18
3.47
2.12
f1 (ppm)

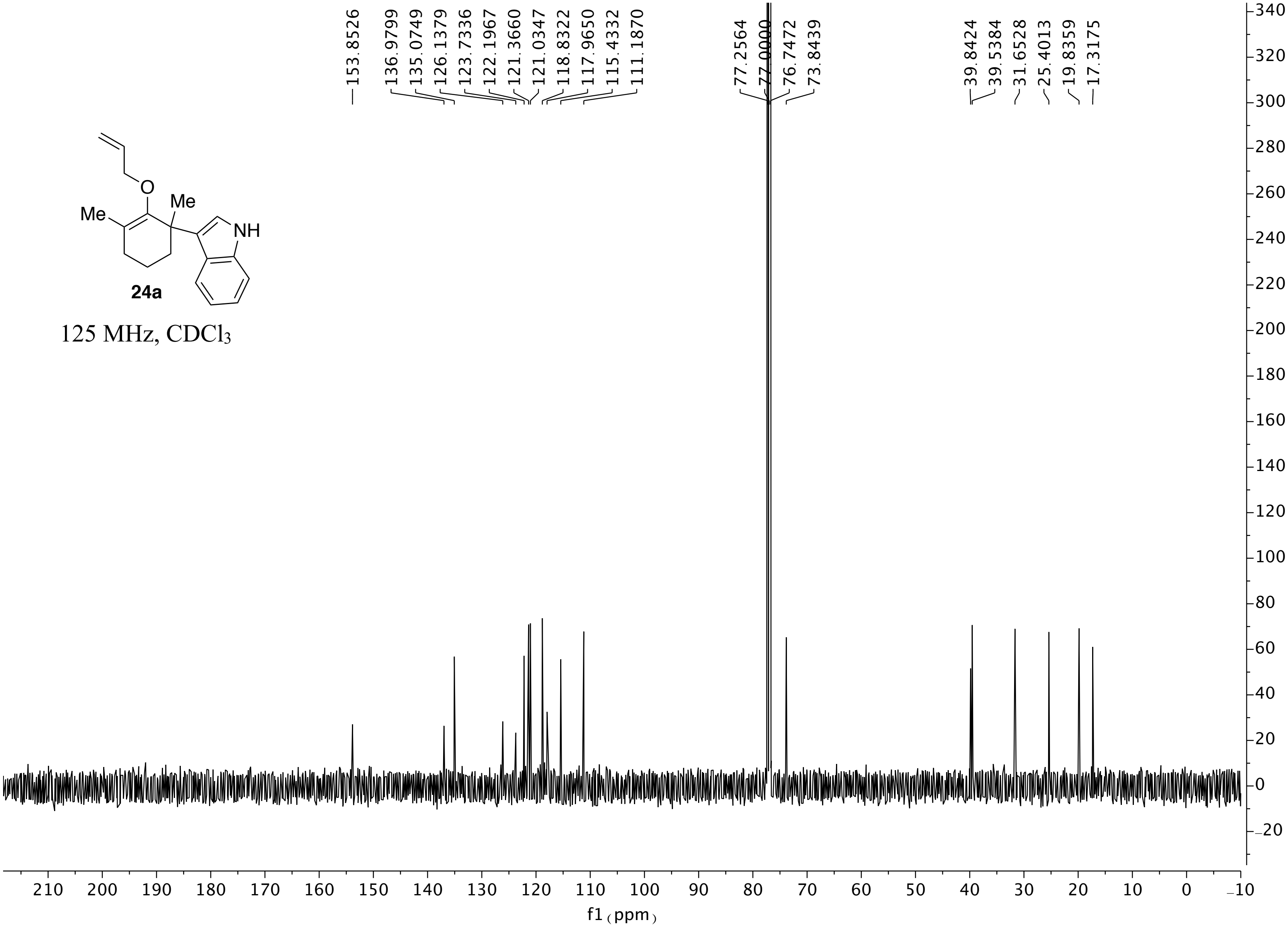

24a

125 MHz, $CDCl_3$

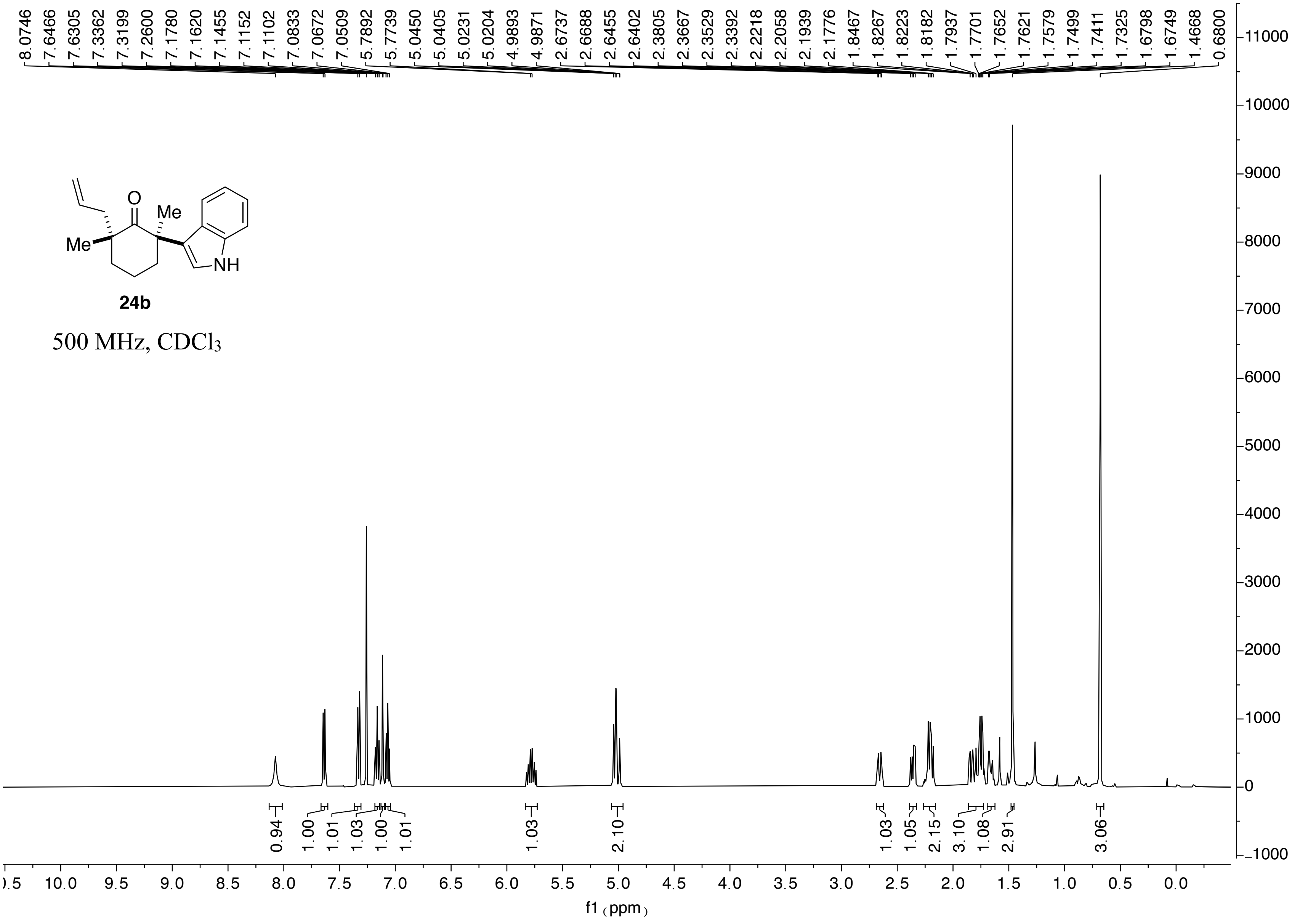
8.0746
7.6466
7.6305
7.3362
7.3199
7.2600
7.1780
7.1620
7.1455
7.1152
7.1102
7.0833
7.0672
7.0509
5.7892
5.7739
5.0450
5.0405
5.0231
5.0204
4.9893
4.9871
2.6737
2.6688
2.6455
2.6402
2.3805
2.3667
2.3529
2.3392
2.2218
2.2058
2.1939
2.1776
1.8467
1.8267
1.8223
1.8182
1.7937
1.7701
1.7652
1.7621
1.7579
1.7499
1.7411
1.7325
1.6798
1.6749
1.4668
0.6800
Me
O
Me
NH
24b
500 MHz, CDCl3
0.94
1.00
1.01
1.03
1.00
1.01
1.03
2.10
1.03
1.05
2.15
3.10
1.08
2.91
3.06
f1 (ppm)

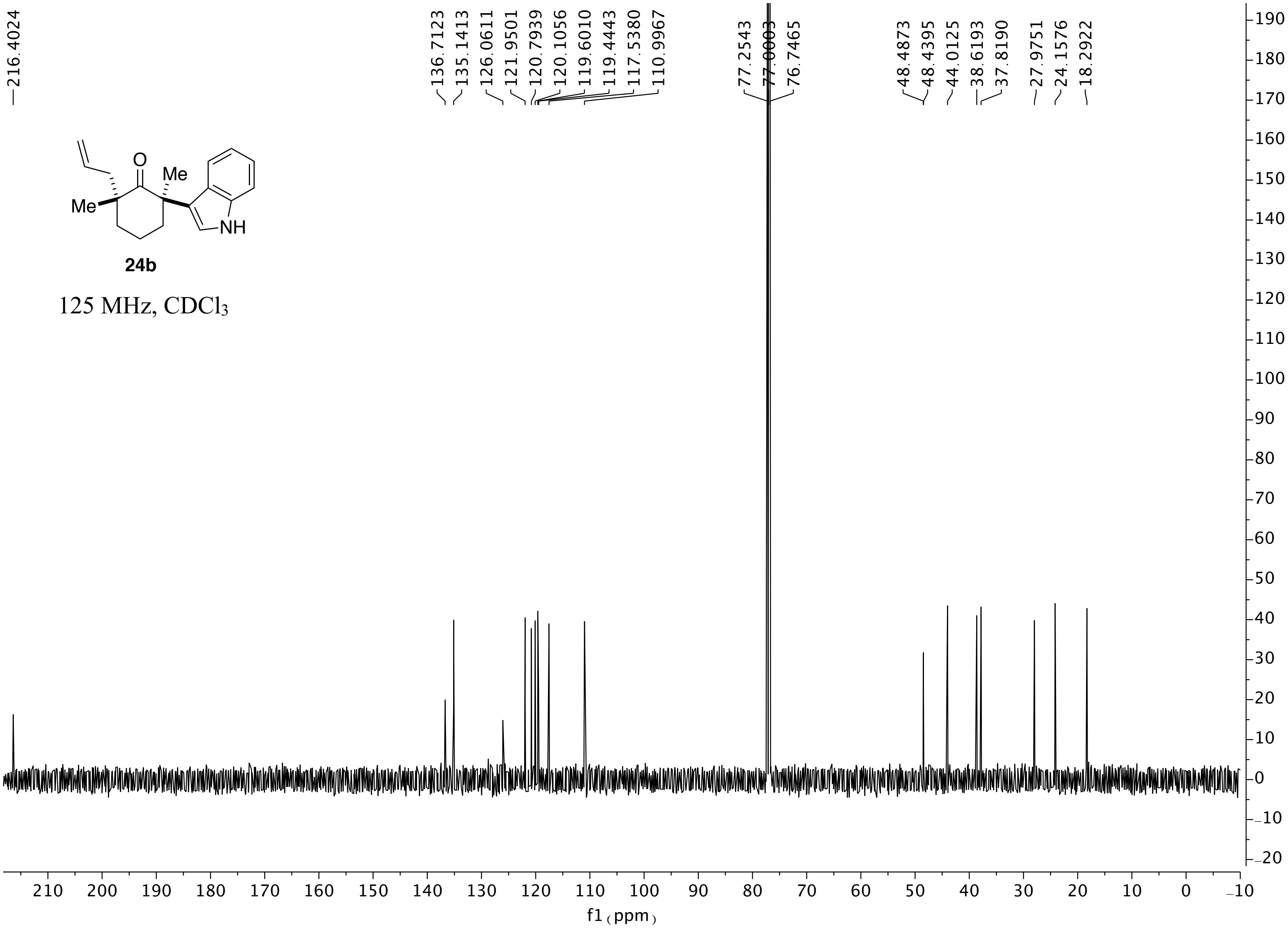

24b

125 MHz, $CDCl_3$

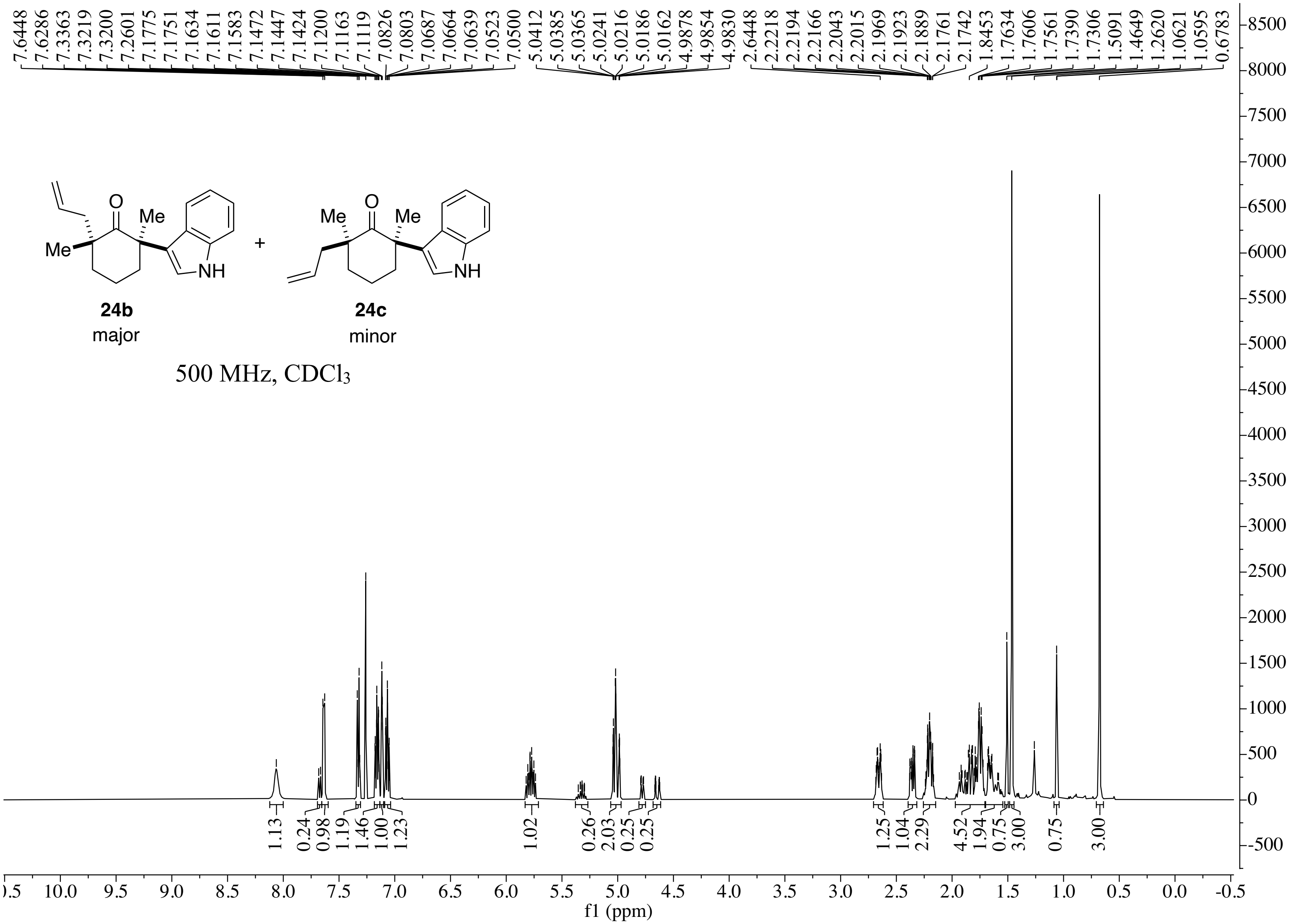
24b
major
24c
minor
500 MHz, CDCl$_3$
f1 (ppm)

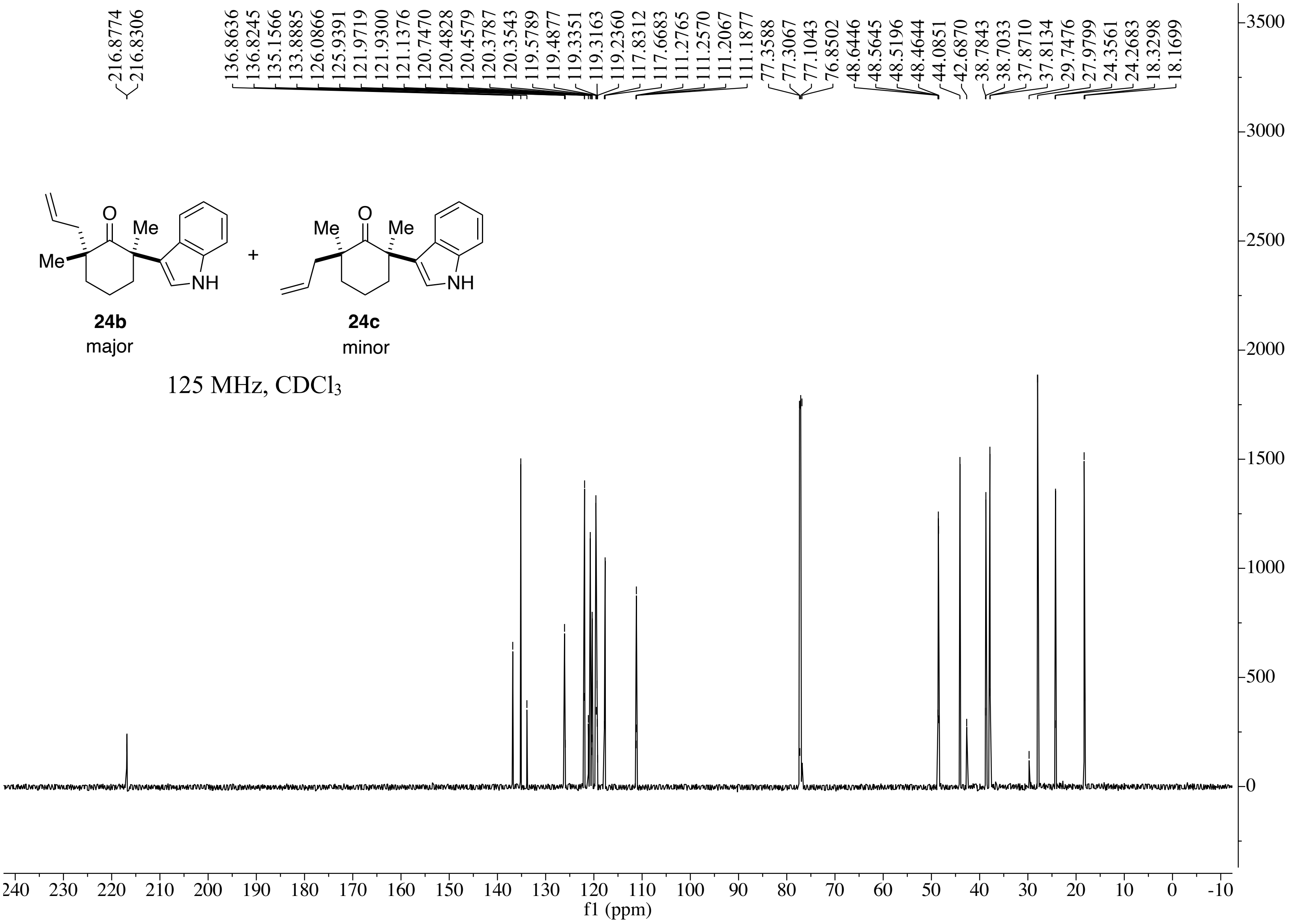
216.8774
216.8306
136.8636
136.8245
135.1566
133.8885
126.0866
125.9391
121.9719
121.9300
121.1376
120.7470
120.4828
120.4579
120.3787
120.3543
119.5789
119.4877
119.3351
119.3163
119.2360
117.8312
117.6683
111.2765
111.2570
111.2067
111.1877
77.3588
77.3067
77.1043
76.8502
48.6446
48.5645
48.5196
48.4644
44.0851
42.6870
38.7843
38.7033
37.8710
37.8134
29.7476
27.9799
24.3561
24.2683
18.3298
18.1699
Me
O
Me
NH
+
Me
O
Me
NH
24b
major
24c
minor
125 MHz, CDCl3
f1 (ppm)

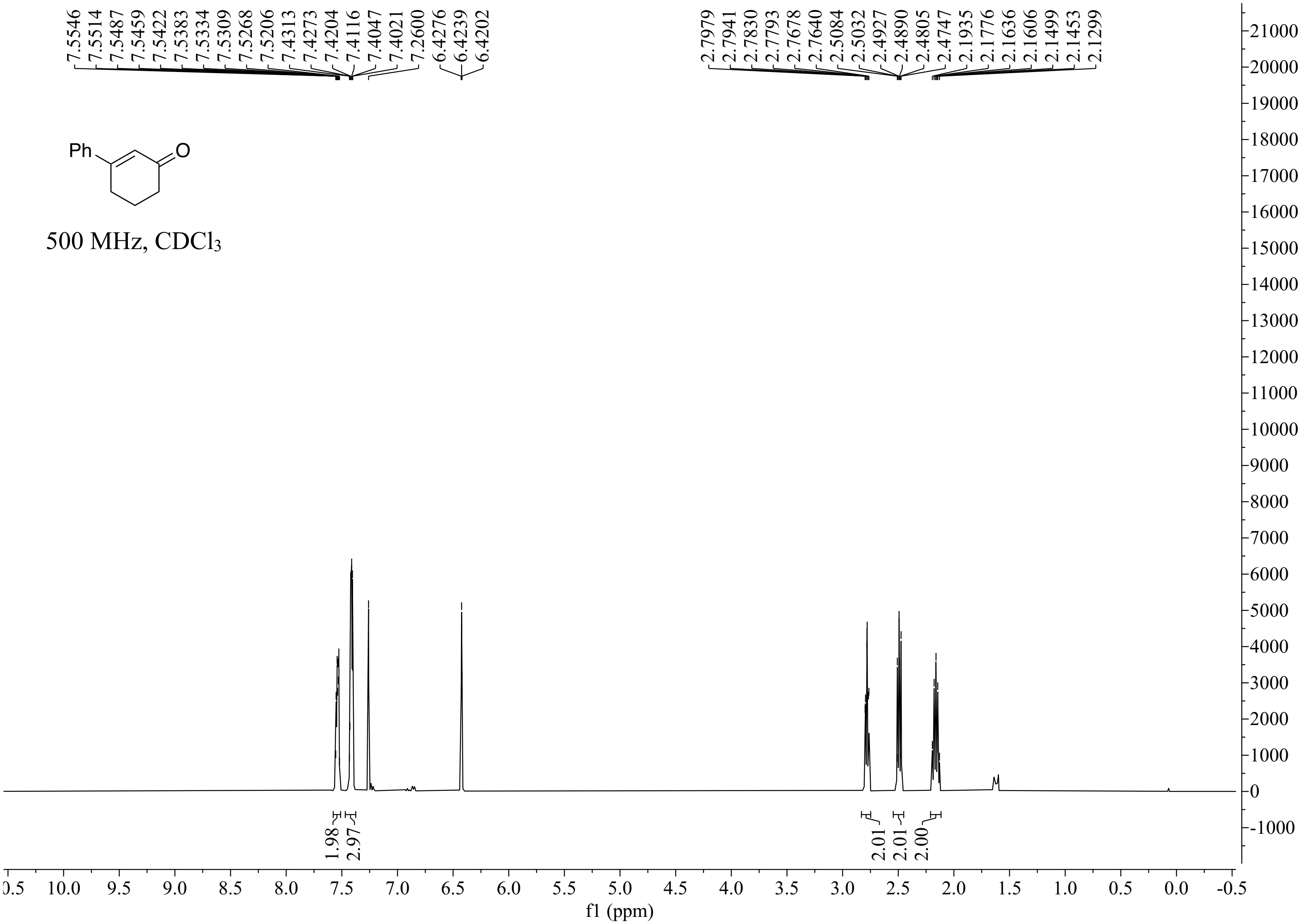

500 MHz, $CDCl_3$

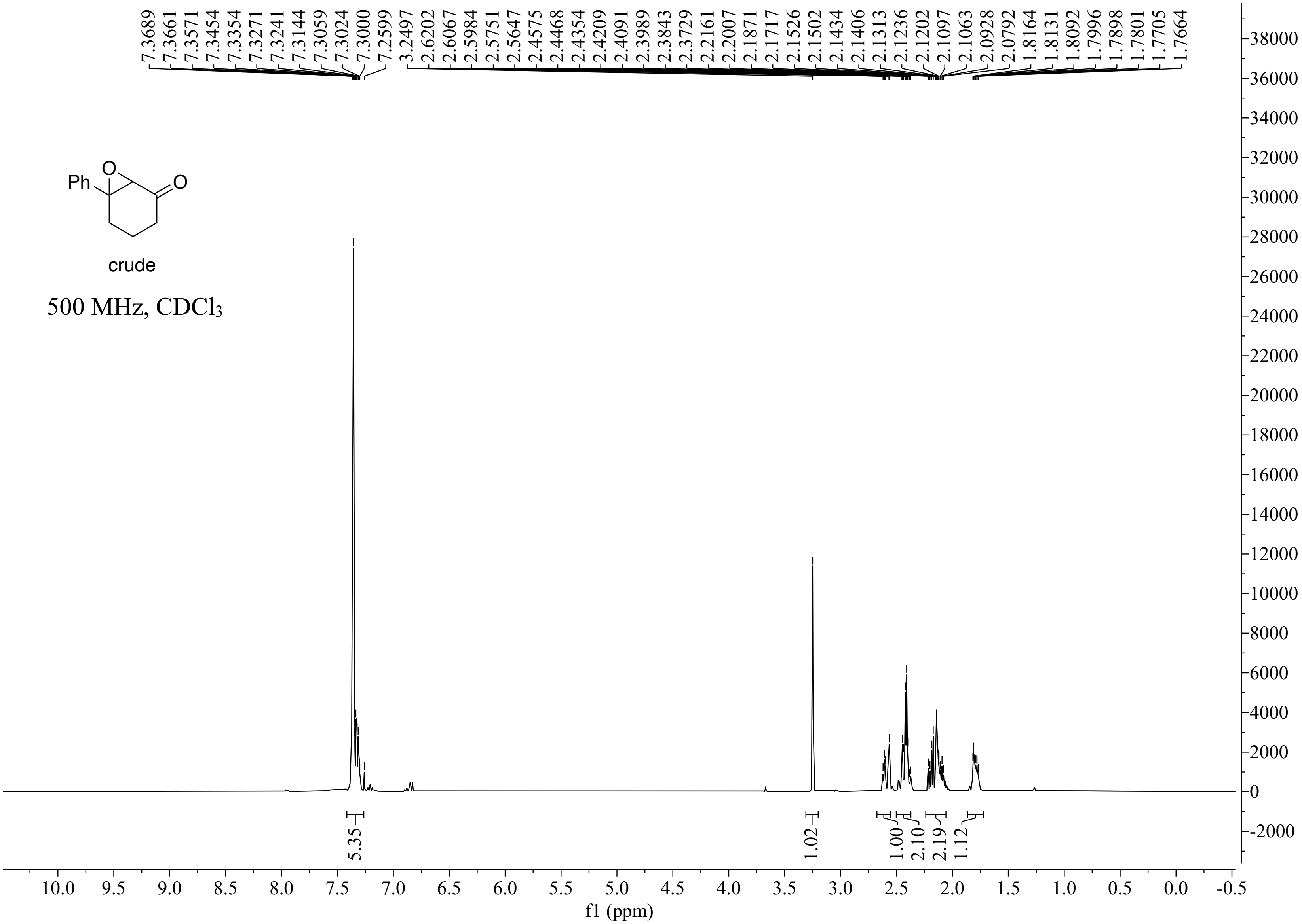
Ph
O
O
crude
500 MHz, CDCl3
f1 (ppm)

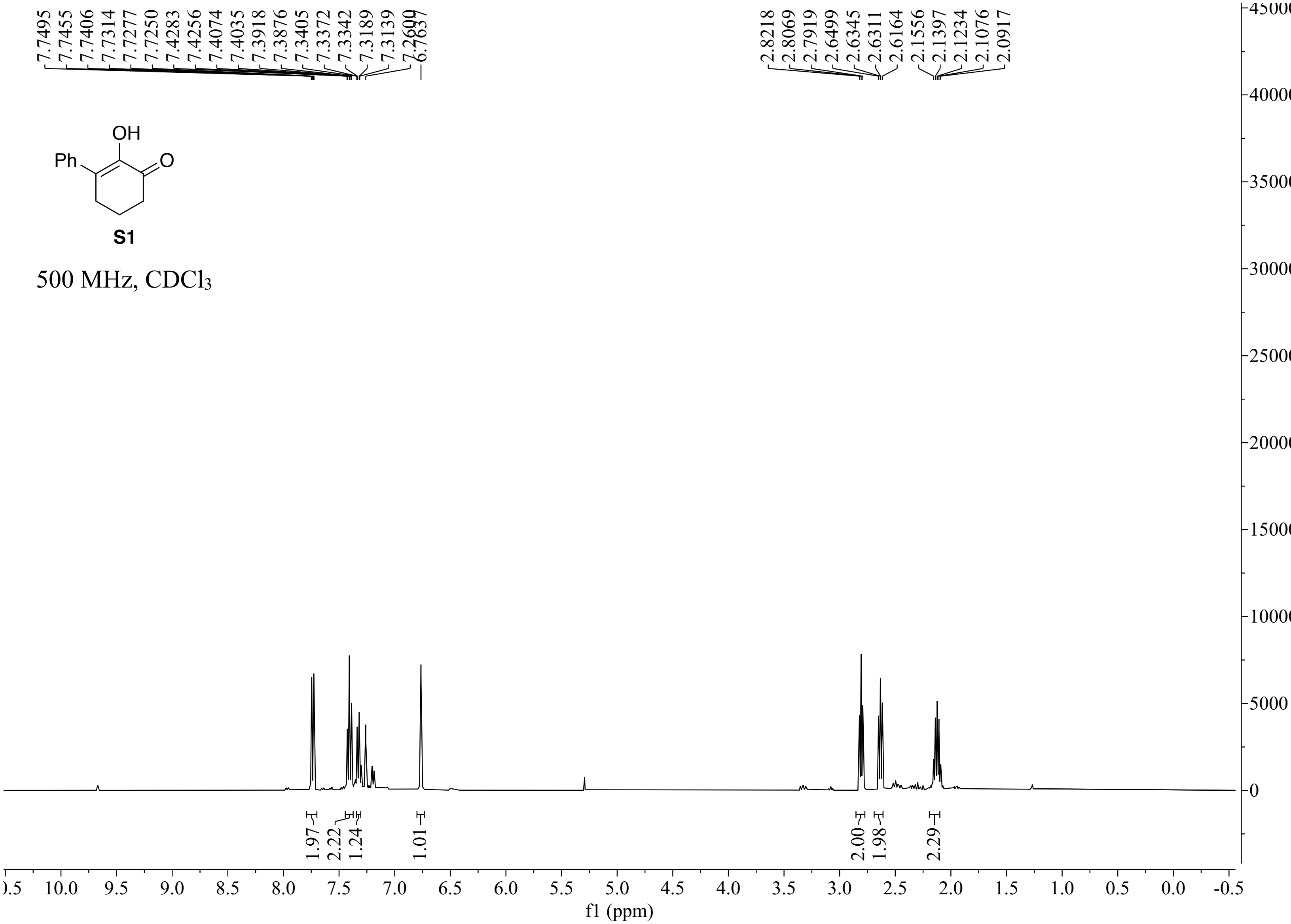
7.7495
7.7455
7.7406
7.7314
7.7277
7.7250
7.4283
7.4256
7.4074
7.4035
7.3918
7.3876
7.3405
7.3372
7.3342
7.3189
7.3139
7.2600
6.7637
2.8218
2.8069
2.7919
2.6499
2.6345
2.6311
2.6164
2.1556
2.1397
2.1234
2.1076
2.0917
OH
Ph
O
S1
500 MHz, CDCl3
1.97
2.22
1.24
1.01
2.00
1.98
2.29
f1 (ppm)

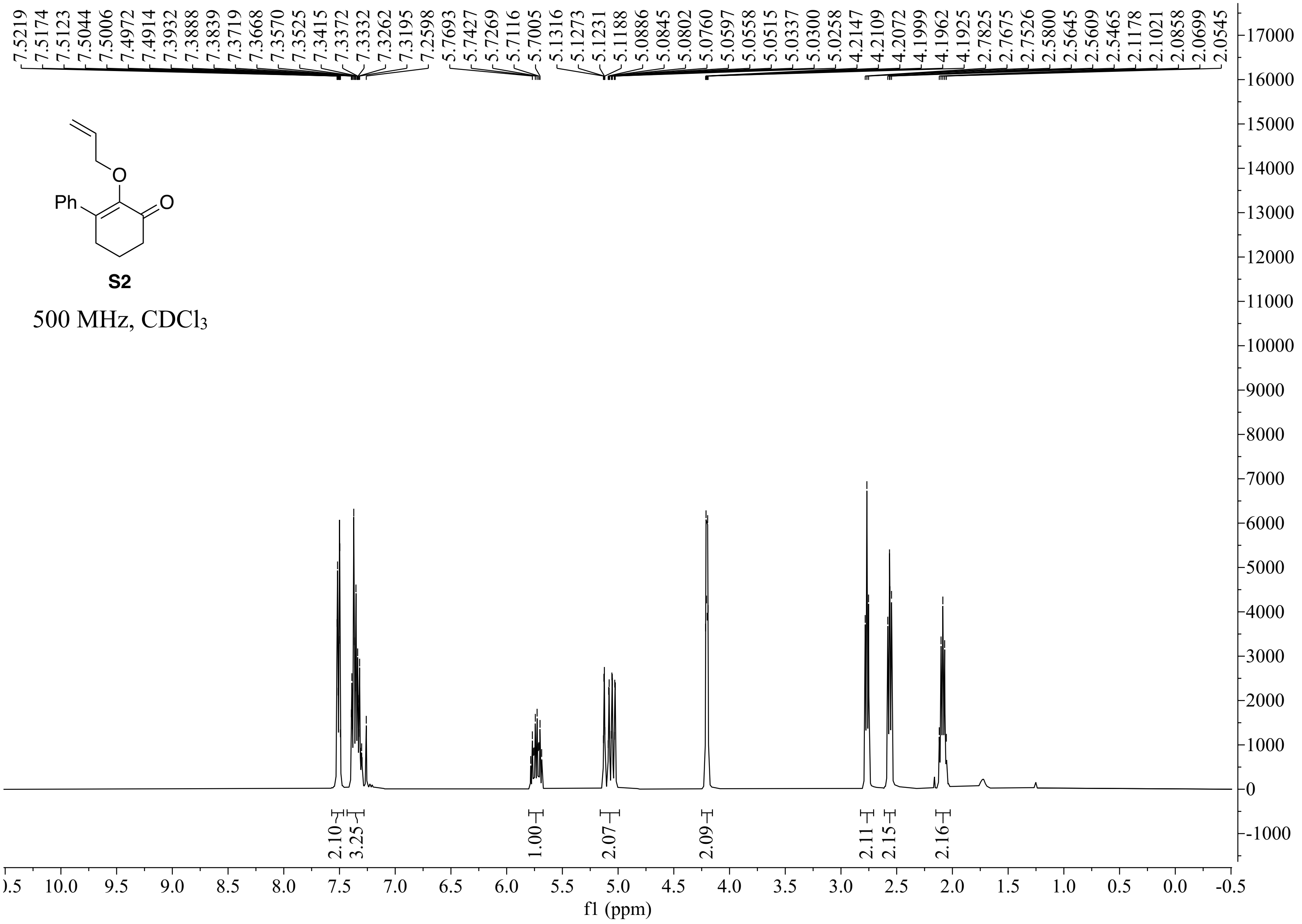
7.5219
7.5174
7.5123
7.5044
7.5006
7.4972
7.4914
7.3932
7.3888
7.3839
7.3719
7.3668
7.3570
7.3525
7.3415
7.3372
7.3332
7.3262
7.3195
7.2598
5.7693
5.7427
5.7269
5.7116
5.7005
5.1316
5.1273
5.1231
5.1188
5.0886
5.0845
5.0802
5.0760
5.0597
5.0558
5.0515
5.0337
5.0300
5.0258
4.2147
4.2109
4.2072
4.1999
4.1962
4.1925
2.7825
2.7675
2.7526
2.5800
2.5645
2.5609
2.5465
2.1178
2.1021
2.0858
2.0699
2.0545
Ph
O
O
S2
500 MHz, CDCl3
2.10
3.25
1.00
2.07
2.09
2.11
2.15
2.16
f1 (ppm)

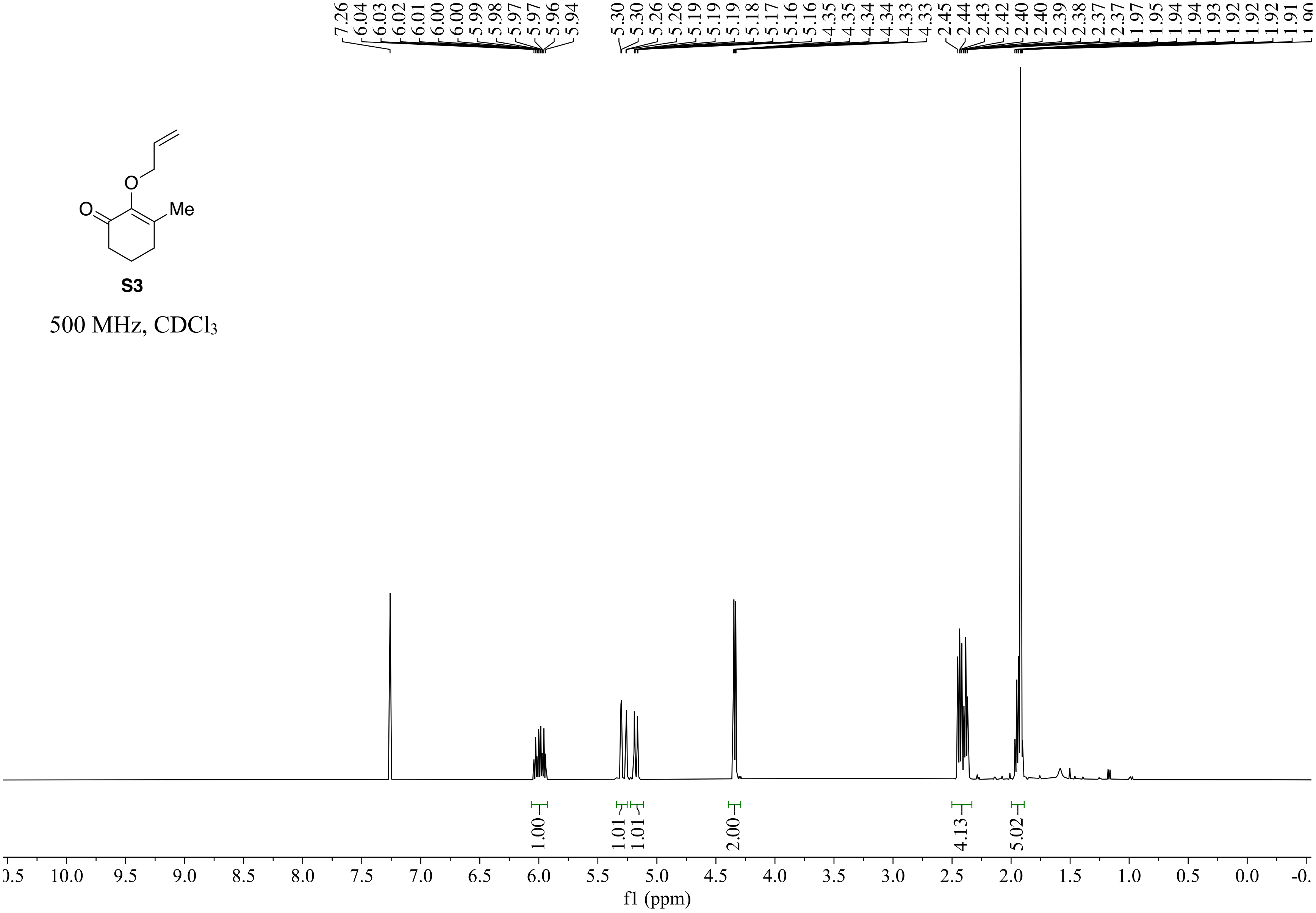

S3

500 MHz, $CDCl_3$

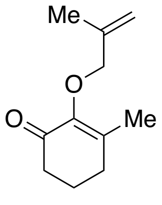

S4

500 MHz, $CDCl_3$

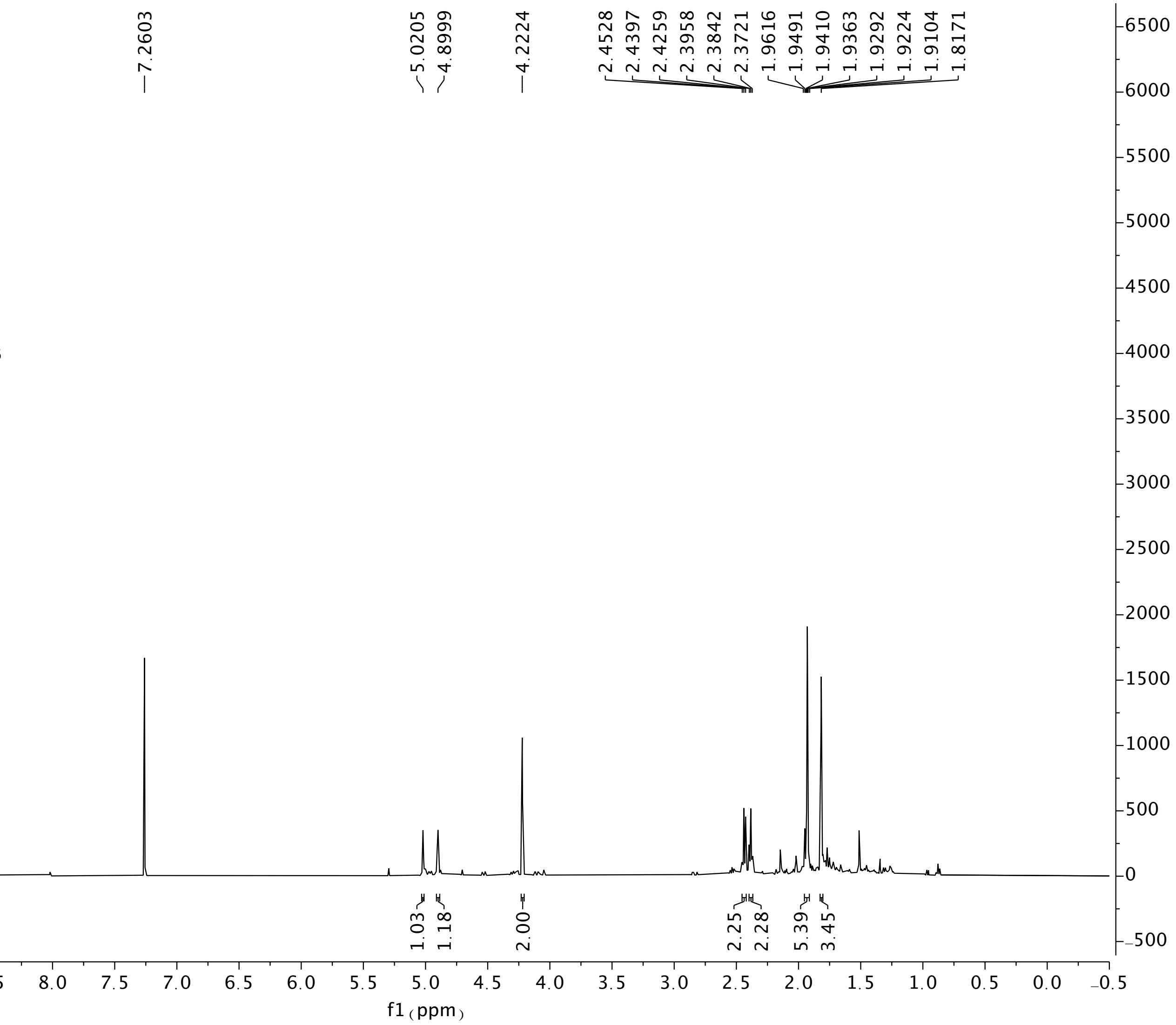

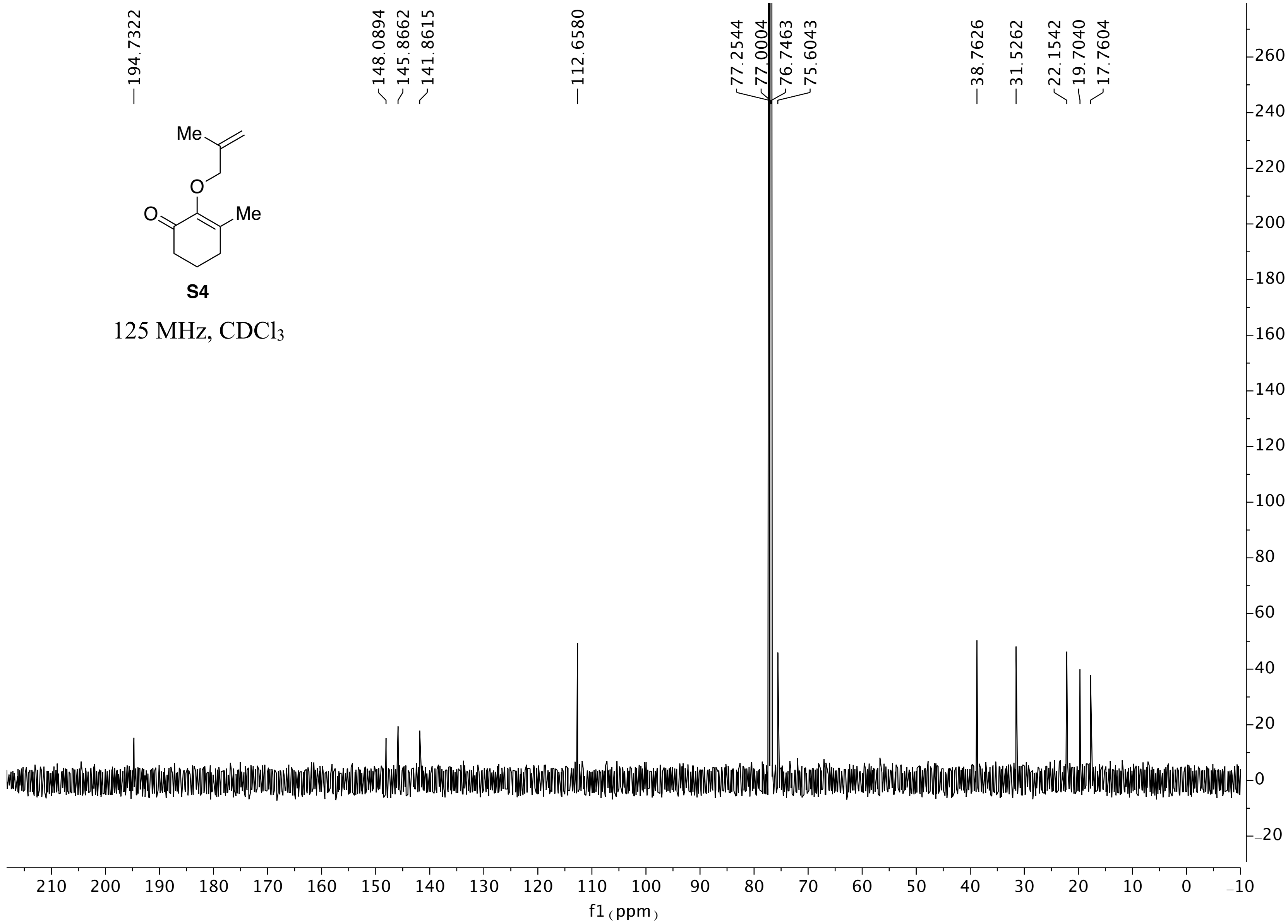

194.7322
148.0894
145.8662
141.8615
112.6580
77.2544
77.0004
76.7463
75.6043
38.7626
31.5262
22.1542
19.7040
17.7604
Me
O
O
Me
S4
125 MHz, CDCl3
f1 (ppm)

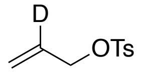

S6

500 MHz, $CDCl_3$

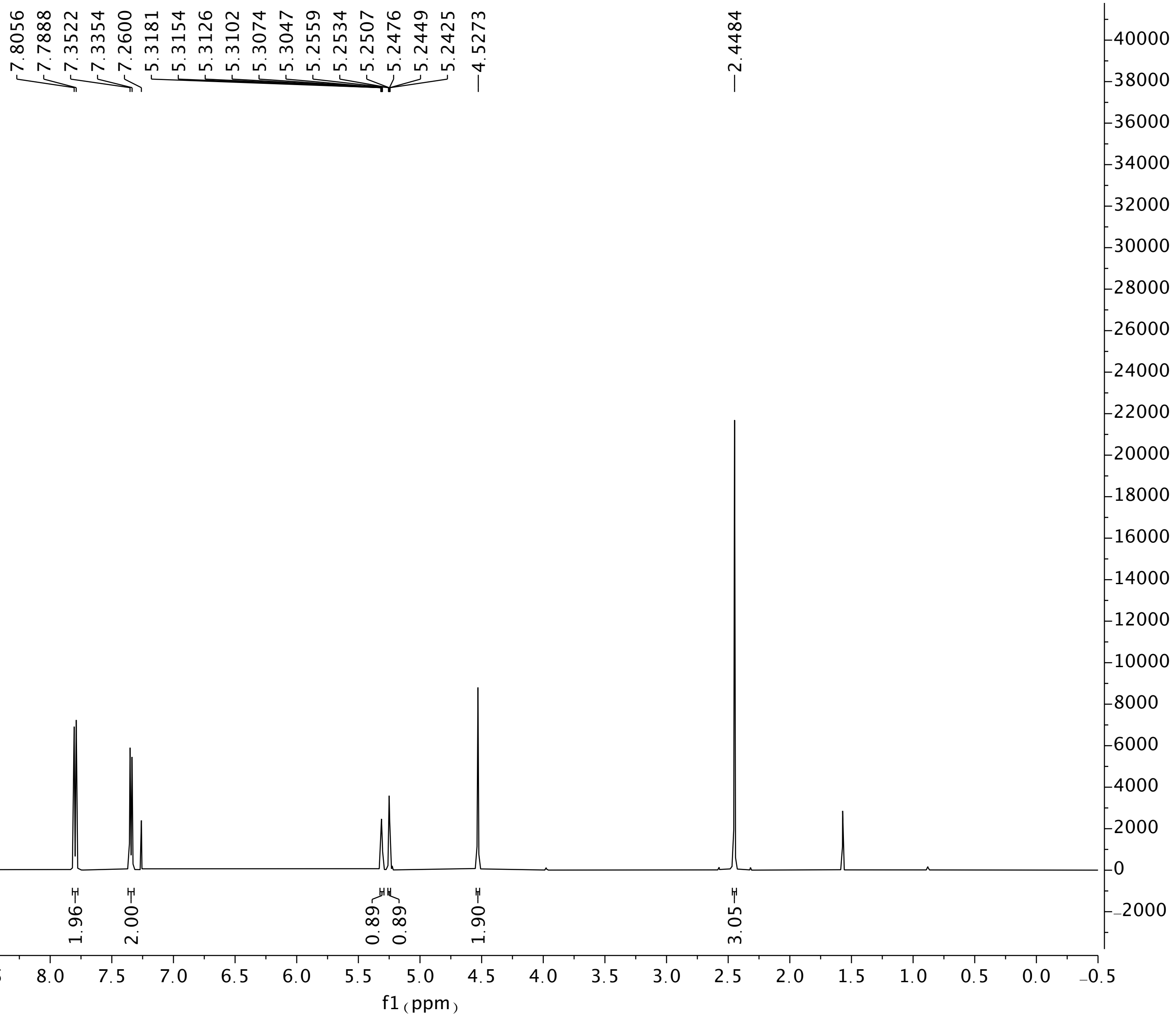

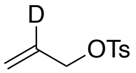

S6

125 MHz, $CDCl_3$

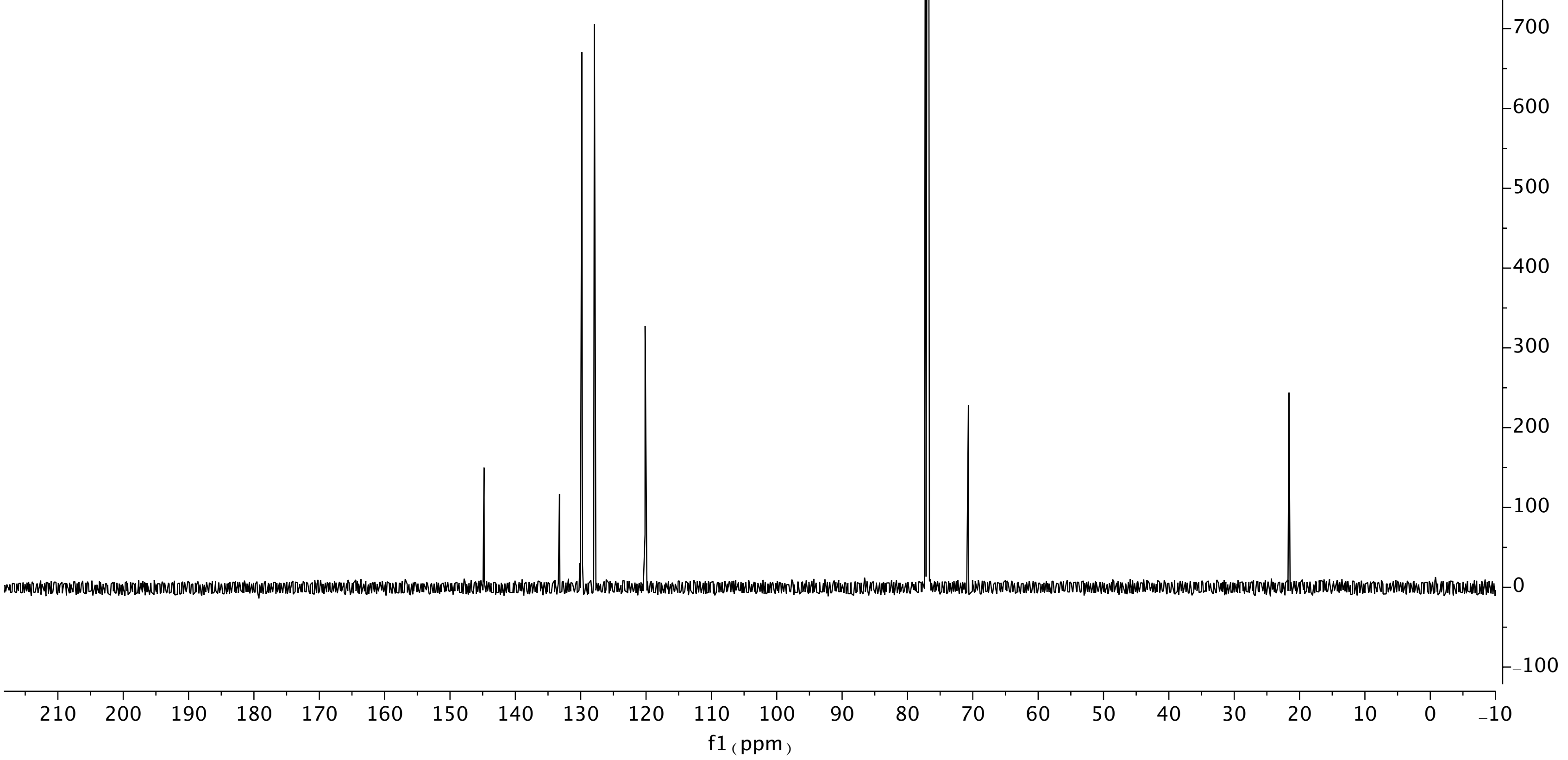

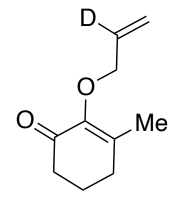

S7

500 MHz, $CDCl_3$

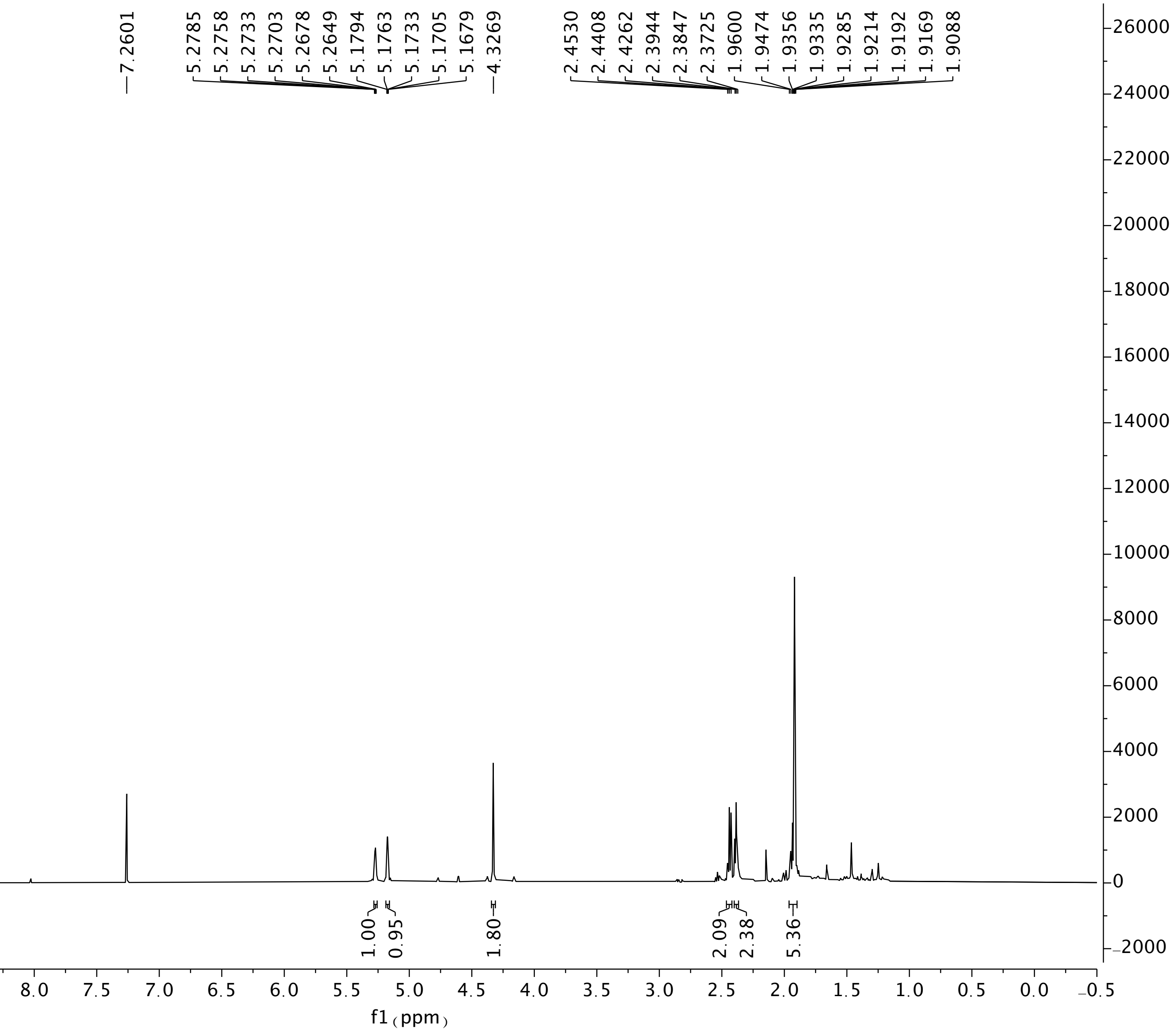

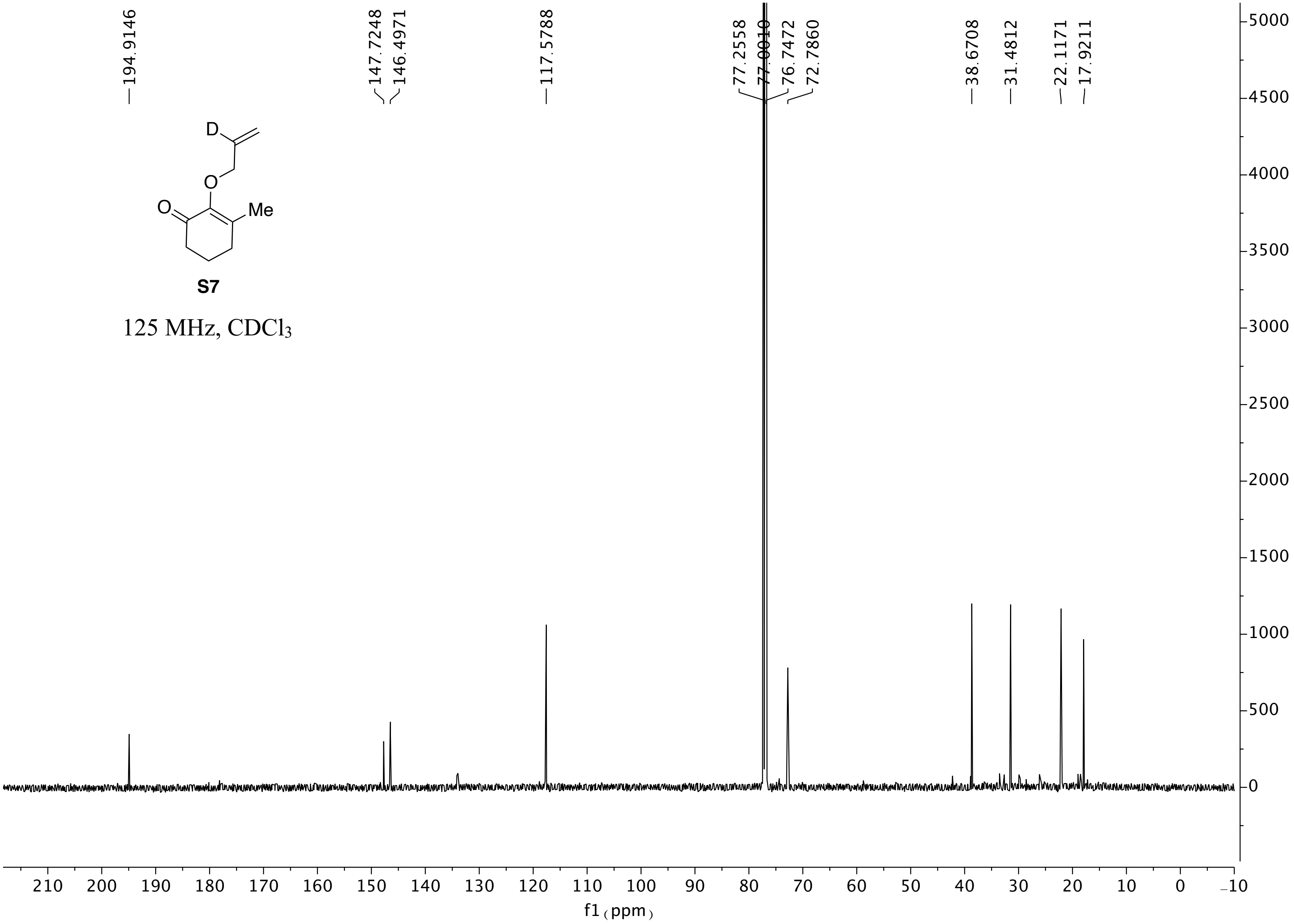
194.9146
147.7248
146.4971
117.5788
77.2558
77.0010
76.7472
72.7860
38.6708
31.4812
22.1171
17.9211
D
O
O
Me
S7
125 MHz, $CDCl_3$
5000
4500
4000
3500
3000
2500
2000
1500
1000
500
0
210 200 190 180 170 160 150 140 130 120 110 100 90 80 70 60 50 40 30 20 10 0 -10
f1 (ppm)

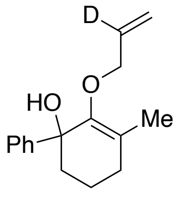

S8

500 MHz, $CDCl_3$

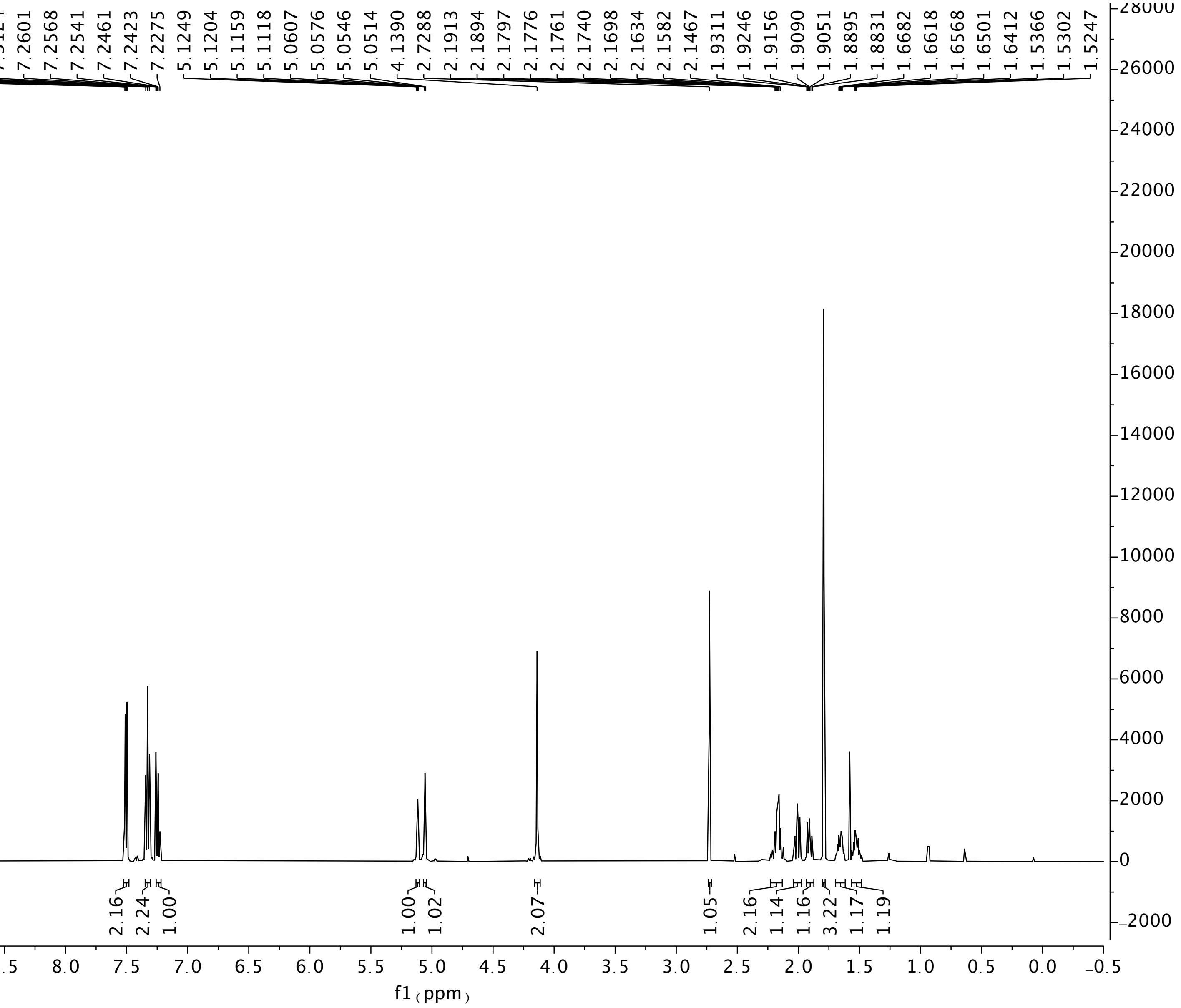

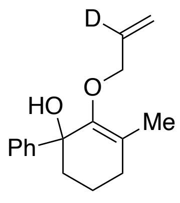

S8

125 MHz, $CDCl_3$

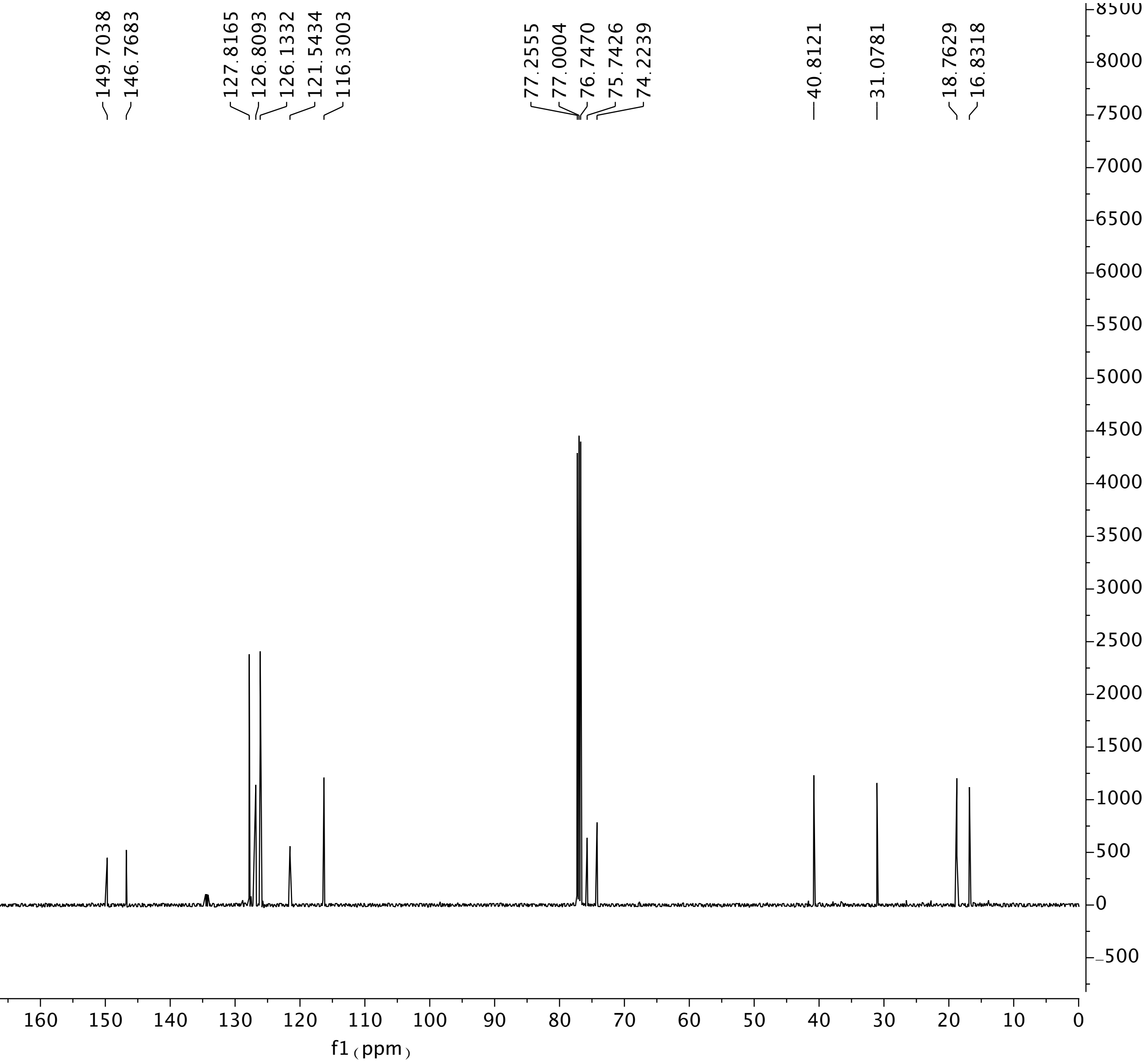

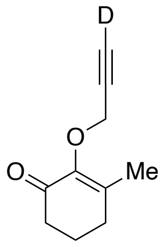

S9

500 MHz, $CDCl_3$

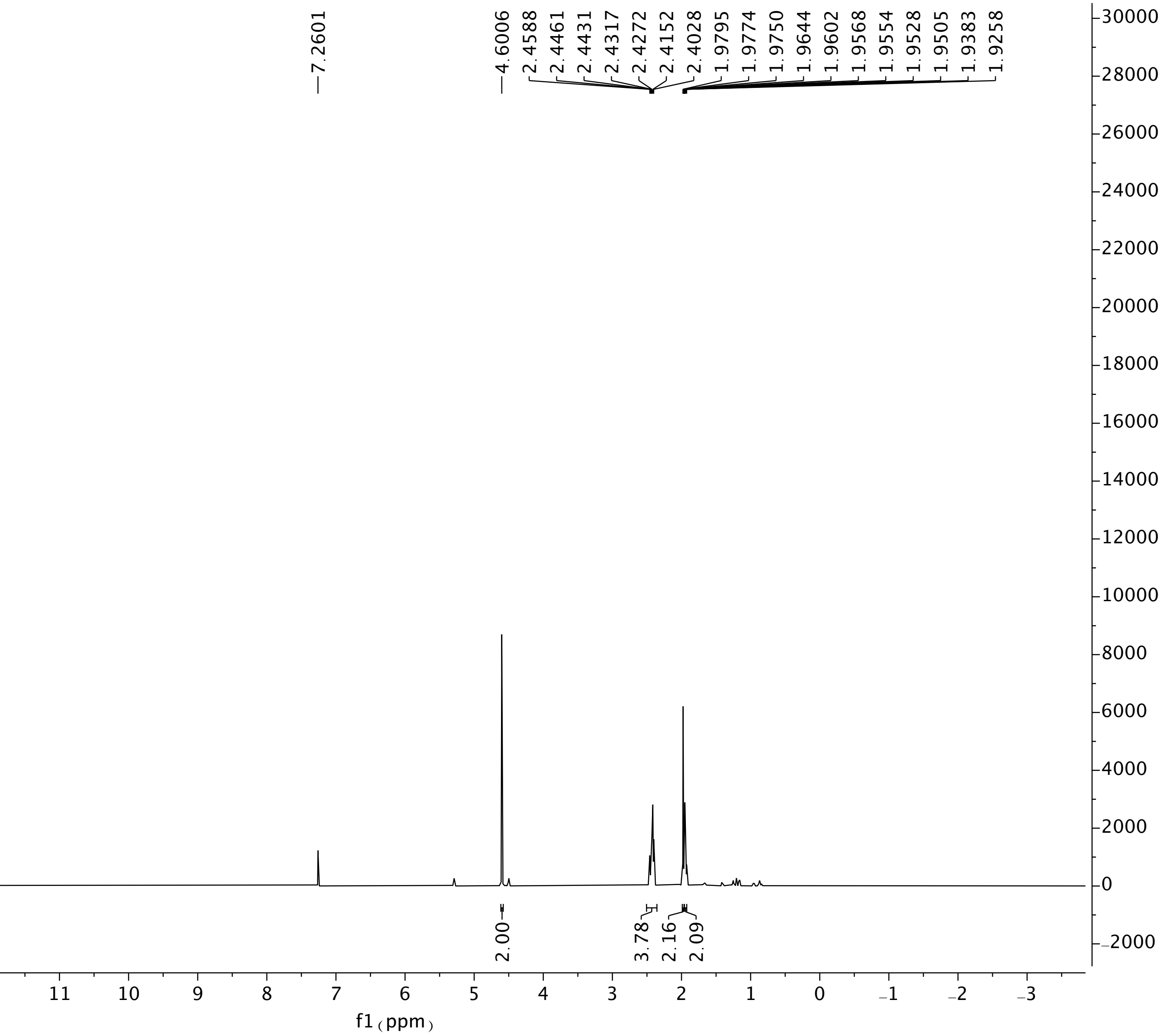

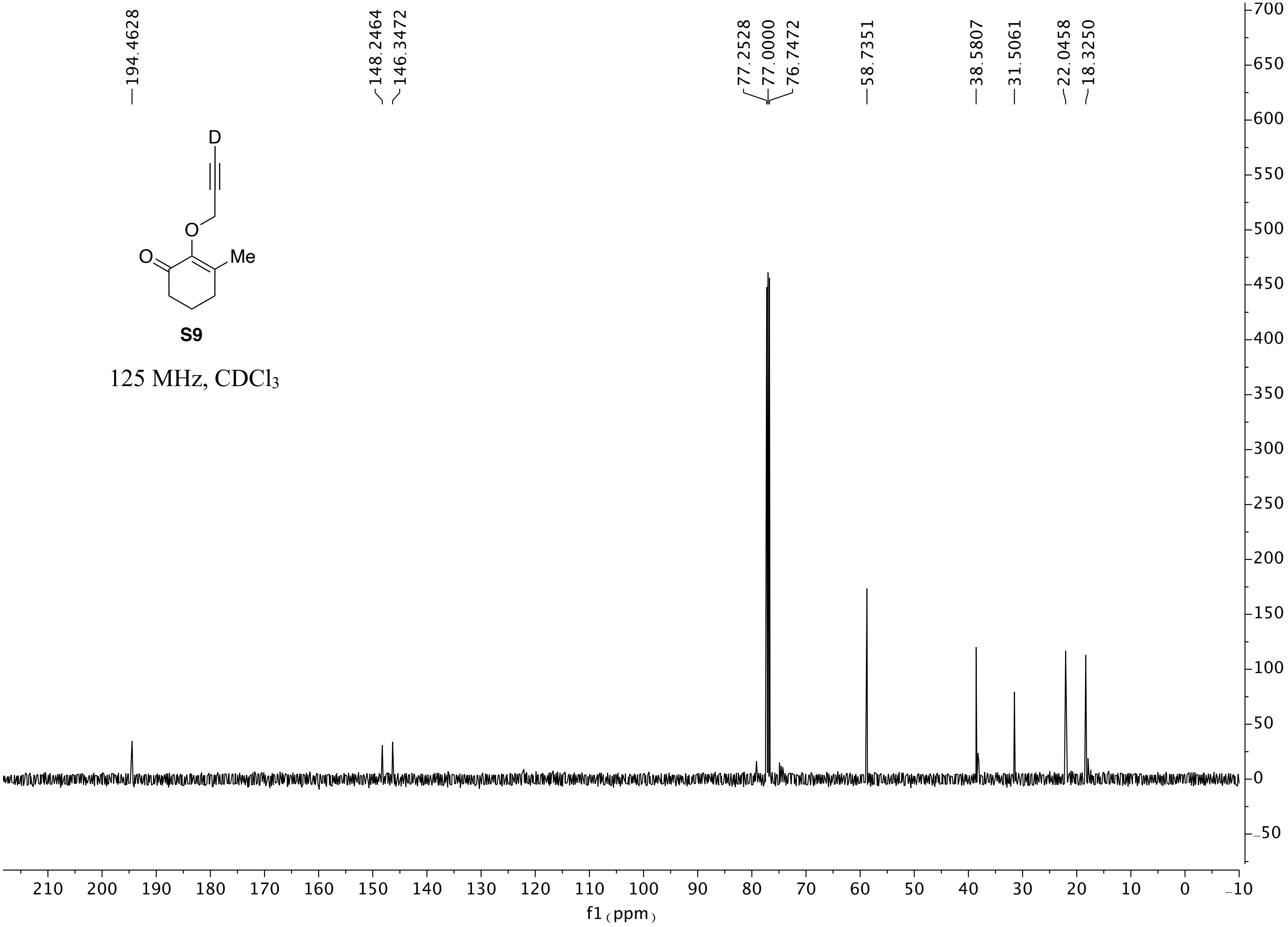
194.4628
148.2464
146.3472
77.2528
77.0000
76.7472
58.7351
38.5807
31.5061
22.0458
18.3250
D
O
O
Me
S9
125 MHz, CDCl3
700
650
600
550
500
450
400
350
300
250
200
150
100
50
0
-50
210 200 190 180 170 160 150 140 130 120 110 100 90 80 70 60 50 40 30 20 10 0 -10
f1 (ppm)

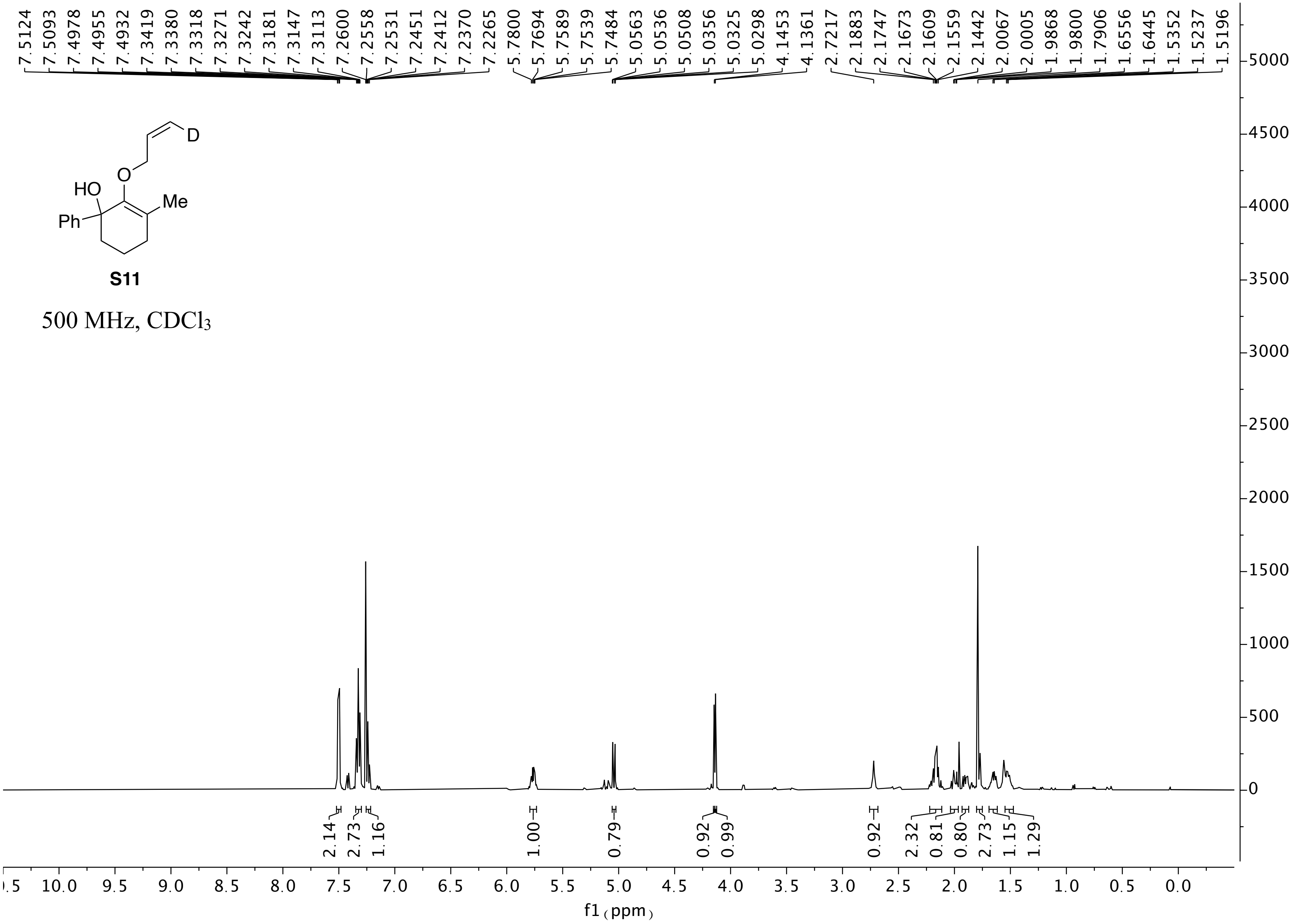
7.5124
7.5093
7.4978
7.4955
7.4932
7.3419
7.3380
7.3318
7.3271
7.3242
7.3181
7.3147
7.3113
7.2600
7.2558
7.2531
7.2451
7.2412
7.2370
7.2265
5.7800
5.7694
5.7589
5.7539
5.7484
5.0563
5.0536
5.0508
5.0356
5.0325
5.0298
4.1453
4.1361
2.7217
2.1883
2.1747
2.1673
2.1609
2.1559
2.1442
2.0067
2.0005
1.9868
1.9800
1.7906
1.6556
1.6445
1.5352
1.5237
1.5196
HO
O
D
Me
Ph
S11
500 MHz, $CDCl_3$
2.14
2.73
1.16
1.00
0.79
0.92
0.99
0.92
2.32
0.81
0.80
2.73
1.15
1.29
f1 (ppm)

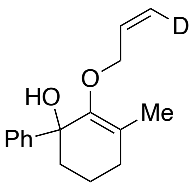

S11

125 MHz, $CDCl_3$

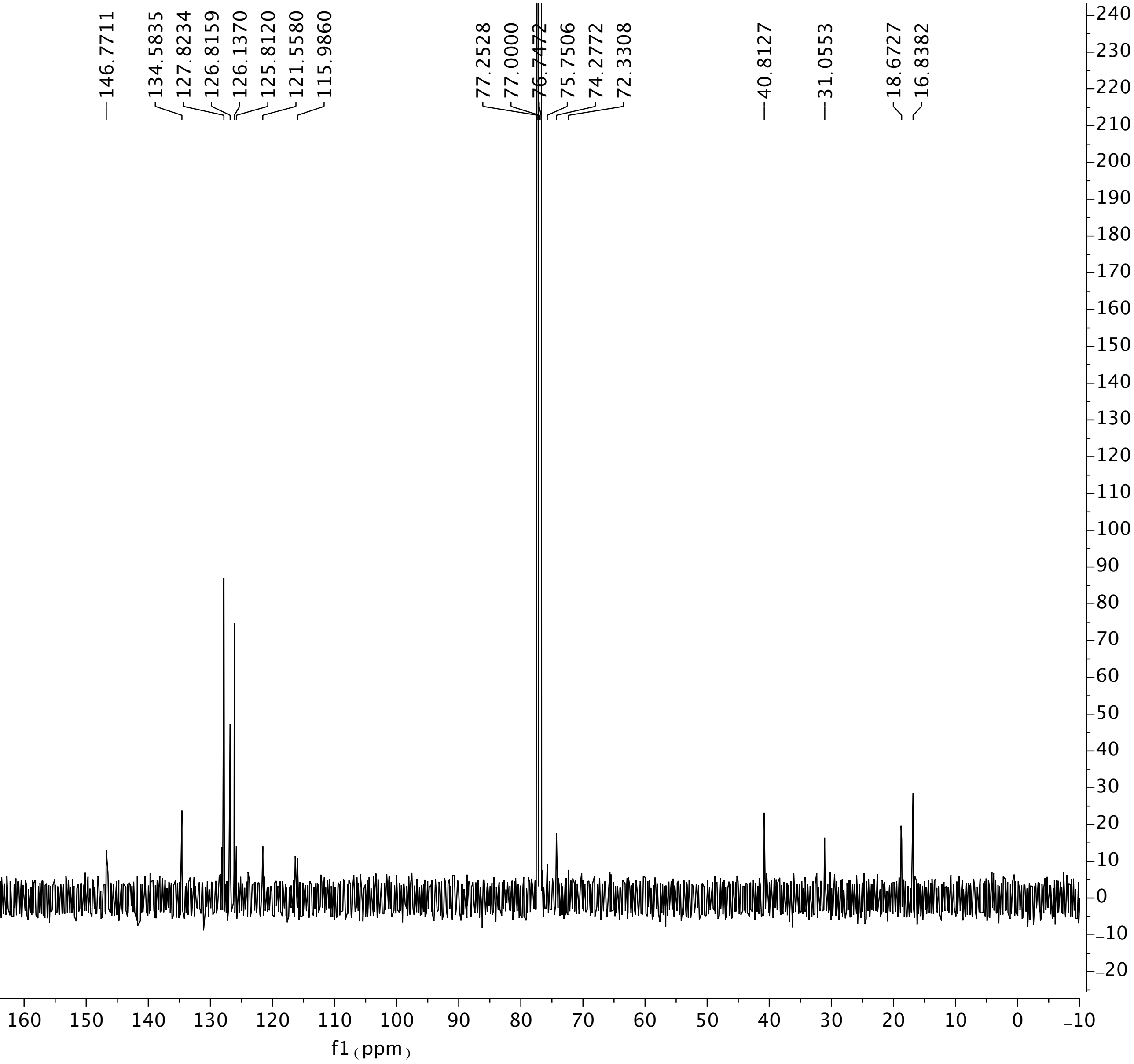

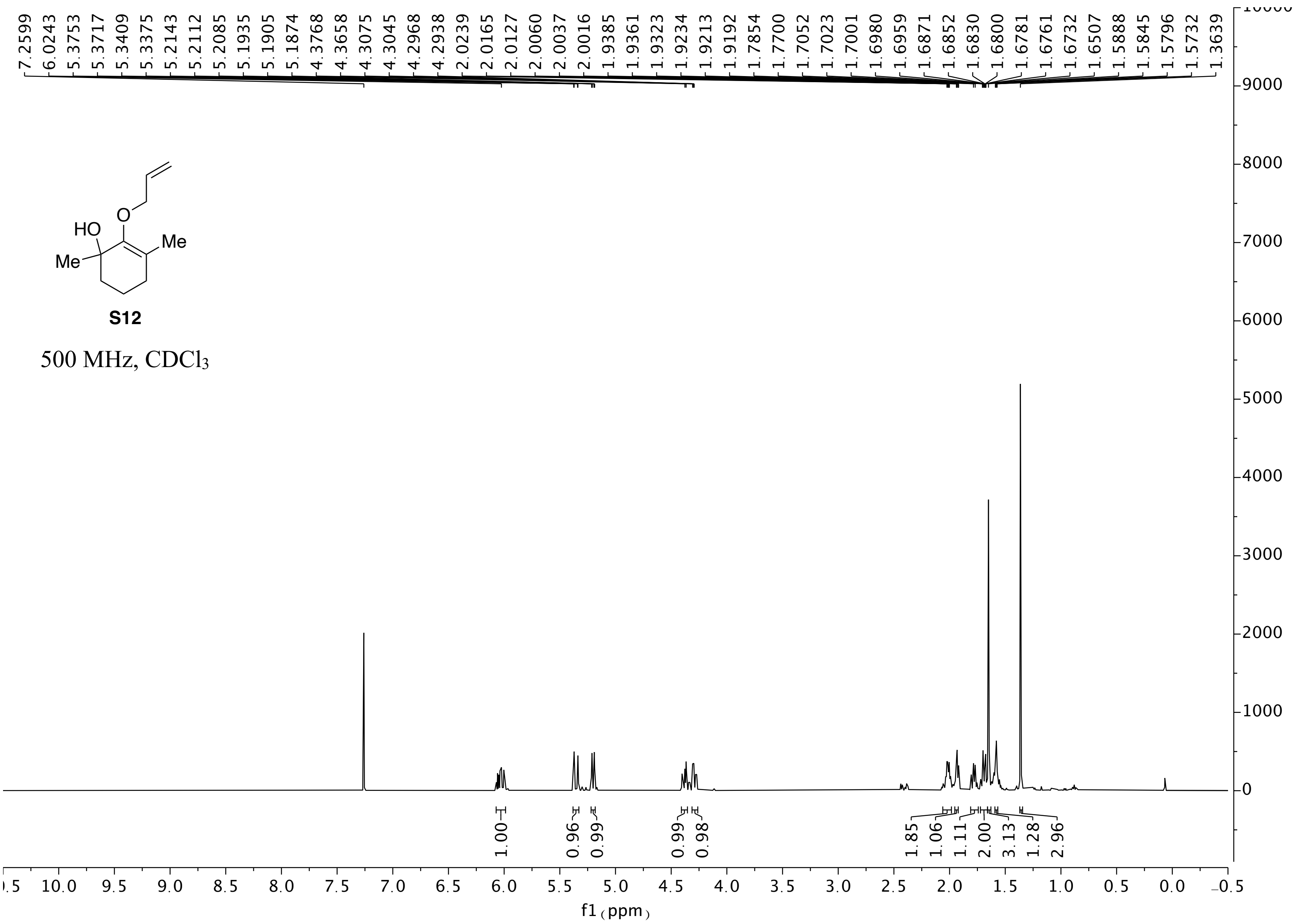

7.2599
6.0243
5.3753
5.3717
5.3409
5.3375
5.2143
5.2112
5.2085
5.1935
5.1905
5.1874
4.3768
4.3658
4.3075
4.3045
4.2968
4.2938
2.0239
2.0165
2.0127
2.0060
2.0037
2.0016
1.9385
1.9361
1.9323
1.9234
1.9213
1.9192
1.7854
1.7700
1.7052
1.7023
1.7001
1.6980
1.6959
1.6871
1.6852
1.6830
1.6800
1.6781
1.6761
1.6732
1.6507
1.5888
1.5845
1.5796
1.5732
1.3639
HO
O
Me
Me
S12
500 MHz, CDCl3
1.00
0.96
0.99
0.99
0.98
1.85
1.06
1.11
2.00
3.13
1.28
2.96
f1 (ppm)

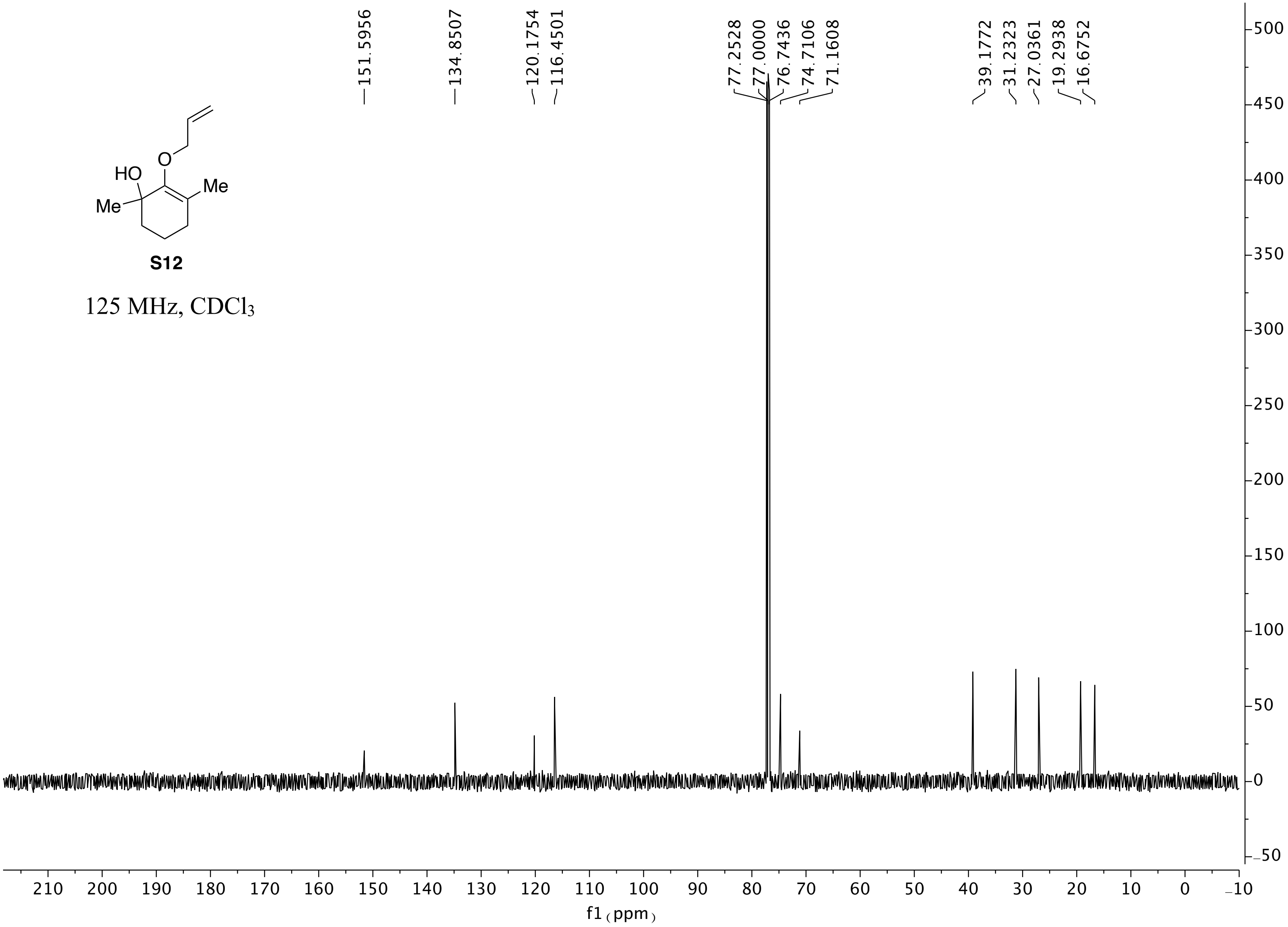
HO
O
Me
Me
S12
125 MHz, CDCl3
151.5956
134.8507
120.1754
116.4501
77.2528
77.0000
76.7436
74.7106
71.1608
39.1772
31.2323
27.0361
19.2938
16.6752
f1 (ppm)

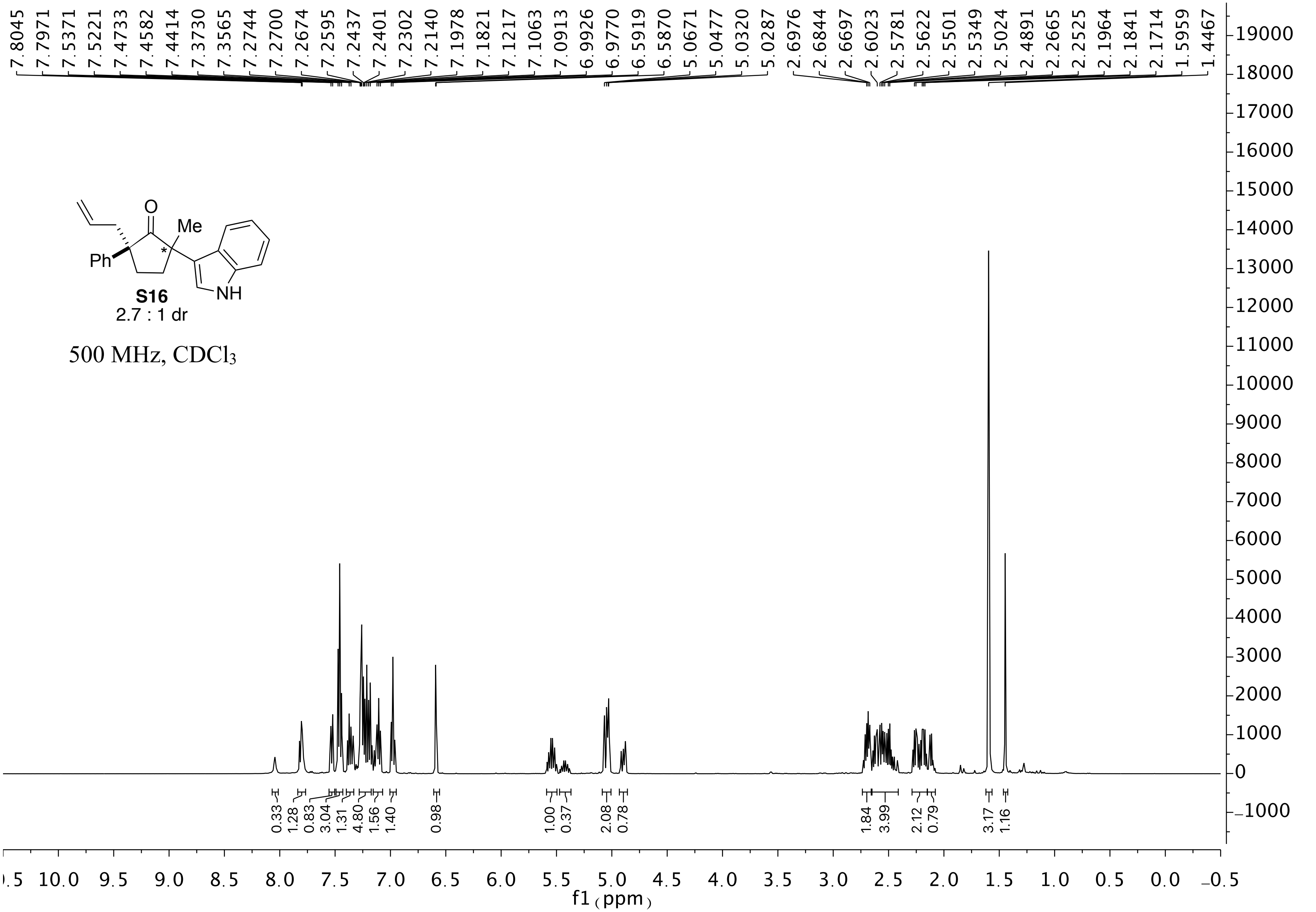
7.8045
7.7971
7.5371
7.5221
7.4733
7.4582
7.4414
7.3730
7.3565
7.2744
7.2700
7.2674
7.2595
7.2437
7.2401
7.2302
7.2140
7.1978
7.1821
7.1217
7.1063
7.0913
6.9926
6.9770
6.5919
6.5870
5.0671
5.0477
5.0320
5.0287
2.6976
2.6844
2.6697
2.6023
2.5781
2.5622
2.5501
2.5349
2.5024
2.4891
2.2665
2.2525
2.1964
2.1841
2.1714
1.5959
1.4467
O
Me
Ph
*
NH
S16
2.7 : 1 dr
500 MHz, CDCl3
0.33
1.28
0.83
3.04
1.31
4.80
1.56
1.40
0.98
1.00
0.37
2.08
0.78
1.84
3.99
2.12
0.79
3.17
1.16
f1 (ppm)

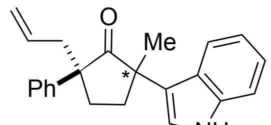

S16
2.7 : 1 dr

125 MHz, $CDCl_3$

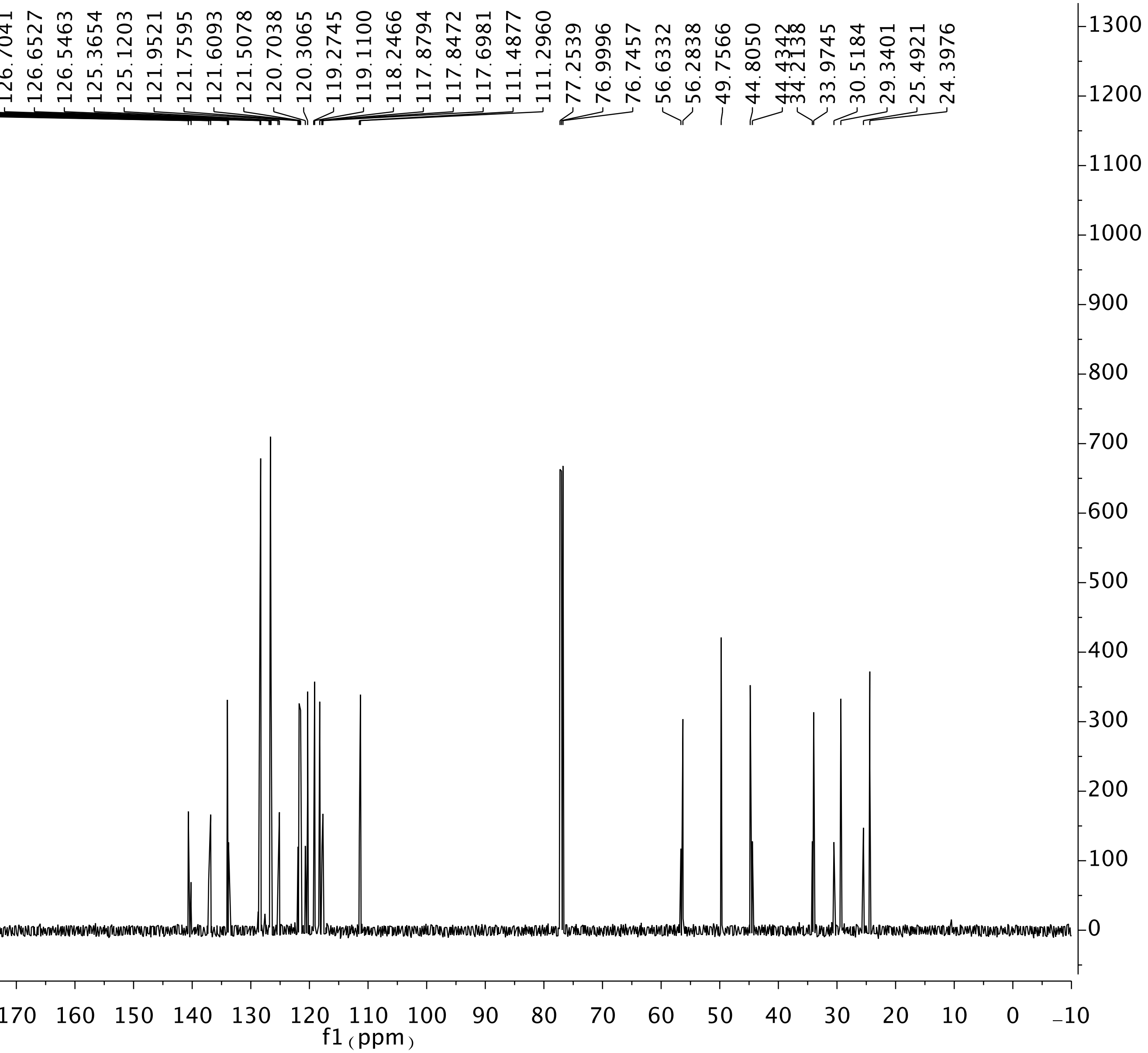

Supplement: Supplementary file 2 — ol2c01890_si_002.pdf [file ol2c01890_si_002.pdf]
